# Supplementary material for: Turning sulfonyl and sulfonimidoyl fluoride electrophiles into sulfur(VI) radicals for alkene ligation
Source: Nat Commun. 2023 Aug 24;14:5168. doi: 10.1038/s41467-023-40615-0 (PMC10449886; doi:10.1038/s41467-023-40615-0)
Supplement: Supplementary file 1 — Supplementary Information [file 41467_2023_40615_MOESM1_ESM.pdf]

SUPPLEMENTARY INFORMATION

Turning Sulfonyl and Sulfonimidoyl Fluoride Electrophiles into Sulfur(VI) Radicals for Alkene Ligation

Xing Wu[1,+], Wenbo Zhang[1,+], Guangwu Sun[1], Xi Zou[1], Xiaoru Sang[1], Yongmin He[1] & Bing Gao[1,*]

[1] State Key Laboratory of Chemo/Bio-Sensing and Chemometrics, College of Chemistry and Chemical Engineering, Hunan University, Changsha, 410082, China.

* E-mail: gaobing@hnu.edu.cn

Table of contents

Section 1. General information....2

Section 2. Preparation of the starting materials....3

Section 3. The photochemical setup and optimization of reaction conditions....26

Section 4. The substrate scope....32

Section 5. The control experiments....63

Section 6. Competitive reaction and products derivatizations....86

Section 7. NMR spectra....93

Section 8. Supplementary references....249

Section 1. General information

^{1}H, ^{13}C and ^{19}F NMR spectra were recorded at room temperature on a Bruker AM-400 instrument. Proton magnetic resonance (^{1}H NMR) spectra were recorded at 400 MHz. Carbon magnetic resonance (^{13}C NMR) spectra were recorded at 100 MHz. Fluorine magnetic resonance (^{19}F NMR) spectra were recorded at 376 MHz. The ^{1}H, ^{13}C and ^{19}F NMR spectra were recorded at 297 K in $CDCl_3$, DMSO-d_6 and the chemical shifts (δ) were presented in parts per million (ppm). Spectroscopic data of ^{1}H NMR were reported as follows: chemical shift, multiplicity (s = singlet, d = doublet, t = triplet, q = quartet, quint = quintet, sext = sextet, sept = septet, m = multiplet, br = broad), coupling constant (*J*) in Hertz (Hz), and integration. NMR spectra were processed by MestreNova. Spectroscopic data of ^{13}C and ^{19}F NMR were reported in a similar pattern if applicable, with the exception that singlet peaks in the ^{13}C NMR spectra were reported only with their chemical shifts. For ^{1}H NMR, the chemical shifts were calibrated in reference to the residual undeuterated solvents ($CDCl_3$, 7.26 ppm; CD_2Cl_2, 5.32 ppm; DMSO- d_6, 2.50 ppm). Spectroscopic data of the ^{13}C NMR spectra were calibrated in reference to the deuterated solvents ($CDCl_3$, 77.16 ppm; CD_2Cl_2, 54.00 ppm, DMSO-d_6, 39.52 ppm). High resolution mass spectrometry (HRMS) was recorded on a MAT-95-XP instrument with (EI)ionization mode and Waters G2-XS qtof instrument with (ESI) electrospray ionization. Melting points (m.p.) were determined using a MP-430 auto melting point apparatus. GC-MS data were recorded on an Agilent 8860 GC system with an Agilent 5977B Inert MSD system operating in electron impact ionization (EI) mode. LC-MS data were recorded on the Shimadzu2020 instrument. X-ray crystallography was performed on a Bruker D8 Quest instrument. Thin layer chromatography (TLC) was performed using TLC silica gel plates HSG F254 (Yantai) and visualized using UV light, iodine, phosphomolybdic acid or potassium permanganate. Silica gel column chromatography was carried out using 300-400 mesh silica gel (Yantai). All purchased reagents and solvents were used as received, without further purification or special handling practice.

Section 2. Preparation of the starting materials

2.1 Preparation of sulfonyl fluorides

Sulfonyl fluorides **1u** was purchased and used as received. The others were synthesized according to the literature procedures.[1-3]

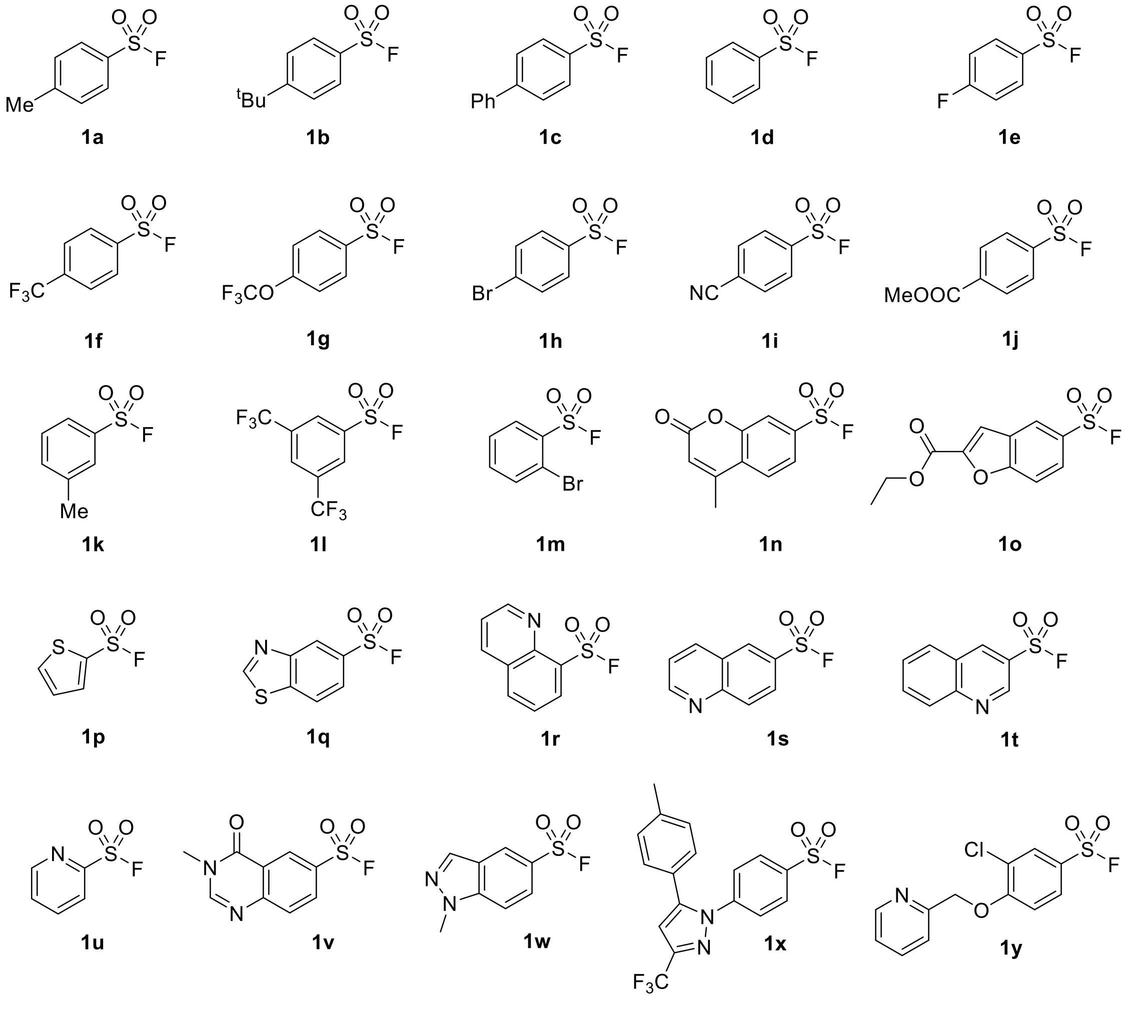

Supplementary Figure 1. The scope of sulfonyl fluorides

General Procedure：

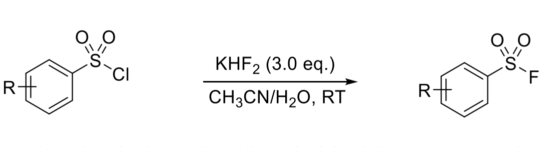

General Procedure A: To a stirred solution of sulfonyl chloride (10.0 mmol, 1.0 eq.) in acetonitrile (15.0 mL) was added KHF_2 (2.34 g, 3.0 eq.) and water (15.0 mL). The reaction mixture was stirred at room temperature for 12 h. Then the resulting mixture was transferred to a separatory funnel and the organic layer was collected. The aqueous phase was extracted with ethyl acetate for three times. The combined organic layer was washed with saturated brine, dried over anhydrous sodium sulfate, filtered, and concentrated in vacuo. The product was purified by flash column chromatography.

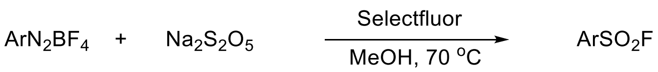

General Procedure B: Under the N_2 atmosphere, a 10 mL Schlenk tube was charged with aryldiazonium salts (1.0 eq), $Na_2S_2O_5$ (3.0 eq.), Selectfluor (2.0 eq.) and MeOH (0.2 M). After stirred at 70 ºC for 9 h, the reaction mixture was filtered through a pad of celite, eluted with ethyl acetate. The filtrate was concentrated and purified by flash column chromatography on silica gel to give the sulfonyl fluorides.

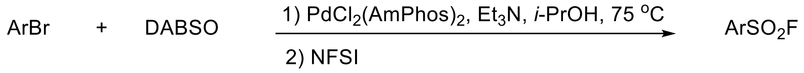

General Procedure C: Under the N_2 atmosphere, a 10 mL Schlenk tube was charged with DABSO (0.6 eq.), $PdCl_2(AmPhos)_2$ (0.05 eq.) and aryl bromides (1.0 eq.). Anhydrous isopropanol (0.25 M) and anhydrous triethylamine (3.0 eq.) were added sequentially and the reaction mixture stirred at 75 °C for 24 h. After cooling to r.t., NFSI (1.5 eq.) was added and the reaction mixture stirred for 3 h until completion. The reaction mixture was concentrated in vacuo, then dissolved in EtOAc and filtered through celite. The filtrate was washed with sat. aq. $Na_2S_2O_3$ and brine, dried ($MgSO_4$), and concentrated organic phase in vacuo to leave the crude product which was purified by column chromatography on silica gel to give the sulfonyl fluorides.

4-methylbenzenesulfonyl fluoride (**1a**)

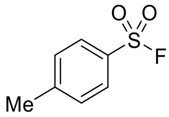

According to **General Procedure A** (30.0 mmol scale), **1a** was obtained as a white solid (5.19 g, 99% yield). R_f = 0.20 (PE), stained by UV light (254, 365 nm). **^{1}H NMR** (400 MHz, $CDCl_3$) δ 7.87 (d, *J* = 8.4 Hz, 2H), 7.41 (d, *J* = 8.1 Hz, 2H), 2.48 (s, 3H); **^{19}F NMR** (376 MHz, $CDCl_3$) δ 66.2 (s, 1F). Spectroscopic data of **1a** matched the literature values.[4]

4-(*tert*-butyl)benzenesulfonyl fluoride (**1b**)

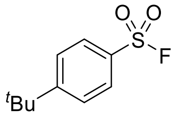

According to **General Procedure A** (10.0 mmol scale), **1b** was obtained as white solid (1.88 g, 87% yield). R_f = 0.20 (PE), stained by UV light (254, 365 nm). **^{1}H NMR** (400 MHz, $CDCl_3$) δ 7.96 – 7.91 (m, 2H), 7.66 – 7.61 (m, 2H), 1.37 (s, 9H); **^{19}F NMR** (376 MHz, $CDCl_3$) δ 66.1 (s, 1F). Spectroscopic data of **1b** matched the literature values.[2]

[1,1'-biphenyl]-4-sulfonyl fluoride (**1c**)

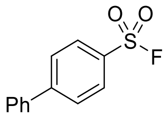

According to **General Procedure A** (10.0 mmol scale), **1c** was obtained as a white solid (2.09 g, 89% yield). R_f = 0.20 (PE), stained by UV light (254, 365 nm). **^{1}H NMR** (400 MHz, $CDCl_3$) δ 8.12 – 8.04 (m, 2H), 7.90 – 7.76 (m, 2H), 7.66 – 7.59 (m, 2H), 7.58 – 7.43 (m, 3H); **^{19}F NMR** (376 MHz, $CDCl_3$) δ 66.4 (s, 1F). Spectroscopic data of **1c** matched the literature values.[4]

Benzenesulfonyl fluoride (**1d**)

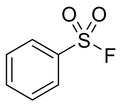

According to **General Procedure A** (50.0 mmol scale), **1d** was obtained as a colorless oil (7.17 g, 90% yield). R_f = 0.30 (PE), stained by UV light (254, 365 nm). **^{1}H NMR** (400 MHz, $CDCl_3$) δ 8.00 (d, 2H), 7.78 (t, J = 7.6 Hz, 1H), 7.63 (t, 2H); **^{19}F NMR** (376 MHz, $CDCl_3$) δ 65.8 (s, 1F). Spectroscopic data of **1d** matched the literature values.[4]

4-fluorobenzenesulfonyl fluoride (**1e**)

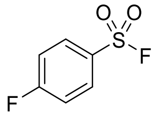

According to **General Procedure A** (10.0 mmol scale), **1e** was obtained as colorless oil (800.0 mg, 45% yield). R_f = 0.30 (PE), stained by UV light (254, 365 nm). **^{1}H NMR** (400 MHz, $CDCl_3$) δ 8.08 – 8.01 (m, 2H), 7.31 (t, J = 8.6 Hz, 2H); **^{19}F NMR** (376 MHz, $CDCl_3$) δ 66.7 (s, 1F), -99.4 (s, 1F). Spectroscopic data of **1e** matched the literature values.[5]

4-(trifluoromethyl)benzenesulfonyl fluoride (**1f**)

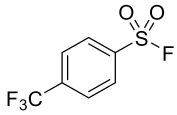

According to **General Procedure A** (20.0 mmol scale), **1f** was obtained as white solid (3.44 g, 76% yield). R_f = 0.30 (PE), stained by UV light (254, 365 nm). **^{1}H NMR** (400 MHz, $CDCl_3$) δ 8.17 (d, J = 8.3 Hz, 2H), 7.92 (d, J = 8.2 Hz, 2H); **^{19}F NMR** (376 MHz, $CDCl_3$) δ 65.9 (s, 1F), -63.5 (s, 3F). Spectroscopic data of **1f** matched the literature values.[4]

4-(trifluoromethoxy)benzenesulfonyl fluoride (**1g**)

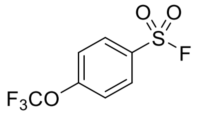

According to **General Procedure A** (10.0 mmol scale), **1j** was obtained as colorless oil (2.10g, 86% yield). R_f = 0.40 (PE), stained by UV light (254, 365 nm). **^{1}H NMR** (400 MHz, $CDCl_3$) δ 8.12 – 8.06 (m, 2H), 7.49 – 7.43 (m, 2H); **^{19}F NMR** (376 MHz, $CDCl_3$) δ 66.4 (s, 1F), -57.8 (s, 3F). Spectroscopic data of **1j** matched the literature values.[5]

4-bromobenzenesulfonyl fluoride (**1h**)

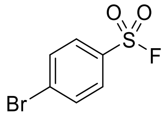

According to **General Procedure A** (10.0 mmol scale), **1h** was obtained as a colorless oil (2.01 g, 84% yield). R_f = 0.30 (PE), stained by UV light (254, 365 nm). **^{1}H NMR** (400 MHz, $CDCl_3$) δ 7.91 – 7.83 (m,

2H), 7.78 (d, *J* = 8.6 Hz, 2H); **^{19}F NMR** (376 MHz, $CDCl_3$) δ 66.4 (s, 1F). Spectroscopic data of **1h** matched the literature values.[4]

4-cyanobenzenesulfonyl fluoride (**1i**)

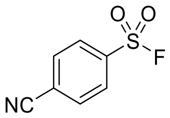

According to **General Procedure A** (16.0 mmol scale), **1i** was obtained as white solid (2.40 g, 84% yield). R_f = 0.20 (PE/EA = 40:1), stained by UV light (254, 365 nm). **^{1}H NMR** (400 MHz, $CDCl_3$) δ 8.15 (d, *J* = 8.6 Hz, 2H), 7.95 (d, *J* = 8.0 Hz, 2H); **^{19}F NMR** (376 MHz, $CDCl_3$) δ 65.9 (s, 1F). Spectroscopic data of **1i** matched the literature values.[2]

Methyl 4-(fluorosulfonyl)benzoate (**1j**)

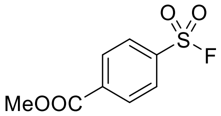

According to **General Procedure** A (2.50 mmol scale), **1g** was obtained as white solid (525.0 mg, 96% yield). R_f = 0.20 (PE/EA=40:1), stained by UV light (254, 365 nm). **^{1}H NMR** (400 MHz, $CDCl_3$) δ 8.31 – 8.26 (m, 2H), 8.18 – 8.04 (m, 2H), 4.00 (s, 3H); **^{19}F NMR** (376 MHz, $CDCl_3$) δ 65.7 (s, 1F). Spectroscopic data of **1g** matched the literature values.[3]

3-methylbenzenesulfonyl fluoride (**1k**)

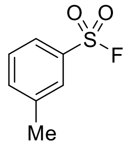

According to **General Procedure A** (2.50 mmol scale), **1k** was obtained as colorless oil (180.0 mg, 41% yield). R_f = 0.20 (PE), stained by UV light (254, 365 nm). **^{1}H NMR** (400 MHz, $CDCl_3$) δ 7.83 – 7.78 (m, 2H), 7.60 – 7.55 (m, 1H), 7.54 – 7.48 (m, 1H), 2.47 (s, 3H); **^{19}F NMR** (376 MHz, $CDCl_3$) δ 65.8 (s, 1F). Spectroscopic data of **1k** matched the literature values.[5]

3,5-bis(trifluoromethyl)benzenesulfonyl fluoride (**1l**)

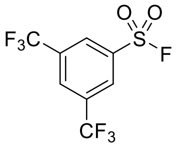

According to **General Procedure A** (3.2 mmol scale), **1l** was obtained as a light-yellow oil (578.0 mg, 62% yield). R_f = 0.40 (PE), stained by UV light (254, 365 nm). **^{1}H NMR** (400 MHz, $CDCl_3$) δ 8.47 (s, 2H), 8.29 (s, 1H); **^{19}F NMR** (376 MHz, $CDCl_3$) δ 66.5 (s, 1F), -63.3 (s, 6F). Spectroscopic data of **1l** matched the literature values.[6]

2-bromobenzenesulfonyl fluoride (**1m**)

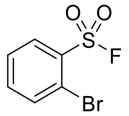

According to **General Procedure A** (2.50 mmol scale), **1m** was obtained as brown solid (240.0 mg, 40% yield). R_f = 0.30 (PE), stained by UV light (254, 365 nm). **^{1}H NMR** (400 MHz, $CDCl_3$) δ 8.13 (dd, J = 7.4, 2.3 Hz, 1H), 7.84 (dd, J = 7.4, 1.8 Hz, 1H), 7.62 – 7.52 (m, 2H); **^{19}F NMR** (376 MHz, $CDCl_3$) δ 58.0 (s, 1F). Spectroscopic data of **1m** matched the literature values.[2]

4-Methyl-2-oxo-2H-chromene-7-sulfonyl fluoride (**1n**)

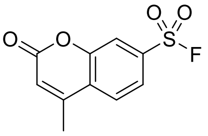

According to **General Procedure B** (4.2 mmol scale), **1n** was obtained as a white solid (492.0 mg, 49% yield). R_f = 0.30 (PE/EA = 5:1), stained by UV light (254, 365 nm). **^{1}H NMR** (400 MHz, $CDCl_3$) δ 7.87 (s, 3H), 6.48 (s, 1H), 2.51 (s, 3H); **^{19}F NMR** (376 MHz, $CDCl_3$) δ 66.3 (s, 1F). Spectroscopic data of **1n** matched the literature values.[2]

Ethyl 5-(fluorosulfonyl)benzofuran-2-carboxylate (**1o**)

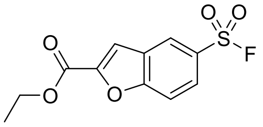

According to **General Procedure B** (5.0 mmol scale), **1o** was obtained as a white solid (596.0 mg, 44% yield). R_f = 0.40 (PE/EA = 10:1), stained by UV light (254, 365 nm). **^{1}H NMR** (400 MHz, $CDCl_3$) δ 8.42 (s, 1H), 8.05 (d, J = 8.7 Hz, 1H), 7.80 (d, J = 8.8 Hz, 1H), 7.63 (s, 1H), 4.46 (q, J = 14.0, 6.9 Hz, 2H), 1.43 (t, J = 7.0 Hz, 3H); **^{19}F NMR** (376 MHz, $CDCl_3$) δ 67.6 (s, 1F). Spectroscopic data of **1o** matched the literature values.[2]

Thiophene-2-sulfonyl fluoride (**1p**)

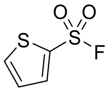

According to **General Procedure A** (5.0 mmol scale), **1p** was obtained as a light-yellow oil (467.0 mg, 56% yield). R_f = 0.20 (PE), stained by UV light (254, 365 nm). **^{1}H NMR** (400 MHz, $CDCl_3$) δ 7.92 (m, J = 3.9, 1.4 Hz, 1H), 7.88 (dd, J = 5.0, 1.4 Hz, 1H), 7.26 – 7.22 (m, 1H); **^{19}F NMR** (376 MHz, $CDCl_3$) δ 71.8 (s, 1F). Spectroscopic data of **1p** matched the literature values.[7]

Benzo[d]thiazole-5-sulfonyl fluoride (**1q**)

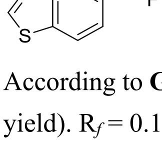

According to **General Procedure B** (2.7 mmol scale), **1q** was obtained as a white solid (315.6 mg, 37% yield). R_f = 0.15 (PE/EA = 5:1), stained by UV light (254, 365 nm). **^{1}H NMR** (400 MHz, $CDCl_3$) δ 9.23

(s, 1H), 8.80 (s, 1H), 8.23 (d, J = 8.5 Hz, 1H), 8.04 (d, J = 8.5 Hz, 1H); **^{19}F NMR** (376 MHz, $CDCl_3$) δ 67.4 (s, 1F). Spectroscopic data of **1q** matched the literature values.[2]

Quinoline-8-sulfonyl fluoride (**1r**)

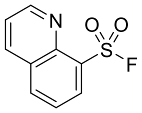

According to **General Procedure A** (10.0 mmol scale), **1r** was obtained as white solid (1.76 g, 83% yield). R_f = 0.20 (PE/EA = 5:1), stained by UV light (254, 365 nm). **^{1}H NMR** (400 MHz, $CDCl_3$) δ 9.18 (dd, J = 4.3, 1.7 Hz, 1H), 8.52 (dd, J = 7.5, 1.4 Hz, 1H), 8.31 (dd, J = 8.4, 1.8 Hz, 1H), 8.23 (dd, J = 8.3, 1.4 Hz, 1H), 7.71 (td, J = 8.6, 7.5, 1.4 Hz, 1H), 7.62 (dd, J = 8.4, 4.3 Hz, 1H); **^{19}F NMR** (376 MHz, $CDCl_3$) δ 60.2 (s, 1F). Spectroscopic data of **1r** matched the literature values.[8]

Quinoline-6-sulfonyl fluoride (**1s**)

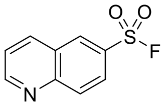

According to **General Procedure B** (2.7 mmol scale), **1s** was obtained as a white solid (108.0 mg, 19% yield). R_f = 0.12 (PE/EA = 5:1), stained by UV light (254, 365 nm). **^{1}H NMR** (400 MHz, $CDCl_3$) δ 9.14 (d, J = 3.4 Hz, 1H), 8.61 (s, 1H), 8.34 (t, J = 9.0 Hz, 2H), 8.17 (d, J = 8.9 Hz, 1H), 7.62 (dd, J = 8.3, 4.2 Hz, 1H); **^{19}F NMR** (376 MHz, $CDCl_3$) δ 66.4 (s, 1F). Spectroscopic data of **1s** matched the literature values.[2]

Quinoline-3-sulfonyl fluoride (**1t**)

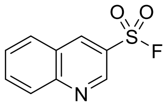

According to **General Procedure C** (4.7 mmol scale), **1t** was obtained as a white solid (496.0 mg, 50% yield). R_f = 0.50 (PE/EA = 5:1), stained by UV light (254, 365 nm). **^{1}H NMR** (400 MHz, $CDCl_3$) δ 9.34 (s, 1H), 8.89 (s, 1H), 8.27 (d, J = 8.4 Hz, 1H), 8.05 – 7.99 (m, 2H), 7.78 (t, J = 6.7 Hz, 1H); **^{19}F NMR** (376 MHz, $CDCl_3$) δ 68.8 (s, 1F). Spectroscopic data of **1t** matched the literature values.[3]

3-Methyl-4-oxo-3,4-dihydroquinazoline-6-sulfonyl fluoride (**1v**)

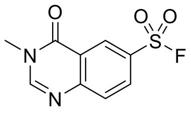

According to **General Procedure C** (2.0 mmol scale), **1v** was obtained as a light-yellow solid (125.6 mg, 26% yield). R_f = 0.20 (PE/EA = 1:1), stained by UV light (254, 365 nm). **^{1}H NMR** (400 MHz, $CDCl_3$) δ 8.98 (s, 1H), 8.25 (dd, J = 8.7, 1.8 Hz, 1H), 8.21 (s, 1H), 7.91 (d, J = 8.7 Hz, 1H), 3.62 (s, 3H); **^{19}F NMR** (376 MHz, $CDCl_3$) δ 66.6 (s, 1F). Spectroscopic data of **1v** matched the literature values.[3]

1-Methyl-1H-indazole-5-sulfonyl fluoride (**1w**)

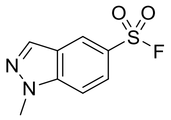

According to **General Procedure C** (5.0 mmol scale), **1w** was obtained as a yellow solid (284.0 mg, 66% yield). R_f = 0.4 (PE/EA = 5:1), stained by UV light (254, 365 nm). **^{1}H NMR** (400 MHz, $CDCl_3$) δ 8.46 (s, 1H), 8.17 (s, 1H), 7.89 (d, J = 8.8 Hz, 1H), 7.57 (d, J = 8.9 Hz, 1H), 4.14 (s, 3H); **^{19}F NMR** (376 MHz, $CDCl_3$) δ 68.0 (s, 1F). Spectroscopic data of **1w** matched the literature values.[3]

4-(5-(p-tolyl)-3-(trifluoromethyl)-1H-pyrazol-1-yl)benzenesulfonyl fluoride (**1x**)

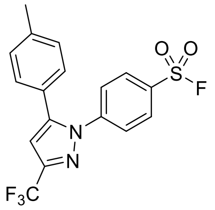

According to **General Procedure C** (2.0 mmol scale), **1x** was obtained as a white solid (426.0 mg, 55% yield). R_f = 0.35 (PE/EA = 40:1), stained by UV light (254, 365 nm). **^{1}H NMR** (400 MHz, $CDCl_3$) δ 7.99 (d, J = 8.7 Hz, 2H), 7.60 (d, J = 8.5 Hz, 2H), 7.22 (d, J = 7.9 Hz, 2H), 7.14 (d, J = 7.9 Hz, 2H), 6.77 (s, 1H), 2.41 (s, 3H); **^{19}F NMR** (376 MHz, $CDCl_3$) δ 66.4 (s, 1F), 62.6 (s, 3F). Spectroscopic data of **1x** matched the literature values.[3]

3-Chloro-4-(pyridin-2-ylmethoxy)benzenesulfonyl fluoride (**1y**)

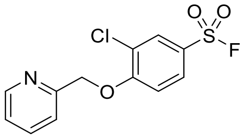

According to **General Procedure B** (5.0 mmol scale), **1y** was obtained as a white solid (834.0 mg, 55% yield). R_f = 0.20 (PE/EA = 5:1), stained by UV light (254, 365 nm). **^{1}H NMR** (400 MHz, $CDCl_3$) δ 8.61 (d, J = 4.6 Hz, 1H), 8.04 (d, J = 2.1 Hz, 1H), 7.87 (dd, J = 8.8, 2.1 Hz, 1H), 7.77 (t, J = 7.7 Hz, 1H), 7.58 (d, J = 7.8 Hz, 1H), 7.30 – 7.27 (m, 1H), 7.19 (d, J = 8.8 Hz, 1H), 5.39 (s, 2H); **^{19}F NMR** (376 MHz, $CDCl_3$) δ 67.5 (s, 1F). Spectroscopic data of **1y** matched the literature values.[2]

2.2 Preparation of alkenes

Alkenes **2d**, **2h**, **2j**, **2n**, **2u**, **2r**, **2s**, **2t** were synthesized according to the literature procedures.[9-12] The others were purchased and used as received.

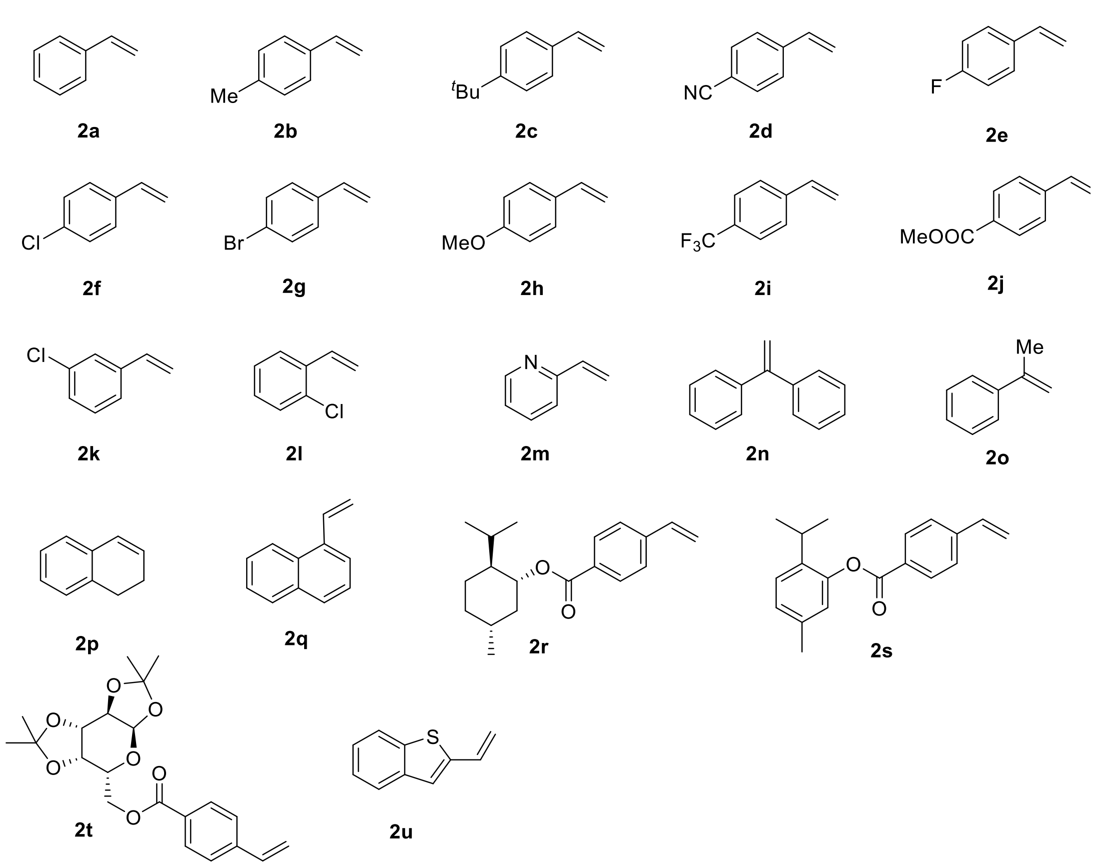

Supplementary Figure 2. The scope of alkenes

General Procedure：

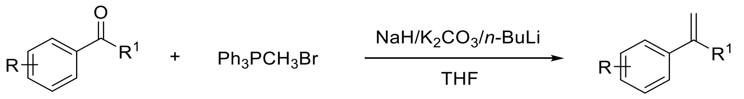

General Procedure D: An oven-dried 100-mL Schlenk flask equipped with a magnetic stir bar was charged with methyltriphenylphosphonium bromide (12.0 mmol, 1.20 eq.) and THF (15.0 mL), under nitrogen atmosphere. The solution was cooled to 0 ºC, then was added NaH (12.0 mmol, 1.20 eq.). The resulting reaction mixture was stirred for 30 mins at room temperature. Aromatic aldehyde (10.0 mmol, 1.0 eq.) in THF (5.0 mL) was the added dropwise at 0 ºC, and the reaction was stirred for another 12 h at room temperature. The reaction was quenched with water and the aqueous layer was extracted with DCM for three times. The combined organic layer was washed with saturated brine, dried over anhydrous sodium sulfate, and concentrated in vacuo. The product was purified by flash column chromatography.

General Procedure E: Aldehyde (10.0 mmol, 1.0 eq.) was added to a solution of methyltriphenylphosphonium bromide (12.0 mmol, 1.20 eq.) and K_2CO_3 (15.0 mmol, 1.50 eq.) in THF (15.0 mL). The mixture was refluxed at 75 ºC overnight. After being cooled to room temperature, solvent was removed by rotary evaporation. The residue was diluted with petroleum ether and filtered through celite, then concentrated under vacuum. The product was purified by flash column chromatography (petroleum ether).

General Procedure F: An oven-dried Schlenk flask was charged with the corresponding triphenylphosphonium bromide (2.0 mmol, 1.0 eq.) and THF (0.20 M) under nitrogen. The suspension was cooled to -20 °C and *n*-BuLi (2.50 M in hexane, 1.0 eq.) was added dropwise. The mixture was stirred for 2 h at -20 °C. Afterwards aldehyde (2.0 mmol, 1.0 eq.) was added dropwise as a solution in THF (1.25 M). The solution was warm to room temperature, then stirred for another 10 h. The reaction was quenched with NH_4Cl(aq.) and extracted with DCM for three times. The combined organic layer was washed with saturated brine, dried over anhydrous sodium sulfate, and concentrated in vacuo. The product was purified by flash column chromatography.

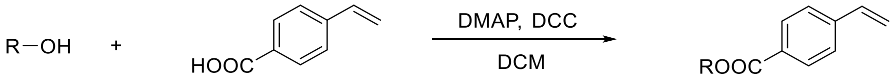

General Procedure G: To a 50 mL round-bottom flask was added alcohols (1.2 eq.), benzoic acid derivatives (1.0 eq.), DCC (1.2 eq.), DMAP (20 mol%) and DCM (0.1 M). The resulting solution was stirred at room temperature overnight, then quenched with H_2O, extracted with DCM. The combined organic layer was washed with brine, dried over Na_2SO_4, and concentrated. The residue was purified by flash chromatography on a silica gel to give the product.

4-vinylbenzonitrile (**2d**)

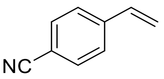

According to **General Procedure D** (10.0 mmol scale), **2d** was obtained as a colorless oil (1.02 g, 80% yield). R_f = 0.35 (PE/EA = 40: 1), stained by UV light (254, 365 nm). **^{1}H NMR** (400 MHz, $CDCl_3$) δ 7.59 (d, *J* = 8.3 Hz, 2H), 7.46 (d, *J* = 8.3 Hz, 2H), 6.71 (dd, *J* = 17.6, 10.9 Hz, 1H), 5.86 (d, *J* = 17.6 Hz, 1H), 5.43 (d, *J* = 10.9 Hz, 1H). Spectroscopic data of **2d** matched the literature values.[13]

1-methoxy-4-vinylbenzene (**2h**)

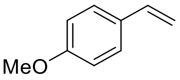

According to **General Procedure D** (10.0 mmol scale), **2h** was obtained as a colorless oil (967.0 mg, 72% yield). R_f = 0.60 (PE/EA = 40: 1), stained by UV light (254, 365 nm). **^{1}H NMR** (400 MHz, $CDCl_3$) δ 7.39 – 7.31 (m, 2H), 6.91 – 6.83 (m, 2H), 6.67 (dd, *J* = 17.6, 10.9 Hz, 1H), 5.62 (dd, *J* = 17.6, 1.0 Hz, 1H), 5.13 (dd, *J* = 10.9, 1.0 Hz, 1H), 3.82 (s, 3H). Spectroscopic data of **2h** matched the literature values.[13]

Methyl 4-vinylbenzoate (**2j**)

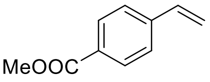

According to **General Procedure D** (20.0 mmol scale), **2j** was obtained as white solid (2.13 g, 66% yield). R_f = 0.50 (PE/EA = 20: 1), stained by UV light (254, 365 nm). ^{1}H NMR (400 MHz, $CDCl_3$) δ 8.03 – 7.96 (m, 2H), 7.51 – 7.41 (m, 2H), 6.75 (dd, *J* = 17.6, 10.9 Hz, 1H), 5.86 (dd, *J* = 17.6, 0.8 Hz, 1H), 5.38 (dd, *J* = 10.9, 0.8 Hz, 1H), 3.91 (s, 3H). Spectroscopic data of **2j** matched the literature values.[13]

Ethene-1,1-diyldibenzene (**2n**)

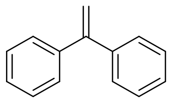

According to **General Procedure E** (10.0 mmol scale), **2n** was obtained as a colorless oil (1.60 g, 89% yield). R_f = 0.70 (PE), stained by UV light (254, 365 nm). **^{1}H NMR** (400 MHz, $CDCl_3$) δ 7.41 – 7.32 (m, 10H), 5.49 (s, 2H). Spectroscopic data of **2n** matched the literature values.[14]

(1R,2S,5R)-2-isopropyl-5-methylcyclohexyl 4-vinylbenzoate (**2r**)

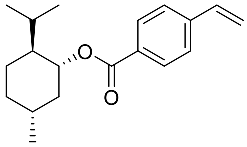

According to **General Procedure G** (3.0 mmol scale), **2r** was obtained as a colorless oil (554 mg, 65% yield). R_f = 0.53 (PE/EA = 50: 1), stained by UV light (254, 365 nm). **^{1}H NMR** (400 MHz, $CDCl_3$) δ δ 8.01 (d, J = 8.1 Hz, 2H), 7.46 (d, J = 8.1 Hz, 2H), 6.75 (dd, J = 17.6, 10.9 Hz, 1H), 5.85 (d, J = 17.6 Hz, 1H), 5.36 (d, J = 10.9 Hz, 1H), 4.94 (td, J = 10.8, 4.2 Hz, 1H), 2.13 (d, J = 11.8 Hz, 1H), 1.98 – 1.95 (m, 1H), 1.73 (d, J = 11.6 Hz, 2H), 1.58 – 1.53 (m, 2H), 1.18 – 1.06 (m, 2H), 0.93 (dd, J = 6.4, 4.1 Hz, 7H), 0.80 (d, J = 6.9 Hz, 3H). Spectroscopic data of **2r** matched the literature values.[12]

2-isopropyl-5-methylphenyl 4-vinylbenzoate (**2s**)

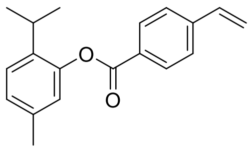

According to **General Procedure G** (3.0 mmol scale), **2s** was obtained as a colorless oil (227 mg, 27% yield). R_f = 0.51 (PE/EA = 50: 1), stained by UV light (254, 365 nm). **^{1}H NMR** (400 MHz, $CDCl_3$) δ 8.24 (d, J = 7.2 Hz, 2H), 7.58 (d, J = 7.3 Hz, 2H), 7.30 (d, J = 7.7 Hz, 1H), 7.11 (d, J = 7.8 Hz, 1H), 7.01 (s, 1H), 6.83 (dd, J = 17.4, 10.9 Hz, 1H), 5.95 (d, J = 17.6 Hz, 1H), 5.46 (d, J = 10.7 Hz, 1H), 3.12 (dt, J = 13.0, 6.5 Hz, 1H), 2.39 (s, 3H), 1.27 (d, J = 5.9 Hz, 6H); **^{13}C NMR** (100 MHz, $CDCl_3$) δ 165.2, 148.3, 142.7, 137.3, 136.7, 136.0, 130.6, 128.8, 127.2, 126.6, 126.4, 123.0, 117.0, 27.4, 23.1, 21.0; **HRMS** (EI, m/z): calcd. for $C_{19}H_{20}O_2$ $[M]^+$: 280.1458, found: 280.1460.

((3aR,5R,5aS,8aS,8bR)-2,2,7,7-tetramethyltetrahydro-5H-bis([1,3]dioxolo)[4,5-b:4',5'-d]pyran-5-yl)methyl 4-vinylbenzoate (**2t**)

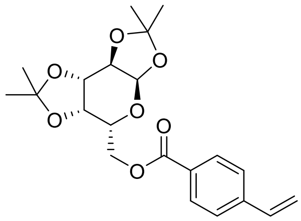

According to **General Procedure G** (3.0 mmol scale), **2t** was obtained as a white solid (772 mg, 66% yield). R_f = 0.25 (PE/EA = 10: 1), stained by UV light (254, 365 nm). **^{1}H NMR** (400 MHz, $CDCl_3$) δ 8.00 (d, J = 8.3 Hz, 2H), 7.45 (d, J = 8.3 Hz, 2H), 6.74 (dd, J = 17.6, 10.9 Hz, 1H), 5.85 (d, J = 17.6 Hz, 1H), 5.56 (d, J = 4.9 Hz, 1H), 5.37 (d, J = 10.9 Hz, 1H), 4.65 (dd, J = 7.9, 2.4 Hz, 1H), 4.52 (dd, J = 11.5,

4.8 Hz, 1H), 4.42 (dd, J = 11.5, 7.5 Hz, 1H), 4.35 – 4.31 (m, 2H), 4.19 – 4.16 (m, 1H), 1.51 (s, 3H), 1.47 (s, 3H), 1.35 (s, 3H), 1.33 (s, 3H). Spectroscopic data of **2t** matched the literature values.[15]

2-vinylbenzo[*b*]thiophene (**2u**)

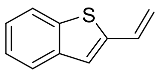

According to **General Procedure E** (10.0 mmol scale), **2u** was obtained as a colorless oil (1.60 g, 89% yield). R_f = 0.65 (PE), stained by UV light (254, 365 nm). ^{1}**H NMR** (400 MHz, $CDCl_3$) δ 7.80 – 7.74 (m, 1H), 7.72 – 7.67 (m, 1H), 7.35 – 7.27 (m, 2H), 7.17 (s, 1H), 6.93 (dd, J = 17.3, 10.8 Hz, 1H), 5.68 (d, J = 17.3 Hz, 1H), 5.31 (d, J = 10.7 Hz, 1H). Spectroscopic data of **2u** matched the literature values.[16]

2.3 Preparation of β-(hetero)arylethenesulfonyl fluorides and alkyl sulfonyl fluorides

All sulfonyl fluorides were synthesized according to the literature procedures.[17-21]

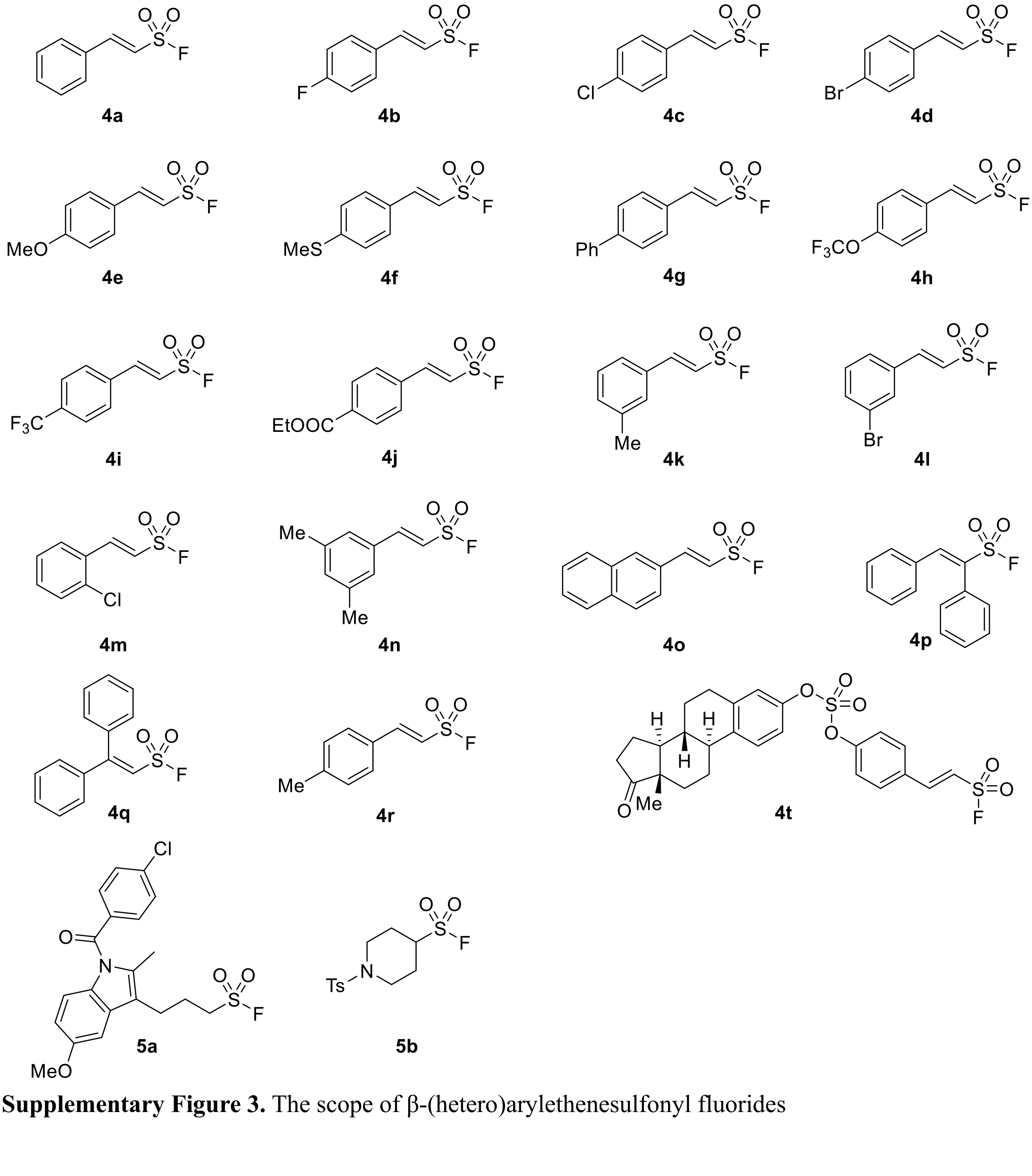

Supplementary Figure 3. The scope of β-(hetero)arylethenesulfonyl fluorides

2.3.1 General Procedure

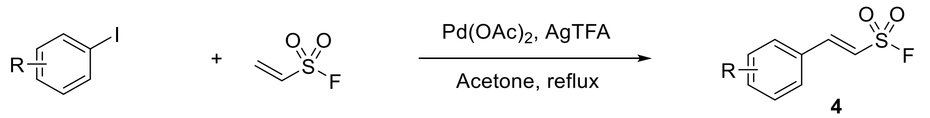

4

General Procedure H: An oven-dried Schlenk tube equipped with a magnetic stir bar was charged with AgTFA (2.4 mmol, 1.2 eq.), $Pd(OAc)_2$ (9.0 mg, 2 mol%), acetone (5.0 mL), (hetero)aryl iodide (2.0 mmol) and ethenesulfonyl fluoride (ESF, 440.0 mg, 4.0 mmol, 2.0 eq.). The mixture was refluxed at 60 °C until aryl iodide was consumed completely. Thereafter, the solvent was removed under the reduced pressure and the product was purified by flash column chromatography.

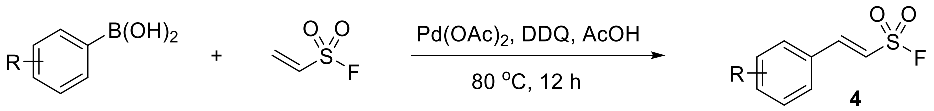

4

General Procedure I: An oven-dried Schlenk tube (25.0 mL) was charged with AcOH (5.0 mL), arylboronic acid (1.0 mmol, 1.0 eq.), 2,3-dicyano-5,6-dichlorobenzoquinone (DDQ, 1.5 mmol, 1.5 eq.), $Pd(OAc)_2$ (11.2 mg, 5 mol%), and ethenesulfonyl fluoride (ESF, 330.0 mg, 3.0 mmol, 3.0 eq.). The resulting mixture was allowed to react at 80 °C for 12 h. After the reaction was completed, the solvent was removed under the reduced pressure and the product was purified by flash column chromatography.

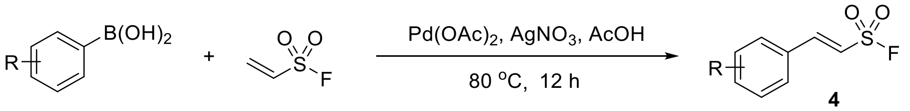

4

General procedure J: An oven-dried Schlenk tube (25.0 mL) was charged with AcOH (5.0 mL), arylboronic acid (3.0 mmol, 1.0 eq.), $AgNO_3$ (6.0 mmol, 2.0 eq.), $Pd(OAc)_2$ (5 mol%) and ethenesulfonyl fluoride (ESF, 9.0 mmol, 3.0 eq.). The mixture was allowed to react at 80 °C for 12 h. After the reaction was completed, the solvent was removed under reduced pressure and the product was purified by flash column chromatography.

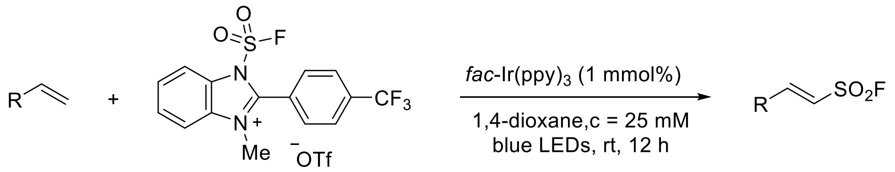

General procedure K: Under N_2 atmosphere, anhydrous 1,4-dioxane (4.0 mL, 0.025 M) and olefin substrate (0.1 mmol, 1.0 eq.) were added to an oven-dried Schlenk tube (10.0 mL) charged with *fac*-Ir(ppy)$_3$ (0.001 mmol, 1 mol%) and imidazolium salt (0.2 mmol, 2.0 eq.). The reaction mixture was allowed to stir at room temperature under irradiation with 5 W blue LEDs for 12 h. The product was purified by flash chromatography to give the corresponding pure product.

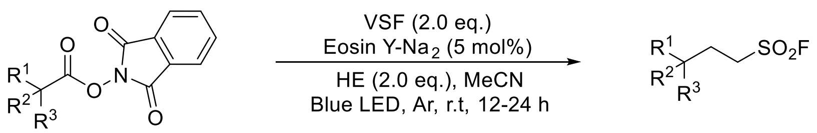

General procedure L: Under N_2 atmosphere, an oven-dried Schlenk tube (10.0 mL) was charged with NHPI redox-active ester (0.2 mmol, 1 eq.), Eosin Y-Na_2 (0.01 mmol, 0.05 eq.) and HE (0.4 mmol, 2 eq.), followed by the addition of dry MeCN (2 mL) and VSF (0.4 mmol, 2 eq.). The reaction mixture was stirred at room temperature for 12 to 24 h under the irradiation of 5 W blue LED band. The product was purified by flash chromatography to give the corresponding pure product.

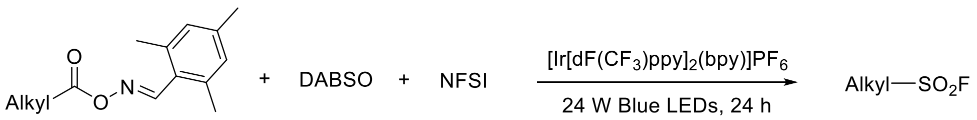

General procedure M: Under N_2 atmosphere, an oven-dried pressure tube (10.0 mL) was charged with oxime ester (0.1 mmol, 1.0 eq.), DABSO (0.15 mmol, 1.5 eq.), NFSI (0.175 mmol, 1.75 eq.), K_3PO_4 (0.1 mmol, 1.0 eq.), [Ir[dF(CF_3)ppy]$_2$(bpy)]PF_6 (1 mol%), and 3 mL solvent (MeCN:CH_2Cl_2=2:1). The reaction mixture was irradiated with 24 W Blue LEDs at room temperature. After 24 h, the reaction mixture was filtered through a pad of celite, eluted with ethyl acetate, concentrated, and purified by flash column chromatography on silica gel to give the desired product.

2.3.2 Other Procedures

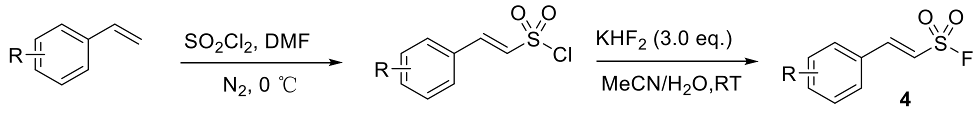

An oven-dried 25-mL Schlenk flask equipped with a magnetic stir bar was charged with sulfuryl chloride (10.0 mmol), under nitrogen atmosphere. Then, dry DMF (2.0 eq., 20.0 mmol) was added dropwise at 0 °C. The reaction mixture was warmed to room temperature and stirred for 30 mins. After that, olefin (10.0 mmol, 1.0 eq.) was added in three portion and the reaction mixture was gradually heated to 90 °C and stirred for another 3 h. After the reaction was completed, the resulting mixture was cooled and quenched with H_2O (100 mL), diluted with ether (100 mL), then washed with H_2O (2 ×100 mL) and brine (2 ×100 mL). The organic layer was dried with anhydrous sodium sulfate (Na_2SO_4) and concentrated in vacuo. Without further purification, the crude product was used for fluorination according to **General procedure A**. And compound **4** could be obtained.

(*E*)-2-phenylethene-1-sulfonyl fluoride (**4a**)

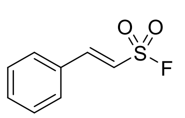

According to **General Procedure I** (1.0 mmol scale), **4a** was obtained as white solid (139 mg, 75% yield). R_f = 0.40 (PE/EA = 20:1), stained by UV light (254, 365 nm). **^{1}H NMR** (400 MHz, $CDCl_3$) δ 7.81 (d, J = 15.5 Hz, 1H), 7.59 – 7.45 (m, 5H), 6.92 – 6.83 (m, 1H); **^{19}F NMR** (376 MHz, $CDCl_3$) δ 62.3 (s, 1F). Spectroscopic data of **4a** matched the literature values.[17]

(*E*)-2-(4-fluorophenyl)ethene-1-sulfonyl fluoride (**4b**)

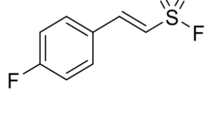

According to **General Procedure I** (1.0 mmol scale), **4b** was obtained as white solid (480.0 mg, 79% yield). R_f = 0.20 (PE/EA = 40:1), stained by UV light (254, 365 nm). **^{1}H NMR** (400 MHz, $CDCl_3$) δ 7.78 (d, J = 15.4 Hz, 1H), 7.64 – 7.52 (m, 2H), 7.18 (t, J = 8.3 Hz, 2H), 6.81 (d, J = 15.5 Hz, 1H); **^{19}F NMR** (376 MHz, $CDCl_3$) δ 62.4 (s, 1F), -105.0 (s, 1F). Spectroscopic data of **4b** matched the literature values.[17]

(*E*)-2-(4-chlorophenyl)ethene-1-sulfonyl fluoride (**4c**)

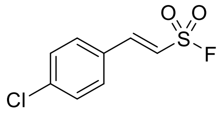

According to **General Procedure I** (3.0 mmol scale), **4c** was obtained as white solid (486.0 mg, 73% yield). R_f = 0.40 (PE/EA = 20:1), stained by UV light (254, 365 nm). **^{1}H NMR** (400 MHz, $CDCl_3$) δ 7.77 (d, J = 15.5 Hz, 1H), 7.53 – 7.42 (m, 4H), 6.85 (dd, J = 15.5, 2.5 Hz, 1H); **^{19}F NMR** (376 MHz, $CDCl_3$) δ 62.3 (s, 1F). Spectroscopic data of **4c** matched the literature values.[17]

(*E*)-2-(4-bromophenyl)ethene-1-sulfonyl fluoride (**4d**)

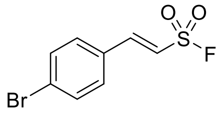

According to **General Procedure I** (3.0 mmol scale), **4d** was obtained as white solid (380.0 mg, 49% yield). R_f = 0.40 (PE/EA = 20:1), stained by UV light (254, 365 nm). **^{1}H NMR** (400 MHz, $CDCl_3$) δ 7.75 (d, J = 15.6 Hz, 1H), 7.62 (d, J = 8.6 Hz, 2H), 7.42 (d, J = 8.5 Hz, 2H), 6.87 (dd, J = 15.5, 2.5 Hz, 1H); **^{19}F NMR** (376 MHz, $CDCl_3$) δ 62.3 (s, 1F). Spectroscopic data of **4d** matched the literature values.[17]

(*E*)-2-(4-methoxyphenyl)ethene-1-sulfonyl fluoride (**4e**)

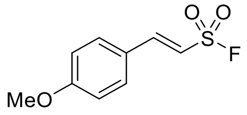

According to **General Procedure H** (2.0 mmol scale), **4e** was obtained as white solid (480.0 mg, 88% yield). R_f = 0.10 (PE/EA = 40:1), stained by UV light (254, 365 nm). **^{1}H NMR** (400 MHz, $CDCl_3$) δ 7.75 (d, J = 15.4 Hz, 1H), 7.54 – 7.47 (m, 2H), 7.03 – 6.90 (m, 2H), 6.70 (dd, J = 15.4, 2.6 Hz, 1H), 4.32 – 3.58 (s, 3H); **^{19}F NMR** (376 MHz, $CDCl_3$) δ 63.0 (s, 1F). Spectroscopic data of **4e** matched the literature values.[17]

(*E*)-2-(4-(methylthio)phenyl)ethene-1-sulfonyl fluoride (**4f**)

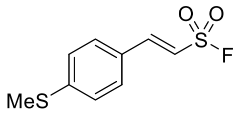

According to **General Procedure I** (3.0 mmol scale), **4f** was obtained as light-yellow solid (448.0 mg, 65% yield). R_f = 0.30 (PE/EA = 20:1), stained by UV light (254, 365 nm). **^{1}H NMR** (400 MHz, $CDCl_3$) δ 7.74 (d, J = 15.4 Hz, 1H), 7.48 – 7.40 (m, 2H), 7.31 – 7.23 (m, 2H), 6.79 (dd, J = 15.4, 2.5 Hz, 1H), 2.52 (s, 3H); **^{19}F NMR** (376 MHz, $CDCl_3$) δ 62.9 (s, 1F). Spectroscopic data of **4f** matched the literature values.[18]

(*E*)-2-([1,1'-biphenyl]-4-yl)ethene-1-sulfonyl fluoride (**4g**)

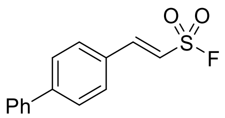

According to **General Procedure I** (3.0 mmol scale), **4g** was obtained as white solid (321.0 mg, 41% yield). R_f = 0.40 (PE/EA = 20:1), stained by UV light (254, 365 nm). **^{1}H NMR** (400 MHz, $CDCl_3$) δ 7.85 (d, *J* = 15.5 Hz, 1H), 7.71 (d, *J* = 8.5 Hz, 2H), 7.66 – 7.59 (m, 4H), 7.51 – 7.46 (m, 2H), 7.45 – 7.39 (m, 1H), 6.89 (dd, *J* = 15.5, 2.5 Hz, 1H); **^{19}F NMR** (376 MHz, $CDCl_3$) δ 62.6 (s, 1F). Spectroscopic data of **4g** matched the literature values.[17]

(*E*)-2-(4-(trifluoromethoxy)phenyl)ethene-1-sulfonyl fluoride (**4h**)

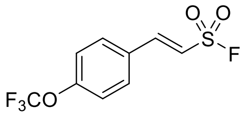

According to **General Procedure I** (3.0 mmol scale), **4h** was obtained as white solid (390.0 mg, 48% yield). R_f = 0.50 (PE/EA = 20:1), stained by UV light (254, 365 nm). **^{1}H NMR** (400 MHz, $CDCl_3$) δ 7.80 (d, *J* = 15.5 Hz, 1H), 7.61 (d, *J* = 8.4 Hz, 2H), 7.32 (d, *J* = 8.3 Hz, 2H), 6.86 (dd, *J* = 15.5, 2.5 Hz, 1H); **^{19}F NMR** (376 MHz, $CDCl_3$) δ 62.2 (s, 1F), -57.7 (s, 3F). Spectroscopic data of **4h** matched the literature values.[17]

(*E*)-2-(4-(trifluoromethyl)phenyl)ethene-1-sulfonyl fluoride (**4i**)

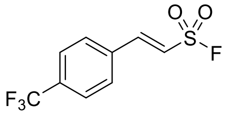

According to **General Procedure J** (3.0 mmol scale), **4i** was obtained as light green solid (525.0 mg, 69% yield). R_f = 0.30 (PE/EA = 20:1), stained by UV light (254, 365 nm). **^{1}H NMR** (400 MHz, $CDCl_3$) δ 7.84 (d, *J* = 15.6 Hz, 1H), 7.75 (d, *J* = 8.2 Hz, 2H), 7.68 (d, *J* = 8.2 Hz, 2H), 6.97 (dd, *J* = 15.6, 2.5 Hz, 1H); **^{19}F NMR** (376 MHz, $CDCl_3$) δ 62.0 (s, 1F), -63.2 (s, 3F). Spectroscopic data of **4i** matched the literature values.[17]

Ethyl (*E*)-4-(2-(fluorosulfonyl)vinyl)benzoate (**4j**)

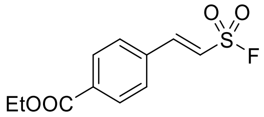

According to **General Procedure H** (2.0 mmol scale), **4j** was obtained as white solid (421.0 mg, 82% yield). R_f = 0.30 (PE/EA = 20:1), stained by UV light (254, 365 nm). **^{1}H NMR** (400 MHz, $CDCl_3$) δ 8.13 (d, *J* = 8.4 Hz, 2H), 7.83 (d, *J* = 15.6 Hz, 1H), 7.62 (d, *J* = 8.4 Hz, 2H), 6.97 (dd, *J* = 15.5, 2.5 Hz, 1H), 4.41 (q, *J* = 7.1 Hz, 2H), 1.41 (t, *J* = 7.1 Hz, 3H); **^{19}F NMR** (376 MHz, $CDCl_3$) δ 62.1 (s, 1F). Spectroscopic data of **4j** matched the literature values.[17]

(*E*)-2-(*m*-tolyl)ethene-1-sulfonyl fluoride (**4k**)

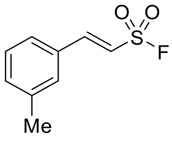

According to **General Procedure H** (2.0 mmol scale), **4k** was obtained as a yellow oil (375.0 mg, 94% yield). R_f = 0.40 (PE/EA = 20:1), stained by UV light (254, 365 nm). **^{1}H NMR** (400 MHz, $CDCl_3$) δ 7.78 (dd, *J* = 15.5, 1.2 Hz, 1H), 7.40 – 7.31 (m, 4H), 6.86 (dd, *J* = 15.5, 2.5 Hz, 1H), 2.41 (s, 3H); **^{19}F NMR** (376 MHz, $CDCl_3$) δ 62.4 (s, 1F), -105.0 (m, 1F). Spectroscopic data of **4k** matched the literature values.[17]

(*E*)-2-(3-bromophenyl)ethene-1-sulfonyl fluoride (**4l**)

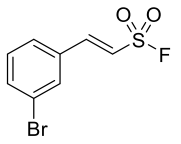

According to **General Procedure I** (3.0 mmol scale), **4l** was obtained as white solid (476.0 mg, 60% yield). R_f = 0.40 (PE/EA = 20:1), stained by UV light (254, 365 nm). **^{1}H NMR** (400 MHz, $CDCl_3$) δ 7.74 (d, *J* = 15.6 Hz, 1H), 7.71 – 7.70 (m, 1H), 7.65 (d, *J* = 8.0 Hz, 1H), 7.48 (d, *J* = 7.8 Hz, 1H), 7.36 (t, *J* = 7.9 Hz, 1H), 6.88 (dd, *J* = 15.5, 2.5 Hz, 1H); **^{19}F NMR** (376 MHz, $CDCl_3$) δ 62.2 (s, 1F). Spectroscopic data of **4l** matched the literature values.[17]

(*E*)-2-(2-chlorophenyl)ethene-1-sulfonyl fluoride (**4m**)

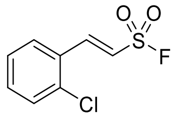

According to **General Procedure I** (3.0 mmol scale), **4m** was obtained as a colorless oil (225.0 mg, 34% yield). R_f = 0.30 (PE/EA = 20:1), stained by UV light (254, 365 nm). **^{1}H NMR** (400 MHz, $CDCl_3$) δ 8.23 (d, *J* = 15.5 Hz, 1H), 7.61 (d, *J* = 7.6 Hz, 1H), 7.54 – 7.42 (m, 2H), 7.41 – 7.34 (m, 1H), 6.94 (dd, *J* = 15.5, 2.5 Hz, 1H); **^{19}F NMR** (376 MHz, $CDCl_3$) δ 62.0 (s, 1F). Spectroscopic data of **4m** matched the literature values.[18]

(*E*)-2-(3,5-dimethylphenyl)ethene-1-sulfonyl fluoride (**4n**)

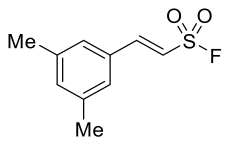

According to **General Procedure I** (3.0 mmol scale), **4n** was obtained as white solid (492.0 mg, 77% yield). R_f = 0.50 (PE/EA = 20:1), stained by UV light (254, 365 nm). **^{1}H NMR** (400 MHz, $CDCl_3$) δ 7.75 (d, *J* = 15.5, 1.2 Hz, 1H), 7.16 (s, 3H), 6.83 (dd, *J* = 15.5, 2.5 Hz, 1H), 2.36 (s, 6H); **^{19}F NMR** (376 MHz, $CDCl_3$) δ 62.4 (s, 1F). Spectroscopic data of **4n** matched the literature values.[17]

(*E*)-2-(naphthalen-2-yl)ethene-1-sulfonyl fluoride (**4o**)

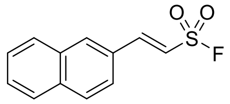

According to **General Procedure I** (3.0 mmol scale), **4o** was obtained as white solid (306.0 mg, 43% yield). R_f = 0.40 (PE/EA = 20:1), stained by UV light (254, 365 nm). **^{1}H NMR** (400 MHz, $CDCl_3$) δ 8.00 (s, 1H), 7.96 (d, *J* = 15.5, 1.1 Hz, 1H), 7.92 – 7.86 (m, 3H), 7.64 – 7.55 (m, 3H), 6.96 (dd, *J* = 15.4, 2.5

Hz, 1H); ^{19}**F NMR** (376 MHz, $CDCl_3$) δ 62.7 (s, 1F). Spectroscopic data of **4o** matched the literature values.[17]

(E)-1,2-diphenylethene-1-sulfonyl fluoride (**4p**)

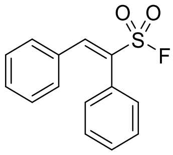

According to **General Procedure K** (0.2 mmol scale), **4p** was obtained as white solid (22.0 mg, 42% yield). R_f = 0.30 (PE/EA = 100:1), stained by UV light (254, 365 nm). ^{1}**H NMR** (400 MHz, $CDCl_3$) δ 7.94 (s, 1H), 7.53 – 7.47 (m, 3H), 7.44 (d, *J* = 6.6 Hz, 2H), 7.34 (t, *J* = 7.6 Hz, 1H), 7.23 (t, *J* = 7.8 Hz, 2H), 7.11 (d, *J* = 7.6 Hz, 2H); ^{19}**F NMR** (376 MHz, $CDCl_3$) δ 53.1 (s, 1F). Spectroscopic data of **4p** matched the literature values.[19]

2,2-Diphenylethene-1-sulfonyl fluoride (**4q**)

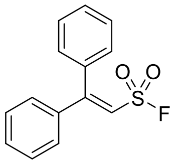

According to **General Procedure K** (0.2 mmol scale), **4q** was obtained as white solid (42.5 mg, 81% yield). R_f = 0.40 (PE/EA = 100:1), stained by UV light (254, 365 nm). ^{1}**H NMR** (400 MHz, $CDCl_3$) δ 7.53 – 7.45 (m, 4H), 7.40 (t, *J* = 7.6 Hz, 2H), 7.32 (t, *J* = 7.2 Hz, 4H), 6.84 (s, 1H); ^{19}**F NMR** (376 MHz, $CDCl_3$) δ 68.1 (s, 1F). Spectroscopic data of **4q** matched the literature values.[19]

(*E*)-2-(*p*-tolyl)ethene-1-sulfonyl fluoride (**4r**)

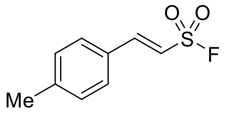

According to **General Procedure I** (5.0 mmol scale), **4r** was obtained as white solid (715.0 mg, 72% yield). R_f = 0.40 (PE/EA = 20:1), stained by UV light (254, 365 nm). ^{1}**H NMR** (400 MHz, $CDCl_3$) δ 7.69 (d, *J* = 15.4 Hz, 1H), 7.36 (d, *J* = 7.9 Hz, 2H), 7.19 (d, *J* = 8.0 Hz, 2H), 6.72 (dd, *J* = 15.5, 2.5 Hz, 1H), 2.34 (s, 3H); ^{19}**F NMR** (376 MHz, $CDCl_3$) δ 62.6 (s, 1F). Spectroscopic data of **4r** matched the literature values.[22]

4-((*E*)-2-(fluorosulfonyl)vinyl)phenyl ((8*R*,9*S*,13*S*,14*S*)-13-methyl-17-oxo-7,8,9,11,12,13,14,15,16,17-decahydro-6*H*-cyclopenta[*a*]phenanthren-3-yl) sulfate (**4t**)

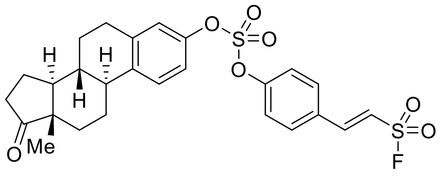

According to **General Procedure H** (1.2 mmol scale), **4t** was obtained as white solid (501.0 mg, 78% yield). R_f = 0.20 (PE/DCM = 1:1), stained by UV light (254, 365 nm). ^{1}**H NMR** (400 MHz, $CDCl_3$) δ 7.80 (d, *J* = 15.5 Hz, 1H), 7.64 (d, *J* = 8.8 Hz, 2H), 7.45 (d, *J* = 8.7 Hz, 2H), 7.33 (d, *J* = 8.5 Hz, 1H),

7.10 – 7.01 (m, 2H), 6.89 (dd, J = 15.5, 2.5 Hz, 1H), 2.93 (dd, J = 9.0, 4.3 Hz, 2H), 2.57 – 2.46 (m, 1H), 2.43 – 2.37 (m, 1H), 2.35 – 2.25 (m, 1H), 2.21 – 2.12 (m, 1H), 2.12 – 1.94 (m, 3H), 1.72 – 1.39 (m, 6H), 0.92 (s, 3H); **^{19}F NMR** (376 MHz, $CDCl_3$) δ 62.2 (s, 1F). Spectroscopic data of **4t** matched the literature values.[17]

3-(1-(4-chlorobenzoyl)-5-methoxy-2-methyl-1H-indol-3-yl)propane-1-sulfonyl fluoride (**5a**)

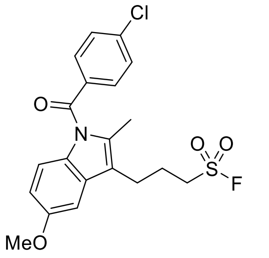

According to **General Procedure L** (2.75 mmol scale), **5a** was obtained as white solid (800.0 mg, 55% yield). R_f = 0.15 (PE/EA = 10:1), stained by UV light (254, 365 nm). **^{1}H NMR** (400 MHz, $CDCl_3$) δ 7.66 (d, J = 8.4 Hz, 2H), 7.48 (d, J = 8.4 Hz, 2H), 6.90 (d, J = 2.3 Hz, 1H), 6.84 (d, J = 9.0 Hz, 1H), 6.68 (dd, J = 9.0, 2.4 Hz, 1H), 3.84 (s, 3H), 3.41 (td, J = 7.5, 4.3 Hz, 2H), 2.90 (t, J = 7.3 Hz, 2H), 2.37 (s, 3H), 2.33 – 2.25 (m, 2H); **^{19}F NMR** (376 MHz, $CDCl_3$) δ 54.3 (s, 1F). Spectroscopic data of **5a** matched the literature values.[20]

1-Tosylpiperidine-4-sulfonyl fluoride (**5b**)

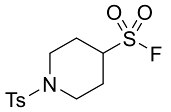

According to **General Procedure M** (0.2 mmol scale), **5b** was obtained as white solid (17.7 mg, 55% yield). R_f = 0.18 (PE/EA = 5:1), stained by UV light (254, 365 nm). **^{1}H NMR** (400 MHz, $CDCl_3$) δ 7.64 (d, J = 8.2 Hz, 2H), 7.34 (d, J = 8.1 Hz, 2H), 3.91 (d, J = 12.3 Hz, 2H), 3.25 (ddd, J = 11.5, 7.8, 2.2 Hz, 1H), 2.47 – 2.40 (m, 5H), 2.30 – 2.27 (m, 2H), 2.07 (ddd, J = 24.9, 11.9, 4.2 Hz, 2H); **^{19}F NMR** (376 MHz, $CDCl_3$) δ 42.9 (s, 1F). Spectroscopic data of **5b** matched the literature values.[21]

2.4 Preparation of sulfonimidoyl fluoride

The substrates were synthesized according to the literature procedure.[23-26]

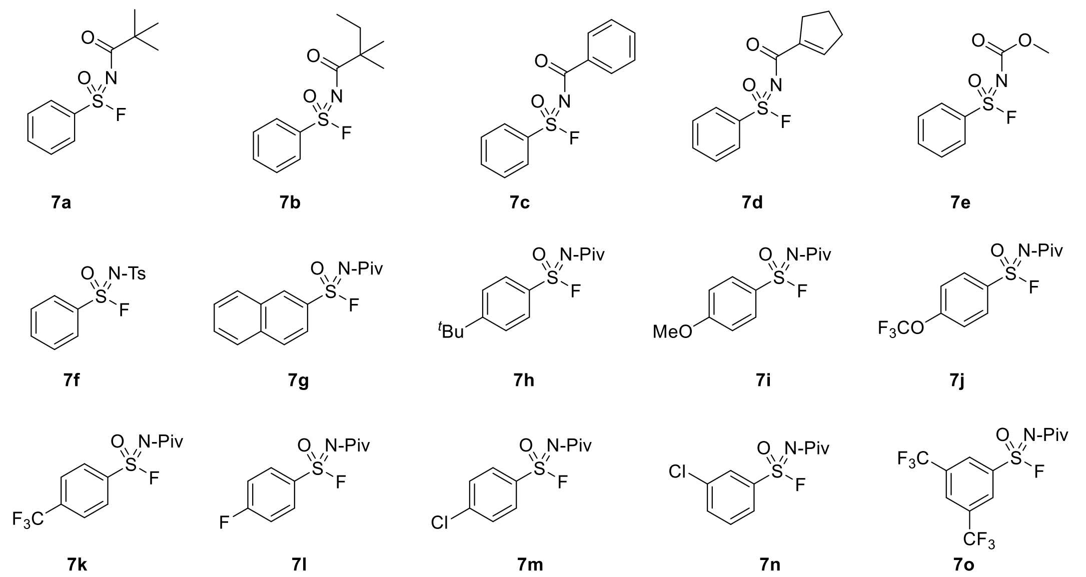

Supplementary Figure 4. The scope of sulfonimidoyl fluoride

General Procedure：

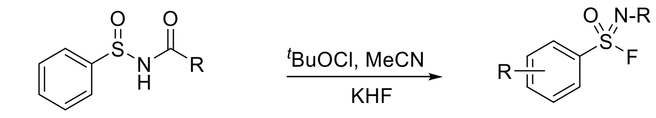

General Procedure N: To a 50 mL round bottom flask, sulfonamide synthesized according to literature[27-28] (2.5 mmol, 1 eq.) was added with acetonitrile (12 mL). Then, tert-butyl hypochlorite (2.88 mmol, 1.15 eq.) was added slowly to the round bottom flask at 0 ºC. The reaction was allowed to warm to room temperature and reacted for one hour. We added the aqueous solution of potassium hydrofluoride (2.0 M, 10 eq.) to the reaction solution. The reaction was then stirred for overnight. Next, we extracted three times with ethyl acetate, combined the organic layer, washed it with sodium chloride solution, and dried it with anhydrous sodium sulfate. The sulfonimidoyl fluoride products were purified by silica gel column chromatography using hexane/ethyl acetate as eluent.

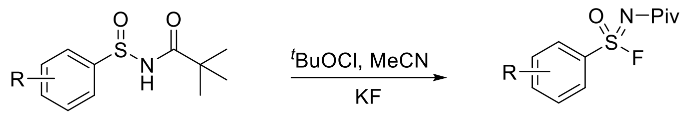

General Procedure O: To a 250 mL round bottom flask, sulfinamide (20 mmol, 1 eq.) was added with acetonitrile (0.2 M). The flask was then set in an ice bath and allowed to cool for 5 mins. Tert-butyl hypochlorite (1.5 eq.) was dissolved in acetonitrile (3.0 M) and added to the reaction flask slowly over the course of 5 mins. The reaction was allowed to warm to room temperature at which time potassium fluoride (4.0 eq.) was added to the reaction. The reaction was then stirred at room temperature until it reacts completely. At this time, the reaction was filtered through a pad of celite and the solvent was removed under reduced pressure. The sulfonimidoyl fluoride products were purified by silica gel column chromatography using hexane/ethyl acetate as eluent.

N-pivaloylbenzenesulfonimidoyl fluoride (**7a**)

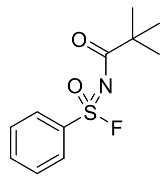

According to **General Procedure N** (21.7 mmol scale), **7a** was obtained as colorless oil (2.81 g, 54% yield). R_f = 0.42 (Petroleum ether /EtOAc = 10/1), stained by UV light (254, 365 nm). **^{1}H NMR** (400 MHz, $CDCl_3$) δ 8.07 (d, *J* = 7.9 Hz, 2H), 7.76 (t, *J* = 7.5 Hz, 1H), 7.62 (t, *J* = 7.8 Hz, 2H), 1.23 (s, 9H); **^{19}F NMR** (376 MHz, $CDCl_3$) δ 66.0 (s, 1F); Spectroscopic data of **7a** matched the literature values[24].

N-(2,2-dimethylbutanoyl)benzenesulfonimidoyl fluoride (**7b**)

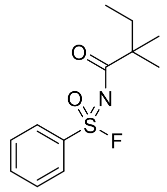

According to **General Procedure N** (16.7 mmol scale), **7b** was obtained as colorless oil (3.37 g, 82% yield). R_f = 0.52 (Petroleum ether /EtOAc = 10/1), stained by UV light (254, 365 nm).**^{1}H NMR** (400 MHz, $CDCl_3$) δ 8.04 (d, *J* = 8.1 Hz, 2H), 7.74 (t, *J* = 7.5 Hz, 1H), 7.61 (t, *J* = 7.8 Hz, 2H), 1.60 (q, *J* = 7.5 Hz, 2H), 1.16 (d, *J* = 3.3 Hz, 6H), 0.85 (t, *J* = 7.5 Hz, 3H); **^{13}C NMR** (100 MHz, $CDCl_3$) δ 183.2, 135.5, 134.6 (d, *J* = 21.0 Hz), 129.6, 127.8, 46.0, 33.3, 24.5, 24.5, 9.1; **^{19}F NMR** (376 MHz, $CDCl_3$) δ 66.2 (s, 1F); **HRMS** (ESI, m/z): calcd. for $C_{12}H_{16}FNNaO_2S$ $[M+Na]^+$: 280.0778, found: 280.0784.

N-benzoylbenzenesulfonimidoyl fluoride (**7c**)

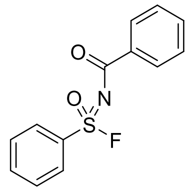

According to the literature[27] procedure (2.0 mmol scale), **7c** was obtained as colorless oil (430.0 mg, 82% yield). R_f = 0.41 (Petroleum ether /EtOAc = 10/1), stained by UV light (254, 365 nm). **^{1}H NMR** (400 MHz, $CDCl_3$) δ 8.19 – 8.14 (m, 4H), 7.80 (t, *J* = 7.5 Hz, 1H), 7.66 (t, *J* = 7.8 Hz, 2H), 7.57 (t, *J* = 7.3 Hz, 1H), 7.44 (t, *J* = 7.6 Hz, 2H); **^{19}F NMR** (376 MHz, $CDCl_3$) δ 65.5 (s, 1F); Spectroscopic data of **7c** matched the literature values[25].

N-(cyclopent-1-ene-1-carbonyl)benzenesulfonimidoyl fluoride (**7d**)

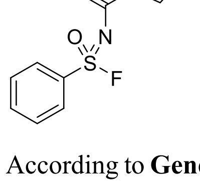

According to **General Procedure N** (9.0 mmol scale), **7d** was obtained as white solid (1.80 g, 80% yield). R_f = 0.40 (Petroleum ether /EtOAc = 10/1), stained by UV light (254, 365 nm). **^{1}H NMR** (400 MHz, $CDCl_3$) δ 8.11 (d, *J* = 7.8 Hz, 2H), 7.76 (t, *J* = 7.5 Hz, 1H), 7.63 (t, *J* = 7.8 Hz, 2H), 6.91 (s, 1H), 2.62 –

2.58 (m, 2H), 2.55 – 2.51 (m, 2H), 1.97 (p, J = 7.6 Hz, 2H); **^{13}C NMR** (100 MHz, $CDCl_3$) δ 168.3, 146.1, 141.9, 135.4, 134.9 (d, J = 21.0 Hz), 129.7, 128.0, 33.7, 31.1, 23.4; **^{19}F NMR** (376 MHz, $CDCl_3$) δ 66.8 (s, 1F); **HRMS** (ESI, m/z): calcd. for $C_{12}H_{12}FNNaO_2S$ $[M+Na]^+$: 276.0465, found: 276.0467

Methyl (fluoro(oxo)(phenyl)-l6-sulfanylidene)carbamate (**7e**)

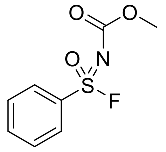

According to **General Procedure N** (1.5 mmol scale), **7e** was obtained as colorless oil (245.0 mg, 75% yield). R_f = 0.30 (Petroleum ether /EtOAc = 10/1), stained by UV light (254, 365 nm). **^{1}H NMR** (400 MHz, $CDCl_3$) δ 8.09 (d, J = 8.0 Hz, 2H), 7.77 (t, J = 7.5 Hz, 1H), 7.61 (t, J = 7.8 Hz, 2H), 3.80 (s, 3H); **^{13}C NMR** (100 MHz, $CDCl_3$) δ 154.4, 135.9, 133.4 (d, J = 21.0 Hz), 129.7, 128.2, 54.3; **^{19}F NMR** (376 MHz, $CDCl_3$) δ 68.9 (s, 1F); **HRMS** (ESI, m/z): calcd. for $C_8H_8FNNaO_3S$ $[M+Na]^+$: 240.0101, found: 240.0105.

N-tosylbenzenesulfonimidoyl fluoride (**7f**)

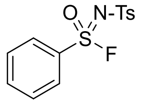

According to the literature[28] procedure (2.6 mmol scale), **7f** was obtained as white solid (710.0 mg, 88% yield). R_f = 0.30 (Petroleum ether /EtOAc = 5/1), stained by UV light (254, 365 nm). **^{1}H NMR** (400 MHz, $CDCl_3$) δ 8.05 – 8.03 (m, 2H), 7.94 (d, J = 8.3 Hz, 2H), 7.79 (t, J = 7.5 Hz, 1H), 7.62 (t, J = 7.9 Hz, 2H), 7.33 (d, J = 8.1 Hz, 2H), 2.44 (s, 3H); **^{19}F NMR** (376 MHz, $CDCl_3$) δ 73.7 (s, 1F); Spectroscopic data of **7f** matched the literature values[26].

N-pivaloylnaphthalene-2-sulfonimidoyl fluoride (**7g**)

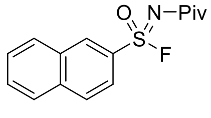

According to **General Procedure O** (2.0 mmol scale), **7a** was obtained as white solid (447.0 mg, 76% yield). R_f = 0.44 (Petroleum ether /EtOAc = 20/1), stained by UV light (254, 365 nm). **^{1}H NMR** (400 MHz, $CDCl_3$) δ 8.66 (s, 1H), 8.06 – 7.96 (m, 4H), 7.71 (dt, J = 14.6, 7.1 Hz, 2H), 1.28 (s, 9H); **^{13}C NMR** (100 MHz, $CDCl_3$) δ 183.9, 136.0, 131.9, 131.5 (d, J = 21.0 Hz), 130.5, 130.4, 130.1, 129.8, 128.4, 128.2, 121.9, 42.5, 27.3; **^{19}F NMR** (376 MHz, $CDCl_3$) δ 66.3 (s, 1F); **HRMS** (ESI, m/z): calcd. for $C_{15}H_{16}FNNaO_2S$ $[M+Na]^+$: 316.0778, found: 316.0769.

4-(tert-butyl)-N-pivaloylbenzenesulfonimidoyl fluoride (**7h**)

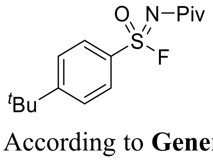

According to **General Procedure O** (3.0 mmol scale), **7b** was obtained as white solid (563.0 mg, 63% yield). R_f = 0.57 (Petroleum ether /EtOAc = 20/1), stained by UV light (254, 365 nm). **^{1}H NMR** (400 MHz, $CDCl_3$) δ 7.99 (d, J = 8.5 Hz, 2H), 7.62 (d, J = 8.5 Hz, 2H), 1.36 (s, 9H), 1.24 (s, 9H); **^{13}C NMR**

(100 MHz, $CDCl_3$) δ 183.9, 159.8, 131.6 (d, J = 21.0 Hz), 127.9, 126.8, 42.4, 35.6, 31.1, 27.3; **^{19}F NMR** (376 MHz, $CDCl_3$) δ 66.0 (s, 1F); **HRMS** (ESI, m/z): calcd. for $C_{15}H_{22}FNNaO_2S$ $[M+Na]^+$: 322.1274, found: 322.1244.

4-methoxy-N-pivaloylbenzenesulfonimidoyl fluoride (**7i**)

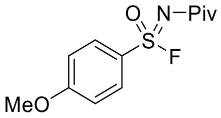

According to **General Procedure O** (2.7 mmol scale), **7c** was obtained as white solid (283.0 mg, 38% yield). R_f = 0.30 (Petroleum ether /EtOAc = 10/1), stained by UV light (254, 365 nm). **^{1}H NMR** (400 MHz, $CDCl_3$) δ 8.01 (d, J = 9.0 Hz, 2H), 7.06 (d, J = 9.0 Hz, 2H), 3.92 (s, 3H), 1.24 (s, 9H); **^{13}C NMR** (100 MHz, $CDCl_3$) δ 184.0, 165.2, 130.5, 125.7 (d, J = 21.0 Hz), 114.9, 56.1, 42.4, 27.3; **^{19}F NMR** (376 MHz, $CDCl_3$) δ 67.1 (s, 1F); **HRMS** (ESI, m/z): calcd. for $C_{12}H_{16}FNNaO_3S$ $[M+Na]^+$: 296.0727, found: 296.0730.

N-pivaloyl-4-(trifluoromethoxy)benzenesulfonimidoyl fluoride (**7j**)

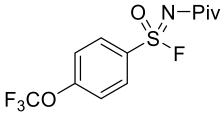

According to **General Procedure O** (3.0 mmol scale), **7d** was obtained as colorless oil (714.1 mg, 73% yield). R_f = 0.30 (Petroleum ether /EtOAc = 20/1), stained by UV light (254, 365 nm). **^{1}H NMR** (400 MHz, $CDCl_3$) δ 8.15 (d, J = 8.8 Hz, 2H), 7.45 (d, J = 8.6 Hz, 2H), 1.24 (s, 9H); **^{13}C NMR** (100 MHz, $CDCl_3$) δ 183.6, 154.3, 132.8 (d, J = 23.0 Hz), 130.5, 121.2, 120.3 (q, J = 259.0 Hz), 42.5, 27.2; **^{19}F NMR** (376 MHz, $CDCl_3$) δ -57.7 (s, 3F); **HRMS** (ESI, m/z): calcd. for $C_{12}H_{13}F_4NNaO_3S$ $[M+Na]^+$: 350.0444, found: 350.0445.

N-pivaloyl-4-(trifluoromethyl)benzenesulfonimidoyl fluoride (**7k**)

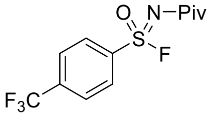

According to **General Procedure O** (3.0 mmol scale), **7e** was obtained as colorless oil (774.5 mg, 83% yield). R_f = 0.30 (Petroleum ether /EtOAc = 20/1), stained by UV light (254, 365 nm). **^{1}H NMR** (400 MHz, $CDCl_3$) δ 8.21 (d, J = 8.3 Hz, 2H), 7.90 (d, J = 8.3 Hz, 2H), 1.22 (s, 9H); **^{13}C NMR** (100 MHz, $CDCl_3$) δ 183.4, 138.3 (d, J = 23.0 Hz), 136.9 (q, J = 33.0 Hz), 128.6, 126.9 (q, J = 4.0 Hz), 122.9 (q, J = 272.0 Hz), 42.4, 27.0; **^{19}F NMR** (376 MHz, $CDCl_3$) δ 66.0 (s, 1F), -63.6 (s, 3F); **HRMS** (ESI, m/z): calcd. for $C_{12}H_{13}F_4NNaO_2S$ $[M+Na]^+$: 334.0495, found: 334.0495.

4-fluoro-N-pivaloylbenzenesulfonimidoyl fluoride (**7l**)

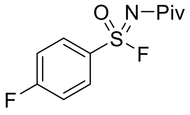

According to **General Procedure O** (4.7 mmol scale), **7f** was obtained as colorless oil (1.10 g, 86% yield). R_f = 0.51 (Petroleum ether /EtOAc = 10/1), stained by UV light (254, 365 nm). **^{1}H NMR** (400 MHz, $CDCl_3$) δ 8.13 – 8.10 (m, 2H), 7.31 (t, J = 8.4 Hz, 2H), 1.23 (s, 9H); **^{13}C NMR** (100 MHz, $CDCl_3$)

δ 183.6, 166.8 (d, *J* = 258.0 Hz), 131.1 (d, *J* = 10.0 Hz), 130.7 (dd, *J* = 22.0, 3.0 Hz), 117.2 (d, *J* = 23.0 Hz), 42.4, 27.2; **^{19}F NMR** (376 MHz, $CDCl_3$) δ 66.9 (s, 1F), -99.7 (s, 1F); **HRMS** (ESI, m/z): calcd. for $C_{11}H_{13}F_2NNaO_2S$ $[M+Na]^+$: 284.0527, found: 284.0530.

4-chloro-N-pivaloylbenzenesulfonimidoyl fluoride (**7m**)

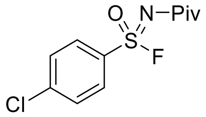

According to **General Procedure O** (3.0 mmol scale), **7g** was obtained as white solid (260.0 mg, 31% yield). R_f = 0.51 (Petroleum ether /EtOAc = 20/1), stained by UV light (254, 365 nm). **^{1}H NMR** (400 MHz, $CDCl_3$) δ 8.00 (d, *J* = 8.7 Hz, 2H), 7.58 (d, *J* = 8.7 Hz, 2H), 1.21 (s, 9H); **^{13}C NMR** (100 MHz, $CDCl_3$) δ 183.5, 142.4, 133.1 (d, *J* = 22.0 Hz), 130.0, 129.3, 42.3, 27.1; **^{19}F NMR** (376 MHz, $CDCl_3$) δ -66.6 (s, 1F); **HRMS** (ESI, m/z): calcd. for $C_{11}H_{13}ClFNaNO_2S$ $[M+Na]^+$: 300.0232, found: 300.0230.

3-chloro-N-pivaloylbenzenesulfonimidoyl fluoride (**7n**)

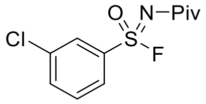

According to **General Procedure O** (10.5 mmol scale), **7h** was obtained as white solid (2.70 g, 94% yield). R_f = 0.50 (Petroleum ether /EtOAc = 10/1), stained by UV light (254, 365 nm). **^{1}H NMR** (400 MHz, $CDCl_3$) δ 8.05 (s, 1H), 7.97 (d, *J* = 8.0 Hz, 1H), 7.74 (d, *J* = 8.1 Hz, 1H), 7.58 (t, *J* = 8.0 Hz, 1H), 1.25 (s, 9H); **^{13}C NMR** (100 MHz, $CDCl_3$) δ 183.5, 136.4 (d, *J* = 23.0 Hz), 136.0, 135.6, 131.0, 128.0, 126.1, 42.5, 27.2; **^{19}F NMR** (376 MHz, $CDCl_3$) δ 66.5 (s, 1F); **HRMS** (ESI, m/z): calcd. for $C_{11}H_{13}ClFNNaO_2S$ $[M+Na]^+$: 300.0232, found: 300.0230.

N-pivaloyl-3,5-bis(trifluoromethyl)benzenesulfonimidoyl fluoride (**7o**)

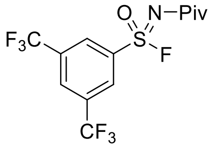

According to **General Procedure O** (3.0 mmol scale), **7i** was obtained as white solid (924.0 mg, 81% yield). R_f = 0.40 (Petroleum ether /EtOAc = 20/1), stained by UV light (254, 365 nm). **^{1}H NMR** (400 MHz, $CDCl_3$) δ 8.51 (s, 2H), 8.26 (s, 1H), 1.25 (s, 9H); **^{13}C NMR** (100 MHz, $CDCl_3$) δ 183.0, 137.8 (d, *J* = 65.0 Hz), 133.9 (q, *J* = 35.0 Hz), 129.0 (t, *J* = 3.0 Hz), 128.3 (d, *J* = 3.0 Hz), 122.2 (d, *J* = 272.0 Hz), 42.6, 27.1; **^{19}F NMR** (376 MHz, $CDCl_3$) δ 67.5 (s, 1F), 63.2 (s, 3F); **HRMS** (ESI, m/z): calcd. for $C_{13}H_{12}F_7NNaO_2S$ $[M+Na]^+$: 402.0369, found: 402.0370.

Section 3. The photochemical setup and optimization of reaction conditions

3.1 The photochemical setup

Wrap a 5 W lamp band around the outside of the beaker as a reaction equipment. At room temperature, the Schlenk tubes with reaction mixtures was set in the beaker.

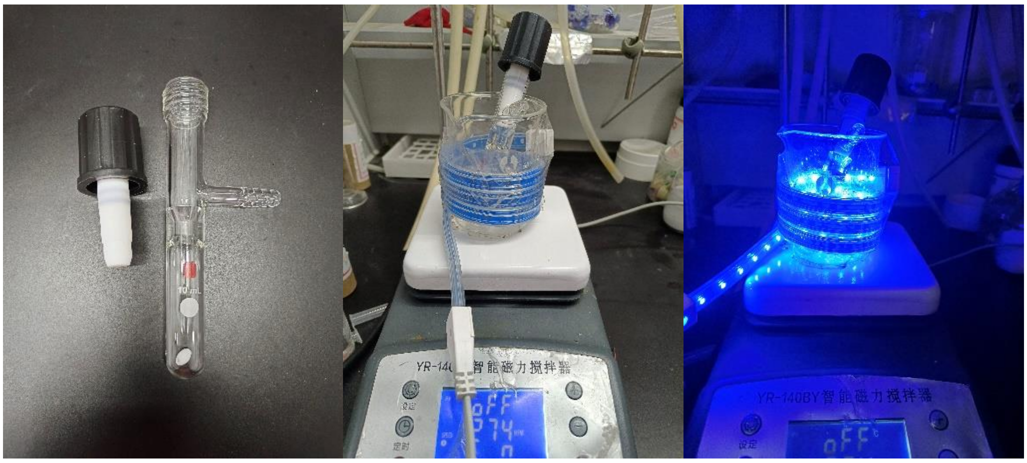

Supplementary Figure 5. The photochemical setup

3.2 Optimization of the reaction conditions

Supplementary Table 1. Evaluation of bases

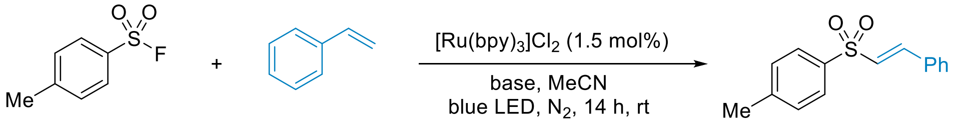

1a (0.40 mmol) **2a** (0.20 mmol) **3aa**

Entry	variations from the standard condition	Yield (%)[a]
1	DBU (3.5 eq.)	96
2	DBU (1.0 eq.)	12
3	DBU (2.0 eq.)	51
4	DBU (3.0 eq.)	88
5	DBU (4.0 eq.)	92
6	DBN	16
7	MTBD	21
8	TBD	7
9	BTMG	75
10	TMG	N.R.
11	1,3-diphenylguanidine	N.R.
12	KHMDS	N.R.
13	DIPEA	N.R.
14	Et_3N	N.R.
15	TMEDA	N.R.
16	DABCO	N.R.
17	Cy_2NMe	N.R.
18	N-methyl imidazole	N.R.
19	DMAP	N.R.
20	$CsCO_3$	N.R.
21	DIPEA (3.0 eq.)+DBU (0.5 eq.)	9
22	Na_2CO_3 (3.0 eq.)+DBU (0.5 eq.)	trace

[a]Yield was determined by ^{1}H NMR with CH_2Br_2 (0.10 mmol) as the internal standard. Reaction condition:**1a** (0.40 mmol), **2a** (0.20 mmol), base (0.70 mmol), $[Ru(bpy)_3]Cl_2$ (1.5 mol%), MeCN (2.0 mL), blue LED (5 W),stirred at rt for 14 h.

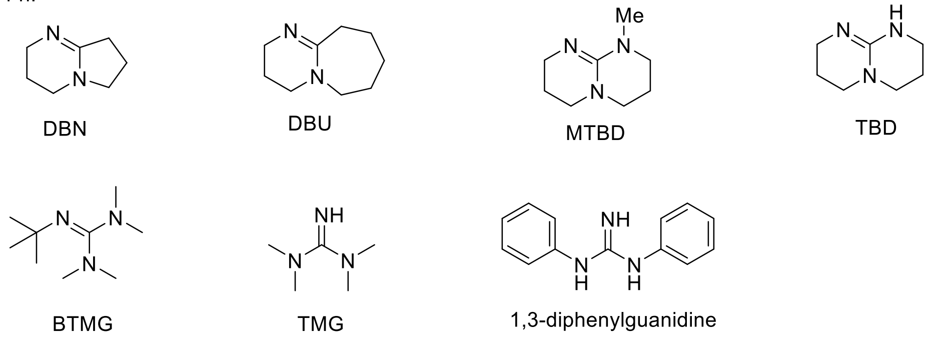

Supplementary Table 2. Evaluation of solvents

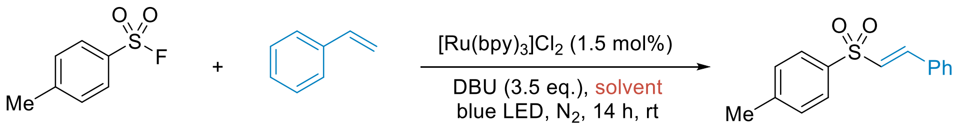

Entry	variations from the standard condition	Yield (%)[a]
1	MeCN (extra dry)	96
2	MeCN (regular)	45
3	CH_2Cl_2	N.R.
4	Acetone	N.R.
5	DCE	N.R.
6	THF	N.R.
7	DMF	74%
8	DMSO	99(85[b])

[a]Yield was determined by ^1H NMR with CH_2Br_2 (0.10 mmol) as the internal standard. [b]isolated yield. Reaction condition: **1a** (0.40 mmol), **2a** (0.20 mmol), DBU (0.70 mmol), $[Ru(bpy)_3]Cl_2$ (1.5 mol%), solvent (2.0 mL), blue LED (5 W), stirred at rt for 14 h.

Supplementary Table 3. Evaluation of photoredox catalysts

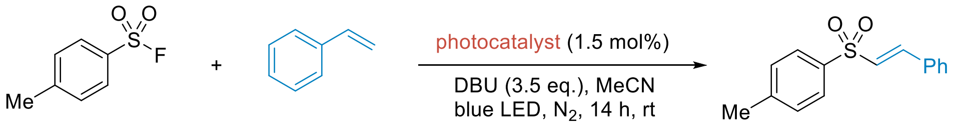

1a (0.40 mmol) + **2a** (0.20 mmol) → **3aa**

Entry	variations from the standard condition	Yield (%)[a]
1	$[Ir(dFppy)_2bpy]PF_6$	27
2	$[Ru(bpy)_3]Cl_2$ (0.5 mol%)	60
3	$[Ru(bpy)_3]Cl_2$ (1.0 mol%)	78
4	$[Ru(bpy)_3]Cl_2$ (1.5 mol%)	96
5	$[Ir(dF(CF_3)ppy)_2(dtbpy)]PF_6$	19
6	$Ir[(p\text{-}CF_3\text{-}ppy)_3]$	66
7	*fac*-$Ir(ppy)_3$	52
8	$Acr\text{-}Mes^+ ClO_4^-$	N.R.
9	Rhodamine B	trace
10	EosinY	8

[a]Yield was determined by ^1H NMR with CH_2Br_2 (0.10 mmol) as the internal standard. Reaction condition: **1a** (0.40 mmol), **2a** (0.20 mmol), DBU (0.70 mmol), photocatalyst (1.5 mol%), MeCN (2.0 mL), blue LED, stirred at rt for 14 h. EosinY: $E_{red}(EY^*/EY^+)$ = -1.1; Rhodamune B: E_{red} = -0.96.

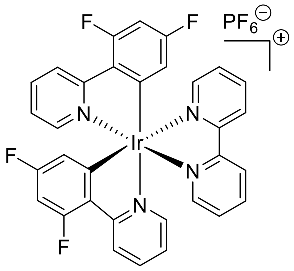

$[Ir(dFppy)_2bpy]PF_6$

$E_{1/2ox}$ = +1.66 V

$E_1/_2$ (M^+/M^*) = -0.80 V

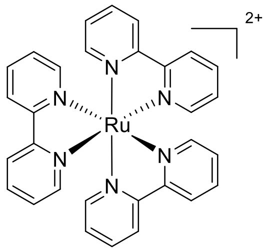

$[Ru(bpy)_3]Cl_2$

$E_{1/2ox}$ = +0.77 V

$E_1/_2$ (M^+/M^*) = -0.83 V

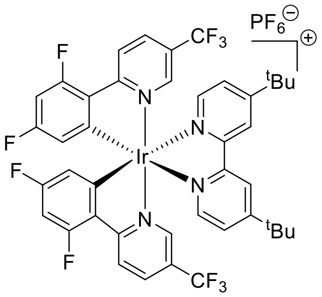

$(Ir[dF(CF_3)ppy]_2(dtbpy))PF_6$

$E_{1/2ox}$ = +1.29 V

$E_1/_2$ (M^+/M^*) = -0.89 V

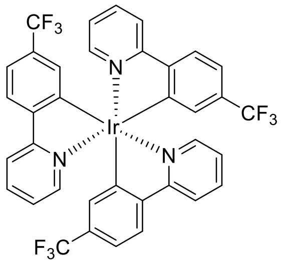

fac-$Ir[(p\text{-}CF_3\text{-}ppy)_3]$

$E_{1/2ox}$ = +0.59 V

$E_1/_2$ (M^+/M^*) = -1.695 V

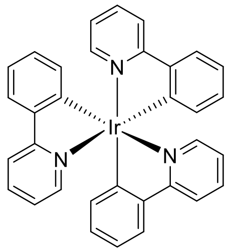

fac-$Ir(ppy)_3$

$E_{1/2ox}$ = +0.78 V

$E_1/_2$ (M^+/M^*) = -1.73 V

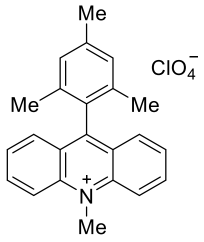

$Acr\text{-}Mes^+ClO_4^-$

Supplementary Table 4. Evaluation of the ratio of 1a and 2a

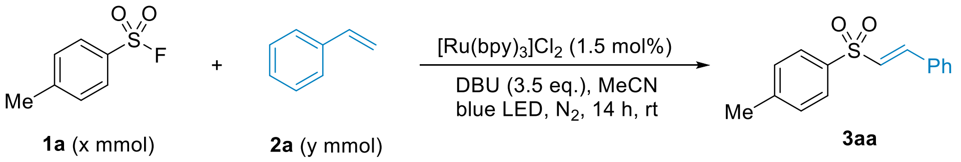

Entry	**1a**/**2a** (x mmol / y mmol)	Yield (%)[a]
1	0.4 / 0.2	96
2	0.3 / 0.2	82
3	0.2 / 0.2	51
4	0.2 / 0.3	48
5	0.2 / 0.4	28

[a]Yield was determined by ^{1}H NMR with CH_2Br_2 (0.10 mmol) as the internal standard. Reaction condition: **1a** (x mmol), **2a** (y mmol), DBU (0.70 mmol), $[Ru(bpy)_3]Cl_2$ (1.5 mol%), MeCN (2.0 mL), blue LED (5 W), stirred at rt for 14 h.

Supplementary Table 5. Control experiments

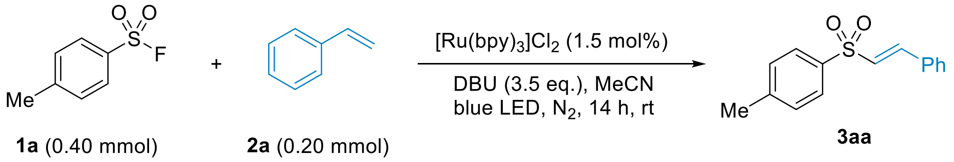

Entry	variations from the standard condition	Yield (%)[a]
1	none	96
2	no photocatalyst	N.R.
3	no base	N.R.
4	no light	N.R.
5	in air	13
6	Incandescent lamps	10
7	Incandescent lamps and no photocatalyst	N.R.

[a]Yield was determined by ^{1}H NMR with CH_2Br_2 (0.10 mmol) as the internal standard. Reaction condition: **1a** (0.40 mmol), **2a** (0.20 mmol), DBU (0.70 mmol), $[Ru(bpy)_3]Cl_2$ (1.5 mol%), MeCN (2.0 mL), blue LED (5 W), stirred at rt for 14 h.

3.3 Light on/off experiment

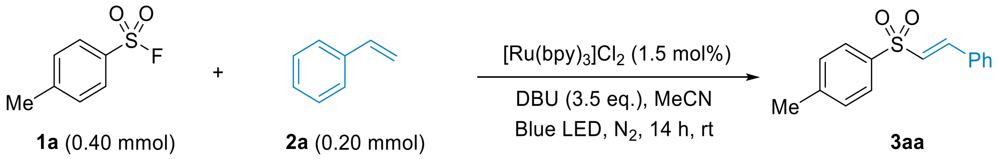

To a 10 mL Schlenk tube equipped with a magnetic stirring bar was charged **1a** (0.40 mmol, 2.0 eq.), $[Ru(bpy)_3]Cl_2$ (0.003 mmol, 1.5 mol%) under N_2 atmosphere. Subsequently, dry MeCN (2.0 mL), **2a** (0.20 mmol, 1.0 eq.), and DBU (0.70 mmol, 3.5 eq.) were added. The reaction was stirred and illuminated

with blue LED for 2 hours at room temperature, then 2 hours in darkness. This process was repeated within 12 hours. The product was monitored by GC-MS at each time interval.

Time	2.0 h	4.0 h	6.0 h	8.0 h	10.0 h	12.0 h
Yield (%)	25	25	40	40	50	50

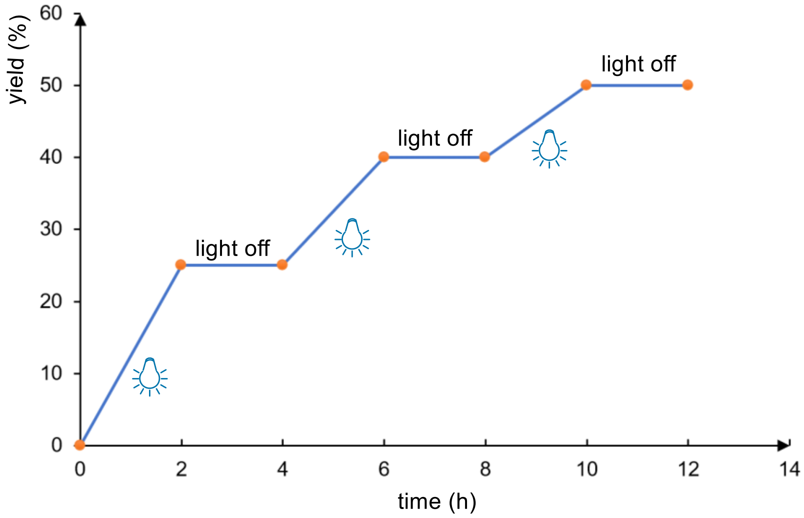

Supplementary Figure 6. Light on/off experiments

Section 4. The substrate scope

4.1 General procedures

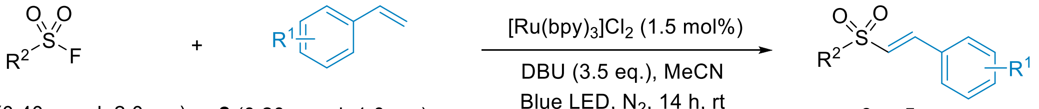

1 or **4** (0.40 mmol, 2.0 eq.) **2** (0.20 mmol, 1.0 eq.) **3** or **5**

General Procedure P: To a 10 mL Schlenk tube equipped with a magnetic stirring bar was charged sulfonyl fluoride (2.0 eq.) and $[Ru(bpy)_3]Cl_2$ (1.5 mol%) under the N_2 atmosphere. Subsequently, dry MeCN (0.10 M), olefins (1.0 eq.) and DBU (3.5 eq.) were added. The mixture was irradiated with blue LEDs and stirred at room temperature for 14 h. After that, the residue was purified by silica gel chromatography to give the product **3** or **5**.

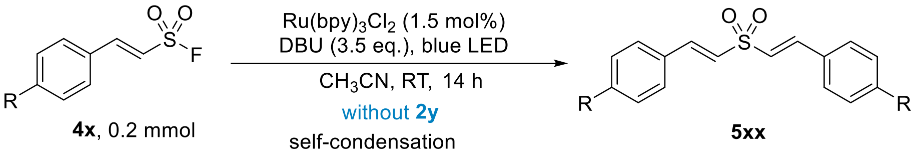

General Procedure Q: To a 10 mL Schlenk tube equipped with a magnetic stirring bar was charged sulfonyl fluoride (0.2 mmol) and $[Ru(bpy)_3]Cl_2$ (1.5 mol%) under the N_2 atmosphere. Subsequently, dry MeCN (0.10 M) and DBU (0.7 mmol) were added. The mixture was irradiated with blue LEDs and stirred at room temperature. After that, the residue was purified by silica gel chromatography to give the product **5xx**.

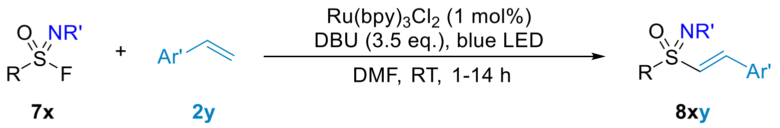

General Procedure R: To a 10 mL Schlenk tube equipped with a magnetic stirring bar was charged sulfonyl fluoride (2.0 eq.), and $[Ru(bpy)_3]Cl_2$ (1.0 mol%) under the N_2 atmosphere. Subsequently, dry DMF (0.10 M), olefins (1.0 eq.) and DBU (3.50 eq.) were added. The mixture was irradiated with blue LEDs and stirred at room temperature. Progress of the reaction was monitored by TLC. After completion, the residue was purified by silica gel chromatography to get the product **8**.

4.2 Standard condition

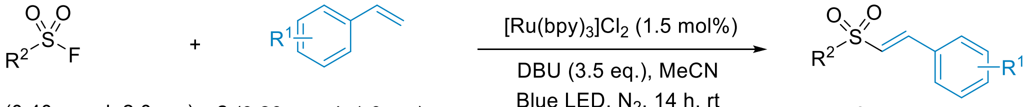

1 or 4 (0.40 mmol, 2.0 eq.) **2** (0.20 mmol, 1.0 eq.) **3** or **5**

To a 10 mL Schlenk tube equipped with a magnetic stirring bar was charged sulfonyl fluoride (0.40 mmol, 2.0 eq.), and $[Ru(bpy)_3]Cl_2$ (0.003 mmol, 1.5 mol%). Then the reaction tube was evacuated and backfilled with N_2 three times. Subsequently, dry MeCN (2.0 mL), olefins (0.20 mmol, 1.0 eq.) and DBU (0.70 mmol, 3.5 eq.) were added. The mixture was irradiated with blue LEDs and stirred at room temperature for 14 h. After that, the residue was purified by silica gel chromatography to give the product **3** or **5**.

4.3 Spectral characterization for products

(E)-1-methyl-4-(styrylsulfonyl)benzene (**3aa**)

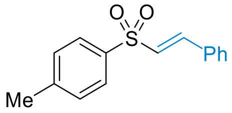

Compound **3aa** was prepared according to **General procedure P** (0.20 mmol scale, 14 h) to afford 46.2 mg (89%) as a white solid. R_f = 0.50 (Petroleum ether /EtOAc = 5/1); **m.p.** = 119 – 122 ºC; **^{1}H NMR** (400 MHz, $CDCl_3$) δ 7.83 (d, *J* = 8.3 Hz, 2H), 7.65 (d, *J* = 15.4 Hz, 1H), 7.48 – 7.46 (m, 2H), 7.40 – 7.37 (m, 3H), 7.34 (d, *J* = 8.0 Hz, 2H), 6.85 (d, *J* = 15.4 Hz, 1H), 2.43 (s, 3H); **^{13}C NMR** (100 MHz, $CDCl_3$) δ 144.5, 142.1, 137.9, 132.6, 131.2, 130.1, 129.2, 128.6, 127.8, 127.8, 216.7; **HRMS** (EI, m/z): calcd. for $C_{15}H_{13}O_2S$ $[M]^+$: 258.0709, found: 258.0711. The spectroscopic data of **3aa** matched the reported values.[29]

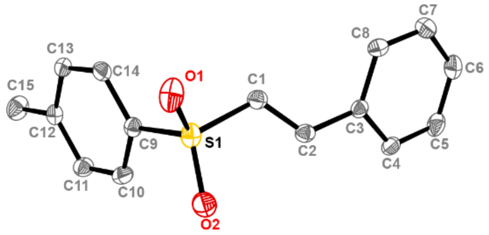

ORTEP drawing of **3aa** showing thermal ellipsoids at the 30% probability level

Crystal Data and Structure Refinement.

Identification code	CCDC 2180194	
Empirical formula	$C_{15}H_{13}O_2S$	
Formula weight	258.07	
Temperature	273.15 K	
Wavelength	0.71073 Å	
Crystal system	Orthorhombic	
Space group	$P2_12_12_1$	
Unit cell dimensions	a = 5.4880(9) Å	*α* = 90°.
	b = 8.1791(17) Å	*β* = 90°.
	c = 29.652(5) Å	*γ* = 90°.
Volume	1331.0(4) $Å^3$	
Z	22	
Density (calculated)	1.289 g/cm^3	
Absorption coefficient	0.234 mm^{-1}	
F(000)	544.0	
Crystal size	0.12 x 0.1 x 0.08 mm^3	
Radiation	MoKa (λ = 0.71073)	
Theta range for data collection	5.166 to 56.57°.	
Index ranges	-7<=h<=7, -10<=k<=7, -39<=l<=38	

Reflections collected	7370
Independent reflections	3263 [R(int) = 0.0384]
Completeness to theta = 56.57°	99.6 %
Absorption correction	Empirical
Refinement method	Full-matrix least-squares on F^2
Data / restraints / parameters	3263 / 0 / 164
Goodness-of-fit on F^2	1.024
Final R indices [I>2sigma(I)]	R1 = 0.0444, wR2 = 0.0920
R indices (all data)	R1 = 0.0589, wR2 = 0.0987
Largest diff. peak and hole	0.23 and -0.30 e.$Å^{-3}$

(E)-1-(tert-butyl)-4-(styrylsulfonyl)benzene (**3ba**)

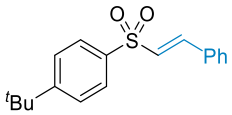

Compound **3ba** was prepared according to **General procedure P** (0.20 mmol scale, 14 h) to afford 58.2 mg (97%) as a white solid. R_f = 0.47 (Petroleum ether /EtOAc = 5/1); **m.p.** = 113 – 115 °C; **^{1}H NMR** (400 MHz, $CDCl_3$) δ 7.86 (d, *J* = 8.6 Hz, 2H), 7.67 (d, *J* = 15.4 Hz, 1H), 7.57 – 7.54 (m, 2H), 7.49 – 7.47 (m, 2H), 7.41 – 7.38 (m, 3H), 6.86 (d, *J* = 15.4 Hz, 1H), 1.34 (s, 9H); **^{13}C NMR** (100 MHz, $CDCl_3$) δ 157.5, 142.1, 137.8, 132.6, 131.2, 129.2, 128.7, 127.8, 127.7, 126.5, 35.4, 31.2; **HRMS** (EI, m/z): calcd. for $C_{15}H_{13}O_2S$ $[M]^+$: 300.1179, found: 300.1178. The spectroscopic data of **3ba** matched the reported values.[30]

(E)-4-(styrylsulfonyl)-1,1'-biphenyl (**3ca**)

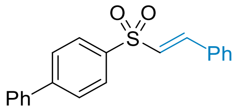

Compound **3ca** was prepared according to **General procedure P** (0.20 mmol scale, 14 h) to afford 63.0 mg (98%) as a white solid. R_f = 0.37 (Petroleum ether /EtOAc = 5/1); **m.p.** = 158 – 161 °C; **^{1}H NMR** (400 MHz, $CDCl_3$) δ 8.03 (d, *J* = 8.5 Hz, 2H), 7.76 – 7.71 (m, 3H), 7.61 – 7.59 (m, 2H), 7.51 – 7.46 (m, 4H), 7.44 – 7.39 (m, 4H), 6.93 (d, *J* = 15.4 Hz, 1H); **^{13}C NMR** (100 MHz, $CDCl_3$) δ 146.5, 142.5, 139.3, 139.3, 132.5, 131.3, 129.2, 129.2, 128.7, 128.7, 128.3, 128.1, 127.5, 127.4; **HRMS** (EI, m/z): calcd. for $C_{15}H_{13}O_2S$ $[M]^+$: 320.0866, found: 320.0865. The spectroscopic data of **3ca** matched the reported values.[30]

(E)-(2-(phenylsulfonyl)vinyl)benzene (**3da**)

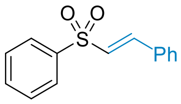

Compound **3da** was prepared according to **General procedure P** (0.20 mmol scale, 14 h) to afford 41.6 mg (86%) as a white solid (and 67% yield for 70 mins). R_f = 0.27 (Petroleum ether /EtOAc = 10/1); **m.p.** = 72 – 74 °C; **^{1}H NMR** (400 MHz, $CDCl_3$) δ 7.96 – 7.94 (m, 2H), 7.69 (d, *J* = 15.4 Hz, 1H), 7.63 – 7.59

(m, 1H), 7.56 – 7.52 (m, 2H), 7.48 (dd, *J* = 7.5, 1.9 Hz, 2H), 7.41 – 7.36 (m, 3H), 6.87 (d, *J* = 15.4 Hz, 1H); **^{13}C NMR** (100 MHz, $CDCl_3$) δ 142.6, 140.8, 133.5, 132.5, 131.3, 129.4, 129.2, 128.7, 127.7, 127.4; **HRMS** (EI, m/z): calcd. for $C_{14}H_{12}O_2S$ $[M]^+$: 244.0553, found: 244.0558. The spectroscopic data of **3da** matched the reported values.[30]

(E)-1-fluoro-4-(styrylsulfonyl)benzene (**3ea**)

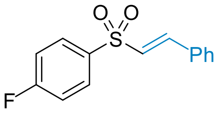

Compound **3ea** was prepared according to **General procedure P** (0.20 mmol scale, 14 h) to afford 47.6 mg (91%) as a white solid. R_f = 0.41 (Petroleum ether /EtOAc = 5/1); **m.p.** = 79 – 81 °C; **^{1}H NMR** (400 MHz, $CDCl_3$) δ 7.98 – 7.95 (m, 2H), 7.68 (d, *J* = 15.4 Hz, 1H), 7.47 (s, 2H), 7.40 – 7.38 (m, 3H), 7.23 – 7.17 (m, 2H), 6.89 – 6.84 (m, 1H); **^{13}C NMR** (100 MHz, $CDCl_3$) δ 165.7 (d, *J* = 254.0 Hz), 142.8, 137.0 (d, *J* = 3.0 Hz), 132.3, 131.4, 130.6 (d, *J* = 10.0 Hz), 129.2, 128.7, 127.3, 116.7 (d, *J* = 22.0 Hz); **^{19}F NMR** (376 MHz, $CDCl_3$) δ -104.0 (s, 1F); **HRMS** (EI, m/z): calcd. for $C_{14}H_{11}FO_2S$ $[M]^+$: 262.0458, found: 262.0457. The spectroscopic data of **3ea** matched the reported values.[29]

(E)-1-(styrylsulfonyl)-4-(trifluoromethyl)benzene (**3fa**)

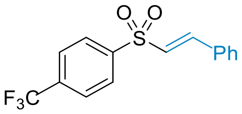

Compound **3fa** was prepared according to **General procedure P** (0.20 mmol scale, 14 h) to afford 58.9 mg (95%) as a white solid (and 92% yield for 5 mins). R_f = 0.51 (Petroleum ether /EtOAc = 5/1); **m.p.** = 129 – 130 °C; **^{1}H NMR** (400 MHz, $CDCl_3$) δ 8.09 (d, *J* = 8.2 Hz, 2H), 7.81 (d, *J* = 8.1 Hz, 2H), 7.74 (d, *J* = 15.4 Hz, 1H), 7.51 – 7.49 (m, 2H), 7.46 – 7.39 (m, 3H), 6.89 – 6.84 (m, 1H); **^{13}C NMR** (100 MHz, $CDCl_3$) δ 144.5, 144.2, 135.2 (q, *J* = 3.3 Hz), 132.2, 131.8, 129.3, 128.9, 128.4, 126.6 (q, *J* = 3.0 Hz), 126.4, 123.3 (q, *J* = 271.0 Hz); **^{19}F NMR** (376 MHz, $CDCl_3$) δ -63.2 (s, 3F); **HRMS** (EI, m/z): calcd. for $C_{15}H_{11}F_3O_2S$ $[M]^+$: 312.0426, found: 312.0421. The spectroscopic data of **3fa** matched the reported values.[29]

(E)-1-(styrylsulfonyl)-4-(trifluoromethoxy)benzene (**3ga**)

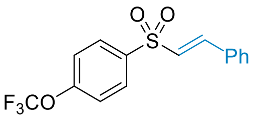

Compound **3ga** was prepared according to **General procedure P** (0.20 mmol scale, 14 h) to afford 65.0 mg (98%) as a white solid. R_f = 0.50 (Petroleum ether /EtOAc = 5/1); **m.p.** = 92 – 96 °C; **^{1}H NMR** (400 MHz, $CDCl_3$) δ 8.02 – 7.99 (m, 2H), 7.71 (d, *J* = 15.4 Hz, 1H), 7.49 (dd, *J* = 7.6, 1.7 Hz, 2H), 7.42 – 7.35 (m, 5H), 6.87 (d, *J* = 15.4 Hz, 1H); **^{13}C NMR** (100 MHz, $CDCl_3$) δ 152.8 (q, *J* = 2.0 Hz), 143.4, 139.2, 132.2, 131.6, 130.0, 129.2, 128.8, 126.9, 121.3 (d, *J* = 1.0 Hz), 120.3 (q, *J* = 258.0 Hz); **^{19}F NMR** (376 MHz, $CDCl_3$) δ -57.7 (s, 3F); **HRMS** (EI, m/z): calcd. for $C_{15}H_{11}F_3O_3S$ $[M]^+$: 328.0376, found: 328.0370. The spectroscopic data of **3ga** matched the reported values.[30]

(E)-1-bromo-4-(styrylsulfonyl)benzene (**3ha**)

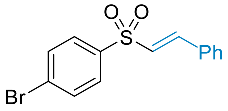

Compound **3ha** was prepared according to **General procedure P** (0.20 mmol scale, 14 h) to afford 57.5 mg (89%) as a white solid. R_f = 0.47 (Petroleum ether /EtOAc = 5/1); **m.p.** = 99 – 101 °C; **^{1}H NMR** (400 MHz, $CDCl_3$) δ 7.82 – 7.79 (m, 2H), 7.70 – 7.67 (m, 3H), 7.48 (dd, *J* = 7.6, 1.6 Hz, 2H), 7.42 – 7.37 (m, 3H), 6.84 (d, *J* = 15.4 Hz, 1H); **^{13}C NMR** (100 MHz, $CDCl_3$) δ 143.2, 139.9, 132.8, 132.3, 131.5, 129.3, 129.3, 128.8, 126.9; **HRMS** (EI, m/z): calcd. for $C_{14}H_{11}BrO_2S$ $[M]^+$: 321.9658, found: 321.9660. The spectroscopic data of **3ha** matched the reported values.[29]

(E)-4-(styrylsulfonyl)benzonitrile (**3ia**)

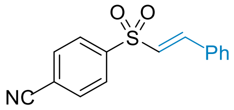

Compound **3ia** was prepared according to **General procedure P** (0.20 mmol scale, 14 h) to afford 49.1 mg (91%) as a white solid (and 80% yield for 5 mins). R_f = 0.33 (Petroleum ether /EtOAc = 5/1); **m.p.** = 121 – 124 °C; **^{1}H NMR** (400 MHz, $CDCl_3$) δ 8.06 (d, *J* = 8.4 Hz, 2H), 7.84 (d, *J* = 8.4 Hz, 2H), 7.74 (d, *J* = 15.4 Hz, 1H), 7.50 (d, *J* = 6.8 Hz, 2H), 7.45 – 7.38 (m, 3H), 6.84 (d, *J* = 15.4 Hz, 1H); **^{13}C NMR** (100 MHz, $CDCl_3$) δ 145.2, 144.8, 133.2, 132.0, 131.9, 129.4, 128.9, 128.4, 125.9, 117.3, 117.2; **HRMS** (EI, m/z): calcd. for $C_{15}H_{11}NO_2S$ $[M]^+$: 269.0505, found: 269.0503. The spectroscopic data of **3ia** matched the reported values.[30]

Methyl *(E)*-4-(styrylsulfonyl)benzoate (**3ja**)

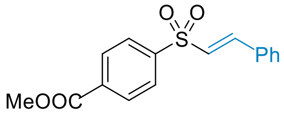

Compound **3ja** was prepared according to **General procedure P** (0.20 mmol scale, 14 h) to afford 51.0 mg (84%) as a white solid. R_f = 0.41 (Petroleum ether /EtOAc = 3/1); **m.p.** = 112 – 115 °C; **^{1}H NMR** (400 MHz, $CDCl_3$) δ 8.19 (d, *J* = 8.2 Hz, 2H), 8.01 (d, *J* = 8.4 Hz, 2H), 7.72 (d, *J* = 15.4 Hz, 1H), 7.50 – 7.48 (m, 2H), 7.43 – 7.37 (m, 3H), 6.86 (d, *J* = 15.4 Hz, 1H), 3.95 (s, 3H); **^{13}C NMR** (100 MHz, $CDCl_3$) δ 165.6, 144.8, 143.8, 134.6, 132.3, 131.6, 130.6, 129.3, 128.8, 127.8, 126.6, 52.8; **HRMS** (EI, m/z): calcd. for $C_{16}H_{14}O_4S$ $[M]^+$: 302.0607, found: 302.0610. The spectroscopic data of **3ja** matched the reported values.[31]

(E)-1-methyl-3-(styrylsulfonyl)benzene (**3ka**)

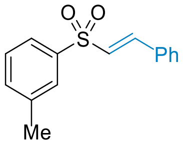

Compound **3ka** was prepared according to **General procedure P** (0.20 mmol scale, 14 h) to afford 43.0 mg (83%) as a colorless oil. R_f = 0.43 (Petroleum ether /EtOAc = 5/1); **^{1}H NMR** (400 MHz, $CDCl_3$) δ 7.75 (d, *J* = 5.3 Hz, 2H), 7.67 (d, *J* = 15.4 Hz, 1H), 7.49 – 7.47 (m, 2H), 7.43 – 7.36 (m, 5H), 6.87 (d, *J* = 15.4 Hz, 1H), 2.42 (s, 3H); **^{13}C NMR** (100 MHz, $CDCl_3$) δ 142.3, 140.6, 139.7, 134.3, 132.5, 131.2,

129.3, 129.2, 128.6, 128.0, 127.5, 124.9, 21.4; **HRMS** (EI, m/z): calcd. for $C_{15}H_{14}O_2S$ $[M]^+$: 258.0709, found: 258.0706. The spectroscopic data of **3ka** matched the reported values.[30]

(E)-1-(styrylsulfonyl)-3,5-bis(trifluoromethyl)benzene (**3la**)

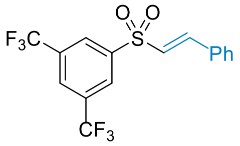

Compound **3la** was prepared according to **General procedure P** (0.20 mmol scale, 14 h) to afford 64.1 mg (84%) as a white solid. R_f = 0.55 (Petroleum ether /EtOAc = 20/1); **m.p.** = 110 – 113 °C; **^{1}H NMR** (400 MHz, $CDCl_3$) δ 8.41 (s, 2H), 8.10 (s, 1H), 7.81 (d, *J* = 15.4 Hz, 1H), 7.53 (d, *J* = 6.8 Hz, 2H), 7.48 – 7.40 (m, 3H), 6.89 (d, *J* = 15.4 Hz, 1H); **^{13}C NMR** (100 MHz, $CDCl_3$) δ 145.6, 144.0, 133.4 (q, *J* = 34.0 Hz), 132.1, 131.9, 129.4, 129.1, 128.2 (d, *J* = 3.0 Hz), 127.1 (q, *J* = 4.0 Hz), 125.5, 122.5 (q, *J* = 272 Hz); **^{19}F NMR** (376 MHz, $CDCl_3$) δ -63.0 (s, 3F); **HRMS** (EI, m/z): calcd. for $C_{16}H_{10}F_6O_2S$ $[M]^+$: 380.0300, found: 380.0301.

(E)-1-bromo-2-(styrylsulfonyl)benzene (**3ma**)

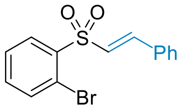

Compound **3ma** was prepared according to **General procedure P** (0.20 mmol scale, 14 h) to afford 30.0 mg (46%) as a white solid. R_f = 0.43 (Petroleum ether /EtOAc = 5/1); **m.p.** = 96 – 98 °C; **^{1}H NMR** (400 MHz, $CDCl_3$) δ 8.27 (dd, *J* = 7.9, 1.4 Hz, 1H), 7.79 (d, *J* = 15.4 Hz, 1H), 7.73 (d, *J* = 7.8 Hz, 1H), 7.54 – 7.51 (m, 3H), 7.46 – 7.38 (m, 4H), 7.11 (d, *J* = 15.4 Hz, 1H); **^{13}C NMR** (100 MHz, $CDCl_3$) δ 145.6, 140.1, 135.6, 134.6, 132.5, 131.6, 131.2, 129.3, 128.8, 128.2, 125.2, 121.2; **HRMS** (EI, m/z): calcd. for $C_{14}H_{11}BrO_2S$ $[M]^+$: 321.9663, found: 321.9662. The spectroscopic data of **3ma** matched the reported values.[31]

(E)-4-methyl-7-(styrylsulfonyl)-2H-chromen-2-one (**3na**)

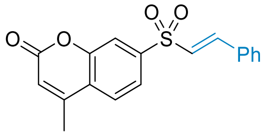

Compound **3na** was prepared according to **General procedure P** (0.20 mmol scale, 14 h) to afford 20.6 mg (32%) as a white solid. R_f = 0.16 (Petroleum ether /EtOAc = 3/1); **m.p.** = 166 – 169 °C; **^{1}H NMR** (400 MHz, $CDCl_3$) δ 7.83 (d, *J* = 8.4 Hz, 2H), 7.77 – 7.69 (m, 2H), 7.49 (d, *J* = 7.1 Hz, 2H), 7.42 – 7.37 (m, 3H), 6.88 (d, *J* = 15.4 Hz, 1H), 6.39 (s, 1H), 2.46 (s, 3H); **^{13}C NMR** (100 MHz, $CDCl_3$) δ 159.4, 153.4, 151.2, 144.2, 143.7, 132.1, 131.7, 129.3, 128.8, 126.3, 126.1, 123.8, 122.8, 117.8, 116.5, 18.8; **HRMS** (EI, m/z): calcd. for $C_{18}H_{14}O_4S$ $[M]^+$: 326.0607, found: 326.0611.

Ethyl *(E)*-5-(styrylsulfonyl)benzofuran-2-carboxylate (**3oa**)

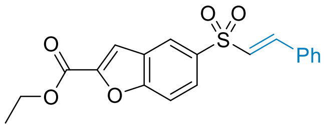

Compound **3oa** was prepared according to **General procedure P** (0.20 mmol scale, 14 h) to afford 62.5 mg (88%) as a white solid. R_f = 0.20 (Petroleum ether /EtOAc = 5/1); **m.p.** = 131 – 134 °C; **^{1}H NMR** (400 MHz, $CDCl_3$) δ 8.35 (s, 1H), 7.99 – 7.97 (m, 1H), 7.70 (d, *J* = 2.5 Hz, 1H), 7.67 (d, *J* = 3.6 Hz, 1H), 7.57 (s, 1H), 7.46 (d, *J* = 7.7 Hz, 2H), 7.39 – 7.33 (m, 3H), 6.90 (d, *J* = 15.4 Hz, 1H), 4.43 (q, *J* = 7.1 Hz, 2H), 1.41 (t, *J* = 7.1 Hz, 3H); **^{13}C NMR** (100 MHz, $CDCl_3$) δ 158.8, 157.5, 148.0, 142.5, 136.8, 132.3, 131.3, 129.1, 128.6, 127.5, 127.4, 126.7, 123.8, 113.6, 113.5, 62.0, 14.3; **HRMS** (EI, m/z): calcd. for $C_{19}H_{16}O_5S$ $[M]^+$: 356.0713, found: 356.0713.

(E)-2-(styrylsulfonyl)thiophene (**3pa**)

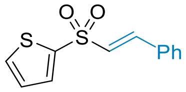

Compound **3pa** was prepared according to **General procedure P** (0.20 mmol scale, 14 h) to afford 40.0 mg (80%) as a white solid. R_f = 0.33 (Petroleum ether /EtOAc = 10/1); **m.p.** = 98 – 102 °C; **^{1}H NMR** (400 MHz, $CDCl_3$) δ 7.72 (dd, *J* = 3.8, 1.3 Hz, 1H), 7.69 – 7.66 (m, 2H), 7.50 – 7.48 (m, 2H), 7.44 – 7.37 (m, 3H), 7.14 – 7.12 (m, 1H), 6.96 (d, *J* = 15.4 Hz, 1H); **^{13}C NMR** (100 MHz, $CDCl_3$) δ 142.4, 142.3, 134.0, 133.6, 132.4, 131.4, 129.2, 128.7, 128.1, 128.0; **HRMS** (EI, m/z): calcd. for $C_{12}H_9O_2S$ $[M]^+$: 250.0117, found: 250.0119. The spectroscopic data of **3pa** matched the reported values.[29]

(E)-5-(styrylsulfonyl)benzo*[d]*thiazole (**3qa**)

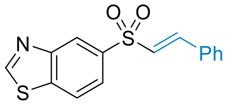

Compound **3qa** was prepared according to **General procedure P** (0.20 mmol scale, 14 h) to afford 57.9 mg (96%) as a white solid. R_f = 0.20 (Petroleum ether /EtOAc = 3/1); **m.p.** = 144 – 147 °C; **^{1}H NMR** (400 MHz, $CDCl_3$) δ 9.13 (s, 1H), 8.71 (s, 1H), 8.10 (d, *J* = 8.4 Hz, 1H), 7.97 (d, *J* = 8.4 Hz, 1H), 7.72 (d, *J* = 15.4 Hz, 1H), 7.46 (d, *J* = 6.8 Hz, 2H), 7.39 – 7.35 (m, 3H), 6.92 (d, *J* = 15.4 Hz, 1H); **^{13}C NMR** (100 MHz, $CDCl_3$) δ 156.8, 153.1, 142.9, 139.2, 139.1, 132.2, 131.4, 129.1, 128.6 127.2, 123.7, 123.5, 123.2; **HRMS** (EI, m/z): calcd. for $C_{15}H_{11}NO_2S_2$ $[M]^+$: 301.0226, found: 301.0227.

(E)-8-(styrylsulfonyl)quinoline (**3ra**)

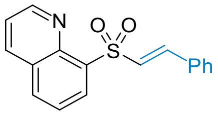

Compound **3ra** was prepared according to **General procedure P** (0.20 mmol scale, 14 h) to afford 56.4 mg (95%) as a white solid. R_f = 0.35 (Petroleum ether /EtOAc = 2/1); **m.p.** = 138 – 142 °C; **^{1}H NMR** (400 MHz, $CDCl_3$) δ 9.13 (dd, *J* = 4.3, 1.7 Hz, 1H), 8.56 (dd, *J* = 7.4, 1.4 Hz, 1H), 8.23 (dd, *J* = 8.3, 1.7 Hz, 1H), 8.06 (dd, *J* = 8.2, 1.3 Hz, 1H), 7.87 (d, *J* = 15.6 Hz, 1H), 7.77 (d, *J* = 15.6 Hz, 1H), 7.64 (t, *J* = 8.0 Hz, 1H), 7.54 – 7.51 (m, 1H), 7.48 (dd, *J* = 6.6, 3.1 Hz, 2H), 7.35 – 7.32 (m, 3H); **^{13}C NMR** (100 MHz, $CDCl_3$) δ 151.5, 144.1, 143.2, 138.4, 136.7, 134.2, 133.0, 131.0, 130.6, 129.0, 128.6, 128.4, 125.7, 122.3; **HRMS** (EI, m/z): calcd. for $C_{17}H_{13}NO_2S$ $[M+H]^+$: 295.0662, found: 295.0663. The spectroscopic data of **3ra** matched the reported values.[32]

(E)-6-(styrylsulfonyl)quinoline (**3sa**)

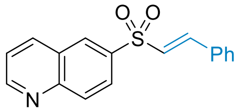

Compound **3sa** was prepared according to **General procedure P** (0.20 mmol scale, 14 h) to afford 45.5 mg (77%) as a white solid. R_f = 0.10 (Petroleum ether /EtOAc = 3/1); **m.p.** = 90 – 94 °C; **^{1}H NMR** (400 MHz, $CDCl_3$) δ 9.03 (s, 1H), 8.53 (s, 1H), 8.28 (d, *J* = 8.3 Hz, 1H), 8.21 (d, *J* = 8.9 Hz, 1H), 8.10 (d, *J* = 8.9 Hz, 1H), 7.75 (d, *J* = 15.4 Hz, 1H), 7.53 – 7.50 (m, 1H), 7.47 (d, *J* = 7.1 Hz, 2H), 7.37 – 7.35 (m, 3H), 6.93 (d, *J* = 15.4 Hz, 1H); **^{13}C NMR** (100 MHz, $CDCl_3$) δ 153.4, 149.6, 143.4, 138.5, 137.4, 132.2, 131.5, 131.4, 129.3, 129.2, 128.7, 127.5, 126.9, 126.4, 122.7; **HRMS** (EI, m/z): calcd. for $C_{17}H_{13}NO_2S$ $[M]^+$: 295.0662, found: 295.0663.

(E)-3-(styrylsulfonyl)quinoline (**3ta**)

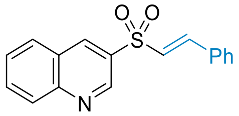

Compound **3ta** was prepared according to **General procedure P** (0.20 mmol scale, 14 h) to afford 55.5 mg (94%) as a white solid. R_f = 0.21 (Petroleum ether /EtOAc = 5/1); **m.p.** = 137 – 141 °C; **^{1}H NMR** (400 MHz, $CDCl_3$) δ 9.28 (s, 1H), 8.78 (s, 1H), 8.16 (d, *J* = 8.5 Hz, 1H), 7.94 (d, *J* = 8.2 Hz, 1H), 7.86 (t, *J* = 7.7 Hz, 1H), 7.78 (d, *J* = 15.4 Hz, 1H), 7.66 (t, *J* = 7.5 Hz, 1H), 7.47 (d, *J* = 7.0 Hz, 2H), 7.40 – 7.34 (m, 3H), 6.95 (d, *J* = 15.4 Hz, 1H); **^{13}C NMR** (100 MHz, $CDCl_3$) δ 149.5, 147.1, 143.9, 137.2, 133.8, 132.8, 132.1, 131.6, 129.6, 129.2, 129.2, 128.8, 128.4, 126.8, 126.4; **HRMS** (EI, m/z): calcd. for $C_{17}H_{13}NO_2S$ $[M]^+$: 295.0662, found: 295.0663.

(E)-2-(styrylsulfonyl)pyridine (**3ua**)

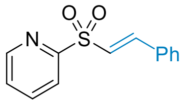

Compound **3ua** was prepared according to **General procedure P** (0.20 mmol scale, 14 h) to afford 19.6 mg (39%) as a white solid. R_f = 0.30 (Petroleum ether /EtOAc = 2/1); **m.p.** = 94 – 98 °C; **^{1}H NMR** (400 MHz, $CDCl_3$) δ 8.74 (d, *J* = 4.6 Hz, 1H), 8.14 (d, *J* = 7.9 Hz, 1H), 7.95 (td, *J* = 7.8, 1.5 Hz, 1H), 7.78 (d, *J* = 15.5 Hz, 1H), 7.53 – 7.50 (m, 3H), 7.45 – 7.37 (m, 3H), 7.11 (d, *J* = 15.5 Hz, 1H); **^{13}C NMR** (100 MHz, $CDCl_3$) δ 158.7, 150.5, 145.2, 138.3, 132.5, 131.5, 129.2, 128.9, 127.2, 124.8, 122.0; **HRMS** (EI, m/z): calcd. for $C_{13}H_{11}NO_2S$ $[M]^+$: 245.0505, found: 245.0507. The spectroscopic data of **3ua** matched the reported values.[33]

(E)-3-methyl-6-(styrylsulfonyl)quinazolin-4(3*H*)-one (**3va**)

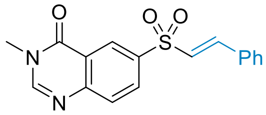

Compound **3va** was prepared according to **General procedure P** (0.20 mmol scale, 14 h) to afford 57.8 mg (89%) as a white solid. R_f = 0.10 (Petroleum ether /EtOAc = 1/1); **m.p.** = 182 – 185 °C; **^{1}H NMR** (400 MHz, $CDCl_3$) δ 8.82 (s, 1H), 8.17 (d, *J* = 8.6 Hz, 1H), 8.13 (s, 1H), 7.77 (d, *J* = 8.6 Hz, 1H), 7.69 (d, *J* = 15.4 Hz, 1H), 7.46 (d, *J* = 7.7 Hz, 2H), 7.41 – 7.34 (m, 3H), 6.89 (d, *J* = 15.4 Hz, 1H), 3.59 (s, 3H); **^{13}C NMR** (100 MHz, $CDCl_3$) δ 160.5, 151.4, 149.5, 143.6, 139.3, 132.2, 132.0, 131.5, 129.2, 129.1,

128.8, 127.6, 126.8, 122.3, 34.4; **HRMS** (EI, m/z): calcd. for $C_{17}H_{14}N_2O_3S$ $[M]^+$: 326.0720, found: 326.0719.

(E)-1-methyl-5-(styrylsulfonyl)-1H-indazole (**3wa**)

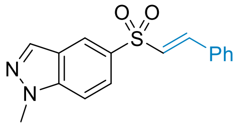

Compound **3wa** was prepared according to **General procedure P** (0.20 mmol scale, 14 h) to afford 31.4 mg (53%) as a white solid. R_f = 0.14 (Petroleum ether /EtOAc = 3/1); **m.p.** = 144 – 147 °C; **^{1}H NMR** (400 MHz, $CDCl_3$) δ 8.41 (s, 1H), 8.11 (s, 1H), 7.86 (d, *J* = 8.3 Hz, 1H), 7.67 (d, *J* = 15.4 Hz, 1H), 7.50 (d, *J* = 8.9 Hz, 1H), 7.46 – 7.44 (m, 2H), 7.36 (s, 3H), 6.89 (d, *J* = 15.4 Hz, 1H), 4.10 (s, 3H); **^{13}C NMR** (100 MHz, $CDCl_3$) δ 141.8, 141.2, 134.7, 132.9, 132.5, 131.2, 129.1, 128.6, 127.9, 124.7, 123.5, 123.3, 110.1, 36.0; **HRMS** (EI, m/z): calcd. for $C_{16}H_{14}N_2O_2S$ $[M]^+$: 298.0770, found: 298.0769.

(E)-1-(4-(styrylsulfonyl)phenyl)-5-(p-tolyl)-3-(trifluoromethyl)-1H-pyrazole (**3xa**)

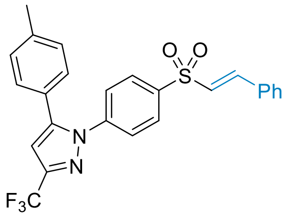

Compound **3xa** was prepared according to **General procedure P** (0.20 mmol scale, 14 h) to afford 79.3 mg (85%) as a white solid. R_f = 0.38 (Petroleum ether /EtOAc = 5/1); **m.p.** = 172 – 175 °C; **^{1}H NMR** (400 MHz, $CDCl_3$) δ 7.93 (d, *J* = 8.6 Hz, 2H), 7.69 (d, *J* = 15.4 Hz, 1H), 7.52 – 7.47 (m, 4H), 7.44 – 7.37 (m, 3H), 7.18 (d, *J* = 8.0 Hz, 2H), 7.12 (d, *J* = 8.1 Hz, 2H), 6.86 (d, *J* = 15.4 Hz, 1H), 6.74 (s, 1H), 2.38 (s, 3H); **^{13}C NMR** (100 MHz, $CDCl_3$) δ 145.3, 144.2 (q, *J* = 38.0 Hz), 143.4, 143.2, 140.2, 139.9, 132.2, 131.5, 129.9, 129.2, 128.8 (t, *J* = 4.0 Hz), 126.8, 125.7, 125.6 121.1 (q, *J* = 267.0 Hz), 106.5, 21.4; **^{19}F NMR** (376 MHz, $CDCl_3$) δ -62.4 (s, 3F); **HRMS** (EI, m/z): calcd. for $C_{25}H_{19}F_3N_2O_2S$ $[M]^+$: 468.1114, found: 468.1113.

(E)-2-((2-chloro-4-(styrylsulfonyl)phenoxy)methyl)pyridine (**3ya**)

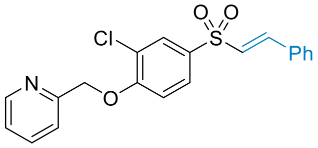

Compound **3ya** was prepared according to **General procedure P** (0.20 mmol scale, 14 h) to afford 64.6 mg (84%) as a white solid. R_f = 0.36 (Petroleum ether /EtOAc = 1/1); **m.p.** = 167 – 170 °C; **^{1}H NMR** (400 MHz, $CDCl_3$) δ 8.56 (d, *J* = 4.6 Hz, 1H), 7.95 (s, 1H), 7.77 (d, *J* = 8.7 Hz, 1H), 7.72 (t, *J* = 7.7 Hz, 1H), 7.62 (d, *J* = 15.4 Hz, 1H), 7.55 (d, *J* = 7.8 Hz, 1H), 7.46 – 7.44 (m, 2H), 7.38 – 7.34 (m, 3H), 7.24 – 7.21 (m, 1H), 7.09 (d, *J* = 8.7 Hz, 1H), 6.83 (d, *J* = 15.4 Hz, 1H), 5.31 (s, 2H); **^{13}C NMR** (100 MHz, $CDCl_3$) δ 157.8, 155.5, 149.3, 142.4, 137.2, 133.6, 132.3, 131.3, 129.9, 129.1, 128.6, 128.1, 127.3, 124.1, 123.1, 121.3, 113.6, 71.6; **HRMS** (ESI, m/z): calcd. for $C_{20}H_{17}ClNNaO_3S$ $[M+Na]^+$: 408.0432, found: 408.0438.

(E)-1-methyl-4-(2-((4-(trifluoromethyl)phenyl)sulfonyl)vinyl)benzene (**3fb**)

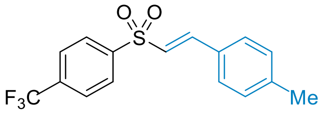

Compound **3fb** was prepared according to **General procedure P** (0.20 mmol scale, 14 h) to afford 63.4 mg (97%) as a white solid (and 86% yield for 10 mins). R_f = 0.26 (Petroleum ether /EtOAc = 20/1); **m.p.** = 170 – 173 °C; **^{1}H NMR** (400 MHz, $CDCl_3$) δ 8.08 (d, *J* = 8.2 Hz, 2H), 7.80 (d, *J* = 8.3 Hz, 2H), 7.71 (d, *J* = 15.4 Hz, 1H), 7.39 (d, *J* = 8.1 Hz, 2H), 7.20 (d, *J* = 8.0 Hz, 2H), 6.81 (d, *J* = 15.4 Hz, 1H), 2.37 (s, 3H); **^{13}C NMR** (100 MHz, $CDCl_3$) δ 144.8, 144.2, 142.5, 135.0 (q, *J* = 33.0 Hz), 130.0, 129.5, 128.9, 128.3, 126.6 (q, *J* = 4.0 Hz), 125.2, 123.3 (q, *J* = 271.0 Hz), 21.6; **^{19}F NMR** (376 MHz, $CDCl_3$) δ -63.2 (s, 3F); **HRMS** (EI, m/z): calcd. for $C_{16}H_{13}F_3O_2S$ $[M]^+$: 326.0583, found: 326.0585. The spectroscopic data of **3fb** matched the reported values.[34]

(E)-1-(tert-butyl)-4-(2-((4-(trifluoromethyl)phenyl)sulfonyl)vinyl)benzene (**3fc**)

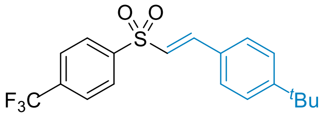

Compound **3fc** was prepared according to **General procedure P** (0.20 mmol scale, 14 h) to afford 65.0 mg (88%) as a white solid (and 91% yield for 5 mins). R_f = 0.23 (Petroleum ether /EtOAc = 20/1); **m.p.** = 152 – 154 °C; **^{1}H NMR** (400 MHz, $CDCl_3$) δ 8.09 (d, *J* = 7.6 Hz, 2H), 7.80 (d, *J* = 7.3 Hz, 2H), 7.74 (dd, *J* = 15.4, 2.4 Hz, 1H), 7.44 (s, 4H), 6.84 (dd, *J* = 15.4, 2.6 Hz, 1H), 1.31 (s, 9H); **^{13}C NMR** (100 MHz, $CDCl_3$) δ 155.6, 144.7, 144.2, 135.0 (q, *J* = 35.0 Hz), 129.4, 128.8, 128.3, 126.5 (q, *J* = 3.0 Hz), 126.3, 123.3 (q, *J* = 271.0 Hz), 35.1, 31.2; **^{19}F NMR** (376 MHz, $CDCl_3$) δ -63.2 (s, 3F); **HRMS** (EI, m/z): calcd. for $C_{19}H_{19}F_3O_2S$ $[M]^+$: 368.1052, found: 368.1049.

(E)-4-(2-((4-(trifluoromethyl)phenyl)sulfonyl)vinyl)benzonitrile (**3fd**)

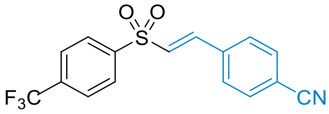

Compound **3fd** was prepared according to **General procedure P** (0.20 mmol scale, 14 h) to afford 49.9 mg (74%) as a white solid (and 86% yield for 15 mins). R_f = 0.29 (Petroleum ether /EtOAc = 5/1); **m.p.** = 193 – 196 °C; **^{1}H NMR** (400 MHz, DMSO-d^6) δ 8.17 (d, *J* = 8.2 Hz, 2H), 8.05 (d, *J* = 8.4 Hz, 2H), 7.96 (d, *J* = 8.3 Hz, 2H), 7.92 – 7.81 (m, 4H); **^{13}C NMR** (100 MHz, DMSO-d^6) δ 144.0, 141.4, 136.7, 133.4 (q, *J* = 32.0 Hz), 132.8, 130.5, 129.7, 128.4, 126.9 (q, *J* = 4.0 Hz), 123.3 (q, *J* = 271.0 Hz), 118.3, 113.2; **^{19}F NMR** (376 MHz, DMSO-d^6) δ -61.8 (s, 3F); **HRMS** (EI, m/z): calcd. for $C_{16}H_{10}F_3NO_2S$ $[M]^+$: 337.0379, found: 337.0378.

(E)-1-fluoro-4-(2-((4-(trifluoromethyl)phenyl)sulfonyl)vinyl)benzene (**3fe**)

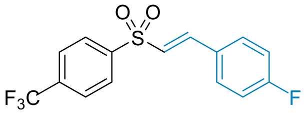

Compound **3fe** was prepared according to **General procedure P** (0.20 mmol scale, 5 mins) to afford 59.3 mg (89%) as a white solid (and 91% yield for 5 mins). R_f = 0.32 (Petroleum ether /EtOAc = 10/1); **m.p.** = 159 – 161 °C; **^{1}H NMR** (400 MHz, $CDCl_3$) δ 8.08 (d, *J* = 8.2 Hz, 2H), 7.80 (d, *J* = 8.2 Hz, 2H),

7.70 (d, J = 15.4 Hz, 1H), 7.51 – 7.48 (m, 2H), 7.08 (t, J = 8.0 Hz, 2H), 6.81 (d, J = 15.4 Hz, 1H); **^{13}C NMR** (100 MHz, $CDCl_3$) δ 164.7 (d, J = 252.0 Hz), 144.4, 142.8, 135.1 (q, J = 33.0 Hz), 131.0 (d, J = 9.0 Hz), 128.4 (d, J = 3.0 Hz), 128.3, 126.6 (q, J = 4.0 Hz), 126.1 (d, J = 2.0 Hz), 123.2 (q, J = 272.0 Hz), 116.5 (d, J = 22.0 Hz); **^{19}F NMR** (376 MHz, $CDCl_3$) δ -63.2 (s, 3F), -107.0 (s, 1F); **HRMS** (EI, m/z): calcd. for $C_{15}H_{10}F_4O_2S$ $[M]^+$: 330.0332, found: 330.0334.

(E)-1-chloro-4-(2-((4-(trifluoromethyl)phenyl)sulfonyl)vinyl)benzene (**3ff**)

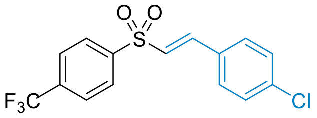

Compound **3ff** was prepared according to **General procedure N** (0.20 mmol scale, 14 h) to afford 52.9 mg (77%) as a white solid. R_f = 0.27 (Petroleum ether /EtOAc = 10/1); **m.p.** = 190 – 194 °C; **^{1}H NMR** (400 MHz, DMSO-d^6) δ 8.15 (d, J = 8.2 Hz, 2H), 8.04 (d, J = 8.2 Hz, 2H), 7.80 (d, J = 8.4 Hz, 2H), 7.72 (d, J = 15.9 Hz, 2H), 7.51 (d, J = 8.3 Hz, 2H); **^{13}C NMR** (100 MHz, DMSO-d^6) δ 144.5, 142.1, 136.0, 133.2 (q, J = 32.0 Hz), 131.2, 130.9, 129.1, 128.2, 127.8, 126.8 (q, J = 4.0 Hz), 123.3 (q, J = 272.0 Hz); **^{19}F NMR** (376 MHz, DMSO-d^6) δ -61.8 (s, 3F); **HRMS** (EI, m/z): calcd. for $C_{15}H_{10}ClF_3O_2S$ $[M]^+$: 346.0037, found: 346.0036.

(E)-1-bromo-4-(2-((4-(trifluoromethyl)phenyl)sulfonyl)vinyl)benzene (**3fg**)

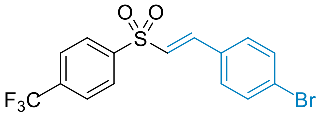

Compound **3fg** was prepared according to **General procedure P** (0.20 mmol scale, 14 h) to afford 47.6 mg (62%) as a white solid. R_f = 0.50 (Petroleum ether /EtOAc = 5/1); **m.p.** = 205 – 208 °C; **^{1}H NMR** (400 MHz, $CDCl_3$) δ 8.08 (d, J = 8.2 Hz, 2H), 7.82 (d, J = 8.2 Hz, 2H), 7.67 (d, J = 15.4 Hz, 1H), 7.54 (d, J = 8.3 Hz, 2H), 7.36 (d, J = 8.3 Hz, 2H), 6.85 (d, J = 15.4 Hz, 1H).; **^{13}C NMR** (100 MHz, $CDCl_3$) δ 144.3, 142.7, 135.4 (q, J = 35.0 Hz), 132.6, 131.1, 130.2, 128.5, 127.2, 126.7 (q, J = 3.0 Hz), 126.3, 123.3 (q, J = 272.0 Hz); **^{19}F NMR** (376 MHz, $CDCl_3$) δ -63.2 (s, 3F); **HRMS** (EI, m/z): calcd. for $C_{15}H_{10}BrF_3O_2S$ $[M]^+$: 389.9531, found: 389.9530.

(E)-1-methoxy-4-(2-((4-(trifluoromethyl)phenyl)sulfonyl)vinyl)benzene (**3fh**)

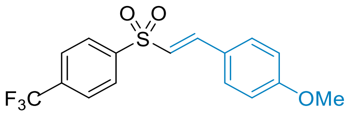

Compound **3fh** was prepared according to **General procedure P** (0.20 mmol scale, 14 h) to afford 47.7 mg (70%) as a white solid. R_f = 0.37 (*n*-hexane /CH_2Cl_2 = 1/2); **m.p.** = 178 – 182 °C; **^{1}H NMR** (400 MHz, DMSO-d^6) δ 8.14 (d, J = 8.2 Hz, 2H), 8.02 (d, J = 8.3 Hz, 2H), 7.74 – 7.67 (m, 3H), 7.49 (d, J = 15.3 Hz, 1H), 6.99 (d, J = 8.7 Hz, 2H), 3.79 (s, 3H); **^{13}C NMR** (100 MHz, DMSO-d^6) δ 161.9, 145.1, 143.4, 133.0 (q, J = 22.0 Hz), 131.1, 128.0, 126.7 (q, J = 4.0 Hz), 124.8, 124.0, 123.4 (q, J = 272.0 Hz), 114.7, 55.4; **^{19}F NMR** (376 MHz, DMSO-d^6) δ -61.8 (s, 3F); **HRMS** (EI, m/z): calcd. for $C_{15}H_{10}BrF_3O_2S$ $[M]^+$: 342.0532, found: 342.0533.

(E)-1-(trifluoromethyl)-4-(2-((4-(trifluoromethyl)phenyl)sulfonyl)vinyl)benzene (**3fi**)

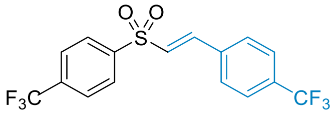

Compound **3ea** was prepared according to **General procedure P** (0.20 mmol scale, 14 h) to afford 58.6 mg (78%) as a white solid. R_f = 0.50 (Petroleum ether /EtOAc = 5/1); **m.p.** = 190 – 192 °C; **^{1}H NMR** (400 MHz, DMSO-*d*^{6}) δ 8.17 (d, *J* = 8.3 Hz, 2H), 8.06 (d, *J* = 8.4 Hz, 2H), 7.99 (d, *J* = 8.1 Hz, 2H), 7.92 – 7.86 (m, 2H), 7.80 (d, *J* = 8.3 Hz, 2H); **^{13}C NMR** (100 MHz, DMSO-*d*^{6}) δ 144.2, 141.6, 136.2, 133.3 (q, *J* = 32.0 Hz), 130.8 (q, *J* = 32.0 Hz), 130.0, 129.8, 128.4, 126.9 (q, *J* = 4.0 Hz), 125.8 (q, *J* = 3.0 Hz), 123.8 (q, *J* = 271.0 Hz), 123.3 (q, *J* = 271.0 Hz); **^{19}F NMR** (376 MHz, DMSO-*d*^{6}) δ -61.5 (s, 3F), -61.8 (s, 3F); **HRMS** (EI, m/z): calcd. for $C_{16}H_{10}F_6O_2S$ $[M]^+$: 380.0300, found: 380.0301.

Methyl *(E)*-4-(2-((4-(trifluoromethyl)phenyl)sulfonyl)vinyl)benzoate (**3fj**)

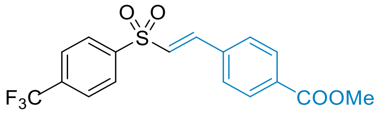

Compound **3fj** was prepared according to **General procedure P** (0.20 mmol scale, 14 h) to afford 57.3 mg (77%) as a white solid. R_f = 0.35 (Petroleum ether /EtOAc = 5/1); **m.p.** = 195 – 198 °C; **^{1}H NMR** (400 MHz, DMSO-*d*^{6}) δ 8.18 (d, *J* = 8.3 Hz, 2H), 8.04 (d, *J* = 8.4 Hz, 2H), 7.98 (d, *J* = 8.4 Hz, 2H), 7.90 (d, *J* = 8.4 Hz, 2H), 7.84 – 7.79 (m, 2H), 3.85 (s, 3H); **^{13}C NMR** (100 MHz, DMSO-*d*^{6}) δ 165.5, 144.2, 141.9, 136.6, 133.3 (q, *J* = 32.0 Hz), 131.6, 129.7, 129.6, 129.3, 128.4, 126.8 (q, *J* = 4.0 Hz), 123.3 (q, *J* = 272.0 Hz), 52.3; **^{19}F NMR** (376 MHz, DMSO-*d*^{6}) δ -61.8 (s, 3F); **HRMS** (EI, m/z): calcd. for $C_{17}H_{12}F_3O_4S$ $[M]^+$: 370.0481, found: 370.0480.

(E)-1-chloro-3-(2-((4-(trifluoromethyl)phenyl)sulfonyl)vinyl)benzene (**3fk**)

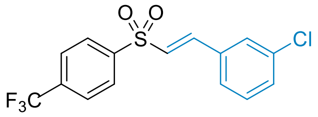

Compound **3fk** was prepared according to **General procedure P** (0.20 mmol scale, 14 h) to afford 57.7 mg (84%) as a white solid (and 74% yield for 10 mins). R_f = 0.51 (Petroleum ether /EtOAc = 5/1); **m.p.** = 138 – 143 °C; **^{1}H NMR** (400 MHz, DMSO-*d*^{6}) δ 8.16 (d, *J* = 8.0 Hz, 2H), 8.04 (d, *J* = 8.1 Hz, 2H), 7.91 (s, 1H), 7.83 – 7.72 (m, 3H), 7.51 – 7.43 (m, 2H); **^{13}C NMR** (100 MHz, DMSO-*d*^{6}) δ 144.3, 141.8, 134.4, 133.8, 133.3 (q, *J* = 32.0 Hz), 130.9, 130.7, 128.8, 128.4, 128.3, 128.0, 126.8 (q, *J* = 3.0 Hz), 123.3 (q, *J* = 271.0 Hz); **^{19}F NMR** (376 MHz, DMSO-*d*^{6}) δ -61.8 (s, 3F); **HRMS** (EI, m/z): calcd. for $C_{15}H_{10}ClF_3O_2S$ $[M]^+$: 346.0037, found: 346.0036.

(E)-1-chloro-2-(2-((4-(trifluoromethyl)phenyl)sulfonyl)vinyl)benzene (**3fl**)

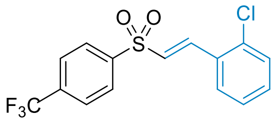

Compound **3fl** was prepared according to **General procedure P** (0.20 mmol scale, 14 h) to afford 60.9 mg (88%) as a white solid (and 86% yield for 10 mins). R_f = 0.51 (Petroleum ether /EtOAc = 5/1); **m.p.** = 164 – 166 °C; **^{1}H NMR** (400 MHz, DMSO-*d*^{6}) δ 8.18 (d, *J* = 8.2 Hz, 2H), 8.04 (d, *J* = 8.4 Hz, 2H), 7.98 (d, *J* = 15.4 Hz, 1H), 7.92 (dd, *J* = 7.8, 1.4 Hz, 1H), 7.83 (d, *J* = 15.4 Hz, 1H), 7.56 (dd, *J* = 8.0, 1.0 Hz, 1H), 7.48 (td, *J* = 7.7, 1.5 Hz, 1H), 7.42 – 7.38 (m, 1H); **^{13}C NMR** (100 MHz, DMSO-*d*^{6}) δ 144.0,

138.0, 134.3, 133.4 (q, J = 33.0 Hz), 132.9, 130.1, 130.0, 129.7, 129.0, 128.4, 127.8, 126.8(q, J = 4.0 Hz), 123.3 (q, J = 271.0 Hz);^{19}**F NMR** (376 MHz, DMSO-d^6) δ -61.9 (s, 3F); **HRMS** (EI, m/z): calcd. for $C_{15}H_{10}ClF_3O_2S$ $[M]^+$: 346.0037, found: 346.0036.

(E)-2-(2-((4-(trifluoromethyl)phenyl)sulfonyl)vinyl)pyridine (**3fm**)

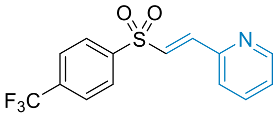

Compound **3fm** was prepared according to **General procedure P** (0.20 mmol scale, 14 h) to afford 56.2 mg (89%) as a white solid. R_f = 0.24 (Petroleum ether /EtOAc = 5/1); **m.p.** = 110 – 113 °C; ^{1}**H NMR** (400 MHz, DMSO-d^6) δ 8.64 (dd, J = 4.7, 0.7 Hz, 1H), 8.20 (d, J = 8.2 Hz, 2H), 8.03 (d, J = 8.4 Hz, 2H), 7.90 (td, J = 7.7, 1.7 Hz, 1H), 7.82 (d, J = 7.7 Hz, 1H), 7.78 (s, 2H), 7.45 (ddd, J = 7.5, 4.7, 1.1 Hz, 1H); ^{13}**C NMR** (100 MHz, DMSO-d^6) δ 150.5, 150.1, 144.0, 142.6, 137.4, 133.4 (q, J = 32.0 Hz), 130.5, 128.5, 126.8 (q, J = 4.0 Hz), 125.8, 125.6, 123.3 (q, J = 271.0 Hz); ^{19}**F NMR** (376 MHz, DMSO-d^6) δ -61.9 (s, 3F); **HRMS** (EI, m/z): calcd. for $C_{14}H_{10}F_3NO_2S$ $[M]^+$: 313.0379, found: 313.0380.

(2-((4-(trifluoromethyl)phenyl)sulfonyl)ethene-1,1-diyl)dibenzene (**3fn**)

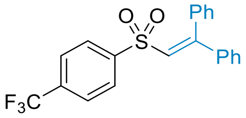

Compound **3fn** was prepared according to **General procedure P** (0.20 mmol scale, 14 h) to afford 58.4 mg (74%) as a white solid. R_f = 0.32 (Petroleum ether /EtOAc = 5/1); **m.p.** = 133 – 136 °C; ^{1}**H NMR** (400 MHz, DMSO-d^6) δ 7.87 – 7.81 (m, 4H), 7.46 – 7.41 (m, 2H), 7.40 – 7.34 (m, 3H), 7.33 – 7.27 (m, 4H), 7.00 – 6.98 (m, 2H); ^{13}**C NMR** (100 MHz, DMSO-d^6) δ 154.9, 145.1, 138.1, 135.2, 132.7 (q, J = 32.0 Hz), 130.5, 129.1, 128.7, 128.6, 128.2, 128.1, 127.8, 127.7, 126.2 (q, J = 4.0 Hz), 123.3 (q, J = 271.0 Hz); ^{19}**F NMR** (376 MHz, DMSO-d^6) δ -61.8 (s, 3F); **HRMS** (EI, m/z): calcd. for $C_{21}H_{15}F_3O_2S$ $[M]^+$: 388.0739, found: 388.0738. The spectroscopic data of **3fs** matched the reported values.[35]

1-((2-phenylallyl)sulfonyl)-4-(trifluoromethyl)benzene (**3fo**)

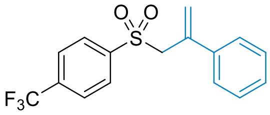

Compound **3fo** was prepared according to **General procedure P** (0.20 mmol scale, 14 h) to afford 24.6 mg (38%) as a white solid. R_f = 0.50 (Petroleum ether /EtOAc = 10/1); **m.p.** = 121 – 124 °C; ^{1}**H NMR** (400 MHz, $CDCl_3$) δ 7.86 (d, J = 8.2 Hz, 2H), 7.63 (d, J = 8.3 Hz, 2H), 7.21 – 7.15 (m, 5H), 5.61 (s, 1H), 5.30 (s, 1H), 4.32 (s, 2H); ^{13}**C NMR** (100 MHz, $CDCl_3$) δ 142.0, 138.4, 136.4, 135.4 (q, J = 33.0 Hz), 129.4, 128.6, 128.4, 126.3, 126.0 (q, J = 4.0 Hz), 123.2 (q, J = 272.0 Hz), 122.5, 62.3; ^{19}**F NMR** (376 MHz, $CDCl_3$) δ -63.3 (s, 3F); **HRMS** (EI, m/z): calcd. for $C_{16}H_{13}F_3O_2S$ $[M]^+$: 326.0583, found: 326.0582. The spectroscopic data of **3fo** matched the reported values.[36]

(*E*)-1-((2-phenylprop-1-en-1-yl)sulfonyl)-4-(trifluoromethyl)benzene (**3fo'**)

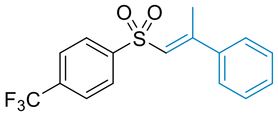

Compound **3fo'** was prepared according to **General procedure P** (0.20 mmol scale, 14 h) to afford 5.3 mg (8%) as colorless oil. R_f = 0.25 (Petroleum ether /EtOAc = 20/1); **^{1}H NMR** (400 MHz, $CDCl_3$) δ 8.11 (d, J = 8.1 Hz, 2H), 7.83 (d, J = 8.1 Hz, 2H), 7.41 – 7.37 (m, 5H), 6.61 (s, 5H), 2.55 (s, 3H); **^{13}C NMR** (100 MHz, $CDCl_3$) δ 155.3, 145.8, 139.9, 135.0 (q, J = 33.0 Hz), 130.4, 128.9, 127.9, 126.5 (q, J = 4.0 Hz), 126.4, 123.3 (q, J = 271.0 Hz), 17.4; **^{19}F NMR** (376 MHz, $CDCl_3$) δ -63.1 (s, 3F); **HRMS** (EI, m/z): calcd. for $C_{16}H_{13}F_3O_2S$ $[M]^+$: 326.0583, found: 326.0584.

3-tosyl-1,2-dihydronaphthalene (**3ap**)

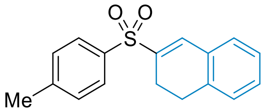

Compound **3ar** was prepared according to **General procedure P** (0.20 mmol scale, 14 h) to afford 41.2 mg (72%) as a white solid. R_f = 0.50 (Petroleum ether /EtOAc = 5/1); **m.p.** = 123 – 126 °C; **^{1}H NMR** (400 MHz, $CDCl_3$) δ 7.80 (d, J = 8.3 Hz, 2H), 7.56 (s, 1H), 7.32 (d, J = 8.1 Hz, 2H), 7.29 – 7.20 (m, 3H), 7.12 (d, J = 6.9 Hz, 1H), 2.86 (t, J = 8.3 Hz, 2H), 2.49 (td, J = 8.3, 1.1 Hz, 2H), 2.42 (s, 3H); **^{13}C NMR** (100 MHz, $CDCl_3$) δ 144.4, 138.6, 136.8, 135.6, 134.8, 131.1, 130.5, 130.0, 129.1, 128.1, 127.9, 127.2, 27.7, 21.8, 21.7; **HRMS** (EI, m/z): calcd. for $C_{17}H_{16}O_2S$ $[M]^+$: 284.0866, found: 284.0867. The spectroscopic data of **3ar** matched the reported values.[30]

(E)-1-(2-tosylvinyl)naphthalene (**3aq**)

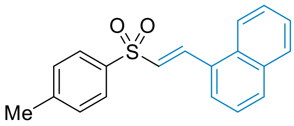

Compound **3aq** was prepared according to **General procedure P** (0.20 mmol scale, 14 h) to afford 34.0 mg (55%) as a white solid. R_f = 0.27 (Petroleum ether /EtOAc = 10/1); **m.p.** = 86 – 89 °C; **^{1}H NMR** (400 MHz, $CDCl_3$) δ 8.50 (d, J = 15.2 Hz, 1H), 8.17 (d, J = 8.3 Hz, 1H), 7.92 – 7.87 (m, 4H), 7.67 – 7.53 (m, 3H), 7.45 (t, J = 7.7 Hz, 1H), 7.36 (d, J = 7.8 Hz, 2H), 6.95 (d, J = 15.2 Hz, 1H), 2.44 (s, 3H); **^{13}C NMR** (100 MHz, $CDCl_3$) δ 144.6, 139.1, 137.8, 133.8, 131.5, 131.4, 130.2, 130.1, 129.7, 129.0, 127.9, 127.4, 126.6, 125.8, 125.4, 123.2, 21.8; **HRMS** (EI, m/z): calcd. for $C_{19}H_{16}O_2S$ $[M]^+$: 308.0866, found: 308.0864. The spectroscopic data of **3aq** matched the reported values.[30]

(1R,2S,5R)-2-isopropyl-5-methylcyclohexyl4-((E)-2-((4-trifluoromethyl)phenyl)sulfonyl)benzoate (**3fr**)

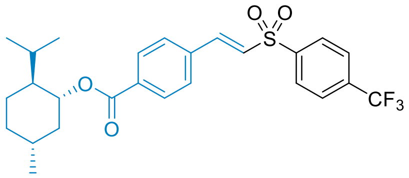

Compound **3fr** was prepared according to **General procedure P** (0.20 mmol scale, 14 h) to afford 89.2 mg (90%) as a white solid. R_f = 0.32 (Petroleum ether /EtOAc = 10/1); **m.p.** = 122 – 125 °C; **^{1}H NMR** (400 MHz, $CDCl_3$) δ 8.09 (d, J = 8.1 Hz, 2H), 8.05 (d, J = 8.1 Hz, 2H), 7.81 (d, J = 8.1 Hz, 2H), 7.76 (d, J = 15.4 Hz, 1H), 7.56 (d, J = 8.1 Hz, 2H), 6.97 (d, J = 15.4 Hz, 1H), 4.92 (td, J = 10.8, 4.0 Hz, 1H), 2.09 (d, J = 12.0 Hz, 1H), 1.92 – 1.87 (m, 1H), 1.71 (d, J = 11.4 Hz, 2H), 1.54 (t, J = 10.2 Hz, 2H), 1.16 –

1.04 (m, 2H), 0.90 (t, J = 5.7 Hz, 7H), 0.76 (d, J = 6.9 Hz, 3H).; **^{13}C NMR** (100 MHz, $CDCl_3$) δ 165.1, 144.1, 142.7, 136.0, 135.3 (q, J = 33.0 Hz), 133.4, 130.3, 128.7, 128.5, 126.7 (q, J = 4.0 Hz), 123.2 (q, J = 271.0 Hz), 75.5, 47.3, 41.0, 34.3, 31.5, 26.6, 23.7, 22.1, 20.8, 16.6; **^{19}F NMR** (376 MHz, $CDCl_3$) δ -63.2 (s, 3F); **HRMS** (ESI, m/z): calcd. for $C_{26}H_{29}F_3NaO_4S$ $[M+Na]^+$: 517.1631, found: 517.1634.

2-isopropyl-5-methylphenyl (E)-4-(2-((4-(trifluoromethyl)phenyl)sulfonyl)vinyl)benzoate (**3fs**)

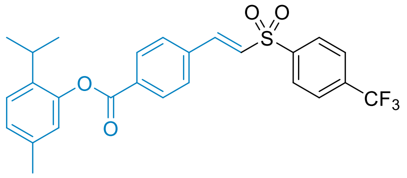

Compound **3fs** was prepared according to **General procedure P** (0.20 mmol scale, 14 h) to afford 45.3 mg (46%) as a white solid. R_f = 0.23 (Petroleum ether /EtOAc = 10/1); **m.p.** = 102 – 105 °C; **^{1}H NMR** (400 MHz, $CDCl_3$) δ 8.25 (d, J = 8.1 Hz, 2H), 8.13 (d, J = 8.1 Hz, 2H), 7.86 – 7.80 (m, 3H), 7.65 (d, J = 8.1 Hz, 2H), 7.25 (d, J = 7.2 Hz, 1H), 7.08 (d, J = 7.8 Hz, 1H), 7.03 (d, J = 15.4 Hz, 1H), 6.94 (s, 1H), 3.05 – 2.99 (m, 1H), 2.34 (s, 3H), 1.22 (s, 3H), 1.20 (s, 3H); **^{13}C NMR** (100 MHz, $CDCl_3$) δ 164.5, 144.0, 142.4, 137.1, 136.9, 136.8, 135.4 (q, J = 32.0 Hz), 132.1, 130.9, 129.2, 128.9, 128.5, 128.3, 127.5, 126.7 (q, J = 4.0 Hz), 123.2 (q, J = 271.0 Hz), 122.8, 27.4, 23.1, 20.9; **^{19}F NMR** (376 MHz, $CDCl_3$) δ -63.2 (s, 3F);**HRMS** (EI, m/z): calcd. for $C_{26}H_{23}F_3O_4S$ $[M]^+$: 488.1264, found: 488.1264.

((3aR,5R,5aS,8aS,8bR)-7,7-dimethyltetrahydro-5H-bis([1,3]dioxolo)[4,5-b:4',5'-d]pyran-5-yl)methyl 4-((E)-2-((4-(trifluoromethyl)phenyl)sulfonyl)vinyl)benzoate (**3ft**)

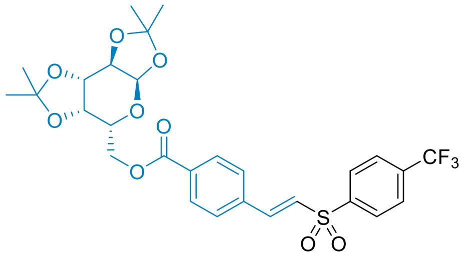

Compound **3ft** was prepared according to **General procedure P** (0.20 mmol scale, 14 h) to afford 127.2.0 mg (99%) as a white solid. R_f = 0.23 (Petroleum ether /EtOAc = 5/1); **m.p.** = 139 – 143 °C; **^{1}H NMR** (400 MHz, $CDCl_3$) δ 8.07 (d, J = 8.1 Hz, 2H), 8.03 (d, J = 8.1 Hz, 2H), 7.80 (d, J = 8.1 Hz, 2H), 7.73 (d, J = 15.4 Hz, 1H), 7.53 (d, J = 8.1 Hz, 2H), 6.95 (d, J = 15.4 Hz, 1H), 5.54 (d, J = 4.8 Hz, 1H), 4.63 (d, J = 7.8 Hz, 1H), 4.51 (dd, J = 11.6, 4.3 Hz, 1H), 4.41 (dd, J = 11.4, 7.8 Hz, 1H), 4.33 – 4.28 (m, 2H), 4.16 (d, J = 5.1 Hz, 1H), 1.48 (s, 3H), 1.44 (s, 3H), 1.32 (s, 3H), 1.30 (s, 3H); **^{13}C NMR** (100 MHz, $CDCl_3$) δ; 165.5, 144.0, 142.5, 136.2, 135.3 (q, J = 33.0 Hz), 132.6, 130.5, 128.8, 128.7, 128.5, 126.7 (q, J = 4.0 Hz), 123.2 (q, J = 271.0 Hz), 109.8, 108.9, 96.4, 71.2, 70.8, 70.6, 66.2, 64.5, 26.1, 26.0, 25.0, 24.5; **^{19}F NMR** (376 MHz, $CDCl_3$) δ -63.2 (s, 3F); **HRMS** (ESI, m/z): calcd. for $C_{28}H_{29}F_3NaO_9S$ $[M+Na]^+$: 621.1377, found: 621.1378.

Methyl 4-(*(E)*-2-(((E)-styryl)sulfonyl)vinyl)benzoate (**5aj**)

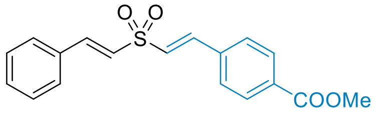

Compound **5aj** was prepared according to **General procedure P** (0.20 mmol scale, 14 h) to afford 53.0 mg (80%) as a white solid. R_f = 0.20 (Petroleum ether /EtOAc = 5/1); **m.p.** = 202 – 204 °C; **^{1}H NMR** (400 MHz, $CDCl_3$) δ 8.05 (d, *J* = 7.8 Hz, 2H), 7.65 (d, *J* = 15.4 Hz, 2H), 7.56 (d, *J* = 8.0 Hz, 2H), 7.51 (d, *J* = 6.4 Hz, 2H), 7.40 (d, *J* = 6.3 Hz, 3H), 6.96 (d, *J* = 15.5 Hz, 1H), 6.87 (d, *J* = 15.4 Hz, 1H), 3.91 (s, 3H); **^{13}C NMR** (100 MHz, $CDCl_3$) δ 166.2, 144.2, 141.9, 136.7, 132.5, 132.3, 131.5, 130.3, 129.2, 129.0, 128.7, 128.5, 126.0, 52.5; **HRMS** (EI, m/z): calcd. for $C_{18}H_{16}O_4S$ $[M]^+$: 328.0764, found: 328.0766.

Methyl 4-(*(E)*-2-((*(E)*-4-fluorostyryl)sulfonyl)vinyl)benzoate (**5bj**)

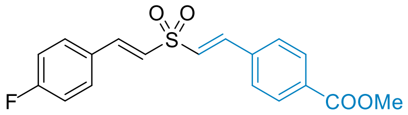

Compound **5bj** was prepared according to **General procedure P** (0.20 mmol scale, 14 h) to afford 29.6 mg (44%) as a white solid. R_f = 0.24 (DCM); **m.p.** = 168 – 172 °C; **^{1}H NMR** (400 MHz, $CDCl_3$) δ 8.06 (d, *J* = 8.2 Hz, 2H), 7.67 – 7.56 (m, 4H), 7.53 – 7.50 (m, 2H), 7.10 (t, *J* = 8.4 Hz, 2H), 6.95 (d, *J* = 15.5 Hz, 1H), 6.79 (d, *J* = 15.4 Hz, 1H), 3.92 (s, 3H); **^{13}C NMR** (100 MHz, $CDCl_3$) δ 166.3, 164.6 (d, *J* = 252.0 Hz), 143.0, 142.1, 136.7, 132.4, 130.8 (d, *J* = 9.0 Hz), 130.4, 128.9, 128.8 (d, *J* = 3.0 Hz), 128.5, 125.8, 116.6 (d, *J* = 22.0 Hz), 52.5; **^{19}F NMR** (376 MHz, $CDCl_3$) δ -107.3 (s, 1F); **HRMS** (EI, m/z): calcd. for $C_{18}H_{14}FO_4S$ $[M]^+$: 346.0670, found: 346.0671.

Methyl 4-(*(E)*-2-((*(E)*-4-chlorostyryl)sulfonyl)vinyl)benzoate (**5cj**)

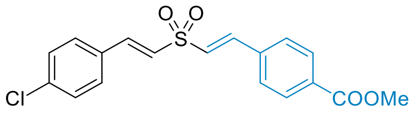

Compound **5cj** was prepared according to **General procedure P** (0.20 mmol scale, 14 h) to afford 46.4 mg (63%) as a white solid. R_f = 0.21 (Petroleum ether /EtOAc = 5/1); **m.p.** = 190 – 193 °C; **^{1}H NMR** (400 MHz, $CDCl_3$) δ 8.06 (d, *J* = 8.3 Hz, 2H), 7.65 (d, *J* = 15.5 Hz, 1H), 7.62 – 7.55 (m, 3H), 7.44 (d, *J* = 8.6 Hz, 2H), 7.38 (d, *J* = 8.5 Hz, 2H), 6.95 (d, *J* = 15.5 Hz, 1H), 6.84 (d, *J* = 15.4 Hz, 1H), 3.92 (s, 3H); **^{13}C NMR** (100 MHz, $CDCl_3$) δ 166.2, 142.8, 142.3, 137.6, 136.6, 132.4, 131.0, 130.4, 129.9, 129.6, 128.7, 128.6, 126.7, 52.5; **HRMS** (EI, m/z): calcd. for $C_{18}H_{15}ClO_4S$ $[M]^+$: 362.0374, found: 362.0375.

Methyl 4-(*(E)*-2-((*(E)*-4-bromostyryl)sulfonyl)vinyl)benzoate (**5dj**)

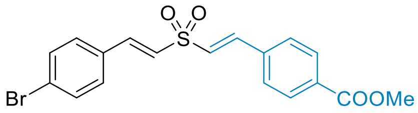

Compound **5dj** was prepared according to **General procedure P** (0.20 mmol scale, 14 h) to afford 46.4 mg (56%) as a white solid. R_f = 0.41 (DCM); **m.p.** = 151 – 154 °C; **^{1}H NMR** (400 MHz, $CDCl_3$) δ 8.06 (d, *J* = 8.3 Hz, 2H), 7.65 (d, *J* = 15.5 Hz, 1H), 7.61 – 7.53 (m, 5H), 7.37 (d, *J* = 8.4 Hz, 2H), 6.94 (d, *J* = 15.5 Hz, 1H), 6.86 (d, *J* = 15.4 Hz, 1H), 3.93 (s, 3H); **^{13}C NMR** (100 MHz, $CDCl_3$) δ 166.2, 142.8, 142.3, 136.7, 132.6, 132.5, 131.4, 130.4, 130.1, 128.7, 128.6, 126.8, 126.0, 52.5; **HRMS** (EI, m/z): calcd. for $C_{18}H_{15}BrO_4S$ $[M]^+$: 405.9869, found: 405.9870.

Methyl 4-(*(E)*-2-((*(E)*-4-methoxystyryl)sulfonyl)vinyl)benzoate (**5ej**)

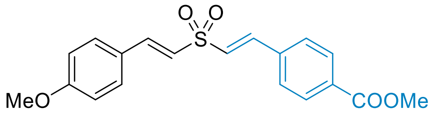

Compound **5ej** was prepared according to **General procedure P** (0.20 mmol scale, 14 h) to afford 62.0 mg (86%) as a white solid. R_f = 0.36 (Petroleum ether /EtOAc = 2/1); **m.p.** = 152 – 155 ºC; **^{1}H NMR** (400 MHz, $CDCl_3$) δ 8.06 (d, *J* = 8.3 Hz, 2H), 7.65 – 7.55 (m, 4H), 7.46 (d, *J* = 8.7 Hz, 2H), 6.96 – 6.91 (m, 3H), 6.70 (d, *J* = 15.4 Hz, 1H), 3.93 (s, 3H), 3.84 (s, 3H); **^{13}C NMR** (100 MHz, $CDCl_3$) δ 166.3, 162.4, 144.1, 141.3, 136.9, 132.3, 130.6, 130.4, 129.4, 128.5, 125.2, 123.1, 114.7, 55.6, 52.5; **HRMS** (EI, m/z): calcd. for $C_{19}H_{17}O_5S$ $[M]^+$: 358.0869, found: 358.0871.

Methyl 4-(*(E)*-2-((*(E)*-4-(methylthio)styryl)sulfonyl)vinyl)benzoate (**5fj**)

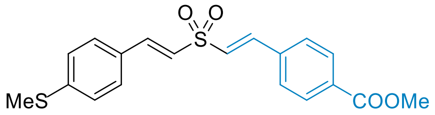

Compound **5fj** was prepared according to **General procedure P** (0.20 mmol scale, 14 h) to afford 63.0 mg (84%) as a white solid. R_f = 0.34 (Petroleum ether /EtOAc = 3/1); **m.p.** = 184 – 187 ºC; **^{1}H NMR** (400 MHz, $CDCl_3$) δ 8.05 (d, *J* = 8.3 Hz, 2H), 7.65 – 7.55 (m, 4H), 7.41 (d, *J* = 8.4 Hz, 2H), 7.22 (d, *J* = 8.4 Hz, 2H), 6.95 (d, *J* = 15.5 Hz, 1H), 6.79 (d, *J* = 15.4 Hz, 1H), 3.92 (s, 3H), 2.49 (s, 3H); **^{13}C NMR** (100 MHz, $CDCl_3$) δ 166.3, 143.9, 143.7, 141.7, 136.8, 132.3, 130.4, 129.1, 129.1, 128.8, 128.5, 126.0, 124.6, 52.5, 15.0; **HRMS** (EI, m/z): calcd. for $C_{19}H_{18}O_4S_2$ $[M]^+$: 374.0641, found: 374.0642.

Methyl 4-(*(E)*-2-((*(E)*-2-([1,1'-biphenyl]-4-yl)vinyl)sulfonyl)vinyl)benzoate (**5gj**)

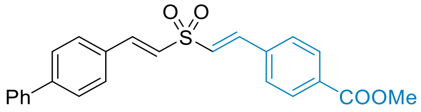

Compound **5gj** was prepared according to **General procedure P** (0.20 mmol scale, 14 h) to afford 36.2 mg (41%) as a white solid. R_f = 0.24 (DCM); **m.p.** = 214 – 217 ºC; **^{1}H NMR** (400 MHz, DMSO-d^6) δ 7.99 (d, *J* = 8.3 Hz, 2H), 7.92 (d, *J* = 8.3 Hz, 2H), 7.87 (d, *J* = 8.2 Hz, 2H), 7.77 – 7.72 (m, 4H), 7.65 (t, 3H), 7.53 (d, *J* = 15.5 Hz, 1H), 7.48 (t, *J* = 7.5 Hz, 2H), 7.39 (t, *J* = 7.3 Hz, 1H), 3.86 (s, 3H); **^{13}C NMR** (100 MHz, DMSO-d^6) δ 165.6, 142.6, 142.1, 140.4, 138.9, 137.1, 131.7, 131.3, 130.4, 129.6, 129.1, 129.0, 128.1, 127.1, 126.7, 52.3; **HRMS** (EI, m/z): calcd. for $C_{24}H_{20}O_4S$ $[M]^+$: 404.1077, found: 404.1078.

Methyl 4-(*(E)*-2-((*(E)*-4-(trifluoromethoxy)styryl)sulfonyl)vinyl)benzoate (**5hj**)

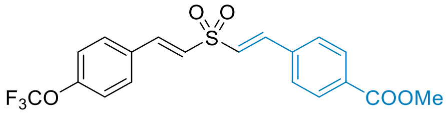

Compound **5hj** was prepared according to **General procedure P** (0.20 mmol scale, 14 h) to afford 54.1 mg (65%) as a white solid. R_f = 0.45 (Petroleum ether /EtOAc = 3/1); **m.p.** = 198 – 200 ºC; **^{1}H NMR** (400 MHz, $CDCl_3$) δ 8.06 (d, *J* = 8.2 Hz, 2H), 7.67 (d, *J* = 10.6 Hz, 1H), 7.63 (d, *J* = 10.6 Hz, 1H), 7.58 – 7.54 (m, 4H), 7.25 (d, *J* = 8.0 Hz, 2H), 6.95 (d, *J* = 15.5 Hz, 1H), 6.86 (d, *J* = 15.4 Hz, 1H), 3.92 (s, 3H); **^{13}C NMR** (100 MHz, $CDCl_3$) δ 166.2, 151.3, 142.4, 136.6, 132.5, 131.0, 130.4, 130.3, 128.7, 128.6, 127.1, 121.4, 120.4 (q, *J* = 257.0 Hz), 52.5; **^{19}F NMR** (376 MHz, $CDCl_3$) δ -57.7 (s, 3F); **HRMS** (EI, m/z): calcd. for $C_{19}H_{15}F_3O_5S$ $[M]^+$: 412.0587, found: 412.0586.

Methyl 4-((E)-2-(((E)-4-(trifluoromethyl)styryl)sulfonyl)vinyl)benzoate (**5ij**)

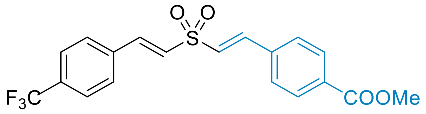

Compound **5ij** was prepared according to **General procedure P** (0.20 mmol scale, 14 h) to afford 35.4 mg (44%) as a white solid. R_f = 0.31 (DCM); **m.p.** = 209 – 212 ºC; **^{1}H NMR** (400 MHz, $CDCl_3$) δ 8.07 (d, J = 8.3 Hz, 2H), 7.70 – 7.66 (m, 4H), 7.63 (d, J = 8.4 Hz, 2H), 7.58 (d, J = 8.3 Hz, 2H), 6.96 (dd, J = 15.5, 1.7 Hz, 2H), 3.93 (s, 3H); **^{13}C NMR** (100 MHz, $CDCl_3$) δ 166.2, 142.9, 142.2, 136.5, 135.9, 132.9 (q, J = 33.0 Hz), 132.6, 130.4, 128.9, 128.9, 128.6, 128.4, 126.3 (q, J = 4.0 Hz), 123.7 (q, J = 271.0 Hz), 52.5; **^{19}F NMR** (376 MHz, $CDCl_3$) δ -63.0 (s, 3F); **HRMS** (EI, m/z): calcd. for $C_{19}H_{14}F_3O_4S$ $[M]^+$: 396.0638, found: 396.0639.

Ethyl 4-((E)-2-(((E)-4-(methoxycarbonyl)styryl)sulfonyl)vinyl)benzoate (**5jj**)

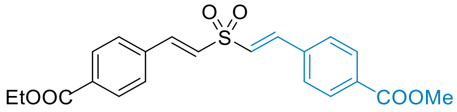

Compound **5jj** was prepared according to **General procedure P** (0.20 mmol scale, 14 h) to afford 59.1 mg (75%) as a white solid. R_f = 0.52 (DCM /MeOH = 100/1); **m.p.** = 199 – 201 ºC; **^{1}H NMR** (400 MHz, $CDCl_3$) δ 8.07 – 8.05 (m, 4H), 7.67 (d, J = 15.5 Hz, 2H), 7.57 (d, J = 7.4 Hz, 4H), 6.96 (d, J = 15.5 Hz, 2H), 4.38 (q, J = 7.1 Hz, 2H), 3.92 (s, 3H), 1.39 (t, J = 7.1 Hz, 3H); **^{13}C NMR** (100 MHz, $CDCl_3$) δ 166.2, 165.7, 142.7, 142.6, 136.6, 136.5, 132.9, 132.5, 130.4, 130.4, 128.6, 128.6, 128.6, 128.4, 61.5, 52.5, 14.4; **HRMS** (EI, m/z): calcd. for $C_{21}H_{20}O_6S$ $[M]^+$: 400.0981, found: 400.0982.

Methyl 4-((E)-2-(((E)-3-methylstyryl)sulfonyl)vinyl)benzoate (**5kj**)

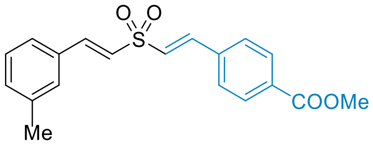

Compound **5kj** was prepared according to **General procedure P** (0.20 mmol scale, 14 h) to afford 46.3 mg (67%) as a white solid. R_f = 0.37 (Petroleum ether /EtOAc = 3/1); **m.p.** = 126 – 129 ºC; **^{1}H NMR** (400 MHz, $CDCl_3$) δ 8.04 (d, J = 7.7 Hz, 2H), 7.62 (dd, J = 15.4, 8.7 Hz, 2H), 7.55 (d, J = 7.7 Hz, 2H), 7.31 – 7.26 (m, 3H), 7.23 (d, J = 6.5 Hz, 1H), 6.97 (d, J = 15.5 Hz, 1H), 6.86 (d, J = 15.4 Hz, 1H), 3.91 (s, 3H), 2.34 (s, 3H); **^{13}C NMR** (100 MHz, $CDCl_3$) δ 166.2, 144.4, 141.7, 138.9, 136.7, 132.4, 132.3 132.2, 130.3, 129.3, 129.1, 128.5, 125.9, 125.7, 52.4, 21.3; **HRMS** (EI, m/z): calcd. for $C_{19}H_{18}O_4S$ $[M]^+$: 342.0920, found: 342.0922.

Methyl 4-((E)-2-(((E)-3-bromostyryl)sulfonyl)vinyl)benzoate (**5lj**)

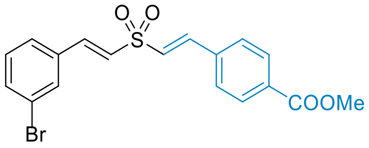

Compound **5lj** was prepared according to **General procedure P** (0.20 mmol scale, 14 h) to afford 35.6 mg (44%) as a white solid. R_f = 0.33 (Petroleum ether /EtOAc = 3/1); **m.p.** = 164 – 167 ºC; **^{1}H NMR** (400 MHz, $CDCl_3$) δ 8.06 (d, J = 8.3 Hz, 2H), 7.68 – 7.64 (m, 2H), 7.59 – 7.54 (m, 4H), 7.43 (d, J = 7.8

Hz, 1H), 7.28 (t, *J* = 12.6, 4.7 Hz, 1H), 6.94 (d, *J* = 15.5 Hz, 1H), 6.88 (d, *J* = 15.4 Hz, 1H), 3.93 (s, 3H); **^{13}C NMR** (100 MHz, $CDCl_3$) δ 166.2, 142.5, 142.4, 136.6, 134.5, 134.3, 132.5, 131.3, 130.8, 130.4, 128.6, 128.6, 127.7, 127.4, 123.4, 52.5; **HRMS** (EI, m/z): calcd. for $C_{18}H_{15}BrO_4S$ $[M]^+$: 405.9869, found: 405.9870.

Methyl 4-(*(E)*-2-((*(E)*-2-chlorostyryl)sulfonyl)vinyl)benzoate (**5mj**)

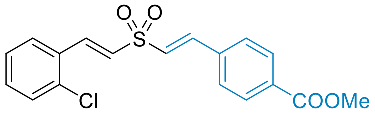

Compound **5mj** was prepared according to **General procedure P** (0.20 mmol scale, 14 h) to afford 33.9 mg (47%) as a white solid. R_f = 0.26 (Petroleum ether /EtOAc = 3/1); **m.p.** = 167 – 171 °C; **^{1}H NMR** (400 MHz, $CDCl_3$) δ 8.08 – 8.04 (m, 3H), 7.67 (d, *J* = 15.5 Hz, 1H), 7.58 (d, *J* = 8.3 Hz, 3H), 7.44 (dd, *J* = 7.9, 0.8 Hz, 1H), 7.36 (td, *J* = 7.7, 1.4 Hz, 1H), 7.31 – 7.27 (m, 1H), 6.97 (d, *J* = 15.5 Hz, 1H), 6.92 (d, *J* = 15.5 Hz, 1H), 3.92 (s, 3H); **^{13}C NMR** (100 MHz, $CDCl_3$) δ 166.2, 142.5, 140.1, 136.7, 135.5, 132.5, 132.3, 130.8, 130.6, 130.4, 128.8, 128.7, 128.6, 128.5, 127.4, 52.5; **HRMS** (EI, m/z): calcd. for $C_{18}H_{14}ClO_4S$ $[M]^+$: 362.0374, found: 362.0375.

Methyl 4-(*(E)*-2-((*(E)*-3,5-dimethylstyryl)sulfonyl)vinyl)benzoate (**5nj**)

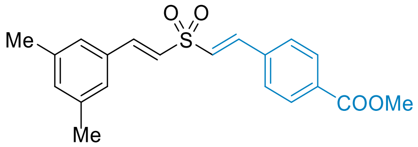

Compound **5nj** was prepared according to **General procedure P** (0.20 mmol scale, 14 h) to afford 46.5 mg (66%) as a white solid. R_f = 0.42 (Petroleum ether /EtOAc = 3/1); **m.p.** = 167 – 171 °C; **^{1}H NMR** (400 MHz, $CDCl_3$) δ 8.05 (d, *J* = 8.3 Hz, 2H), 7.65 – 7.55 (m, 4H), 7.12 (s, 2H), 7.06 (s, 1H), 6.95 (d, *J* = 15.5 Hz, 1H), 6.83 (d, *J* = 15.4 Hz, 1H), 3.92 (s, 3H), 2.31 (s, 6H); **^{13}C NMR** (100 MHz, $CDCl_3$) δ 166.2, 144.6, 141.6, 138.8, 136.8, 133.3, 132.4, 132.3, 130.3, 129.2, 128.5, 126.6, 125.5, 52.5, 21.2; **HRMS** (EI, m/z): calcd. for $C_{20}H_{20}O_4S$ $[M]^+$: 356.1077, found: 356.1079.

Methyl 4-(*(E)*-2-((*(E)*-2-(naphthalen-2-yl)vinyl)sulfonyl)vinyl)benzoate (**5oj**)

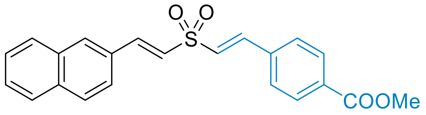

Compound **5oj** was prepared according to **General procedure P** (0.20 mmol scale, 14 h) to afford 36.8 mg (49%) as a light yellow solid. R_f = 0.24 (DCM); **m.p.** = 176 – 178 °C; **^{1}H NMR** (400 MHz, $CDCl_3$) δ 8.05 (d, *J* = 8.3 Hz, 2H), 7.94 (s, 1H), 7.86 – 7.79 (m, 4H), 7.68 (d, *J* = 15.5 Hz, 1H), 7.60 – 7.52 (m, 5H), 7.02 – 6.96 (m, 2H), 3.92 (s, 3H); **^{13}C NMR** (100 MHz, $CDCl_3$) δ 166.2, 144.3, 141.9, 136.8, 134.7, 133.2, 132.3, 131.2, 130.3, 129.9, 129.2, 129.1, 128.9, 128.5, 128.0, 127.9, 127.1, 126.0, 123.5, 52.5; **HRMS** (EI, m/z): calcd. for $C_{22}H_{17}O_4S$ $[M]^+$: 378.0920, found: 378.0923.

(*(E)*-1-(((E)-styryl)sulfonyl)ethene-1,2-diyl)dibenzene (**5pa**)

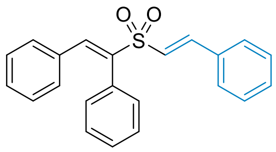

Compound **5pa** was prepared according to **General procedure P** (0.10 mmol scale, 14 h) to afford 11.6 mg (34%) as a white solid. R_f = 0.26 (Petroleum ether /EtOAc = 10/1); **m.p.** = 152 – 156 °C; **^{1}H NMR** (400 MHz, $CDCl_3$) δ 7.87 (s, 1H), 7.42 – 7.38 (m, 10H), 7.31 (d, *J* = 15.5 Hz, 1H), 7.25 (d, *J* = 6.6 Hz, 1H), 7.18 (t, *J* = 7.6 Hz, 2H), 7.09 (d, *J* = 7.6 Hz, 2H), 6.70 (d, *J* = 15.5 Hz, 1H); **^{13}C NMR** (100 MHz, $CDCl_3$) δ 144.8, 140.8, 138.3, 133.0, 132.7, 131.6, 131.2, 130.9, 130.7, 130.2, 129.5, 129.3, 129.2, 128.6, 128.5, 124.7; **HRMS** (EI, m/z): calcd. for $C_{22}H_{18}O_2S$ $[M]^+$: 346.1022, found: 346.1023.

(E)-(2-(styrylsulfonyl)ethene-1,1-diyl)dibenzene (**5qa**)

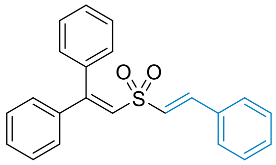

Compound **5qa** was prepared according to **General procedure P** (0.20 mmol scale, 14 h) to afford 56.5 mg (82%) as a white solid. R_f = 0.20 (Petroleum ether /EtOAc = 10/1); **m.p.** = 161 – 165 °C; **^{1}H NMR** (400 MHz, $CDCl_3$) δ 7.74 – 7.64 (m, 11H), 7.63 – 7.59 (m, 4H), 7.47 (d, *J* = 15.4 Hz, 1H), 7.30 (s, 1H), 6.79 (d, *J* = 15.4 Hz, 1H); **^{13}C NMR** (100 MHz, $CDCl_3$) δ 155.0, 143.3, 139.1, 135.9, 132.7, 131.1, 130.5, 130.1, 129.4, 129.0, 128.8, 128.6, 128.5, 128.4, 128.2, 126.4; **HRMS** (EI, m/z): calcd. for $C_{22}H_{18}O_2S$ $[M]^+$: 346.1022, found: 346.1020.

4,4'-((1E,1'E)-sulfonylbis(ethene-2,1-diyl))bis(methylbenzene) (**5rb**)

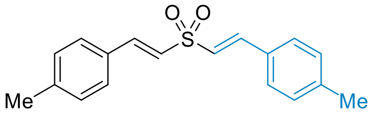

Compound **5rb** was prepared according to **General procedure P** (0.20 mmol scale, 14 h) to afford 47.3 mg (78%) as a white solid. R_f = 0.42 (Petroleum ether /EtOAc = 5/1); **m.p.** = 152 – 155 °C; **^{1}H NMR** (400 MHz, $CDCl_3$) δ 7.60 (d, *J* = 15.4 Hz, 2H), 7.40 (d, *J* = 8.1 Hz, 4H), 7.20 (d, *J* = 8.0 Hz, 4H), 6.81 (d, *J* = 15.4 Hz, 2H), 2.37 (s, 6H); **^{13}C NMR** (100 MHz, $CDCl_3$) δ 143.2, 141.9, 129.9, 128.6, 125.5, 21.6; **HRMS** (EI, m/z): calcd. for $C_{18}H_{18}O_2S$ $[M]^+$: 298.1022, found: 298.1023. The spectroscopic data of **5rb** matched the reported values.[37]

4-(*(E)*-2-((*(E)*-styryl)sulfonyl)vinyl)benzonitrile (**5rd**)

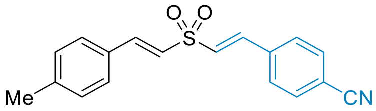

Compound **5rd** was prepared according to **General procedure P** (0.20 mmol scale, 14 h) to afford 44.8 mg (72%) as a white solid. R_f = 0.38 (Petroleum ether /EtOAc = 3/1); **m.p.** = 168 – 171 °C; **^{1}H NMR** (400 MHz, $CDCl_3$) δ 7.70 (d, *J* = 8.2 Hz, 2H), 7.64 (d, *J* = 6.3 Hz, 1H), 7.60 (d, *J* = 7.2 Hz, 3H), 7.41 (d, *J* = 8.0 Hz, 2H), 7.22 (d, *J* = 7.9 Hz, 2H), 6.97 (d, *J* = 15.5 Hz, 1H), 6.79 (d, *J* = 15.4 Hz, 1H), 2.38 (s, 3H); **^{13}C NMR** (100 MHz, $CDCl_3$) δ 144.9, 142.5, 140.5, 137.0, 132.9, 130.5, 130.0, 129.6, 129.0, 128.8, 124.4, 118.1, 114.4, 21.7; **HRMS** (EI, m/z): calcd. for $C_{18}H_{15}NO_2S$ $[M]^+$: 309.0818, found: 309.0819.

1-methoxy-4-(*(E)*-2-((*(E)*-4-methylstyryl)sulfonyl)vinyl)benzene (**5rh**)

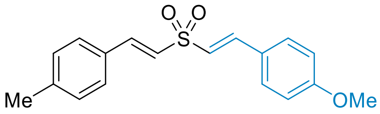

Compound **5rh** was prepared according to **General procedure P** (0.20 mmol scale, 14 h) to afford 34.6 mg (56%) as a white solid. R_f = 0.26 (Petroleum ether /EtOAc = 5/1); **m.p.** = 163 – 166 °C; **^{1}H NMR** (400 MHz, $CDCl_3$) δ 7.59 (d, *J* = 5.9 Hz, 1H), 7.55 (d, *J* = 5.9 Hz, 1H), 7.45 (d, *J* = 8.8 Hz, 2H), 7.39 (d, *J* = 8.1 Hz, 2H), 7.20 (d, *J* = 8.0 Hz, 2H), 6.91 (d, *J* = 8.8 Hz, 2H), 6.80 (d, *J* = 15.4 Hz, 1H), 6.70 (d, *J* = 15.4 Hz, 1H), 3.84 (s, 3H), 2.37 (s, 3H); **^{13}C NMR** (100 MHz, $CDCl_3$) δ 162.2, 143.0, 143.0, 141.9, 130.4, 130.1, 129.9, 128.6, 125.7, 125.3, 123.9, 114.7, 55.6, 21.6; **HRMS** (EI, m/z): calcd. for $C_{18}H_{18}O_3S$ $[M]^+$: 314.0971, found: 314.0972.

Methyl 4-(*(E)*-2-((*(E)*-4-methylstyryl)sulfonyl)vinyl)benzoate (**5rj**)

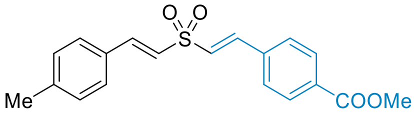

Compound **5rj** was prepared according to **General procedure P** (0.20 mmol scale, 14 h) to afford 42.7 mg (63%) as a white solid. R_f = 0.21 (Petroleum ether /EtOAc = 5/1); **m.p.** = 139 – 142 °C; **^{1}H NMR** (400 MHz, $CDCl_3$) δ 8.06 (d, *J* = 8.3 Hz, 2H), 7.65 (d, *J* = 6.5 Hz, 1H), 7.61 (d, *J* = 6.4 Hz, 1H), 7.56 (d, *J* = 8.3 Hz, 2H), 7.40 (d, *J* = 8.1 Hz, 2H), 7.21 (d, *J* = 7.9 Hz, 2H), 6.95 (d, *J* = 15.5 Hz, 1H), 6.81 (d, *J* = 15.4 Hz, 1H), 3.92 (s, 3H), 2.37 (s, 3H); **^{13}C NMR** (100 MHz, $CDCl_3$) δ 166.3, 144.3, 142.2, 141.6, 136.8, 132.3, 130.3, 130.0, 129.8, 129.2, 128.8, 128.5, 124.8, 52.5, 21.7; **HRMS** (EI, m/z): calcd. for $C_{19}H_{18}O_4S$ $[M]^+$: 342.0920, found: 342.0919.

2-(*(E)*-2-((*(E)*-4-methoxystyryl)sulfonyl)vinyl)pyridine (**5sm**)

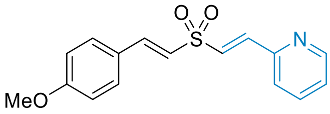

Compound **5sm** was prepared according to **General procedure P** (0.20 mmol scale, 14 h) to afford 55.3 mg (92%) as a yellow oil. R_f = 0.43 (Petroleum ether /EtOAc = 1/1); **m.p.** = 104 – 106 °C; **^{1}H NMR** (400 MHz, $CDCl_3$) δ 8.60 (d, *J* = 4.3 Hz, 1H), 7.71 (td, *J* = 7.7, 1.5 Hz, 1H), 7.58 (d, *J* = 4.2 Hz, 1H), 7.55 (d, *J* = 4.7 Hz, 1H), 7.44 – 7.37 (m, 4H), 7.29 – 7.26 (m, 1H), 6.88 (d, *J* = 8.7 Hz, 2H), 6.70 (d, *J* = 15.4 Hz, 1H), 3.80 (s, 3H); **^{13}C NMR** (100 MHz, $CDCl_3$) δ 162.2, 151.3, 150.3, 143.9, 140.9, 137.1, 131.4, 130.5, 125.4, 125.2, 125.0, 123.2, 114.6, 55.5; **HRMS** (EI, m/z): calcd. for $C_{16}H_{15}NO_3S$ $[M]^+$: 301.0767, found: 301.0766.

2-(*(E)*-2-((*(E)*-4-methoxystyryl)sulfonyl)vinyl)benzo[b]thiophene (**5sp**)

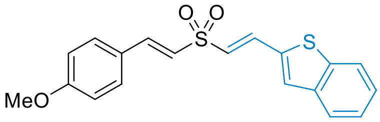

Compound **5sp** was prepared according to **General procedure P** (0.20 mmol scale, 14 h) to afford 36.0 mg (51%) as a light-yellow solid. R_f = 0.36 (Petroleum ether /EtOAc = 3/1); **m.p.** = 152 – 155 °C; **^{1}H NMR** (400 MHz, $CDCl_3$) δ 7.80 – 7.77 (m, 3H), 7.59 (d, *J* = 15.3 Hz, 1H), 7.51 – 7.46 (m, 3H), 7.41 – 7.35 (m, 2H), 6.92 (d, *J* = 8.5 Hz, 2H), 6.72 (dd, *J* = 15.2, 2.4 Hz, 2H), 3.84 (s, 3H).; **^{13}C NMR** (100

MHz, $CDCl_3$) δ 162.3, 143.7, 140.7, 139.4, 137.3, 136.0, 130.6, 130.3, 127.5, 126.8, 125.2, 124.9, 123.3, 122.6, 114.7, 55.6; **HRMS** (EI, m/z): calcd. for $C_{19}H_{16}O_3S_2$ $[M]^+$: 356.0535, found: 356.0536.

(E)-3-((4-methoxystyryl)sulfonyl)-1,2-dihydronaphthalene (**5su**)

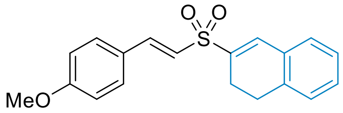

Compound **5su** was prepared according to **General procedure P** (0.20 mmol scale, 14 h) to afford 47.8 mg (73%) as a white solid. R_f = 0.38 (Petroleum ether /EtOAc = 3/1); **m.p.** = 82 – 85 °C; **^{1}H NMR** (400 MHz, $CDCl_3$) δ 7.57 (d, J = 15.4 Hz, 1H), 7.50 – 7.45 (m, 3H), 7.29 – 7.23 (m, 3H), 7.16 (d, J = 7.2 Hz, 1H), 6.92 (d, J = 8.7 Hz, 2H), 6.63 (d, J = 15.4 Hz, 1H), 3.84 (s, 3H), 2.94 (t, J = 8.2 Hz, 2H), 2.63 (t, J = 8.3 Hz, 2H); **^{13}C NMR** (100 MHz, $CDCl_3$) δ 162.2, 143.9, 137.8, 135.7, 135.4, 131.3, 130.5, 129.0, 127.9, 127.2, 125.2, 122.3, 114.6, 55.5, 27.8, 21.8; **HRMS** (EI, m/z): calcd. for $C_{19}H_{18}O_3S$ $[M]^+$: 326.0971, found: 326.0970.

(8S,9S,13S,14S)-17-oxo-7,8,9,11,12,13,14,15,16,17-decahydro-6*H*-cyclopenta[a]phenanthrene-3-yl (4-(*(E)*-2-((*(E)*-styryl)sulfonyl)vinyl)phenyl) sulfate (**5ta**)

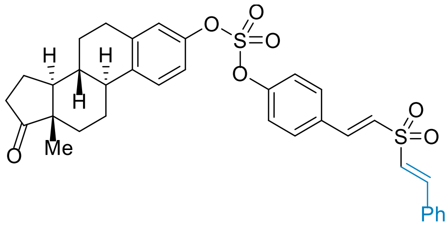

Compound **5ra** was prepared according to **General procedure P** (0.20 mmol scale, 14 h) to afford 97.9 mg (79%) as a white solid. R_f = 0.22 (Petroleum ether /EtOAc = 2/1); **m.p.** = 144 – 148 °C; **^{1}H NMR** (400 MHz, $CDCl_3$) δ; 7.66 – 7.57 (m, 4H), 7.51 (d, J = 6.8 Hz, 2H), 7.42 – 7.37 (m, 5H), 7.31 (d, J = 8.5 Hz, 1H), 7.06 – 7.02 (m, 2H), 6.89 (d, J = 8.3 Hz, 1H), 6.85 (d, J = 8.3 Hz, 1H), 2.91 (d, J = 4.6 Hz, 2H), 2.50 (dd, J = 18.8, 8.6 Hz, 1H), 2.38 (d, J = 10.8 Hz, 1H), 2.27 (t, J = 8.0 Hz, 1H), 2.18 – 1.95 (m, 4H), 1.67 – 1.39 (m, 6H), 0.90 (s, 3H); **^{13}C NMR** (100 MHz, $CDCl_3$) δ 220.5, 152.0, 148.3, 144.2, 141.4, 139.8, 139.2, 132.4, 132.0, 131.5, 130.3, 129.3, 128.7, 128.1, 127.2, 126.0, 121.9, 121.0, 118.0, 50.4, 47.9, 44.2, 37.9, 35.9, 31.6, 29.5, 26.2, 25.8, 21.7, 13.9; **HRMS** (EI, m/z): calcd. for $C_{34}H_{34}O_7S_2$ $[M]^+$: 618.1740, found: 618.1742.

(E)-(4-chlorophenyl)(5-methoxy-2-methyl-3-(3-(styrylsulfonyl)propyl)-1H-indol-1-yl)methanone (**6aa**)

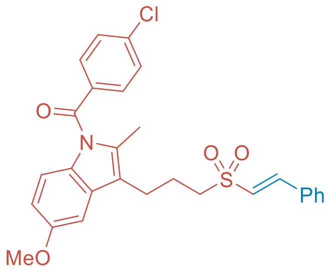

Compound **6aa** was prepared according to **General procedure P** (0.20 mmol scale, 14 h) to afford 12.2 mg (12%) as a light-yellow solid. R_f = 0.31 (Petroleum ether /EtOAc = 3/1); **m.p.** = 122 – 126 °C; **^{1}H NMR** (400 MHz, CD_3CN) δ 7.64 – 7.59 (m, 4H), 7.54 – 7.50 (m, 3H), 7.47 – 7.42 (m, 3H), 7.03 – 6.96

(m, 3H), 6.65 (dd, J = 9.0, 2.5 Hz, 1H), 3.76 (s, 3H), 3.15 – 3.11 (m, 2H), 2.83 (t, J = 7.4 Hz, 2H), 2.21 (s, 3H), 2.07 – 2.03 (m, 2H); ^{13}**C NMR** (100 MHz, CD_3CN) δ 169.2, 157.0, 144.7, 139.2, 135.7, 135.5, 133.6, 132.1, 132.0, 132.0, 131.8, 130.0, 129.9, 129.6, 126.8, 119.0, 116.0, 112.1, 102.2, 56.2, 54.6, 23.6, 22.9, 13.6; **HRMS** (EI, m/z): calcd. for $C_{28}H_{26}ClNO_3S$ $[M]^+$: 507.1266, found: 507.1265.

(E)-4-(styrylsulfonyl)-1-tosylpiperidine (**6ba**)

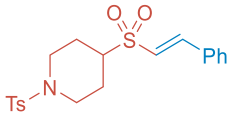

Compound **6ba** was prepared according to **General procedure P** (0.20 mmol scale, 14 h) to afford 8.3 mg (10%) as a white solid. R_f = 0.30 (Petroleum ether /EtOAc = 2/1); **m.p.** = 194 – 196 °C; ^{1}**H NMR** (400 MHz, $CDCl_3$) δ 7.63 (d, J = 8.1 Hz, 2H), 7.56 (d, J = 15.5 Hz, 1H), 7.50 (d, J = 7.8 Hz, 2H), 7.47 – 7.41 (m, 3H), 7.32 (d, J = 8.0 Hz, 2H), 6.69 (d, J = 15.5 Hz, 1H), 3.92 (d, J = 12.0 Hz, 2H), 2.79 (tt, J = 11.9, 3.5 Hz, 1H), 2.42 (s, 3H), 2.37 – 2.31 (m, 2H), 2.21 (d, J = 11.3 Hz, 2H), 1.87 (ddd, J = 24.9, 12.4, 4.1 Hz, 2H); ^{13}**C NMR** (100 MHz, $CDCl_3$) δ 147.2, 144.1, 133.2, 132.1, 131.9, 130.0, 129.4, 128.8, 127.8, 121.6, 60.0, 45.2, 25.0, 21.7; **HRMS** (ESI, m/z): calcd. for $C_{20}H_{23}NNaO_4S_2$ $[M+Na]^+$: 428.0961, found: 428.0966.

((1E,1'E)-sulfonylbis(ethene-2,1-diyl))dibenzene (**5aa**)

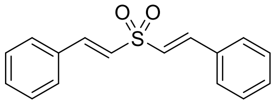

Compound **5aa** was prepared according to **General procedure Q** (0.20 mmol scale, 4 h) to afford 7.6 mg (28%) as a white solid. R_f = 0.33 (Petroleum ether /EtOAc = 5/1); **m.p.** = 98 – 101 °C; ^{1}**H NMR** (400 MHz, $CDCl_3$) δ 7.65 (d, J = 15.4 Hz, 2H), 7.52 (d, J = 5.6 Hz, 4H), 7.43 (s, 6H), 6.86 (d, J = 15.4 Hz, 2H); ^{13}**C NMR** (100 MHz, $CDCl_3$) δ 143.6, 132.7, 131.4, 129.3, 128.7, 126.6; **HRMS** (EI, m/z): calcd. for $C_{16}H_{15}NO_3S$ $[M]^+$: 270.0709, found: 270.0708.[38]

4,4'-((1E,1'E)-sulfonylbis(ethene-2,1-diyl))bis(fluorobenzene) (**5bb**)

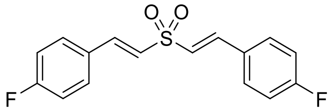

Compound **5bb** was prepared according to **General procedure Q** (0.20 mmol scale, 6 h) to afford 5.1 mg (17%) as a white solid. R_f = 0.15 (Petroleum ether /EtOAc = 10/1); **m.p.** = 175 – 179 °C; ^{1}**H NMR** (400 MHz, $CDCl_3$) δ 7.60 (d, J = 15.4 Hz, 2H), 7.51 (dd, J = 8.5, 5.4 Hz, 4H), 7.11 (t, J = 8.5 Hz, 4H), 6.78 (d, J = 15.4 Hz, 2H); ^{13}**C NMR** (100 MHz, $CDCl_3$) δ 164.5 (d, J = 252.0 Hz), 142.3, 130.6 (d, J = 8.0 Hz), 128.7 (d, J = 3.0 Hz), 126.0 (d, J = 3.0 Hz), 116.4 (d, J = 22.0 Hz); ^{19}**F NMR** (376 MHz, $CDCl_3$) δ -107.5 (s, 1F); **HRMS** (EI, m/z): calcd. for $C_{16}H_{12}F_2O_2S$ $[M]^+$: 306.0521, found: 306.0522.

4,4'-((1E,1'E)-sulfonylbis(ethene-2,1-diyl))bis((trifluoromethoxy)benzene) (**5hh**)

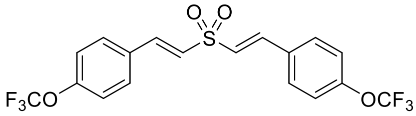

Compound **5hh** was prepared according to **General procedure Q** (0.20 mmol scale, 4 h) to afford 8.5 mg (19%) as a white solid. R_f = 0.24 (Petroleum ether /EtOAc = 10/1); **m.p.** = 157 – 161 °C; **^{1}H NMR** (400 MHz, DMSO-d^6) δ 7.92 (d, J = 8.6 Hz, 4H), 7.62 (d, J = 15.5 Hz, 2H), 7.54 (d, J = 15.5 Hz, 2H), 7.43 (d, J = 8.1 Hz, 4H); **^{13}C NMR** (100 MHz, DMSO-d^6) δ 149.9 (d, J = 2.0 Hz), 140.6, 131.9, 131.0, 128.8, 123.8, 121.3, 120.0 (q, J = 255.0 Hz); **^{19}F NMR** (376 MHz, DMSO-d^6) δ -56.8 (s, 3F); **HRMS** (EI, m/z): calcd. for $C_{16}H_{15}NO_3S$ $[M]^+$: 438.0355, found: 438.0355.

(E)-N-((4-bromostyryl)(oxo)(phenyl)-λ^6-sulfanylidene)pivalamide (**8aa**)

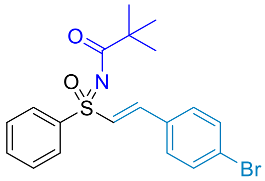

Compound **8aa** was prepared according to **General procedure R** (0.20 mmol scale, 5 h) to afford 74.0 mg (91%) as a white solid. R_f = 0.40 (Petroleum ether /EtOAc = 5/1); **^{1}H NMR** (400 MHz, $CDCl_3$) δ 7.96 (d, J = 7.6 Hz, 2H), 7.62 (d, J = 5.0 Hz, 1H), 7.60 – 7.54 (m, 3H), 7.50 (d, J = 8.4 Hz, 2H), 7.33 (d, J = 8.4 Hz, 2H), 6.91 (d, J = 15.3 Hz, 1H), 1.25 (s, 9H); **^{13}C NMR** (100 MHz, $CDCl_3$) δ 188.0, 141.8, 139.1, 133.5, 132.4, 131.4, 130.1, 129.7, 127.5, 126.7, 125.7, 41.7, 27.8; **HRMS** (ESI, m/z): calcd. for $C_{19}H_{21}BrNO_2S^+$ $[M+H]^+$: 406.0471, found: 406.0476.

(E)-N-((4-bromostyryl)(oxo)(phenyl)-λ^6-sulfanylidene)-2,2-dimethylbutanamide (**8ab**)

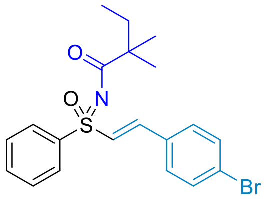

Compound **8ab** was prepared according to **General procedure R** (0.20 mmol scale, 3 h) to afford 57.7 mg (69%) as a white solid. R_f = 0.50 (Petroleum ether /EtOAc = 5/1); **^{1}H NMR** (400 MHz, $CDCl_3$) δ 7.96 (d, J = 7.7 Hz, 2H), 7.63 – 7.54 (m, 4H), 7.50 (d, J = 8.3 Hz, 2H), 7.33 (d, J = 8.4 Hz, 2H), 6.91 (d, J = 15.3 Hz, 1H), 1.66 (q, J = 15.1, 7.6 Hz, 2H), 1.20 (s, 6H), 0.88 (t, J = 7.5 Hz, 3H); **^{13}C NMR** (100 MHz, $CDCl_3$) δ 187.5, 141.8, 139.2, 133.5, 132.4, 131.4, 130.1, 129.6, 127.5, 126.8, 125.7, 45.3, 33.9, 25.2, 25.1, 9.5; **HRMS** (ESI, m/z): calcd. for $C_{20}H_{22}BrNNaO_2S^+$ $[M+Na]^+$: 442.0447, found: 442.0448.

(E)-N-((4-bromostyryl)(oxo)(phenyl)-λ^6-sulfanylidene)benzamide (**8ac**)

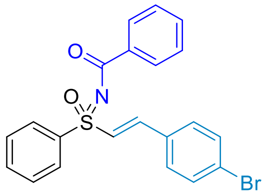

Compound **8ac** was prepared according to **General procedure R** (0.20 mmol scale, 6 h) to afford 63.9 mg (75%) as a white solid. R_f = 0.20 (Petroleum ether /EtOAc = 5/1); **^{1}H NMR** (400 MHz, $CDCl_3$) δ 8.21 (d, J = 7.8 Hz, 2H), 8.07 (d, J = 7.9 Hz, 2H), 7.71 (d, J = 15.3 Hz, 1H), 7.66 – 7.56 (m, 3H), 7.52 (d, J = 8.2 Hz, 3H), 7.42 (t, J = 7.6 Hz, 2H), 7.37 (d, J = 8.3 Hz, 2H), 7.09 (d, J = 15.3 Hz, 1H); **^{13}C NMR** (100 MHz, $CDCl_3$) δ 173.9, 142.2, 138.9, 135.8, 133.7, 132.5, 132.3, 131.3, 130.2, 129.8, 129.6,

128.2, 127.6, 126.5, 125.9; **HRMS** (ESI, m/z): calcd. for $C_{21}H_{16}BrNNaO_2S^+$ [M+Na]$^+$: 447.9977, found: 447.9977.

(E)-N-((4-bromostyryl)(oxo)(phenyl)-λ^6-sulfanylidene)cyclopent-1-ene-1-carboxamide (**8ad**)

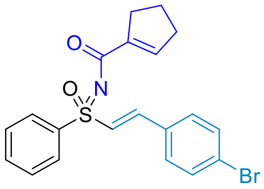

Compound **8ad** was prepared according to **General procedure R** (0.20 mmol scale, 12 h) to afford 55.5 mg (67%) as a white solid. R$_f$ = 0.40 (Petroleum ether /EtOAc = 5/1); **^{1}H NMR** (400 MHz, $CDCl_3$) δ 8.00 (d, *J* = 7.8 Hz, 2H), 7.65 – 7.60 (m, 2H), 7.56 (t, *J* = 7.5 Hz, 2H), 7.51 (d, *J* = 8.2 Hz, 2H), 7.34 (d, *J* = 8.3 Hz, 2H), 7.01 (d, *J* = 15.3 Hz, 1H), 6.87 (s, 1H), 2.60 (t, *J* = 6.5 Hz, 2H), 2.52 (t, *J* = 7.1 Hz, 2H), 1.95 (p, *J* = 7.5 Hz, 2H); **^{13}C NMR** (100 MHz, $CDCl_3$) δ 172.9, 143.0, 142.5, 141.9, 139.1, 133.5, 132.4, 131.4, 130.1, 129.7, 127.6, 126.7, 125.8, 33.6, 31.6, 23.6; **HRMS** (ESI, m/z): calcd. for $C_{20}H_{18}BrNNaO_2S^+$ [M+Na]$^+$: 438.0134, found: 438.0132.

Methyl *(E)*-((4-bromostyryl)(oxo)(phenyl)-λ^6-sulfanylidene)carbamate (**8ae**)

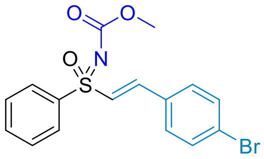

Compound **8ae** was prepared according to **General procedure R** (0.20 mmol scale, 6 h) to afford 27.9 mg (37%) as a white solid. R$_f$ = 0.40 (Petroleum ether /EtOAc = 2/1); **^{1}H NMR** (400 MHz, $CDCl_3$) δ 8.00 (d, *J* = 7.8 Hz, 2H), 7.67 – 7.63 (m, 2H), 7.58 (t, *J* = 7.6 Hz, 2H), 7.52 (d, *J* = 8.4 Hz, 2H), 7.34 (d, *J* = 8.4 Hz, 2H), 6.88 (d, *J* = 15.3 Hz, 1H), 3.68 (s, 3H); **^{13}C NMR** (100 MHz, $CDCl_3$) δ 159.2, 142.6, 138.6, 133.9, 132.5, 131.1, 130.2, 129.8, 127.8, 126.1, 126.0, 53.4; **HRMS** (ESI, m/z): calcd. for $C_{16}H_{14}BrNNaO_3S^+$ [M+Na]$^+$: 401.9770, found: 401.9773.

(E)-N-((4-bromostyryl)(oxo)(phenyl)-λ^6-sulfanylidene)-4-methylbenzenesulfonamide (**8af**)

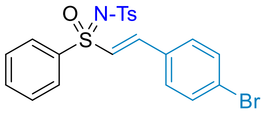

Compound **8af** was prepared according to **General procedure R** (0.20 mmol scale, 6 h) to afford 16.7 mg (18%) as a white solid. R$_f$ = 0.20 (Petroleum ether /EtOAc = 5/1); **^{1}H NMR** (400 MHz, $CDCl_3$) δ 8.01 (d, *J* = 7.9 Hz, 2H), 7.85 (d, *J* = 8.0 Hz, 2H), 7.65 (t, *J* = 7.3 Hz, 1H), 7.59 – 7.51 (m, 5H), 7.32 (d, *J* = 8.2 Hz, 2H), 7.24 (d, *J* = 8.0 Hz, 2H), 6.92 (d, *J* = 15.2 Hz, 1H), 2.37 (s, 3H); **^{13}C NMR** (100 MHz, $CDCl_3$) δ 143.0, 142.6, 140.9, 138.6, 134.2, 132.6, 130.9, 130.3, 129.8, 129.4, 127.9, 126.9, 126.6, 126.4, 21.6; **HRMS** (ESI, m/z): calcd. for $C_{21}H_{18}BrNNaO_3S_2^+$ [M+Na]$^+$: 497.9803, found: 497.9803.

(E)-N-((4-methylstyryl)(oxo)(phenyl)-λ^6-sulfanylidene)pivalamide (**8ag**)

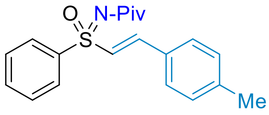

Compound **8ag** was prepared according to **General procedure R** (0.20 mmol scale, 6 h) to afford 49.3 mg (72%) as a white solid. R_f = 0.40 (Petroleum ether /EtOAc = 5/1); **^{1}H NMR** (400 MHz, $CDCl_3$) δ 7.95 (d, *J* = 7.3 Hz, 2H), 7.66 (d, *J* = 15.3 Hz, 1H), 7.62 – 7.52 (m, 3H), 7.38 (d, *J* = 8.0 Hz, 2H), 7.18 (d, *J* = 7.9 Hz, 2H), 6.85 (d, *J* = 15.3 Hz, 1H), 2.36 (s, 3H), 1.26 (s, 9H); **^{13}C NMR** (100 MHz, $CDCl_3$) δ 188.0, 143.3, 142.0, 139.7, 133.2, 129.9, 129.8, 129.6, 128.8, 127.4, 124.5, 41.7, 21.9, 21.6; **HRMS** (ESI, m/z): calcd. for $C_{20}H_{24}NO_2S^+$ $[M+H]^+$: 342.1522, found: 342.1515.

(E)-N-((4-(tert-butyl)styryl)(oxo)(phenyl)-λ^6-sulfanylidene)pivalamide (**8ah**)

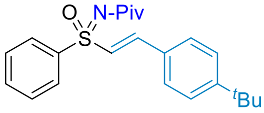

Compound **8ah** was prepared according to **General procedure R** (0.20 mmol scale, 6 h) to afford 57.1 mg (74%) as a white solid. R_f = 0.40 (Petroleum ether /EtOAc = 5/1); **^{1}H NMR** (400 MHz, $CDCl_3$) δ 7.95 (d, *J* = 7.6 Hz, 2H), 7.68 (d, *J* = 15.3 Hz, 1H), 7.61 – 7.52 (m, 3H), 7.41 (q, *J* = 8.5 Hz, 4H), 6.87 (d, *J* = 15.3 Hz, 1H), 1.30 (s, 9H), 1.26 (s, 9H); **^{13}C NMR** (100 MHz, $CDCl_3$) δ 188.0, 155.2, 143.2, 139.7, 133.2, 129.7, 129.6, 128.7, 127.4, 126.1, 124.7, 41.7, 35.1, 31.2, 27.9; **HRMS** (ESI, m/z): calcd. for $C_{23}H_{30}NO_2S^+$ $[M+H]^+$: 384.1992, found: 384.1991.

(E)-N-((4-fluorostyryl)(oxo)(phenyl)-λ^6-sulfanylidene)pivalamide (**8ai**)

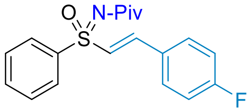

Compound **8ai** was prepared according to **General procedure R** (0.20 mmol scale, 5 h) to afford 46.7 mg (68%) as a white solid. R_f = 0.40 (Petroleum ether /EtOAc = 5/1); **^{1}H NMR** (400 MHz, $CDCl_3$) δ 7.96 (d, *J* = 7.6 Hz, 2H), 7.65 (d, *J* = 15.2 Hz, 1H), 7.61 – 7.53 (m, 3H), 7.48 (dd, *J* = 8.5, 5.4 Hz, 2H), 7.07 (t, *J* = 8.5 Hz, 2H), 6.84 (d, *J* = 15.3 Hz, 1H), 1.26 (s, 9H); **^{13}C NMR** (100 MHz, $CDCl_3$) δ 188.0, 164.5 (d, *J* = 252.0 Hz), 141.9, 139.4, 133.4, 130.8 (d, *J* = 9.0 Hz), 129.7, 128.8 (d, *J* = 3.0 Hz), 127.5, 125.7 (d, *J* = 2.0 Hz), 116.4 (d, *J* = 22.0 Hz), 41.7, 21.9; **^{19}F NMR** (376 MHz, $CDCl_3$) δ -107.7 (s, 1F); **HRMS** (ESI, m/z): calcd. for $C_{19}H_{21}FNO_2S^+$ $[M+H]^+$: 346.1272, found: 346.1277.

(E)-N-((4-chlorostyryl)(oxo)(phenyl)-λ^6-sulfanylidene)pivalamide (**8aj**)

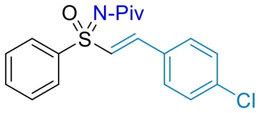

Compound **8aj** was prepared according to **General procedure R** (0.20 mmol scale, 5 h) to afford 54.9 mg (76%) as a white solid. R_f = 0.40 (Petroleum ether /EtOAc = 5/1); **^{1}H NMR** (400 MHz, $CDCl_3$) δ7.96 (d, *J* = 7.6 Hz, 2H), 7.65 – 7.60 (m, 2H), 7.56 (t, *J* = 7.5 Hz, 2H), 7.41 (d, *J* = 8.5 Hz, 2H), 7.34 (d, *J* = 8.5 Hz, 2H), 6.89 (d, *J* = 15.3 Hz, 1H), 1.26 (s, 9H); **^{13}C NMR** (100 MHz, $CDCl_3$) δ 188.0, 141.7, 139.2, 137.4, 133.5, 131.0, 129.9, 129.7, 129.4, 127.5, 126.6, 41.7, 27.8; **HRMS** (ESI, m/z): calcd. for $C_{19}H_{21}ClNO_2S^+$ $[M+H]^+$: 362.0976, found: 362.0981.

(E)-N-((4-cyanostyryl)(oxo)(phenyl)-λ^6-sulfanylidene)pivalamide (**8ak**)

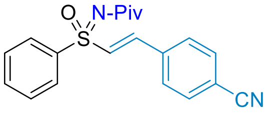

Compound **8ak** was prepared according to **General procedure R** (0.20 mmol scale, 2 h) to afford 61.5 mg (87%) as a white solid. R_f = 0.20 (Petroleum ether /EtOAc = 5/1); **^{1}H NMR** (400 MHz, $CDCl_3$) δ 7.96 (d, 2H), 7.67 – 7.62 (m, 4H), 7.58 – 7.55 (m, 4H), 7.03 (d, *J* = 15.4 Hz, 1H), 1.25 (s, 9H); **^{13}C NMR** (100 MHz, $CDCl_3$) δ 188.1, 140.5, 138.4, 136.7, 133.8, 132.8, 130.1, 129.8, 129.1, 127.6, 118.1, 114.3, 41.7, 27.8; **HRMS** (ESI, m/z): calcd. for $C_{20}H_{21}N_2O_2S^+$ [M+H]$^+$: 353.1318, found: 353.1320.

(E)-N-(oxo(phenyl)(4-(trifluoromethyl)styryl)-λ^6-sulfanylidene)pivalamide (**8al**)

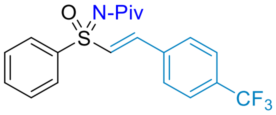

Compound **8al** was prepared according to **General procedure R** (0.20 mmol scale, 2 h) to afford 65.6 mg (83%) as a white solid. R_f = 0.40 (Petroleum ether /EtOAc = 5/1); **^{1}H NMR** (400 MHz, $CDCl_3$) δ 7.98 (d, *J* = 7.7 Hz, 2H), 7.69 (d, *J* = 15.3 Hz, 1H), 7.65 – 7.55 (m, 7H), 7.02 (d, *J* = 15.3 Hz, 1H); **^{13}C NMR** (100 MHz, $CDCl_3$) δ 188.0, 141.1, 138.8, 135.9, 133.7, 132.7 (q, *J* = 33.0 Hz), 129.8, 129.0, 128.9, 127.6, 126.1 (q, *J* = 4.0 Hz), 123.7 (q, *J* = 271.0 Hz), 41.8, 27.8; **^{19}F NMR** (376 MHz, $CDCl_3$) δ -63.0 (s, 3F); **HRMS** (ESI, m/z): calcd. for $C_{20}H_{21}F_3NO_2S^+$ [M+H]$^+$: 396.1240, found: 396.1244.

(E)-N-((3-chlorostyryl)(oxo)(phenyl)-λ^6-sulfanylidene)pivalamide (**8am**)

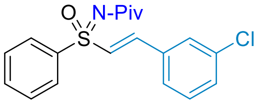

Compound **8am** was prepared according to **General procedure R** (0.20 mmol scale, 6 h) to afford 57.6 mg (80%) as a colourless oil. R_f = 0.35 (Petroleum ether /EtOAc = 5/1); **^{1}H NMR** (400 MHz, $CDCl_3$) δ 7.96 (d, *J* = 8.0 Hz, 2H), 7.64 – 7.54 (m, 4H), 7.45 (s, 1H), 7.37 – 7.26 (m, 3H), 6.93 (d, *J* = 15.3 Hz, 1H), 1.26 (s, 9H); **^{13}C NMR** (100 MHz, $CDCl_3$) δ 188.2, 141.5, 139.0, 135.2, 134.2, 133.5, 131.1, 130.4, 129.7, 128.3, 127.7, 127.5, 127.1, 41.7, 27.8; **HRMS** (ESI, m/z): calcd. for $C_{19}H_{21}ClNO_2S^+$ [M+H]$^+$: 362.0976, found: 362.0981.

(E)-N-((2-chlorostyryl)(oxo)(phenyl)-λ^6-sulfanylidene)pivalamide (**8an**)

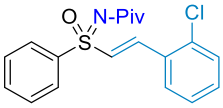

Compound **8an** was prepared according to **General procedure R** (0.20 mmol scale, 6 h) to afford 63.3 mg (87%) as a colourless oil. R_f = 0.35 (Petroleum ether /EtOAc = 5/1); **^{1}H NMR** (400 MHz, $CDCl_3$) δ 8.10 (d, *J* = 15.3 Hz, 1H), 7.97 (d, *J* = 7.7 Hz, 2H), 7.64 – 7.52 (m, 4H), 7.41 (d, *J* = 8.0 Hz, 1H), 7.32 (t, *J* = 7.4 Hz, 1H), 7.27 – 7.23 (m, 1H), 6.96 (d, *J* = 15.3 Hz, 1H), 1.27 (s, 9H); **^{13}C NMR** (100 MHz, $CDCl_3$) δ 187.9, 139.0, 138.9, 135.4, 133.5, 132.1, 130.7, 130.4, 129.7, 128.7, 128.5, 127.7, 127.3, 41.7, 27.8; **HRMS** (ESI, m/z): calcd. for $C_{19}H_{21}ClNO_2S^+$ [M+H]$^+$: 362.0976, found: 362.0977.

(E)-N-((2-methoxystyryl)(oxo)(phenyl)-λ^6-sulfanylidene)pivalamide (**8ao**)

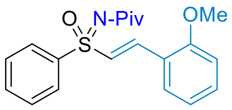

Compound **8ao** was prepared according to **General procedure R** (0.20 mmol scale, 6 h) to afford 44.6 mg (62%) as a white solid. R_f = 0.30 (Petroleum ether /EtOAc = 5/1); **^{1}H NMR** (400 MHz, $CDCl_3$) δ 7.97 – 7.91 (m, 3H), 7.60 – 7.51 (m, 3H), 7.42 (d, *J* = 7.6 Hz, 1H), 7.37 (t, *J* = 7.9 Hz, 1H), 7.07 (d, *J* = 15.3 Hz, 1H), 6.95 (t, *J* = 7.5 Hz, 1H), 6.90 (d, *J* = 8.4 Hz, 1H), 3.86 (s, 3H), 1.26 (s, 9H); **^{13}C NMR** (100 MHz, $CDCl_3$) δ 187.9, 158.8, 139.9, 139.2, 133.0, 132.7, 130.9, 129.5, 127.4, 126.1, 121.3, 120.9, 111.4, 55.7, 41.6, 27.9; **HRMS** (ESI, m/z): calcd. for $C_{20}H_{24}NO_3S^+$ [M+H]$^+$: 358.1471, found: 358.1470.

(E)-N-((2-(naphthalen-2-yl)vinyl)(oxo)(phenyl)-λ^6-sulfanylidene)pivalamide (**8ap**)

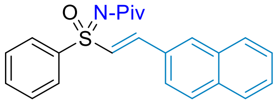

Compound **8ap** was prepared according to **General procedure R** (0.20 mmol scale, 2 h) to afford 68.8 mg (91%) as a white solid. R_f = 0.20 (Petroleum ether /EtOAc = 5/1); **^{1}H NMR** (400 MHz, $CDCl_3$) δ 8.00 (d, *J* = 7.5 Hz, 2H), 7.94 (s, 1H), 7.87 – 7.81 (m, 4H), 7.64 – 7.52 (m, 6H), 7.02 (d, *J* = 15.3 Hz, 1H), 1.29 (s, 9H); **^{13}C NMR** (100 MHz, $CDCl_3$) δ 188.0, 143.3, 139.7, 134.7, 133.4, 133.3, 131.1, 130.0, 129.7, 129.1, 128.8, 128.0, 127.5, 127.1, 125.9, 123.8, 41.8, 27.9; **HRMS** (ESI, m/z): calcd. for $C_{23}H_{23}NNaO_2S^+$ [M+Na]$^+$: 400.1341, found: 400.1340.

(E)-N-(oxo(phenyl)(2-(pyridin-2-yl)vinyl)-λ^6-sulfanylidene)pivalamide (**8aq**)

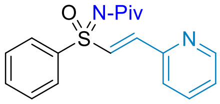

Compound **8aq** was prepared according to **General procedure R** (0.20 mmol scale, 2 h) to afford 57.7 mg (88%) as a colourless oil. R_f = 0.20 (Petroleum ether /EtOAc = 5/1); **^{1}H NMR** (400 MHz, $CDCl_3$) δ 8.58 (d, *J* = 4.5 Hz, 1H), 7.98 (d, *J* = 7.4 Hz, 2H), 7.73 – 7.70 (m, 1H), 7.67 (d, *J* = 14.8 Hz, 1H), 7.62 – 7.52 (m, 3H), 7.46 – 7.39 (m, 2H), 7.29 – 7.26 (m, 1H), 1.25 (s, 9H); **^{13}C NMR** (100 MHz, $CDCl_3$) δ 188.1, 151.1, 150.3, 141.4, 138.8, 137.2, 133.5, 130.8, 129.7, 127.7, 125.7, 125.1, 41.7, 27.8; **HRMS** (ESI, m/z): calcd. for $C_{18}H_{21}N_2O_2S^+$ [M+H]$^+$: 329.1318, found: 329.1323.

(E)-N-((4-bromostyryl)(naphthalen-2-yl)(oxo)-λ^6-sulfanylidene)pivalamide (**8ar**)

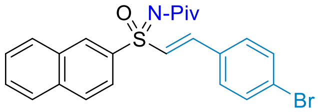

Compound **8ar** was prepared according to **General procedure R** (0.20 mmol scale, 1 h) to afford 78.3 mg (86%) as a white solid. R_f = 0.20 (Petroleum ether /EtOAc = 10/1); **^{1}H NMR** (400 MHz, $CDCl_3$) δ 8.58 (s, 1H), 8.00 – 7.97 (m, 2H), 7.91 – 7.84 (m, 2H), 7.68 – 7.59 (m, 3H), 7.50 (d, *J* = 8.4 Hz, 2H), 7.34 (d, *J* = 8.4 Hz, 2H), 6.98 (d, *J* = 15.3 Hz, 1H), 1.29 (s, 9H); **^{13}C NMR** (100 MHz, $CDCl_3$) δ 188.1, 141.8, 135.9, 135.3, 132.6, 132.4, 131.4, 130.1, 130.1, 129.5, 129.4, 129.4, 128.1, 127.8, 126.8, 125.8, 122.0, 41.8, 27.9; **HRMS** (ESI, m/z): calcd. for $C_{23}H_{22}BrNNaO_2S^+$ [M+Na]$^+$: 478.0447, found: 478.0445.

(E)-N-((4-bromostyryl)(4-(tert-butyl)phenyl)(oxo)-λ^6-sulfanylidene)pivalamide (**8as**)

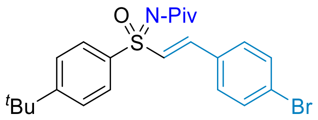

Compound **8as** was prepared according to **General procedure R** (0.20 mmol scale, 6 h) to afford 63.9 mg (69%) as a white solid. R_f = 0.50 (Petroleum ether /EtOAc = 5/1); **^{1}H NMR** (400 MHz, $CDCl_3$) δ 7.86 (d, *J* = 8.5 Hz, 2H), 7.60 (d, *J* = 15.3 Hz, 1H), 7.56 (d, *J* = 8.5 Hz, 2H), 7.50 (d, *J* = 8.4 Hz, 2H), 7.34 (d, *J* = 8.4 Hz, 2H), 6.90 (d, *J* = 15.3 Hz, 1H), 1.33 (s, 9H), 1.26 (s, 9H); **^{13}C NMR** (100 MHz, $CDCl_3$) δ 188.1, 157.5, 141.3, 135.9, 132.4, 131.6, 130.1, 127.4, 127.1, 126.8, 125.6, 41.7, 35.4, 31.2, 27.9; **HRMS** (ESI, m/z): calcd. for $C_{23}H_{28}BrNNaO_2S^+$ [M+Na]$^+$: 484.0916, found: 484.0919.

(E)-N-((4-bromostyryl)(4-methoxyphenyl)(oxo)-λ^6-sulfanylidene)pivalamide (**8at**)

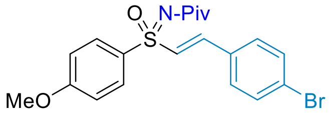

Compound **8at** was prepared according to **General procedure R** (0.20 mmol scale, 12 h) to afford 55.5 mg (64%) as a white solid. R_f = 0.20 (Petroleum ether /EtOAc = 5/1); **^{1}H NMR** (400 MHz, $CDCl_3$) δ 7.88 (d, *J* = 8.9 Hz, 2H), 7.55 (d, *J* = 15.3 Hz, 1H), 7.50 (d, *J* = 8.4 Hz, 2H), 7.33 (d, *J* = 8.4 Hz, 2H), 7.02 (d, *J* = 8.9 Hz, 2H), 6.91 (d, *J* = 15.3 Hz, 1H), 3.86 (s, 3H), 1.25 (s, 9H); **^{13}C NMR** (100 MHz, $CDCl_3$) δ 188.0, 163.8, 140.8, 132.4, 131.6, 130.0, 129.9, 129.8, 127.4, 125.5, 115.0, 55.9, 41.7, 27.9; **HRMS** (ESI, m/z): calcd. for $C_{20}H_{22}BrNNaO_3S^+$ [M+Na]$^+$: 458.0396, found: 458.0398.

(E)-N-((4-bromostyryl)(oxo)(4-(trifluoromethoxy)phenyl)-λ^6-sulfanylidene)pivalamide (**8au**)

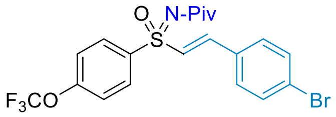

Compound **8au** was prepared according to **General procedure R** (0.20 mmol scale, 4 h) to afford 38.9 mg (40%) as a white solid. R_f = 0.50 (Petroleum ether /EtOAc = 5/1); **^{1}H NMR** (400 MHz, $CDCl_3$) δ 8.00 (d, *J* = 8.8 Hz, 2H), 7.65 (d, *J* = 15.3 Hz, 1H), 7.53 (d, *J* = 8.4 Hz, 2H), 7.37 (t, *J* = 8.3 Hz, 4H), 6.88 (d, *J* = 15.3 Hz, 1H), 1.25 (s, 9H); **^{13}C NMR** (100 MHz, $CDCl_3$) δ 188.0, 152.9, 142.6, 137.6, 132.5, 131.2, 130.2, 129.7, 126.2, 126.1, 121.5, 120.3 (q, *J* = 258.0 Hz) 41.8, 27.8; **^{19}F NMR** (376 MHz, $CDCl_3$) δ -57.7 (s, 3F); **HRMS** (ESI, m/z): calcd. for $C_{20}H_{19}BrF_3NNaO_3S^+$ [M+Na]$^+$: 512.0113, found: 512.0118.

(E)-N-((4-bromostyryl)(oxo)(4-(trifluoromethyl)phenyl)-λ^6-sulfanylidene)pivalamide (**8av**)

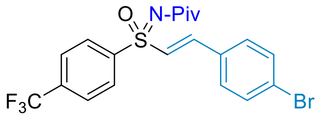

Compound **8av** was prepared according to **General procedure R** (0.20 mmol scale, 2 h) to afford 64.7 mg (71%) as a white solid. R_f = 0.40 (Petroleum ether /EtOAc = 5/1); **^{1}H NMR** (400 MHz, $CDCl_3$) δ 8.07 (d, *J* = 8.3 Hz, 2H), 7.81 (d, *J* = 8.3 Hz, 2H), 7.68 (d, *J* = 15.3 Hz, 1H), 7.53 (d, *J* = 8.4 Hz, 2H), 7.36 (d, *J* = 8.4 Hz, 2H), 6.88 (d, *J* = 15.3 Hz, 1H), 1.25 (s, 9H); **^{13}C NMR** (100 MHz, $CDCl_3$) δ 188.1, 143.4, 143.3, 135.1 (q, *J* = 33.0 Hz), 132.5, 131.0, 130.2, 128.0, 126.84 (q, *J* = 4.0 Hz), 126.3, 125.5, 123.2 (q, *J* = 271.0 Hz), 41.7, 21.7; **^{19}F NMR** (376 MHz, $CDCl_3$) δ -63.2 (s, 3F); **HRMS** (ESI, m/z): calcd. for $C_{20}H_{19}BrF_3NNaO_2S^+$ [M+Na]$^+$: 496.0164, found: 496.0166.

(E)-N-((4-bromostyryl)(4-fluorophenyl)(oxo)-λ6-sulfanylidene)pivalamide (**8aw**)

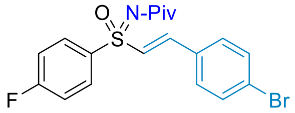

Compound **8aw** was prepared according to **General procedure R** (0.20 mmol scale, 8 h) to afford 60.9 mg (72%) as a white solid. R_f = 0.35 (Petroleum ether /EtOAc = 5/1); **^{1}H NMR** (400 MHz, $CDCl_3$) δ 7.96 (dd, *J* = 8.7, 5.0 Hz, 2H), 7.61 (d, *J* = 15.3 Hz, 1H), 7.51 (d, *J* = 8.3 Hz, 2H), 7.34 (d, *J* = 8.3 Hz, 2H), 7.23 (t, *J* = 8.5 Hz, 2H), 6.89 (d, *J* = 15.3 Hz, 1H), 1.24 (s, 9H); **^{13}C NMR** (100 MHz, $CDCl_3$) δ 187.9, 165.7 (d, *J* = 255.0 Hz), 142.0, 135.0 (d, *J* = 3.0 Hz), 132.4, 131.2, 130.3 (d, *J* = 9.0 Hz), 130.1, 126.5, 125.9, 117.0 (d, *J* = 23.0 Hz), 41.7, 27.8; **^{19}F NMR** (376 MHz, $CDCl_3$) δ -104.0 (s, 1F); **HRMS** (ESI, m/z): calcd. for $C_{19}H_{19}BrFNNaO_2S^+$ $[M+Na]^+$: 446.0196, found: 446.0194.

(E)-N-((4-bromostyryl)(4-chlorophenyl)(oxo)-λ6-sulfanylidene)pivalamide (**8ax**)

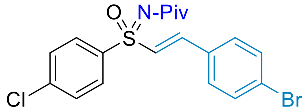

Compound **8ax** was prepared according to **General procedure R** (0.20 mmol scale, 4 h) to afford 76.4 mg (87%) as a white solid. R_f = 0.40 (Petroleum ether /EtOAc = 5/1); **^{1}H NMR** (400 MHz, $CDCl_3$) δ 7.88 (d, *J* = 8.5 Hz, 2H), 7.62 (d, *J* = 15.3 Hz, 1H), 7.52 (d, *J* = 8.5 Hz, 4H), 7.34 (d, *J* = 8.3 Hz, 2H), 6.88 (d, *J* = 15.3 Hz, 1H), 1.24 (s, 9H); **^{13}C NMR** (100 MHz, $CDCl_3$) δ 188.0, 142.4, 140.2, 137.8, 132.9, 131.2, 130.2, 130.0, 128.9, 126.2, 126.0, 41.7, 27.8; **HRMS** (ESI, m/z): calcd. for $C_{19}H_{19}BrClNNaO_2S^+$ $[M+Na]^+$: 461.9900, found: 461.9903.

(E)-N-((4-bromostyryl)(3-chlorophenyl)(oxo)-λ6-sulfanylidene)pivalamide (**8ay**)

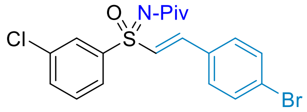

Compound **8ay** was prepared according to **General procedure R** (0.20 mmol scale, 2 h) to afford 68.6 mg (78%) as a white solid. R_f = 0.20 (Petroleum ether /EtOAc = 10/1); **^{1}H NMR** (400 MHz, $CDCl_3$) δ 7.91 (s, 1H), 7.82 (d, *J* = 7.8 Hz, 1H), 7.63 (d, *J* = 15.3 Hz, 1H), 7.57 (d, *J* = 8.0 Hz, 1H), 7.53 – 7.47 (m, 3H), 7.35 (d, *J* = 8.3 Hz, 2H), 6.87 (d, *J* = 15.3 Hz, 1H), 1.25 (s, 9H); **^{13}C NMR** (100 MHz, $CDCl_3$) δ 188.0, 142.7, 141.2, 135.9, 133.6, 132.5, 131.1, 131.0, 130.2, 127.5, 126.1, 125.9, 125.5, 41.7, 27.8; **HRMS** (ESI, m/z): calcd. for $C_{19}H_{19}BrClNNaO_2S^+$ $[M+Na]^+$: 461.9900, found: 461.9902.

(E)-N-((3,5-bis(trifluoromethyl)phenyl)(4-bromostyryl)(oxo)-λ6-sulfanylidene)pivalamide (**8az**)

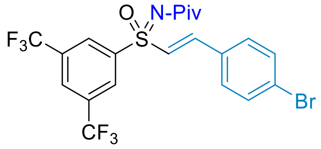

Compound **8az** was prepared according to **General procedure R** (0.20 mmol scale, 1 h) to afford 43.1 mg (40%) as a white solid. R_f = 0.50 (Petroleum ether /EtOAc = 10/1); **^{1}H NMR** (400 MHz, $CDCl_3$) δ 8.35 (s, 2H), 8.09 (s, 1H), 7.75 (d, *J* = 15.2 Hz, 1H), 7.56 (d, *J* = 8.3 Hz, 2H), 7.40 (d, *J* = 8.3 Hz, 2H), 6.86 (d, *J* = 15.2 Hz, 1H), 1.25 (s, 9H); **^{13}C NMR** (100 MHz, $CDCl_3$) δ 188.2, 144.7, 143.2, 133.6 (q, *J* = 34.0 Hz), 132.7, 130.8, 130.4, 127.7, 127.0, 126.8, 124.4, 122.5 (q, *J* = 272.0 Hz), 41.8, 27.6; **^{19}F NMR**

(376 MHz, $CDCl_3$) δ -63.0 (s, 6F); **HRMS** (ESI, m/z): calcd. for $C_{21}H_{18}BrF_6NNaO_2S^+$ $[M+Na]^+$: 564.0038, found: 564.0034.

Section 5. The control experiments

5.1 Radical-clock experiment

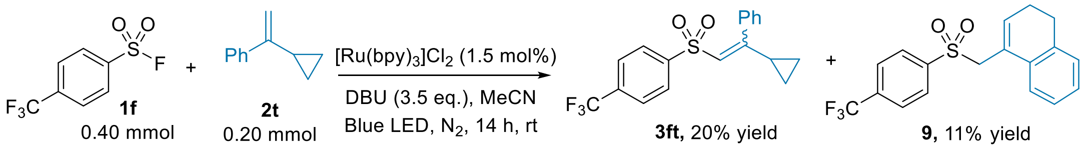

To a 10 mL Schlenk tube equipped with a magnetic stirring bar was charged sulfonyl fluoride **1f** (0.40 mmol, 2.0 eq.) and $[Ru(bpy)_3]Cl_2$ (0.003 mmol, 1.5 mol%) under N_2 atmosphere. Subsequently, dry MeCN (2.0 mL), olefin **2t** (0.20 mmol, 1.0 eq.), and DBU (0.70 mmol, 3.5 eq.) were added. The mixture was illuminated with blue LED and stirred at room temperature for 14 h. Compound **3ft** and **9** were detected by GC-MS, and yields were determined by ^{1}H NMR analysis of the crude products using 0.10 mmol of CH_2Br_2 as the internal standard. The products were purified by silica gel chromatography.

***(E)*-3ft**: White solid, **m.p.** = 105 – 108 °C; **^{1}H NMR** (400 MHz, $CDCl_3$) δ 8.14 (d, J = 8.2 Hz, 2H), 7.83 (d, J = 8.3 Hz, 2H), 7.33 – 7.28 (m, 3H), 7.05 – 7.03 (m, 2H), 6.24 (s, 1H), 2.92 (tt, J = 8.3, 5.2 Hz, 1H), 0.94 – 0.90 (m, 2H), 0.52 – 0.48 (m, 2H); **^{13}C NMR** (100 MHz, $CDCl_3$) δ 162.8.0, 146.2, 136.3, 134.8 (q, J = 33.0 Hz), 128.8, 128.1, 127.9, 127.8, 127.5, 126.4 (q, J = 4.0 Hz), 123.3 (q, J = 271.0 Hz), 12.9, 7.7; **^{19}F NMR** (376 MHz, $CDCl_3$) δ -63.1 (s, 3F); **HRMS** (EI, m/z): calcd. for $C_{18}H_{15}F_3O_2S$ $[M]^+$: 352.0739, found: 352.0740.

***(Z)*-3ft**: colorless oil; **^{1}H NMR** (400 MHz, $CDCl_3$) δ 7.56 – 7.52 (m, 4H), 7.29 (t, J = 7.0 Hz, 1H), 7.20 (t, J = 7.4 Hz, 2H), 6.83 (d, J = 7.5 Hz, 2H), 6.57 (s, 1H), 1.72 – 1.67 (m, 1H), 0.85 (d, J = 7.8 Hz, 2H), 0.52 – 0.50 (m, 2H); **^{19}F NMR** (376 MHz, $CDCl_3$) δ -63.2 (s, 3F); **HRMS** (EI, m/z): calcd. for $C_{18}H_{15}F_3O_2S$ $[M]^+$: 352.0739, found: 352.0740.

Compound **9:** White solid, **m.p.** = 94 – 97 °C; **^{1}H NMR** (400 MHz, $CDCl_3$) δ 7.91 (d, J = 8.1 Hz, 2H), 7.65 (d, J = 8.1 Hz, 2H), 7.09 – 6.99 (m, 4H), 6.00 (t, J = 4.4 Hz, 1H), 4.26 (s, 2H), 2.68 (t, J = 8.0 Hz, 2H), 2.24 (dd, J = 12.4, 7.8 Hz, 2H); **^{13}C NMR** (100 MHz, $CDCl_3$) δ 142.0, 136.0, 135.5 (q, J = 33.0 Hz), 135.5, 132.3, 129.5, 127.9, 127.8, 126.5, 125.9 (q, J = 4.0 Hz), 125.7, 123.2(q, J = 272.0 Hz), 123.0, 60.2, 27.8, 23.5; **^{19}F NMR** (376 MHz, $CDCl_3$) δ -63.3 (s, 3F); **HRMS** (EI, m/z): calcd. for $C_{18}H_{15}F_3O_2S$ $[M]^+$: 352.0739, found: 352.0739. The spectroscopic data of **9** matched the reported values.[39]

5.2 Radical inhibition experiments

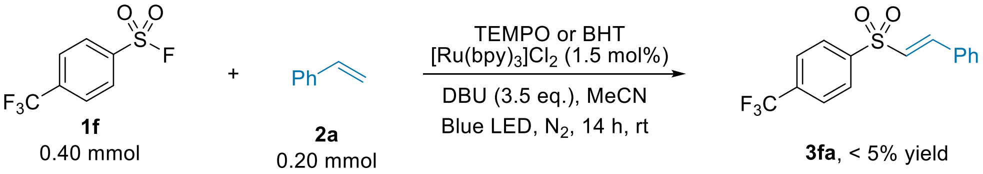

To a 10 mL Schlenk tube equipped with a magnetic stirring bar was charged sulfonyl fluoride (0.40 mmol, 2.0 eq.), $[Ru(bpy)_3]Cl_2$ (0.003 mmol, 1.5 mol%), TEMPO or BHT(0.4 mmol, 2.0 eq.) under N_2 atmosphere. Subsequently, dry MeCN (2.0 mL), olefins (0.20 mmol, 1.0 eq.), and DBU (0.70 mmol, 3.5 eq.) were added. The mixture was illuminated with blue LED and stirred at room temperature for 14 h. After that, the reaction mixture was monitored by GC-MS. The yield of **3fa** was determined by ^{1}H NMR analysis of the crude products using 0.10 mmol CH_2Br_2 as the internal standard.

5.3 Intermediate exclusion experiments

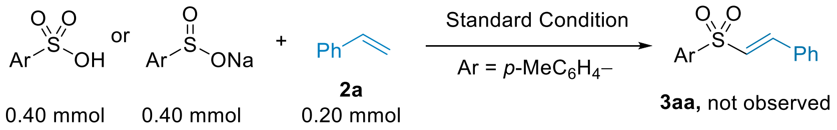

To a 10 mL Schlenk tube equipped with a magnetic stirring bar was charged sulfonate $ArSO_3H$ or sulfinate $ArSO_2^-$ salts (0.40 mmol, 2.0 eq.), $[Ru(bpy)_3]Cl_2$ (0.003 mmol, 1.5 mol%), under N_2 atmosphere. Subsequently, dry MeCN (2.0 mL), olefins (0.20 mmol, 1.0 eq.), and DBU (0.70 mmol, 3.5 eq.) were added. The mixture was illuminated with blue LED and stirred at room temperature for 14 h. After that, the reaction mixture was monitored by GC-MS.

5.4 The essential rule of DBU for S^{VI}-F activation[21]

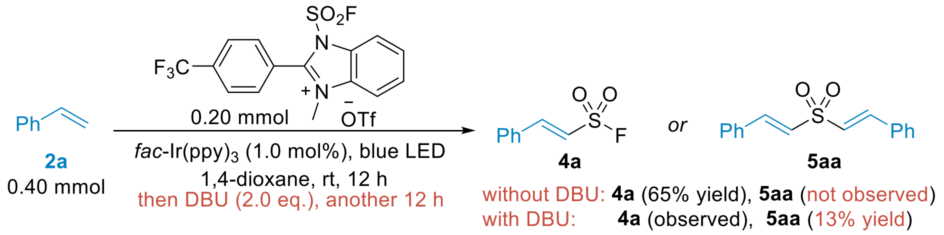

Two parallel reactions were performed as followed: to a 10 mL Schlenk tube equipped with a magnetic stirring bar was charged imidazolium salt (0.20 mmol, 1.0 eq.), *fac*-Ir(ppy)$_3$ (0.001 mmol, 1 mol%), under N_2 atmosphere. Subsequently, anhydrous 1,4-dioxane (4.0 mL, 0.025 M), olefins (0.40 mmol, 2.0 eq.) were added. The mixture was illuminated with blue LED and stirred at room temperature for 12 h. Then the mixture of one tube was determined by GC-MS and ^{1}H NMR, the other tube was added DBU (0.40 mmol, 2.0 eq.) under N_2 atmosphere react another 12 h. After that, the reaction mixture was monitored by GC-MS. The yield of **4a** and **5aa** was determined by ^{1}H NMR analysis of the crude products using 0.10 mmol CH_2Br_2 as the internal standard.

5.5. Hammett plot analysis[40]

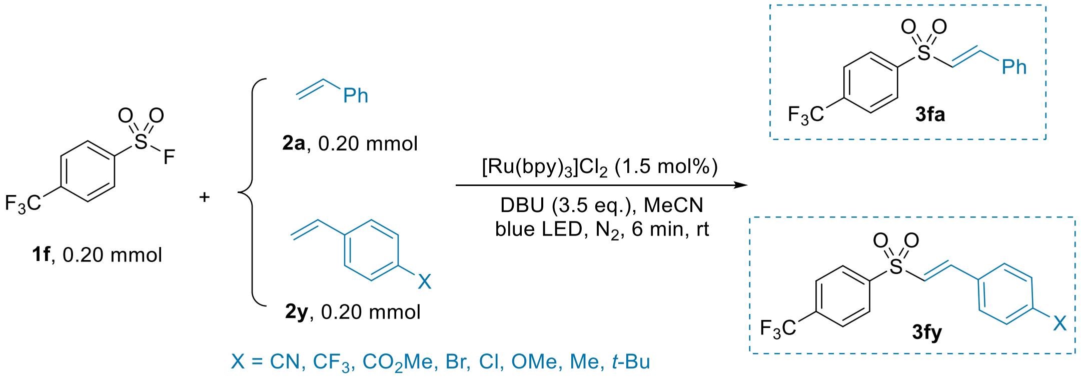

Hammett studies were conducted by competition experiments between styrene (**2a**) and *para*-substituted styrene derivatives (R= CN, CF_3, $COOCH_3$, Br, Cl, OMe, Me, *t*-Bu). The difference in reaction rates were reported as the relative ratios of products **3fy** and **3fa**. The competition experiments were carried out by the following procedure. To a 10 mL Schlenk tube equipped with a magnetic stirring bar was charged **1f** (0.20 mmol, 1.0 eq.) and $[Ru(bpy)_3]Cl_2$ (0.003 mmol, 1.5 mol%) under N_2 atmosphere.

Subsequently, dry MeCN (2.0 mL), **2a** (0.20 mmol, 1.0 eq.), **2y** (0.20 mmol, 1.0 eq.) and DBU (0.70 mmol, 3.5 eq.) were added. The tube was illuminated with blue LEDs for 6 mins. The GC yields of products **3fy** and **3fa** were monitored with external standard method. Each group of competition reactions were performed three times in parallel, and the results were recorded as X/H^1, X/H^2, X/H^3. Based on these three groups of data, we were able to get the average yields ratio of X/H. The analysis was summarized below.

Supplementary Table 6. The original data of Hammett study experiments

X	X/H^1	X/H^2	X/H^3	average X/H	log(X/H)
t-Bu (δ = -0.15)	2.01	1.93	2.05	2.00	0.30
Me (δ = -0.14)	1.95	1.95	2.01	1.97	0.29
OMe (δ = -0.12)	2.84	2.87	2.97	2.89	0.46
Cl (δ = 0.24)	0.88	0.93	0.89	0.90	-0.05
Br (δ = 0.26)	0.77	0.81	0.85	0.81	-0.09
$COOCH_3$ (δ = 0.45)	0.52	0.47	0.56	0.51	-0.29
CF_3 (δ = 0.53)	0.47	0.48	0.48	0.48	-0.32
CN (δ = 0.70)	0.28	0.26	0.26	0.27	-0.57

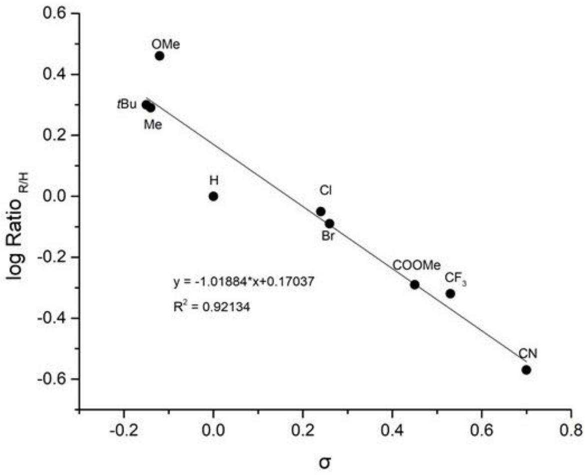

Supplementary Figure 7. Hammett plot analysis

5.6 NMR experiments

5.6.1 The reaction of **1f** and styrene in CD_3CN

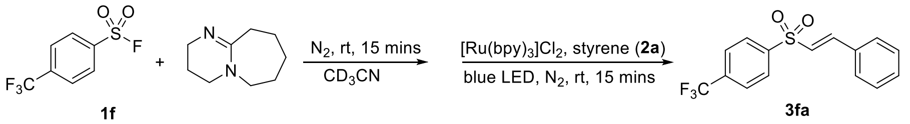

Under the N_2 atmosphere, the NMR spectra of sulfonyl fluoride (**1f**, 0.10 mmol, 1.0 eq.) and 1,3,5-trifluorobenzene (internal standard. 0.10 mmol, 1.0 eq.) in CD_3CN (0.6 mL) were recorded (**Supplementary Figure 8-1**, **9-1**). DBU (0.20 mmol, 2.0 eq.) was then added into the NMR tube. The resulting mixture was shaken for 15 min, and then another group of NMR spectra were collected (**Supplementary Figure 8-2, 9-2**). We found two new species, namely the $ArSO_3H$ and the **Int**, which was likely $RSO_2[DBU]^+$ (confirmed by Mass Spectrum, **Supplementary Figure 10**). Thereafter, the photocatalyst $Ru(bpy)_3Cl_2$ (1.5 mol%) and styrene (**2a**, 0.10 mmol, 1.0 eq.) were added into the NMR tube. The resulting mixture was shaken under the blue LED irradiation (5 W). Another group of NMR spectra were collected 5 mins later, and the full consumption of sulfonyl fluoride and **Int** were observed (**Supplementary Figure 8-3**, **9-3**). No further change was observed after 15 mins of further reaction time (**Supplementary Figure 8-4, 9-4**). The formation of product **3fa** was confirmed by standard sample (**Supplementary Figure 8-5**).

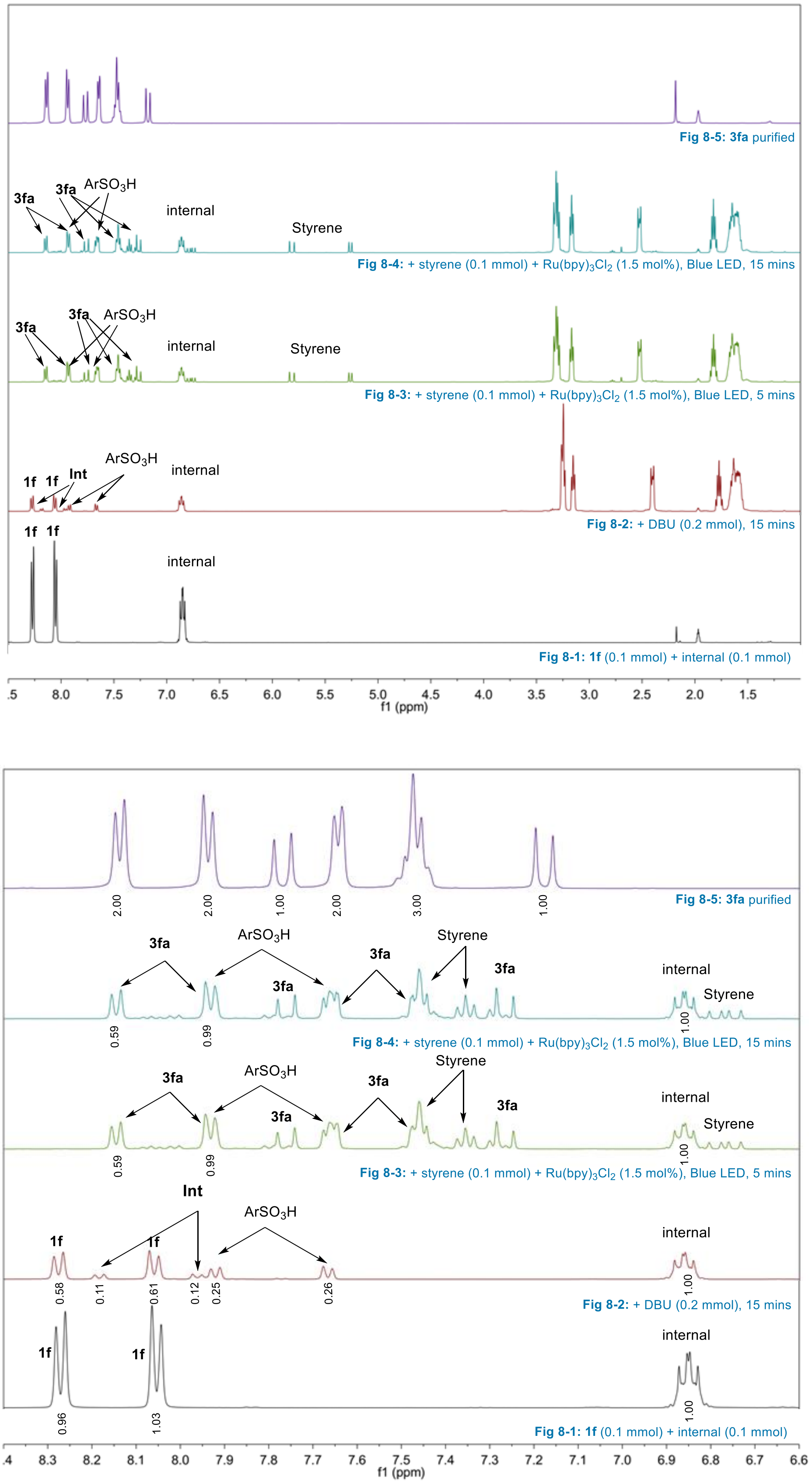

Supplementary Figure 8. ^{1}H NMR monitoring of the reaction of **1f** and styrene in CD_3CN (top: δ 8.5 – 1.0 ppm; bottom: δ 8.4 – 6.6 ppm)

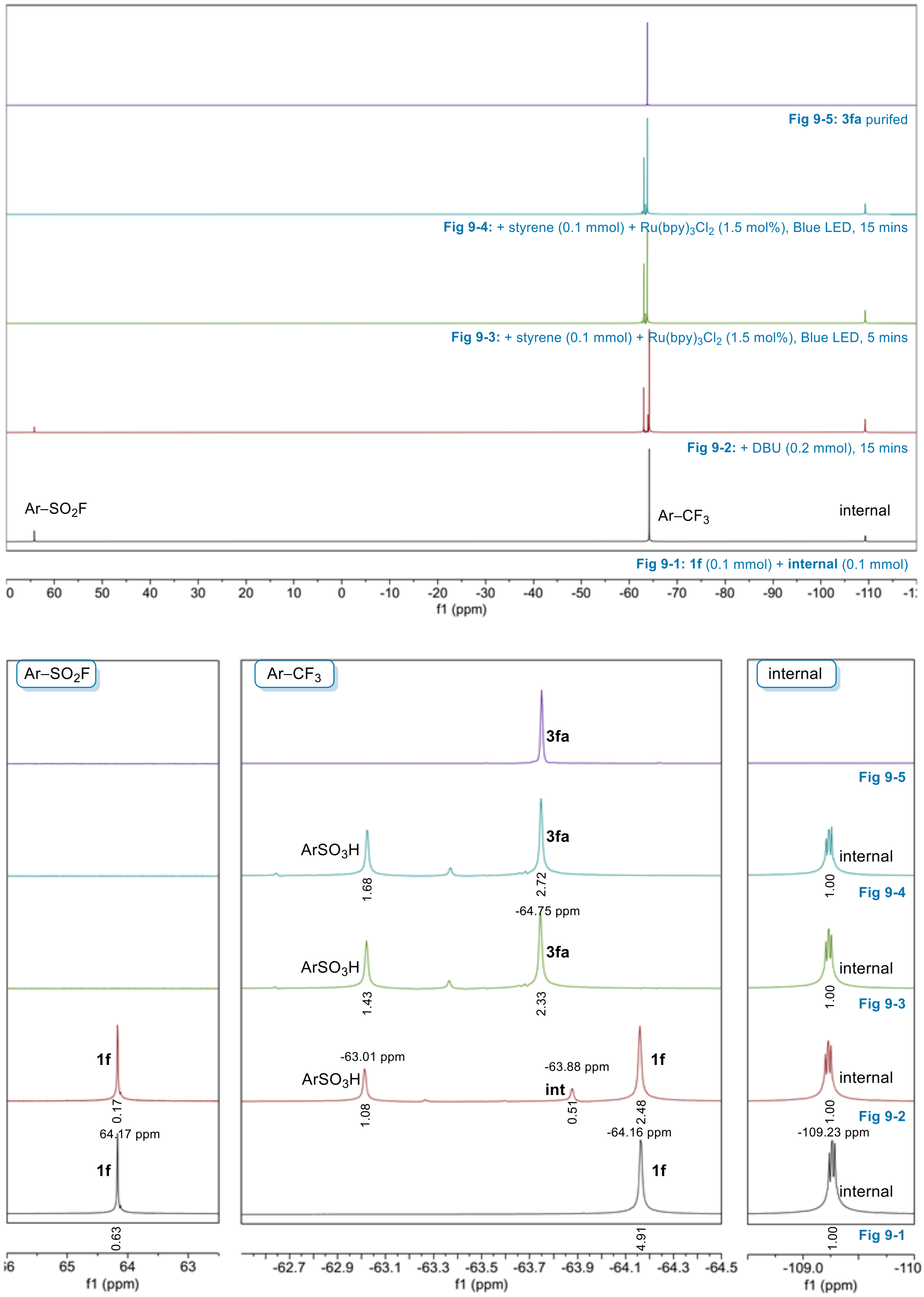

Supplementary Figure 9. ^{19}F NMR monitoring of the reaction of **1f** and styrene in CD_3CN (top: δ 70.0 – -120 ppm; bottom: left δ 66.0 – 63.5 ppm, middle δ -62.5 – -64.5 ppm, right δ -108.5 – -110.0 ppm)

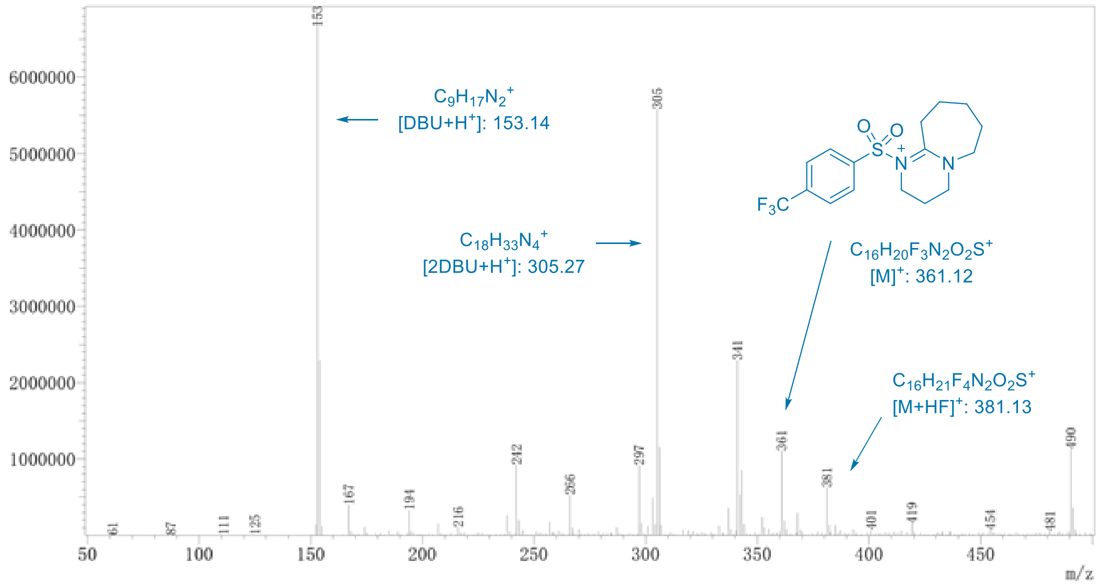

Supplementary Figure 10. MS (ESI) monitoring of the reaction mixture of **1f** and DBU in MeCN.

5.6.2 The reaction of **1f** and styrene in DMSO-d_6

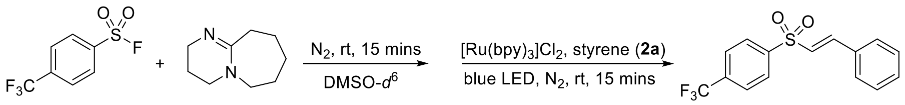

Under the N_2 atmosphere, the NMR spectra of sulfonyl fluoride (**1f**, 0.10 mmol, 1.0 eq.) and 1,3,5-trifluorobenzene (internal standard. 0.10 mmol, 1.0 eq.) in DMSO-d_6 (0.6 mL) were recorded (**Supplementary Figure 11-1**, **12-1**). DBU (0.20 mmol, 2.0 eq.) was then added into the NMR tube. The resulting mixture was shaken for 15 min, and then another group of NMR spectra were collected (**Supplementary Figure 11-2**, **12-2**). Thereafter, the photocatalyst Ru(bpy)$_3$Cl$_2$ (1.5 mol%) and styrene (**2a**, 0.10 mmol, 1.0 eq.) were added into the NMR tube. The resulting mixture was shaken under blue LED irradiation (5 W). The NMR spectra were collected 5 mins later, and the full consumption of sulfonyl fluoride and **Int** were observed (**Supplementary Figure 11-3**, **12-3**). No further change was observed after 15 mins of reaction time (**Supplementary Figure 11-4**, **12-4**). The formation product **3fa** was confirmed by standard sample (**Supplementary Figure 11-5**).

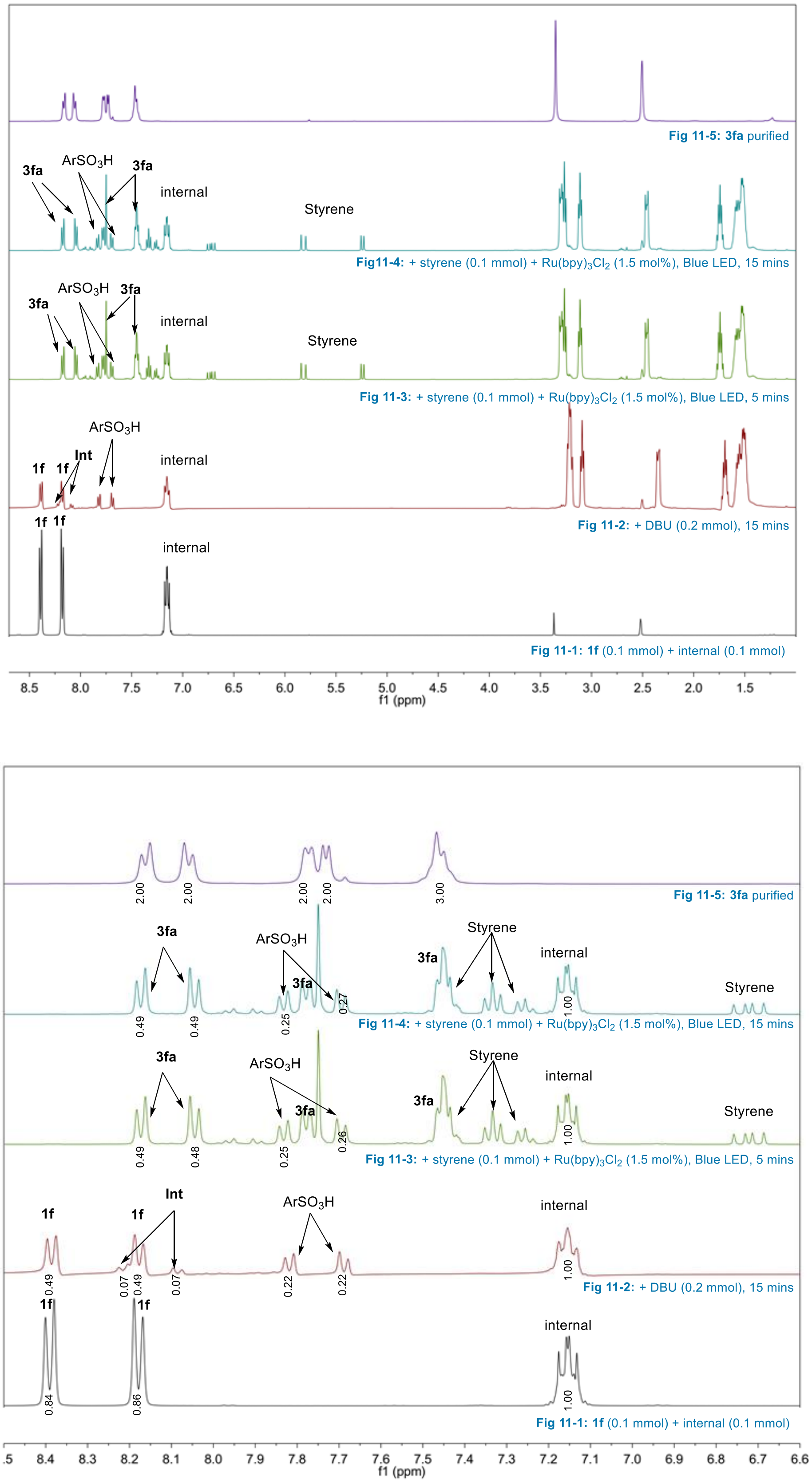

Supplementary Figure 11. ^{1}H NMR monitoring of the reaction of **1f** and styrene in DMSO-d^6 (top: δ 8.7 – 1.0 ppm; bottom: δ 8.5 – 6.6 ppm)

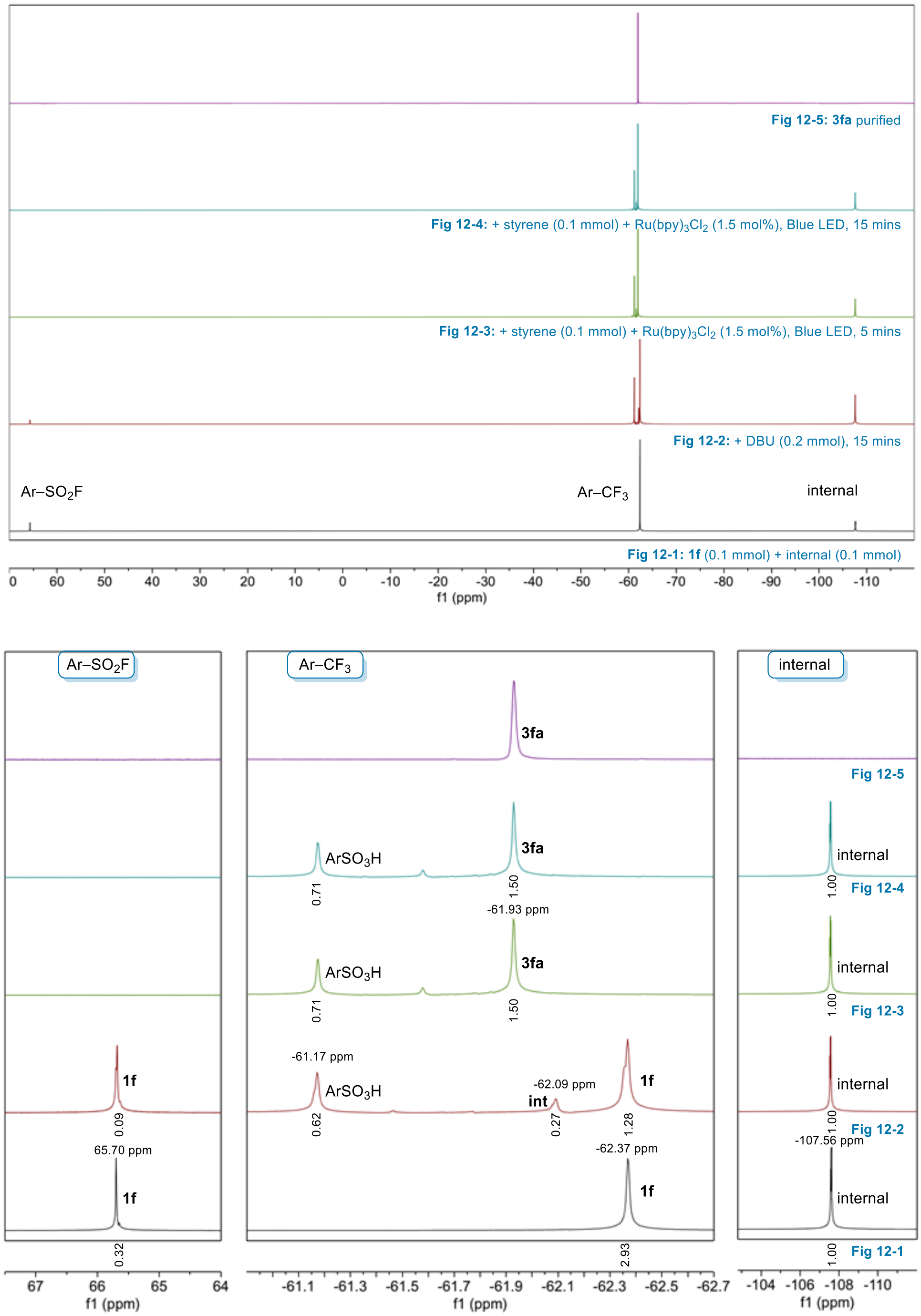

Supplementary Figure 12. ^{19}F NMR monitoring the reaction of **1f** and styrene in DMSO-d^6 (top: δ 70.0 – -120 ppm; bottom: left δ 66.0 – 64.0 ppm, middle δ -60.9 – -62.7 ppm, right δ -103.0 – -112.0 ppm)

5.6.3 study the potential hydrogen bonding of substrate $ArSO_2F$

Table: A brief summary of the NMR experiments for hydrogen bonding study.

entry	NMR experiment	Spectra location	aim & conclusion
1	DBU : **1f** = 2 : 1 (-40 °C →20 °C, in CD_3CN)	Supplementary Figture13, 14 (Page 73-74)	**Aim**: to probe potential hydrogen bonding from -40 °C to 20 °C.<br>**Conclusion**:<br>1) no hydrogen bonding with the $ArSO_2F$ was found.<br>2) $[FHF]^-$ species was found below -20 °C, which was not observable at 20 °C (^{19}F NMR: δ -146.16 ppm, d, J = 124 Hz; ^1H NMR: δ 16.01 ppm, t, J = 128 Hz) [1]
2	DBU : TfOH : **1d** = 1 : 1 : 1 (20 °C→ -40 °C →20 °C, in CD_3CN)	Supplementary Figure 15, 16 (Page 75-76)	**Aim**: (DBU and TfOH are premixed to intentionally prepare a $DBU•H^+$ species), and then to study if it has any hydrogen bonding with the $ArSO_2F$.<br>**Conclusion**: no $[S–F–H]^+$ bonding, but possible $[S=O–H]^+$ bonding with the $ArSO_2F$.
3	DBU : TfOH : **1d** = 1 : 1 : 1 (20 °C→ -60 °C →20 °C, in $CDCl_3$)	Supplementary Figure 17, 18 (Page 77-78)	**Aim**: to rule out the solvent effect and temperature effect in studying the hydrogen bonding of $ArSO_2F$.<br>(CD_3CN solvent might be a better hydrogen bond acceptor than $ArSO_2F$ in previous NMR studies, making the above conclusion inaccurate. A weak coordinating solvent $CDCl_3$ was used in this experiment. And the study was performed at a lower temperature)<br>**Conclusion**: no $[S–F–H]^+$ bonding, but possible $[S=O–H]^+$ bonding with the $ArSO_2F$.
4	TfOH : **1d** = 1 : 1 (20 °C→ -60 °C →20 °C, in $CDCl_3$)	Supplementary Figure 19, 20 (Page 79-80)	**Aim**: to detect potential hydrogen bond by simply mixing the acid with $ArSO_2F$ in $CDCl_3$.<br>**Conclusion**: no $[S–F–H]^+$ bonding, but possible $[S=O–H]^+$ bonding with the $ArSO_2F$.

1f was *p*-$CF_3C_6H_4SO_2F$, and **1d** was $PhSO_2F$.

[1] *J. Fluorine Chem.* **2016**, *192*, 141-146.

5.6.3.1 NMR study of the reaction mixture of (DBU (0.2 mmol) + **1f** (0.1 mmol)) in CD_3CN

Under the N_2 atmosphere, the NMR spectra of sulfonyl fluoride (**1f**, 0.10 mmol, 1.0 eq.) and 1,3,5-trifluorobenzene (internal standard, 0.10 mmol, 1.0 eq.) in CD_3CN (0.6 mL) were recorded at -40 °C (**Supplementary Figure 13-1**, **14-1**). Subsequently, DBU was added at 20 °C and the NMR temperature was lower to -40 °C for 1 h to record another group of NMR spectra (**Supplementary Figure 13-2**, **14-2**). The NMR temperature was gradually warm up from -40 °C to 20 °C, and the spectra were recorded at different temperatures (**Supplementary Figure 13-3 - 13-6**, **Supplementary Figure 14-3 - 14-6**).

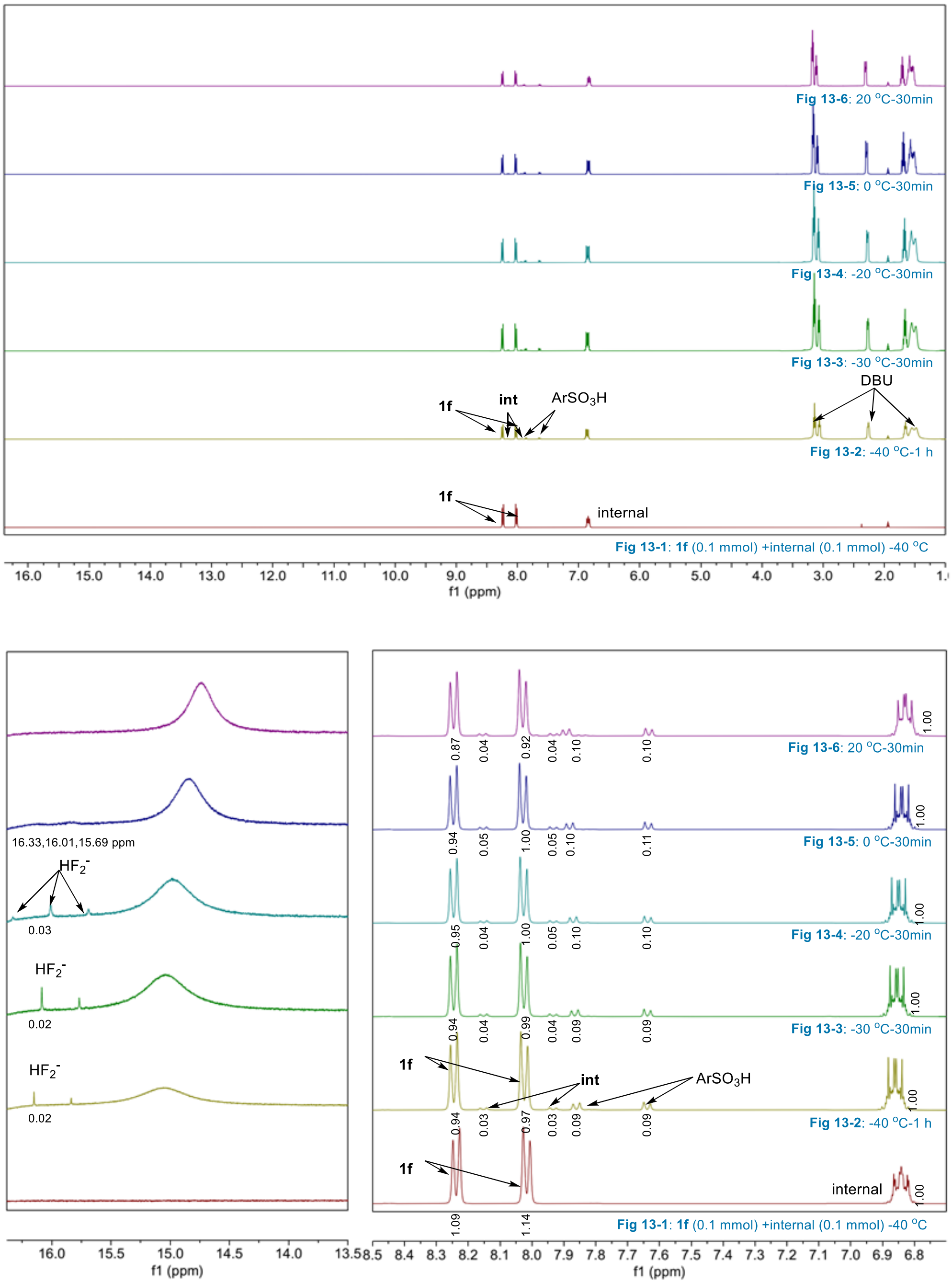

Supplementary Figure 13. ^1H NMR monitoring of the (**1f** (0.1 mmol) + DBU (0.2 mmol)) reaction mixture in CD_3CN (top: δ 16.4 – 1.0 ppm; bottom left δ 16.4 – 13.5 ppm, right δ 8.5 – 6.7 ppm)

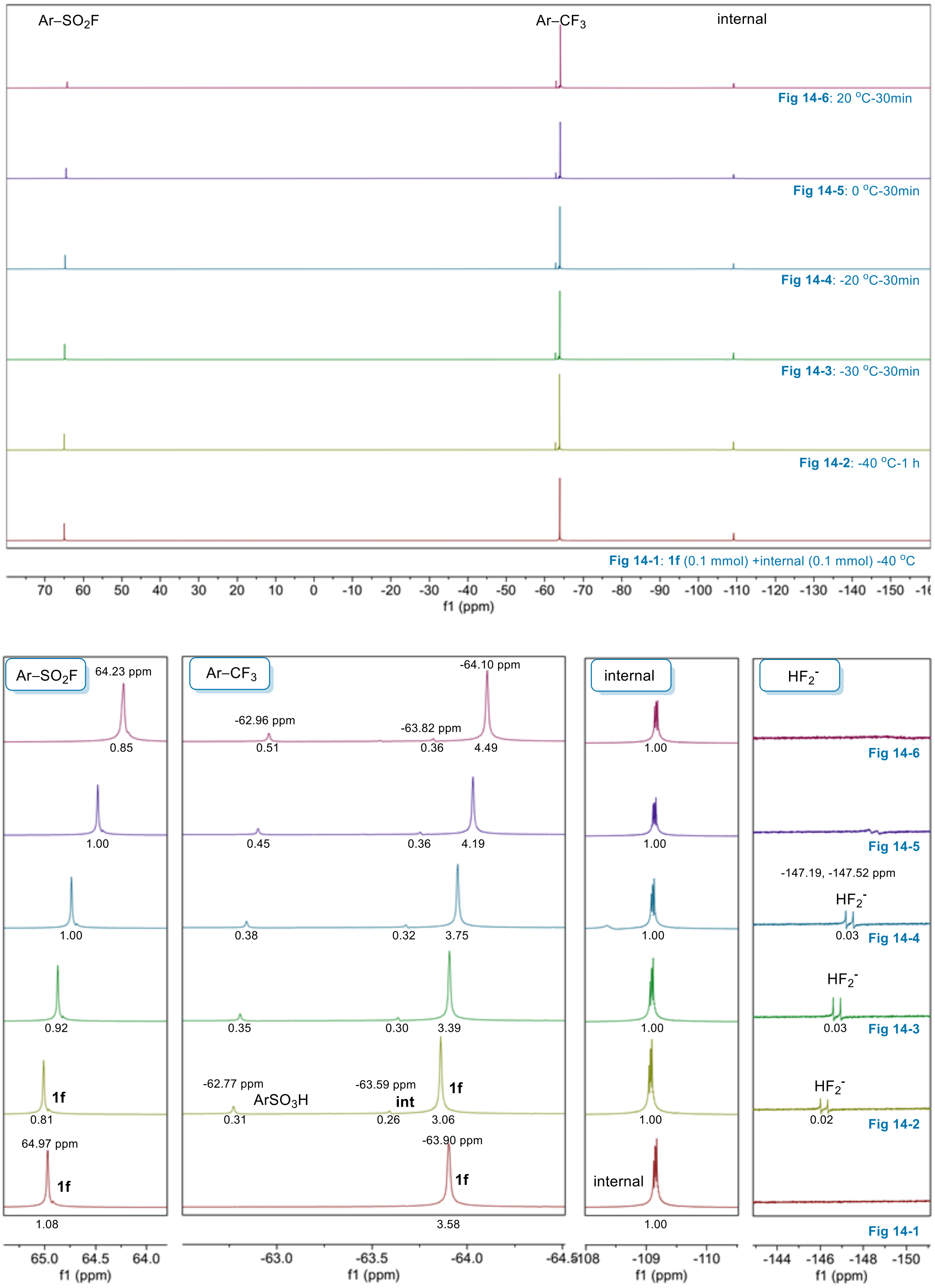

Supplementary Figure 14. ^{19}F NMR monitoring of the (**1f** (0.1 mmol) + DBU (0.2 mmol)) reaction mixture in CD_3CN (top: δ 80.0 – -160.0 ppm; bottom left δ 65.5 – 67.7 ppm, middle 1 δ -62.5 – 64.5 ppm, middle 2 δ -108 – -110.5 ppm, right δ -143– -151.0 ppm.)

5.6.3.2 NMR study of mixture of (DBU (0.1 mmol) + TfOH (0.1 mmol) + **1d** (0.1 mmol)) in CD_3CN (20 ºC→ –40 ºC → 20 ºC)

Under the N_2 atmosphere, phenylsulfonyl fluoride (**1d**, 0.10 mmol, 1.0 eq.) was added to the mixture of DBU (**1d**, 0.10 mmol, 1.0 eq.), TfOH (**1d**, 0.10 mmol, 1.0 eq.), and 1,3,5-trifluorobenzene (internal standard, 0.10 mmol, 1.0 eq.) in CD_3CN (0.6 mL). After that, the NMR spectra were recorded at 20 ºC **Supplementary Figure 15-1**, **16-1**). The NMR temperature was gradually cooled down from 20 °C to -40 °C, and then was warmed up to 20 °C. The related NMR spectra were recorded at different temperatures (**Supplementary Figure 15-2 - 15-7**, **Supplementary Figure 16-2 - 16-7**).

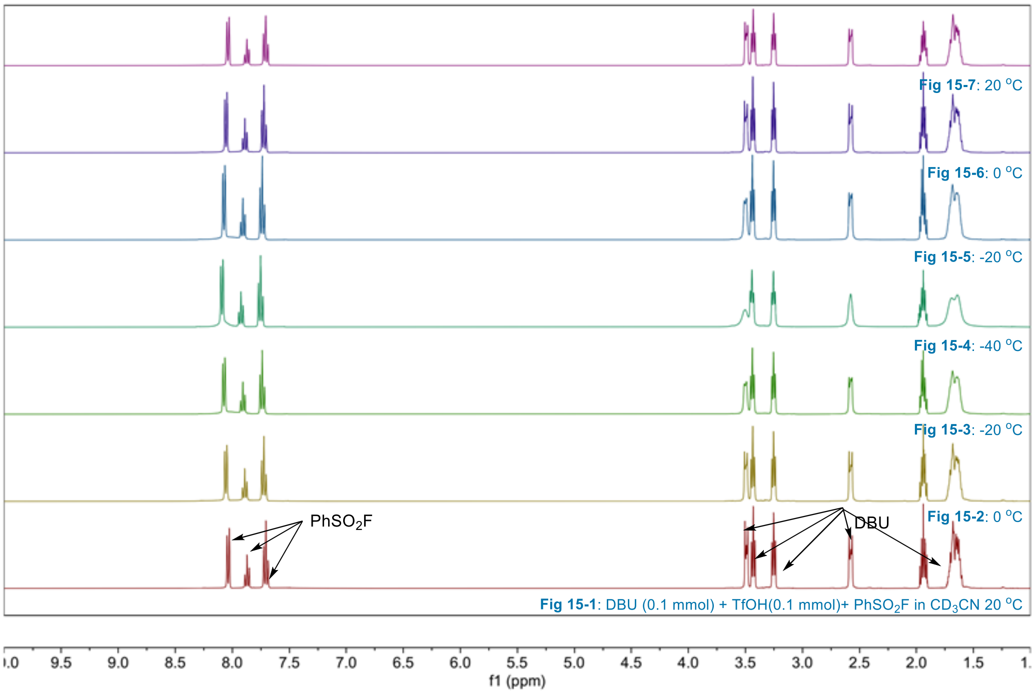

Supplementary Figure 15. ^{1}H NMR monitoring of the potential hydrogen bonding between DBU•H^+ and $PhSO_2F$ in CD_3CN (DBU (0.1 mmol) + TfOH (0.1 mmol) + **1d** (0.1 mmol)) (δ 9.0 – 1.0 ppm)

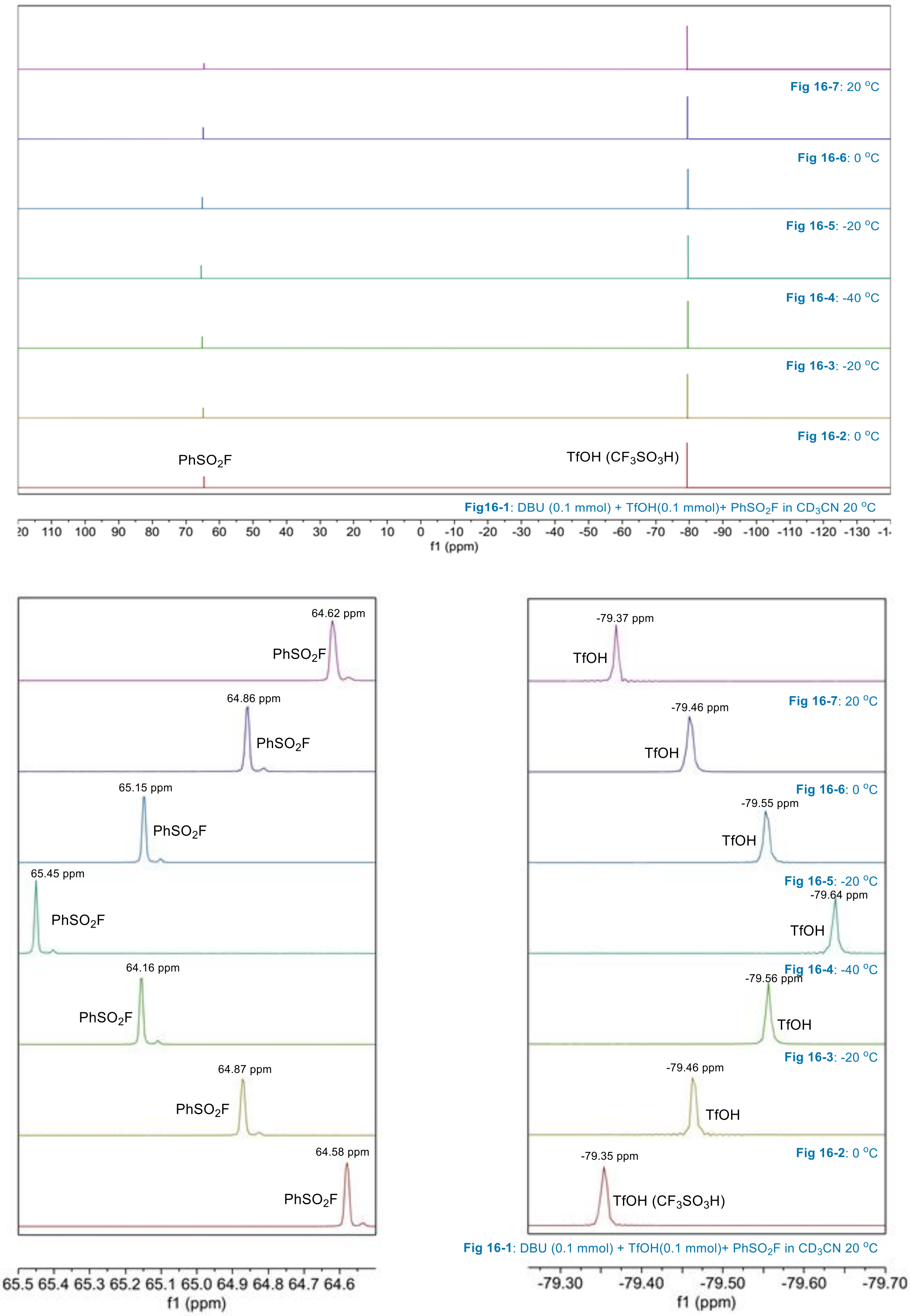

Supplementary Figure 16. ^{19}F NMR monitoring of the potential hydrogen bonding between DBU•H$^+$ and $PhSO_2F$ in CD_3CN (DBU (0.1 mmol) + TfOH (0.1 mmol) + **1d** (0.1 mmol)) (top: δ 120 – -140 ppm; bottom: left δ 65.5 – 64.5 ppm, right δ -79.3– -79.7 ppm.)

(Our analysis and conclusion: The ^{19}F chemical shift changes of -SO_2F and CF_3SO_2- at different temperatures suggests possible [S=O–H]$^+$ bonding with the $ArSO_2F$. The [S–F–H]$^+$ bonding was ruled out because that the split of -SO_2F signal by a proton was not observed.)

5.6.3.3 NMR study of the mixture of (DBU (0.1 mmol) + TfOH (0.1 mmol) + **1d** (0.1 mmol)) in $CDCl_3$ (20 ºC→ -60 ºC → 20 ºC).

Under N_2 atmosphere, sulfonyl fluoride (**1d**, 0.10 mmol, 1.0 eq.) was added to the mixture of DBU (**1d**, 0.10 mmol, 1.0 eq.), TfOH (**1d**, 0.10 mmol, 1.0 eq.), and 1,3,5-trifluorobenzene (internal standard, 0.10 mmol, 1.0 eq.) in $CDCl_3$ (0.6 mL). After that, the NMR spectra were recorded at 20 ºC (**Supplementary Figure 17-1**, **18-1**). The NMR temperature was gradually cooled down from 20 °C to -60 °C, and then was warmed up to 20 °C. The related NMR spectra were recorded at different temperatures (**Supplementary Figure 17-2 - 18-9**, **Supplementary Figure 17-2 - 18-9**).

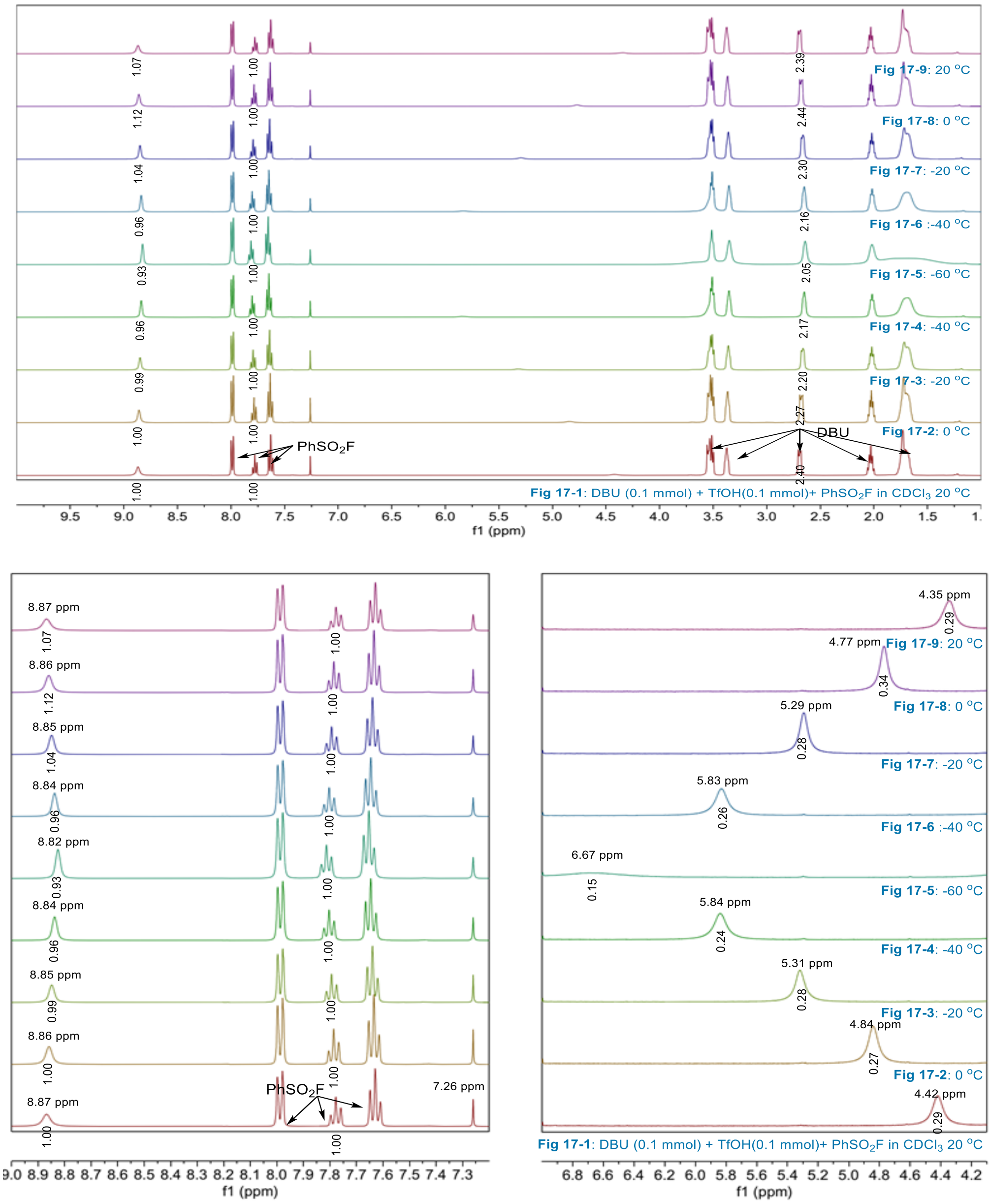

Supplementary Figure 17. ^{1}H NMR monitoring of the potential hydrogen bonding between DBU•H$^+$ and $PhSO_2F$ in $CDCl_3$ (DBU (0.1 mmol) + TfOH (0.1 mmol) + **1d** (0.1 mmol)) (top: δ 10.0 – 1.0 ppm; bottom: left δ 9.0 – 7.2 ppm, right δ 6.7 – 4.2 ppm.)

(Our analysis and conclusion: The ^{1}H chemical shift changes of an active proton species (6.8 ppm – 4.2 ppm) at different temperatures suggests possible [S=O–H]$^+$ bonding with $ArSO_2F$. The [S–F–H]$^+$ bonding was ruled out because that the split of this proton peak by a fluorine was not observed)

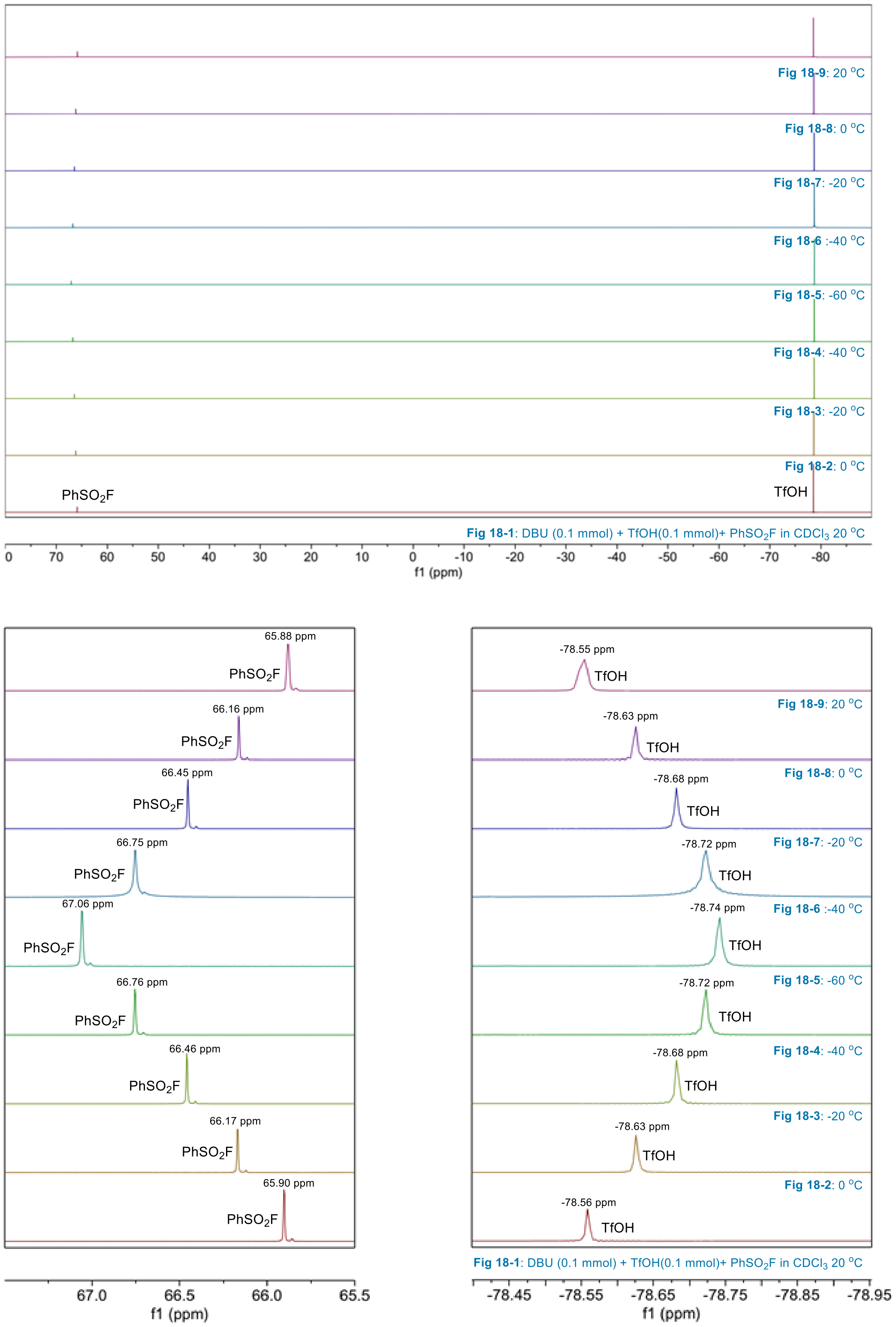

Supplementary Figure 18. ^{19}F NMR monitoring of the potential hydrogen bonding between DBU•H$^+$ and $PhSO_2F$ in $CDCl_3$ (DBU (0.1 mmol) + TfOH (0.1 mmol) + 1d (0.1 mmol)) (top: δ 80.0 – -90.0 ppm; bottom: left δ 67.5 – 65.5 ppm, right δ -78.4– -78.9 ppm.)

(Our analysis and conclusion: The ^{19}F chemical shift changes of $–SO_2F$ and CF_3SO_3H at different temperatures suggests possible $[S{=}O–H]^+$ bonding with the $ArSO_2F$. The $[S–F–H]^+$ bonding was ruled out because that the split of the $–SO_2F$ signal by a proton was not observed)

5.6.3.4 NMR study of the mixture of (TfOH (0.1 mmol) + **1d** (0.1 mmol)) in $CDCl_3$ (20 ºC→ -60 ºC → 20 ºC)

Under N_2 atmosphere, the NMR spectra of sulfonyl fluoride (**1d**, 0.10 mmol, 1.0 eq.), TfOH (0.10 mmol, 1.0 eq.), and 1,3,5-trifluorobenzene (internal standard, 0.10 mmol, 1.0 eq.) in $CDCl_3$ were recorded at 20 ºC (**Supplementary Figure 19-1**, **20-1**). The NMR temperature was gradually cooled down from 20 °C to -60 °C, and then was warmed up to 20 °C. The related NMR spectra were recorded at different temperatures (**Supplementary Figure 19-2 - 20-9**, **Supplementary Figure 19-2 - 20-9**).

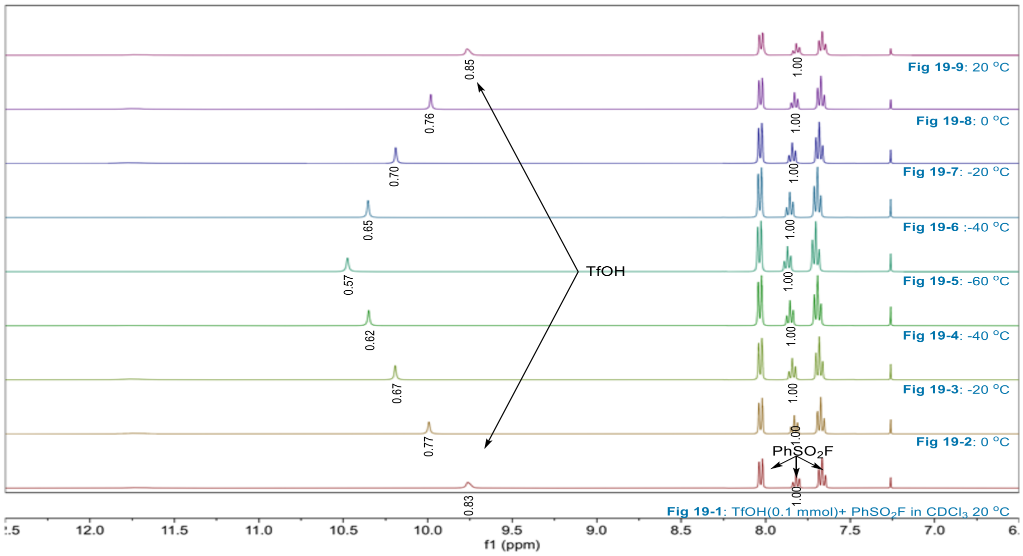

Supplementary Figure 19. ^1H NMR monitoring of the hydrogen bonding of TfOH and $PhSO_2F$ in $CDCl_3$ (**1d** (0.1 mmol) + TfOH (0.1 mmol).

(Our analysis and conclusion: The ^1H chemical shift changes of an active proton species (10.7 ppm – 9.5 ppm) at different temperatures suggests possible $[S{=}O{-}H]^+$ bonding with the $ArSO_2F$. The $[S{-}F{-}H]^+$ bonding was ruled out because that the split of this proton peak by a fluorine was not observed)

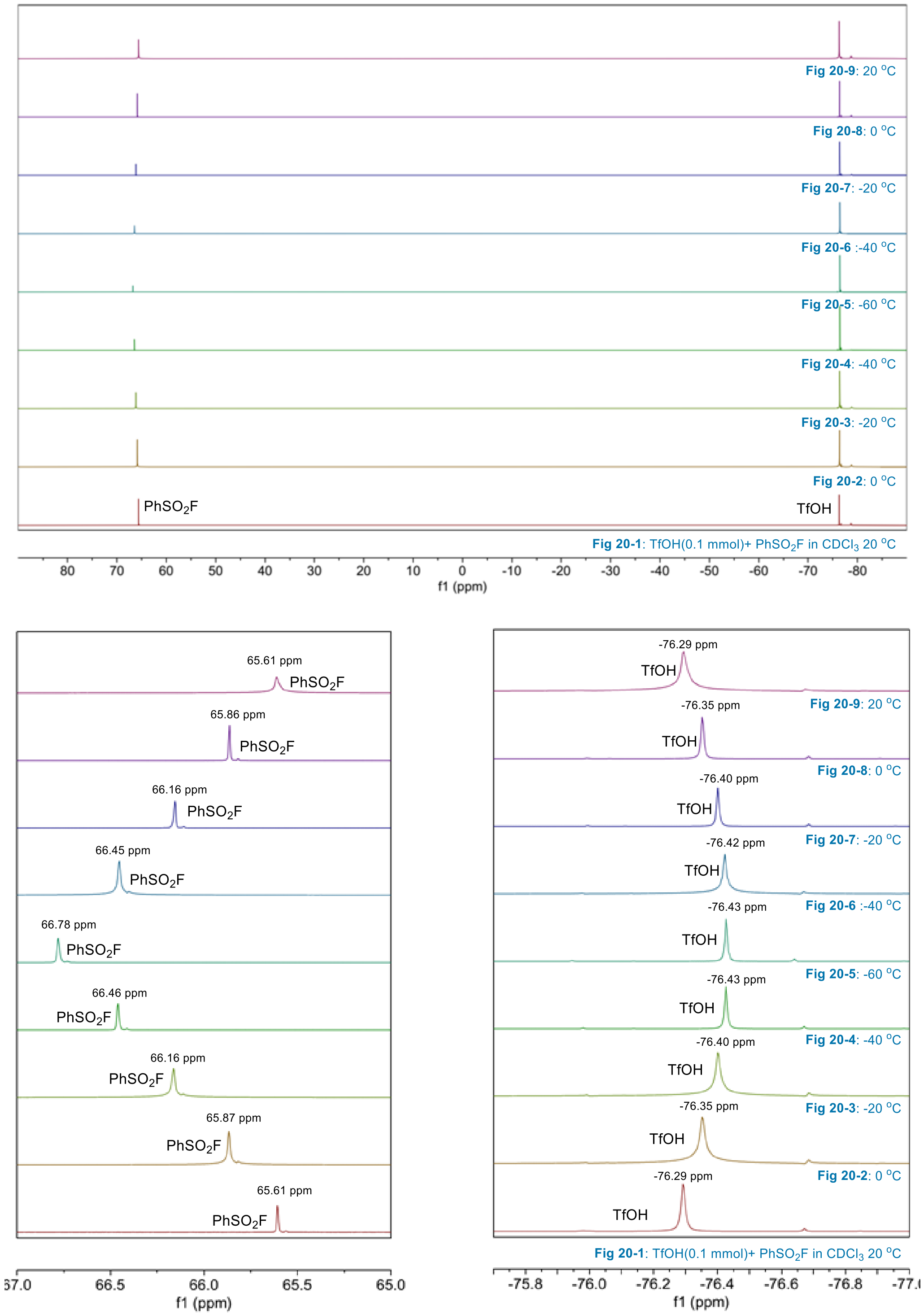

Supplementary Figure 20. ^{19}F NMR monitoring of the hydrogen bonding of TfOH and $PhSO_2F$ in $CDCl_3$ (**1d** (0.1 mmol) + TfOH (0.1 mmol)). (top: δ 90.0 – -90.0 ppm; bottom: left δ 67.0 – 65.0 ppm, right δ -75.7 – -77.0 ppm.)

(Our analysis and conclusion: the ^{19}F chemical shift changes of $–SO_2F$ and CF_3SO_3H at different temperatures suggests possible $[S{=}O–H]^+$ bonding with the $ArSO_2F$. The $[S–F–H]^+$ bonding was ruled out because that the split of the $–SO_2F$ signal by a proton was not observed)

5.6.4 The accumulation of **Int** species?

Under the N_2 atmosphere, the solution of sulfonyl fluoride (**1f**, 0.10 mmol, 1.0 eq.), DBU (0.20 mmol, 2.0 eq.) and 1,3,5-trifluorobenzene (internal standard. 0.10 mmol, 1.0 eq.) in CD_3CN (0.6 mL) were incubated at different temperature for different times, respectively (room temperature for 5 h, 0 ºC for 8 h, -20 ºC for 8 h), -40 ºC for 8 h). Then, NMR spectra were recorded at the corresponding NMR temperatures.

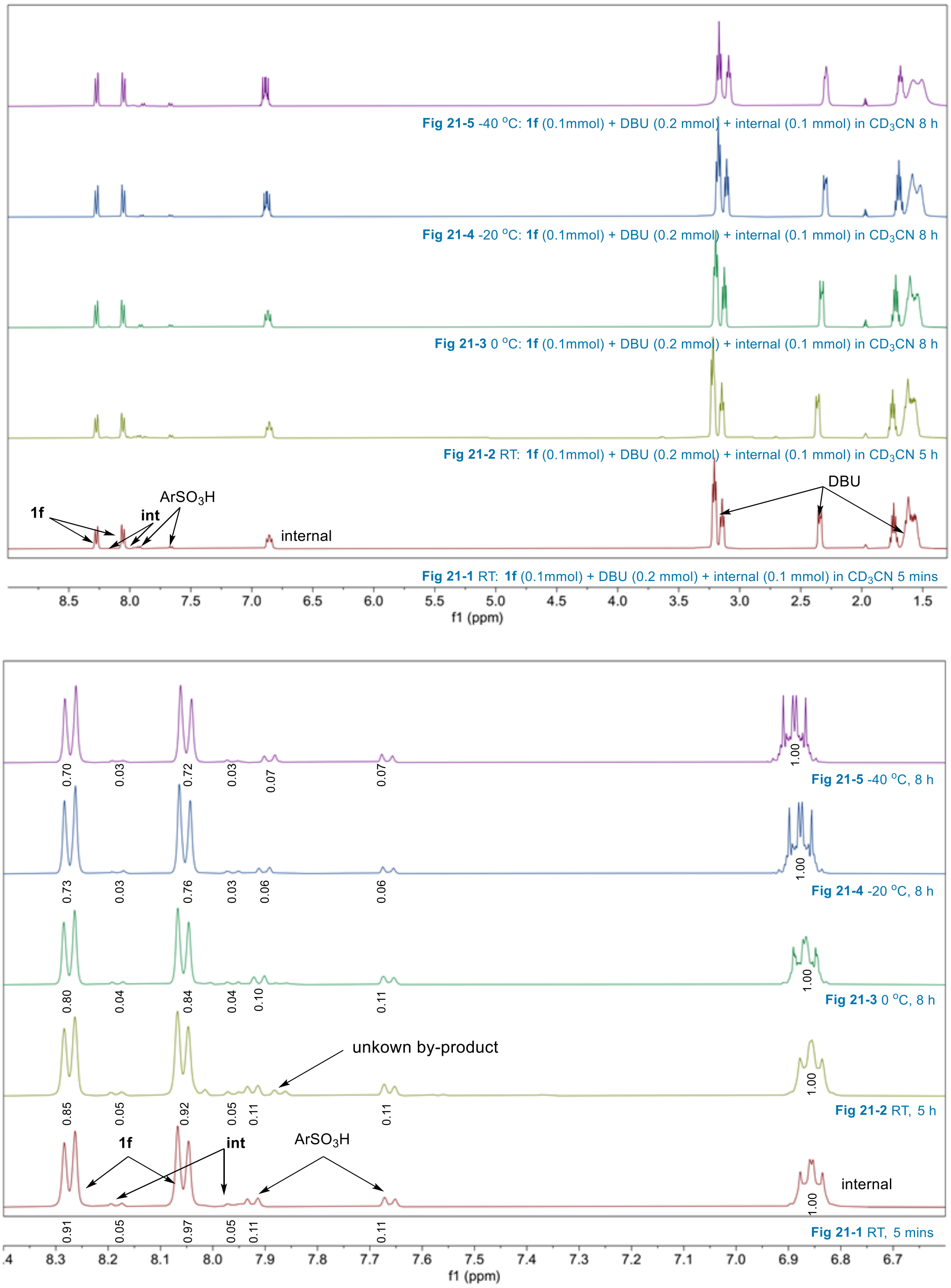

Supplementary Figure 21. ^{1}H NMR monitoring of the reaction mixture of (**1f** (0.1 mmol) + DBU (0.2 mmol)) in CD_3CN (top: δ 8.5 – 1.3 ppm; bottom: δ 8.4 – 6.6 ppm)

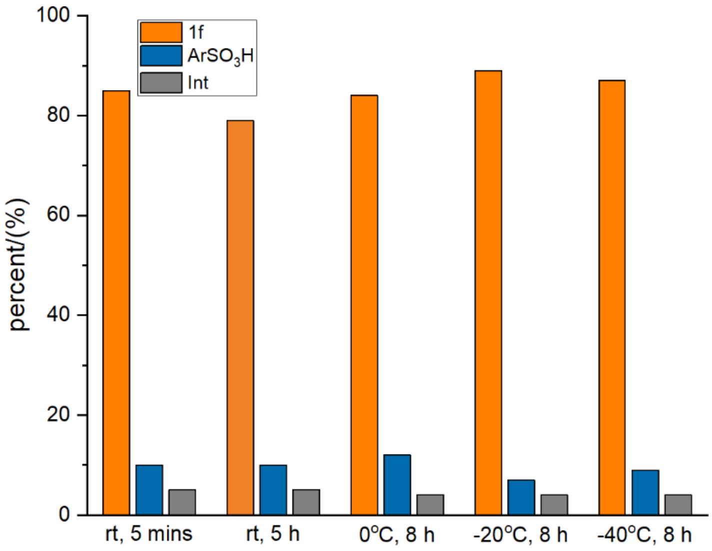

Supplementary Figure 22. Accumulation of **Int** species at different temperatures

analysis and conclusion:

1) the ratio of the **Int** species did not increase with times at room temperature (**Supplementary Figure 21-1**, **21-2**). A by-product was formed after 5 hours of reaction at room temperature. We could not identify its chemical structure (**Supplementary Figure 21-2**).

2) yields of the **Int** species were relatively lower at low reaction temperatures (**Supplementary Figure 21-3** to **Supplementary Figure 21-5**). And we did not find the by-product at low reaction temperatures even with longer reaction time, which was observed at room temperature.

5.6.5 Study the ArSO[=NR][DBU]$^+$ species in the reaction of sulfoximidoyl fluoride substrates

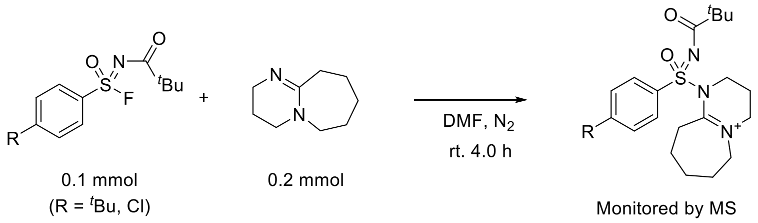

To a 10 mL Schlenk tube equipped with a magnetic stirring bar was charged sulfonyl fluoride (1.0 eq. 0.1 mmol) and DBU (2.0 eq. 0.2 mmol) under N_2 atmosphere. Subsequently, dry DMF (0.10 M) was added. After 4 h of reaction, the resulting mixture was monitored by MS (ESI, Shimadzu).

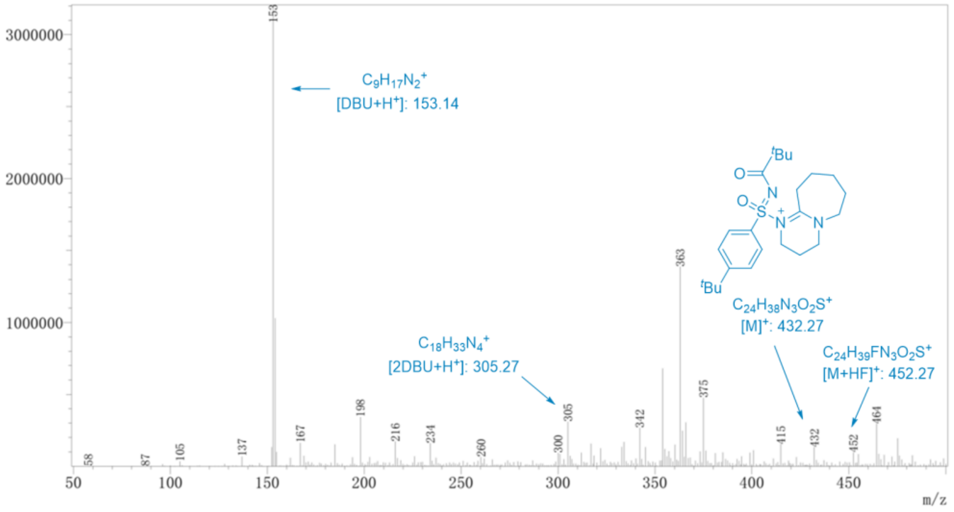

Supplementary Figure 23. MS (ESI) monitoring of the mixture of **7s** and DBU in DMF.

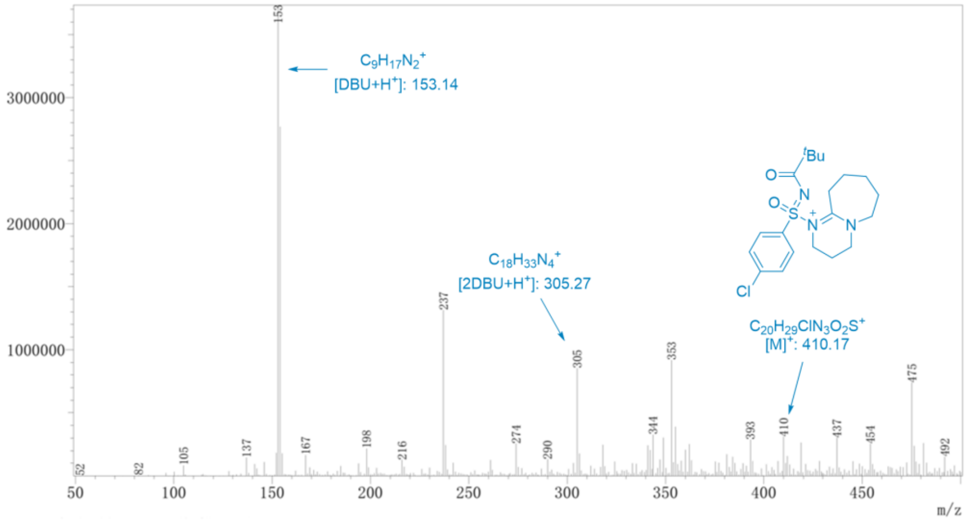

Supplementary Figure 24. MS (ESI) monitoring of the mixture of **7x** and DBU in DMF.

5.7 Stern-Volmer fluorescence quenching study

Visible light luminescence intensities were recorded on Hitachi F-7000 FL Spectrophotometer spectrophotometer. All luminescence were recorded using a fluorescence glass cuvette (10 mm, 3.5 mL). The quenching study of [Ru(bpy)$_3$]Cl$_2$ was performed at different concentrations in MeCN. Under the nitrogen atmosphere, a solution of [Ru(bpy)$_3$]Cl$_2$ (0.005 mM) was treated with sulfonyl fluoride **1a** (or **1f**, 0.01-100 mM), alkene **2a** (0.01-100 mM), or DBU (0.01-100 mM) in anhydrous MeCN at different concentrations. The samples were excited at 450 nm and the emission were measured from 470 to 800 nm.

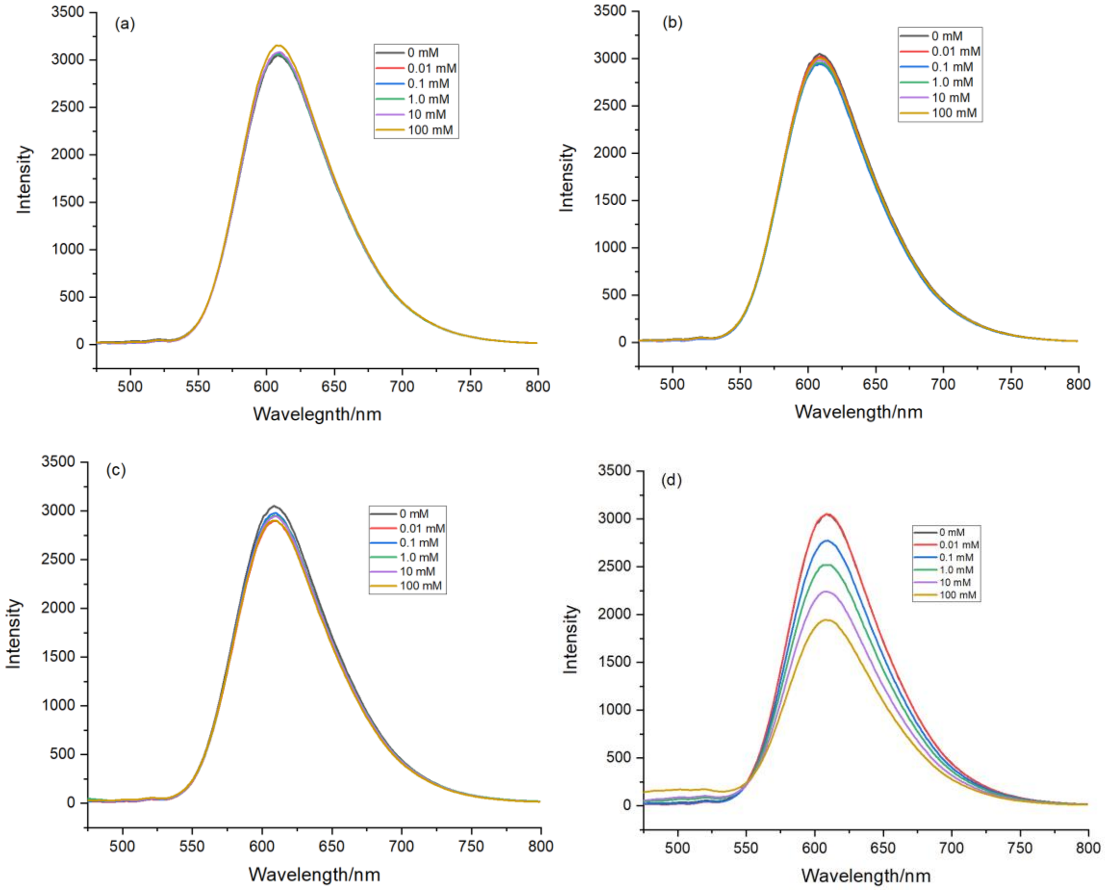

Supplementary Figure 25. [Ru(bpy)$_3$]Cl$_2$ emission quenching respectively by (a) **1a**, (b) **1f**, (c) **2a**, (d) DBU

5.8 Cyclic voltammetry studies[42]

Cyclic voltammograms were collected with a CS2350H-Bipotentiostat. Samples were prepared with 0.01 mmol of substrate in 10 mL of 0.10 M tetra-*n*-butylammonium hexafluorophosphate ($TBAPF_6$) in dry, degassed acetonitrile. Measurements employed a glassy carbon working electrode, platinum wire counter electrode, 3.5 M KCl silver-silver chloride reference electrode, and a scan rate of 100 mV/s. Reductions were measured by scanning potentials in the negative direction and oxidations in the positive direction.

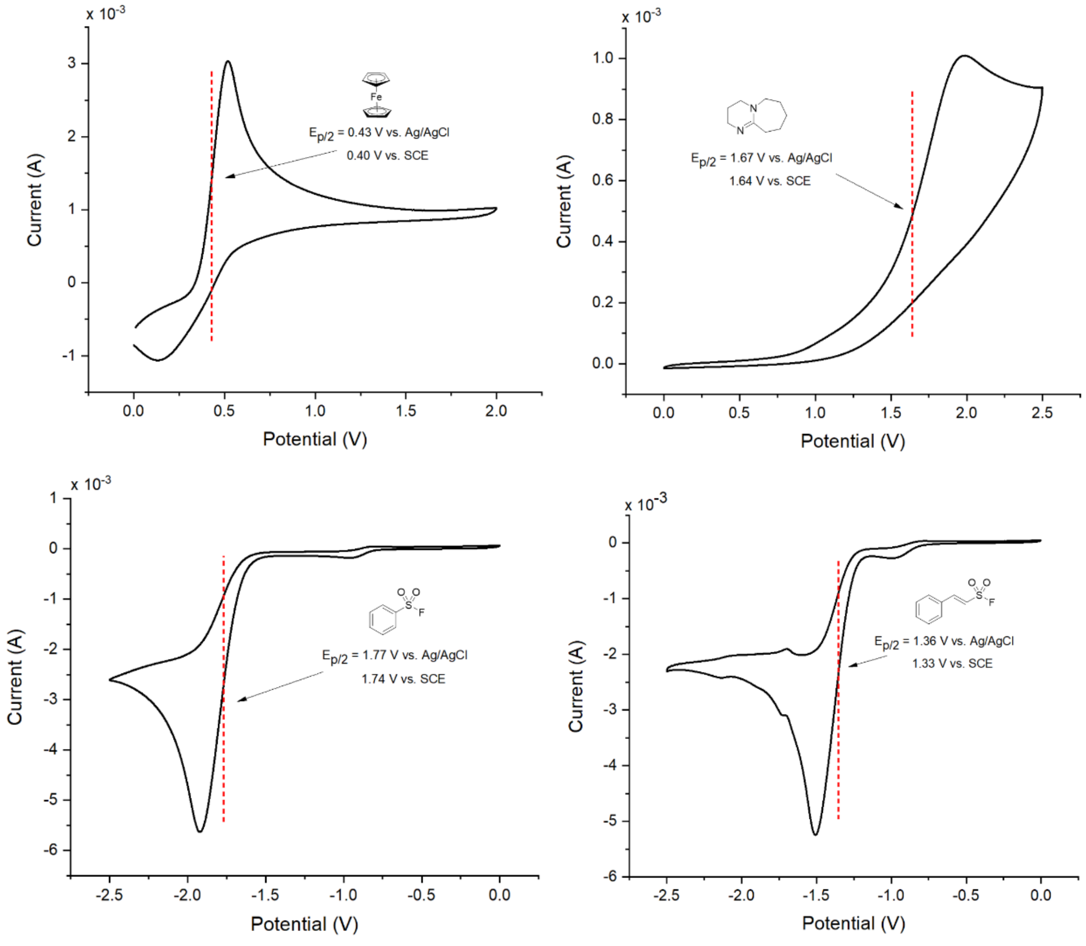

Supplementary Figure 26. Cyclic voltammogram (CV)

Section 6. Competitive reaction and products derivatizations

6.1 Competition of the photoredox and nucleophilic substitution reactions

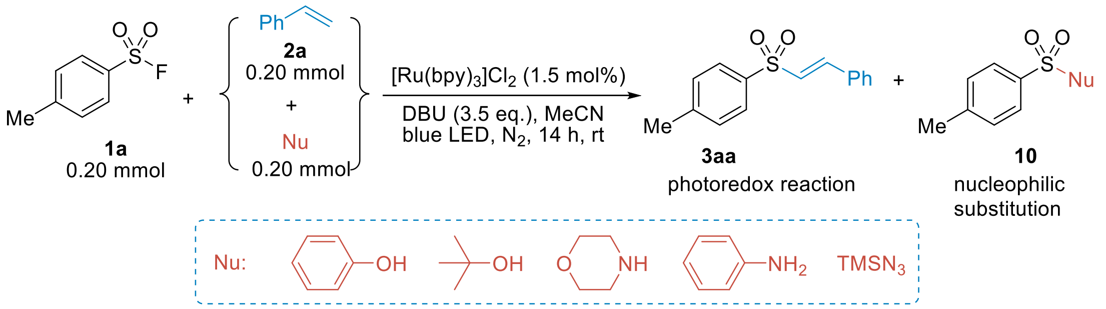

The competition experiments were carried out by following the procedure. To a 10 mL Schlenk tube equipped with a magnetic stirring bar was charged **1a** (0.20 mmol, 1.0 eq.) and $[Ru(bpy)_3]Cl_2$ (0.003 mmol, 1.5 mol%) under N_2 atmosphere. Subsequently, dry MeCN (2.0 mL), **2a** (0.20 mmol, 1.0 eq.), one of the above nucleophiles (0.20 mmol, 1.0 eq.), and DBU (0.70 mmol, 3.5 eq.) were added. The reaction mixture was illuminated with blue LED and stirred at room temperature for 14 h. The yield of nucleophilic substitution products or photoredox reaction products were recorded by ^1H NMR with CH_2Br_2 as the internal standard.

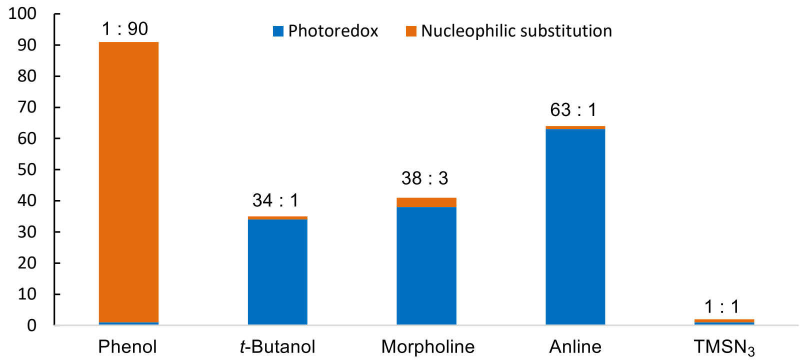

Supplementary Figure 27. Analysis of the competition experiments

6.2 Grams scale synthesis

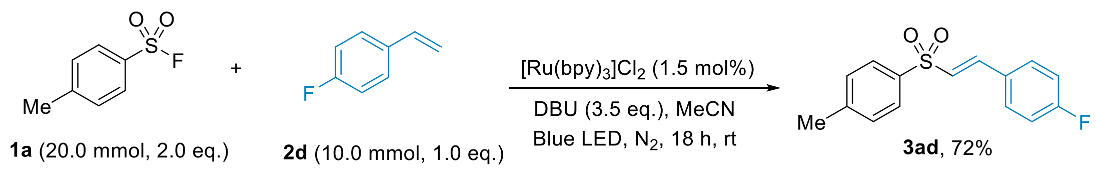

Under N_2 atmosphere, to a 100 mL Schlenk tube equipped with a magnetic stirring bar was charged sulfonyl fluoride (20.0 mmol, 2.0 eq.), $[Ru(bpy)_3]Cl_2$ (0.15 mmol, 1.5 mol%), dry MeCN (60 mL), olefin (10.0 mmol, 1.0 eq.), and DBU (35.0 mmol, 3.5 eq.). The mixture was illuminated with blue LED and stirred at room temperature for 14 h. After that, the residue was purified by silica gel chromatography to give **3ad** (2.0 g) as a white solid. **m.p.** = 94 – 97 °C; **^{1}H NMR** (400 MHz, $CDCl_3$) δ 7.81 (d, J = 8.3 Hz, 2H), 7.61 (d, J = 15.4 Hz, 1H), 7.48 – 7.44 (m, 2H), 7.33 (d, J = 8.0 Hz, 2H), 7.06 (t, J = 8.6 Hz, 2H),

6.79 (d, J = 15.4 Hz, 1H), 2.42 (s, 3H); ^{13}**C NMR** (100 MHz, $CDCl_3$) δ 164.4 (d, J = 251.0 Hz), 144.5, 140.7, 137.8, 130.6 (d, J = 9.0 Hz), 130.1, 128.8 (d, J = 3.0 Hz), 127.8, 127.6 (d, J = 2.0 Hz), 116.5 (d, J = 22.0 Hz), 21.7; ^{19}**F NMR** (376 MHz, $CDCl_3$) δ -108.0 (s, 1F); **HRMS** (EI, m/z): calcd. for $C_{15}H_{13}FO_2S$ $[M]^+$: 276.0165, found: 276.0163.

6.3 Synthesis of 11a

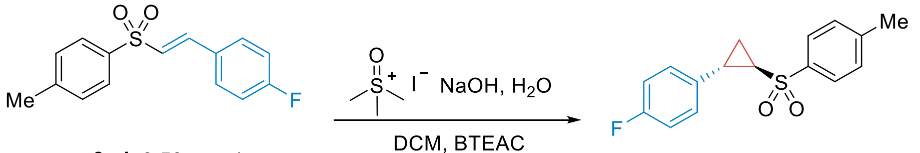

3ad, 0.50 mmol   **11a**, 40%

11a was prepared according to the reported procedure.[43] A mixture of **3ad** (0.50 mmol, 1.0 eq.), trimethylsulfoxonium iodide (0.55 mmol, 1.1 eq.), 50% aq. sodium hydroxide solution (1.25 mL) in DCM (4.0 mL) were stirred till a clear two-phase system was obtained. Then benzyltriethylammonium chloride (BTEAC) (5.0 mg) was added and the reaction mixture was stirred for a period of 2-3 h. Progress of the reaction was monitored by TLC. After completion, it was diluted with water (5.0 mL) and the organic layer was separated, washed with water, brine and dried. After that the solution was concentrated under vacuum, and purified by flash chromatography to afford the product **11a** (48.0 mg, 40%) as a white solid. R_f = 0.34 (Petroleum ether /EtOAc= 10/1); **m.p.** = 109 – 112 °C; ^{1}**H NMR** (400 MHz, $CDCl_3$) δ 7.65 (d, J = 8.1 Hz, 2H), 7.27 – 7.22 (m, 4H), 6.92 (t, J = 8.6 Hz, 2H), 5.52 (s, 1H), 5.17 (s, 1H), 4.21 (s, 2H), 2.39 (s, 3H); ^{13}**C NMR** (100 MHz, $CDCl_3$) δ 162.6 (d, J = 246.0 Hz), 144.8, 135.8, 135.5, 135.1 (d, J = 4.0 Hz), 129.6, 128.7, 128.1 (d, J = 8.0 Hz), 121.8, 115.3 (d, J = 22.0 Hz), 62.4, 21.7; ^{19}**F NMR** (376 MHz, $CDCl_3$) δ -114.1 (s, 1F); **HRMS** (EI, m/z): calcd. for $C_{16}H_{15}FO_2S$ $[M]^+$: 290.0771, found: 290.0769.

6.4 Synthesis of 11b

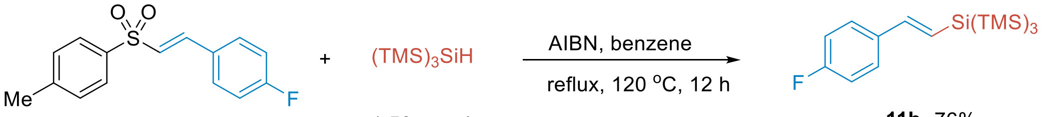

3ad, 0.50 mmol   1.50 mmol   **11b**, 76%

The compound **11b** was synthesized by an analogous procedure described by Xuan jun et al.[44] An oven-dried, two-neck round-bottom-flask equipped with a stirring bar was charged under N_2 atmosphere with **3ad** (0.50 mmol, 1.0 eq.), tris(trimethylsilyl)silane (1.50 mmol, 3.0 eq.), AIBN (1.25 mmol, 2.5 eq.), and dry benzene (5.0 mL) and stirred under reflux for 12 h. After completion, the reaction mixture was concentrated in vacuo and purified by flash column chromatography on silica gel with a mixture of petroleum ether as eluent, affording the final product **11b** (140.0 mg, 76%) as a colorless oil; ^{1}**H NMR** (400 MHz, $CDCl_3$) δ 7.36 – 7.32 (m, 2H), 7.03 – 6.98 (m, 2H), 6.85 (d, J = 18.8 Hz, 1H), 6.37 – 6.32 (m, 1H), 0.22 (s, 27H); ^{13}**C NMR** (100 MHz, $CDCl_3$) δ 162.4 (d, J = 245.0 Hz), 144.2, 135.5, 127.6 (d, J = 8.0 Hz), 122.6, 115.5 (d, J = 21.0 Hz), 1.1; ^{19}**F NMR** (376 MHz, $CDCl_3$) δ -114.8 (s, 1F); **HRMS** (EI, m/z): calcd. for $C_{17}H_{33}FSi_4$ $[M]^+$: 368.1638, found: 368.1639.

6.5 Synthesis of 11c

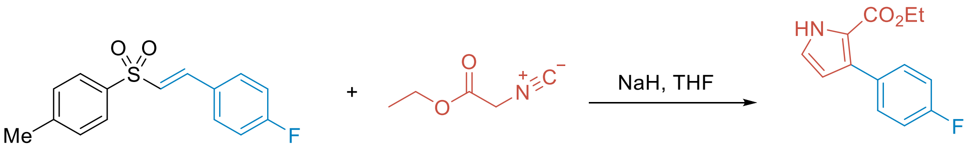

3ad, 0.50 mmol   1.0 mmol   **11c**, 77%

Compound **11c** was prepared by literature method.[31] To a suspension of sodium hydride (60% dispersion in oil, 1.0 mmol, 2.0 eq.), in dry tetrahydrofuran (10.0 mL), was added dropwise over 10 mins a mixture of **3ad** (0.50 mmol, 1.0 eq.) and ethyl isocyanoacetate (1.0 mmol, 2.0 eq.) in tetrahydrofuran (10.0 mL). Hydrogen was evolved, the mixture became slightly warm, and a yellow-brown suspension resulted. The reaction was stirred at 40 °C overnight before quenched by 0.5 mL ethanol. The solvent was removed under reduced pressure, and the crude product was treated with water (20.0 mL) and ether (20.0 mL). The layers were separated, and the aqueous layer was extracted with ether (2x10.0 mL). The combined ether layer was dried over anhydrous sodium sulfate, and the solvent was removed to give the crude product which was purified by flash chromatography to afford the product **11c** (90.0 mg, 77%) as a white solid. R_f = 0.30 (Petroleum ether /EtOAc= 10/1); **m.p.** = 75 – 77 ºC; **^{1}H NMR** (400 MHz, $CDCl_3$) δ 9.52 (s, 1H), 7.55 (dd, *J* = 8.6, 5.6 Hz, 2H), 7.07 (t, *J* = 8.8 Hz, 2H), 6.94 (t, *J* = 2.8 Hz, 1H), 6.33 (t, *J* = 2.7 Hz, 1H), 4.28 (q, *J* = 7.1 Hz, 2H), 1.26 (t, *J* = 7.1 Hz, 3H); **^{13}C NMR** (100 MHz, $CDCl_3$) δ 162.2 (d, *J* = 244.0 Hz), 161.3, 131.4 (d, *J* = 3.0 Hz), 131.2 (d, *J* = 8.0 Hz), 122.1, 118.3, 114.5 (d, *J* = 21.0 Hz), 112.4, 60.4, 14.2; **^{19}F NMR** (376 MHz, $CDCl_3$) δ -115.9 (s, 1F); **HRMS** (EI, m/z): calcd. for $C_{13}H_{12}FNO_2$ $[M]^+$: 233.0847, found: 233.0848.

6.6 Synthesis of 11d

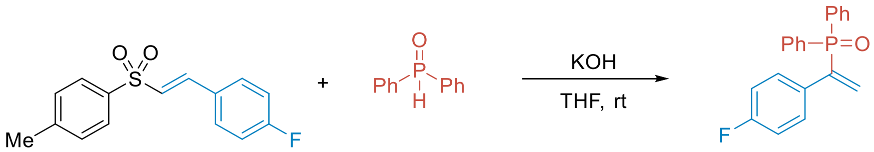

3ad, 0.50 mmol   0.75 mmol   **11d**, 52%

The compound **11d** was prepared according to reported methods.[31] To a 10 mL Schlenk tube equipped with a magnetic stir bar was charged with **3ad** (0.50 mmol, 1.0 eq.), diphenylphosphine oxide (0.75 mmol, 1.5 eq.) and KOH (0.75 mmol, 1.5 eq.) in 5.0 mL of THF. Under N_2 atmosphere, the solution was stirred at room temperature and monitored to completion by TLC analysis. After the reaction completion, the solution was concentrated under vacuum and the crude was purified by flash chromatography to afford the product **11d** (83.0 mg, 52%) as a white solid. R_f = 0.30 (Petroleum ether /EtOAc= 1/1); **m.p.** = 108 – 111 ºC; **^{1}H NMR** (400 MHz, $CDCl_3$) δ 7.68 (dd, *J* = 11.8, 7.2 Hz, 4H), 7.48 – 7.45 (m, 4H), 7.42 – 7.37 (m, 4H), 6.89 (t, *J* = 8.7 Hz, 2H), 6.16 (d, *J* = 40.2 Hz, 1H), 5.65 (d, *J* = 19.7 Hz, 1H); **^{13}C NMR** (100 MHz, $CDCl_3$) δ 162.8 (d, *J* = 246.0 Hz), 143.4 (d, *J* = 93.0 Hz), 133.6 (dd, *J* = 3.0, 10.0 Hz), 132.0 (d, *J* = 10.0 Hz), 132.0 (d, *J* = 10.0 Hz), 131.7 (d, *J* = 10.0 Hz), 130.9, 129.9 (dd, *J* = 4.0, 8.0 Hz), 128.5 (d, *J* = 12.0 Hz), 115.4 (d, *J* = 21.0 Hz); **^{19}F NMR** (376 MHz, $CDCl_3$) δ -113.5 (s, 1F); **HRMS** (EI, m/z): calcd. for $C_{20}H_{16}FOP$ $[M]^+$: 322.0917, found: 322.0918.

6.7 Synthesis of 11e

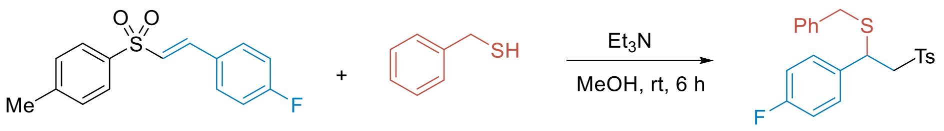

3ad, 0.50 mmol 2.50 mmol **11e**, 89%

The compound **11e** was synthesized by a procedure described by Xuan jun et al.[44] An oven-dried vial equipped with stirring bar was charged with **3ad** (0.50 mmol, 1.0 eq.), benzyl mercaptan (2.50 mmol, 5.0 eq.), triethylamine (0.75 mmol, 1.5 eq.), and dry MeOH (3.0 mL), Under N_2 atmosphere, the mixture was stirred for 6 h at room temperature. After completion, the reaction mixture was concentrated in vacuo and purified by flash column chromatography on silica gel with a mixture of petroleum ether and ethyl acetate as eluent, affording the product **11e** (178.0 mg, 89%) as a colorless oil. R_f = 0.30 (Petroleum ether /EtOAc= 10/1); **^{1}H NMR** (400 MHz, $CDCl_3$) δ 7.47 (d, *J* = 8.1 Hz, 2H), 7.30 – 7.24 (m, 3H), 7.18 – 7.13 (m, 4H), 7.08 (dd, *J* = 8.4, 5.4 Hz, 2H), 6.86 (t, *J* = 8.6 Hz, 2H), 4.17 (dd, *J* = 9.5, 4.5 Hz, 1H), 3.70 – 3.45 (m, 4H), 2.37 (s, 3H); **^{13}C NMR** (100 MHz, $CDCl_3$) δ 162.0 (d, *J* = 246.0 Hz), 144.5, 136.9, 136.4, 134.5 (d, *J* = 3.0 Hz), 129.7 (d, *J* = 8.0 Hz), 129.6, 128.9, 128.6, 127.9, 127.3, 115.4 (d, *J* = 22.0 Hz), 61.4, 42.3, 36.0, 21.5; **^{19}F NMR** (376 MHz, $CDCl_3$) δ -113.9 (s, 1F); **HRMS** (EI, m/z): calcd. for $C_{22}H_{21}FO_2S_2$ $[M]^+$: 400.0962, found: 400.0963.

6.8 Synthesis of 11f

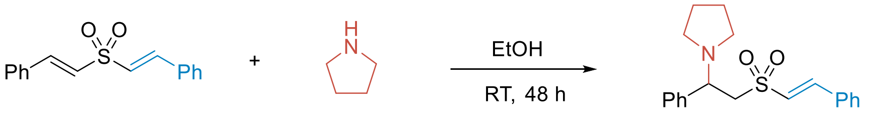

5da, 0.10 mmol 0.40 mmol **11f**, 87%

To a 10 mL Schlenk tube equipped with a magnetic stir bar was charged with **5da** (0.10 mmol, 1.0 eq.) and EtOH (1.0 mL), under N_2 atmosphere, the pyrrolidine (0.40 mmol, 4.0 eq.) was added and stirred at room temperature for 24 h. After completion, the reaction mixture was concentrated in vacuo and purified by flash column chromatography on silica gel with a mixture of petroleum ether and ethyl acetate (3:1) as eluent, affording the product **11f** (30.0 mg, 87%) as a yellow oil. R_f = 0.20 (Petroleum ether /EtOAc= 5/1); **^{1}H NMR** (400 MHz, $CDCl_3$) δ 7.37 – 7.29 (m, 8H), 7.22 (d, *J* = 7.1 Hz, 3H), 6.20 (d, *J* = 15.5 Hz, 1H), 4.05 (dd, *J* = 8.0, 6.2 Hz, 1H), 3.82 (dd, *J* = 14.6, 6.0 Hz, 1H), 3.48 (dd, *J* = 14.6, 8.2 Hz, 1H), 2.54 – 2.49 (m, 2H), 2.42 – 2.37 (m, 2H), 1.72 – 1.62 (m, 4H); **^{13}C NMR** (100 MHz, $CDCl_3$) δ 142.7, 137.9, 132.4, 131.0, 128.9, 128.9, 128.6, 128.4, 128.2, 126.2, 63.5, 60.6, 50.7, 23.2; **HRMS** (EI, m/z): calcd. for $C_{20}H_{23}NO_2S$ $[M]^+$: 341.1444, found: 341.1445.

6.9 Synthesis of 11g

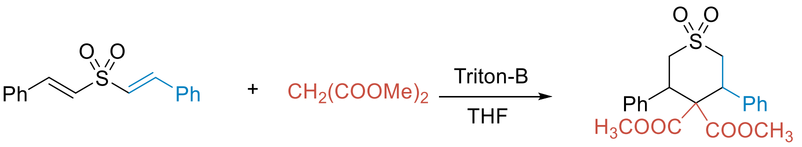

5da, 0.20 mmol 0.60 mmol **11g**, 88%

The preparation of compound **11g** was according to reported literature methods.[45] To a solution of **5da** (0.10 mmol, 1.0 eq.) and dimethyl malonate in 1.0 mL of tetrahydrofuran, under N_2 atmosphere, a catalytic amount of Triton-B (20 uL) was added and refluxed for 12 h. The reaction mixture is cooled and the solvent is removed under reduced pressure. The crude product was purified by flash column chromatography on silica gel with a mixture of petroleum ether and ethyl acetate (3:1) as eluent, affording

the final product **11g** (71.0 mg, 88%) as a white solid. R_f = 0.30 (Petroleum ether /EtOAc= 5/1); **m.p.** = 177 – 180 °C; **^{1}H NMR** (400 MHz, $CDCl_3$) δ 7.30 – 7.26 (m, 6H), 7.15 – 7.13 (m, 4H), 4.34 – 4.21 (m, 4H), 3.56 (s, 3H), 3.35 (s, 3H), 3.14 (d, J = 13.0 Hz, 2H); **^{13}C NMR** (100 MHz, $CDCl_3$) δ 169.0, 168.8, 137.7, 128.7, 128.6, 128.5, 64.4, 52.4, 52.3, 51.9, 48.1; **HRMS** (EI, m/z): calcd. for $C_{21}H_{22}O_6S$ $[M+H]^+$: 403.1210, found: 403.1204.

6.10 Synthesis of 11h

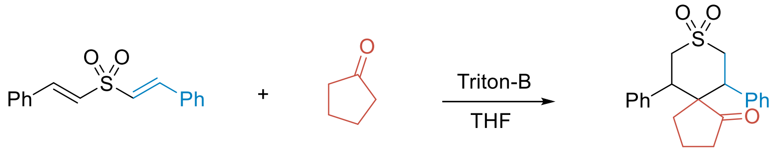

The preparation of compound **11h** was according to reported literature methods.[46] To a solution of **5da** (0.10 mmol, 1.0 eq.) and cyclopentanone in 1.0 mL of tetrahydrofuran, under N_2 atmosphere, a catalytic amount of Triton-B (20 uL) was added and refluxed for 12 h. The reaction mixture is cooled and the solvent is removed under reduced pressure. The product was purified by flash column chromatography on silica gel with a mixture of petroleum ether and ethyl acetate (5:1) as eluent, affording the final product **11h** (17.0 mg, 47%) as a white solid. R_f = 0.24 (Petroleum ether /EtOAc= 5/1); **m.p.** = 264 – 266 °C; **^{1}H NMR** (400 MHz, $CDCl_3$) δ 7.31 – 7.24 (m, 6H), 7.10 (d, J = 7.2 Hz, 4H), 4.01 (d, J = 13.1 Hz, 2H), 3.58 (t, J = 13.8 Hz, 2H), 3.19 (d, J = 14.1 Hz, 2H), 2.20 (t, J = 7.3 Hz, 2H), 1.18 (t, J = 8.1 Hz, 2H), 0.85 – 0.77 (m, 2H); **^{13}C NMR** (100 MHz, $CDCl_3$) δ 220.1, 137.2, 129.2, 128.9, 128.2, 58.4, 52.5, 46.8, 39.5, 22.6, 18.8; **HRMS** (EI, m/z): calcd. for $C_{21}H_{22}O_3S$ $[M]^+$: 354.1284, found: 354.1286.

6.11 Synthesis of 11i

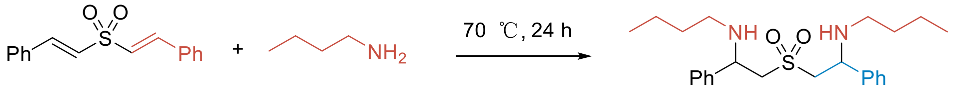

The compound **11i** was synthesized by an analogous procedure described by Xuan jun et al.[44] An oven-dried vial equipped with stirring bar was charged with **5da** (0.10 mmol, 1.0 eq.) and butylamine (0.50 mL), under N_2 atmosphere, the mixture was stirred at 70°C for 24 h. After completion, the reaction mixture was concentrated in vacuo and purified by flash column chromatography on silica gel with a mixture of petroleum ether and ethyl acetate (5:1) as eluent, affording the final product **11i** (42.0 mg, 99%) as a yellow oil. R_f = 0.22 (Petroleum ether /EtOAc= 5/1); **^{1}H NMR** (400 MHz, $CDCl_3$) δ 7.36 – 7.33 (m, 4H), 7.29 – 7.26 (m, 6H), 4.28 – 4.21 (m, 2H), 3.53 (dd, J = 14.3, 10.0 Hz, 1H), 3.29 – 3.27 (m, 2H), 3.20 (dd, J = 14.3, 2.7 Hz, 1H), 2.46 – 2.34 (m, 4H), 1.96 (s, 2H), 1.44 – 1.37 (m, 4H), 1.32 – 1.21 (m, 4H), 0.85 (t, J = 7.2 Hz, 6H); **^{13}C NMR** (100 MHz, $CDCl_3$) δ 141.5, 141.5, 129.0, 129.0, 128.1, 128.1, 127.1, 126.9, 61.3, 61.2, 58.1, 58.0, 47.1, 47.1, 32.2, 32.1, 20.5, 20.5, 14.0; **HRMS** (EI, m/z): calcd. for $C_{24}H_{36}N_2O_2S$ $[M]^+$: 416.2492, found: 416.2492.

6.12 New polymer synthesis

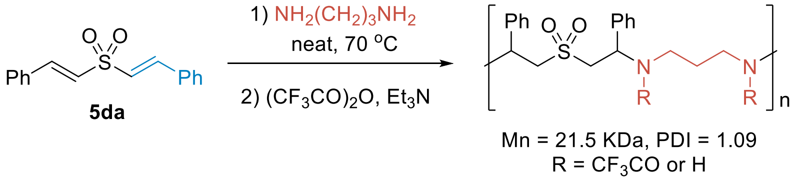

1): An oven-dried vial equipped with stirring bar was charged with **5da** (0.50 mmol, 1.0 eq.) and butylamine (0.60 mmol), under N_2 atmosphere, the mixture was stirred at 70℃. Progress of the reaction was monitored by TLC until **5da** is completely consumed. The reaction mixture was concentrated in vacuo to give the crude product which washed sequentially with a small amount of ethanol and *n*-hexane. The obtained polymer as a white solid (140.0 mg) was used for the next step without further purification. **^{1}H NMR** (400 MHz, $CDCl_3$) δ 7.39 – 7.20 (m, 10H), 4.14 (s, 2H), 3.51 – 3.38 (m, 1H), 3.16 (s, 3H), 2.47 – 2.37 (m, 4H), 2.06 (s, 4H), 1.55 – 1.49 (m, 2H); **^{13}C NMR** (100 MHz, $CDCl_3$) δ 141.3, 129.0, 128.1, 128.0, 127.1, 127.0, 61.1, 67.9, 45.7, 45.4, 45.2, 29.9.

2): Add polymer (34.6 mg), TFAA (63.1 mg), Et_3N (41.0 mg) and dry DCM (0.50 mL) in one pot, under N_2 atmosphere, the mixture was stirred at 0 °C for 3 h. The resulting mixture was washed three times with saturated sodium bicarbonate solution (20mL×3), dried over Na_2SO_4, and evaporated under reduced pressure. The residue was washed with *n*-hexane to afford final product as a white solid (42.0 mg).

GPC characterization (R = CF_3CO)

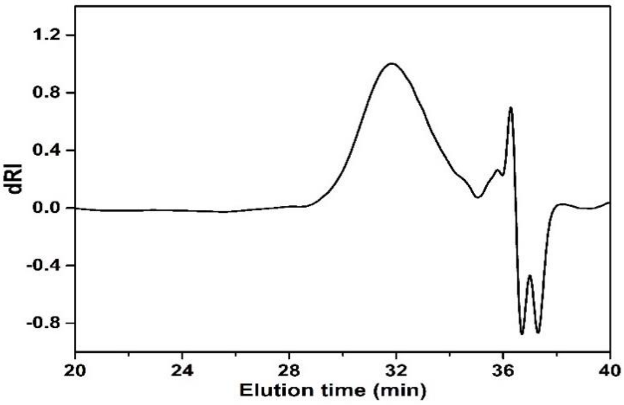

Supplementary Figure 28. GPC characterization

Molecular weight result calculated using a PEG standard curve:

Mn = 21.5 kDa, PDI = 1.09

6.13 Divinyl sulfone-based *D-p-A-p-D* dyes

6.13.1 Synthesis of 4,4'-((1*E*,1'*E*)-sulfonylbis(ethene-2,1-diyl))bis(bromobenzene)

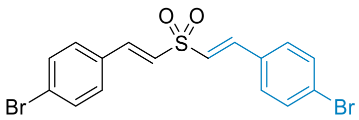

According to **general procedure-H** (10.0 mmol scale), this compound was obtained as white solid (501.0 mg, 78% yield); R_f = 0.50 (PE/EA=5:1); **m.p.** = 235-238 °C; **^{1}H NMR** (400 MHz, $CDCl_3$) δ 7.59 – 7.37 (m, 6H), 7.37 (d, *J* = 7.5 Hz, 4H), 6.84 (d, *J* = 15.4 Hz, 2H); **^{13}C NMR** (100 MHz, $CDCl_3$) δ 142.6, 132.6, 131.5, 130.1, 126.9, 126.0. **HRMS** (EI, m/z): calcd. for $C_{16}H_{12}Br_2O_2S$ $[M]^+$: 425.8919, found: 425.8920.

6.13.2 Synthesis of 5sv according to the reported literature.[46]

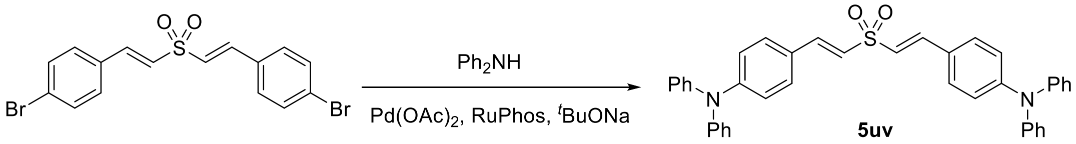

A screw-cap vial equipped with a magnetic stir bar was charged with the aryl halide (0.25 mmol), secondary amine (0.50 mmol), $Pd(OAc)_2$ (0.0025 mmol), RuPhos (0.0050 mmol), and powdered ^{t}BuONa (0.30 mmol). The vial was transferred to a preheated oil bath (110 °C) and stirred for 12 h. And then, the resulting mixture was cooled and dissolved in CH_2Cl_2/H_2O (1:1). The organic phase was separated and evaporated in vacuo. The product was purified by flash column chromatography on silica gel column. Finally, the compound **5sv** was obtained as a yellow-green solid. R_f = 0.43 (Petroleum ether /EtOAc= 20/1); **m.p.** = 125-130 ºC; **^{1}H NMR** (400 MHz, $CDCl_3$) δ 7.51 (d, J = 15.3 Hz, 2H), 7.35 – 7.27 (m, 12H), 7.16 – 7.08 (m, 12H), 6.99 (d, J = 8.5 Hz, 4H), 6.65 (d, J = 15.3 Hz, 2H). **^{13}C NMR** (100 MHz, Chloroform-*d*) δ 150.7, 146.8, 142.4, 129.9, 129.7, 125.8, 125.4, 124.5, 123.6, 121.4; **HRMS** (EI, m/z): calcd. for $C_{40}H_{32}N_2O_2S$ $[M]^+$: 604.2179, found: 604.2179. The spectroscopic data of **5uv** matched the reported values.[47]

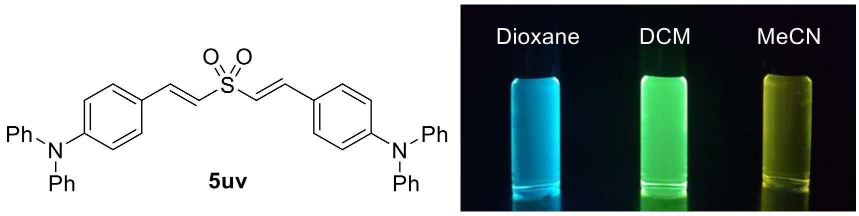

5uv in 1,4-dioxane (left), dichloromethane (middle) and acetonitrile (right) [10^{-3} M] under UV irradiation (365 nm)

Supplementary Figure 29. Divinyl sulfones-based D-π-A-π-D dyes

Section 7. NMR spectra

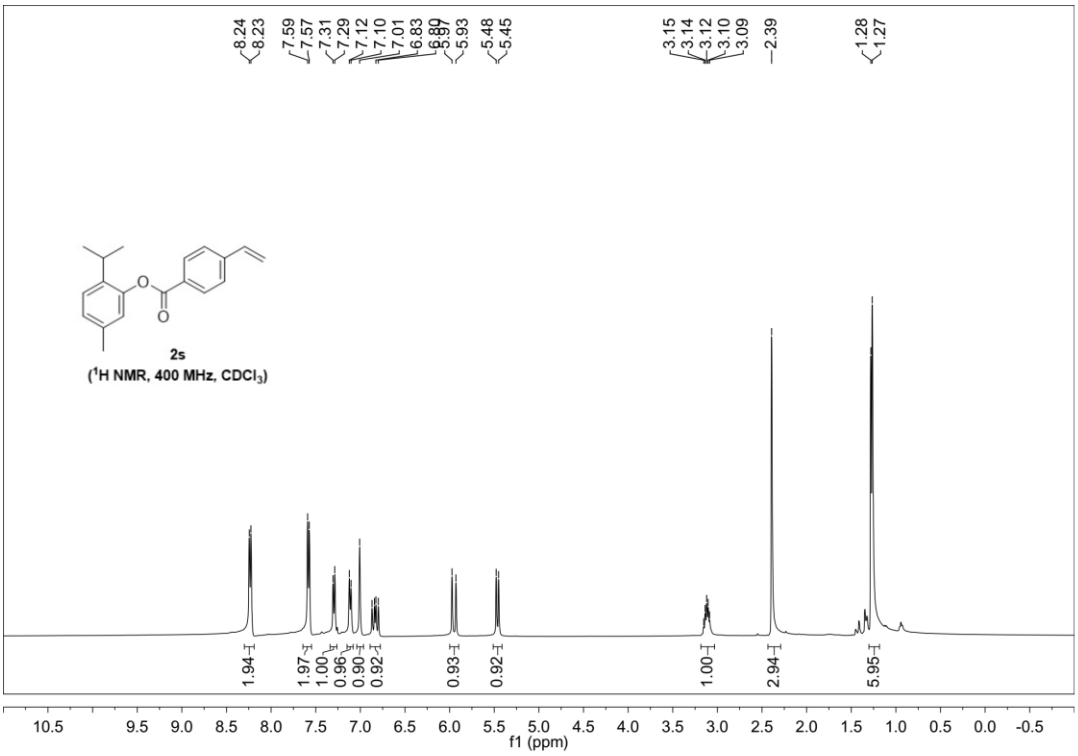

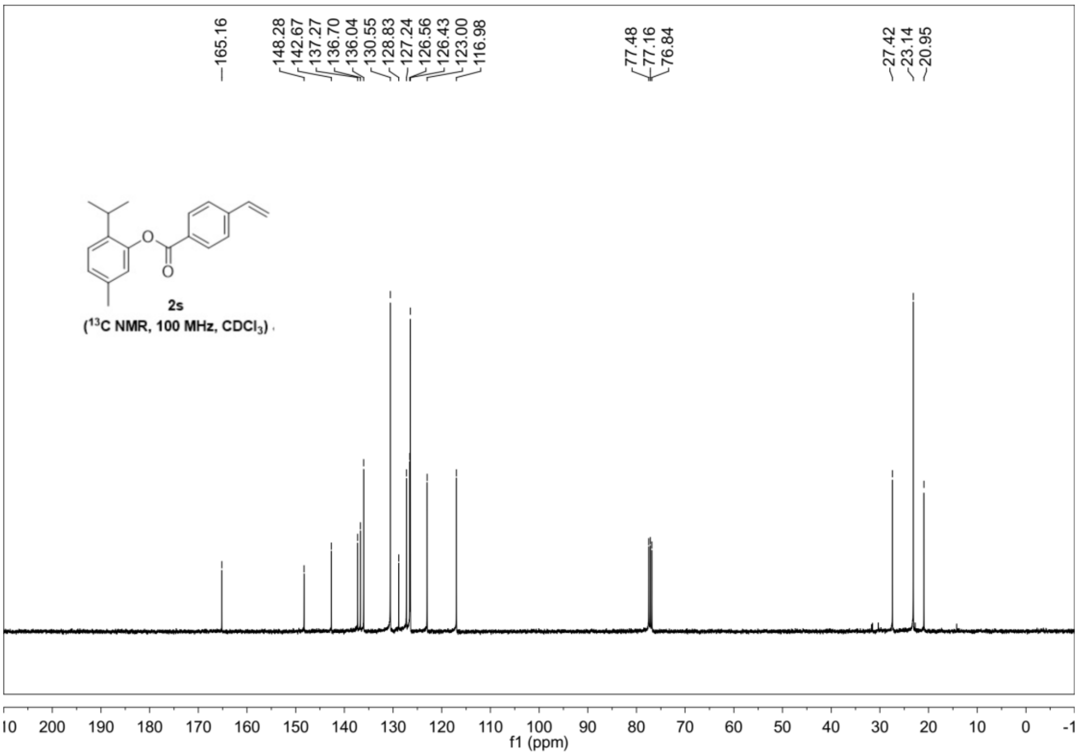

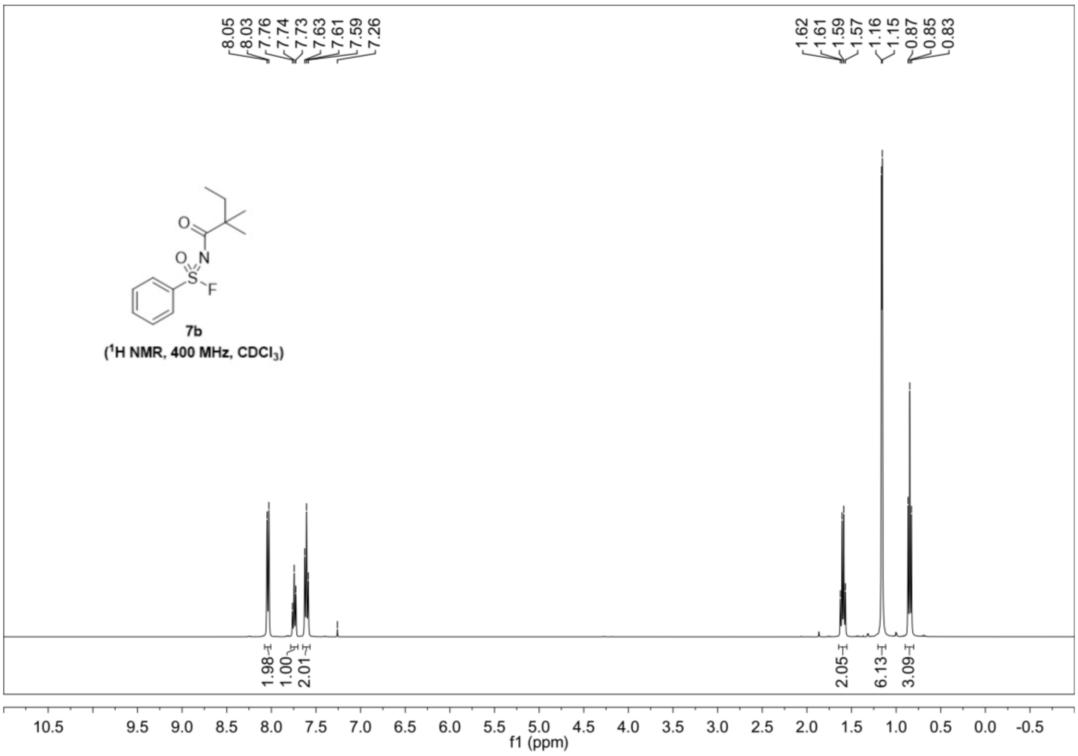
8.05
8.03
7.76
7.74
7.73
7.63
7.61
7.59
7.26
1.62
1.61
1.59
1.57
1.16
1.15
0.87
0.85
0.83
7b
(1H NMR, 400 MHz, CDCl3)
1.98
1.00
2.01
2.05
6.13
3.09
f1 (ppm)

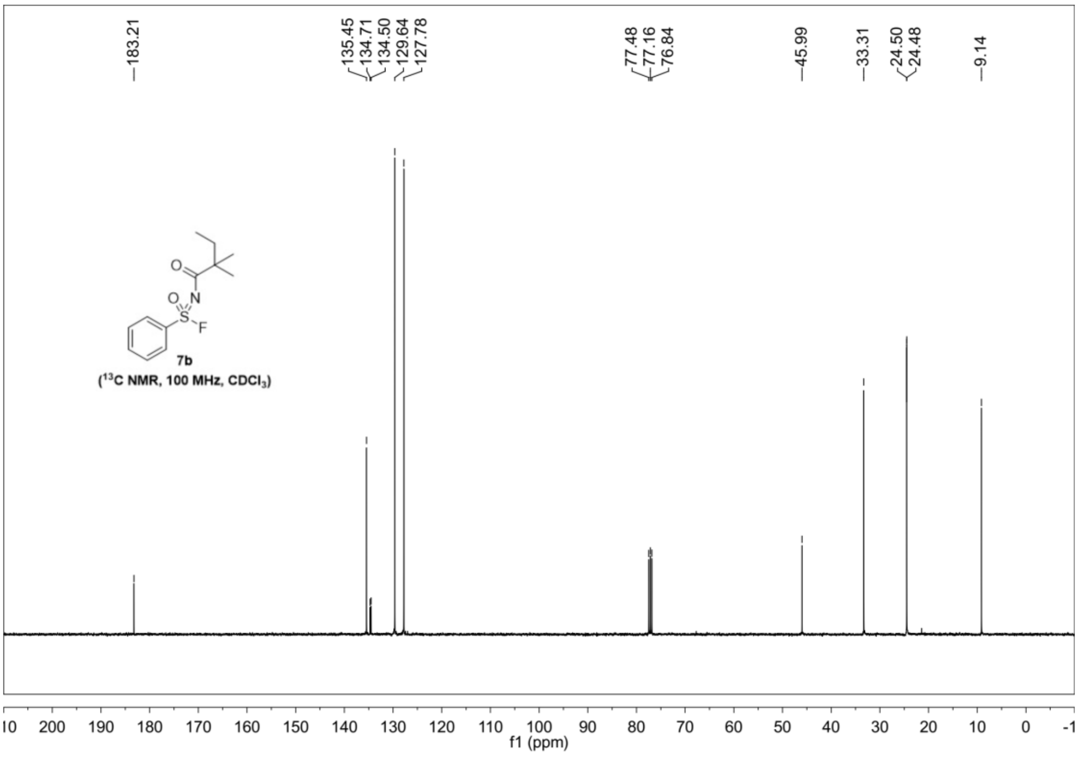
183.21
135.45
134.71
134.50
129.64
127.78
77.48
77.16
76.84
45.99
33.31
24.50
24.48
9.14
7b
(13C NMR, 100 MHz, CDCl3)
f1 (ppm)

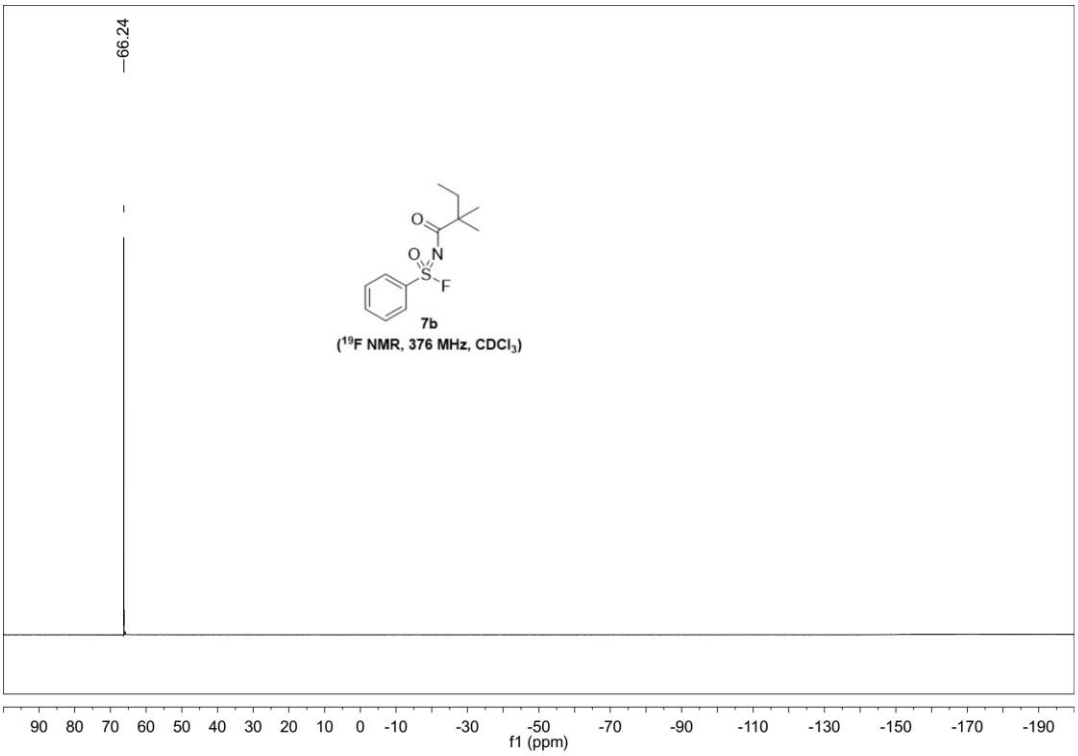
-66.24
7b
(^{19}F NMR, 376 MHz, CDCl$_3$)
90 80 70 60 50 40 30 20 10 0 -10 -30 -50 -70 -90 -110 -130 -150 -170 -190
f1 (ppm)

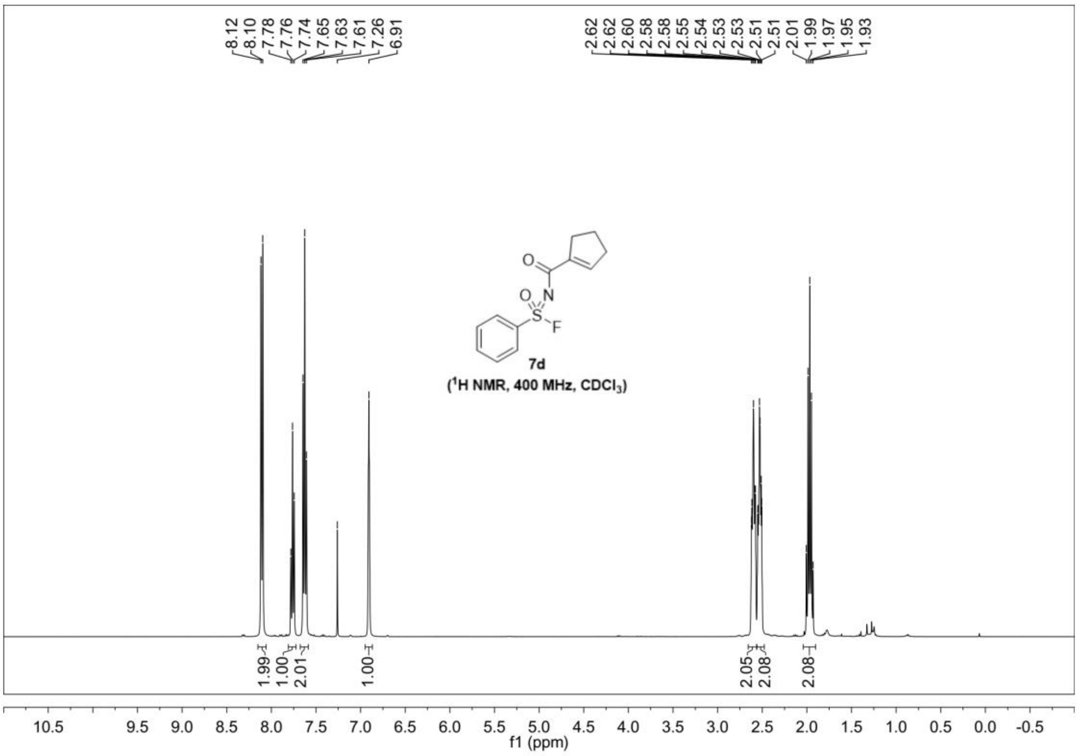
8.12 8.10 7.78 7.76 7.74 7.65 7.63 7.61 7.26 6.91
2.62 2.62 2.60 2.58 2.58 2.55 2.54 2.53 2.53 2.51 2.51 2.01 1.99 1.97 1.95 1.93
7d
(^{1}H NMR, 400 MHz, CDCl$_3$)
1.99 1.00 2.01 1.00 2.05 2.08 2.08
10.5 9.5 9.0 8.5 8.0 7.5 7.0 6.5 6.0 5.5 5.0 4.5 4.0 3.5 3.0 2.5 2.0 1.5 1.0 0.5 0.0 -0.5
f1 (ppm)

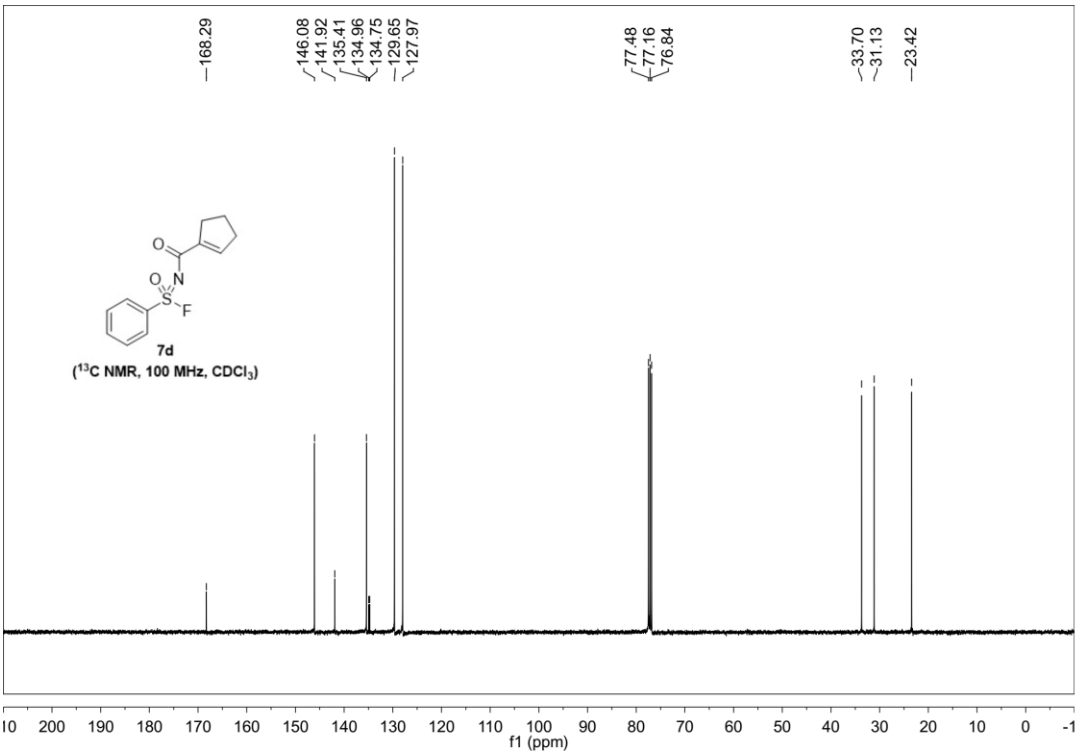
168.29
146.08
141.92
135.41
134.96
134.75
129.65
127.97
77.48
77.16
76.84
33.70
31.13
23.42
7d
(13C NMR, 100 MHz, CDCl3)
f1 (ppm)

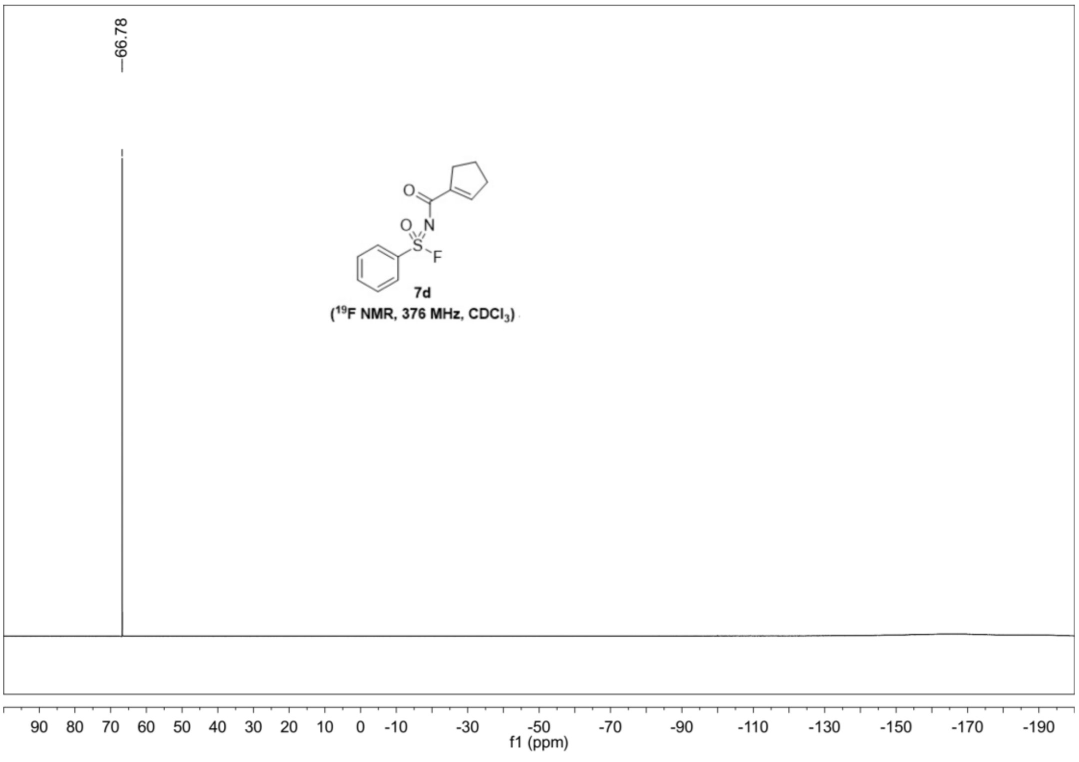
-66.78
7d
(19F NMR, 376 MHz, CDCl3)
f1 (ppm)

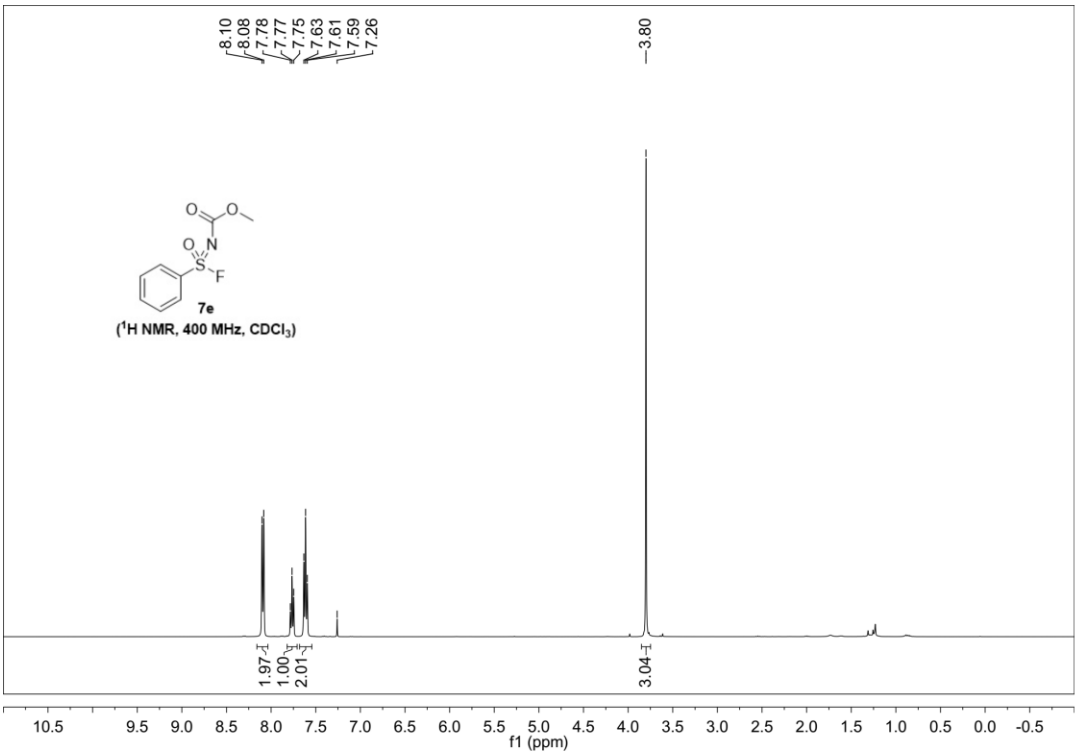
8.10
8.08
7.78
7.77
7.75
7.63
7.61
7.59
7.26
3.80
7e
(1H NMR, 400 MHz, CDCl3)
1.97
1.00
2.01
3.04
f1 (ppm)

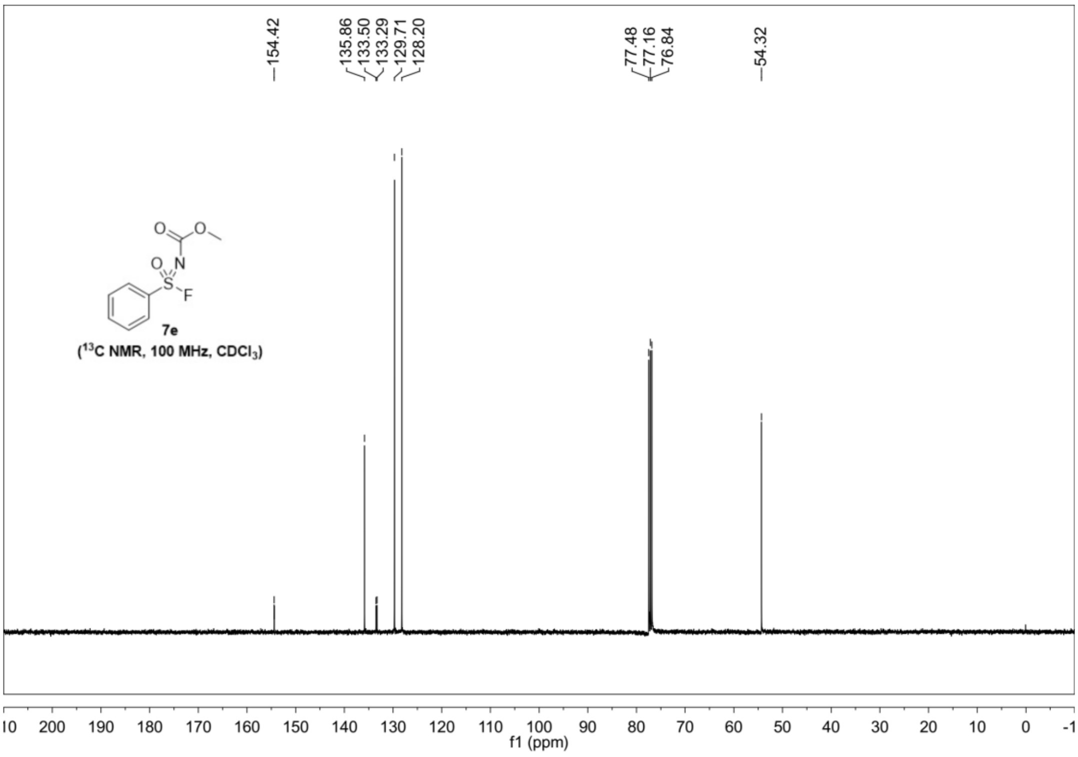
154.42
135.86
133.50
133.29
129.71
128.20
77.48
77.16
76.84
54.32
7e
(13C NMR, 100 MHz, CDCl3)
f1 (ppm)

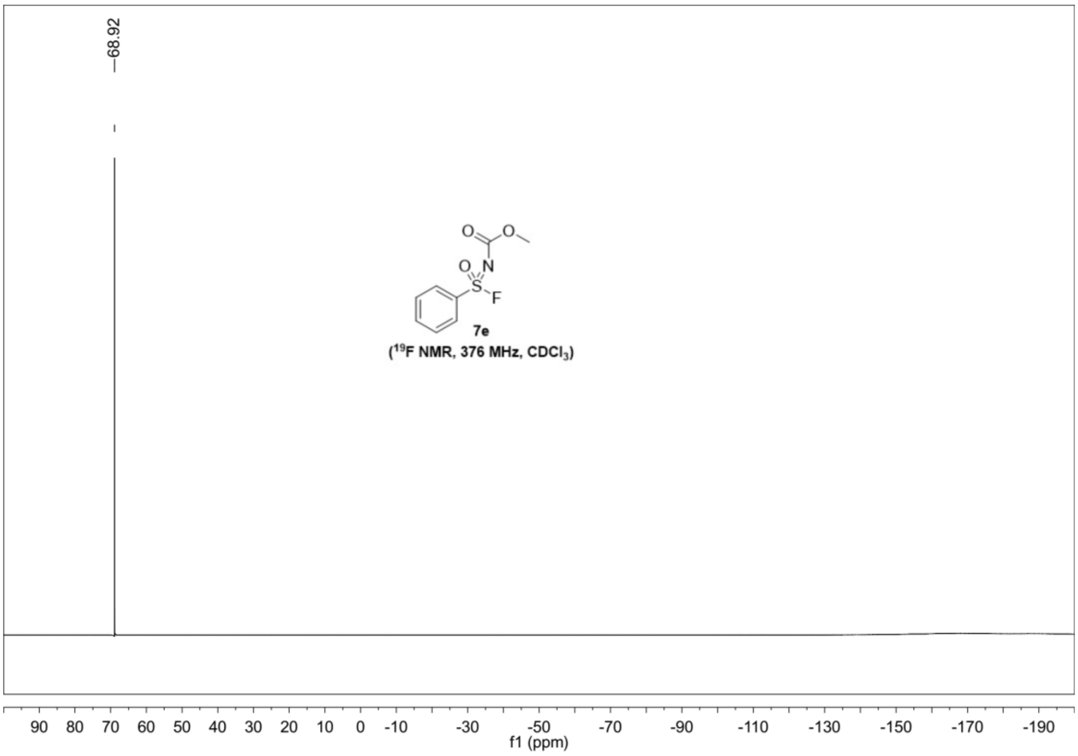

68.92
7e
(^{19}F NMR, 376 MHz, CDCl$_3$)
90 80 70 60 50 40 30 20 10 0 -10 -30 -50 -70 -90 -110 -130 -150 -170 -190
f1 (ppm)

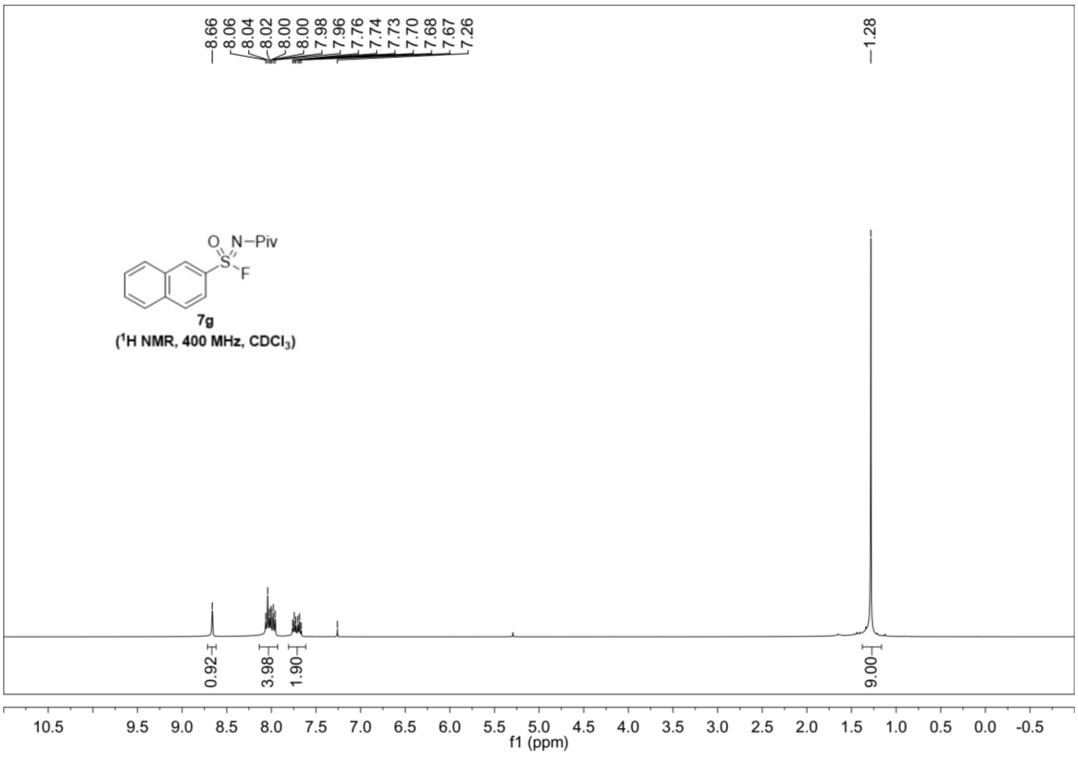

8.66 8.06 8.04 8.02 8.00 8.00 7.98 7.96 7.76 7.74 7.73 7.70 7.68 7.67 7.26
1.28
N−Piv
F
7g
(^{1}H NMR, 400 MHz, CDCl$_3$)
0.92 3.98 1.90 9.00
10.5 9.5 9.0 8.5 8.0 7.5 7.0 6.5 6.0 5.5 5.0 4.5 4.0 3.5 3.0 2.5 2.0 1.5 1.0 0.5 0.0 -0.5
f1 (ppm)

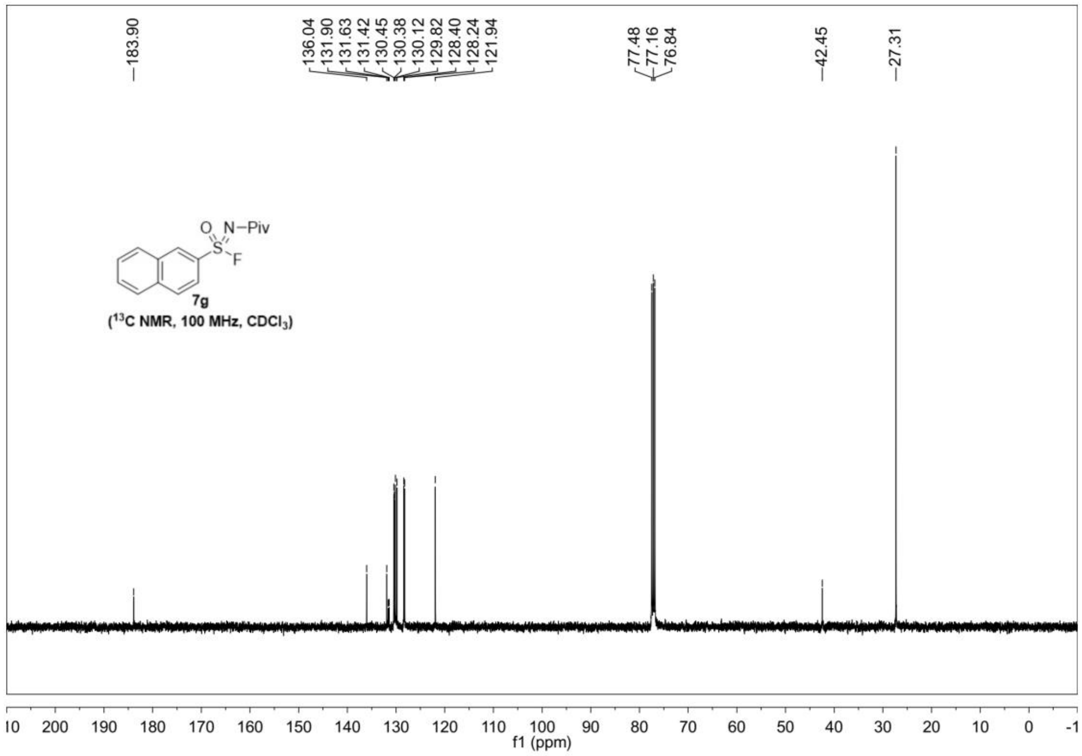
183.90
136.04
131.90
131.63
131.42
130.45
130.38
130.12
129.82
128.40
128.24
121.94
77.48
77.16
76.84
42.45
27.31
O
N−Piv
S
F
7g
(^{13}C NMR, 100 MHz, CDCl$_3$)
f1 (ppm)

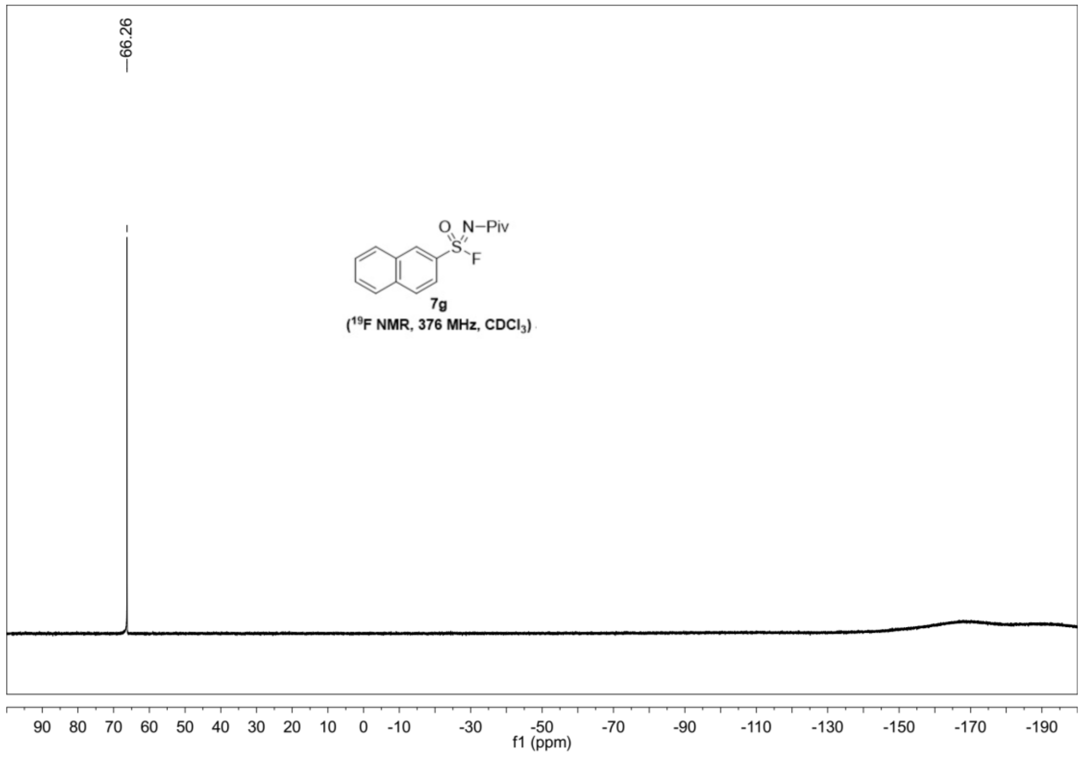
66.26
O
N−Piv
S
F
7g
(^{19}F NMR, 376 MHz, CDCl$_3$)
f1 (ppm)

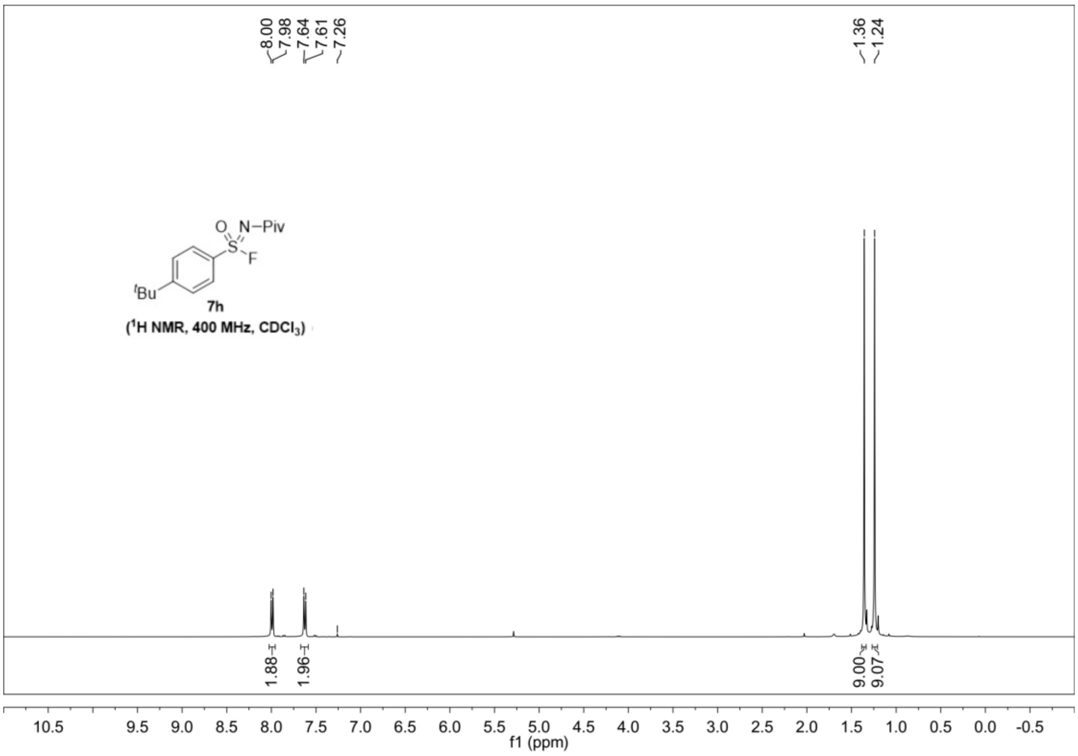

8.00
7.98
7.64
7.61
7.26
1.36
1.24
O N−Piv
S
F
tBu
7h
(1H NMR, 400 MHz, CDCl3)
1.88
1.96
9.00
9.07
10.5 9.5 9.0 8.5 8.0 7.5 7.0 6.5 6.0 5.5 5.0 4.5 4.0 3.5 3.0 2.5 2.0 1.5 1.0 0.5 0.0 -0.5
f1 (ppm)

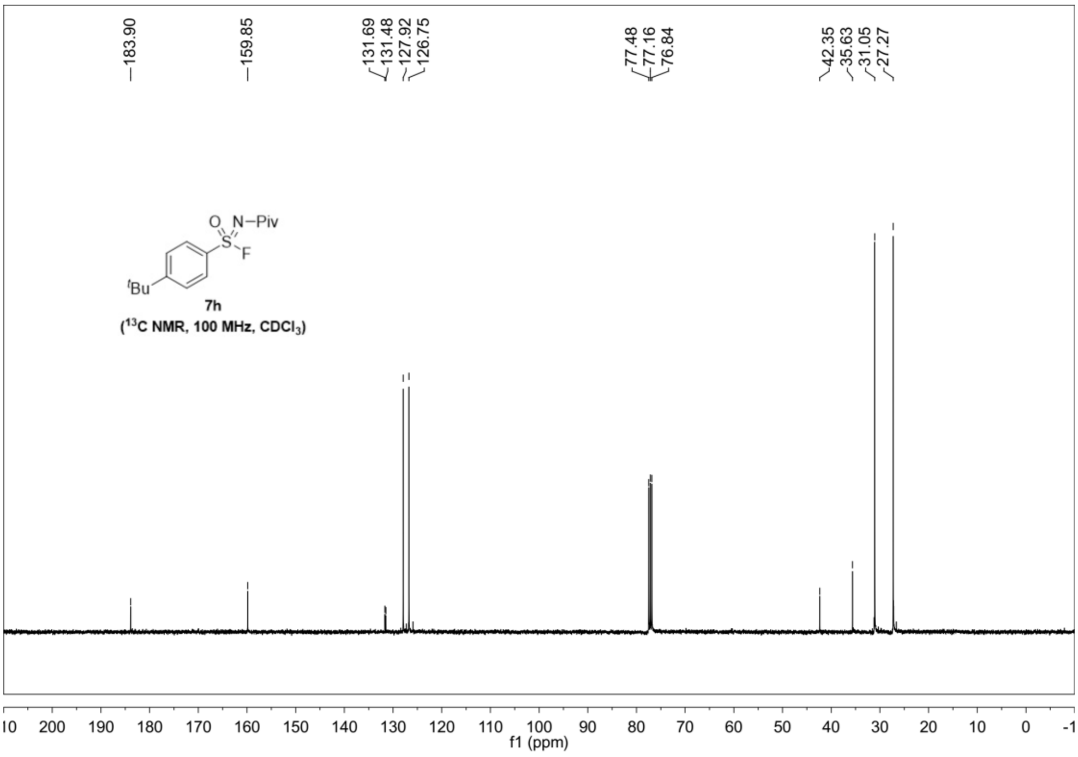

183.90
159.85
131.69
131.48
127.92
126.75
77.48
77.16
76.84
42.35
35.63
31.05
27.27
O N−Piv
S
F
tBu
7h
(13C NMR, 100 MHz, CDCl3)
200 190 180 170 160 150 140 130 120 110 100 90 80 70 60 50 40 30 20 10 0
f1 (ppm)

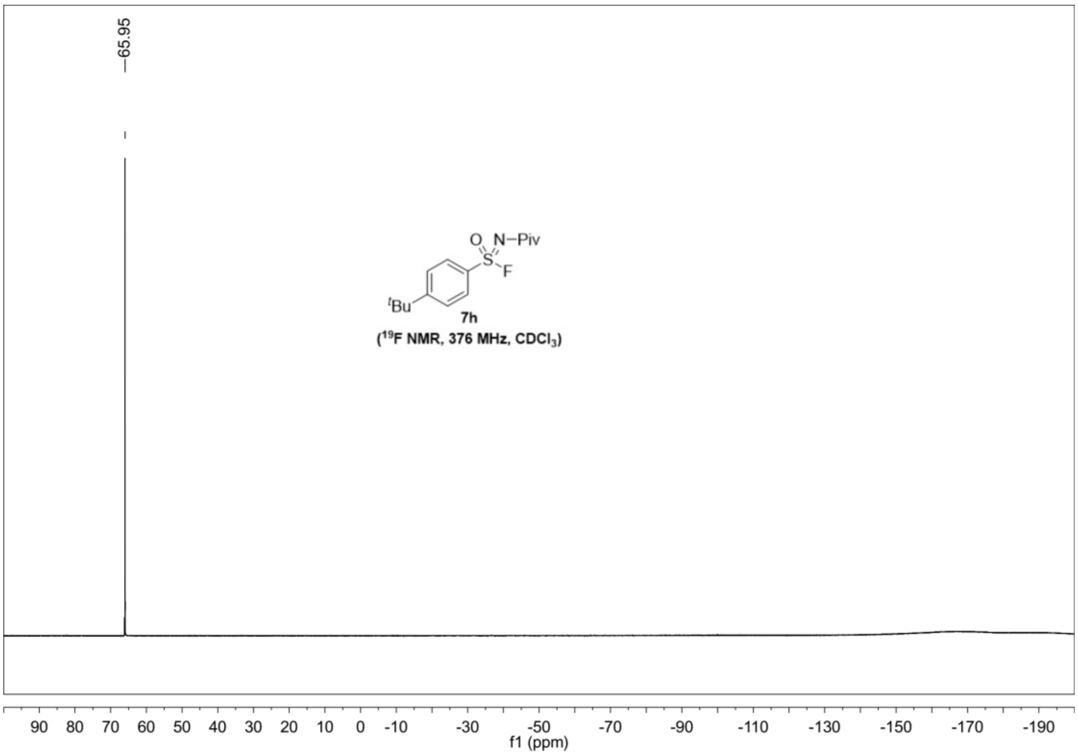

—65.95
O N−Piv
S
F
tBu
7h
(19F NMR, 376 MHz, CDCl3)
90 80 70 60 50 40 30 20 10 0 -10 -30 -50 -70 -90 -110 -130 -150 -170 -190
f1 (ppm)

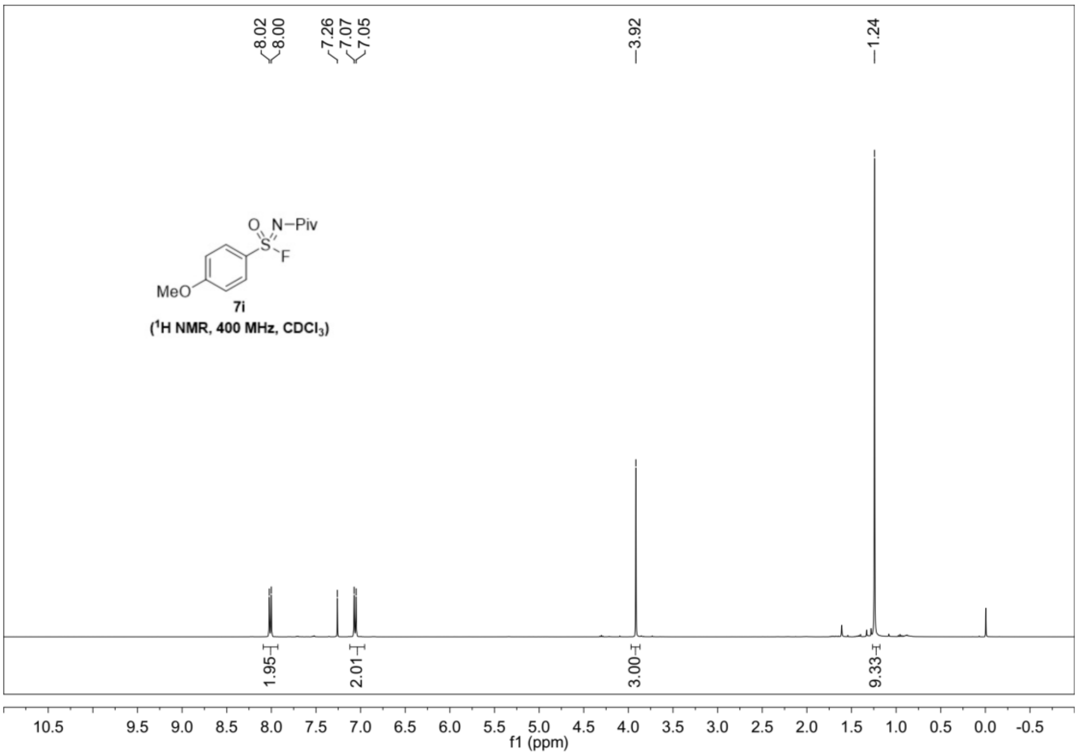

8.02
8.00
7.26
7.07
7.05
—3.92
—1.24
O N−Piv
S
F
MeO
7i
(1H NMR, 400 MHz, CDCl3)
1.95
2.01
3.00
9.33
10.5 9.5 9.0 8.5 8.0 7.5 7.0 6.5 6.0 5.5 5.0 4.5 4.0 3.5 3.0 2.5 2.0 1.5 1.0 0.5 0.0 -0.5
f1 (ppm)

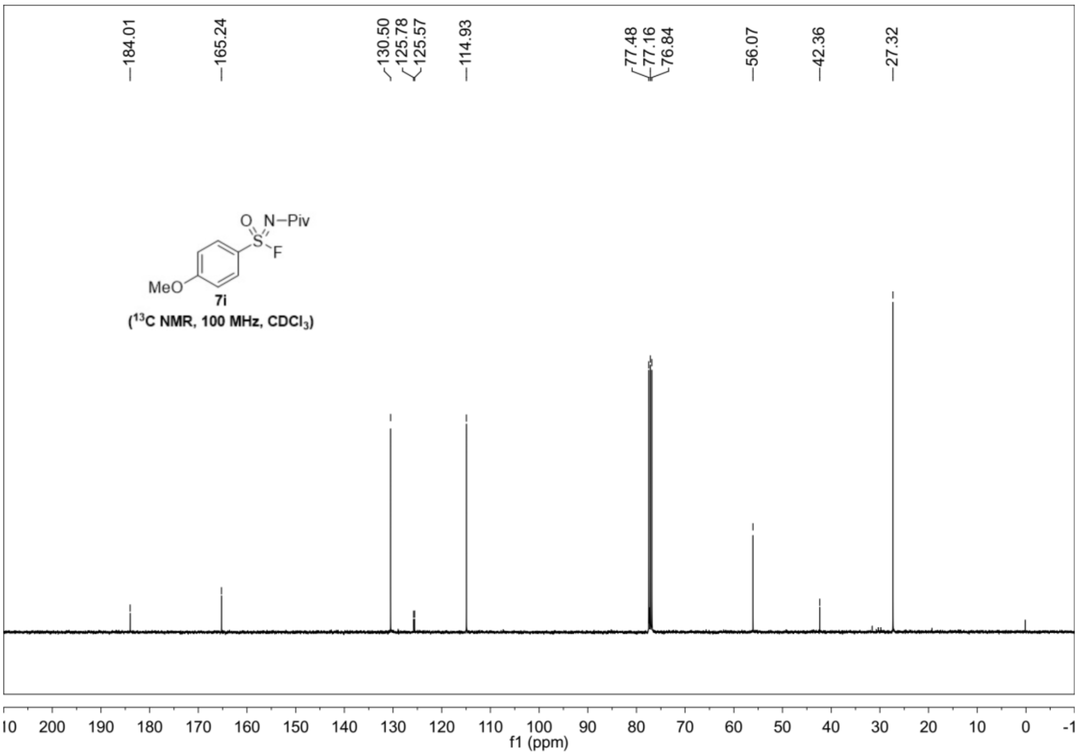
184.01
165.24
130.50
125.78
125.57
114.93
77.48
77.16
76.84
56.07
42.36
27.32
O N−Piv
S
F
MeO
7i
(13C NMR, 100 MHz, CDCl3)
f1 (ppm)

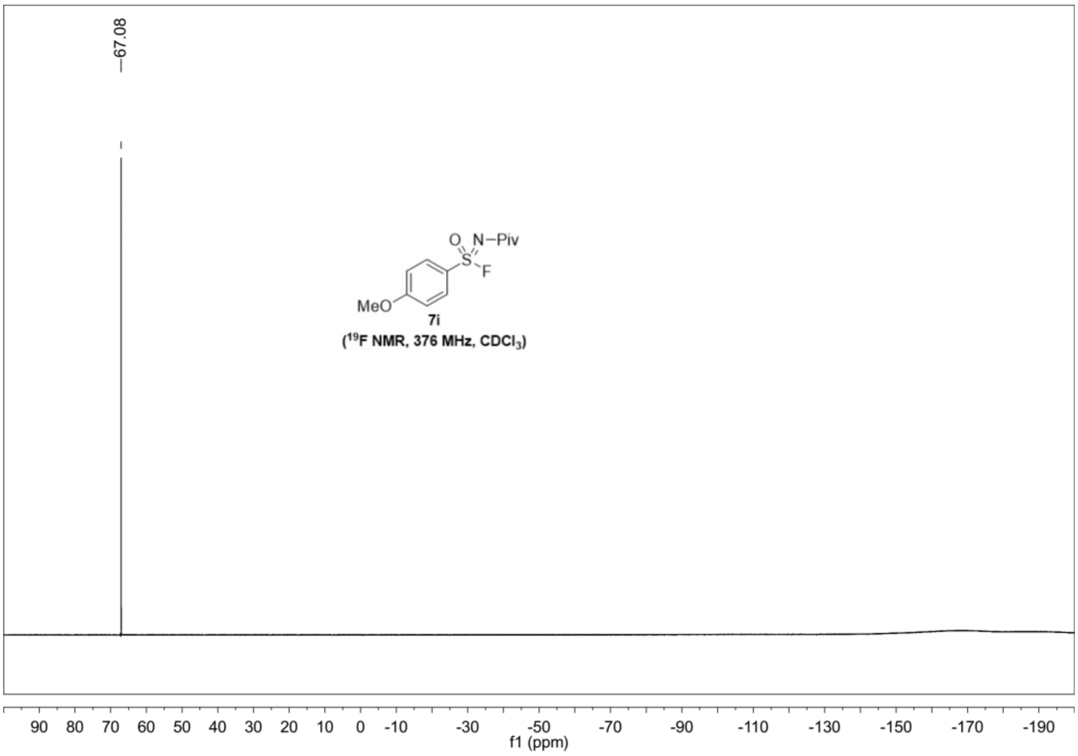
67.08
O N−Piv
S
F
MeO
7i
(19F NMR, 376 MHz, CDCl3)
f1 (ppm)

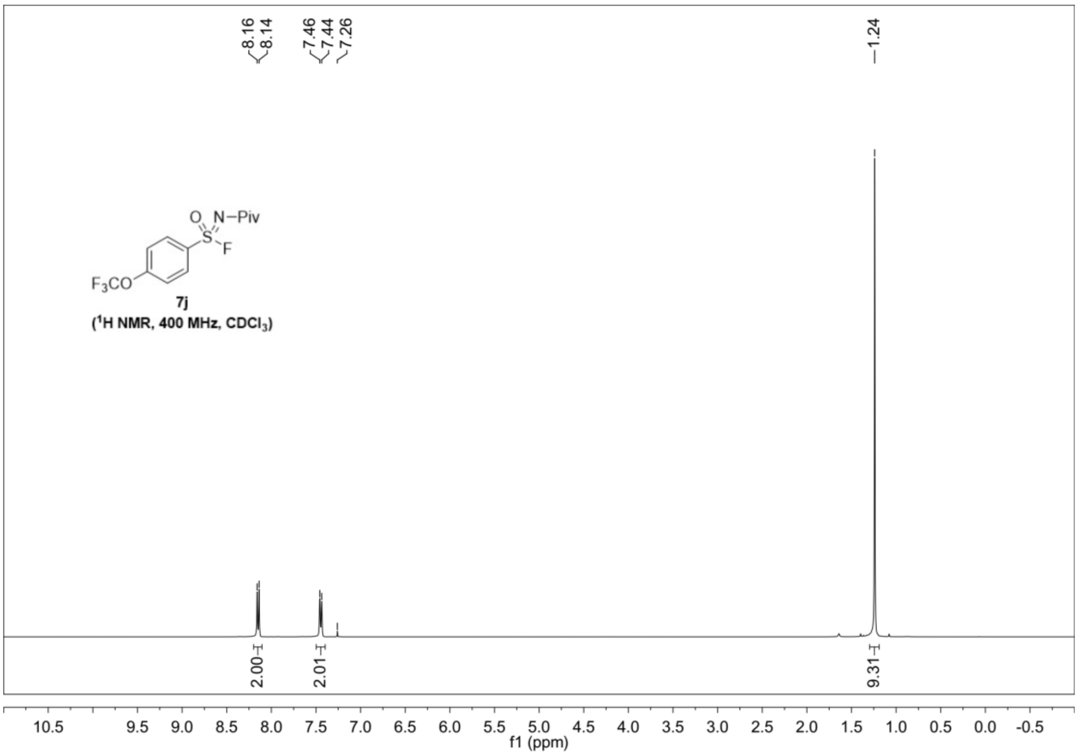

8.16
8.14
7.46
7.44
7.26
1.24
O N−Piv
S
F
F3CO
7j
(1H NMR, 400 MHz, CDCl3)
2.00
2.01
9.31
10.5 9.5 9.0 8.5 8.0 7.5 7.0 6.5 6.0 5.5 5.0 4.5 4.0 3.5 3.0 2.5 2.0 1.5 1.0 0.5 0.0 -0.5
f1 (ppm)

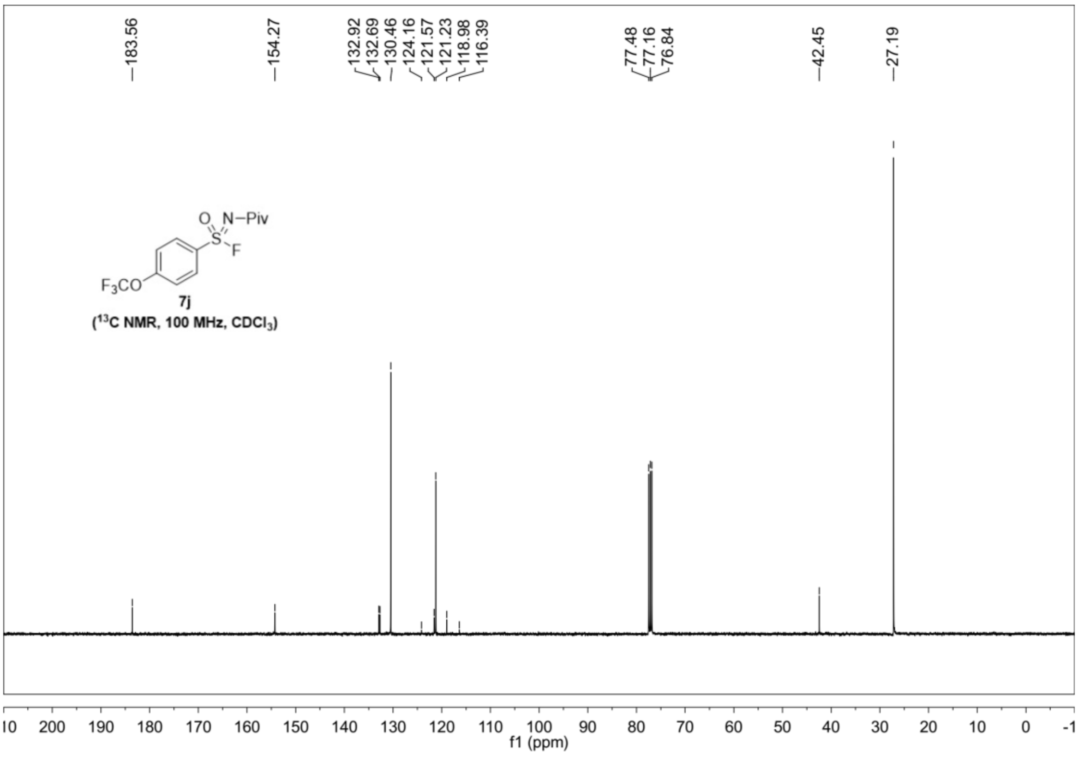

183.56
154.27
132.92
132.69
130.46
124.16
121.57
121.23
118.98
116.39
77.48
77.16
76.84
42.45
27.19
O N−Piv
S
F
F3CO
7j
(13C NMR, 100 MHz, CDCl3)
200 190 180 170 160 150 140 130 120 110 100 90 80 70 60 50 40 30 20 10 0
f1 (ppm)

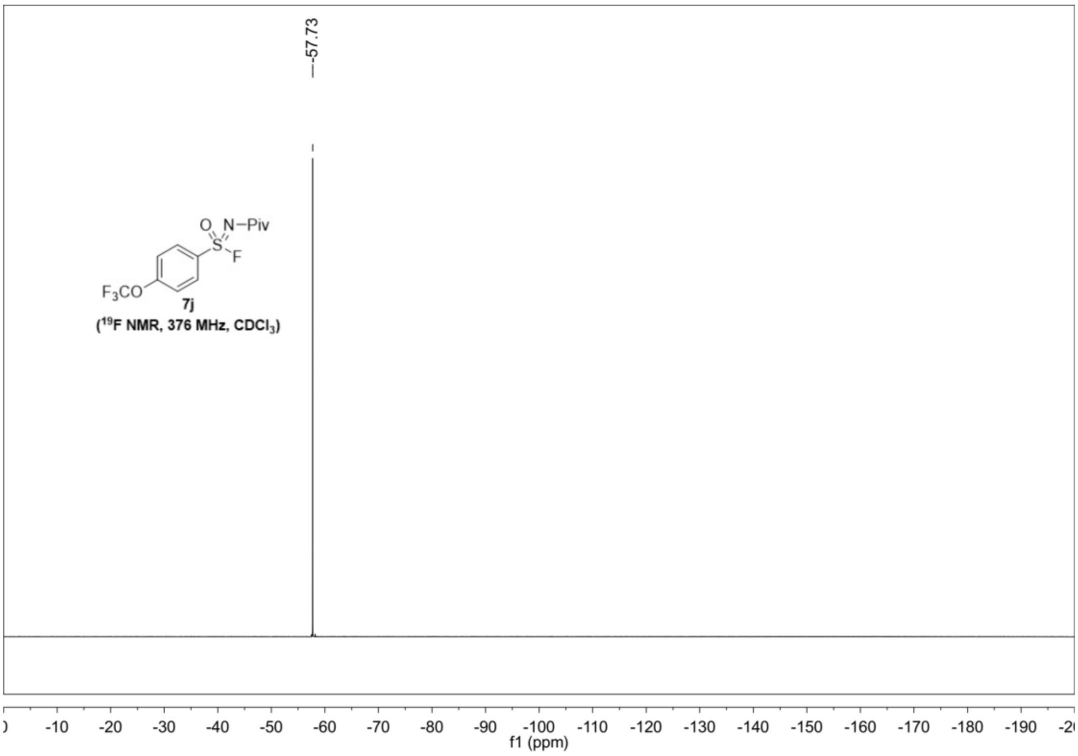
-57.73
N−Piv
F
F3CO
7j
(19F NMR, 376 MHz, CDCl3)
-10 -20 -30 -40 -50 -60 -70 -80 -90 -100 -110 -120 -130 -140 -150 -160 -170 -180 -190
f1 (ppm)

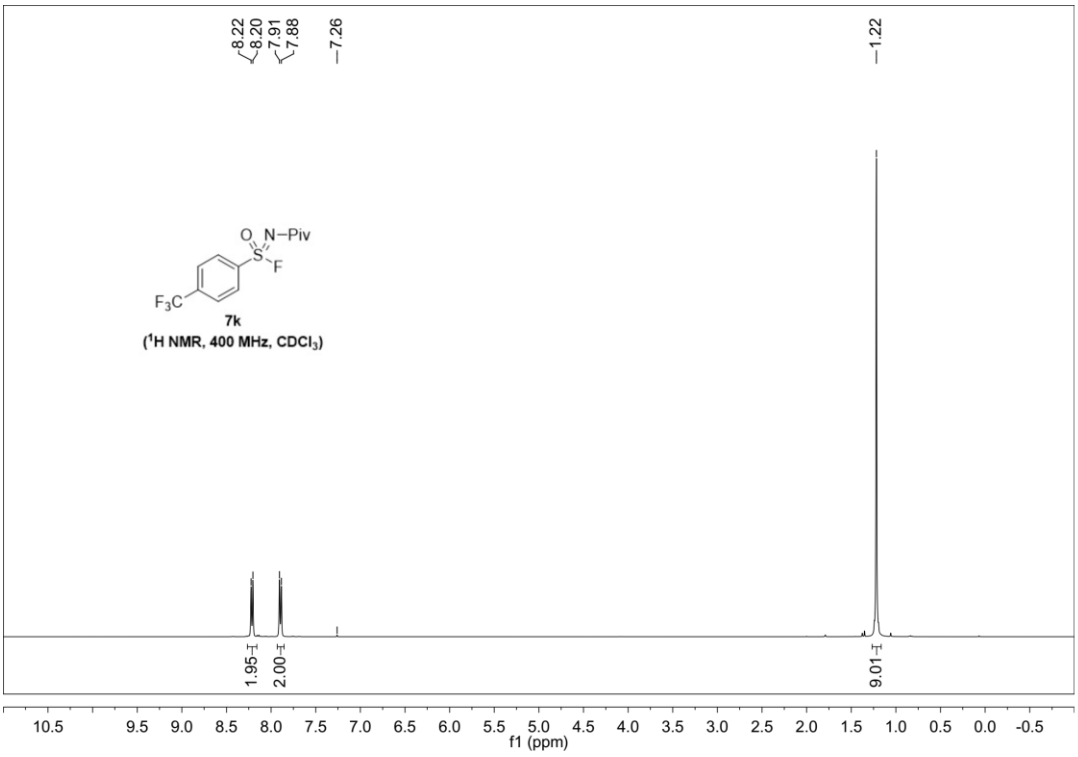
8.22
8.20
7.91
7.88
7.26
1.22
N−Piv
F
F3C
7k
(1H NMR, 400 MHz, CDCl3)
1.95
2.00
9.01
10.5 9.5 9.0 8.5 8.0 7.5 7.0 6.5 6.0 5.5 5.0 4.5 4.0 3.5 3.0 2.5 2.0 1.5 1.0 0.5 0.0 -0.5
f1 (ppm)

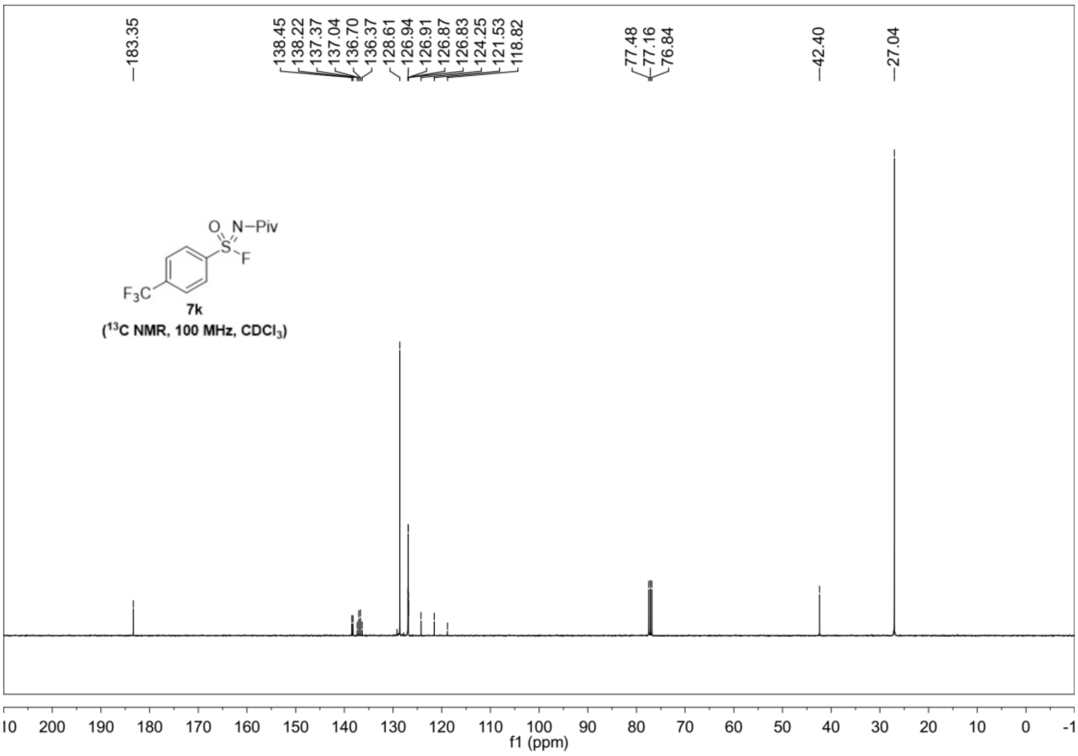

183.35
138.45
138.22
137.37
137.04
136.70
136.37
128.61
126.94
126.91
126.87
126.83
124.25
121.53
118.82
77.48
77.16
76.84
42.40
27.04
O
N−Piv
S
F
F_3C
7k
(^{13}C NMR, 100 MHz, $CDCl_3$)
f1 (ppm)

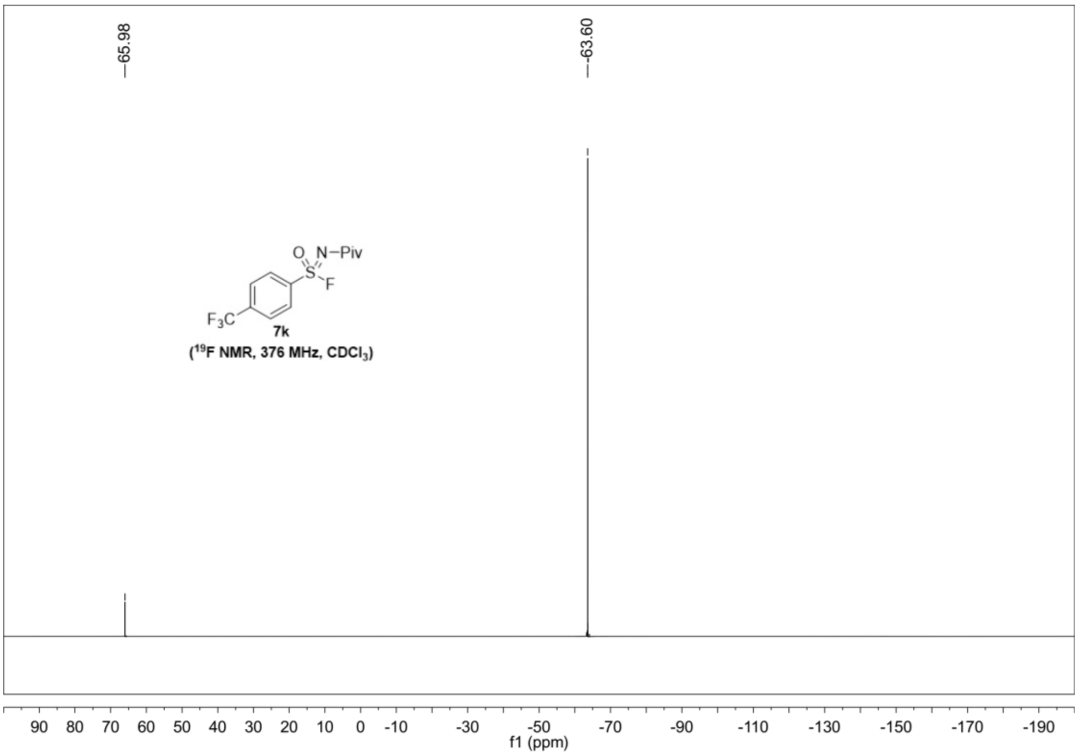

65.98
-63.60
O
N−Piv
S
F
F_3C
7k
(^{19}F NMR, 376 MHz, $CDCl_3$)
f1 (ppm)

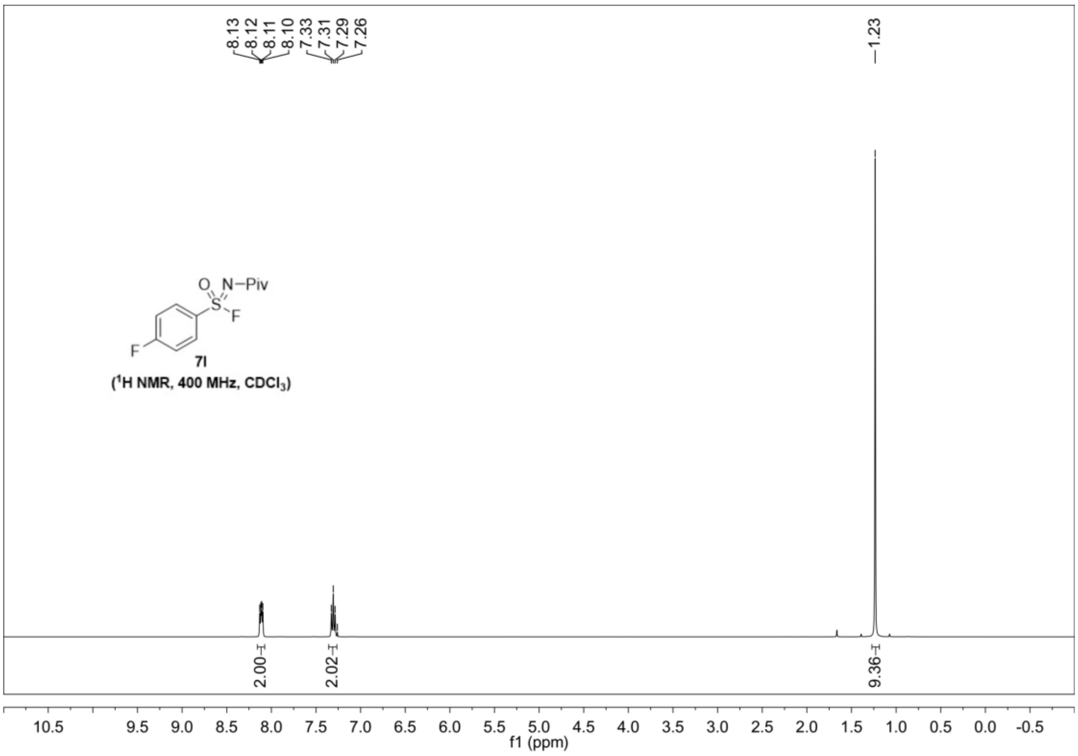
8.13
8.12
8.11
8.10
7.33
7.31
7.29
7.26
1.23
N–Piv
F
F
7l
(1H NMR, 400 MHz, CDCl3)
2.00
2.02
9.36
10.5 9.5 9.0 8.5 8.0 7.5 7.0 6.5 6.0 5.5 5.0 4.5 4.0 3.5 3.0 2.5 2.0 1.5 1.0 0.5 0.0 -0.5
f1 (ppm)

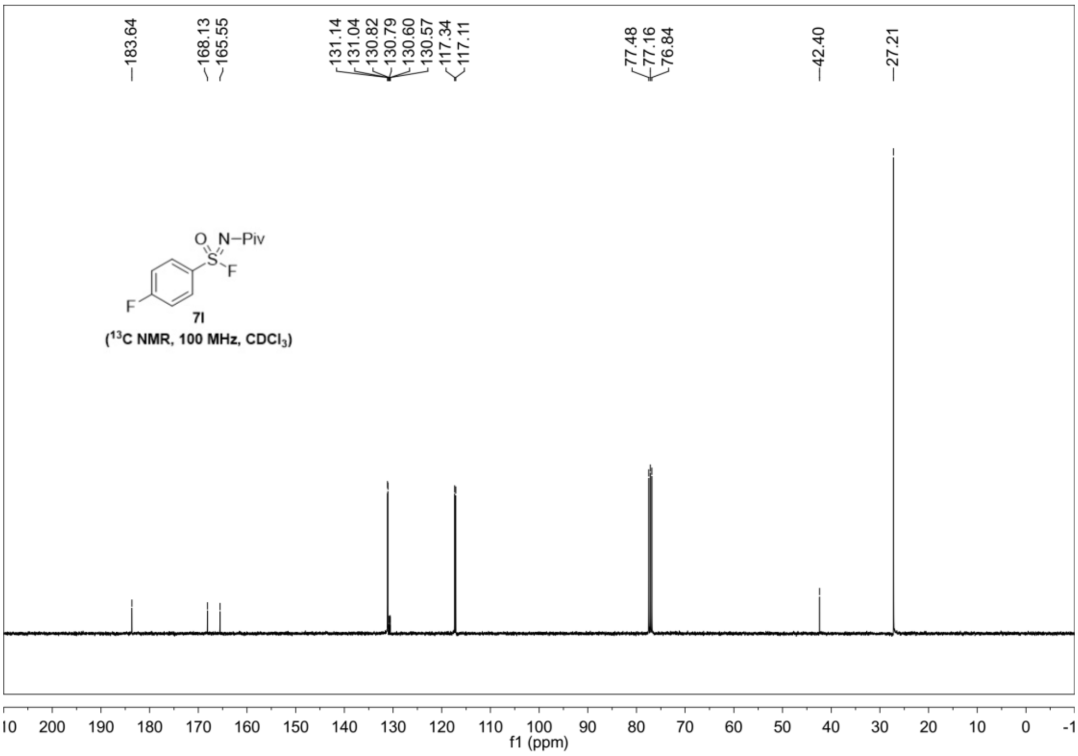
183.64
168.13
165.55
131.14
131.04
130.82
130.79
130.60
130.57
117.34
117.11
77.48
77.16
76.84
42.40
27.21
N–Piv
F
F
7l
(13C NMR, 100 MHz, CDCl3)
200 190 180 170 160 150 140 130 120 110 100 90 80 70 60 50 40 30 20 10 0
f1 (ppm)

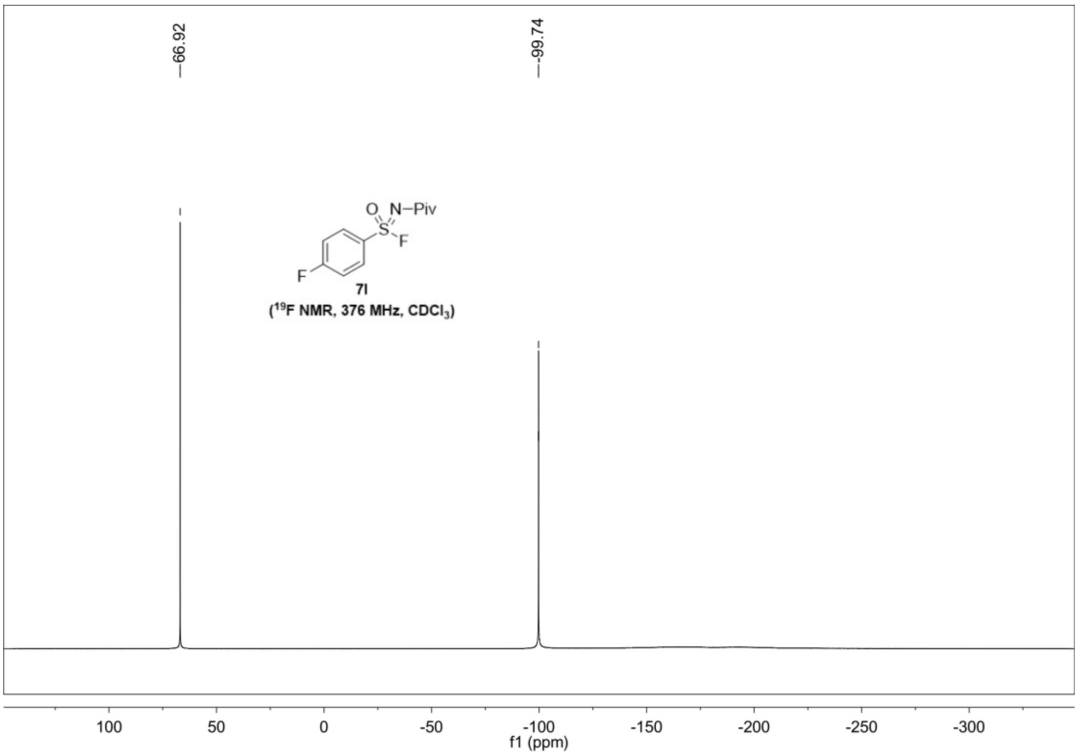

-66.92
-99.74
O N−Piv
S
F
F
7l
(^{19}F NMR, 376 MHz, CDCl$_3$)
100
50
0
-50
-100
-150
-200
-250
-300
f1 (ppm)

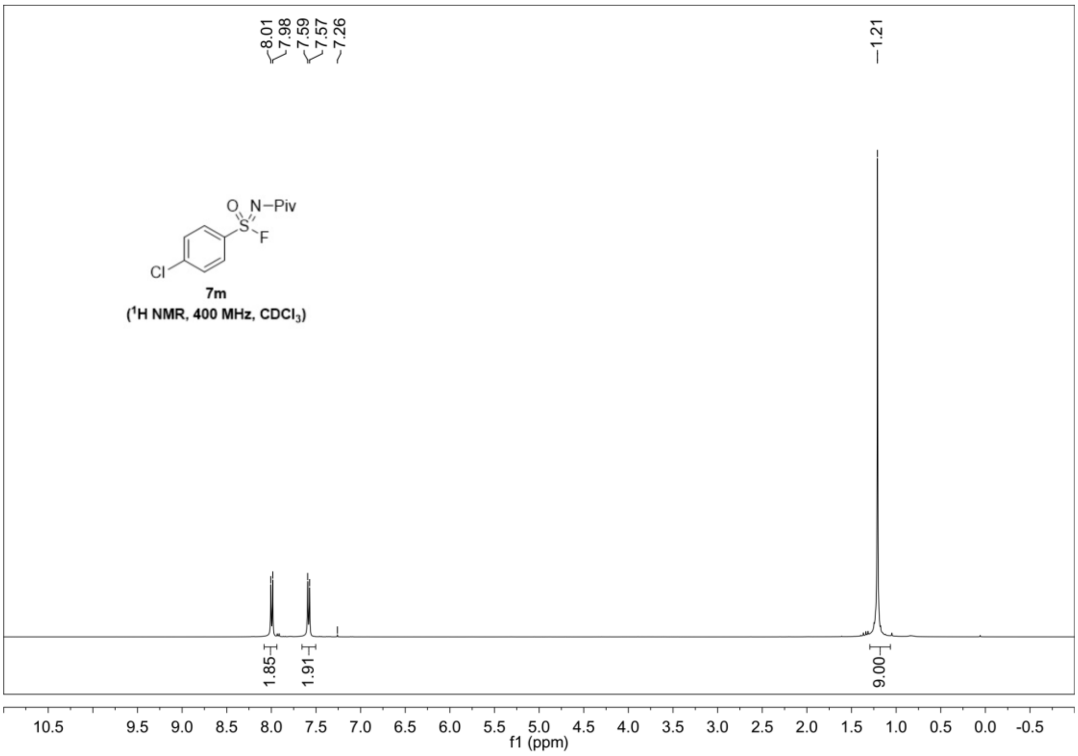

8.01
7.98
7.59
7.57
7.26
1.21
O N−Piv
S
F
Cl
7m
(^{1}H NMR, 400 MHz, CDCl$_3$)
1.85
1.91
9.00
10.5
9.5
9.0
8.5
8.0
7.5
7.0
6.5
6.0
5.5
5.0
4.5
4.0
3.5
3.0
2.5
2.0
1.5
1.0
0.5
0.0
-0.5
f1 (ppm)

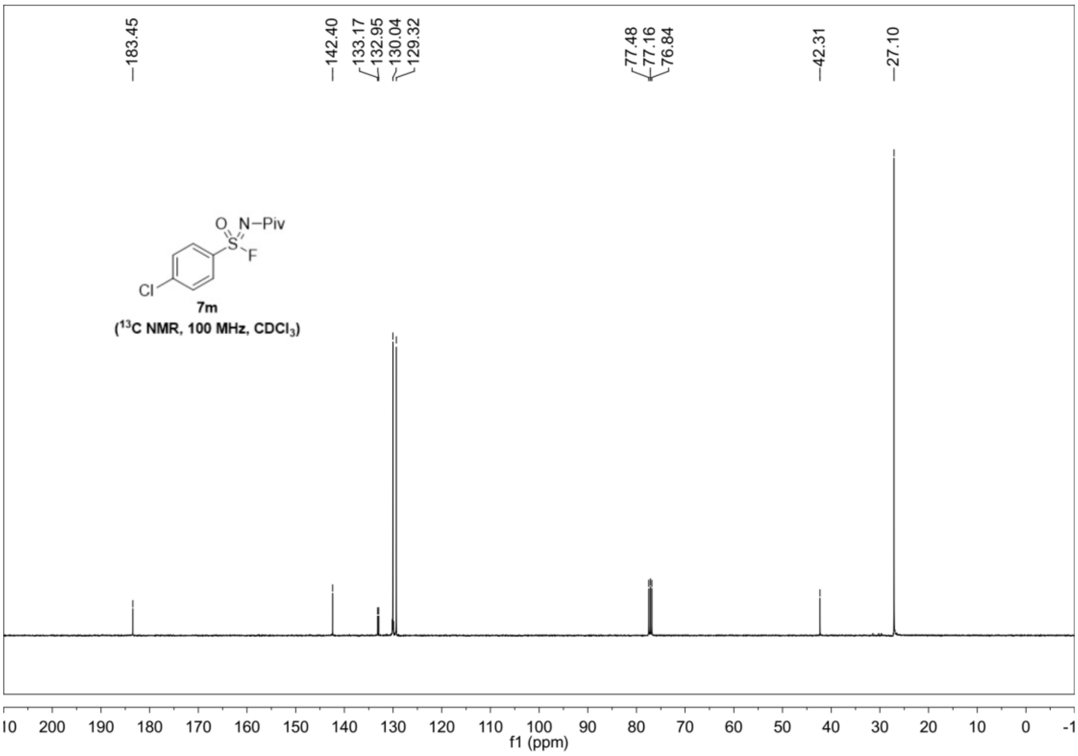
183.45
142.40
133.17
132.95
130.04
129.32
77.48
77.16
76.84
42.31
27.10
O N−Piv
S
F
Cl
7m
(13C NMR, 100 MHz, CDCl3)
f1 (ppm)

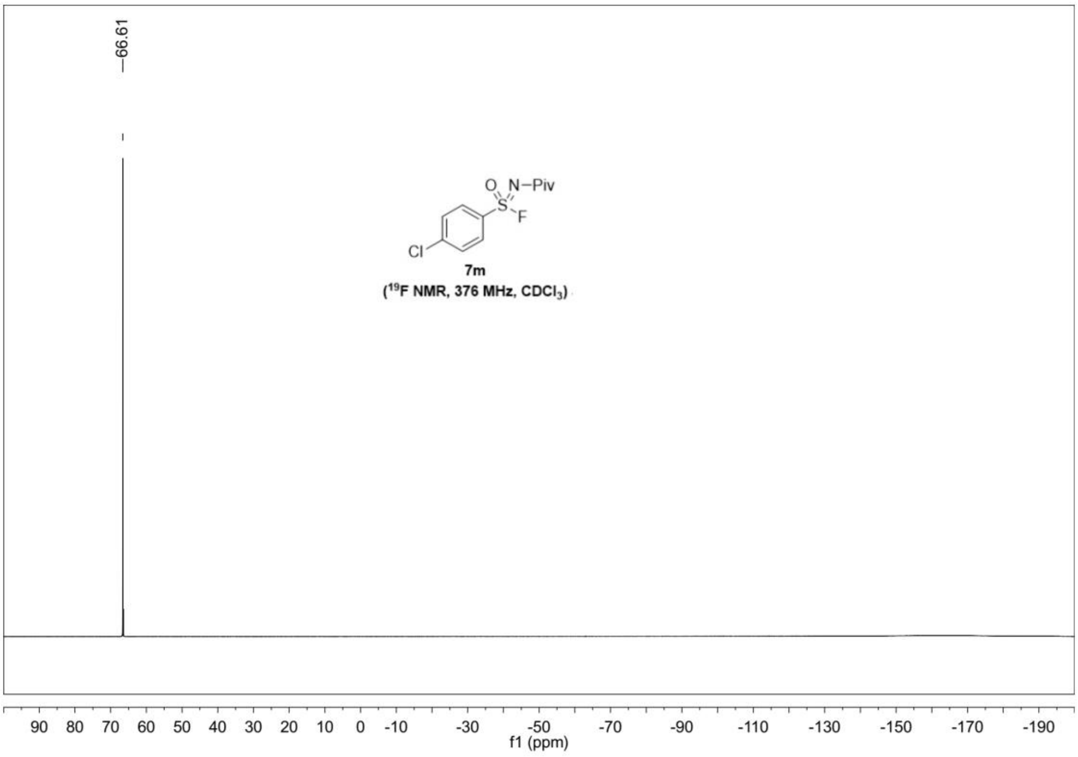
66.61
O N−Piv
S
F
Cl
7m
(19F NMR, 376 MHz, CDCl3)
f1 (ppm)

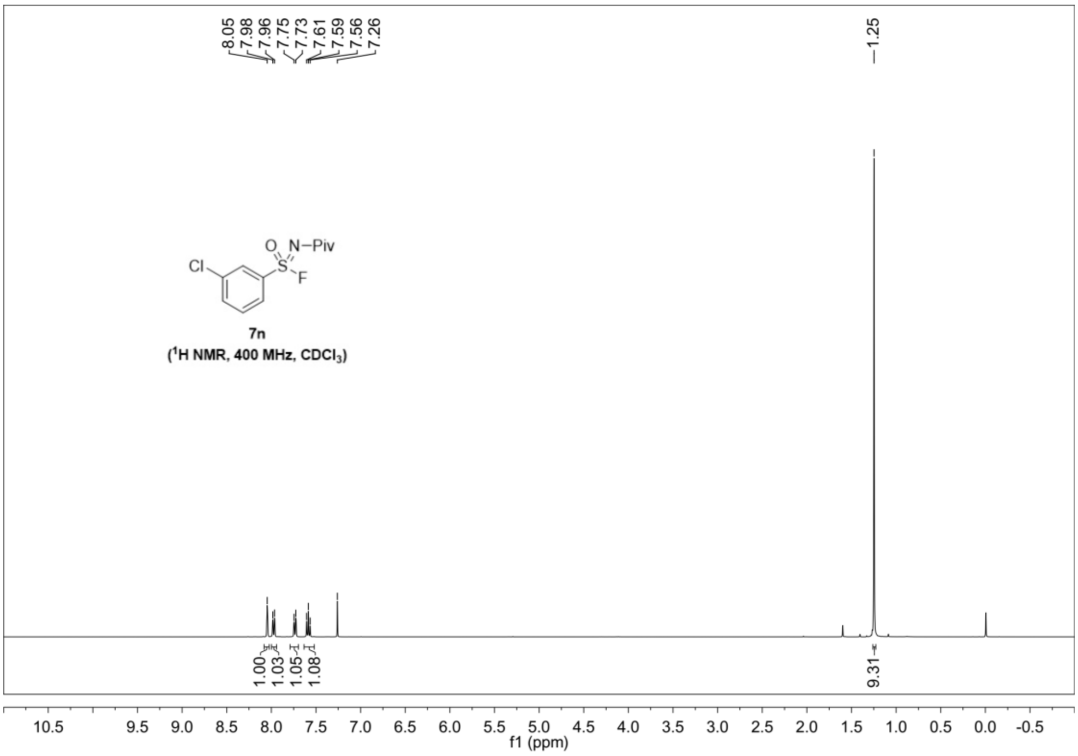
8.05
7.98
7.96
7.75
7.73
7.61
7.59
7.56
7.26
1.25
O N−Piv
Cl
S
F
7n
(¹H NMR, 400 MHz, CDCl3)
1.00
1.03
1.05
1.08
9.31
10.5 9.5 9.0 8.5 8.0 7.5 7.0 6.5 6.0 5.5 5.0 4.5 4.0 3.5 3.0 2.5 2.0 1.5 1.0 0.5 0.0 -0.5
f1 (ppm)

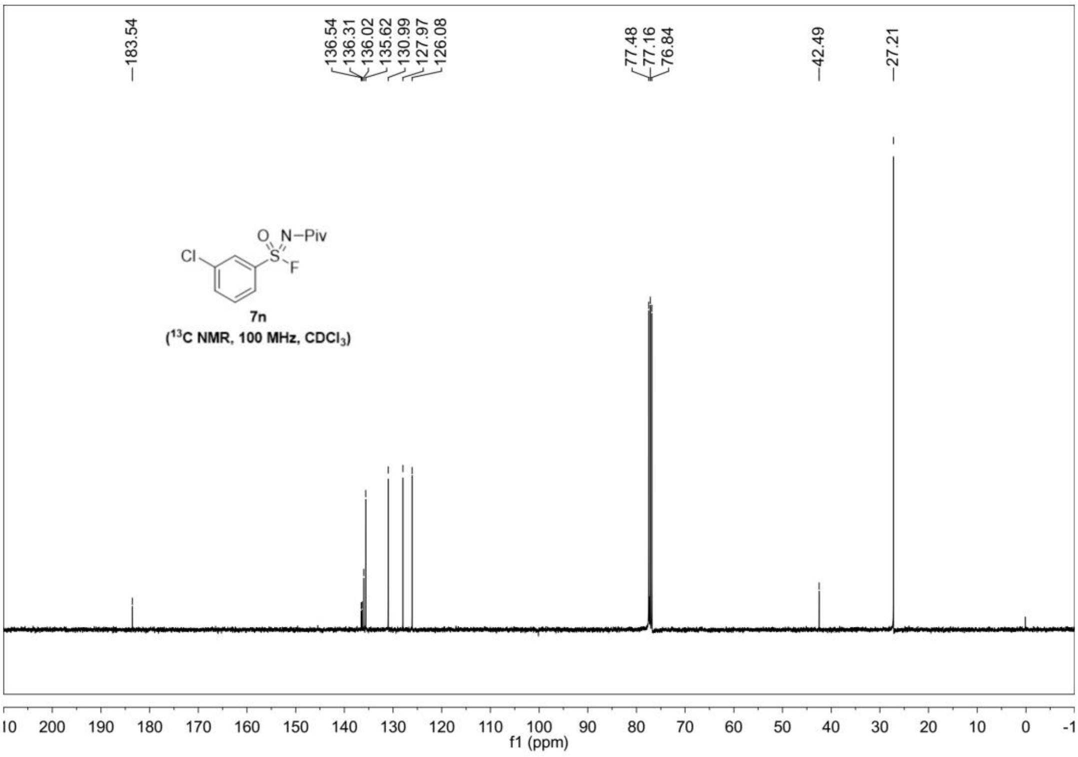
183.54
136.54
136.31
136.02
135.62
130.99
127.97
126.08
77.48
77.16
76.84
42.49
27.21
O N−Piv
Cl
S
F
7n
(¹³C NMR, 100 MHz, CDCl3)
200 190 180 170 160 150 140 130 120 110 100 90 80 70 60 50 40 30 20 10 0
f1 (ppm)

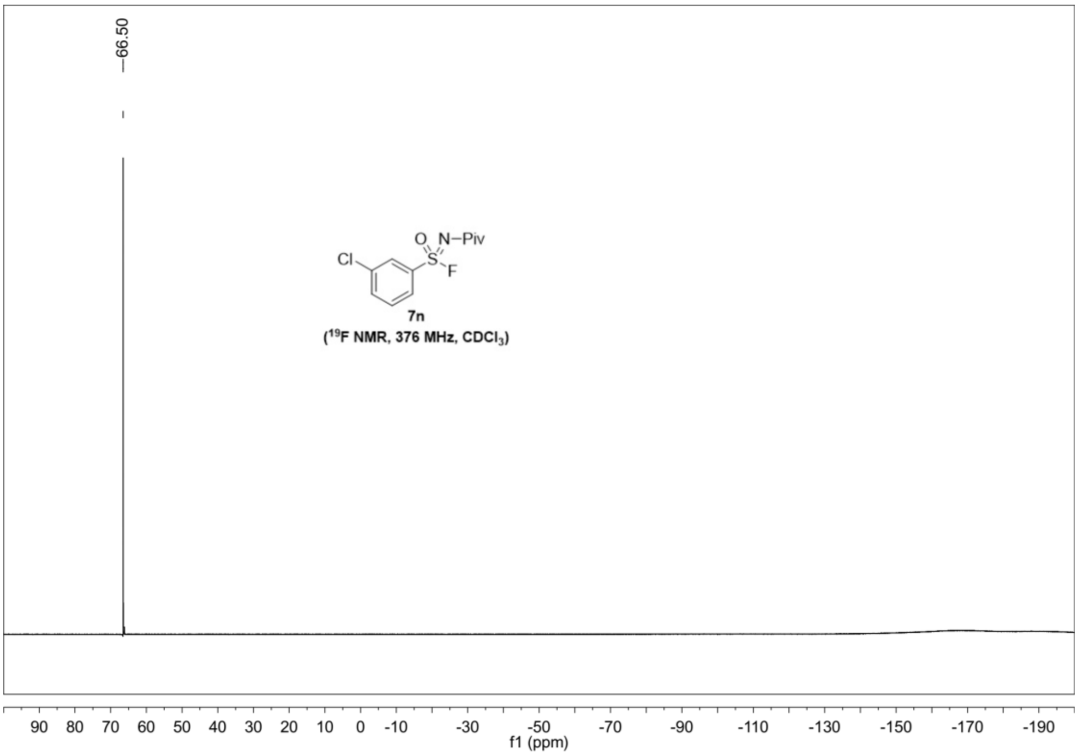

−66.50
Cl
O N−Piv
S
F
7n
(19F NMR, 376 MHz, CDCl3)
90 80 70 60 50 40 30 20 10 0 -10 -30 -50 -70 -90 -110 -130 -150 -170 -190
f1 (ppm)

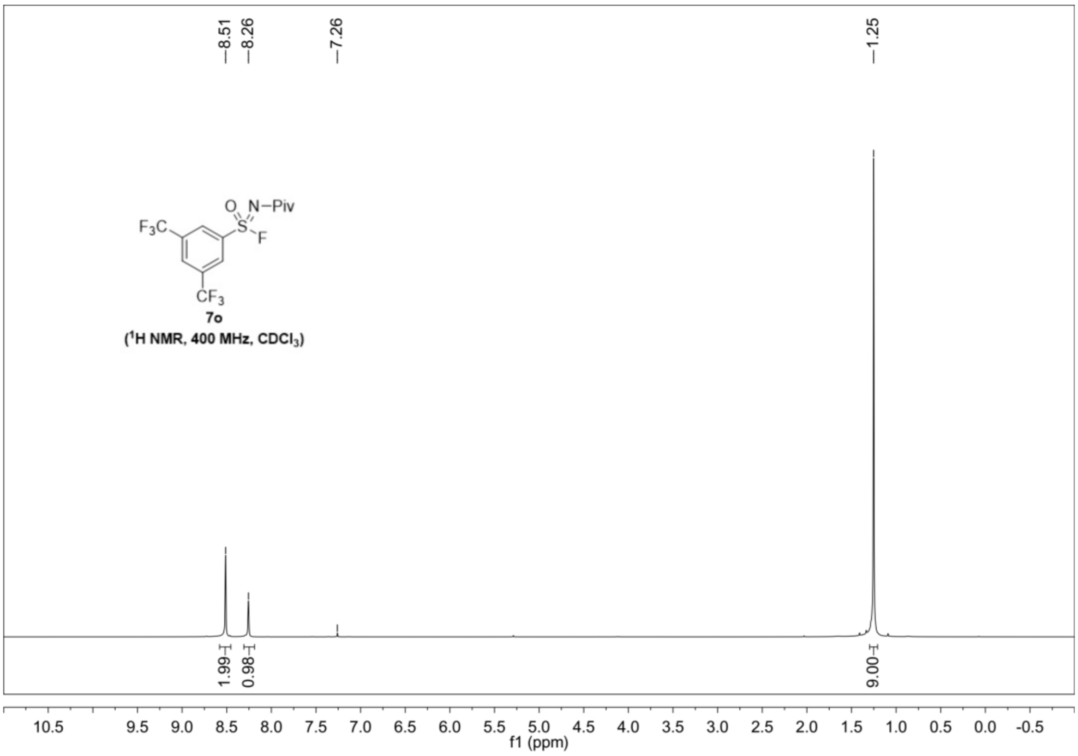

−8.51
−8.26
−7.26
−1.25
F3C
O N−Piv
S
F
CF3
7o
(1H NMR, 400 MHz, CDCl3)
1.99
0.98
9.00
10.5 9.5 9.0 8.5 8.0 7.5 7.0 6.5 6.0 5.5 5.0 4.5 4.0 3.5 3.0 2.5 2.0 1.5 1.0 0.5 0.0 -0.5
f1 (ppm)

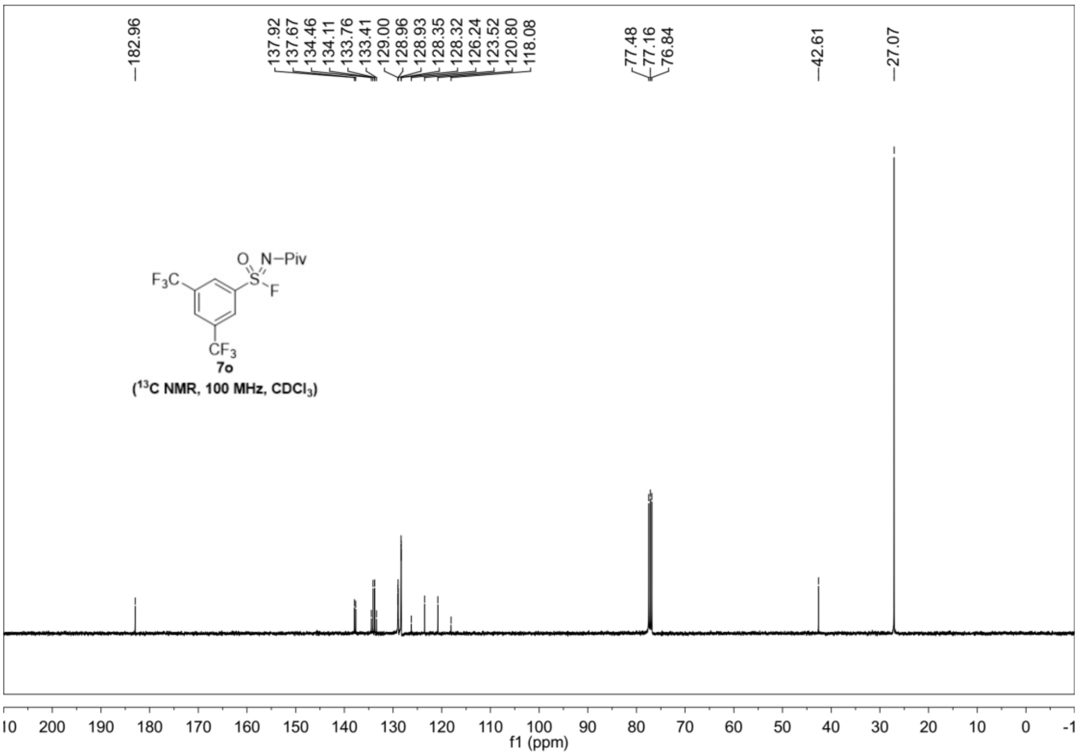

182.96
137.92
137.67
134.46
134.11
133.76
133.41
129.00
128.96
128.93
128.35
128.32
126.24
123.52
120.80
118.08
77.48
77.16
76.84
42.61
27.07
O N−Piv
F_3C
S
F
CF_3
7o
(^{13}C NMR, 100 MHz, $CDCl_3$)
f1 (ppm)

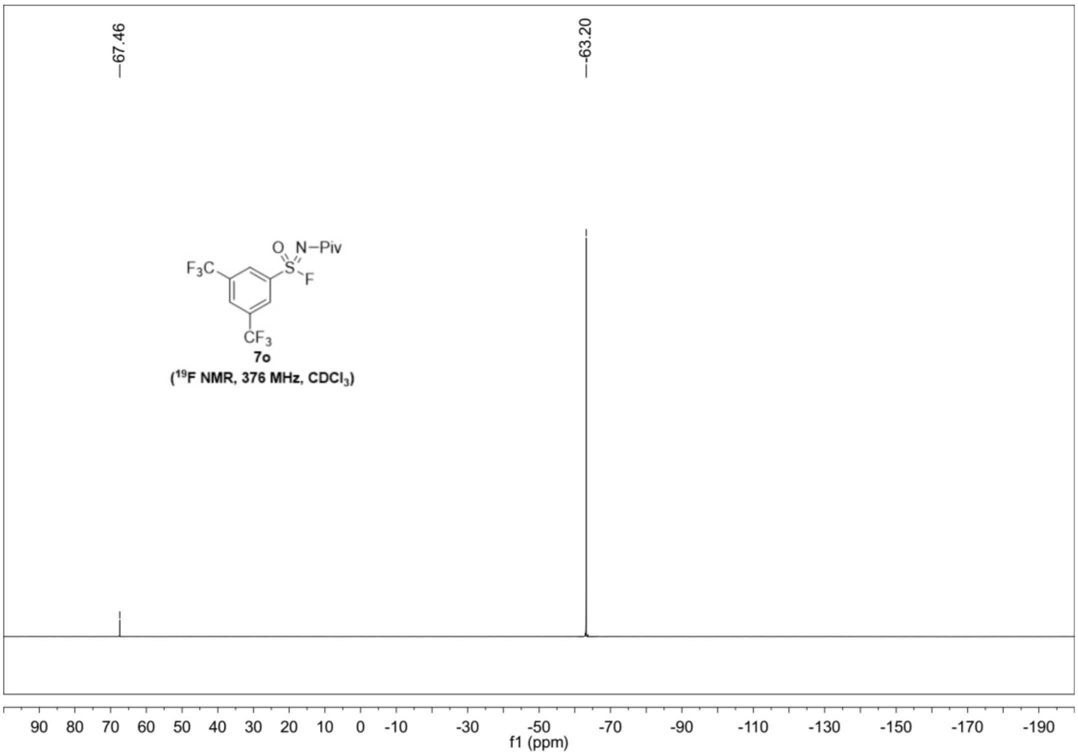

67.46
-63.20
O N−Piv
F_3C
S
F
CF_3
7o
(^{19}F NMR, 376 MHz, $CDCl_3$)
f1 (ppm)

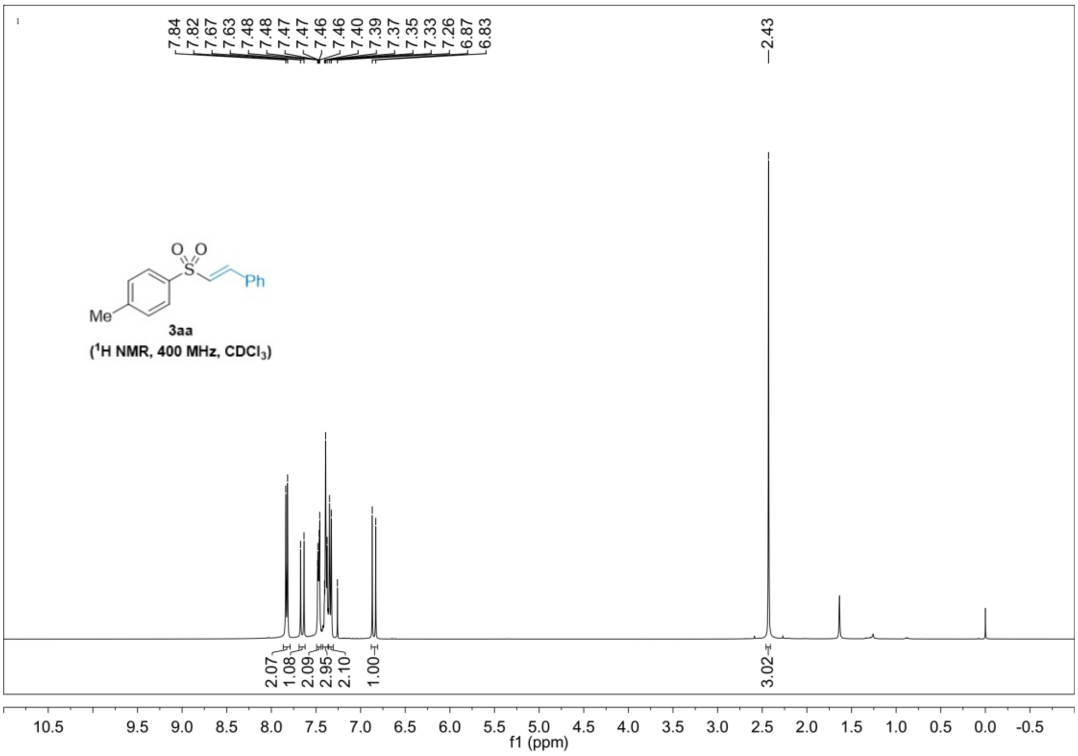
7.84
7.82
7.67
7.63
7.48
7.48
7.47
7.47
7.46
7.46
7.40
7.39
7.37
7.35
7.33
7.26
6.87
6.83
2.43
Me
Ph
3aa
(1H NMR, 400 MHz, CDCl3)
2.07
1.08
2.09
2.95
2.10
1.00
3.02
f1 (ppm)

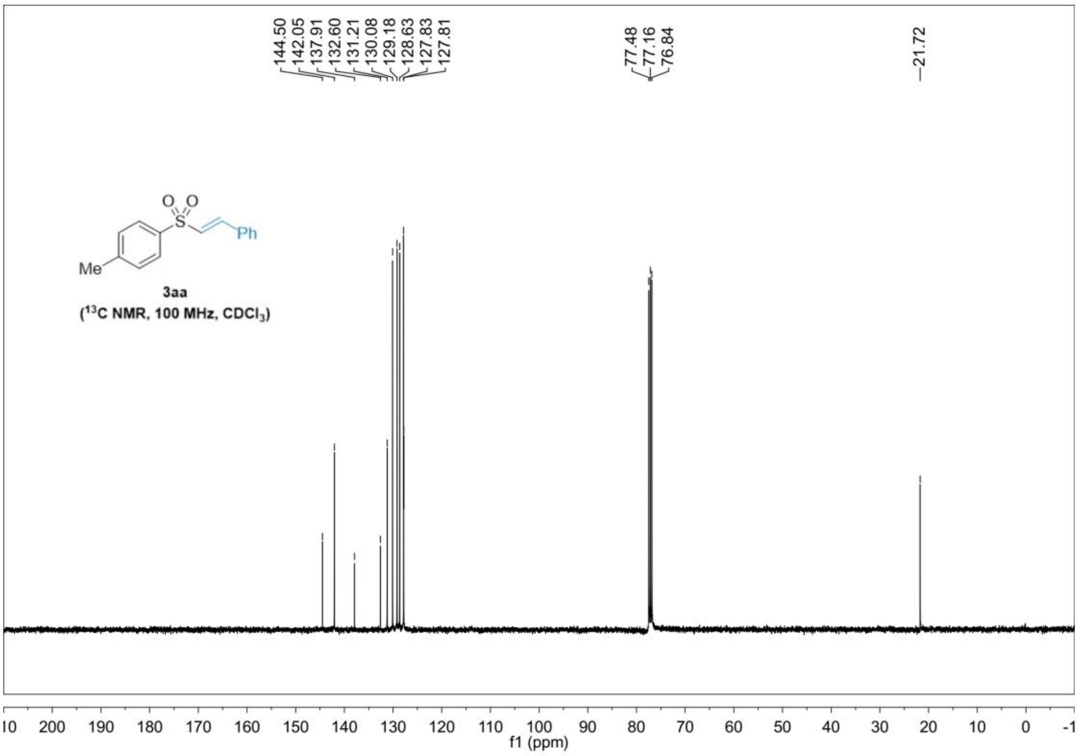
144.50
142.05
137.91
132.60
131.21
130.08
129.18
128.63
127.83
127.81
77.48
77.16
76.84
21.72
Me
Ph
3aa
(13C NMR, 100 MHz, CDCl3)
f1 (ppm)

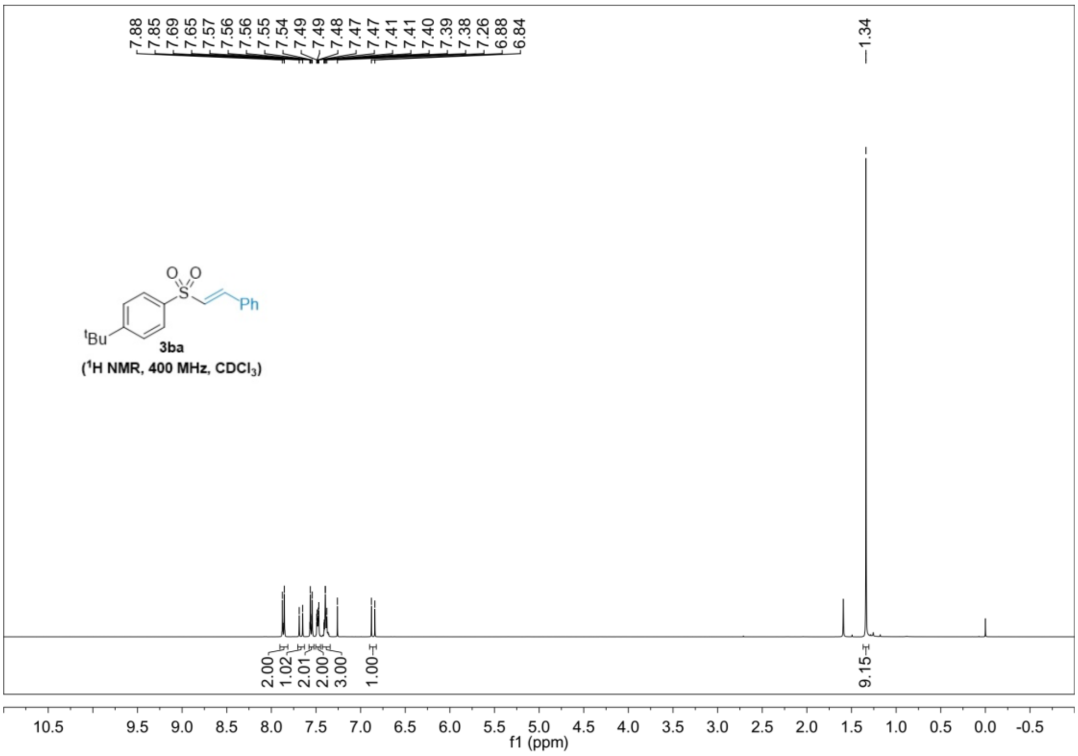

7.88
7.85
7.69
7.65
7.57
7.56
7.56
7.55
7.54
7.49
7.49
7.48
7.47
7.47
7.41
7.41
7.40
7.39
7.38
7.26
6.88
6.84
1.34
Ph
tBu
3ba
(1H NMR, 400 MHz, CDCl3)
2.00
1.02
2.01
2.00
3.00
1.00
9.15
f1 (ppm)

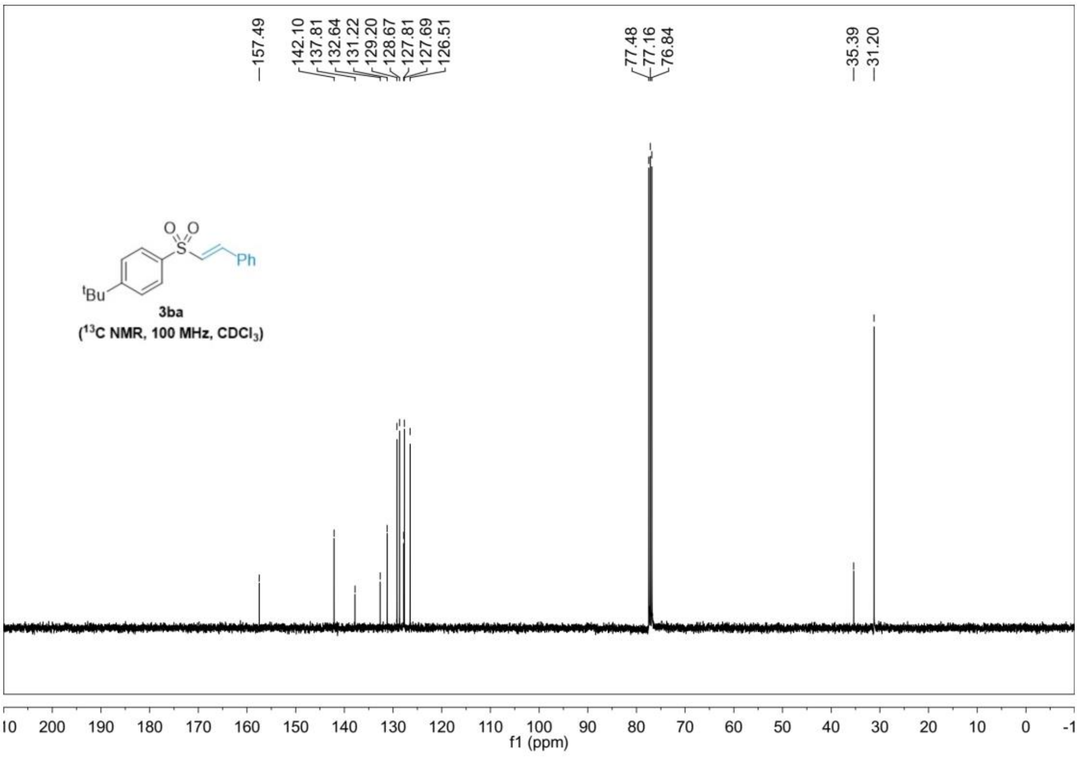

157.49
142.10
137.81
132.64
131.22
129.20
128.67
127.81
127.69
126.51
77.48
77.16
76.84
35.39
31.20
Ph
tBu
3ba
(13C NMR, 100 MHz, CDCl3)
f1 (ppm)

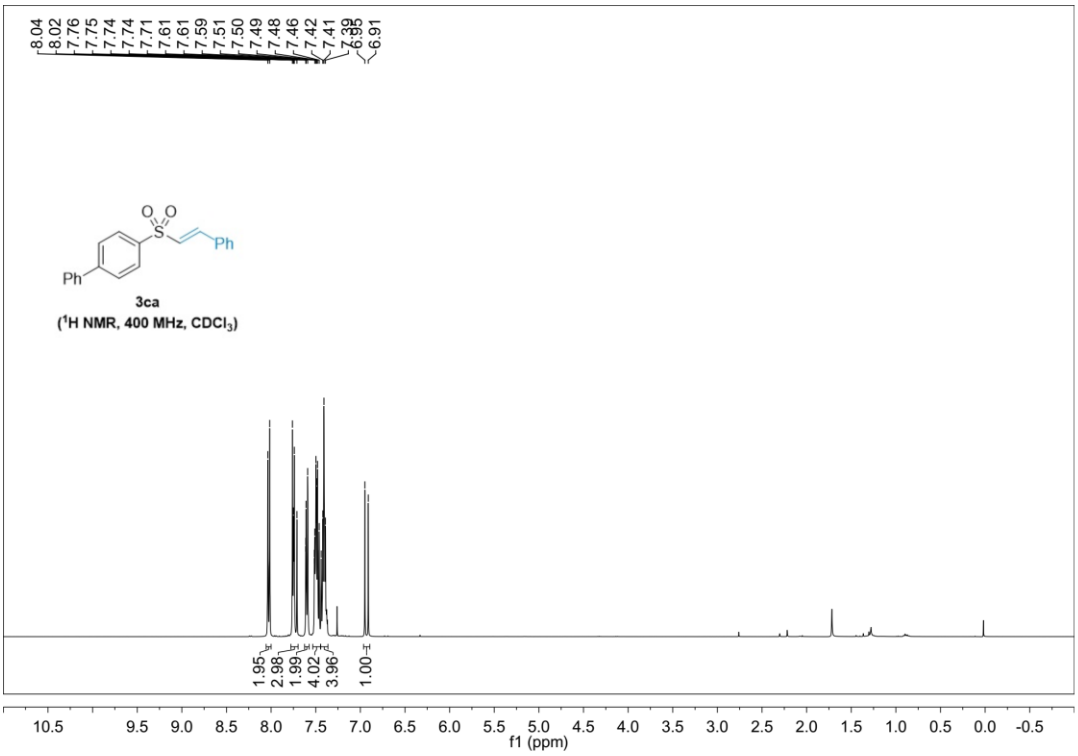
3ca
(1H NMR, 400 MHz, CDCl3)
f1 (ppm)

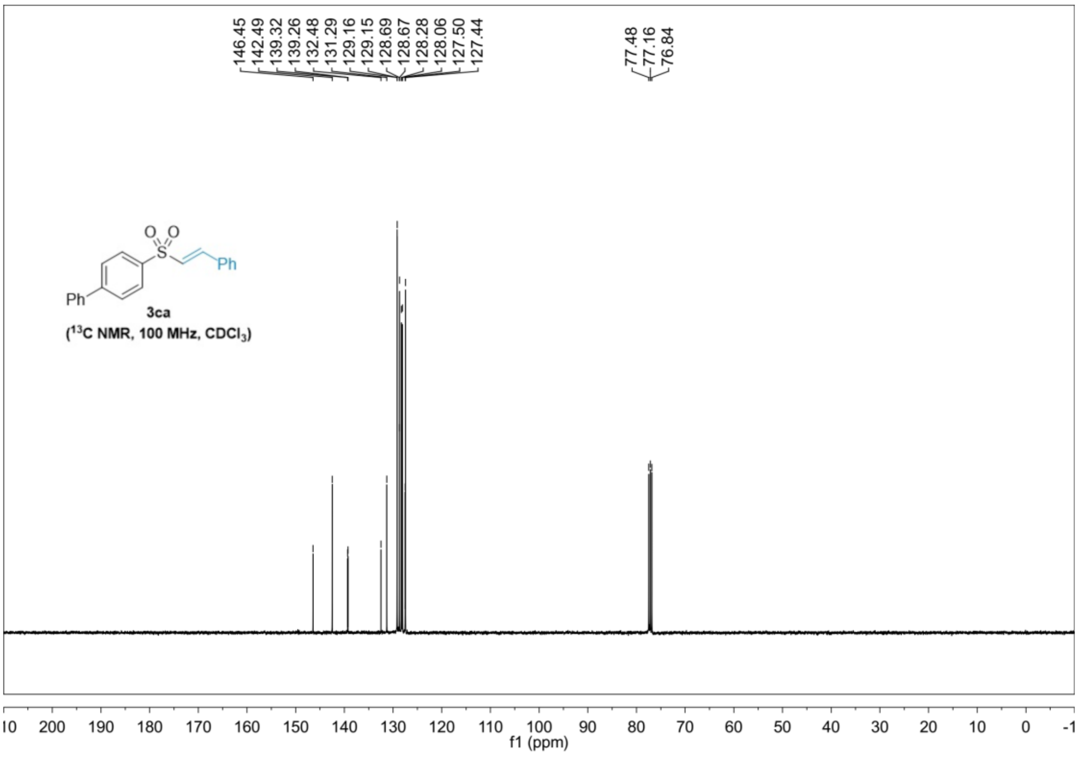
3ca
(13C NMR, 100 MHz, CDCl3)
f1 (ppm)

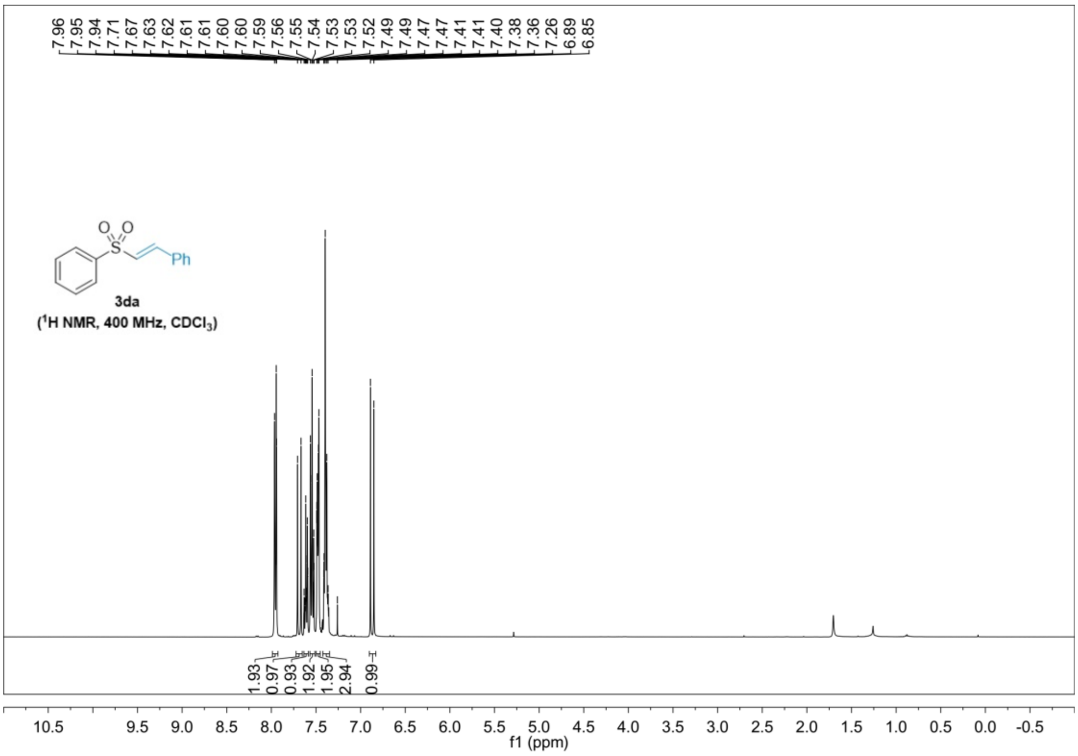
7.96
7.95
7.94
7.71
7.67
7.63
7.62
7.61
7.60
7.60
7.59
7.56
7.55
7.54
7.53
7.53
7.52
7.49
7.49
7.47
7.47
7.41
7.41
7.40
7.38
7.36
7.26
6.89
6.85
Ph
3da
(1H NMR, 400 MHz, CDCl3)
1.93
0.97
0.93
1.92
1.95
2.94
0.99
10.5
9.5
9.0
8.5
8.0
7.5
7.0
6.5
6.0
5.5
5.0
4.5
4.0
3.5
3.0
2.5
2.0
1.5
1.0
0.5
0.0
-0.5
f1 (ppm)

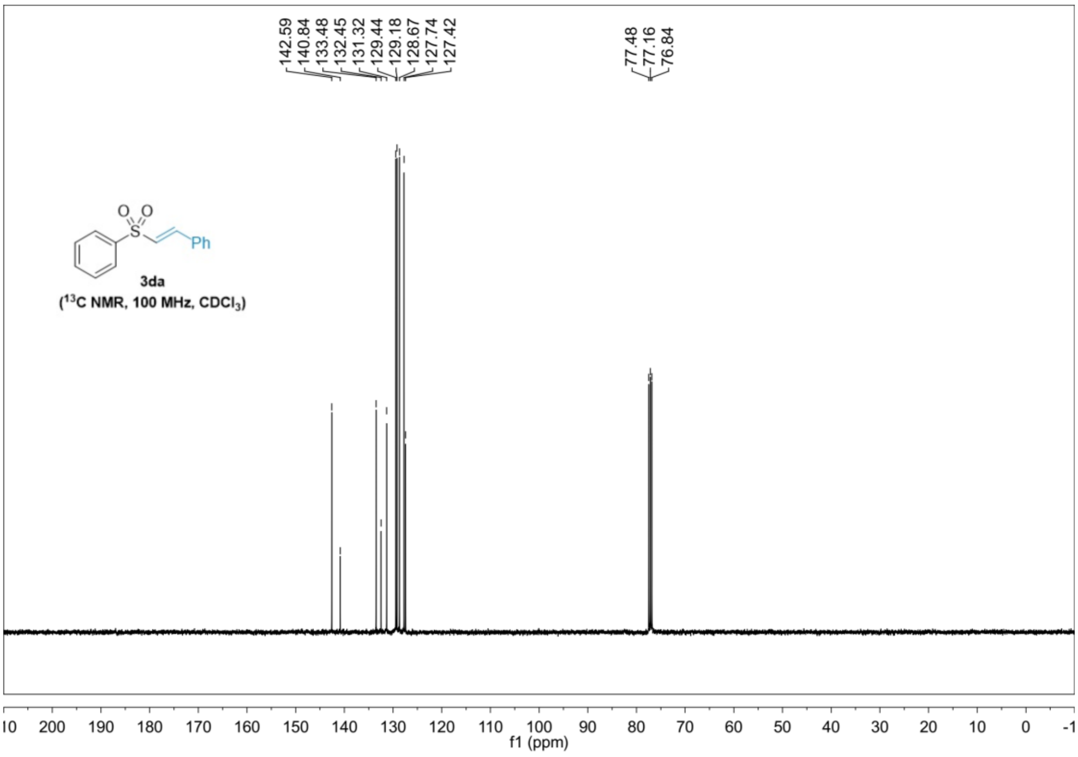
142.59
140.84
133.48
132.45
131.32
129.44
129.18
128.67
127.74
127.42
77.48
77.16
76.84
Ph
3da
(13C NMR, 100 MHz, CDCl3)
200
190
180
170
160
150
140
130
120
110
100
90
80
70
60
50
40
30
20
10
0
f1 (ppm)

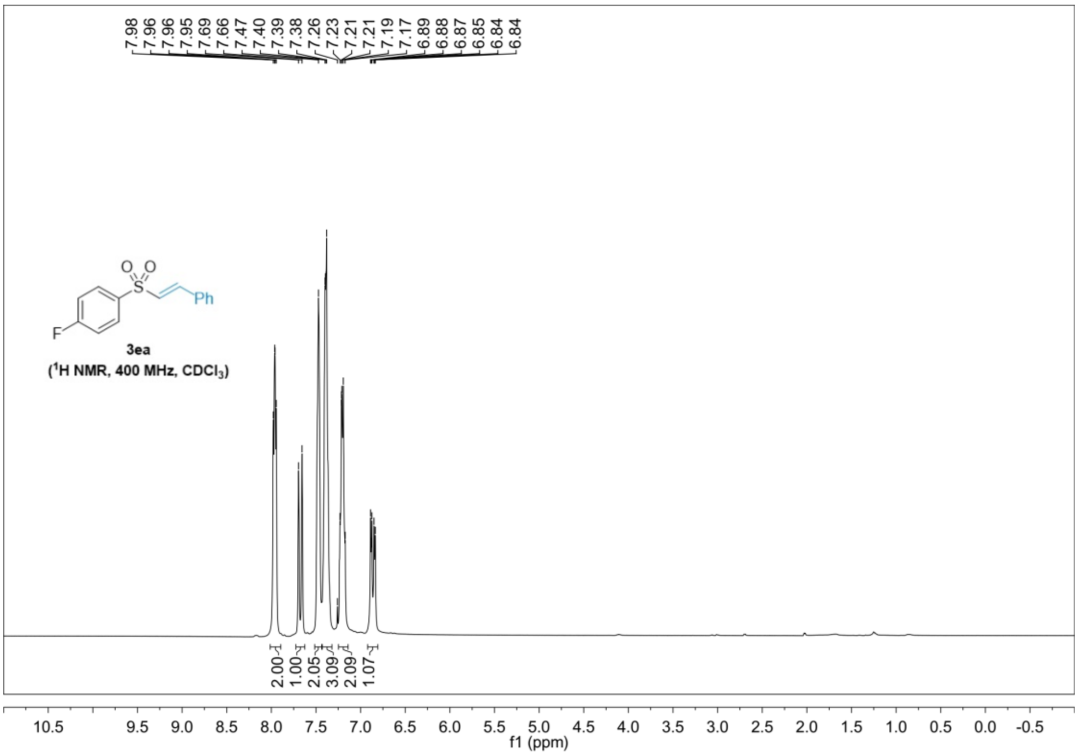
7.98
7.96
7.96
7.95
7.69
7.66
7.47
7.40
7.39
7.38
7.26
7.23
7.21
7.21
7.19
7.17
6.89
6.88
6.87
6.85
6.84
6.84
3ea
(¹H NMR, 400 MHz, CDCl₃)
2.00
1.00
2.05
3.09
2.09
1.07
f1 (ppm)

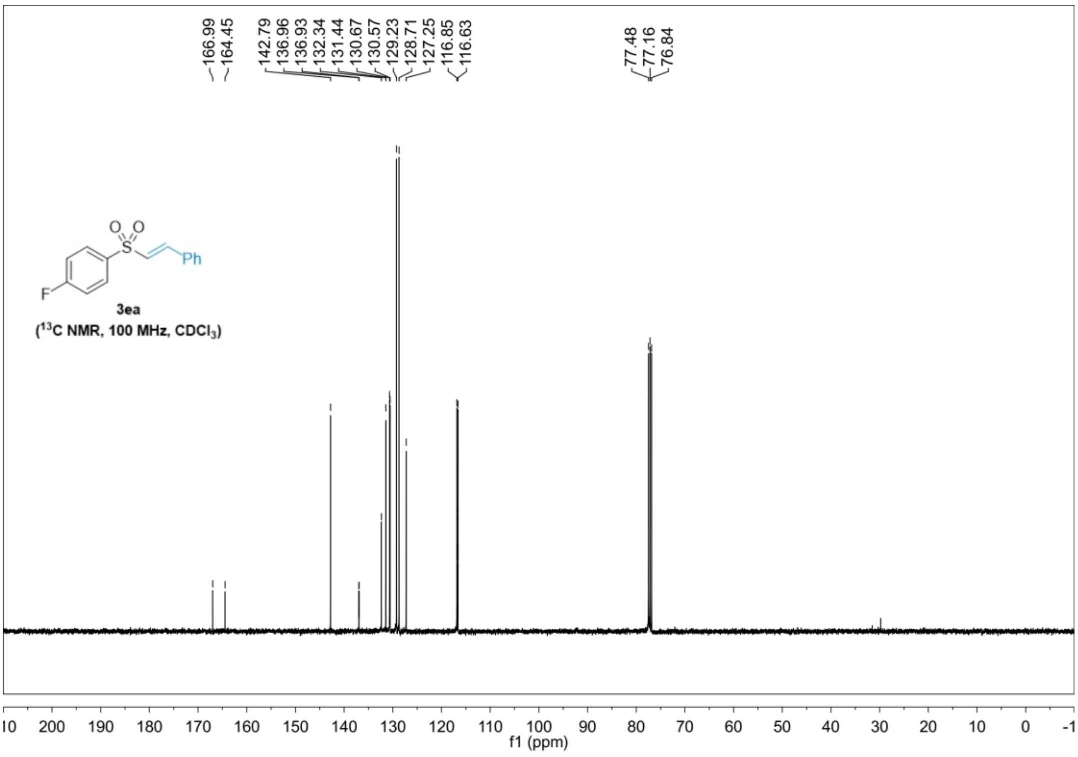
166.99
164.45
142.79
136.96
136.93
132.34
131.44
130.67
130.57
129.23
128.71
127.25
116.85
116.63
77.48
77.16
76.84
3ea
(¹³C NMR, 100 MHz, CDCl₃)
f1 (ppm)

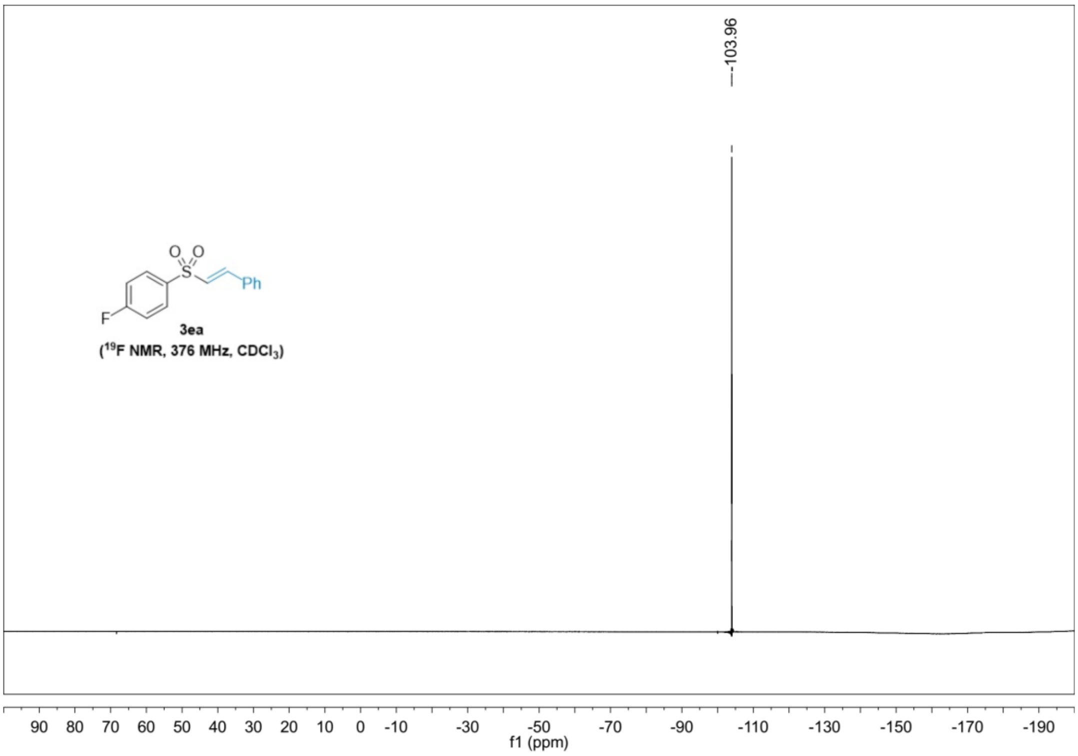

-103.96
F
Ph
3ea
(19F NMR, 376 MHz, CDCl3)
90 80 70 60 50 40 30 20 10 0 -10 -30 -50 -70 -90 -110 -130 -150 -170 -190
f1 (ppm)

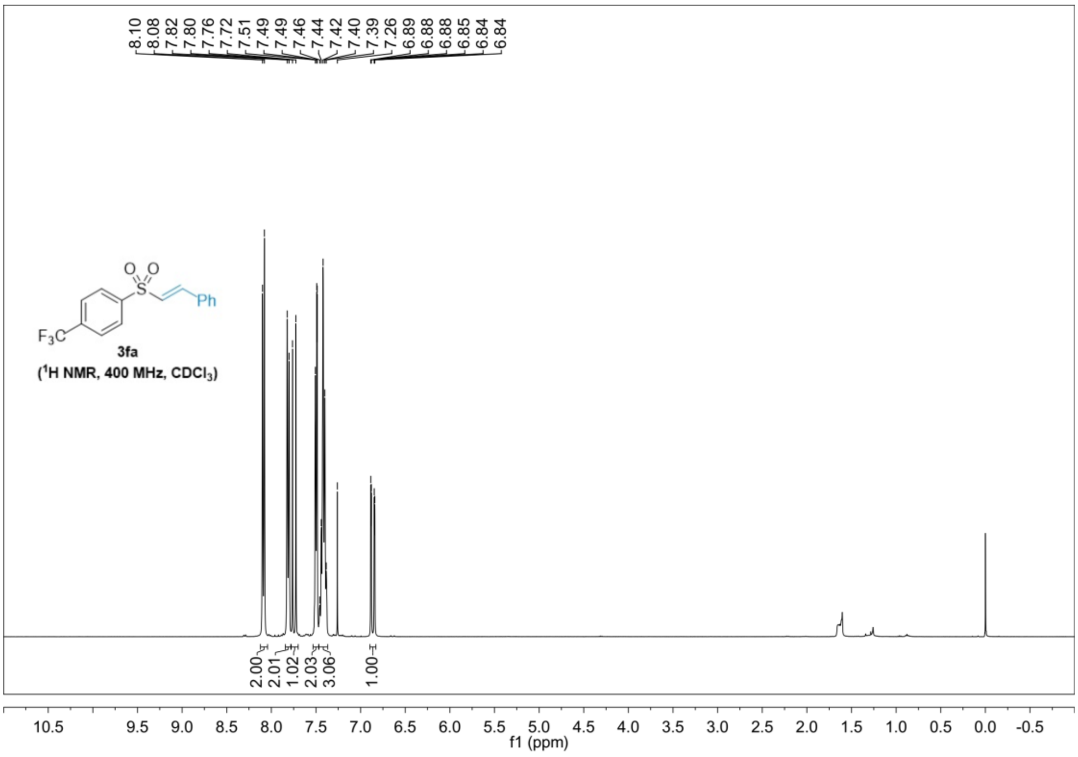

8.10 8.08 7.82 7.80 7.76 7.72 7.51 7.49 7.49 7.46 7.44 7.42 7.40 7.39 7.26 6.89 6.88 6.88 6.85 6.84 6.84
F3C
Ph
3fa
(1H NMR, 400 MHz, CDCl3)
2.00 2.01 1.02 2.03 3.06 1.00
10.5 9.5 9.0 8.5 8.0 7.5 7.0 6.5 6.0 5.5 5.0 4.5 4.0 3.5 3.0 2.5 2.0 1.5 1.0 0.5 0.0 -0.5
f1 (ppm)

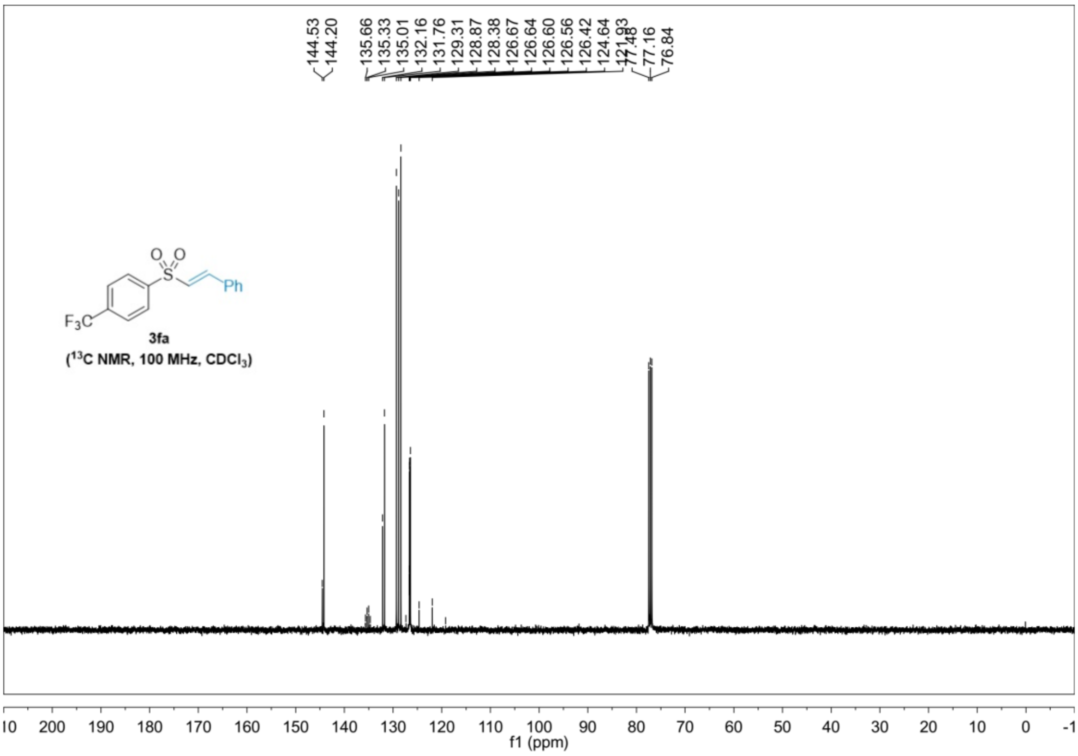
144.53
144.20
135.66
135.33
135.01
132.16
131.76
129.31
128.87
128.38
126.67
126.64
126.60
126.56
126.42
124.64
121.93
77.48
77.16
76.84
F3C
S
O O
Ph
3fa
(13C NMR, 100 MHz, CDCl3)
f1 (ppm)

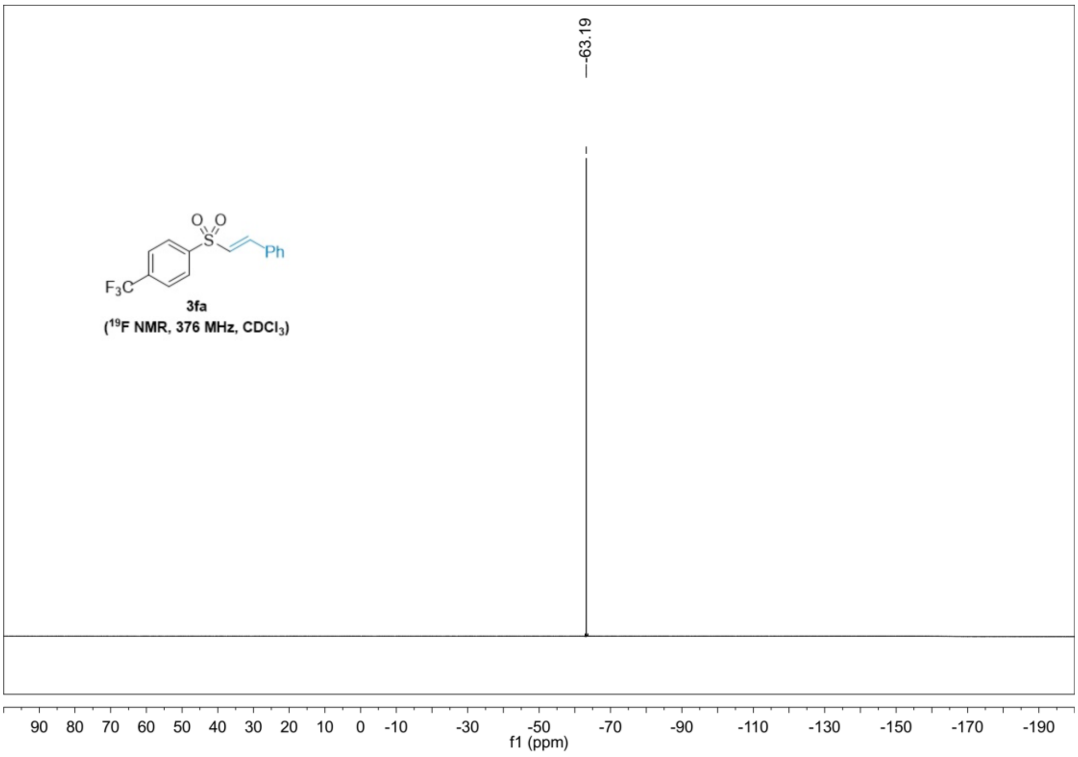
-63.19
F3C
S
O O
Ph
3fa
(19F NMR, 376 MHz, CDCl3)
f1 (ppm)

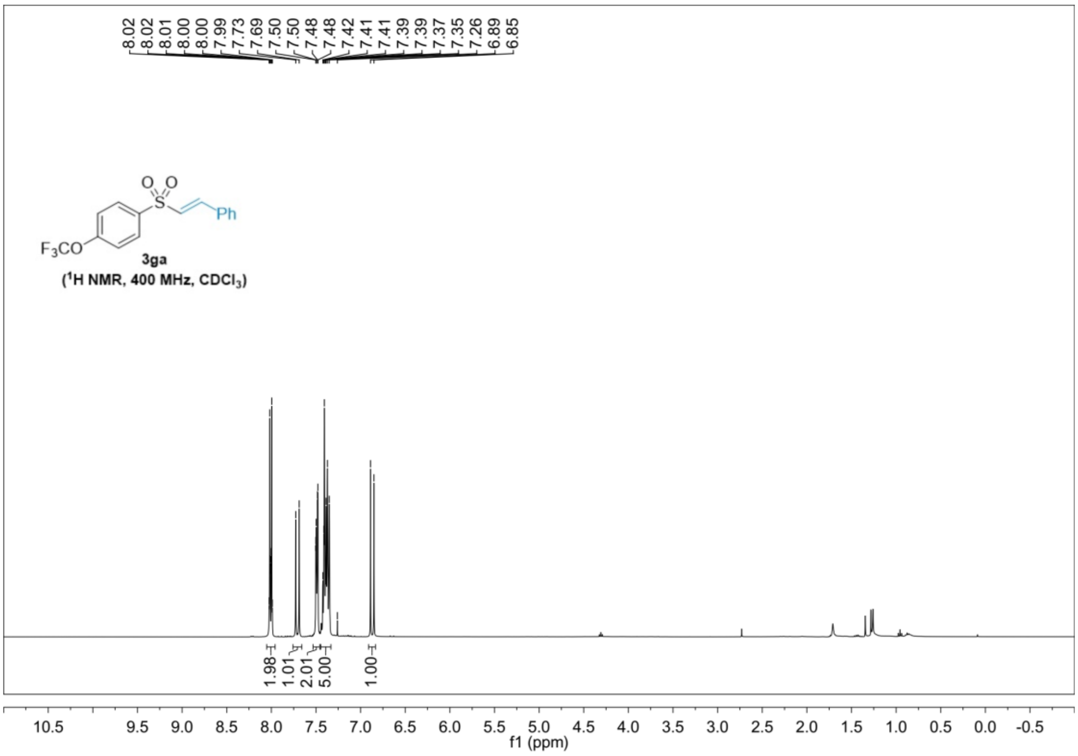
8.02
8.02
8.01
8.00
7.99
7.73
7.69
7.50
7.48
7.48
7.42
7.41
7.41
7.39
7.37
7.35
7.26
6.89
6.85
F3CO
Ph
3ga
(1H NMR, 400 MHz, CDCl3)
1.98
1.01
2.01
5.00
1.00
10.5 9.5 9.0 8.5 8.0 7.5 7.0 6.5 6.0 5.5 5.0 4.5 4.0 3.5 3.0 2.5 2.0 1.5 1.0 0.5 0.0 -0.5
f1 (ppm)

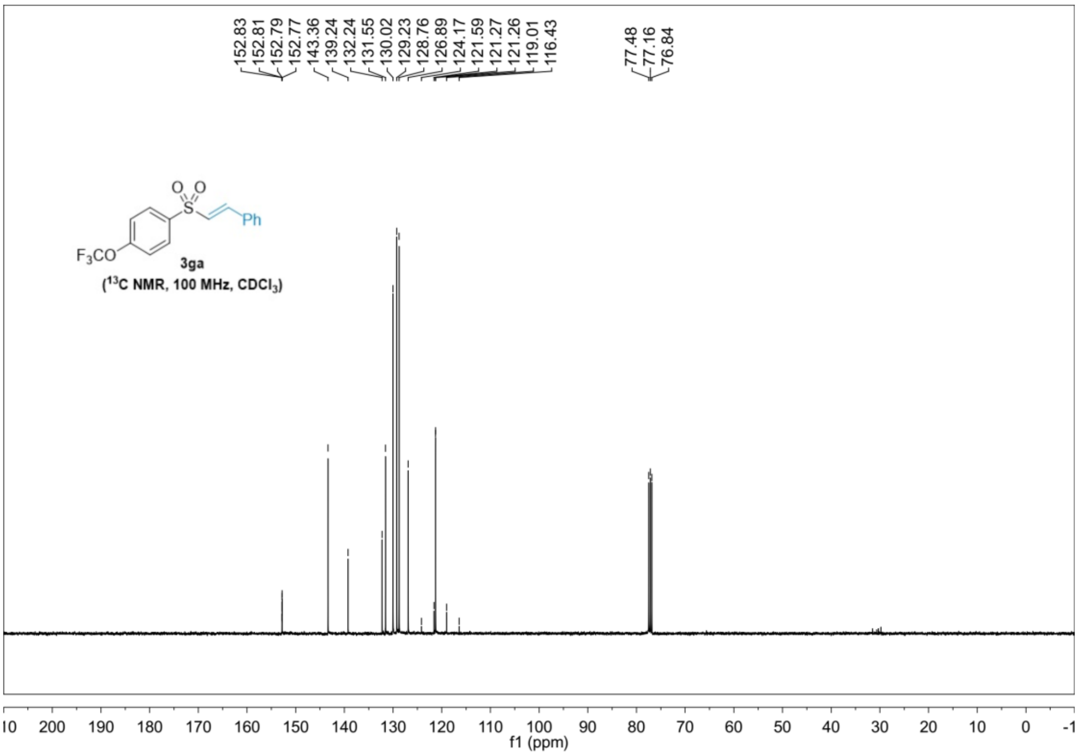
152.83
152.81
152.79
152.77
143.36
139.24
132.24
131.55
130.02
129.23
128.76
126.89
124.17
121.59
121.27
121.26
119.01
116.43
77.48
77.16
76.84
F3CO
Ph
3ga
(13C NMR, 100 MHz, CDCl3)
200 190 180 170 160 150 140 130 120 110 100 90 80 70 60 50 40 30 20 10 0
f1 (ppm)

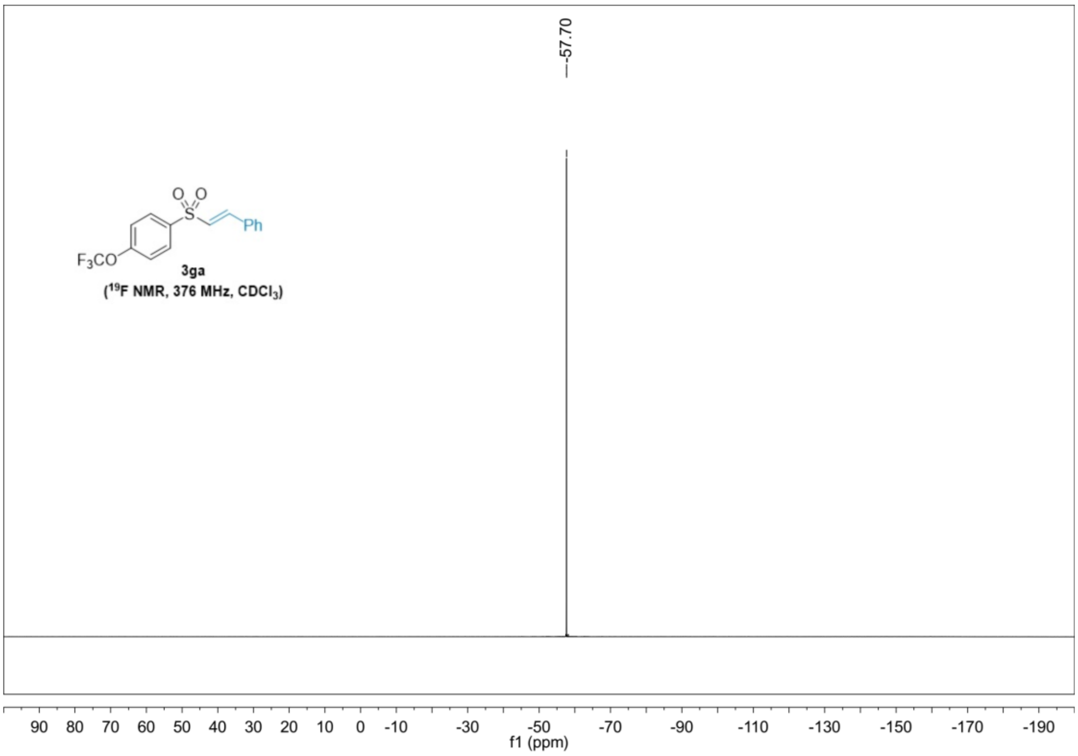

-57.70
F3CO
Ph
3ga
(19F NMR, 376 MHz, CDCl3)
90 80 70 60 50 40 30 20 10 0 -10 -30 -50 -70 -90 -110 -130 -150 -170 -190
f1 (ppm)

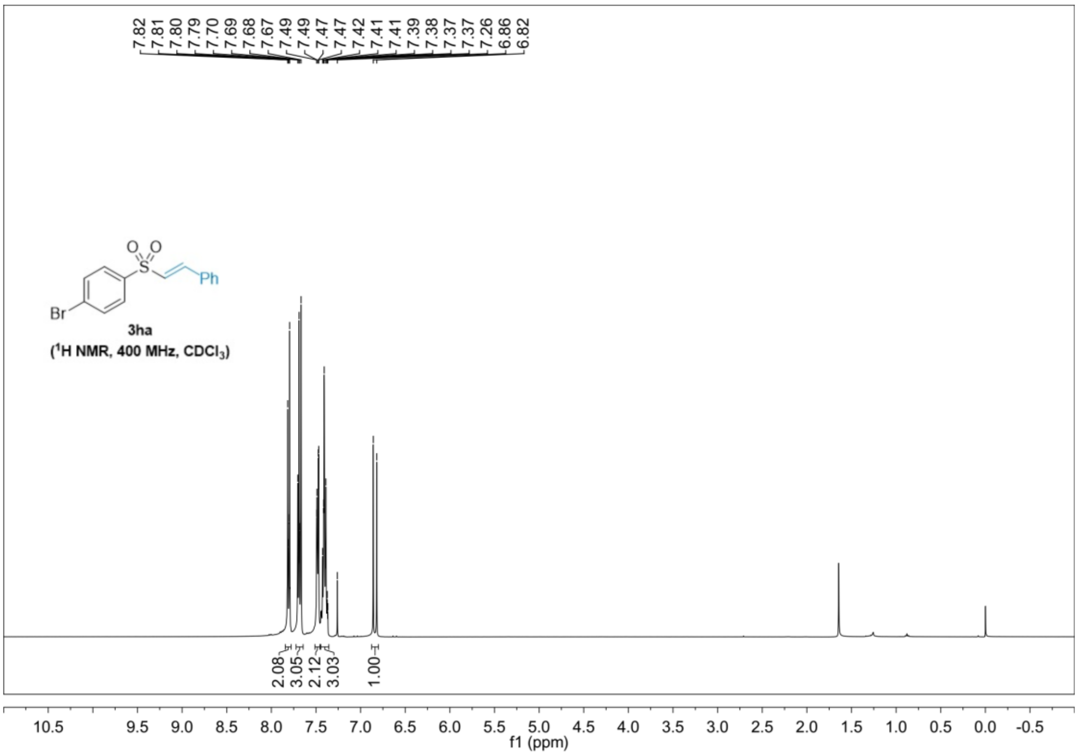

7.82 7.81 7.80 7.79 7.70 7.69 7.68 7.67 7.49 7.49 7.47 7.42 7.41 7.39 7.38 7.37 7.37 7.26 6.86 6.82
Br
Ph
3ha
(1H NMR, 400 MHz, CDCl3)
2.08 3.05 2.12 3.03 1.00
10.5 9.5 9.0 8.5 8.0 7.5 7.0 6.5 6.0 5.5 5.0 4.5 4.0 3.5 3.0 2.5 2.0 1.5 1.0 0.5 0.0 -0.5
f1 (ppm)

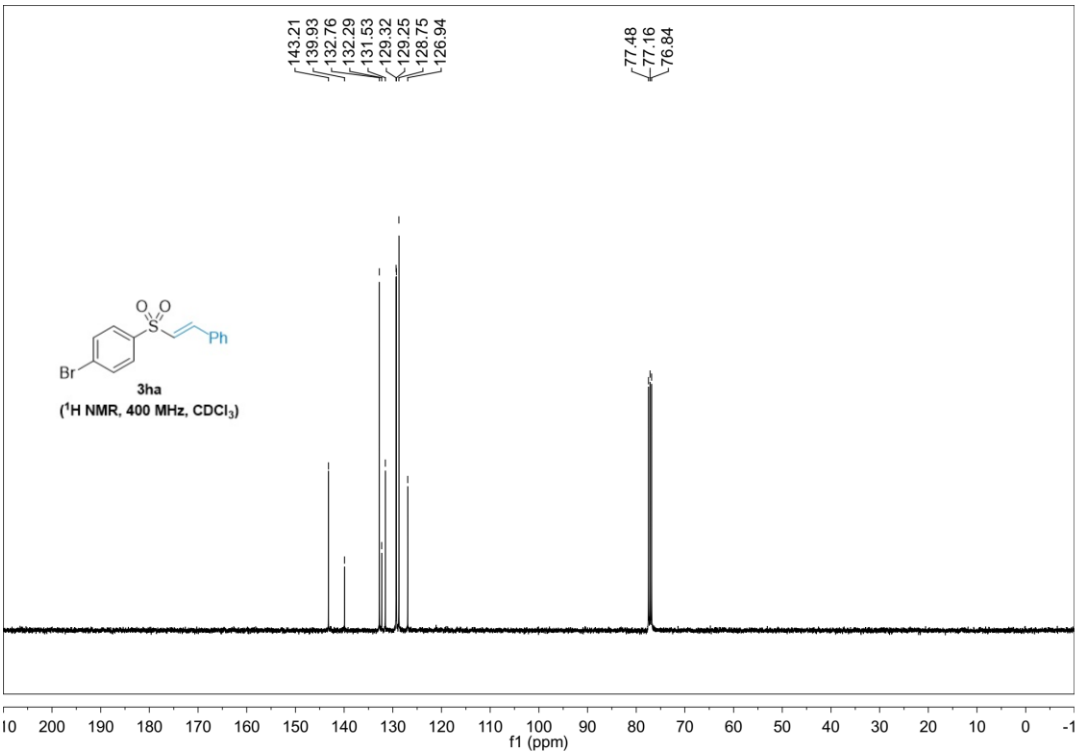
143.21
139.93
132.76
132.29
131.53
129.32
129.25
128.75
126.94
77.48
77.16
76.84
Br
Ph
3ha
(1H NMR, 400 MHz, CDCl3)
f1 (ppm)

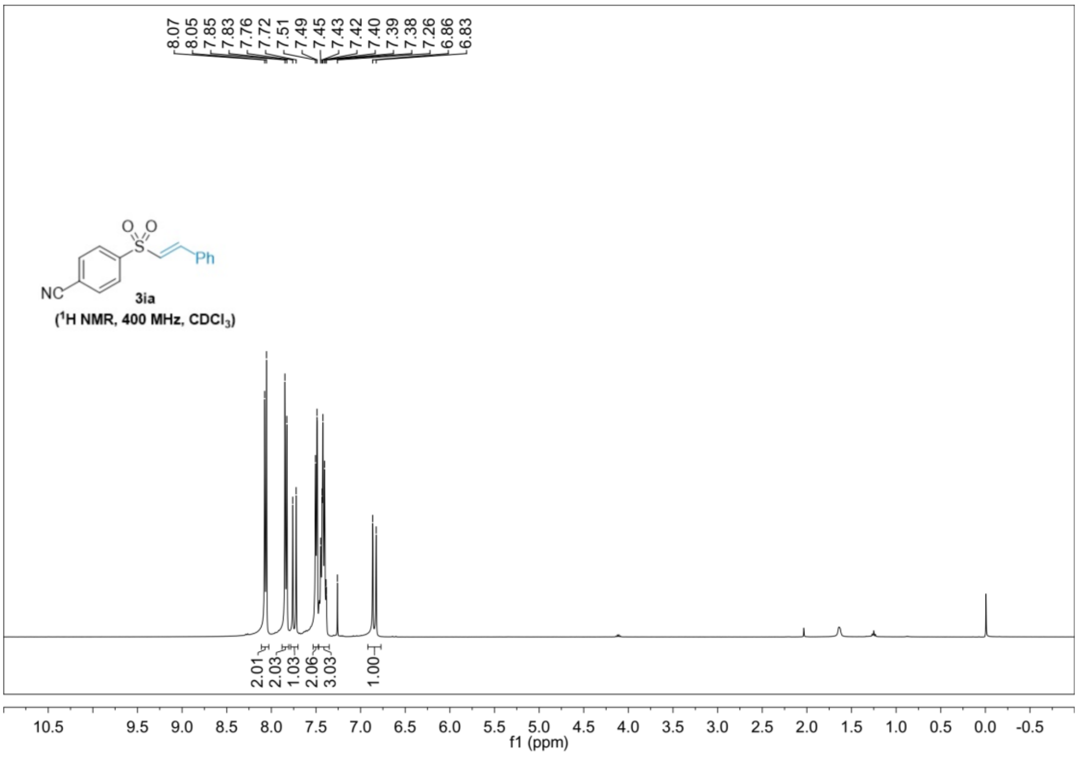
8.07
8.05
7.85
7.83
7.76
7.72
7.51
7.49
7.45
7.43
7.42
7.40
7.39
7.38
7.26
6.86
6.83
NC
Ph
3ia
(1H NMR, 400 MHz, CDCl3)
2.01
2.03
1.03
2.06
3.03
1.00
f1 (ppm)

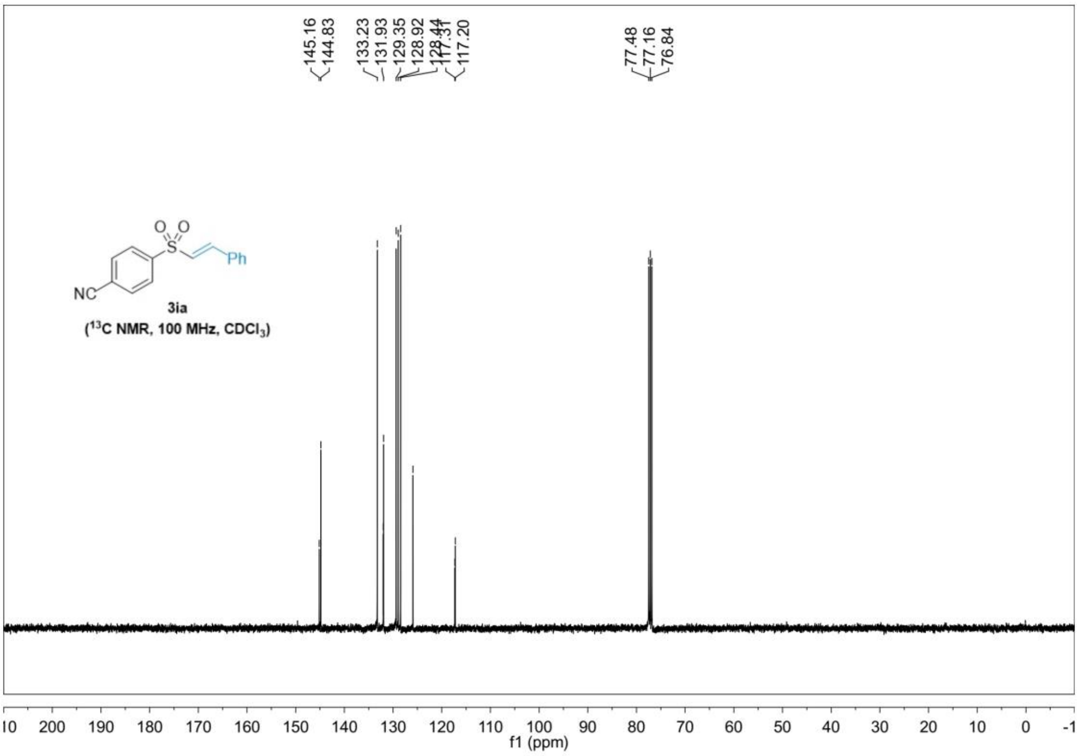
145.16
144.83
133.23
131.93
129.35
128.92
128.44
117.31
117.20
77.48
77.16
76.84
NC
O O
S
Ph
3ia
(13C NMR, 100 MHz, CDCl3)
f1 (ppm)

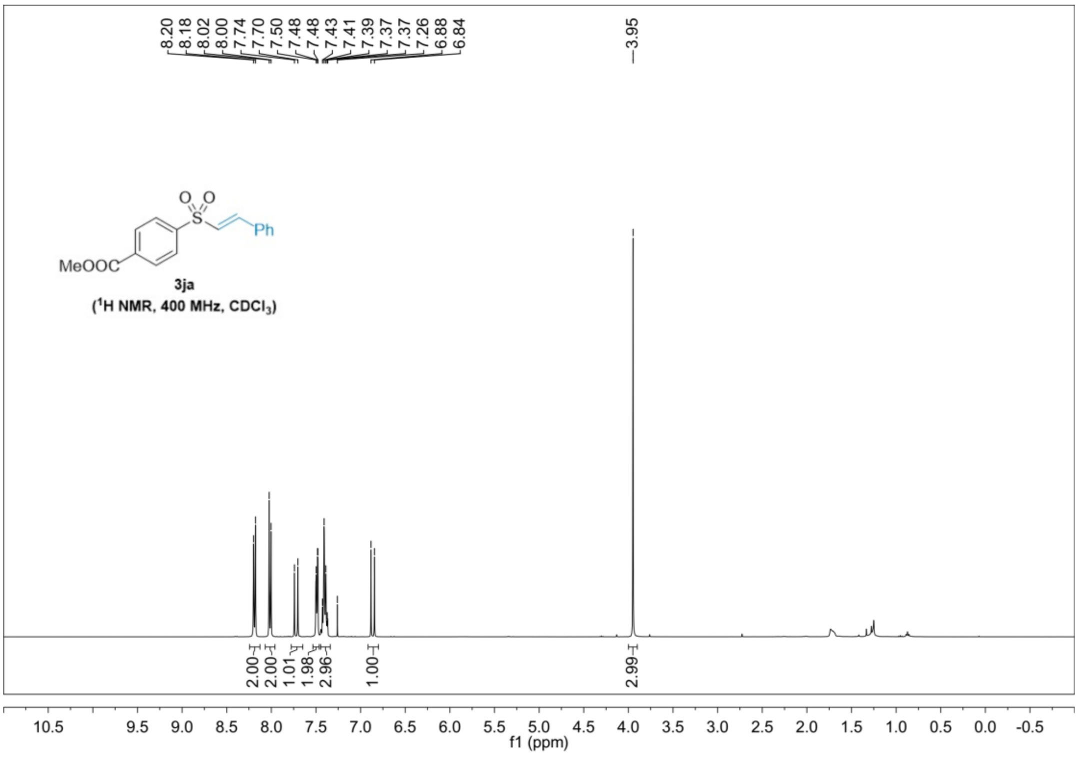
8.20
8.18
8.02
8.00
7.74
7.70
7.50
7.48
7.48
7.43
7.41
7.39
7.37
7.37
7.26
6.88
6.84
3.95
MeOOC
O O
S
Ph
3ja
(1H NMR, 400 MHz, CDCl3)
2.00
2.00
1.01
1.98
2.96
1.00
2.99
f1 (ppm)

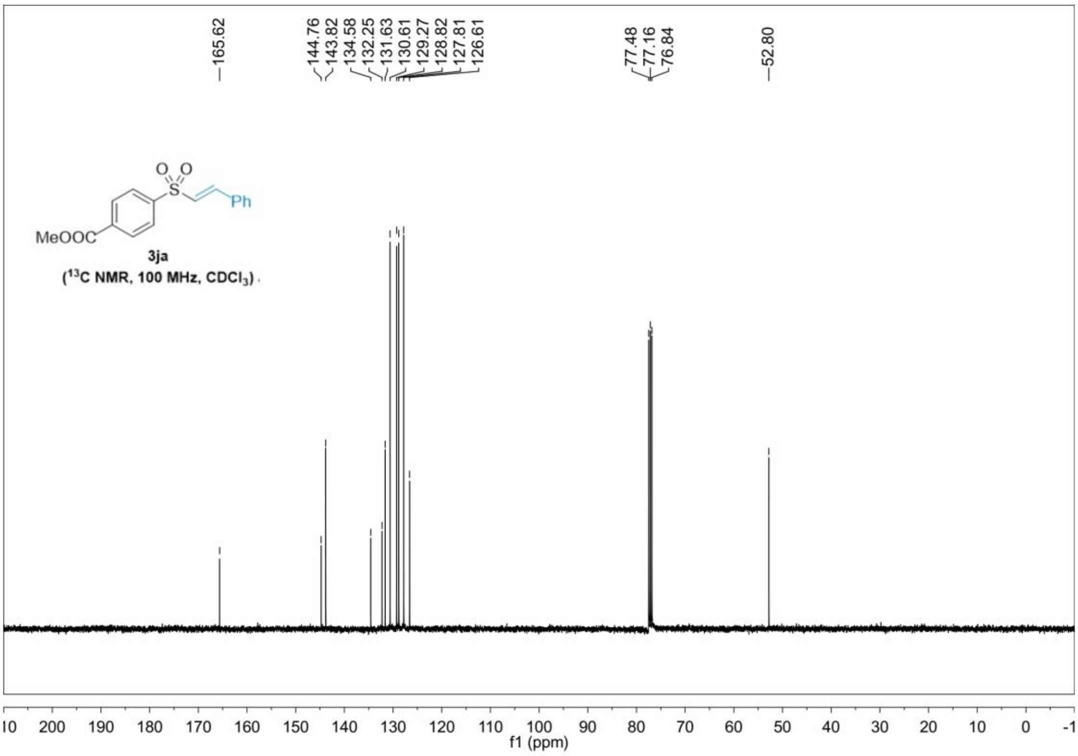
165.62
144.76
143.82
134.58
132.25
131.63
130.61
129.27
128.82
127.81
126.61
77.48
77.16
76.84
52.80
MeOOC
Ph
3ja
(13C NMR, 100 MHz, CDCl3)
f1 (ppm)

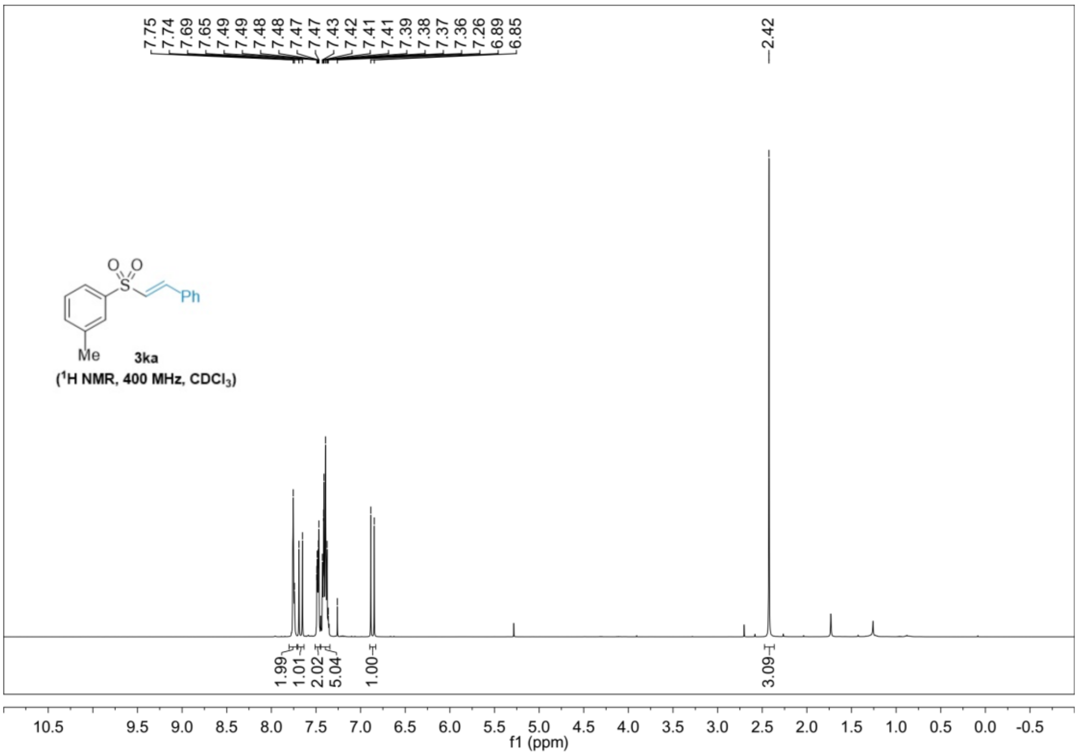
7.75
7.74
7.69
7.65
7.49
7.49
7.48
7.48
7.47
7.47
7.43
7.42
7.41
7.41
7.39
7.38
7.37
7.36
7.26
6.89
6.85
2.42
Ph
Me
3ka
(1H NMR, 400 MHz, CDCl3)
1.99
1.01
2.02
5.04
1.00
3.09
f1 (ppm)

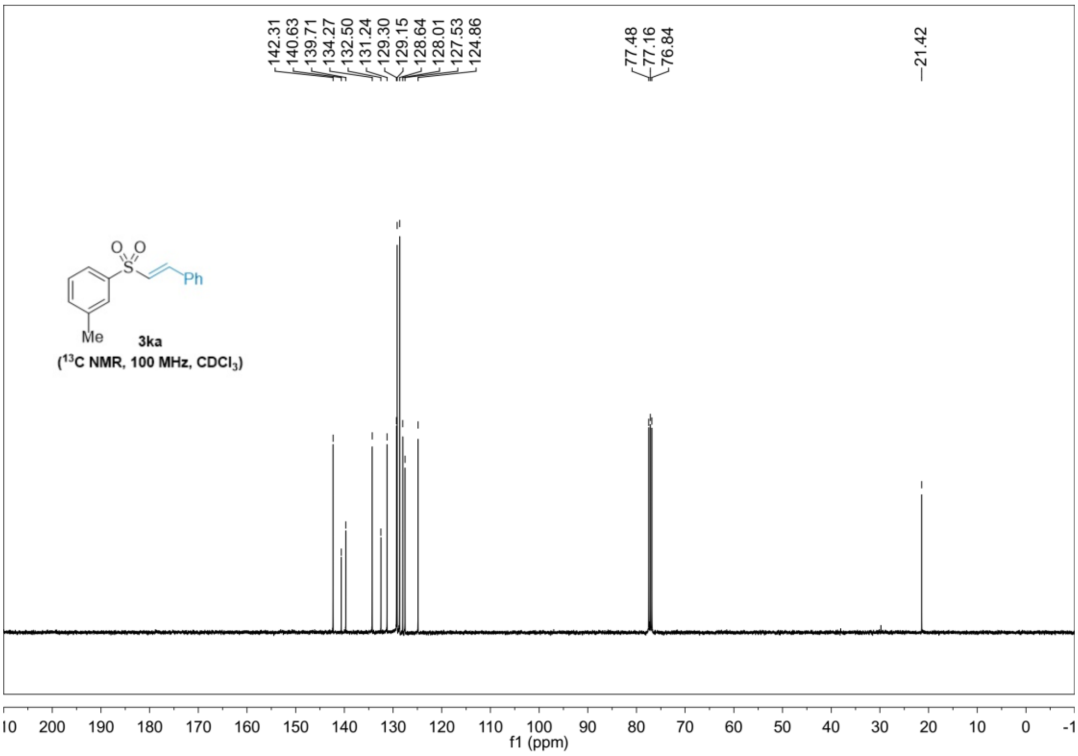
142.31
140.63
139.71
134.27
132.50
131.24
129.30
129.15
128.64
128.01
127.53
124.86
77.48
77.16
76.84
21.42
Me
Ph
3ka
(13C NMR, 100 MHz, CDCl3)
f1 (ppm)

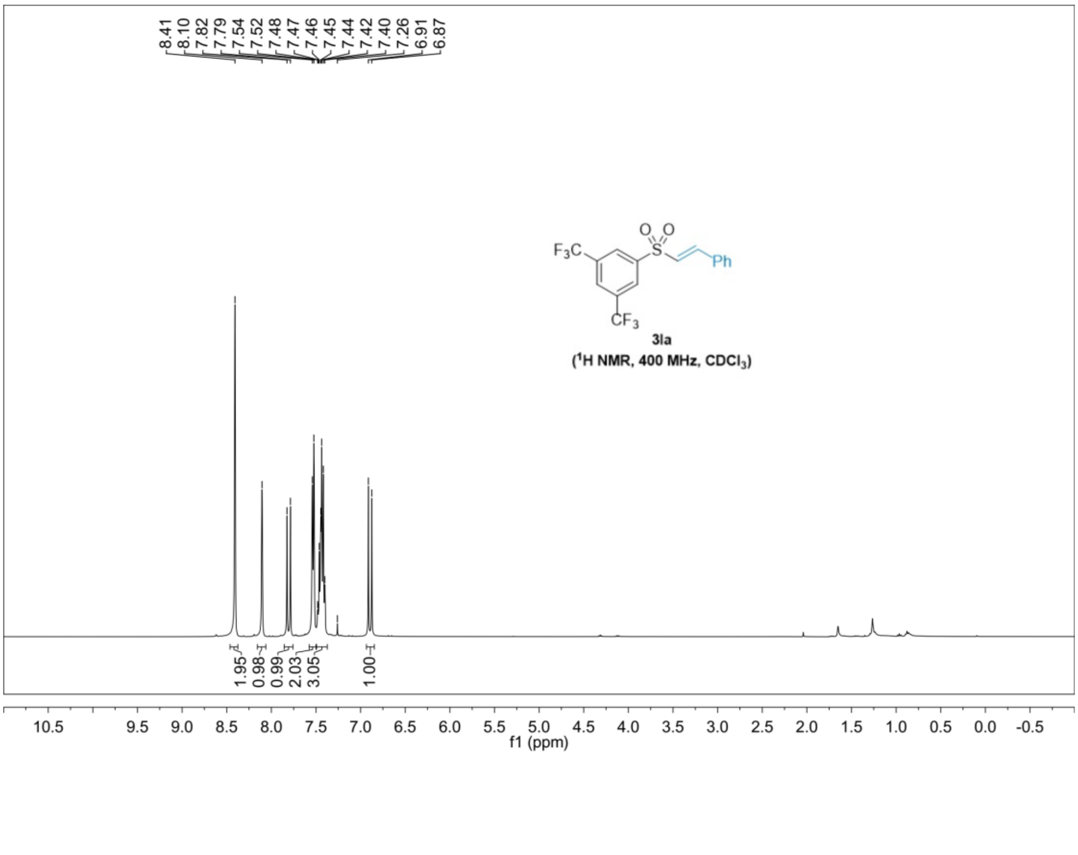
8.41
8.10
7.82
7.79
7.54
7.52
7.48
7.47
7.46
7.45
7.44
7.42
7.40
7.26
6.91
6.87
F3C
CF3
Ph
3la
(1H NMR, 400 MHz, CDCl3)
1.95
0.98
0.99
2.03
3.05
1.00
f1 (ppm)

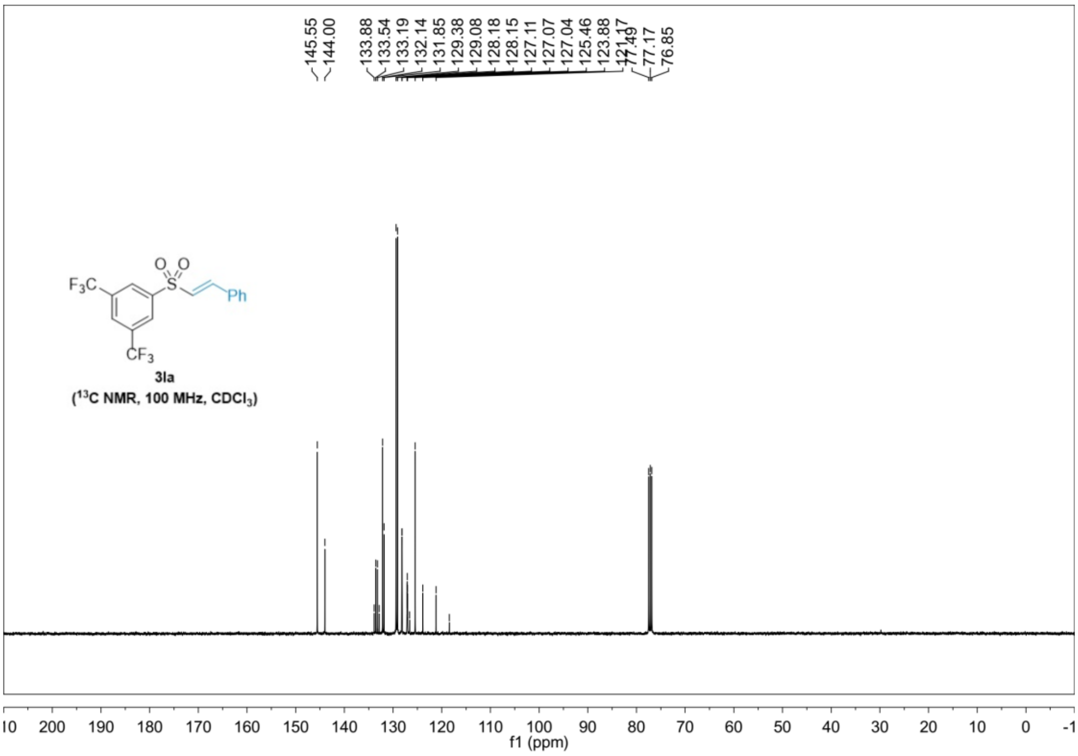
145.55
144.00
133.88
133.54
133.19
132.14
131.85
129.38
129.08
128.18
128.15
127.11
127.07
127.04
125.46
123.88
121.17
77.49
77.17
76.85
3la
(13C NMR, 100 MHz, CDCl3)
f1 (ppm)

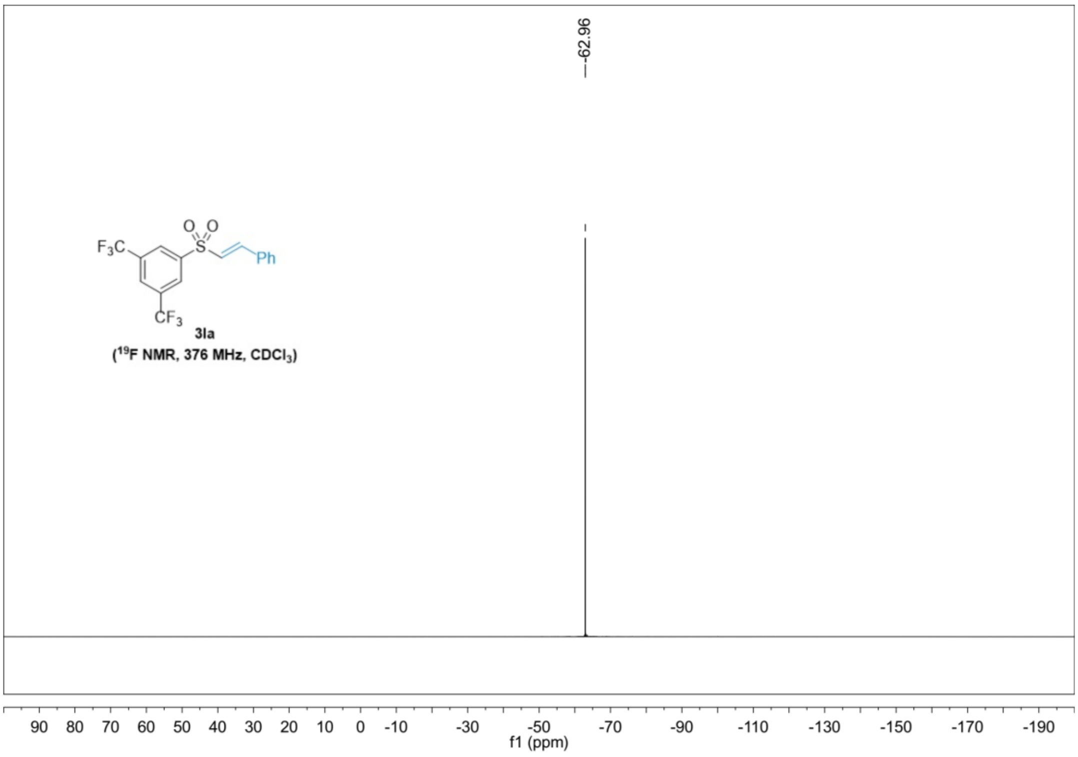
-62.96
3la
(19F NMR, 376 MHz, CDCl3)
f1 (ppm)

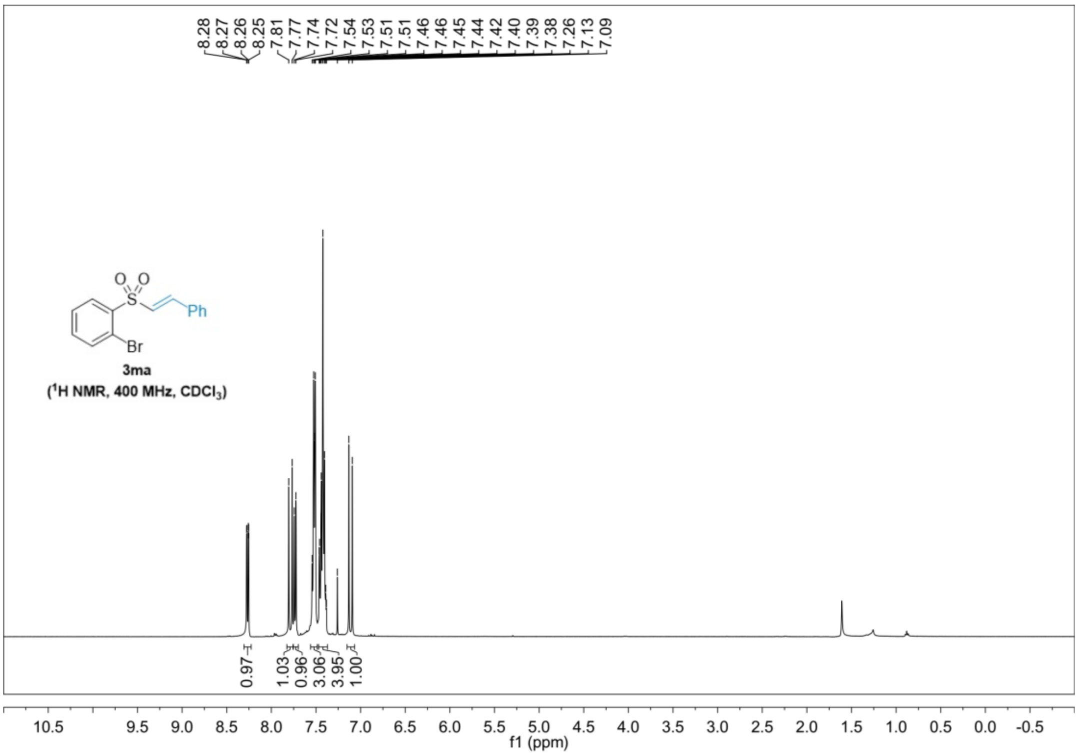
8.28
8.27
8.26
8.25
7.81
7.77
7.74
7.72
7.54
7.53
7.51
7.51
7.46
7.46
7.45
7.44
7.42
7.40
7.39
7.38
7.26
7.13
7.09
Ph
Br
3ma
(1H NMR, 400 MHz, CDCl3)
0.97
1.03
0.96
3.06
3.95
1.00
10.5
9.5
9.0
8.5
8.0
7.5
7.0
6.5
6.0
5.5
5.0
4.5
4.0
3.5
3.0
2.5
2.0
1.5
1.0
0.5
0.0
-0.5
f1 (ppm)

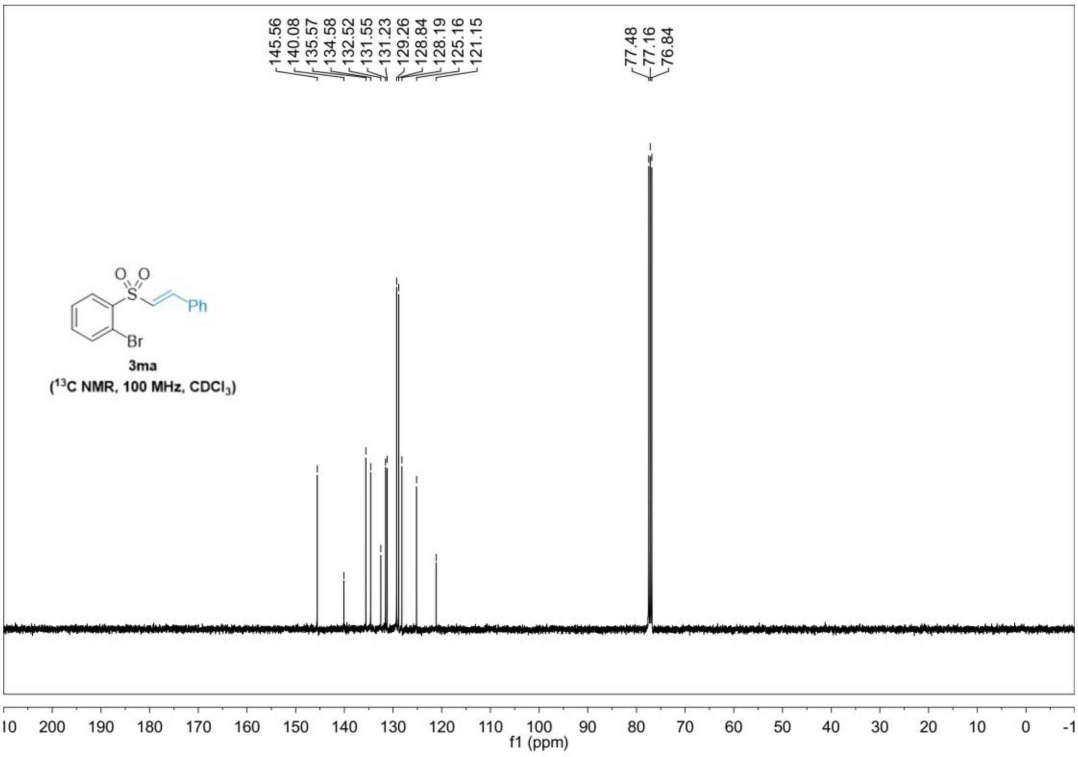
145.56
140.08
135.57
134.58
132.52
131.55
131.23
129.26
128.84
128.19
125.16
121.15
77.48
77.16
76.84
Ph
Br
3ma
(13C NMR, 100 MHz, CDCl3)
200
190
180
170
160
150
140
130
120
110
100
90
80
70
60
50
40
30
20
10
0
f1 (ppm)

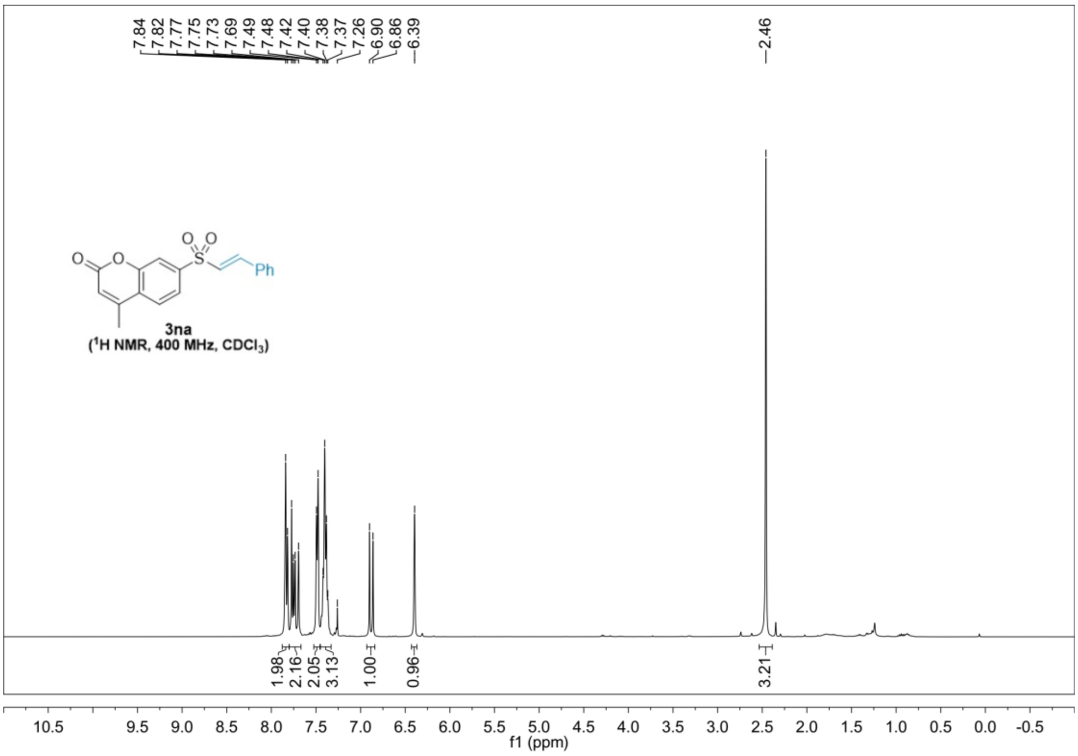

7.84
7.82
7.77
7.75
7.73
7.69
7.49
7.48
7.42
7.40
7.38
7.37
7.26
6.90
6.86
6.39
2.46
3na
(1H NMR, 400 MHz, CDCl3)
1.98
2.16
2.05
3.13
1.00
0.96
3.21
10.5 9.5 9.0 8.5 8.0 7.5 7.0 6.5 6.0 5.5 5.0 4.5 4.0 3.5 3.0 2.5 2.0 1.5 1.0 0.5 0.0 -0.5
f1 (ppm)

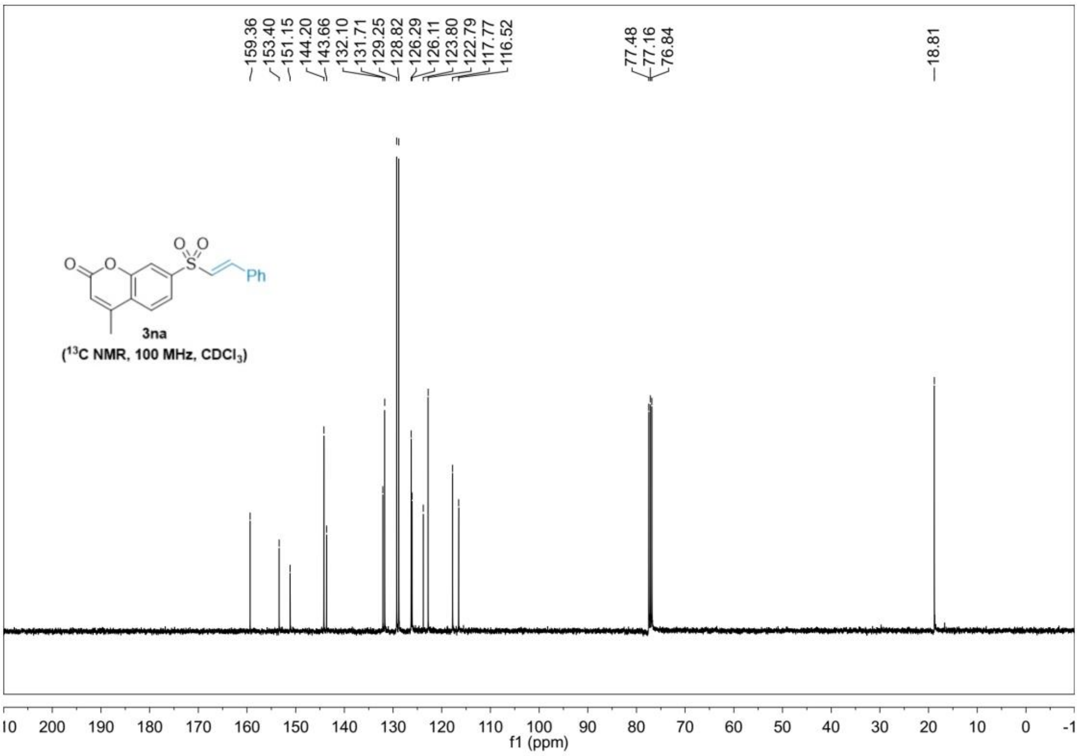

159.36
153.40
151.15
144.20
143.66
132.10
131.71
129.25
128.82
126.29
126.11
123.80
122.79
117.77
116.52
77.48
77.16
76.84
18.81
3na
(13C NMR, 100 MHz, CDCl3)
200 190 180 170 160 150 140 130 120 110 100 90 80 70 60 50 40 30 20 10 0
f1 (ppm)

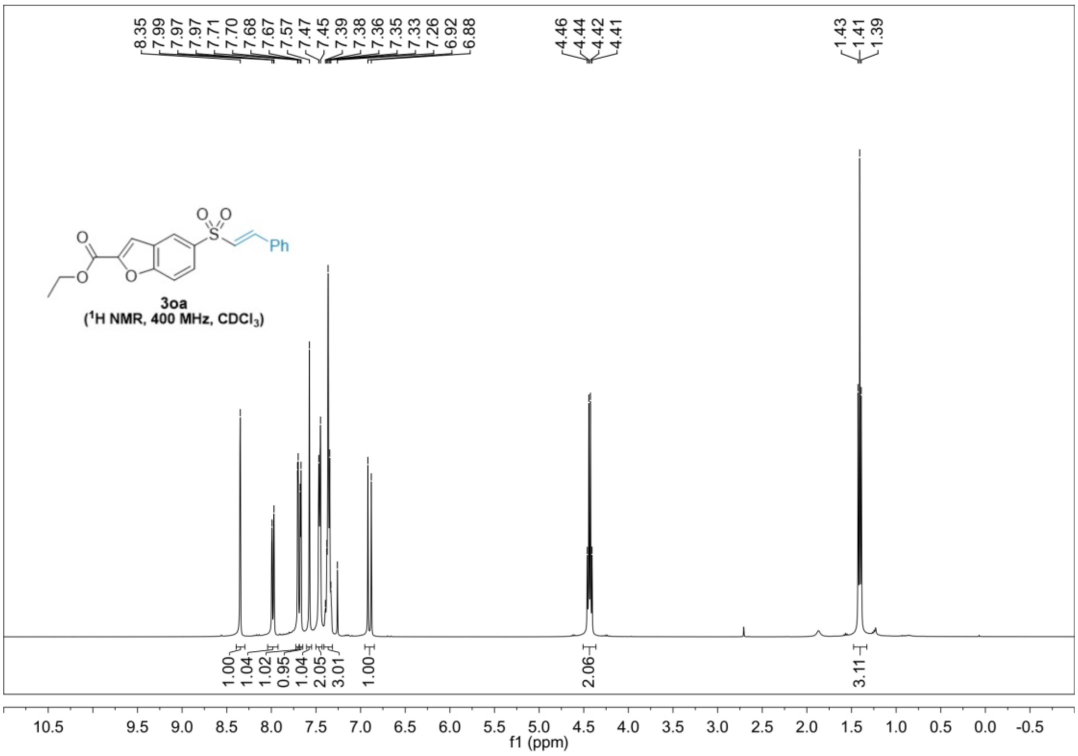

8.35 7.99 7.97 7.97 7.71 7.70 7.68 7.67 7.57 7.47 7.45 7.39 7.38 7.36 7.35 7.33 7.26 6.92 6.88
4.46 4.44 4.42 4.41
1.43 1.41 1.39
Ph
3oa
(1H NMR, 400 MHz, CDCl3)
1.00 1.04 1.02 0.95 1.04 2.05 3.01 1.00
2.06
3.11
f1 (ppm)

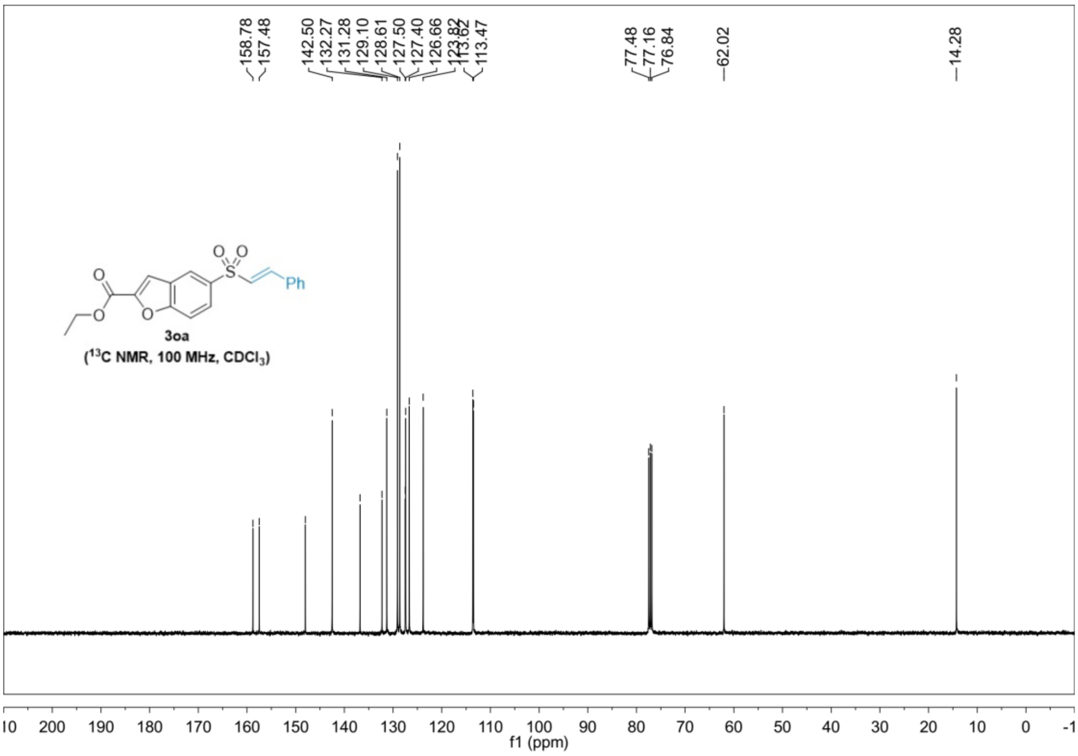

158.78 157.48
142.50 132.27 131.28 129.10 128.61 127.50 127.40 126.66 123.82 113.62 113.47
77.48 77.16 76.84
62.02
14.28
Ph
3oa
(13C NMR, 100 MHz, CDCl3)
f1 (ppm)

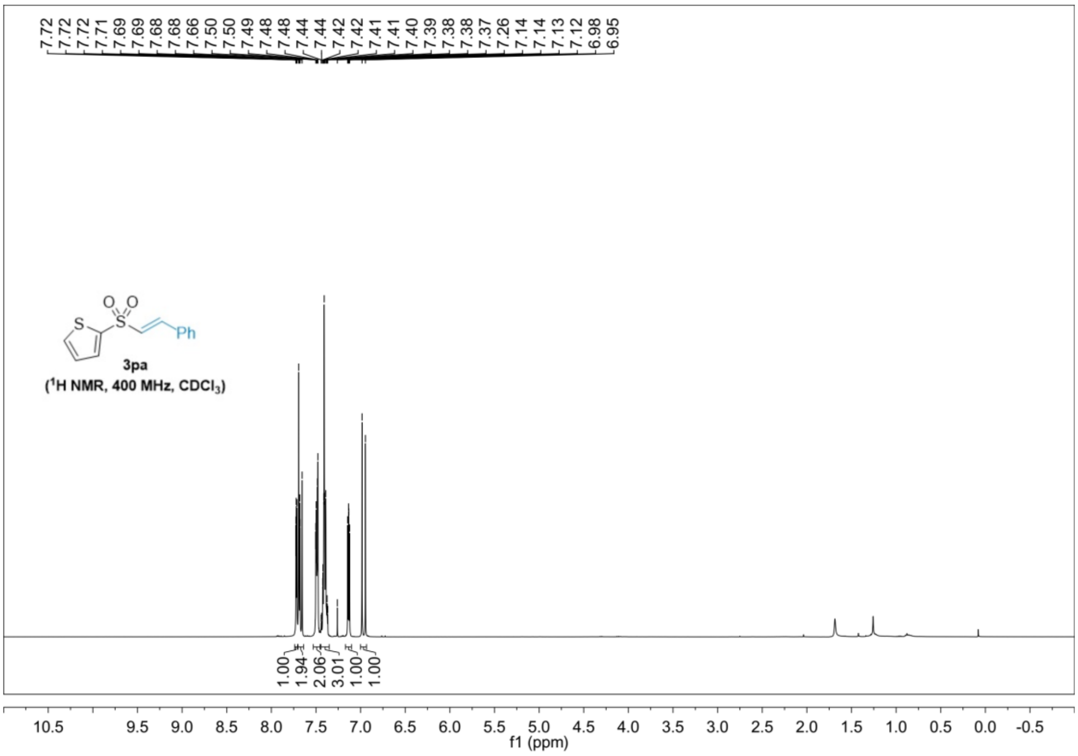
7.72
7.72
7.72
7.71
7.69
7.69
7.68
7.68
7.66
7.50
7.49
7.48
7.48
7.44
7.44
7.42
7.42
7.41
7.40
7.39
7.38
7.38
7.37
7.26
7.14
7.14
7.13
7.12
6.98
6.95
Ph
3pa
(1H NMR, 400 MHz, CDCl3)
1.00
1.94
2.06
3.01
1.00
1.00
10.5
9.5
9.0
8.5
8.0
7.5
7.0
6.5
6.0
5.5
5.0
4.5
4.0
3.5
3.0
2.5
2.0
1.5
1.0
0.5
0.0
-0.5
f1 (ppm)

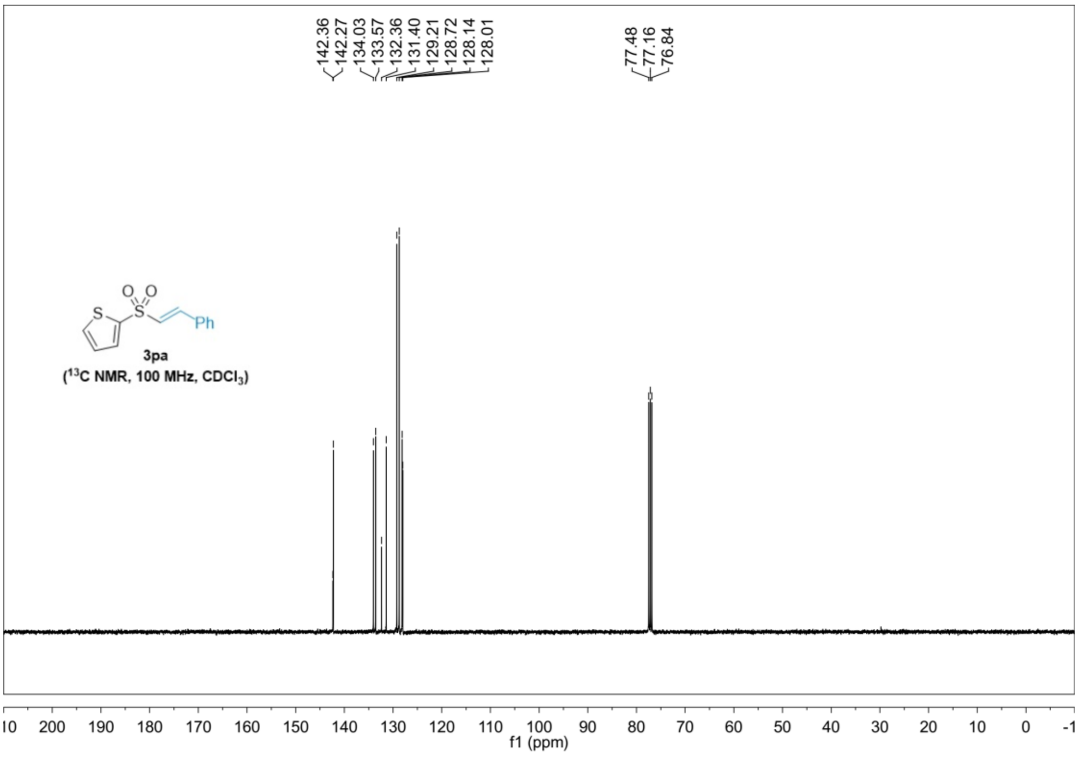
142.36
142.27
134.03
133.57
132.36
131.40
129.21
128.72
128.14
128.01
77.48
77.16
76.84
Ph
3pa
(13C NMR, 100 MHz, CDCl3)
200
190
180
170
160
150
140
130
120
110
100
90
80
70
60
50
40
30
20
10
0
f1 (ppm)

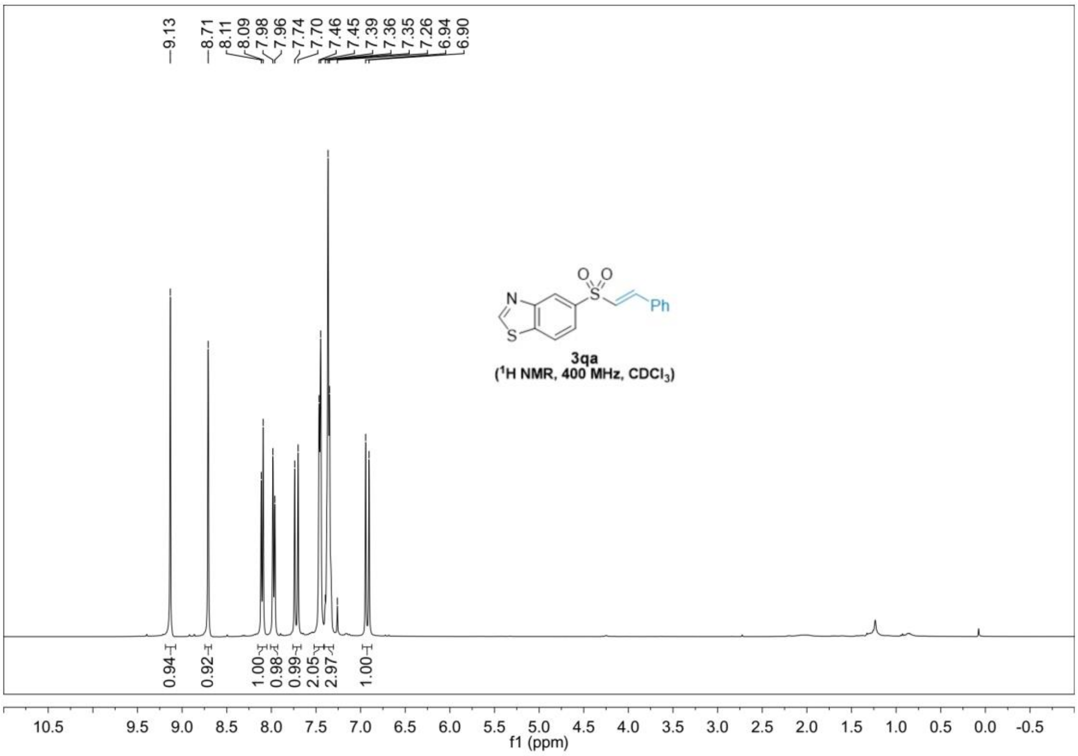
9.13
8.71
8.11
8.09
7.98
7.96
7.74
7.70
7.46
7.45
7.39
7.36
7.35
7.26
6.94
6.90
Ph
3qa
(^{1}H NMR, 400 MHz, CDCl$_3$)
0.94
0.92
1.00
0.98
0.99
2.05
2.97
1.00
f1 (ppm)

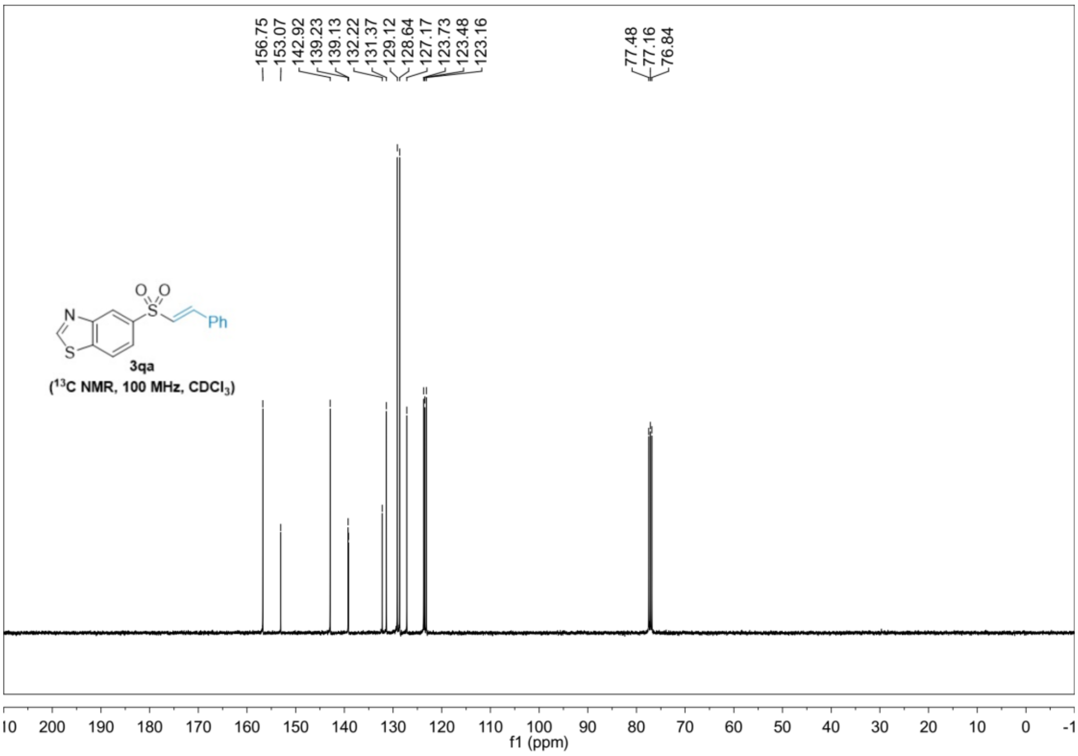
156.75
153.07
142.92
139.23
139.13
132.22
131.37
129.12
128.64
127.17
123.73
123.48
123.16
77.48
77.16
76.84
Ph
3qa
(^{13}C NMR, 100 MHz, CDCl$_3$)
f1 (ppm)

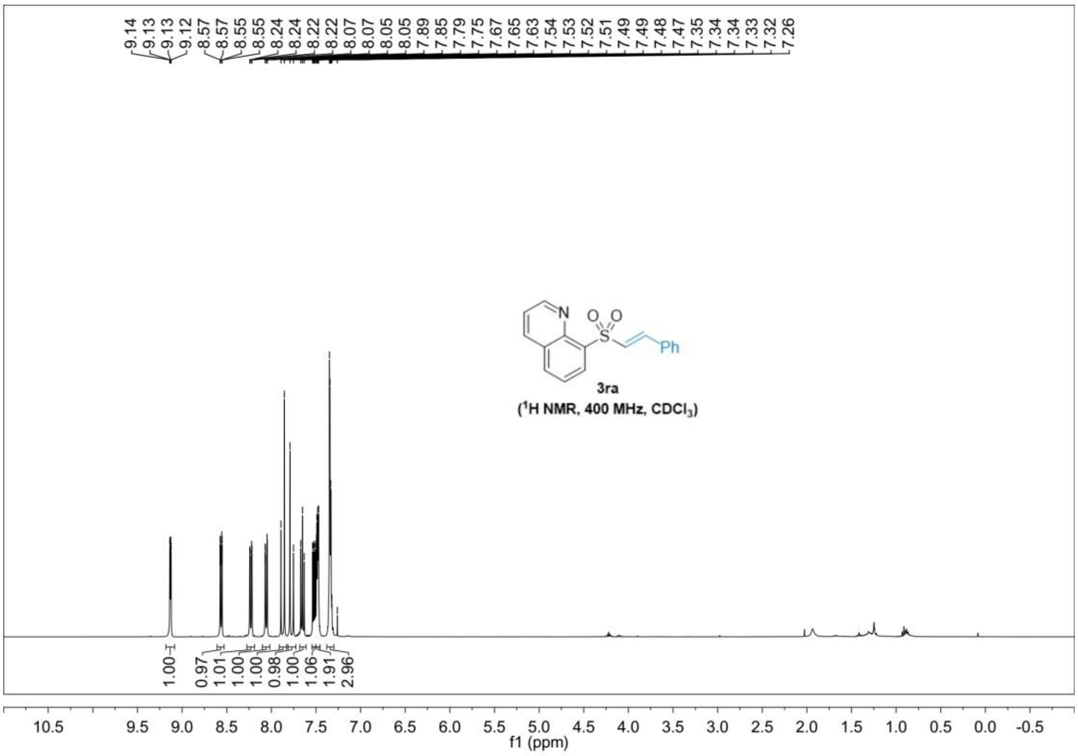
9.14
9.13
9.13
9.12
8.57
8.57
8.55
8.55
8.24
8.24
8.22
8.22
8.07
8.07
8.05
8.05
7.89
7.85
7.79
7.75
7.67
7.65
7.63
7.54
7.53
7.52
7.51
7.49
7.48
7.47
7.35
7.34
7.34
7.33
7.32
7.26
3ra
(^{1}H NMR, 400 MHz, CDCl$_3$)
1.00
0.97
1.01
1.00
1.00
0.98
1.00
1.06
1.91
2.96
f1 (ppm)

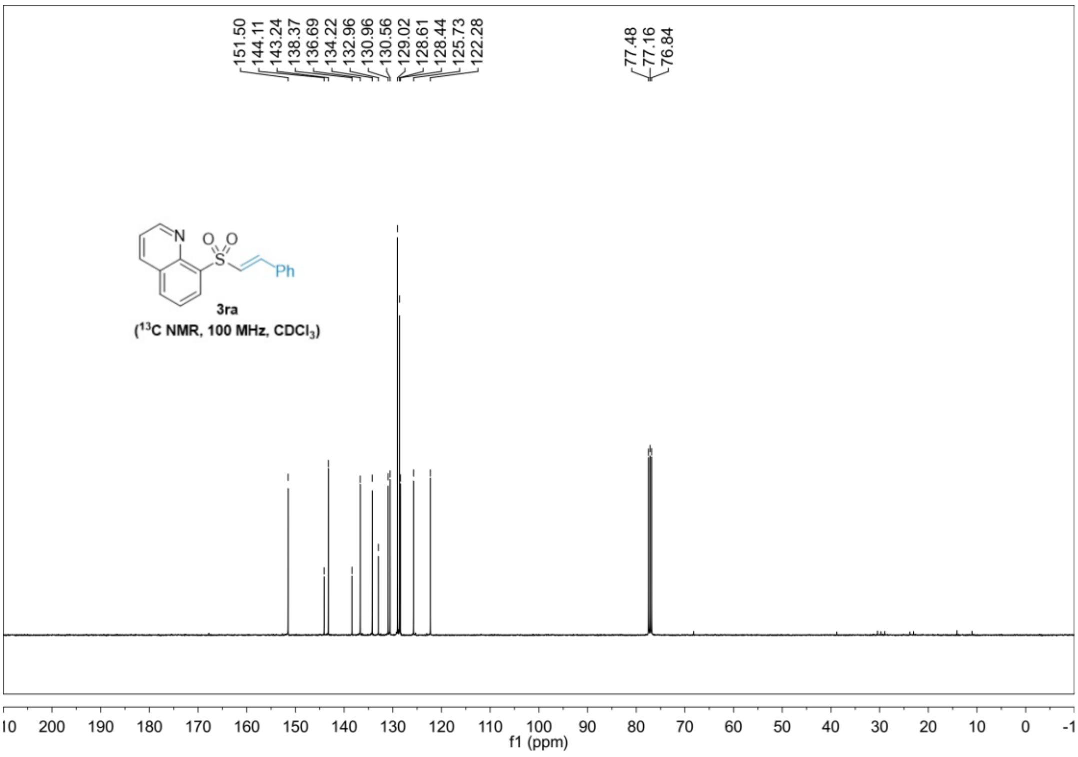
151.50
144.11
143.24
138.37
136.69
134.22
132.96
130.96
130.56
129.02
128.61
128.44
125.73
122.28
77.48
77.16
76.84
3ra
(^{13}C NMR, 100 MHz, CDCl$_3$)
f1 (ppm)

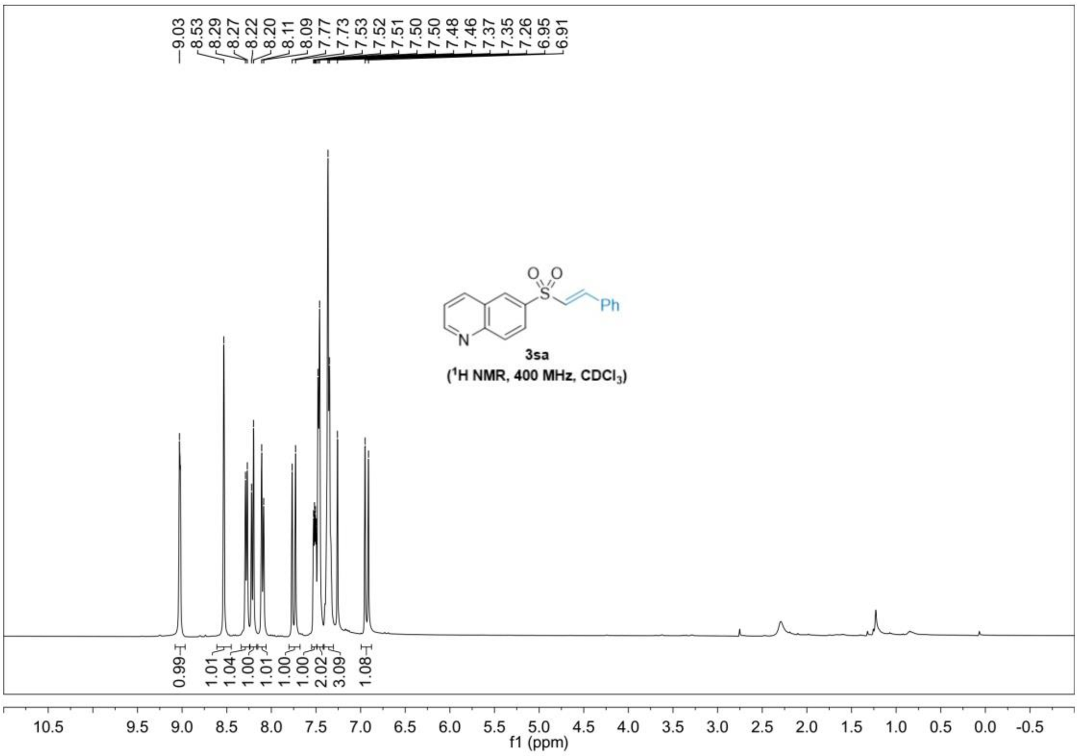
9.03
8.53
8.29
8.27
8.22
8.20
8.11
8.09
7.77
7.73
7.53
7.52
7.51
7.50
7.50
7.48
7.46
7.37
7.35
7.26
6.95
6.91
Ph
N
3sa
(1H NMR, 400 MHz, CDCl3)
0.99
1.01
1.04
1.00
1.01
1.00
1.00
2.02
3.09
1.08
f1 (ppm)

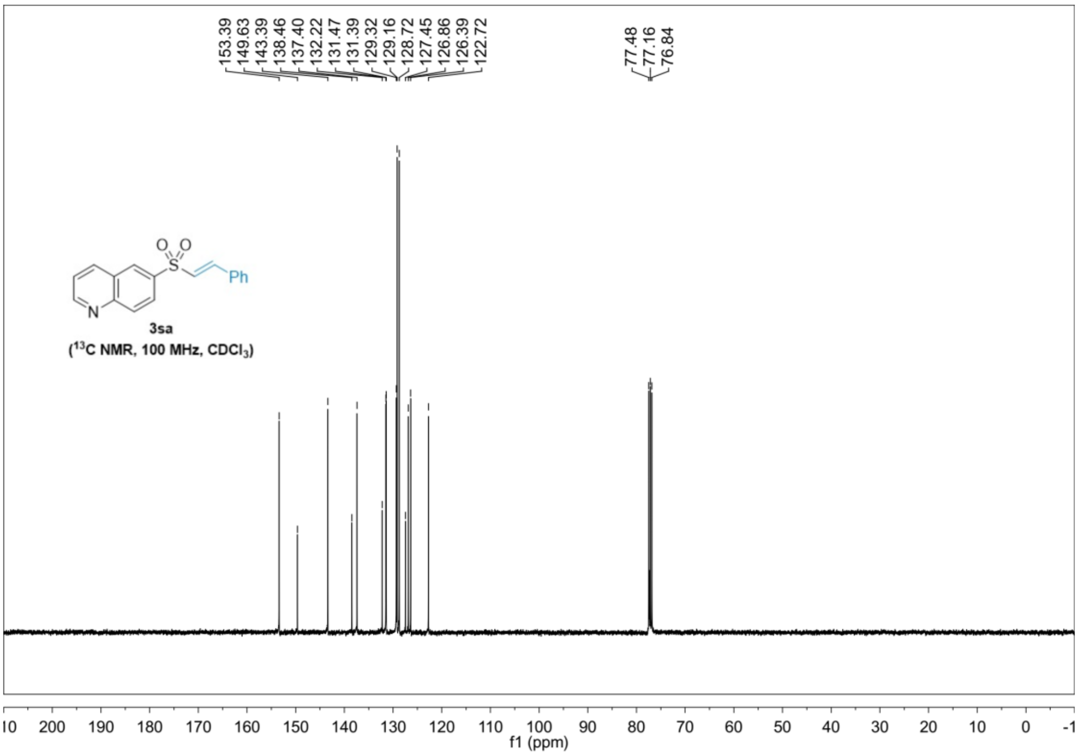
153.39
149.63
143.39
138.46
137.40
132.22
131.47
131.39
129.32
129.16
128.72
127.45
126.86
126.39
122.72
77.48
77.16
76.84
Ph
N
3sa
(13C NMR, 100 MHz, CDCl3)
f1 (ppm)

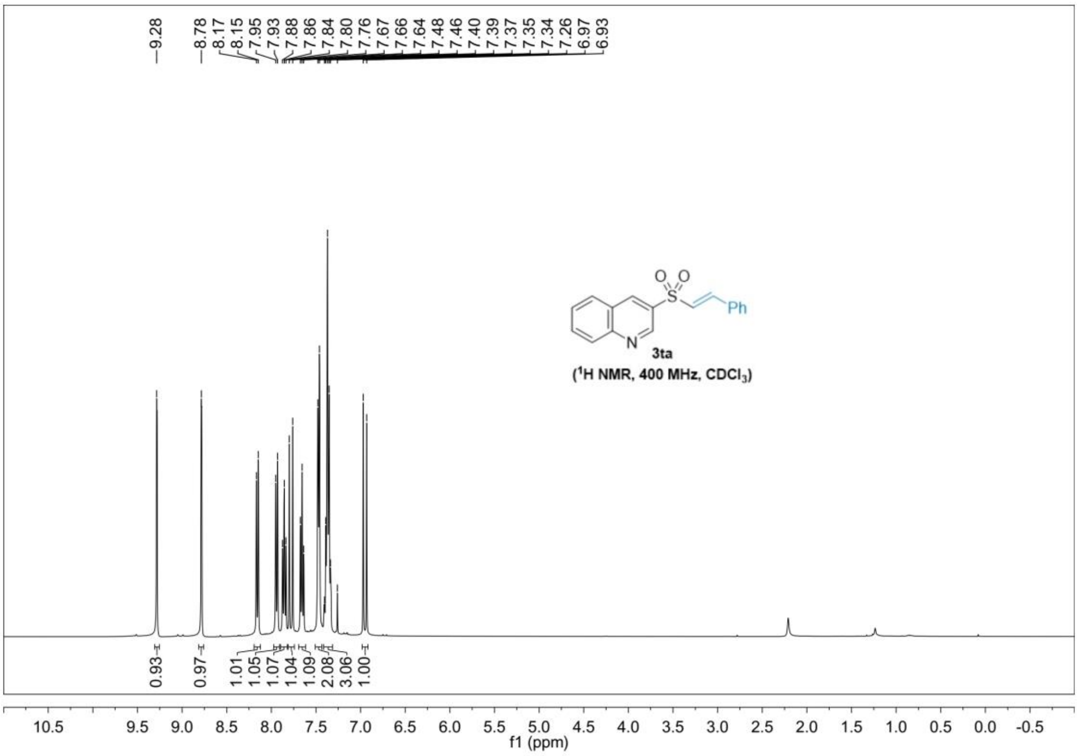
9.28
8.78
8.17
8.15
7.95
7.93
7.88
7.86
7.84
7.80
7.76
7.67
7.66
7.64
7.48
7.46
7.40
7.39
7.37
7.35
7.34
7.26
6.97
6.93
Ph
N
3ta
(^{1}H NMR, 400 MHz, CDCl$_3$)
0.93
0.97
1.01
1.05
1.07
1.04
1.09
2.08
3.06
1.00
10.5 9.5 9.0 8.5 8.0 7.5 7.0 6.5 6.0 5.5 5.0 4.5 4.0 3.5 3.0 2.5 2.0 1.5 1.0 0.5 0.0 -0.5
f1 (ppm)

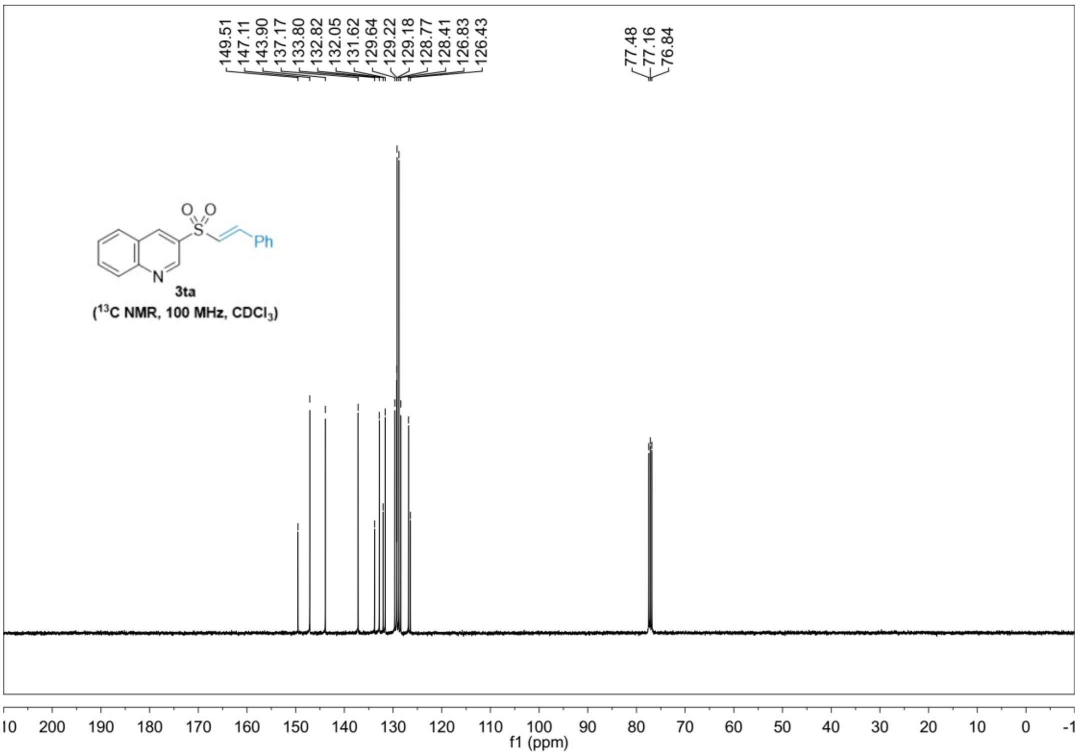
149.51
147.11
143.90
137.17
133.80
132.82
132.05
131.62
129.64
129.22
129.18
128.77
128.41
126.83
126.43
77.48
77.16
76.84
Ph
N
3ta
(^{13}C NMR, 100 MHz, CDCl$_3$)
200 190 180 170 160 150 140 130 120 110 100 90 80 70 60 50 40 30 20 10 0
f1 (ppm)

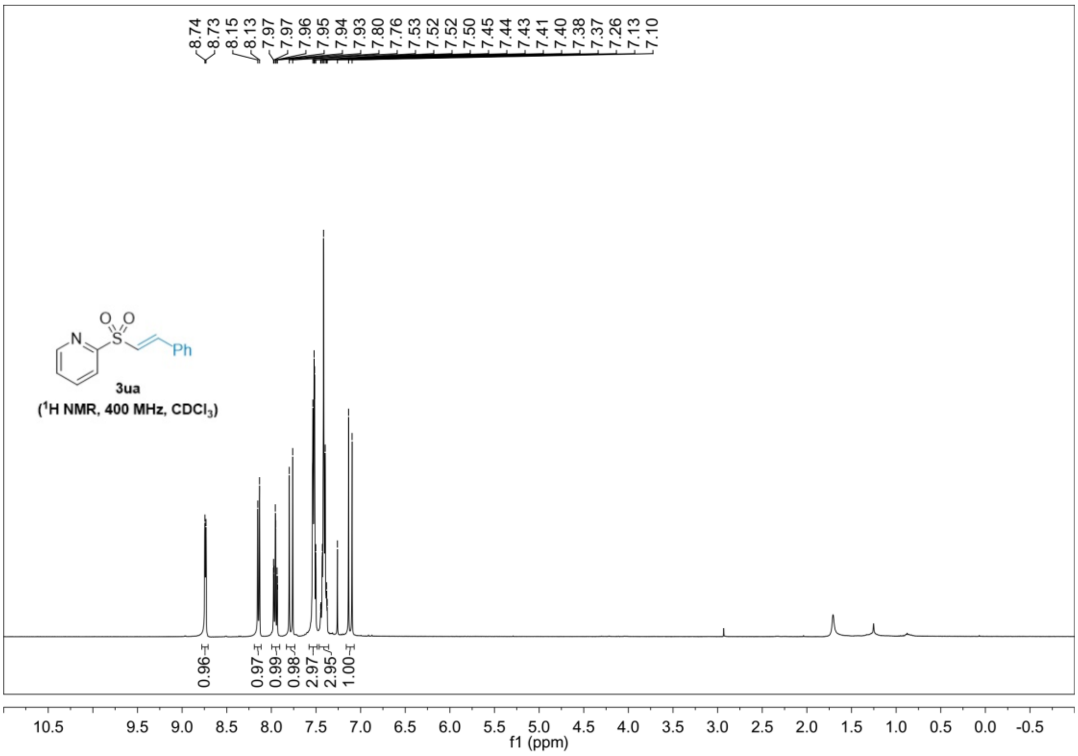
8.74
8.73
8.15
8.13
7.97
7.97
7.96
7.95
7.94
7.93
7.80
7.76
7.53
7.52
7.52
7.50
7.45
7.44
7.43
7.41
7.40
7.38
7.37
7.26
7.13
7.10
3ua
(1H NMR, 400 MHz, CDCl3)
0.96
0.97
0.99
0.98
2.97
2.95
1.00
f1 (ppm)

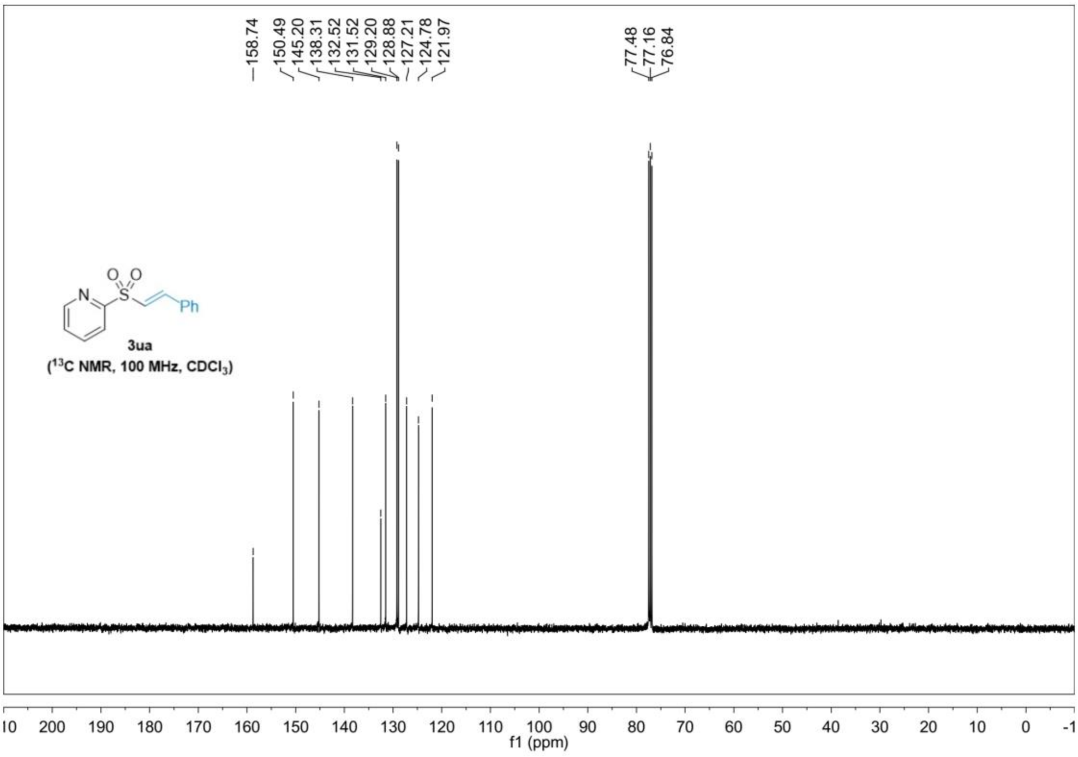
158.74
150.49
145.20
138.31
132.52
131.52
129.20
128.88
127.21
124.78
121.97
77.48
77.16
76.84
3ua
(13C NMR, 100 MHz, CDCl3)
f1 (ppm)

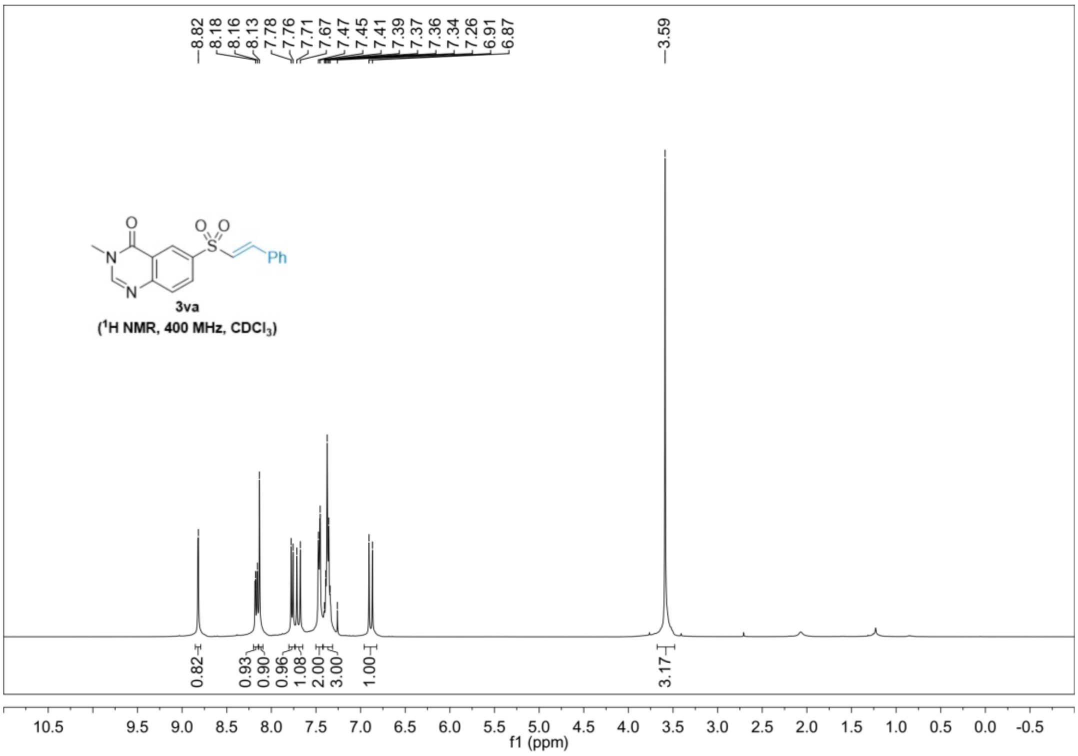
3va
(¹H NMR, 400 MHz, CDCl₃)
8.82 8.18 8.16 8.13 7.78 7.76 7.71 7.67 7.47 7.45 7.41 7.39 7.37 7.36 7.34 7.26 6.91 6.87
3.59
0.82 0.93 0.90 0.96 1.08 2.00 3.00 1.00 3.17
f1 (ppm)

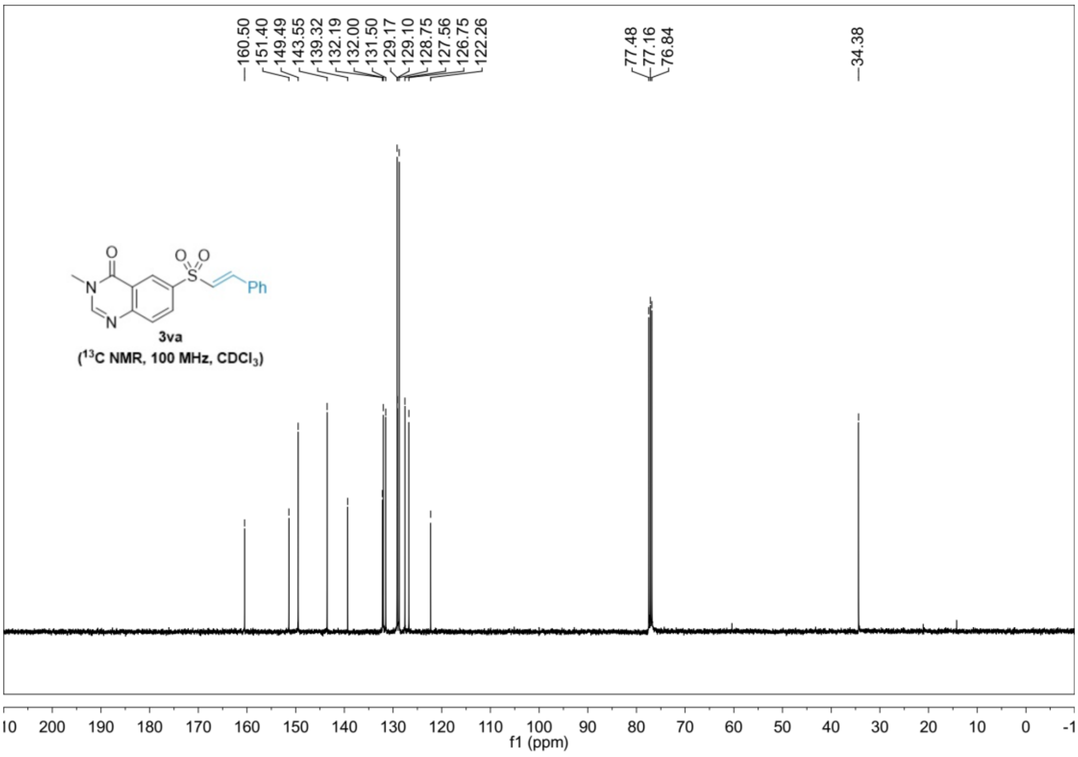
3va
(¹³C NMR, 100 MHz, CDCl₃)
160.50 151.40 149.49 143.55 139.32 132.19 132.00 131.50 129.17 129.10 128.75 127.56 126.75 122.26
77.48 77.16 76.84
34.38
f1 (ppm)

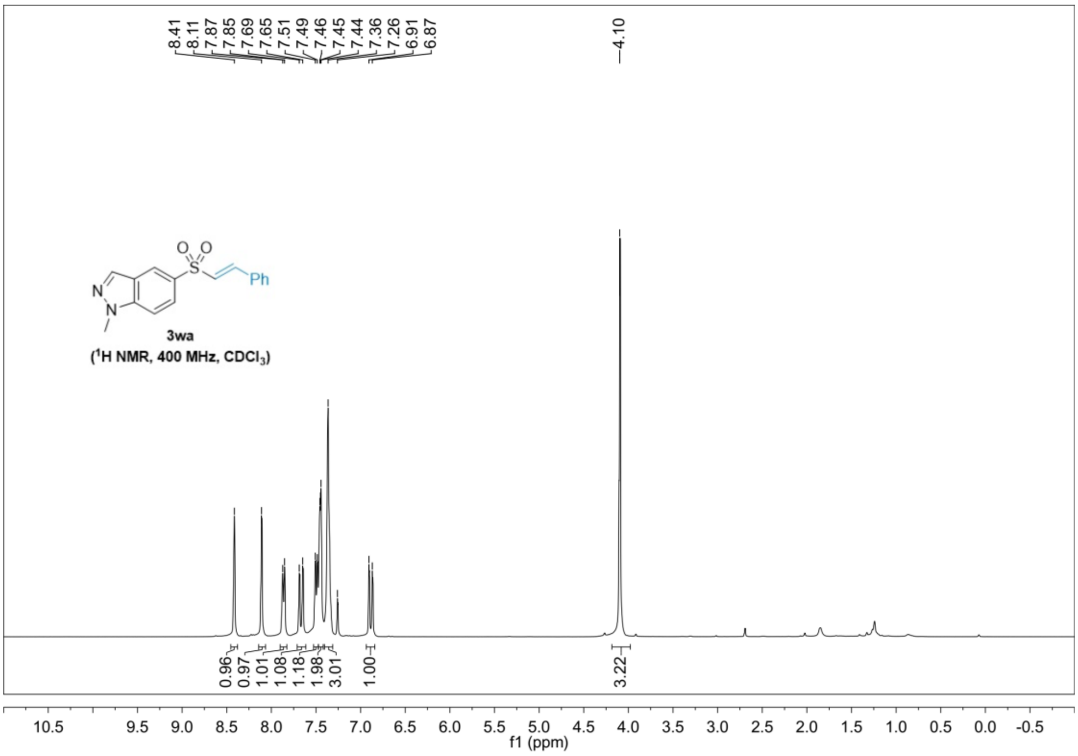
8.41
8.11
7.87
7.85
7.69
7.65
7.51
7.49
7.46
7.45
7.44
7.36
7.26
6.91
6.87
4.10
3wa
(^{1}H NMR, 400 MHz, CDCl$_3$)
0.96
0.97
1.01
1.08
1.18
1.98
3.01
1.00
3.22
f1 (ppm)

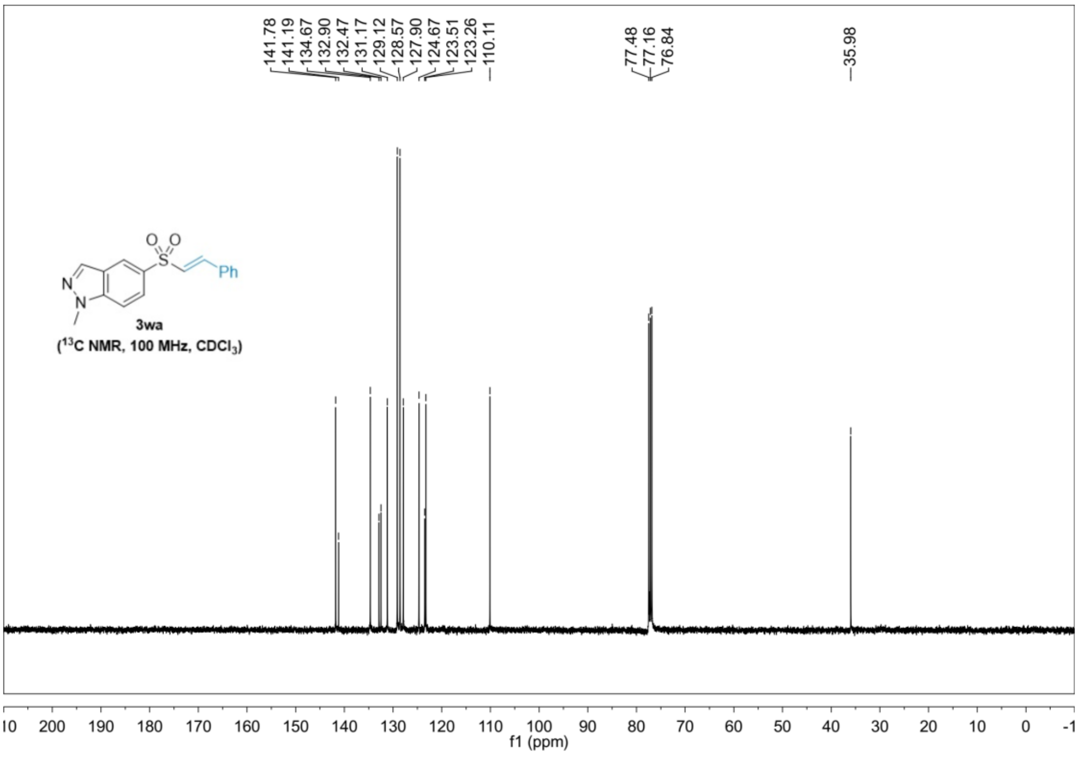
141.78
141.19
134.67
132.90
132.47
131.17
129.12
128.57
127.90
124.67
123.51
123.26
110.11
77.48
77.16
76.84
35.98
3wa
(^{13}C NMR, 100 MHz, CDCl$_3$)
f1 (ppm)

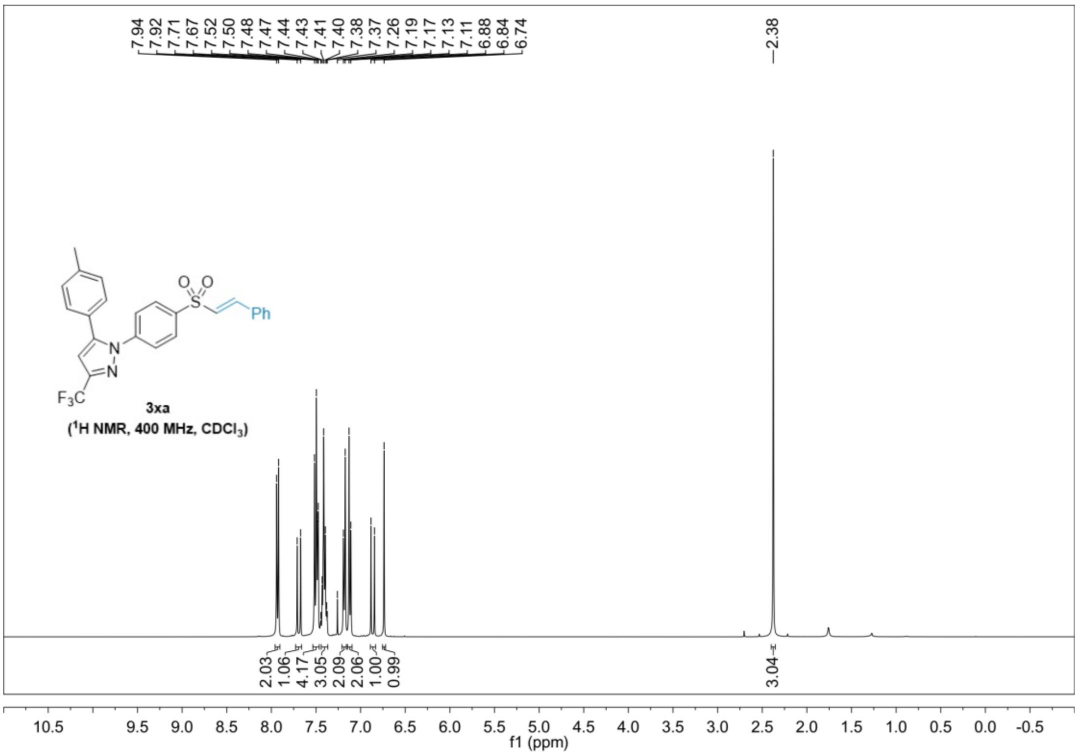
7.94
7.92
7.71
7.67
7.52
7.50
7.48
7.47
7.44
7.43
7.41
7.40
7.38
7.37
7.26
7.19
7.17
7.13
7.11
6.88
6.84
6.74
2.38
Ph
F3C
3xa
(1H NMR, 400 MHz, CDCl3)
2.03
1.06
4.17
3.05
2.09
2.06
1.00
0.99
3.04
f1 (ppm)

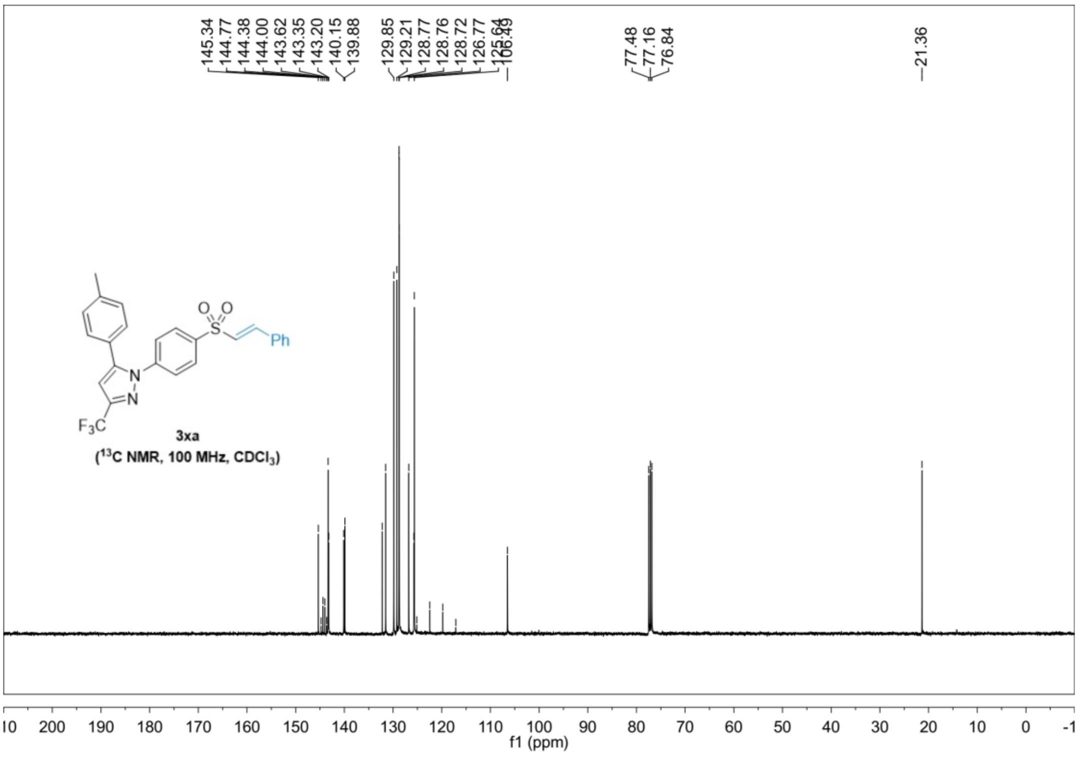
145.34
144.77
144.38
144.00
143.62
143.35
143.20
140.15
139.88
129.85
129.21
128.77
128.76
128.72
126.77
125.64
106.49
77.48
77.16
76.84
21.36
Ph
F3C
3xa
(13C NMR, 100 MHz, CDCl3)
f1 (ppm)

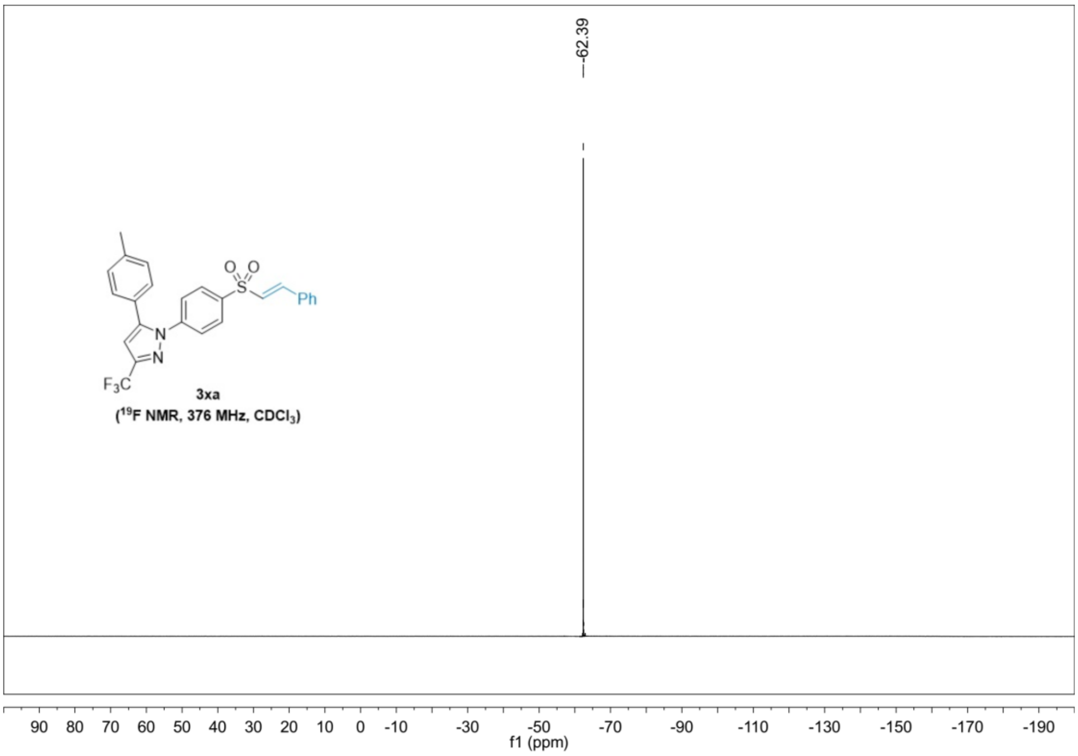
-62.39
3xa
(^{19}F NMR, 376 MHz, CDCl$_3$)
f1 (ppm)

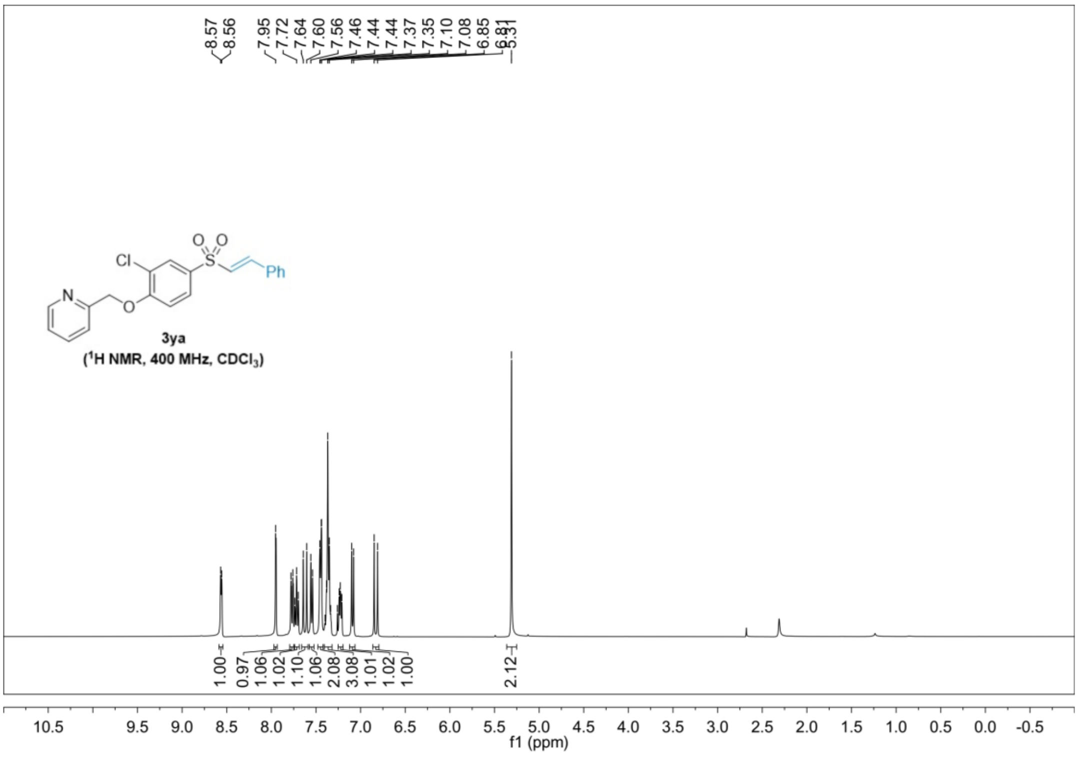
3ya
(^{1}H NMR, 400 MHz, CDCl$_3$)
f1 (ppm)

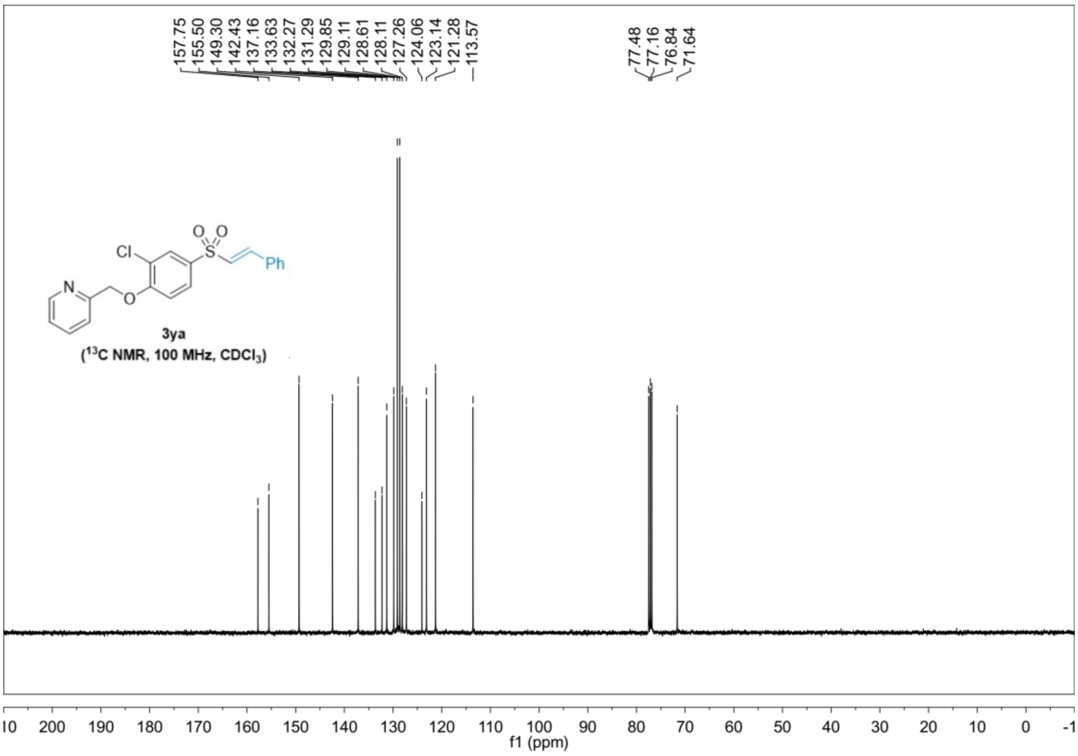
157.75
155.50
149.30
142.43
137.16
133.63
132.27
131.29
129.85
129.11
128.61
128.11
127.26
124.06
123.14
121.28
113.57
77.48
77.16
76.84
71.64
Cl
Ph
N
O
3ya
(^{13}C NMR, 100 MHz, CDCl$_3$)
f1 (ppm)

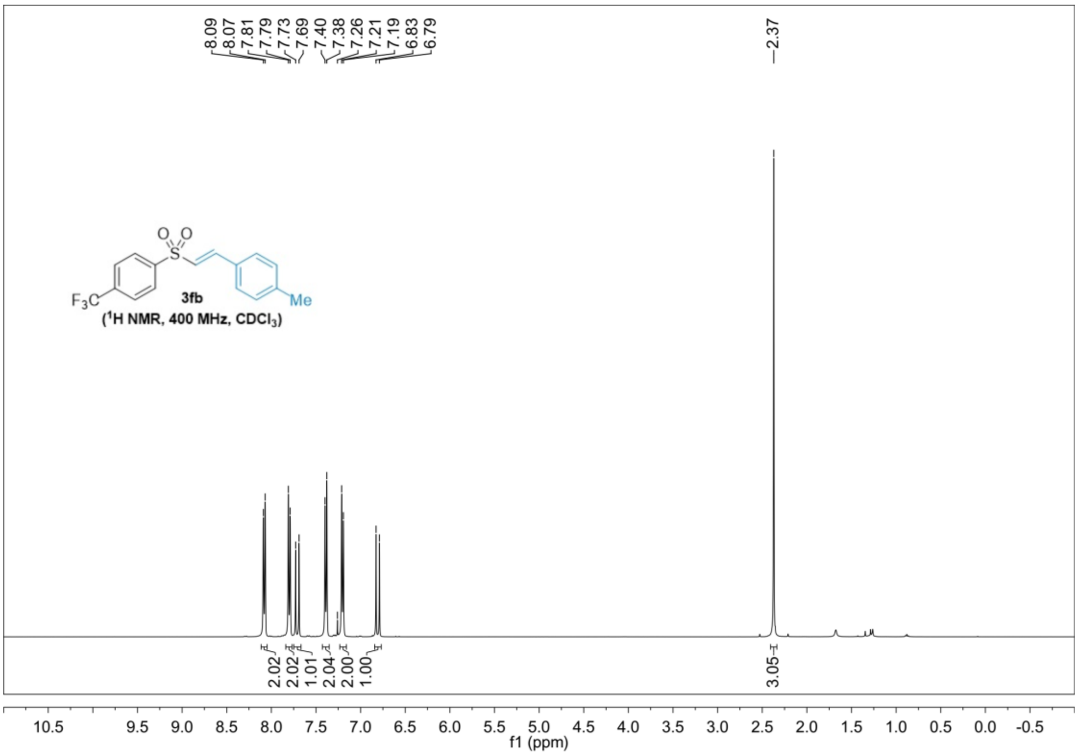
8.09
8.07
7.81
7.79
7.73
7.69
7.40
7.38
7.26
7.21
7.19
6.83
6.79
2.37
F_3C
Me
3fb
(^{1}H NMR, 400 MHz, CDCl$_3$)
2.02
2.02
1.01
2.04
2.00
1.00
3.05
f1 (ppm)

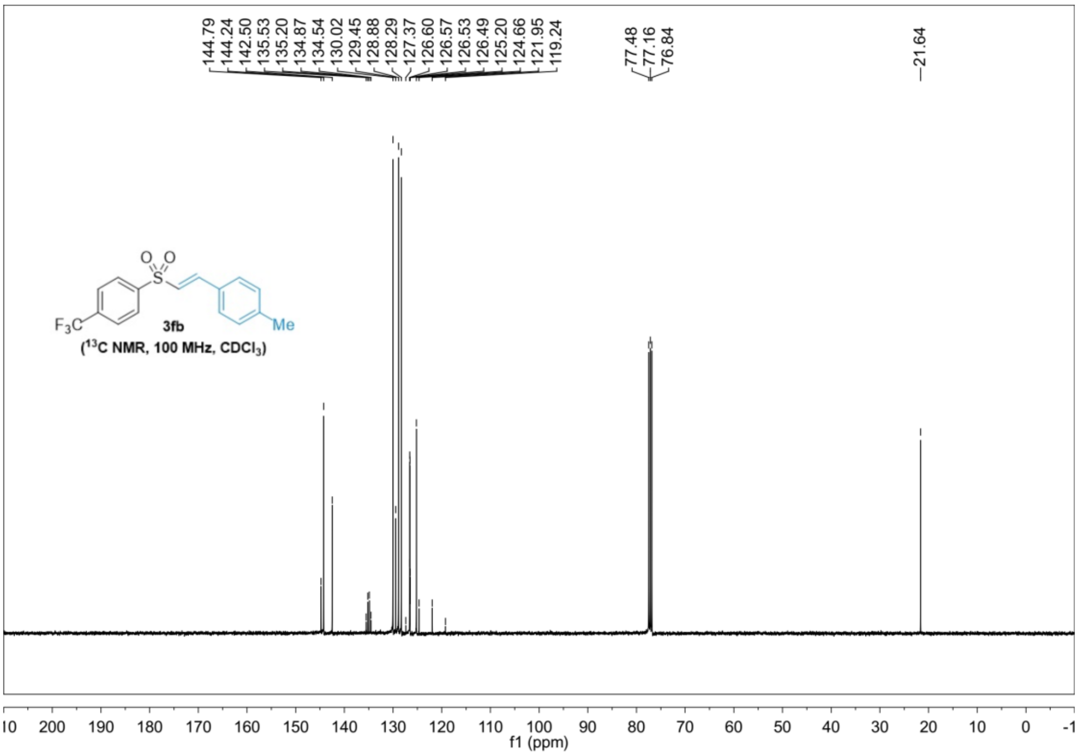
144.79
144.24
142.50
135.53
135.20
134.87
134.54
130.02
129.45
128.88
128.29
127.37
126.60
126.57
126.53
126.49
125.20
124.66
121.95
119.24
77.48
77.16
76.84
21.64
F3C
Me
3fb
(13C NMR, 100 MHz, CDCl3)
f1 (ppm)

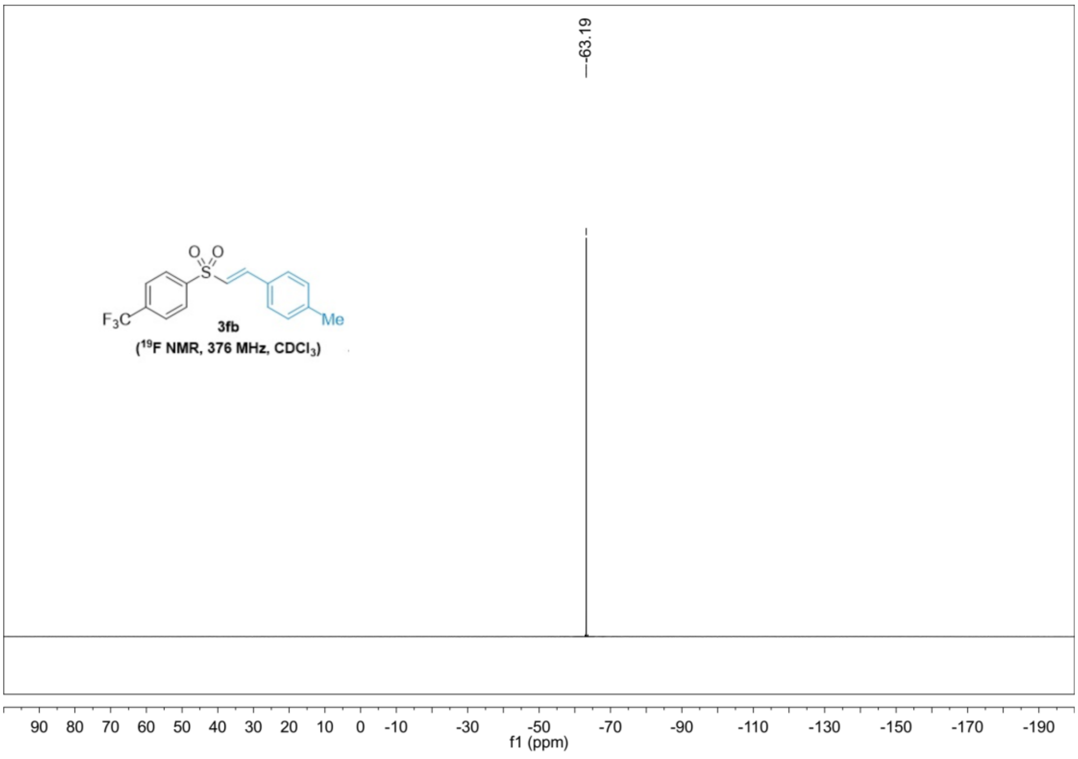
-63.19
F3C
Me
3fb
(19F NMR, 376 MHz, CDCl3)
f1 (ppm)

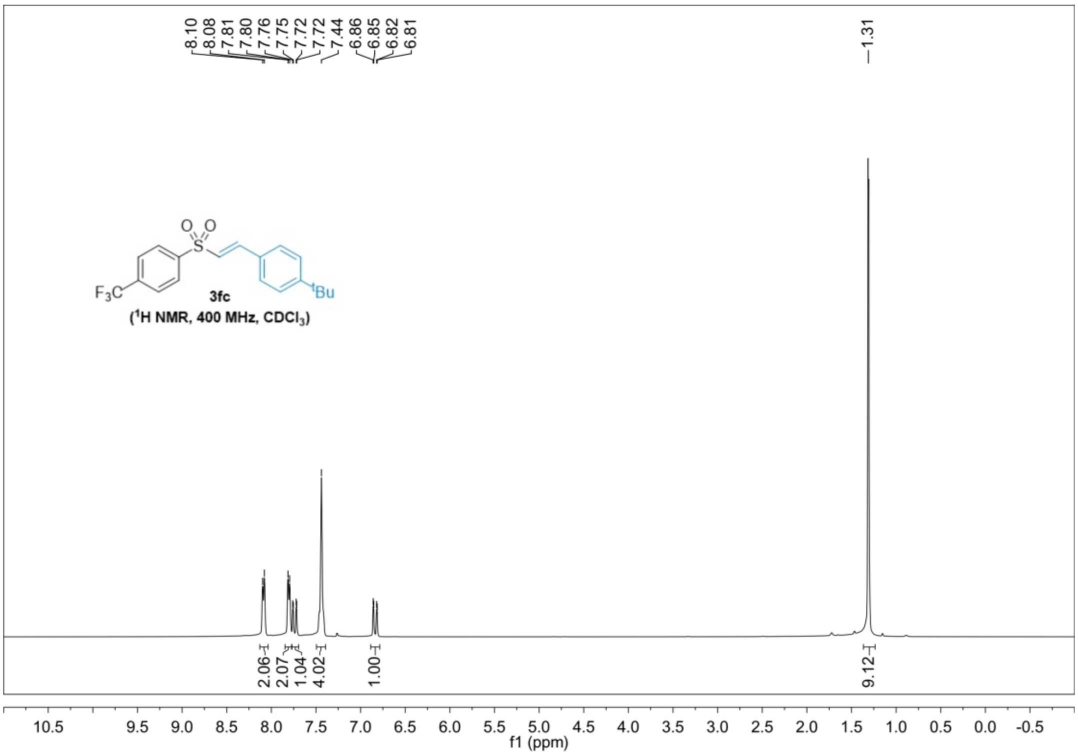
8.10
8.08
7.81
7.80
7.76
7.75
7.72
7.72
7.44
6.86
6.85
6.82
6.81
1.31
F3C
3fc
(1H NMR, 400 MHz, CDCl3)
2.06
2.07
1.04
4.02
1.00
9.12
10.5
9.5
9.0
8.5
8.0
7.5
7.0
6.5
6.0
5.5
5.0
4.5
4.0
3.5
3.0
2.5
2.0
1.5
1.0
0.5
0.0
-0.5
f1 (ppm)

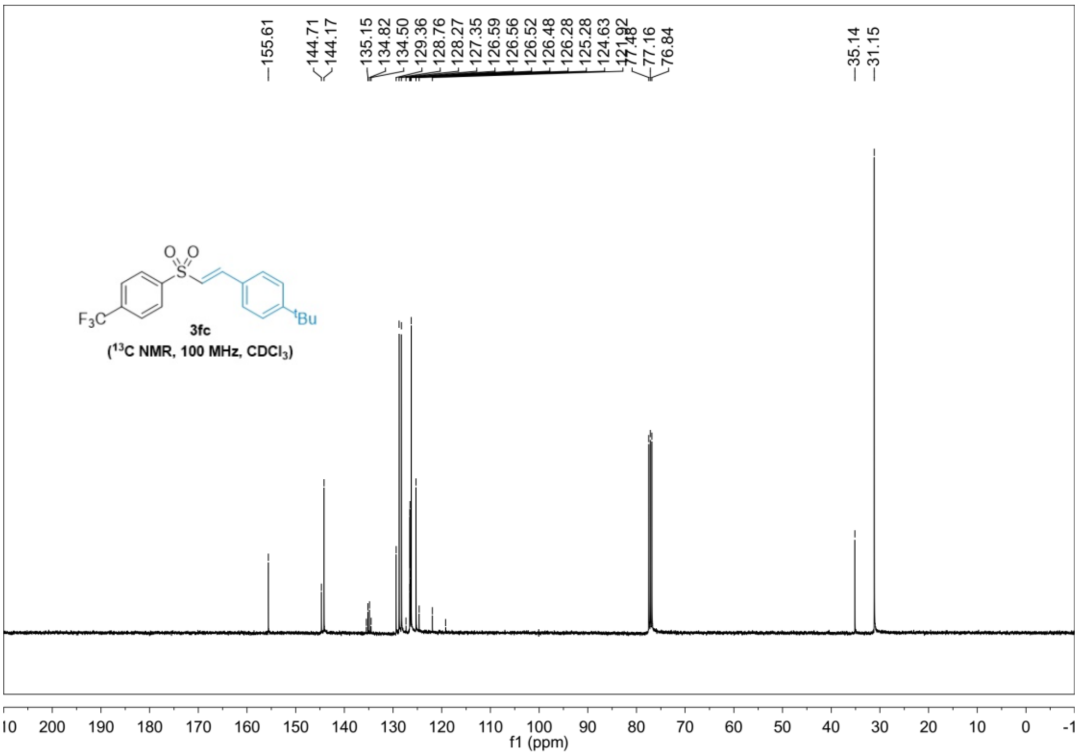
155.61
144.71
144.17
135.15
134.82
134.50
129.36
128.76
128.27
127.35
126.59
126.56
126.52
126.48
126.28
125.28
124.63
121.92
77.48
77.16
76.84
35.14
31.15
F3C
3fc
(13C NMR, 100 MHz, CDCl3)
200
190
180
170
160
150
140
130
120
110
100
90
80
70
60
50
40
30
20
10
0
f1 (ppm)

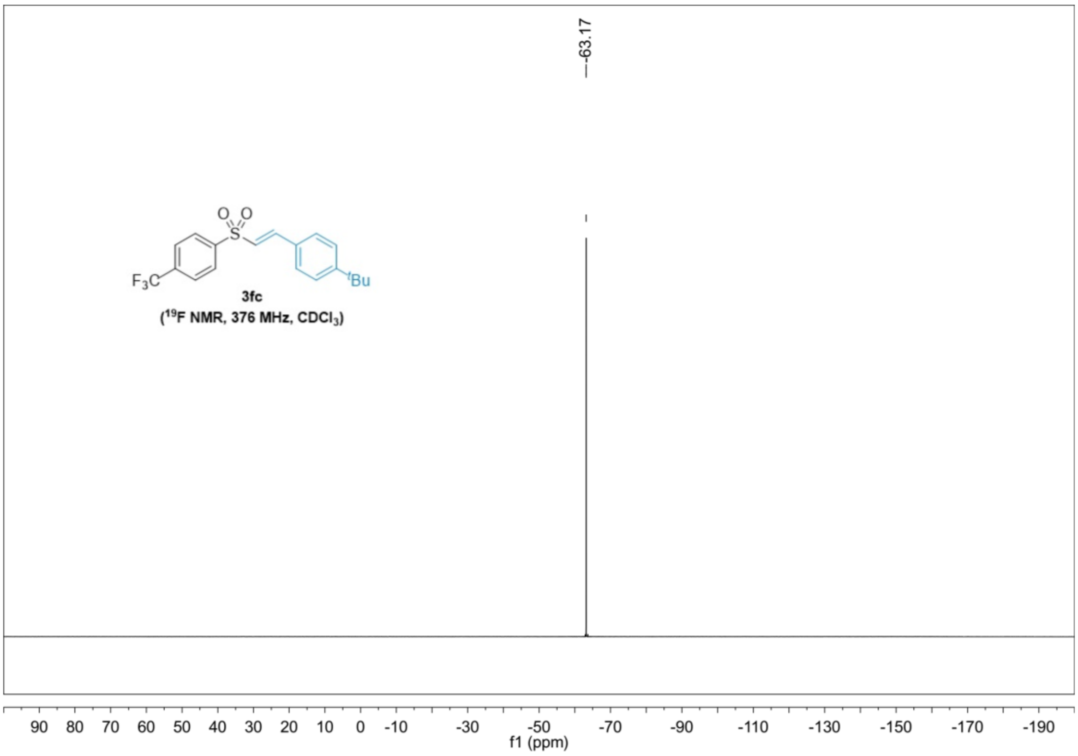

-63.17
F3C
3fc
(19F NMR, 376 MHz, CDCl3)
90 80 70 60 50 40 30 20 10 0 -10 -30 -50 -70 -90 -110 -130 -150 -170 -190
f1 (ppm)

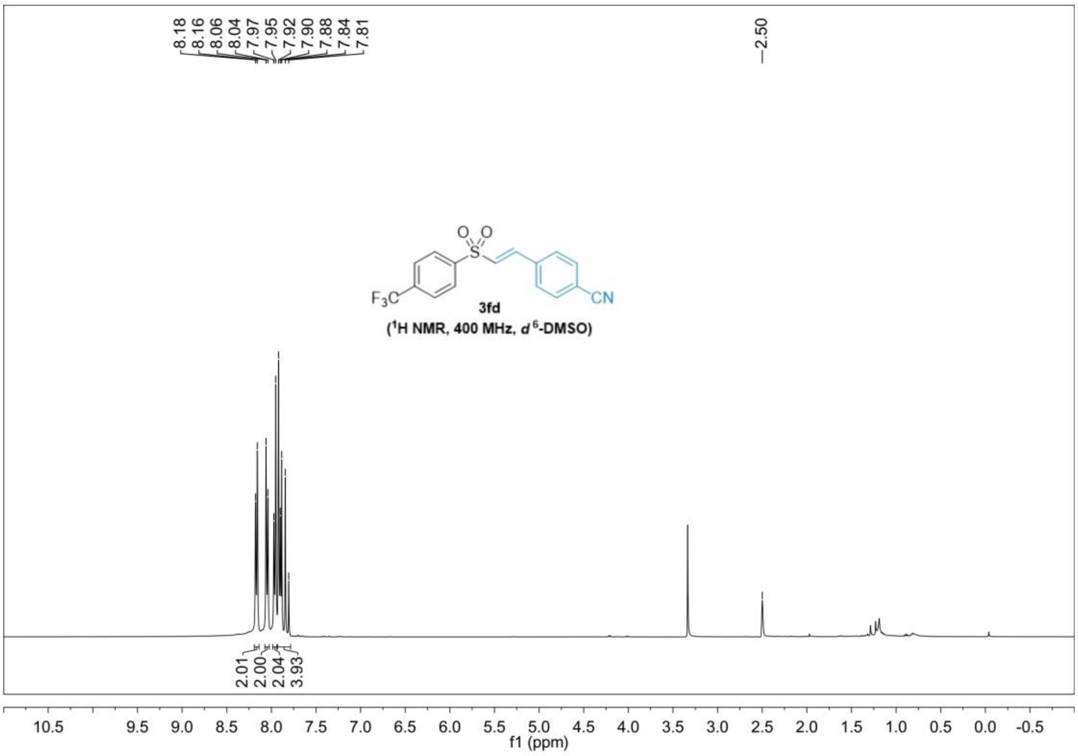

8.18
8.16
8.06
8.04
7.97
7.95
7.92
7.90
7.88
7.84
7.81
2.50
F3C
CN
3fd
(1H NMR, 400 MHz, d6-DMSO)
2.01
2.00
2.04
3.93
10.5 9.5 9.0 8.5 8.0 7.5 7.0 6.5 6.0 5.5 5.0 4.5 4.0 3.5 3.0 2.5 2.0 1.5 1.0 0.5 0.0 -0.5
f1 (ppm)

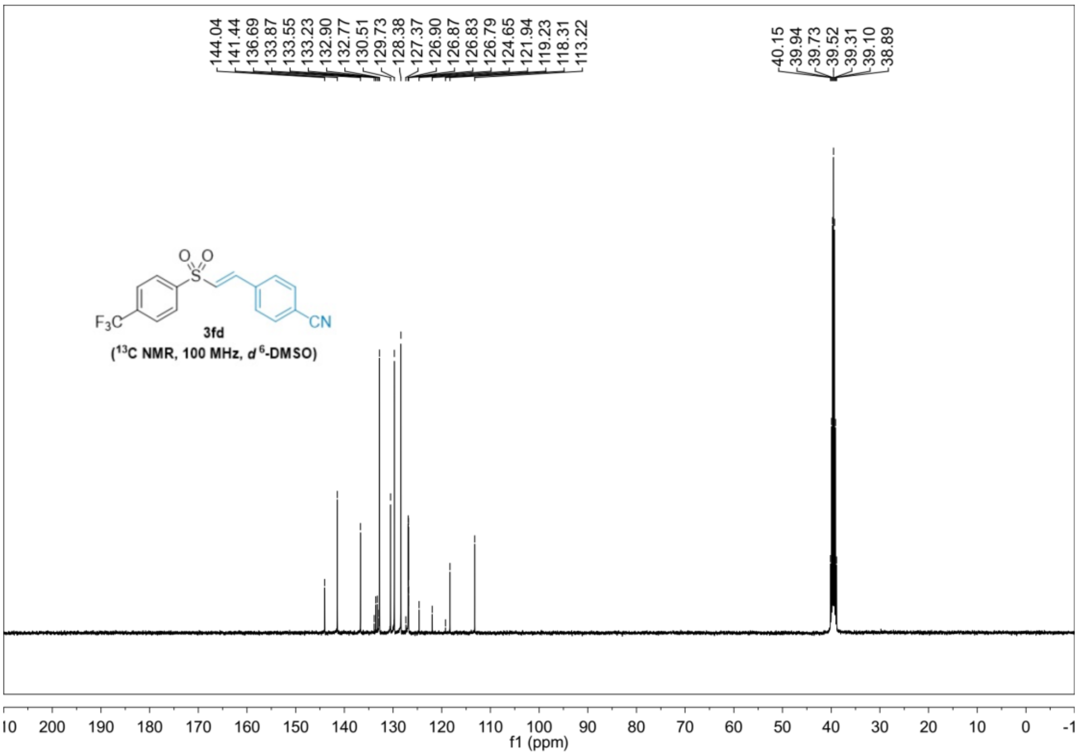

144.04
141.44
136.69
133.87
133.55
133.23
132.90
132.77
130.51
129.73
128.38
127.37
126.90
126.87
126.83
126.79
124.65
121.94
119.23
118.31
113.22
40.15
39.94
39.73
39.52
39.31
39.10
38.89
F3C
CN
3fd
(13C NMR, 100 MHz, d6-DMSO)
f1 (ppm)

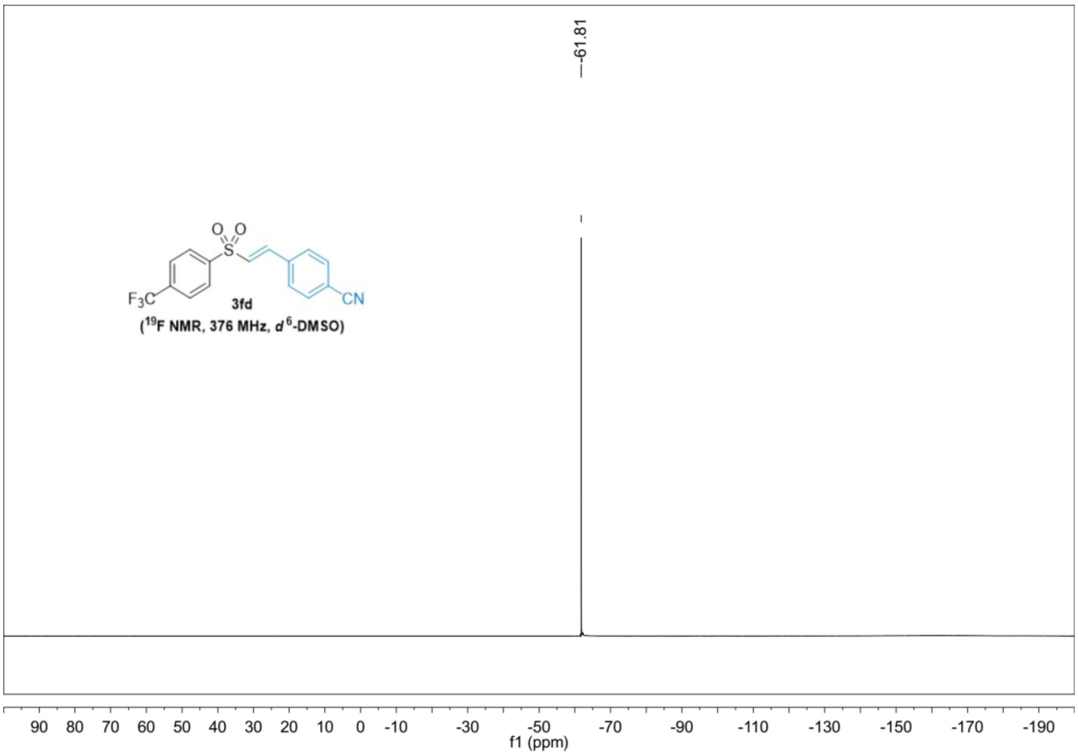

-61.81
F3C
CN
3fd
(19F NMR, 376 MHz, d6-DMSO)
f1 (ppm)

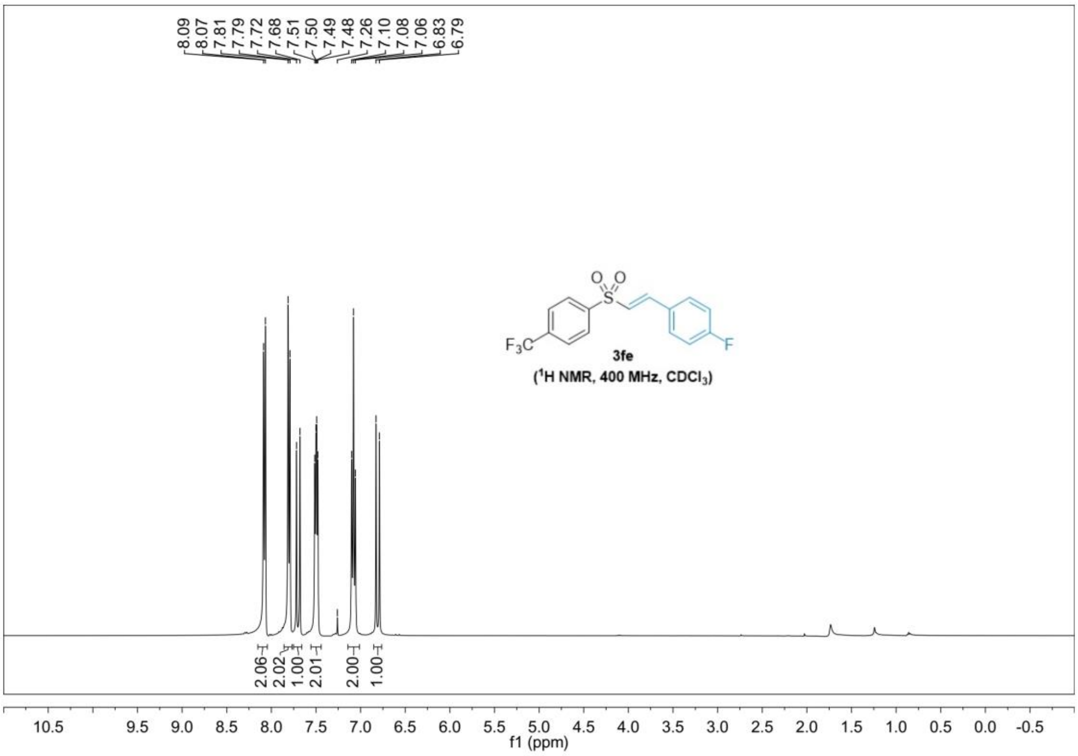
8.09
8.07
7.81
7.79
7.72
7.68
7.51
7.50
7.49
7.48
7.26
7.10
7.08
7.06
6.83
6.79
2.06
2.02
1.00
2.01
2.00
1.00
F3C
3fe
(1H NMR, 400 MHz, CDCl3)
10.5 9.5 9.0 8.5 8.0 7.5 7.0 6.5 6.0 5.5 5.0 4.5 4.0 3.5 3.0 2.5 2.0 1.5 1.0 0.5 0.0 -0.5
f1 (ppm)

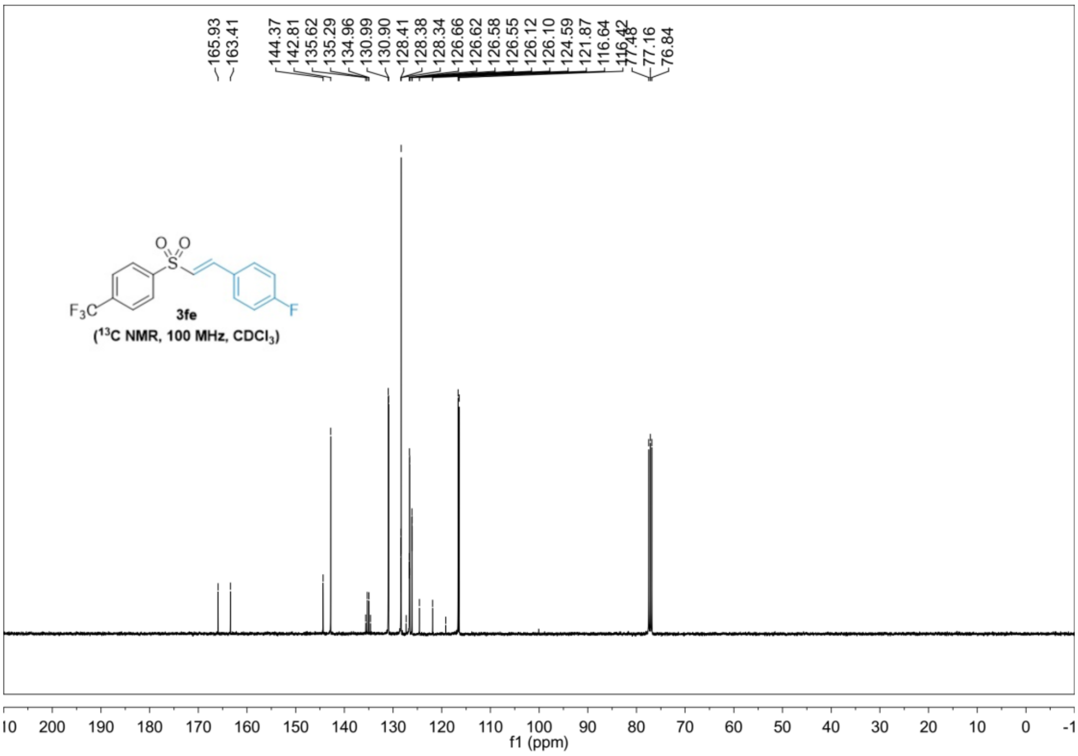
165.93
163.41
144.37
142.81
135.62
135.29
134.96
130.99
130.90
128.41
128.38
128.34
126.66
126.62
126.58
126.55
126.12
126.10
124.59
121.87
116.64
116.42
77.48
77.16
76.84
F3C
3fe
(13C NMR, 100 MHz, CDCl3)
200 190 180 170 160 150 140 130 120 110 100 90 80 70 60 50 40 30 20 10 0
f1 (ppm)

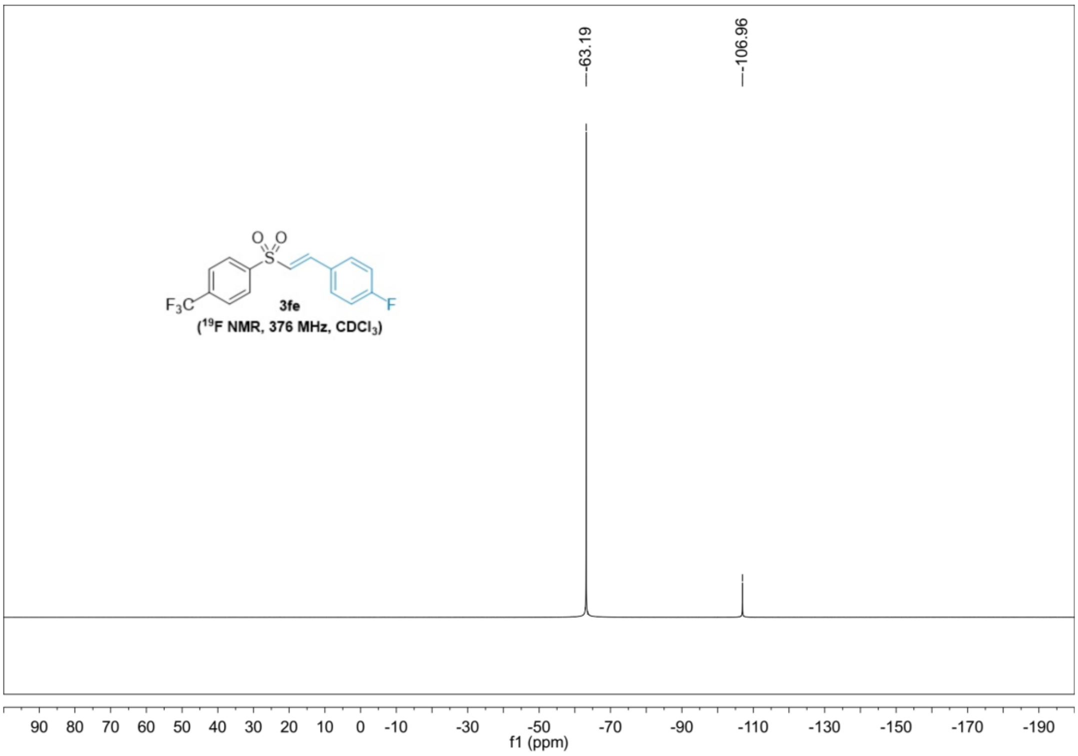
-63.19
-106.96
F3C
3fe
(19F NMR, 376 MHz, CDCl3)
90 80 70 60 50 40 30 20 10 0 -10 -30 -50 -70 -90 -110 -130 -150 -170 -190
f1 (ppm)

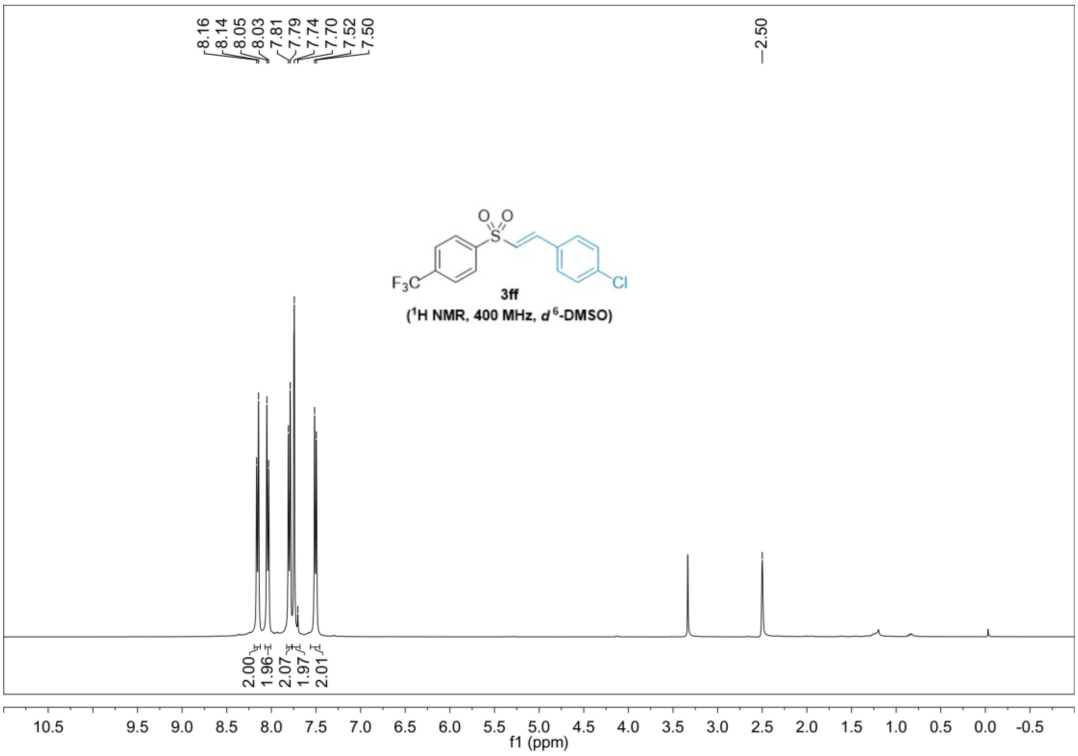
8.16
8.14
8.05
8.03
7.81
7.79
7.74
7.70
7.52
7.50
2.50
F3C
Cl
3ff
(1H NMR, 400 MHz, d6-DMSO)
2.00
1.96
2.07
1.97
2.01
10.5 9.5 9.0 8.5 8.0 7.5 7.0 6.5 6.0 5.5 5.0 4.5 4.0 3.5 3.0 2.5 2.0 1.5 1.0 0.5 0.0 -0.5
f1 (ppm)

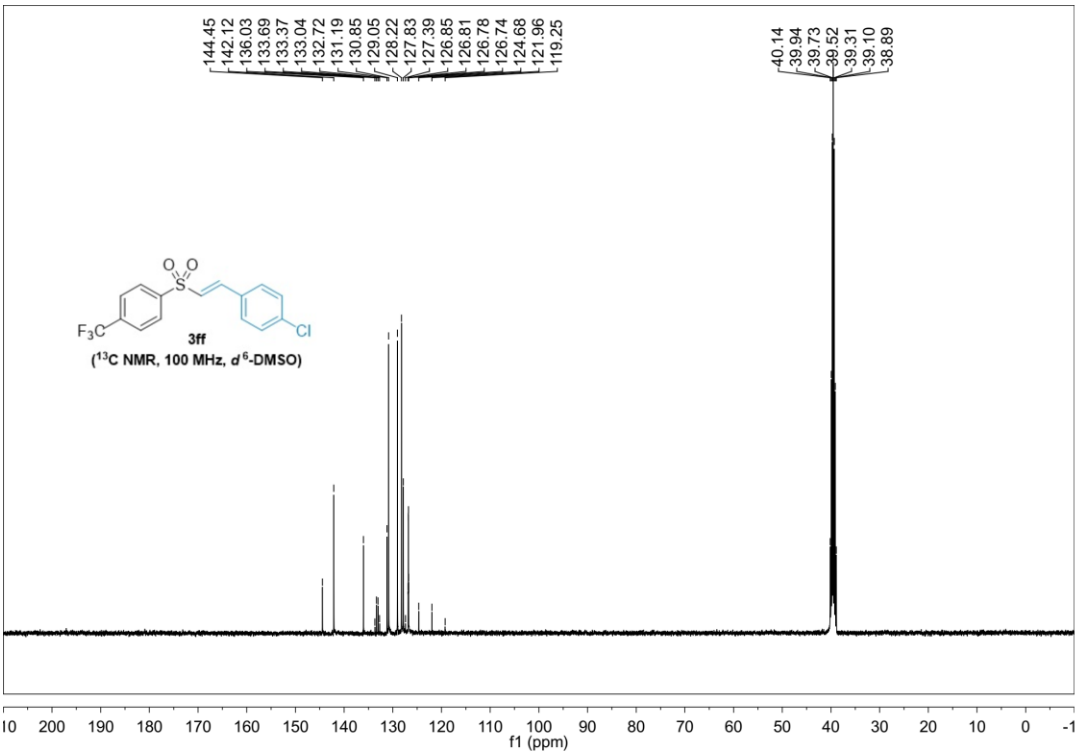
144.45
142.12
136.03
133.69
133.37
133.04
132.72
131.19
130.85
129.05
128.22
127.83
127.39
126.85
126.81
126.78
126.74
124.68
121.96
119.25
40.14
39.94
39.73
39.52
39.31
39.10
38.89
3ff
(13C NMR, 100 MHz, d6-DMSO)
f1 (ppm)

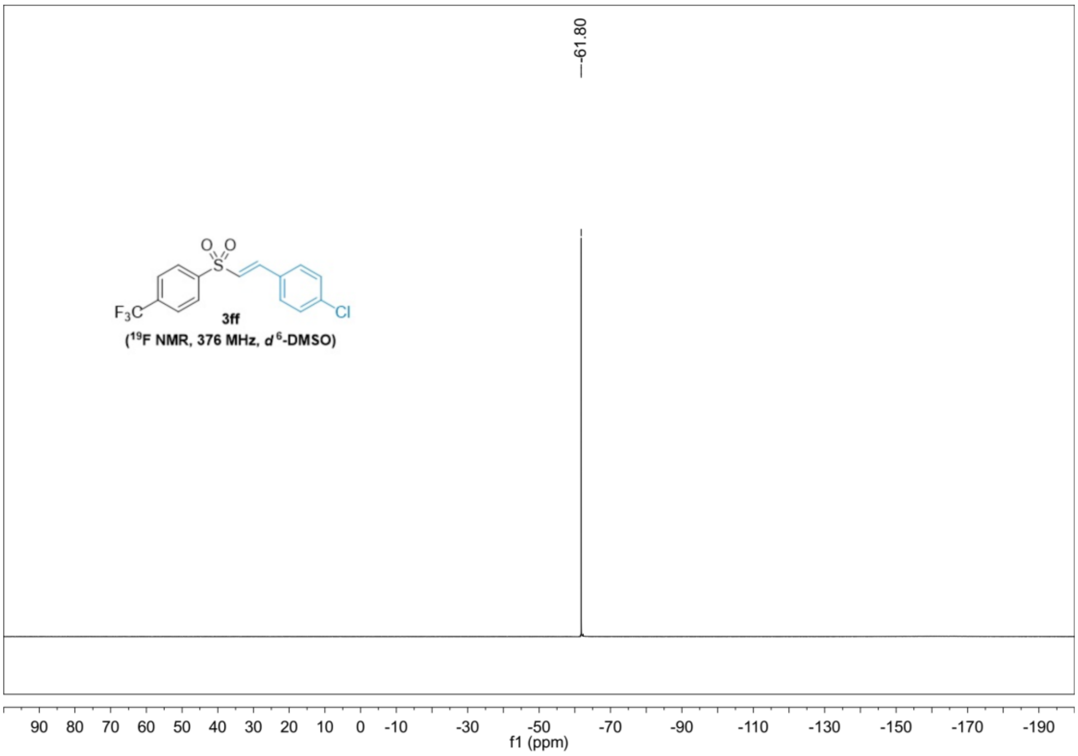
-61.80
3ff
(19F NMR, 376 MHz, d6-DMSO)
f1 (ppm)

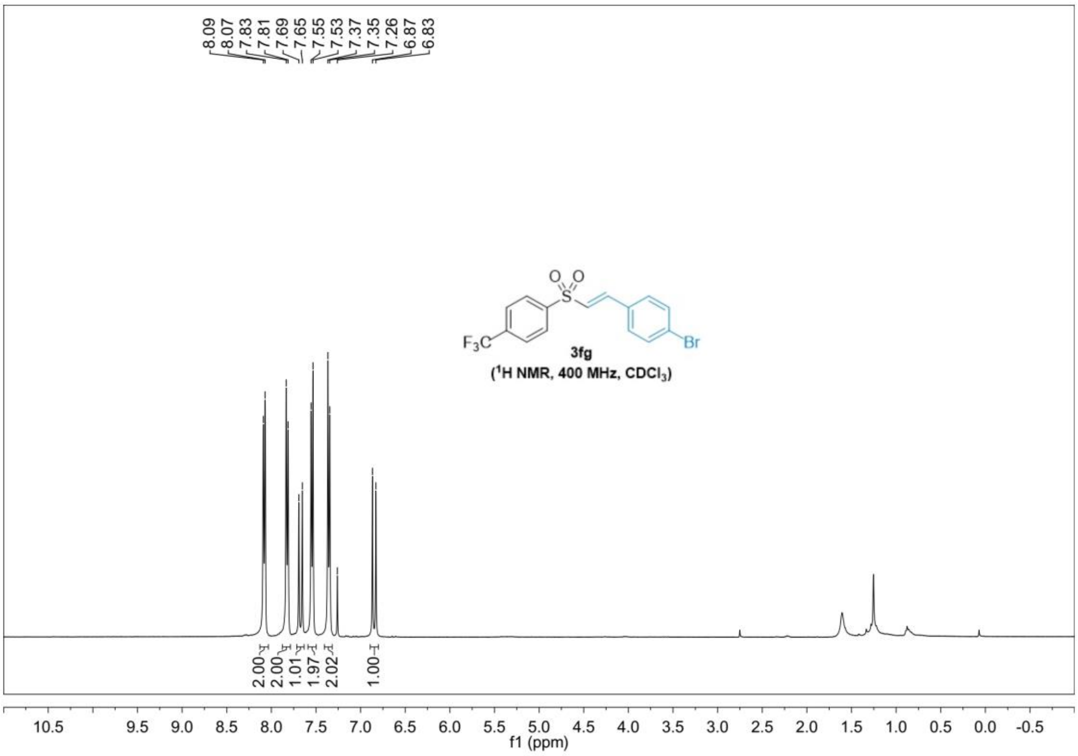
8.09
8.07
7.83
7.81
7.69
7.65
7.55
7.53
7.37
7.35
7.26
6.87
6.83
F3C
Br
3fg
(1H NMR, 400 MHz, CDCl3)
2.00
2.00
1.01
1.97
2.02
1.00
f1 (ppm)

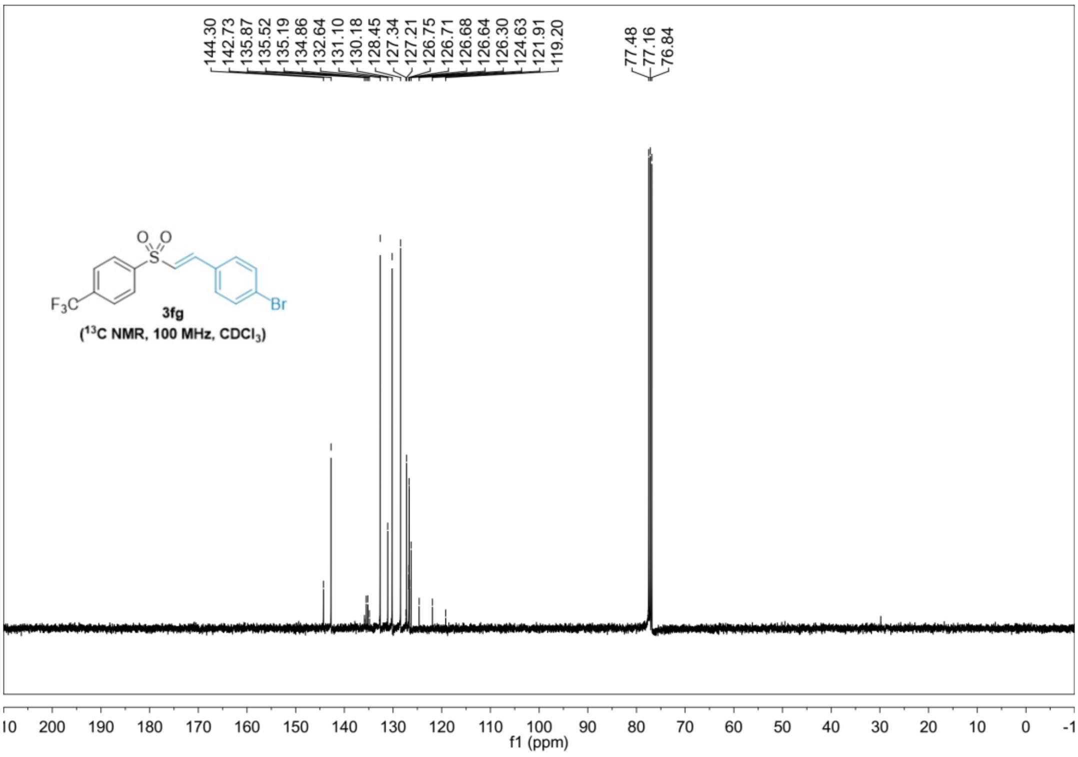
144.30
142.73
135.87
135.52
135.19
134.86
132.64
131.10
130.18
128.45
127.34
127.21
126.75
126.71
126.68
126.64
126.30
124.63
121.91
119.20
77.48
77.16
76.84
F3C
Br
3fg
(13C NMR, 100 MHz, CDCl3)
f1 (ppm)

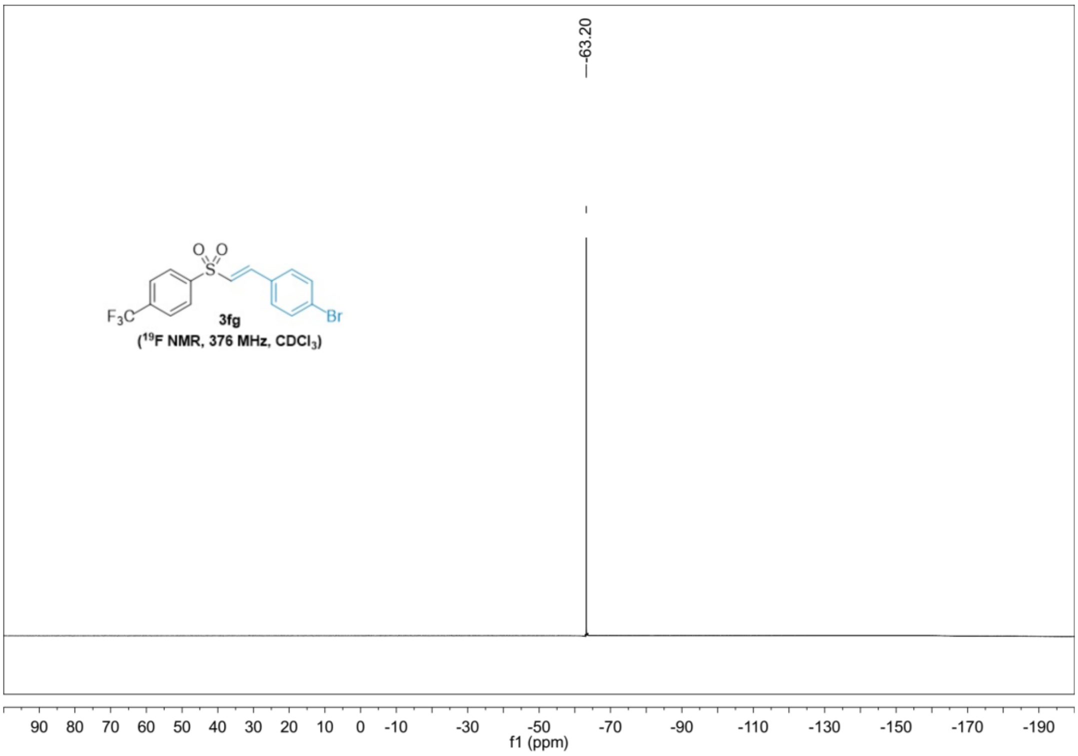

-63.20
F3C
3fg
(19F NMR, 376 MHz, CDCl3)
Br
90 80 70 60 50 40 30 20 10 0 -10 -30 -50 -70 -90 -110 -130 -150 -170 -190
f1 (ppm)

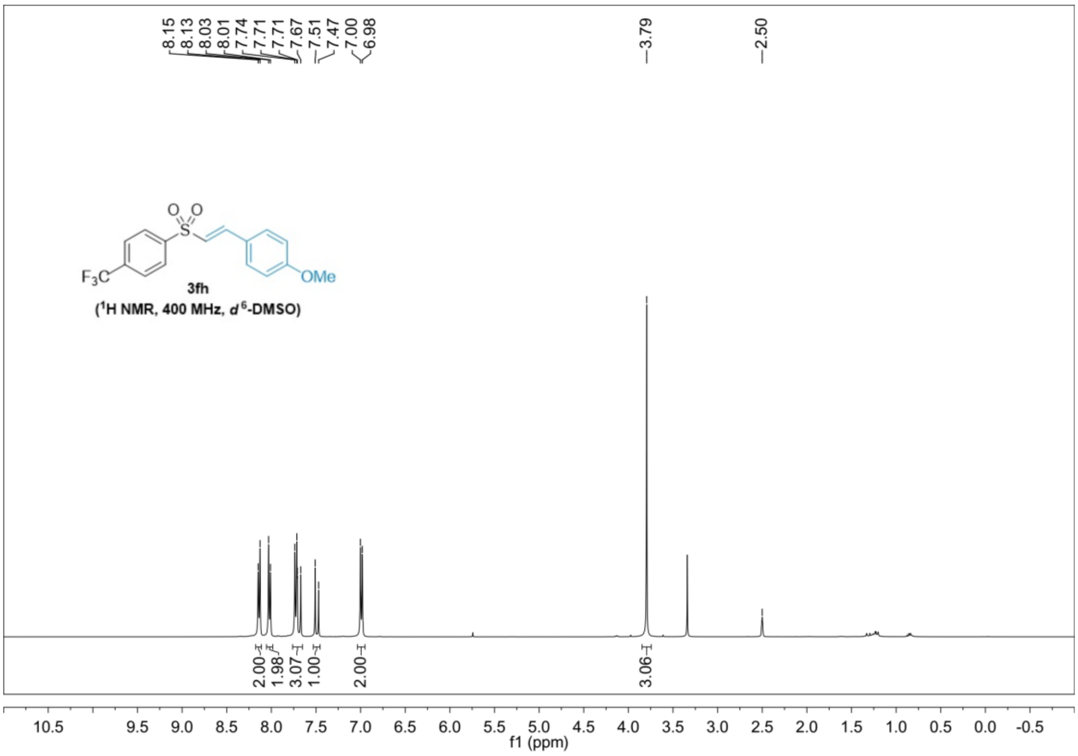

8.15
8.13
8.03
8.01
7.74
7.71
7.71
7.67
7.51
7.47
7.00
6.98
3.79
2.50
F3C
OMe
3fh
(1H NMR, 400 MHz, d6-DMSO)
2.00
1.98
3.07
1.00
2.00
3.06
10.5 9.5 9.0 8.5 8.0 7.5 7.0 6.5 6.0 5.5 5.0 4.5 4.0 3.5 3.0 2.5 2.0 1.5 1.0 0.5 0.0 -0.5
f1 (ppm)

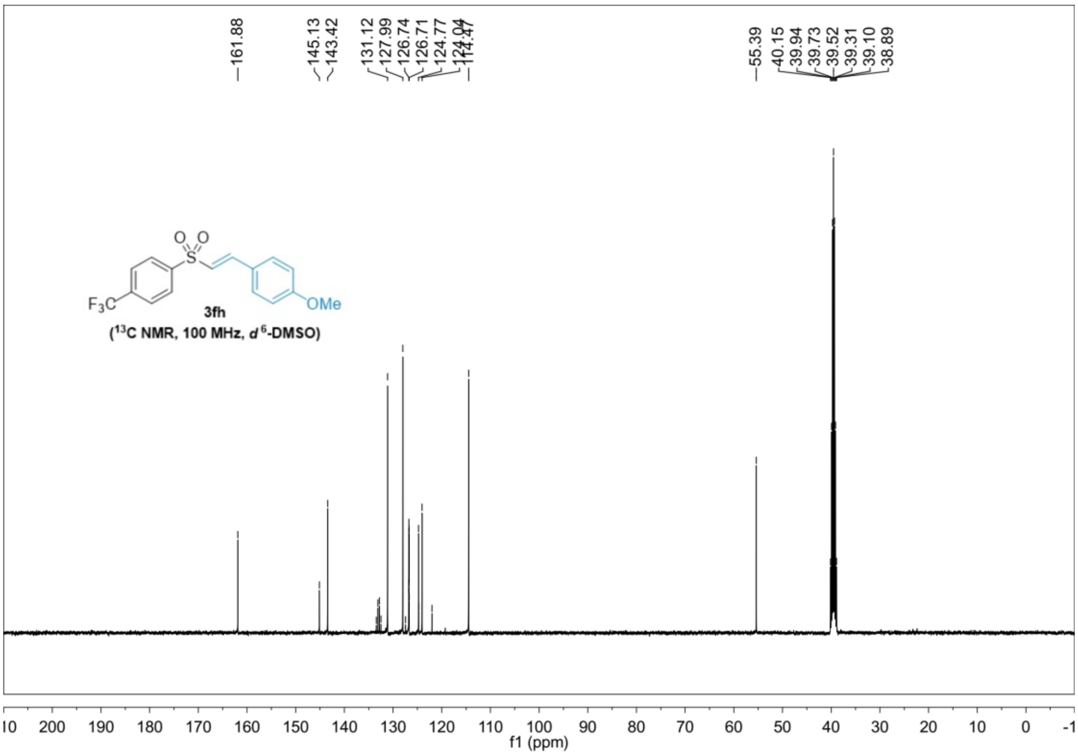
161.88
145.13
143.42
131.12
127.99
126.74
126.71
124.77
124.04
114.47
55.39
40.15
39.94
39.73
39.52
39.31
39.10
38.89
F3C
OMe
3fh
(13C NMR, 100 MHz, d6-DMSO)
f1 (ppm)

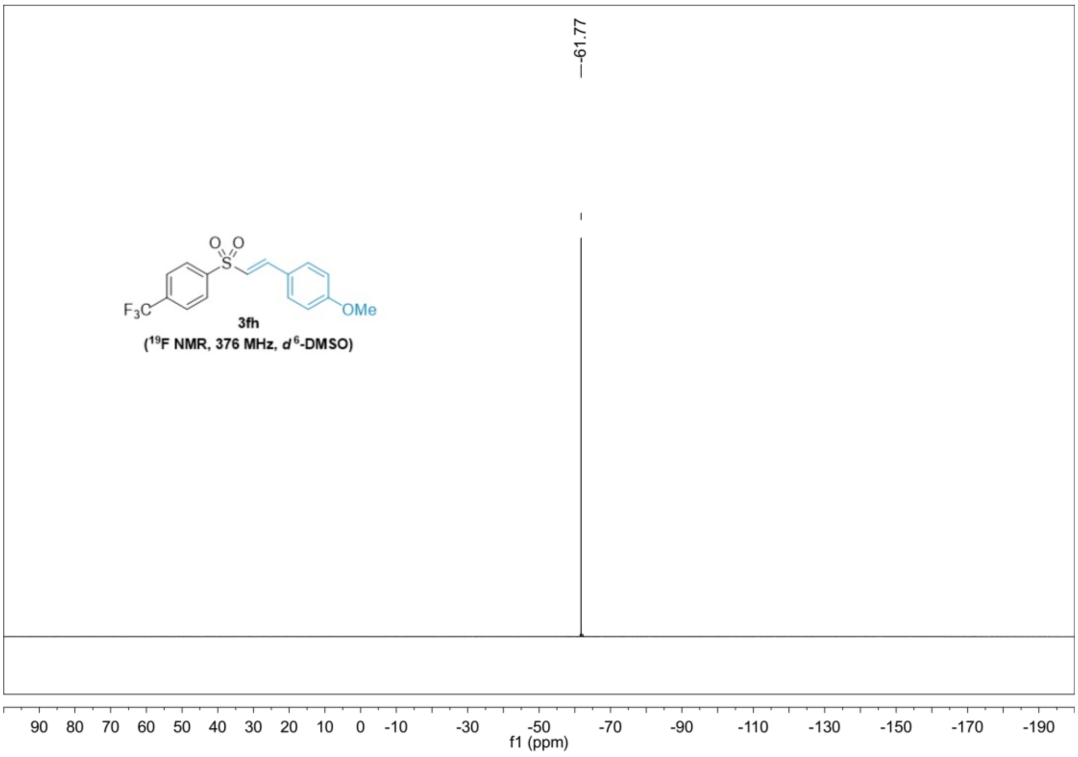
-61.77
F3C
OMe
3fh
(19F NMR, 376 MHz, d6-DMSO)
f1 (ppm)

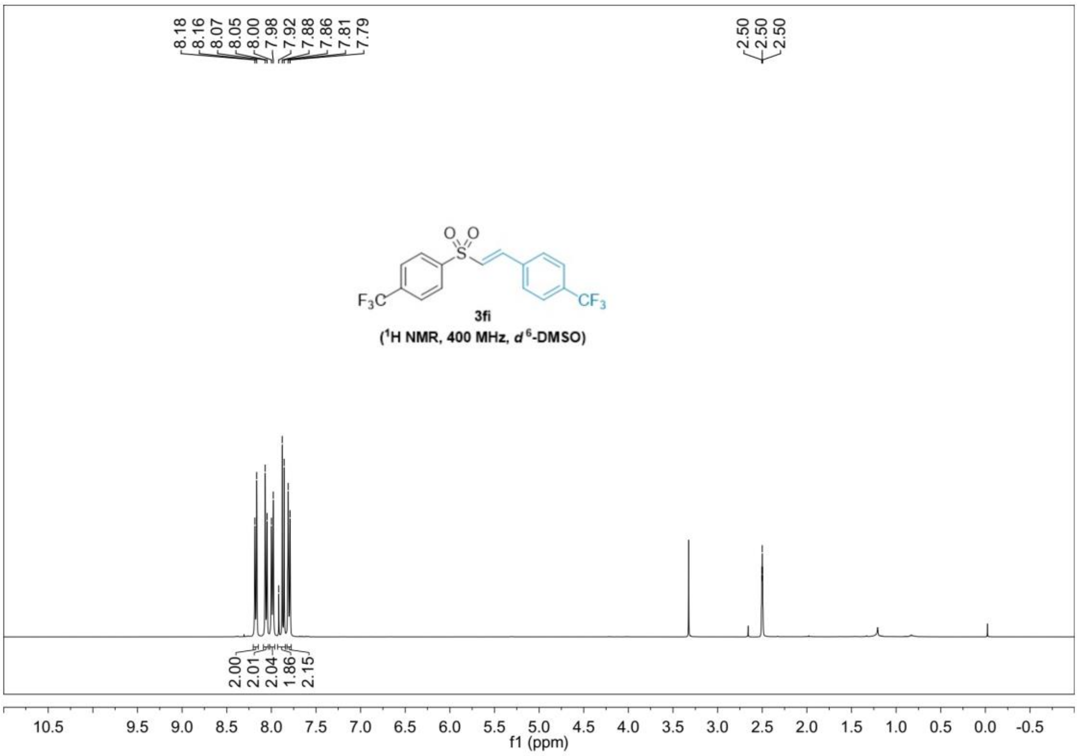
8.18
8.16
8.07
8.05
8.00
7.98
7.92
7.88
7.86
7.81
7.79
2.50
2.50
2.50
3fi
(1H NMR, 400 MHz, d6-DMSO)
2.00
2.01
2.04
1.86
2.15
f1 (ppm)

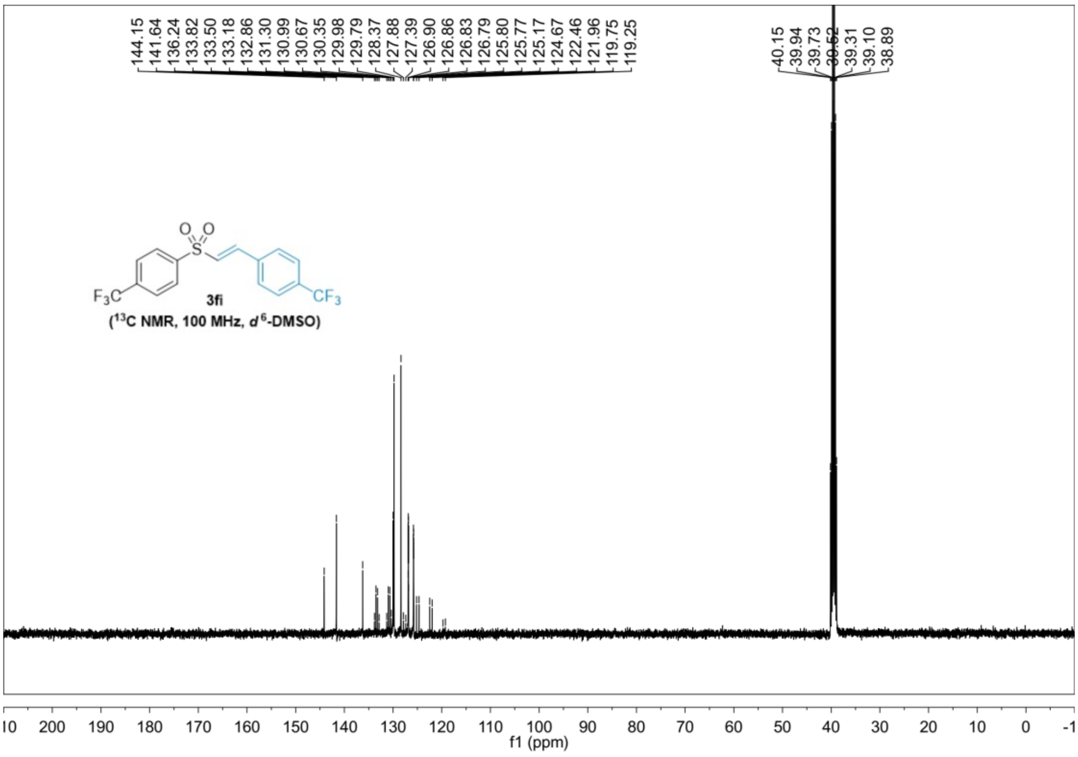
144.15
141.64
136.24
133.82
133.50
133.18
132.86
131.30
130.99
130.67
130.35
129.98
129.79
128.37
127.88
127.39
126.90
126.86
126.83
126.79
125.80
125.77
125.17
124.67
122.46
121.96
119.75
119.25
40.15
39.94
39.73
39.52
39.31
39.10
38.89
3fi
(13C NMR, 100 MHz, d6-DMSO)
f1 (ppm)

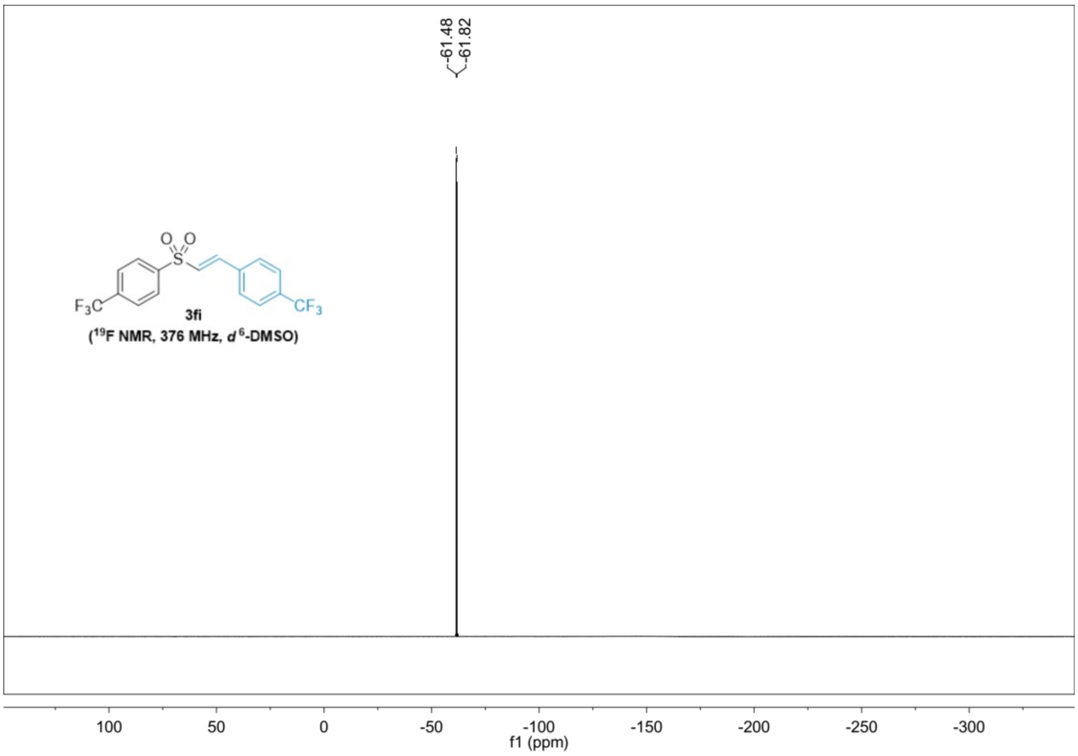

-61.48
-61.82
3fi
(^{19}F NMR, 376 MHz, d^6-DMSO)
100 50 0 -50 -100 -150 -200 -250 -300
f1 (ppm)

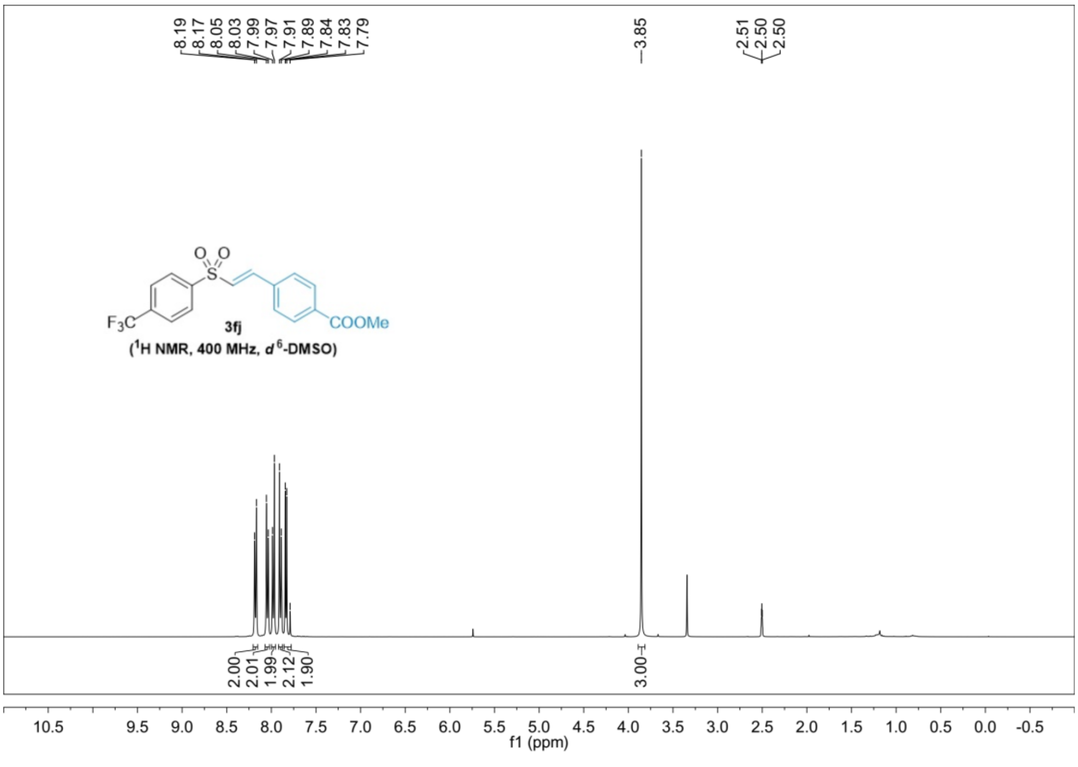

8.19
8.17
8.05
8.03
7.99
7.97
7.91
7.89
7.84
7.83
7.79
3.85
2.51
2.50
2.50
3fj
(^{1}H NMR, 400 MHz, d^6-DMSO)
2.00
2.01
1.99
2.12
1.90
3.00
10.5 9.5 9.0 8.5 8.0 7.5 7.0 6.5 6.0 5.5 5.0 4.5 4.0 3.5 3.0 2.5 2.0 1.5 1.0 0.5 0.0 -0.5
f1 (ppm)

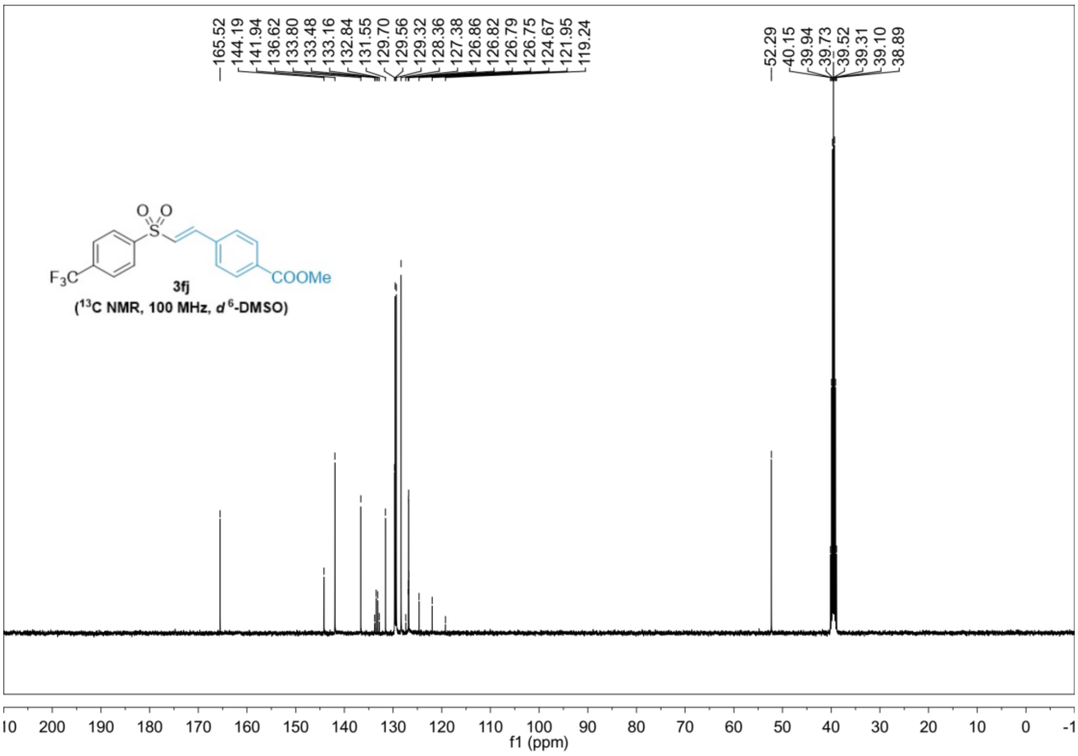
165.52
144.19
141.94
136.62
133.80
133.48
133.16
132.84
131.55
129.70
129.56
129.32
128.36
127.38
126.86
126.82
126.79
126.75
124.67
121.95
119.24
52.29
40.15
39.94
39.73
39.52
39.31
39.10
38.89
F3C
COOMe
3fj
(13C NMR, 100 MHz, d6-DMSO)
f1 (ppm)

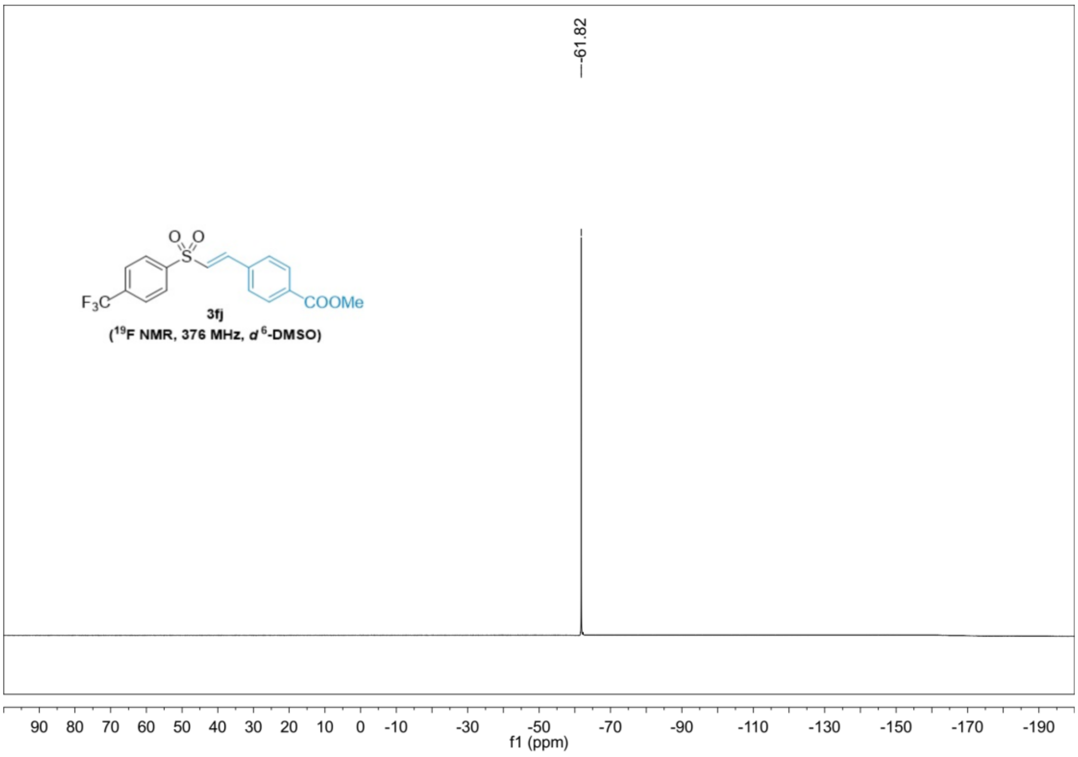
-61.82
F3C
COOMe
3fj
(19F NMR, 376 MHz, d6-DMSO)
f1 (ppm)

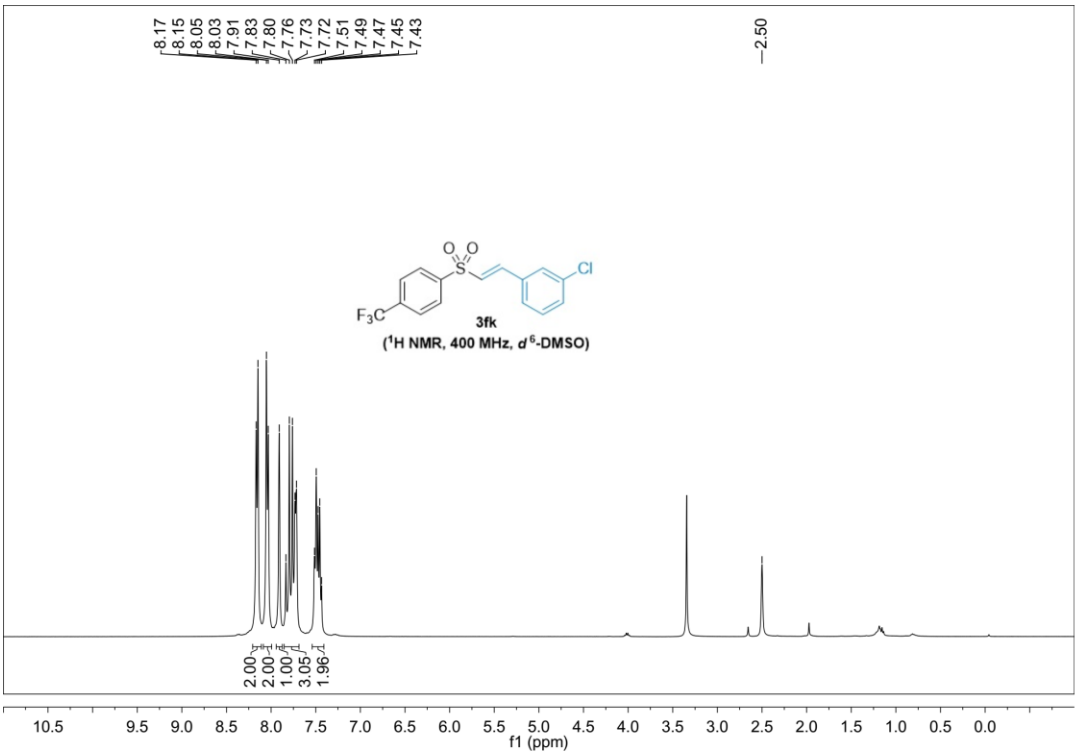
8.17
8.15
8.05
8.03
7.91
7.83
7.80
7.76
7.73
7.72
7.51
7.49
7.47
7.45
7.43
2.50
F_3C
S
O
O
Cl
3fk
(^{1}H NMR, 400 MHz, d^6-DMSO)
2.00
2.00
1.00
3.05
1.96
f1 (ppm)

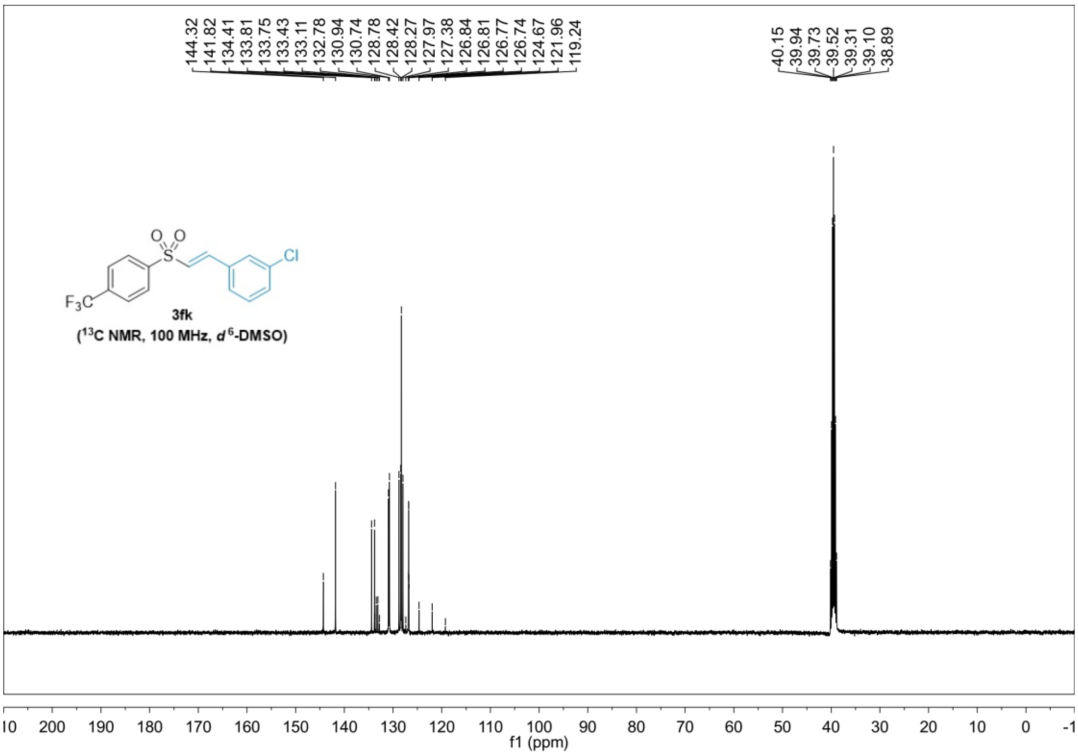
144.32
141.82
134.41
133.81
133.75
133.43
133.11
132.78
130.94
130.74
128.78
128.42
128.27
127.97
127.38
126.84
126.81
126.77
126.74
124.67
121.96
119.24
40.15
39.94
39.73
39.52
39.31
39.10
38.89
F_3C
S
O
O
Cl
3fk
(^{13}C NMR, 100 MHz, d^6-DMSO)
f1 (ppm)

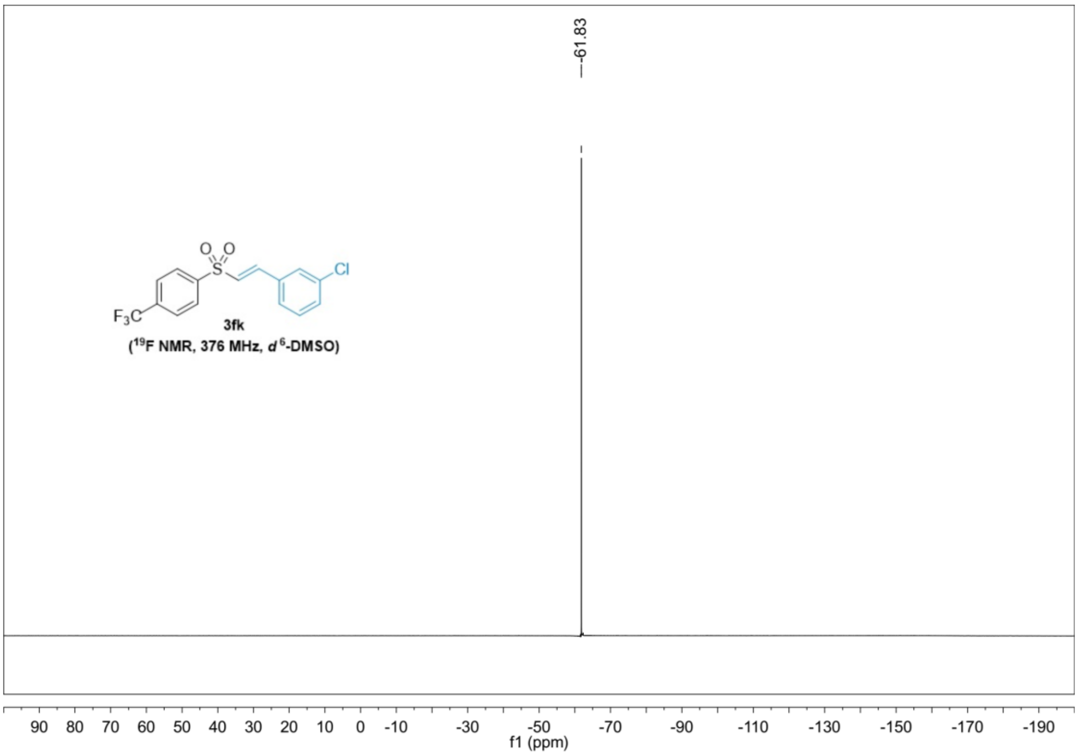

-61.83
3fk
(19F NMR, 376 MHz, d6-DMSO)
f1 (ppm)

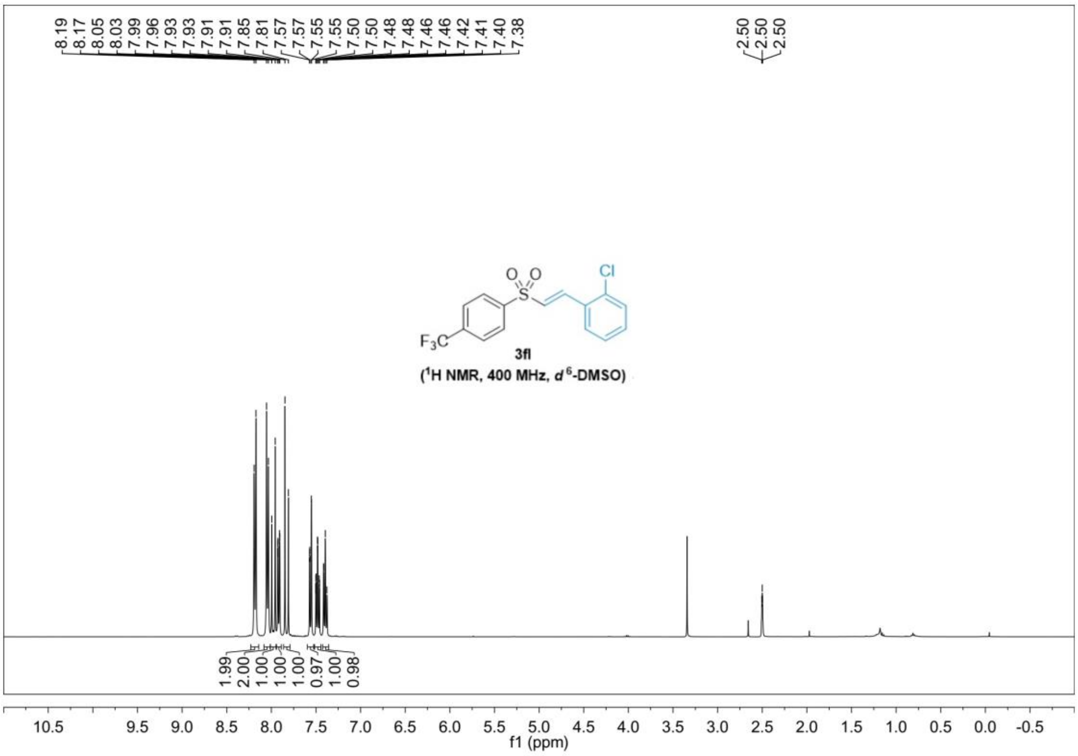

8.19 8.17 8.05 8.03 7.99 7.96 7.93 7.93 7.91 7.85 7.81 7.57 7.57 7.55 7.55 7.50 7.48 7.48 7.46 7.42 7.41 7.40 7.38
2.50 2.50 2.50
3fl
(1H NMR, 400 MHz, d6-DMSO)
1.99 2.00 1.00 1.00 1.00 0.97 1.00 0.98
f1 (ppm)

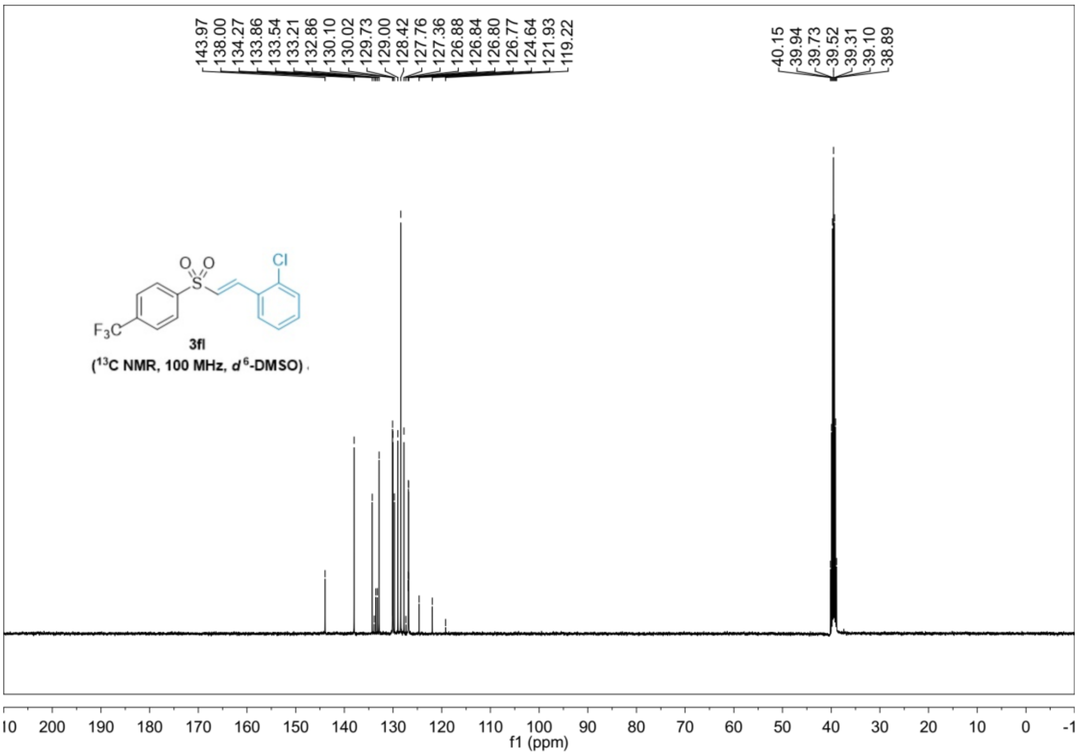
143.97
138.00
134.27
133.86
133.54
133.21
132.86
130.10
130.02
129.73
129.00
128.42
127.76
127.36
126.88
126.84
126.80
126.77
124.64
121.93
119.22
40.15
39.94
39.73
39.52
39.31
39.10
38.89
Cl
F_3C
3fl
(^{13}C NMR, 100 MHz, d^6-DMSO)
f1 (ppm)

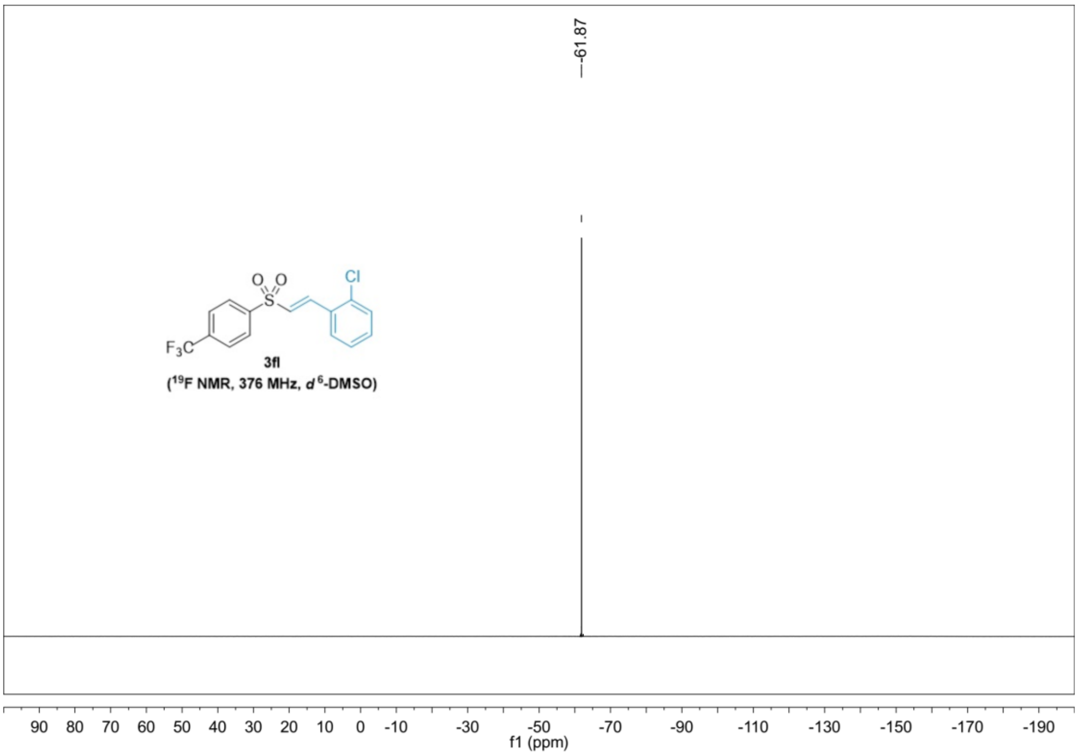
-61.87
Cl
F_3C
3fl
(^{19}F NMR, 376 MHz, d^6-DMSO)
f1 (ppm)

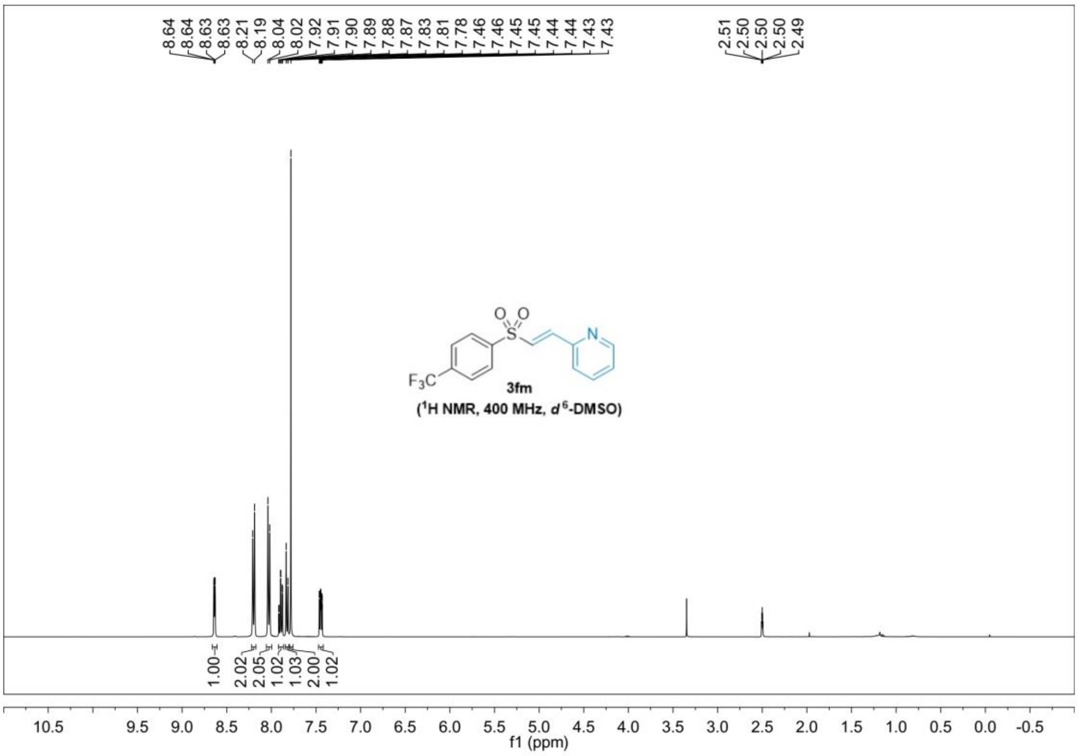
8.64
8.64
8.63
8.63
8.21
8.19
8.04
8.02
7.92
7.91
7.90
7.89
7.88
7.87
7.83
7.81
7.78
7.46
7.46
7.45
7.45
7.44
7.44
7.43
7.43
2.51
2.50
2.50
2.49
3fm
(1H NMR, 400 MHz, d6-DMSO)
1.00
2.02
2.05
1.02
1.03
2.00
1.02
f1 (ppm)

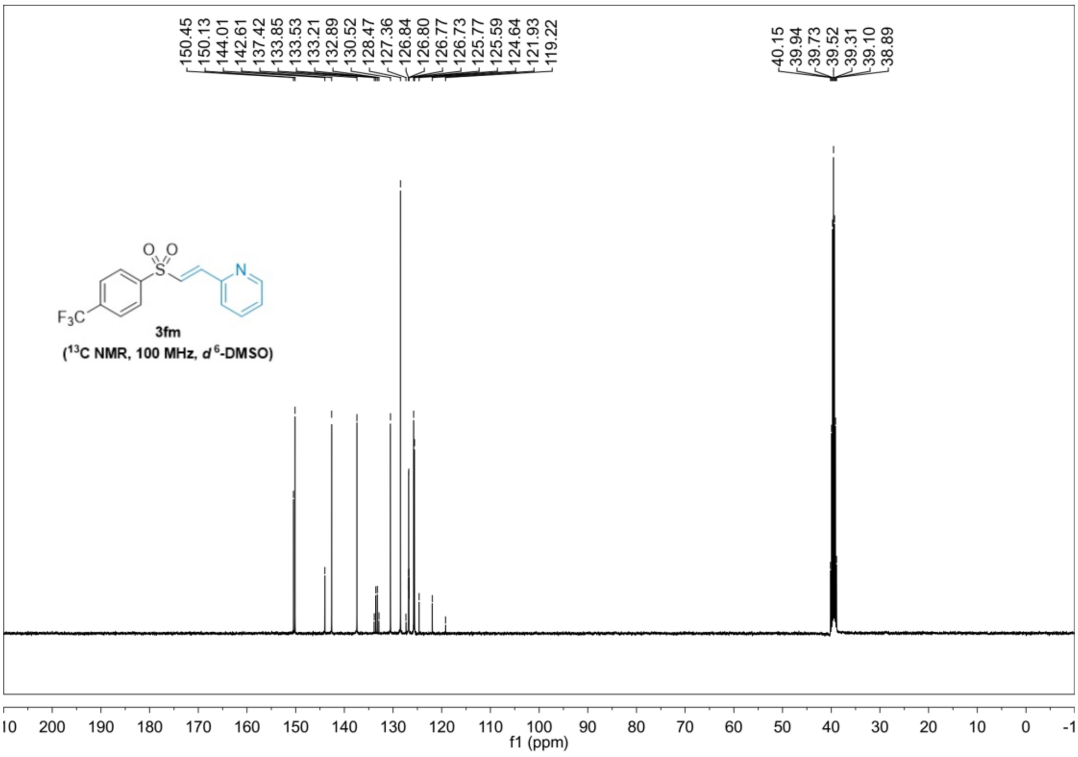
150.45
150.13
144.01
142.61
137.42
133.85
133.53
133.21
132.89
130.52
128.47
127.36
126.84
126.80
126.77
126.73
125.77
125.59
124.64
121.93
119.22
40.15
39.94
39.73
39.52
39.31
39.10
38.89
3fm
(13C NMR, 100 MHz, d6-DMSO)
f1 (ppm)

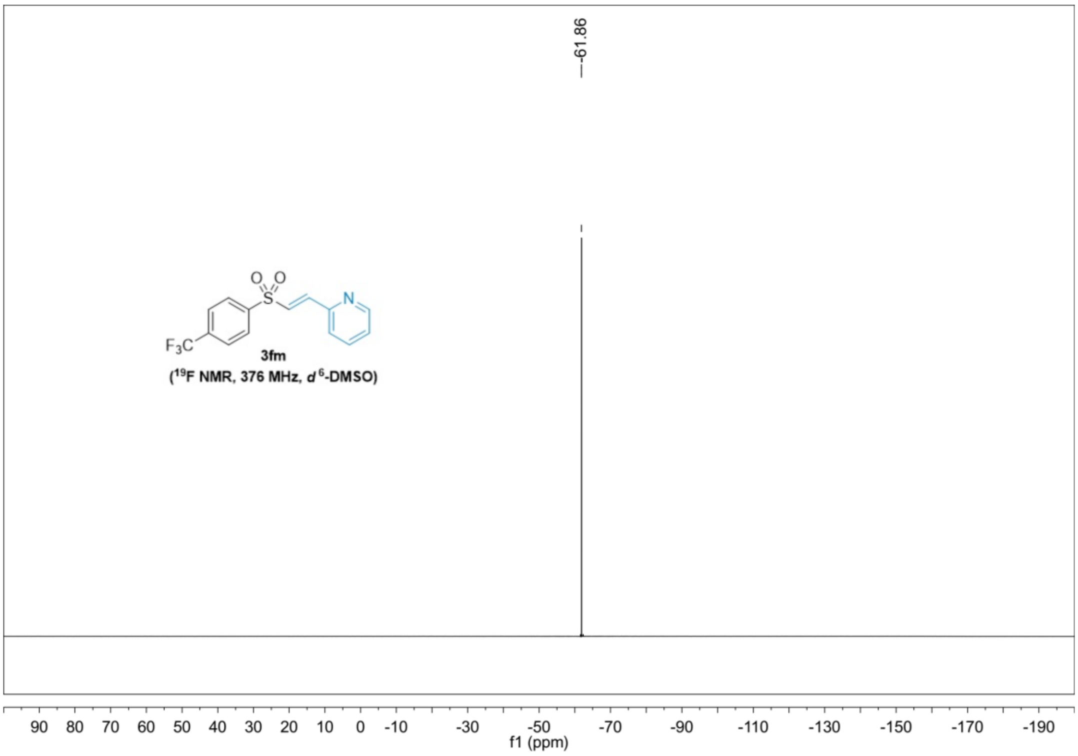
-61.86
F3C
N
3fm
(19F NMR, 376 MHz, d6-DMSO)
90 80 70 60 50 40 30 20 10 0 -10 -30 -50 -70 -90 -110 -130 -150 -170 -190
f1 (ppm)

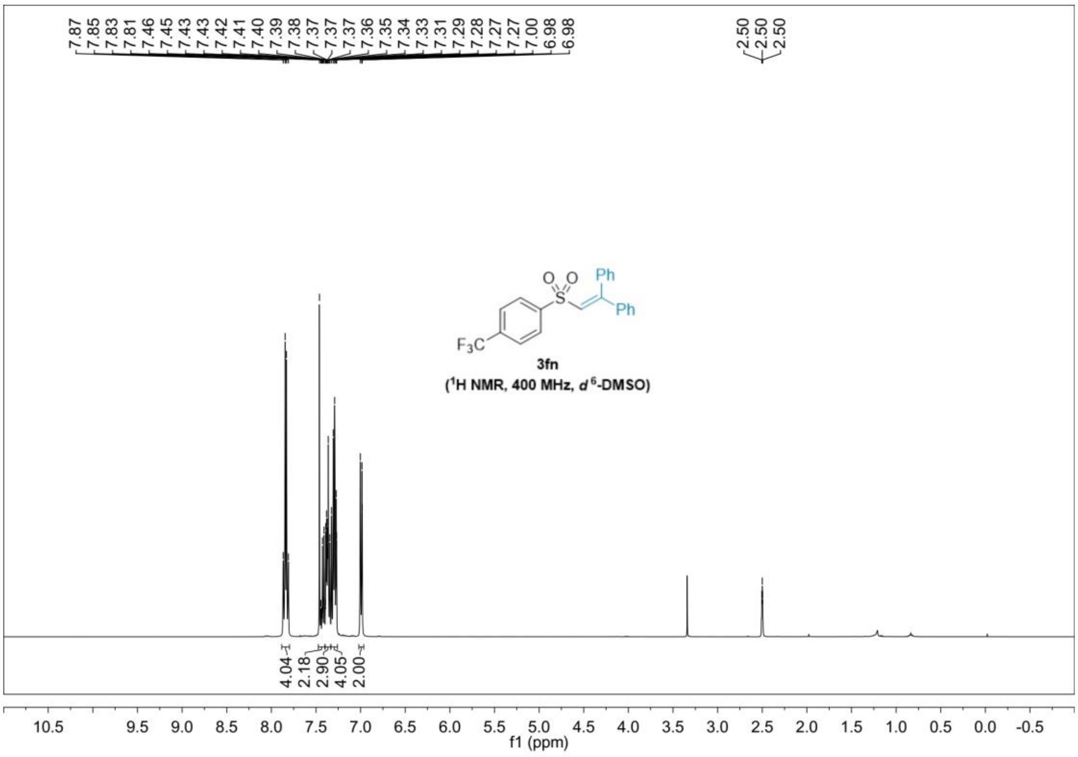
7.87 7.85 7.83 7.81 7.46 7.45 7.43 7.43 7.42 7.41 7.40 7.39 7.38 7.37 7.37 7.37 7.36 7.35 7.34 7.33 7.31 7.29 7.28 7.27 7.27 7.00 6.98 6.98
2.50 2.50 2.50
Ph
Ph
F3C
3fn
(1H NMR, 400 MHz, d6-DMSO)
4.04 2.18 2.90 4.05 2.00
10.5 9.5 9.0 8.5 8.0 7.5 7.0 6.5 6.0 5.5 5.0 4.5 4.0 3.5 3.0 2.5 2.0 1.5 1.0 0.5 0.0 -0.5
f1 (ppm)

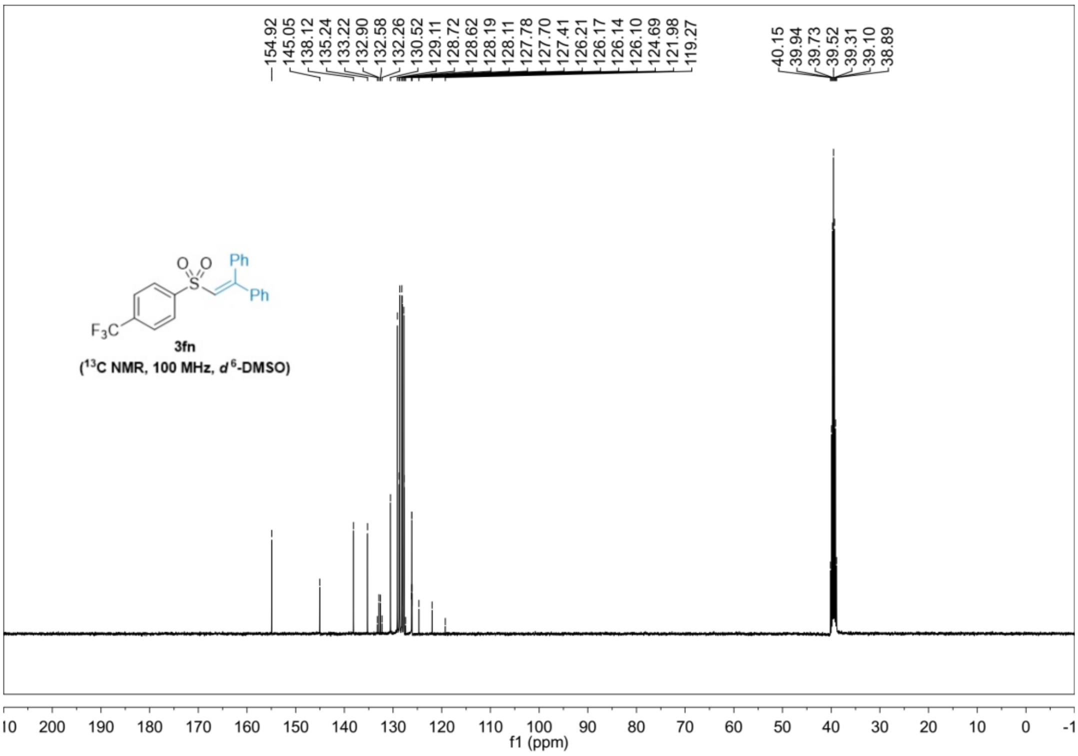

154.92
145.05
138.12
135.24
133.22
132.90
132.58
132.26
130.52
129.11
128.72
128.62
128.19
128.11
127.78
127.70
127.41
126.21
126.17
126.14
126.10
124.69
121.98
119.27
40.15
39.94
39.73
39.52
39.31
39.10
38.89
3fn
(^{13}C NMR, 100 MHz, d^6-DMSO)
f1 (ppm)

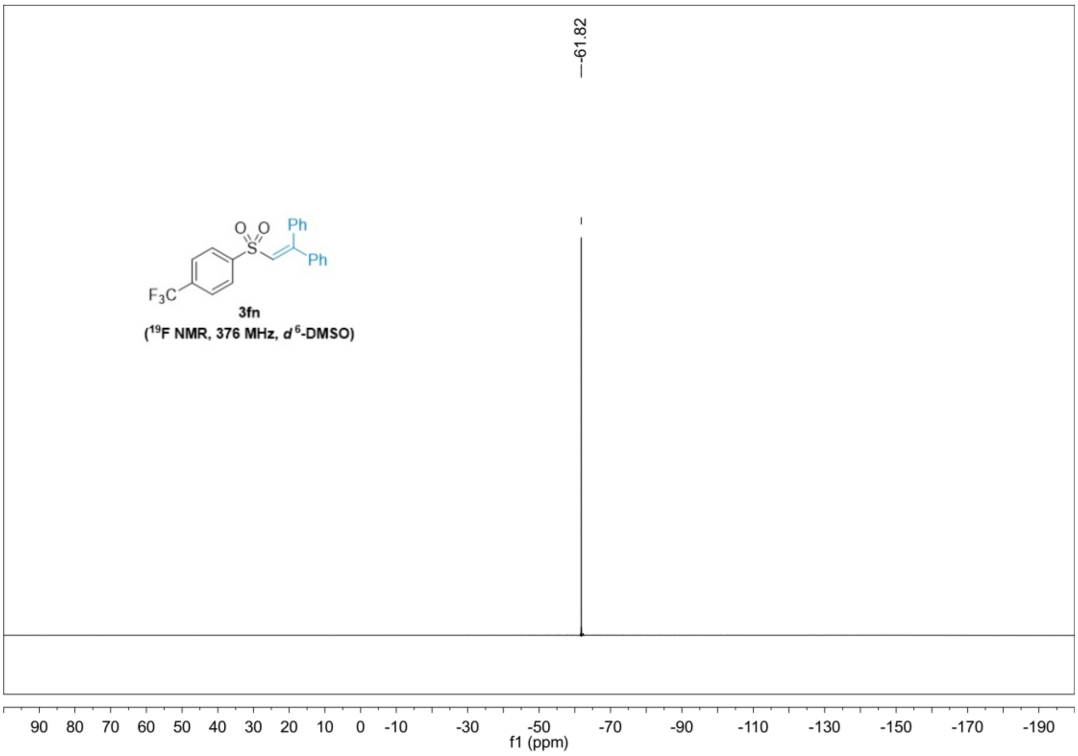

-61.82
3fn
(^{19}F NMR, 376 MHz, d^6-DMSO)
f1 (ppm)

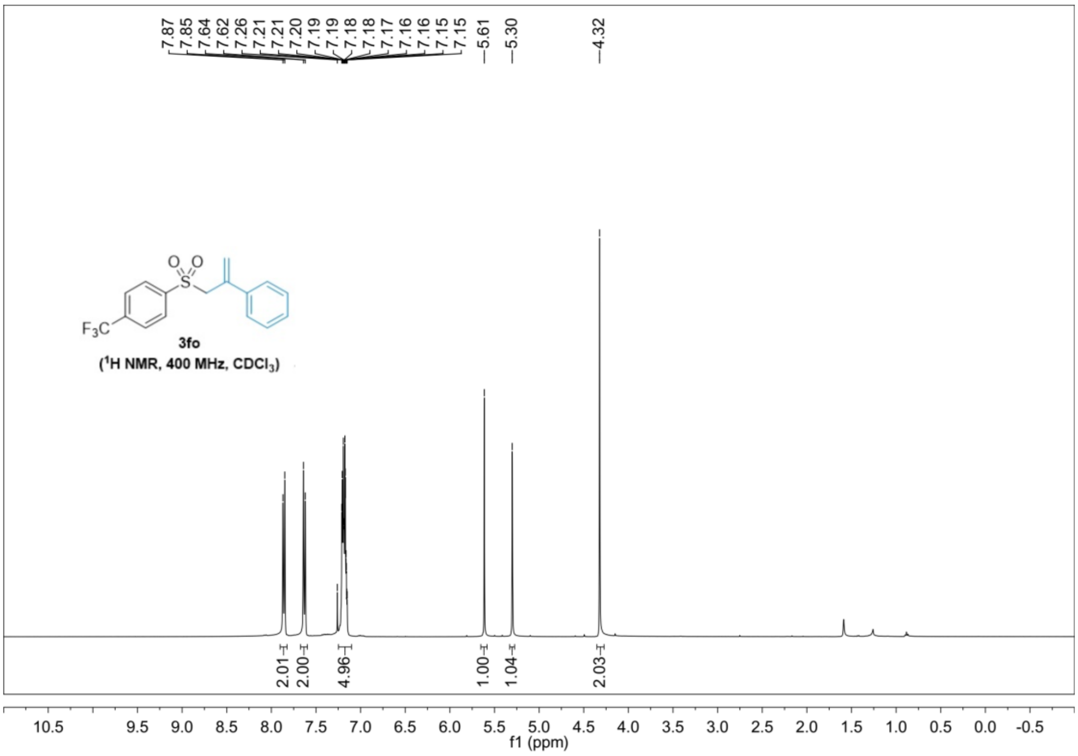
7.87
7.85
7.64
7.62
7.26
7.21
7.21
7.20
7.19
7.19
7.18
7.18
7.17
7.16
7.16
7.15
7.15
5.61
5.30
4.32
F3C
3fo
(1H NMR, 400 MHz, CDCl3)
2.01
2.00
4.96
1.00
1.04
2.03
10.5 9.5 9.0 8.5 8.0 7.5 7.0 6.5 6.0 5.5 5.0 4.5 4.0 3.5 3.0 2.5 2.0 1.5 1.0 0.5 0.0 -0.5
f1 (ppm)

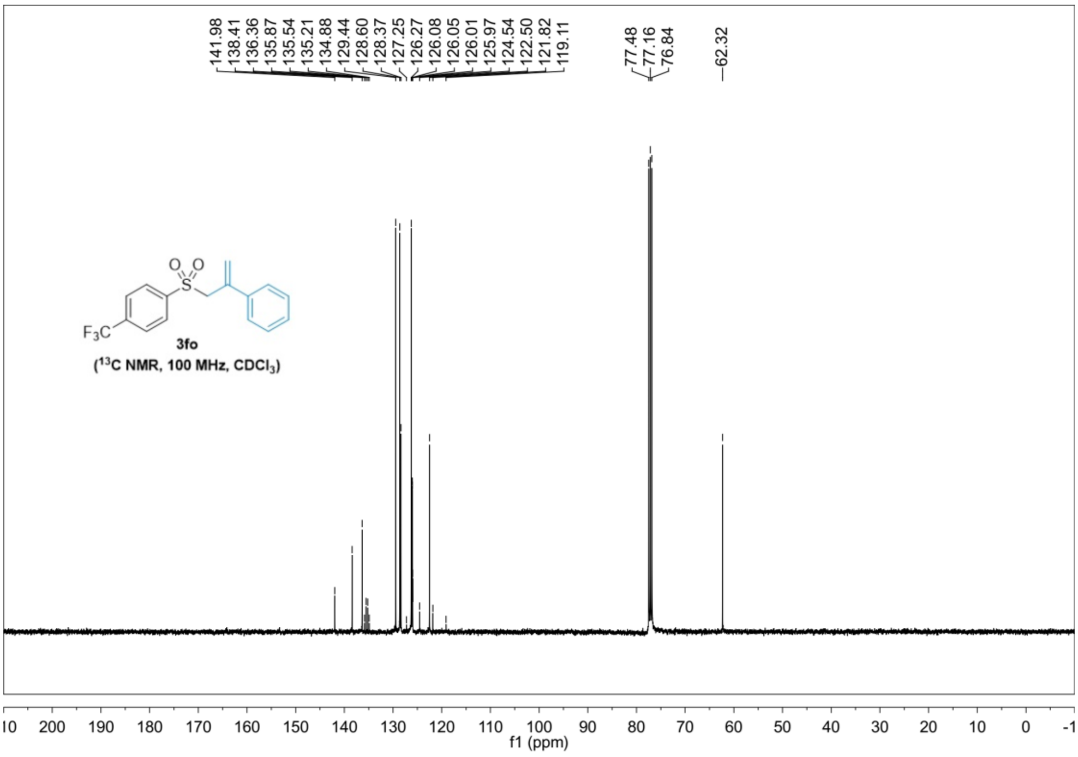
141.98
138.41
136.36
135.87
135.54
135.21
134.88
129.44
128.60
128.37
127.25
126.27
126.08
126.05
126.01
125.97
124.54
122.50
121.82
119.11
77.48
77.16
76.84
62.32
F3C
3fo
(13C NMR, 100 MHz, CDCl3)
200 190 180 170 160 150 140 130 120 110 100 90 80 70 60 50 40 30 20 10 0
f1 (ppm)

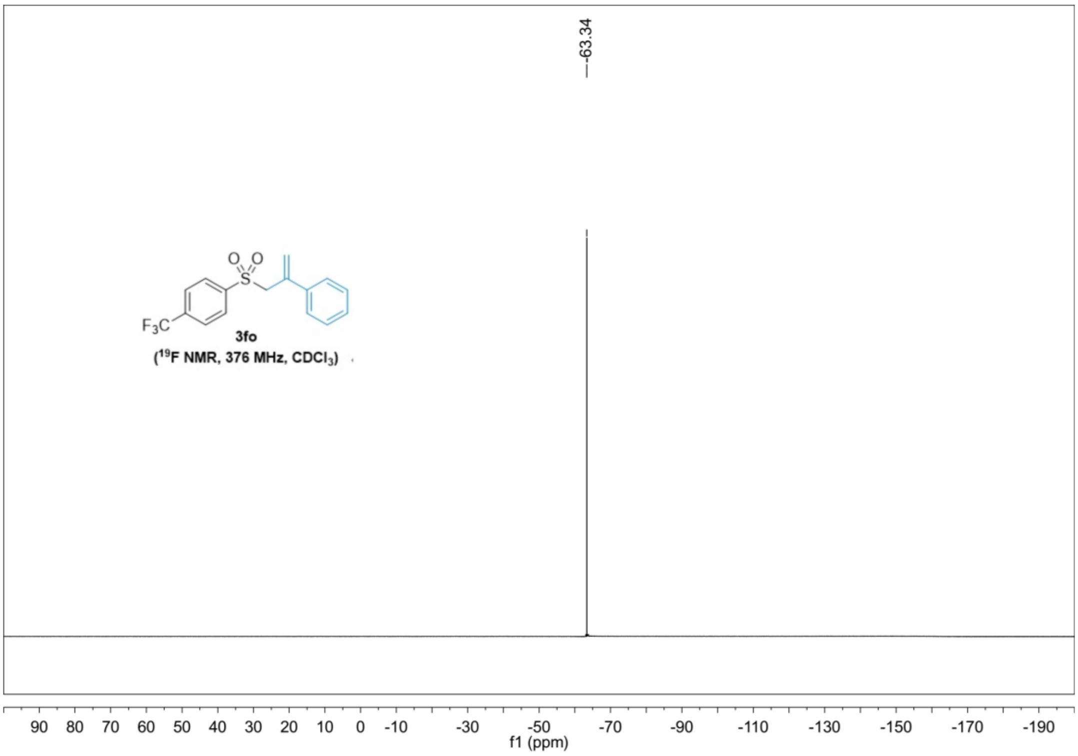

3fo
(19F NMR, 376 MHz, CDCl3)
-63.34
f1 (ppm)

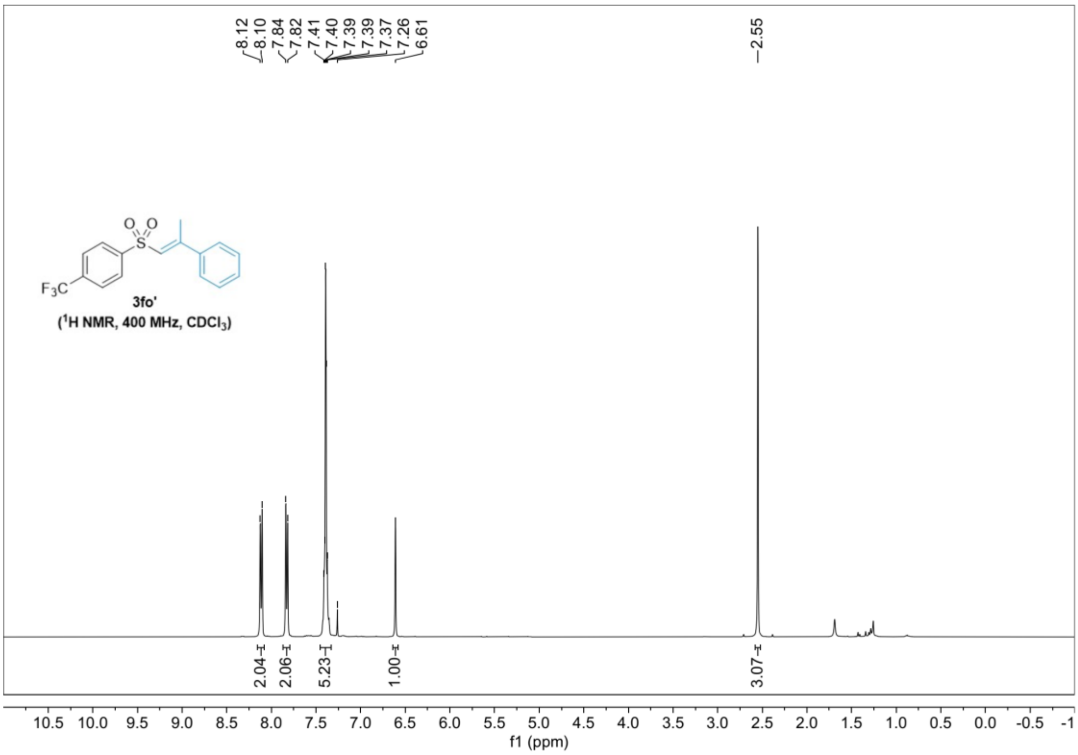

3fo'
(1H NMR, 400 MHz, CDCl3)
8.12
8.10
7.84
7.82
7.41
7.40
7.39
7.39
7.37
7.26
6.61
2.55
2.04
2.06
5.23
1.00
3.07
f1 (ppm)

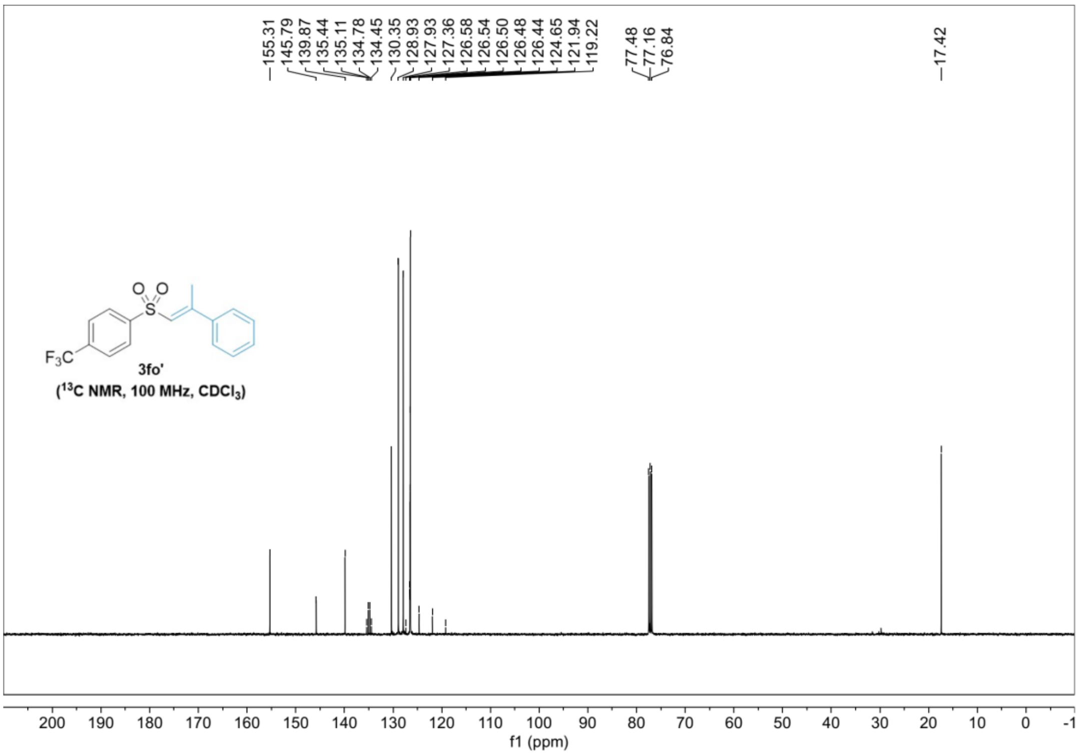
155.31
145.79
139.87
135.44
135.11
134.78
134.45
130.35
128.93
127.93
127.36
126.58
126.54
126.50
126.48
126.44
124.65
121.94
119.22
77.48
77.16
76.84
17.42
F3C
3fo'
(13C NMR, 100 MHz, CDCl3)
200 190 180 170 160 150 140 130 120 110 100 90 80 70 60 50 40 30 20 10 0
f1 (ppm)

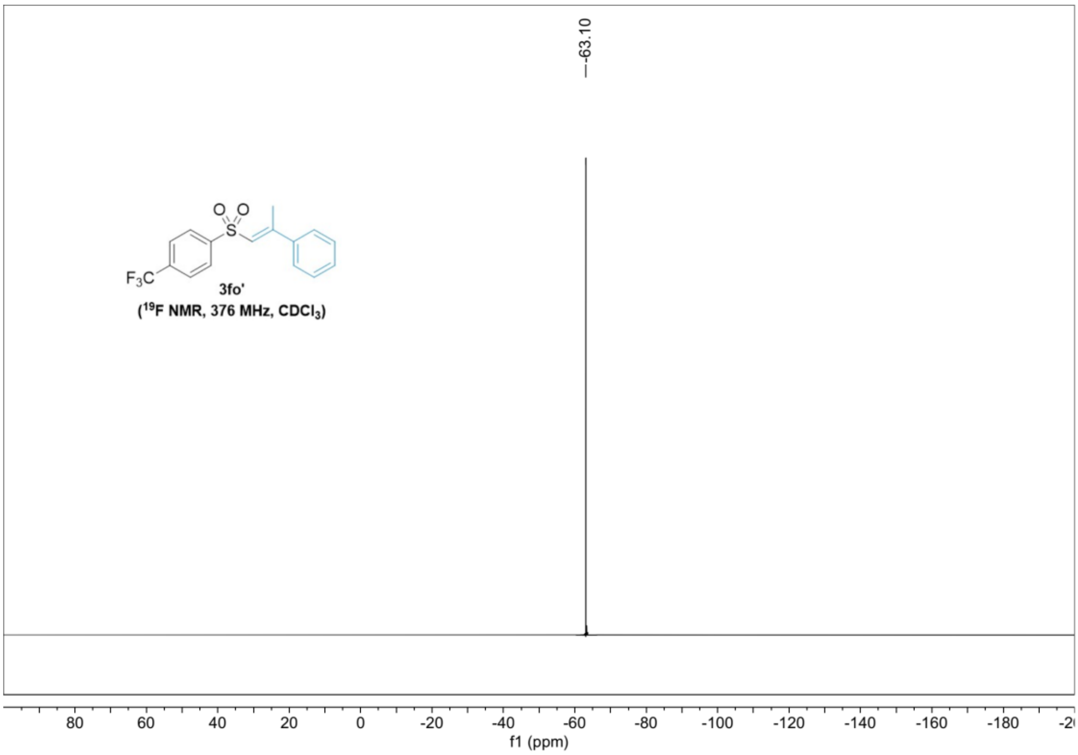
-63.10
F3C
3fo'
(19F NMR, 376 MHz, CDCl3)
80 60 40 20 0 -20 -40 -60 -80 -100 -120 -140 -160 -180
f1 (ppm)

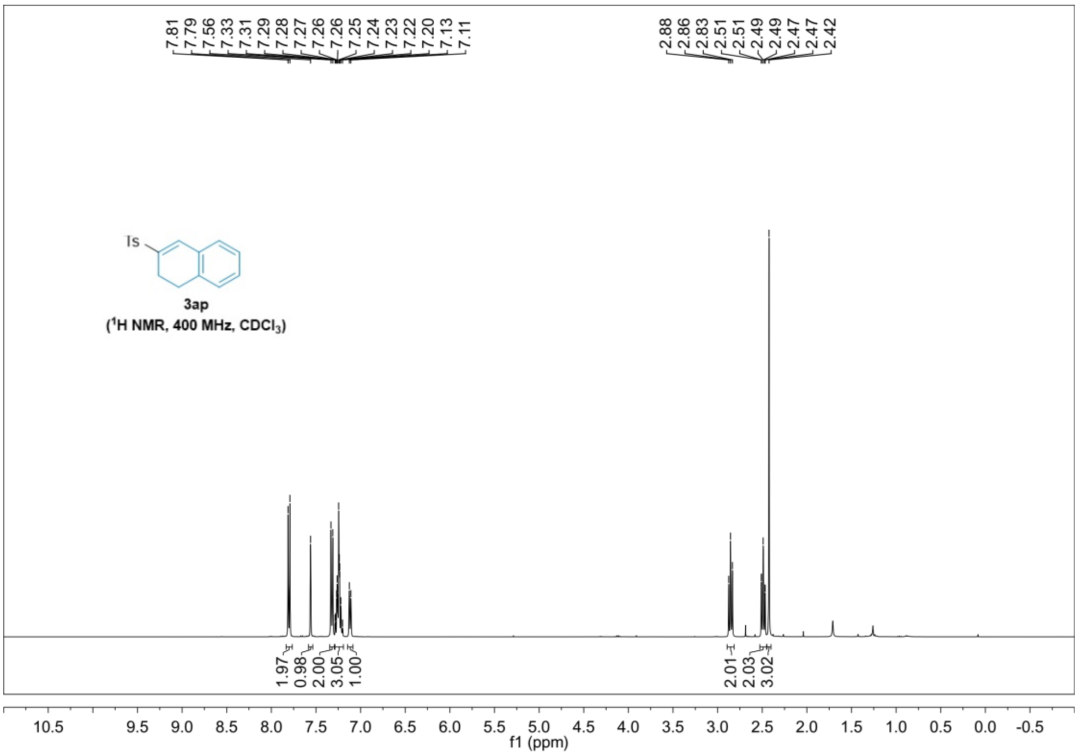
7.81
7.79
7.56
7.33
7.31
7.29
7.28
7.27
7.26
7.26
7.25
7.24
7.23
7.22
7.20
7.13
7.11
2.88
2.86
2.83
2.51
2.51
2.49
2.47
2.47
2.42
Ts
3ap
(¹H NMR, 400 MHz, CDCl3)
1.97
0.98
2.00
3.05
1.00
2.01
2.03
3.02
10.5
9.5
9.0
8.5
8.0
7.5
7.0
6.5
6.0
5.5
5.0
4.5
4.0
3.5
3.0
2.5
2.0
1.5
1.0
0.5
0.0
-0.5
f1 (ppm)

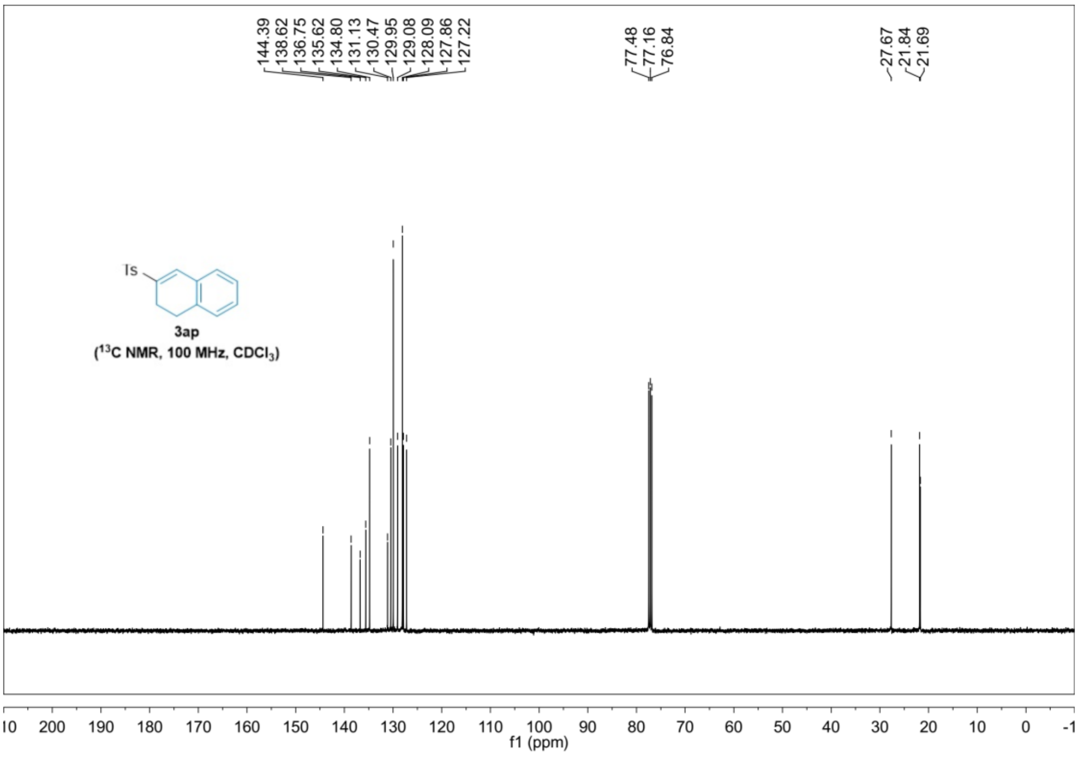
144.39
138.62
136.75
135.62
134.80
131.13
130.47
129.95
129.08
128.09
127.86
127.22
77.48
77.16
76.84
27.67
21.84
21.69
Ts
3ap
(¹³C NMR, 100 MHz, CDCl3)
200
190
180
170
160
150
140
130
120
110
100
90
80
70
60
50
40
30
20
10
0
f1 (ppm)

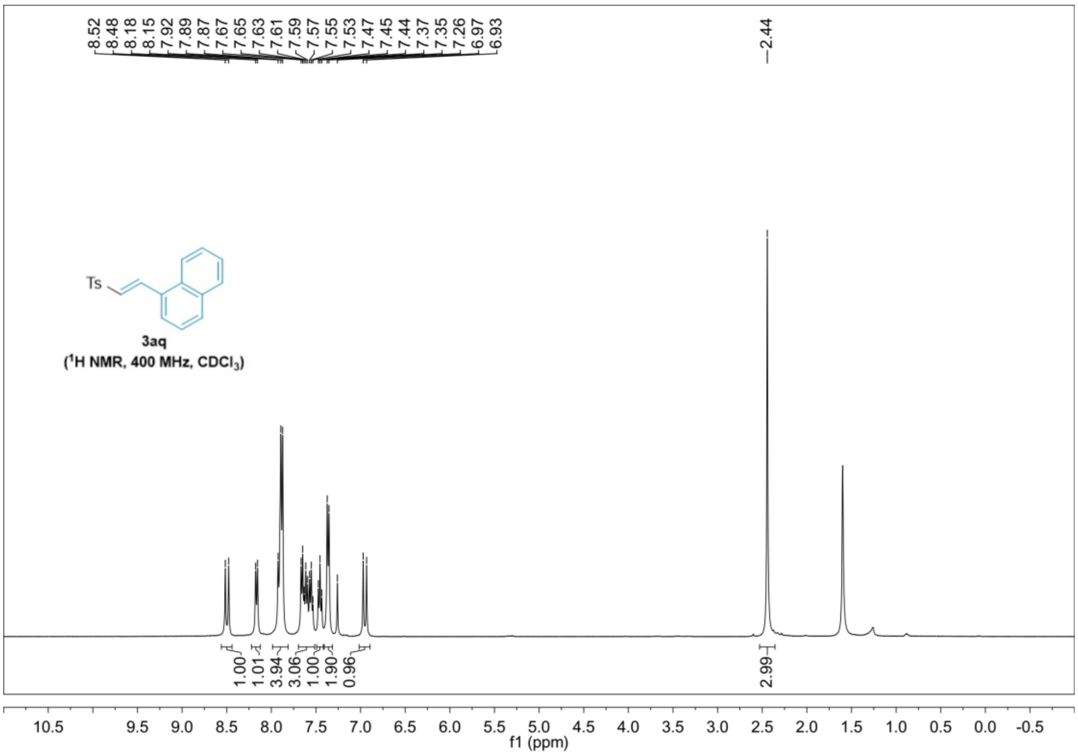
8.52
8.48
8.18
8.15
7.92
7.89
7.87
7.67
7.65
7.63
7.61
7.59
7.57
7.55
7.53
7.47
7.45
7.44
7.37
7.35
7.26
6.97
6.93
2.44
Ts
3aq
(^{1}H NMR, 400 MHz, CDCl$_3$)
1.00
1.01
3.94
3.06
1.00
1.90
0.96
2.99
10.5 9.5 9.0 8.5 8.0 7.5 7.0 6.5 6.0 5.5 5.0 4.5 4.0 3.5 3.0 2.5 2.0 1.5 1.0 0.5 0.0 -0.5
f1 (ppm)

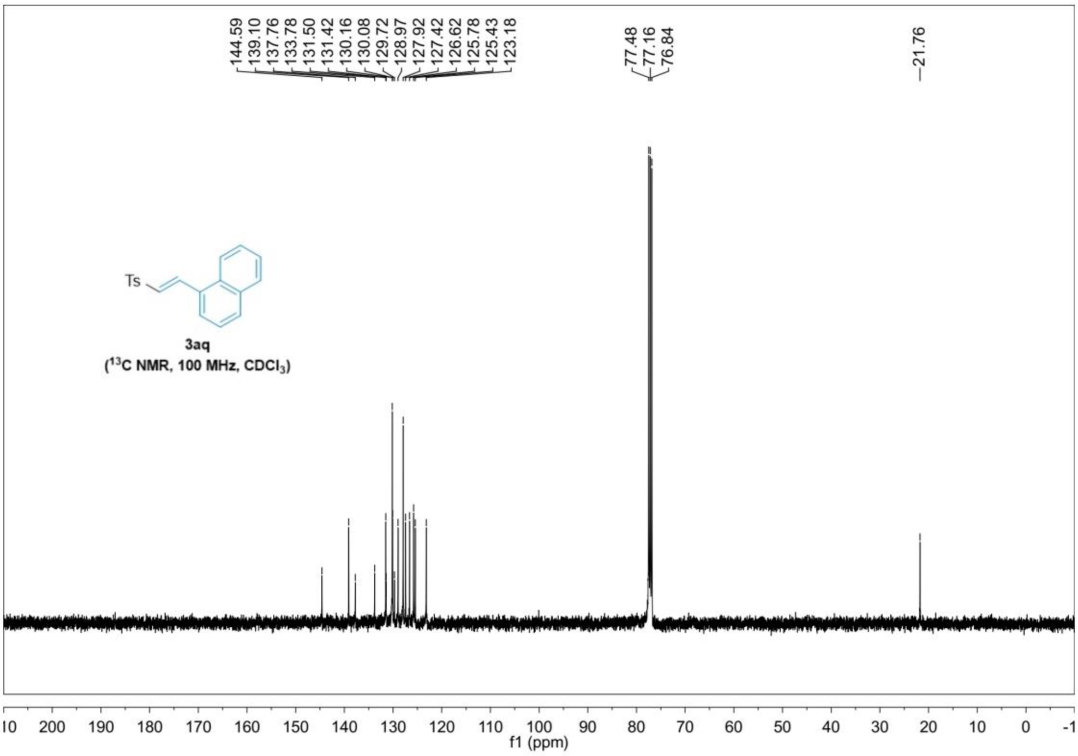
144.59
139.10
137.76
133.78
131.50
131.42
130.16
130.08
129.72
128.97
127.92
127.42
126.62
125.78
125.43
123.18
77.48
77.16
76.84
21.76
Ts
3aq
(^{13}C NMR, 100 MHz, CDCl$_3$)
10 200 190 180 170 160 150 140 130 120 110 100 90 80 70 60 50 40 30 20 10 0 -1
f1 (ppm)

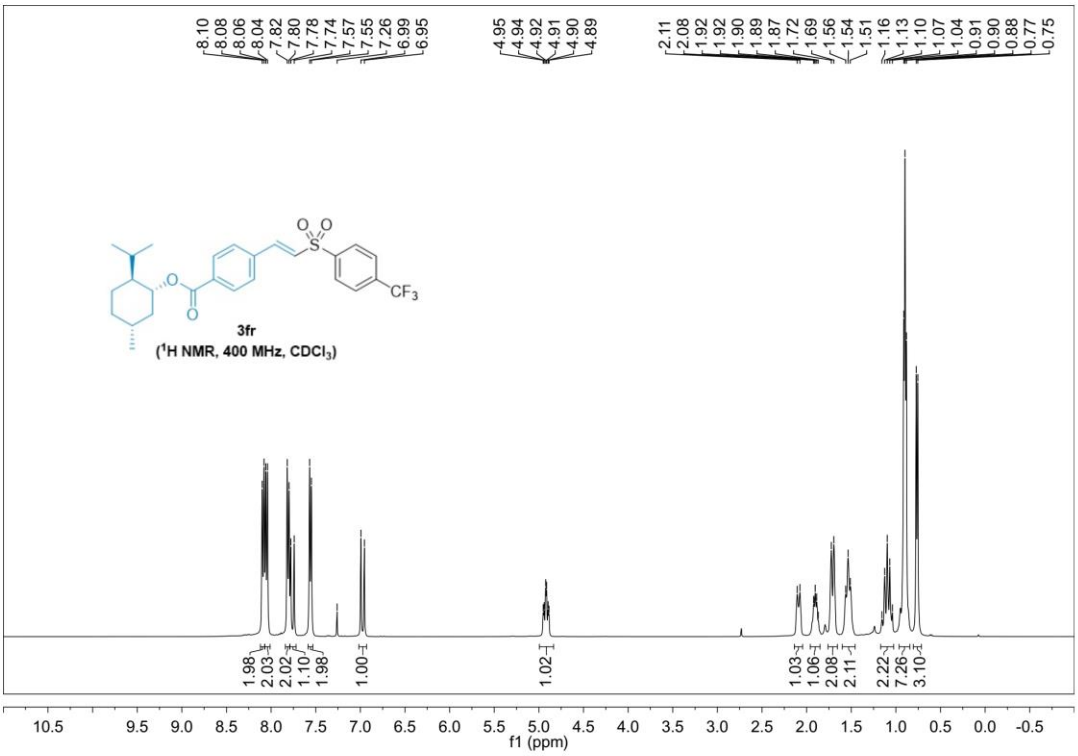
8.10 8.08 8.06 8.04 7.82 7.80 7.78 7.74 7.57 7.55 7.26 6.99 6.95
4.95 4.94 4.92 4.91 4.90 4.89
2.11 2.08 1.92 1.90 1.89 1.87 1.72 1.69 1.56 1.54 1.51 1.16 1.13 1.10 1.07 1.04 0.91 0.90 0.88 0.77 0.75
CF3
3fr
(1H NMR, 400 MHz, CDCl3)
1.98 2.03 2.02 1.10 1.98 1.00 1.02 1.03 1.06 2.08 2.11 2.22 7.26 3.10
f1 (ppm)

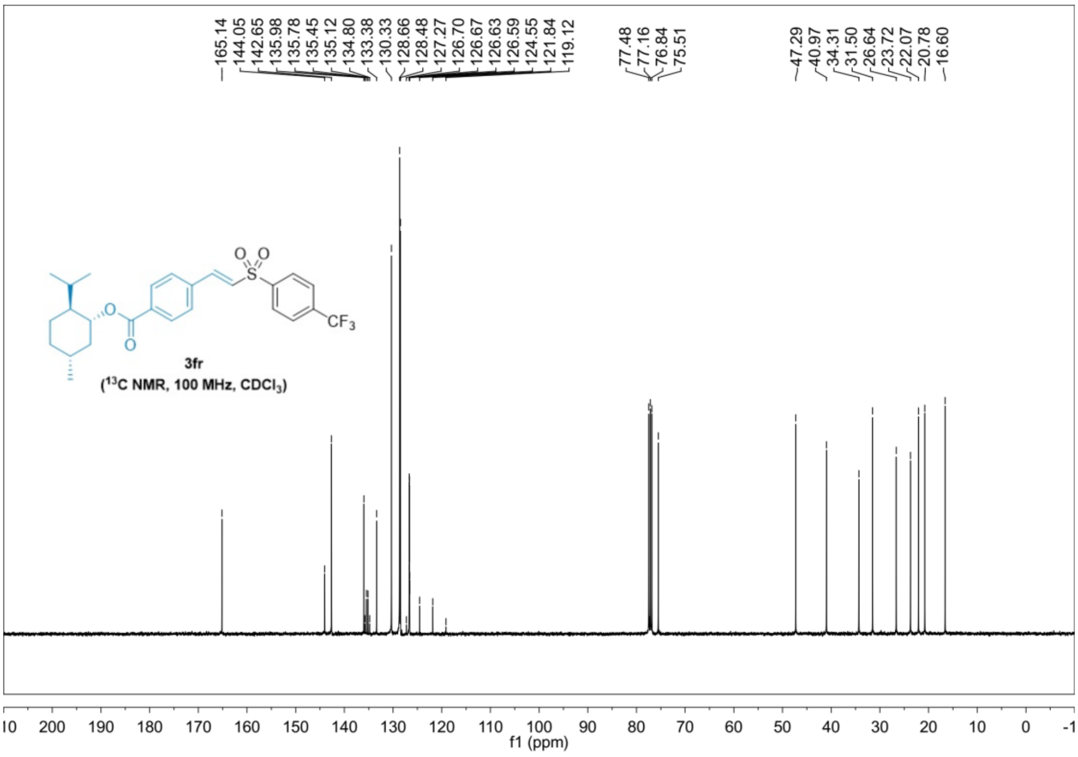
165.14 144.05 142.65 135.98 135.78 135.45 135.12 134.80 133.38 130.33 128.66 128.48 127.27 126.70 126.67 126.63 126.59 124.55 121.84 119.12
77.48 77.16 76.84 75.51
47.29 40.97 34.31 31.50 26.64 23.72 22.07 20.78 16.60
CF3
3fr
(13C NMR, 100 MHz, CDCl3)
f1 (ppm)

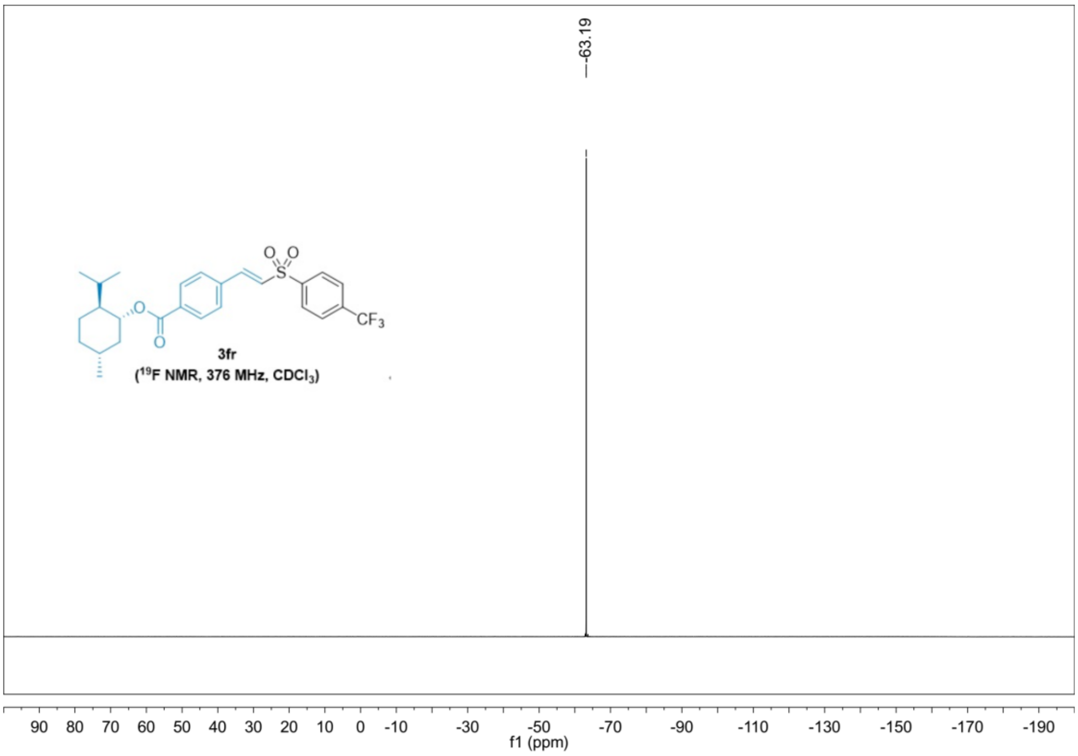

-63.19
3fr
(19F NMR, 376 MHz, CDCl3)
f1 (ppm)

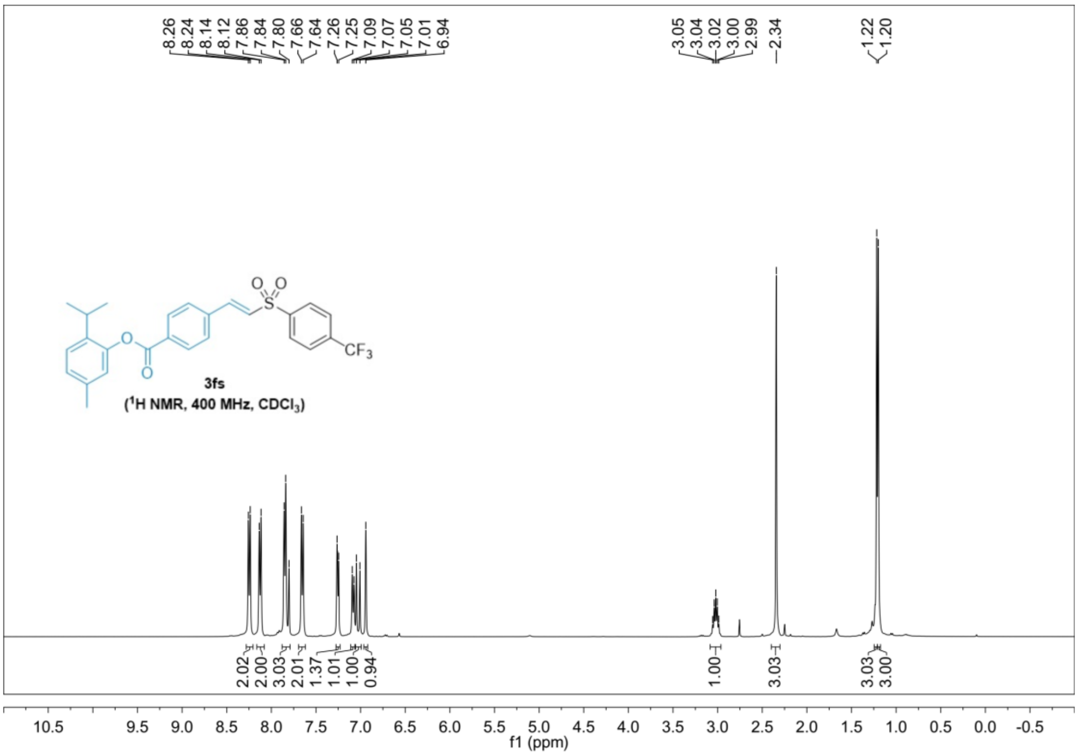

3fs
(1H NMR, 400 MHz, CDCl3)
f1 (ppm)

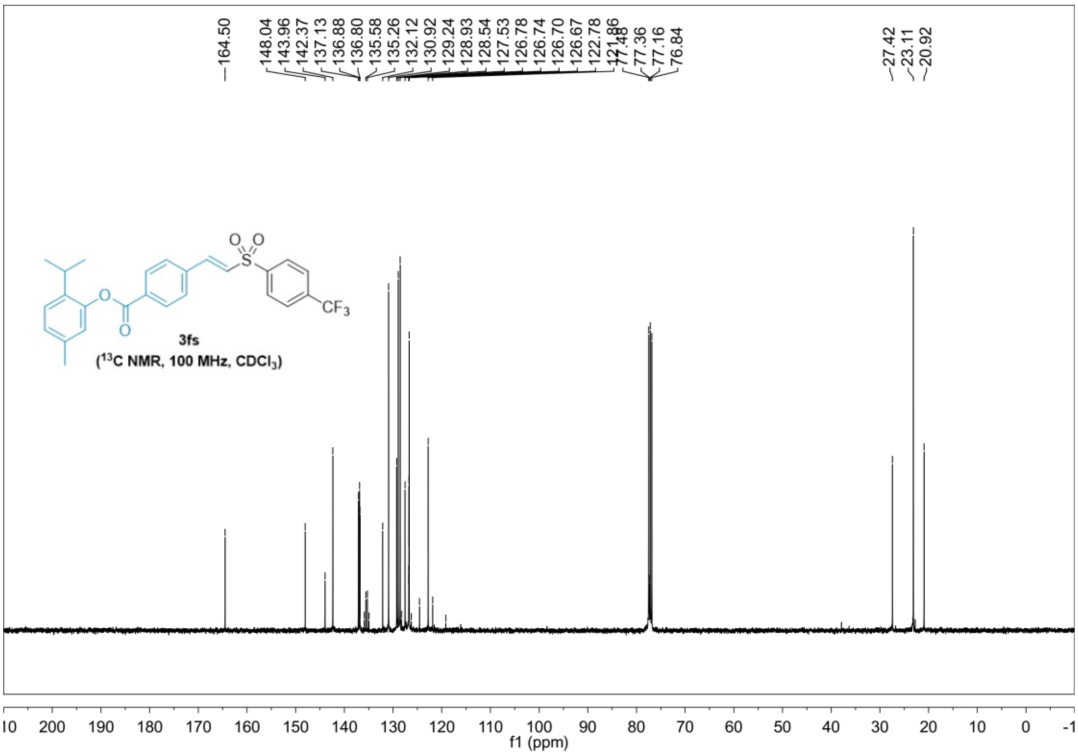
164.50
148.04
143.96
142.37
137.13
136.88
136.80
135.58
135.26
132.12
130.92
129.24
128.93
128.54
127.53
126.78
126.74
126.70
126.67
122.78
121.86
77.48
77.36
77.16
76.84
27.42
23.11
20.92
3fs
(13C NMR, 100 MHz, CDCl3)
f1 (ppm)

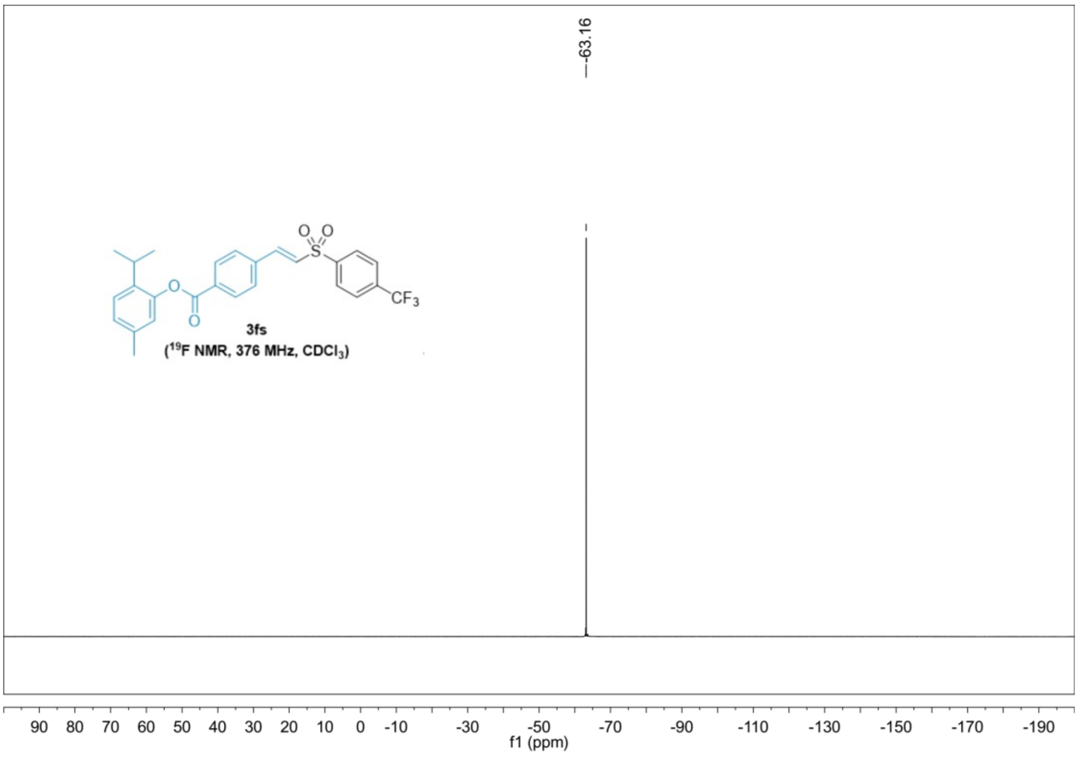
-63.16
3fs
(19F NMR, 376 MHz, CDCl3)
f1 (ppm)

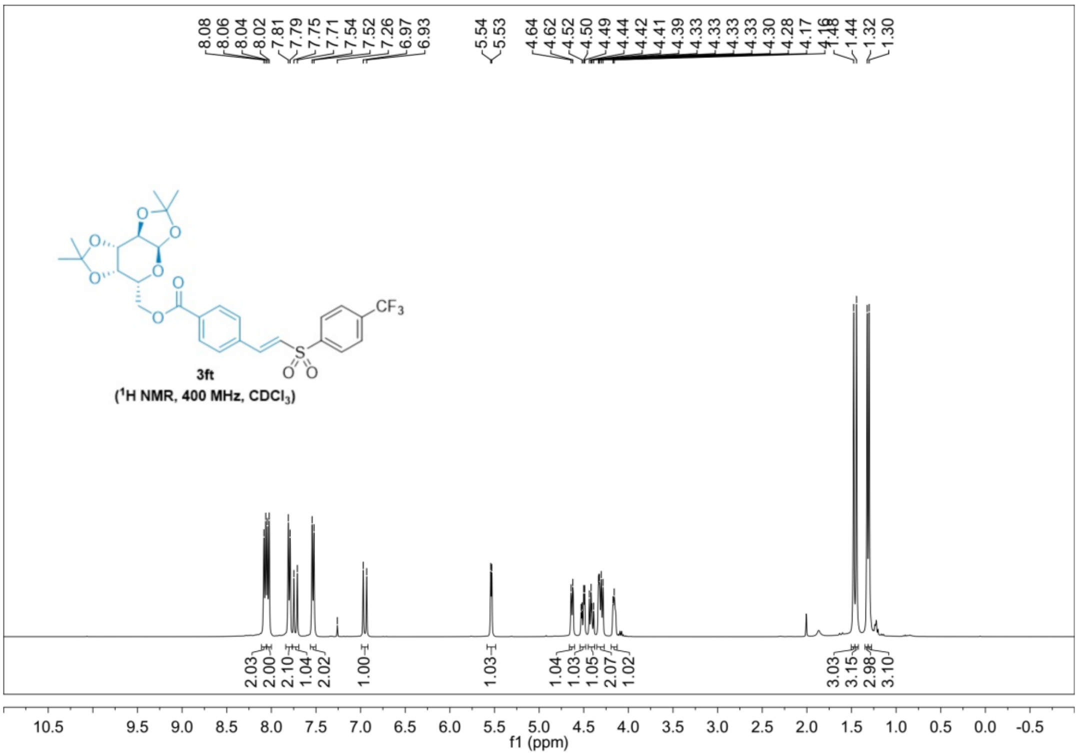
8.08
8.06
8.04
8.02
7.81
7.79
7.75
7.71
7.54
7.52
7.26
6.97
6.93
5.54
5.53
4.64
4.62
4.52
4.50
4.49
4.44
4.42
4.41
4.39
4.33
4.33
4.33
4.33
4.30
4.28
4.17
4.16
1.48
1.44
1.32
1.30
CF3
O
S
3ft
(¹H NMR, 400 MHz, CDCl3)
2.03
2.00
2.10
1.04
2.02
1.00
1.03
1.04
1.03
1.05
2.07
1.02
3.03
3.15
2.98
3.10
f1 (ppm)

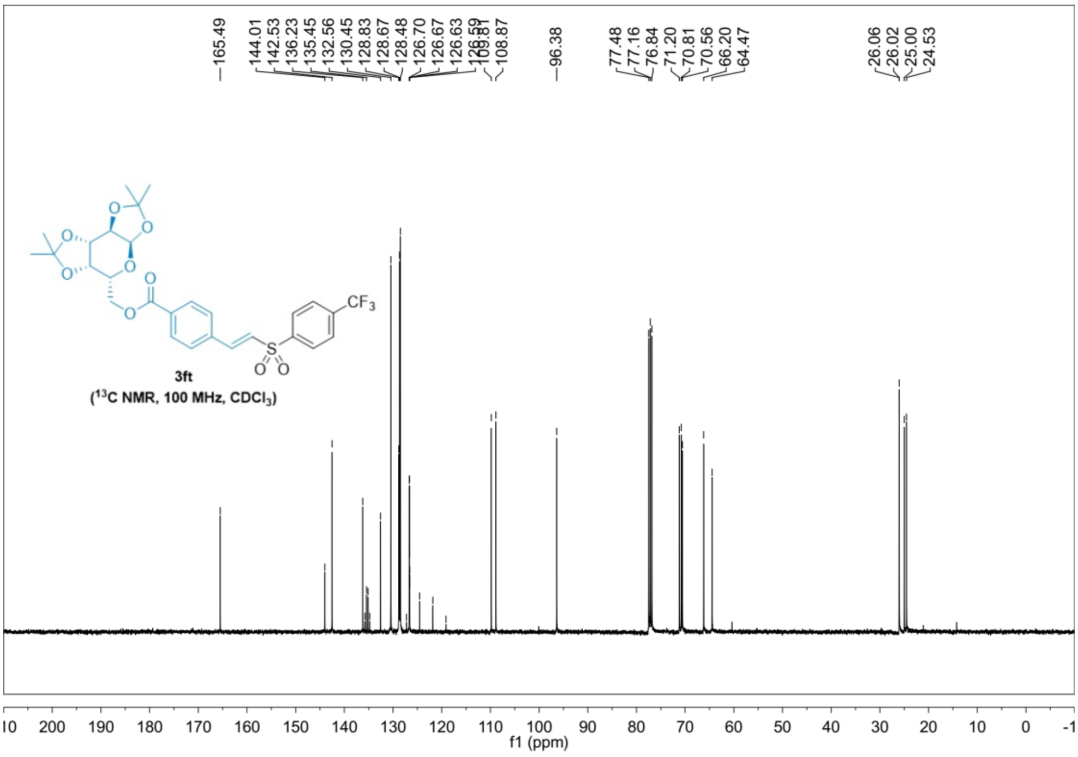
165.49
144.01
142.53
136.23
135.45
132.56
130.45
128.83
128.67
128.48
126.70
126.67
126.63
126.59
109.81
108.87
96.38
77.48
77.16
76.84
71.20
70.81
70.56
66.20
64.47
26.06
26.02
25.00
24.53
CF3
3ft
(¹³C NMR, 100 MHz, CDCl3)
f1 (ppm)

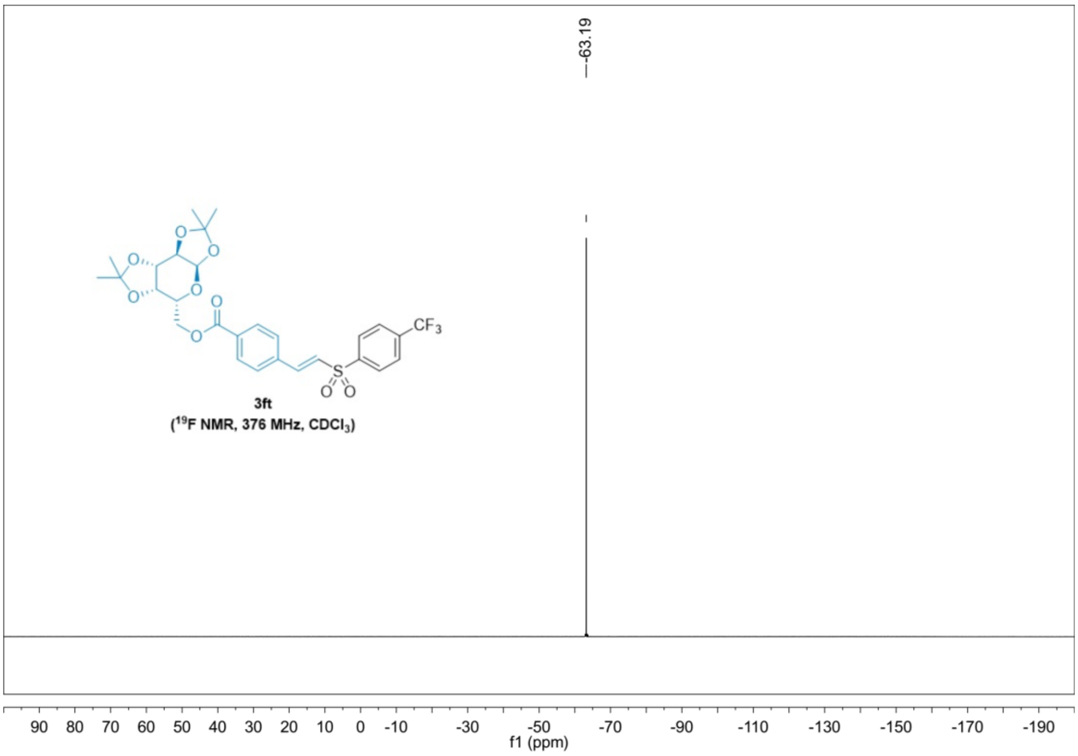
-63.19
CF3
3ft
(19F NMR, 376 MHz, CDCl3)
90 80 70 60 50 40 30 20 10 0 -10 -30 -50 -70 -90 -110 -130 -150 -170 -190
f1 (ppm)

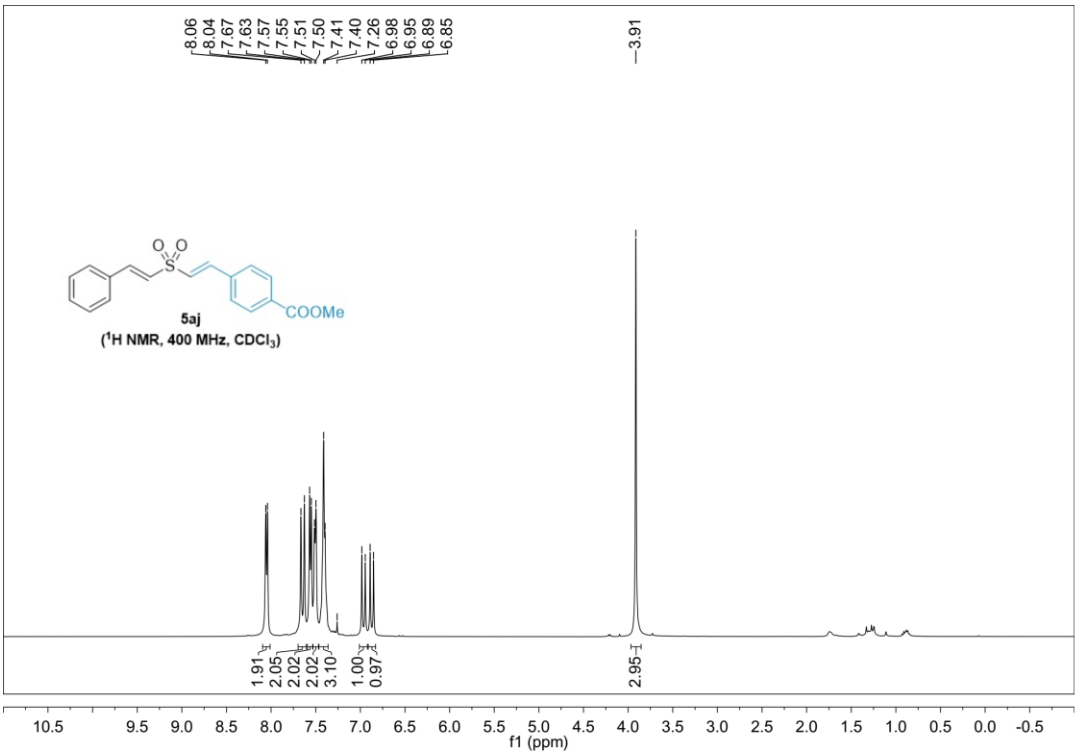
8.06 8.04 7.67 7.63 7.57 7.55 7.51 7.50 7.41 7.40 7.26 6.98 6.95 6.89 6.85
3.91
COOMe
5aj
(1H NMR, 400 MHz, CDCl3)
1.91 2.05 2.02 2.02 3.10 1.00 0.97
2.95
10.5 9.5 9.0 8.5 8.0 7.5 7.0 6.5 6.0 5.5 5.0 4.5 4.0 3.5 3.0 2.5 2.0 1.5 1.0 0.5 0.0 -0.5
f1 (ppm)

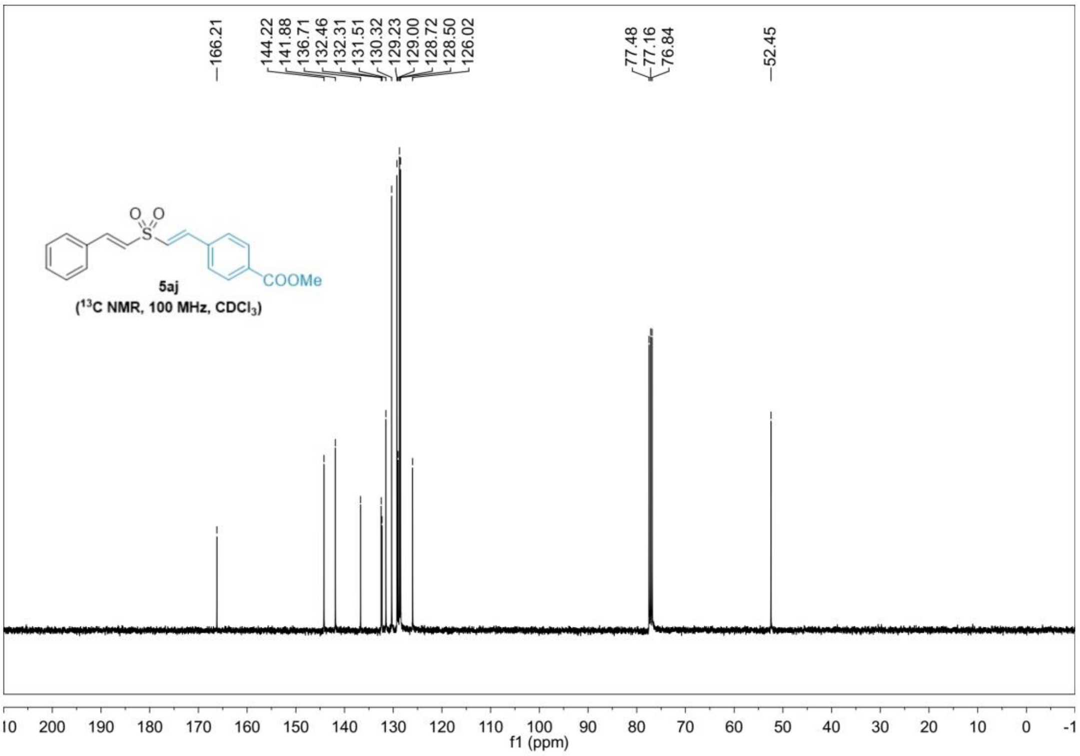

166.21
144.22
141.88
136.71
132.46
132.31
131.51
130.32
129.23
129.00
128.72
128.50
126.02
77.48
77.16
76.84
52.45
COOMe
5aj
(^{13}C NMR, 100 MHz, CDCl$_3$)
10 200 190 180 170 160 150 140 130 120 110 100 90 80 70 60 50 40 30 20 10 0 -1
f1 (ppm)

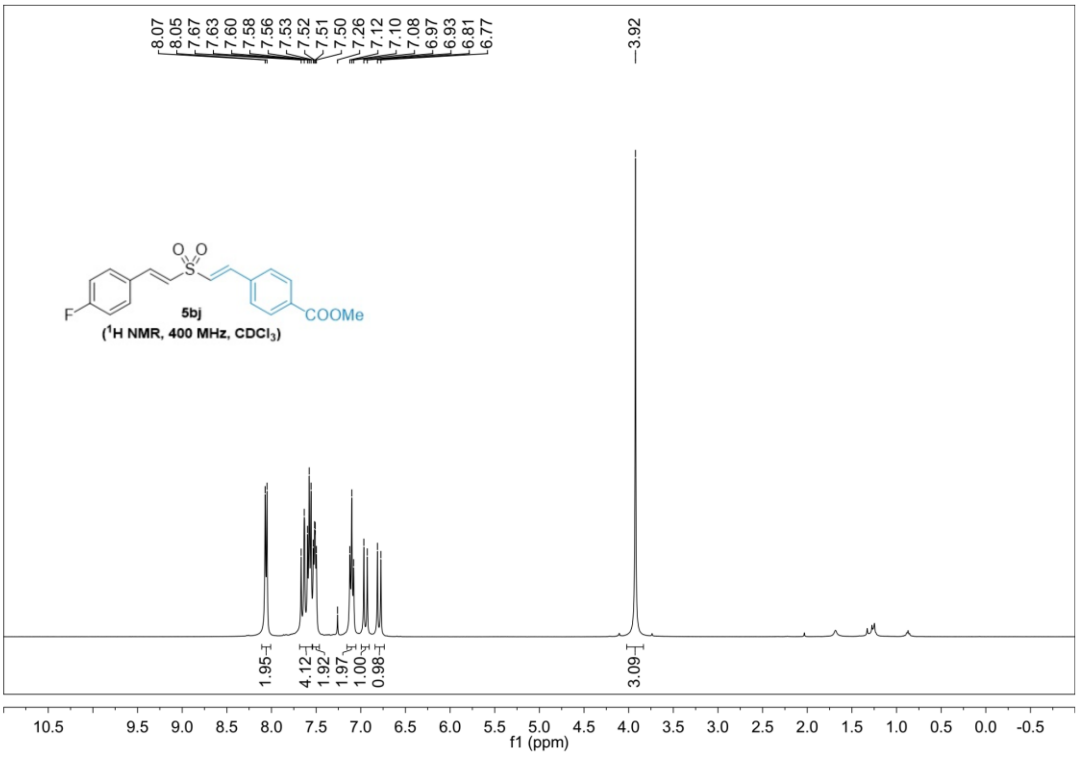

8.07
8.05
7.67
7.63
7.60
7.58
7.56
7.53
7.52
7.51
7.50
7.26
7.12
7.10
7.08
6.97
6.93
6.81
6.77
3.92
F
COOMe
5bj
(^{1}H NMR, 400 MHz, CDCl$_3$)
1.95
4.12
1.92
1.97
1.00
0.98
3.09
10.5 9.5 9.0 8.5 8.0 7.5 7.0 6.5 6.0 5.5 5.0 4.5 4.0 3.5 3.0 2.5 2.0 1.5 1.0 0.5 0.0 -0.5
f1 (ppm)

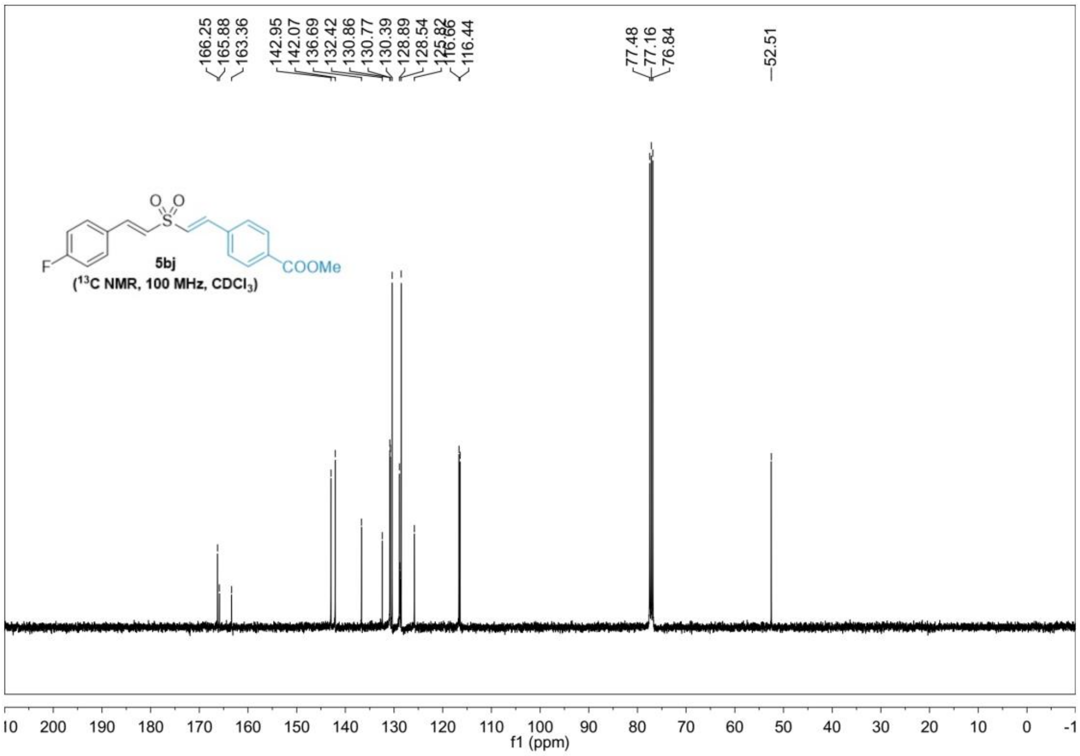
166.25
165.88
163.36
142.95
142.07
136.69
132.42
130.86
130.77
130.39
128.89
128.54
125.82
116.66
116.44
77.48
77.16
76.84
52.51
F
COOMe
5bj
(^{13}C NMR, 100 MHz, CDCl$_3$)
f1 (ppm)

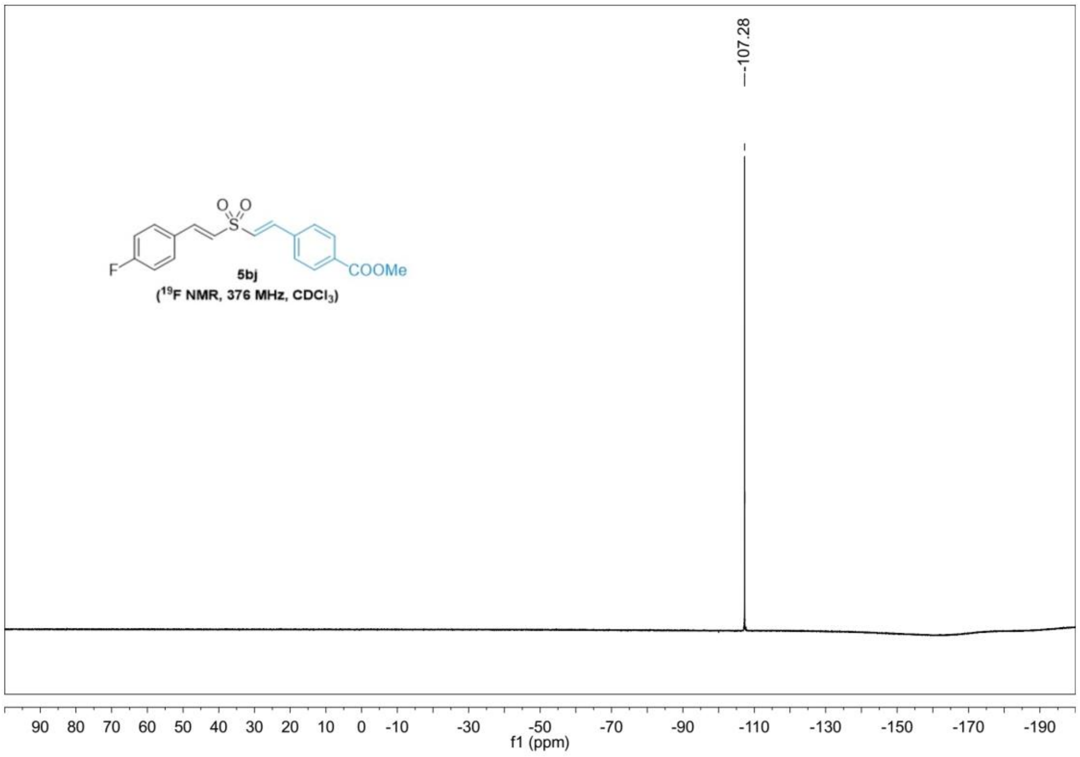
-107.28
F
COOMe
5bj
(^{19}F NMR, 376 MHz, CDCl$_3$)
f1 (ppm)

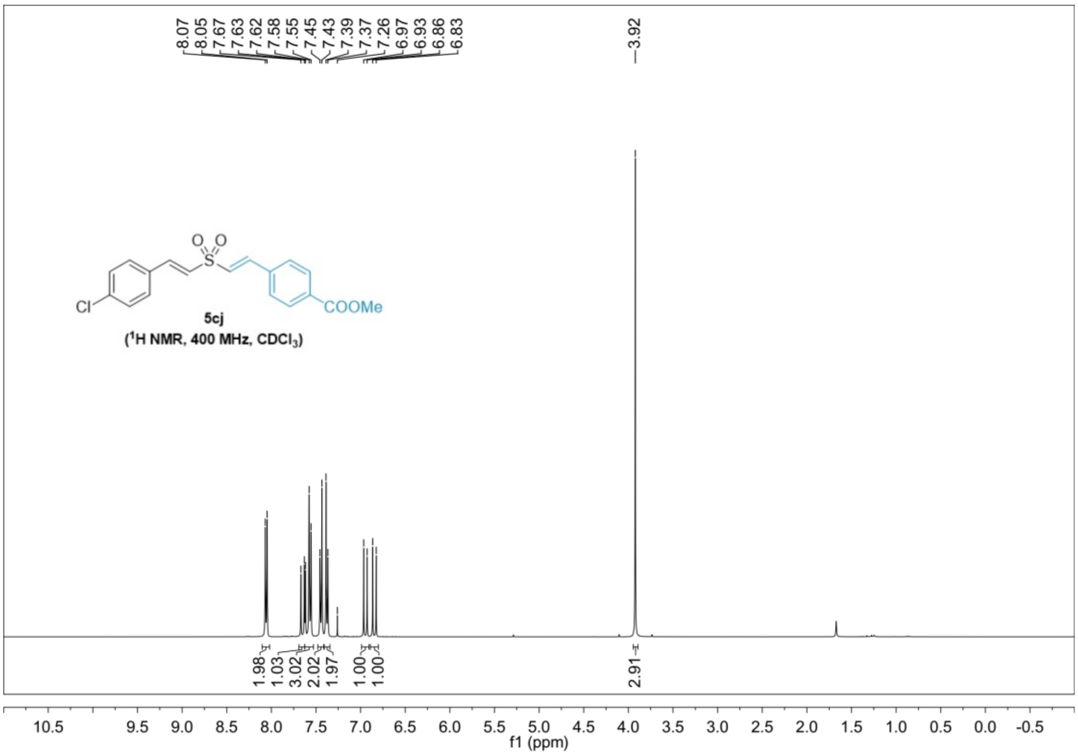
8.07
8.05
7.67
7.63
7.62
7.58
7.55
7.45
7.43
7.39
7.37
7.26
6.97
6.93
6.86
6.83
3.92
Cl
COOMe
5cj
(^{1}H NMR, 400 MHz, CDCl$_3$)
1.98
1.03
3.02
2.02
1.97
1.00
1.00
2.91
f1 (ppm)

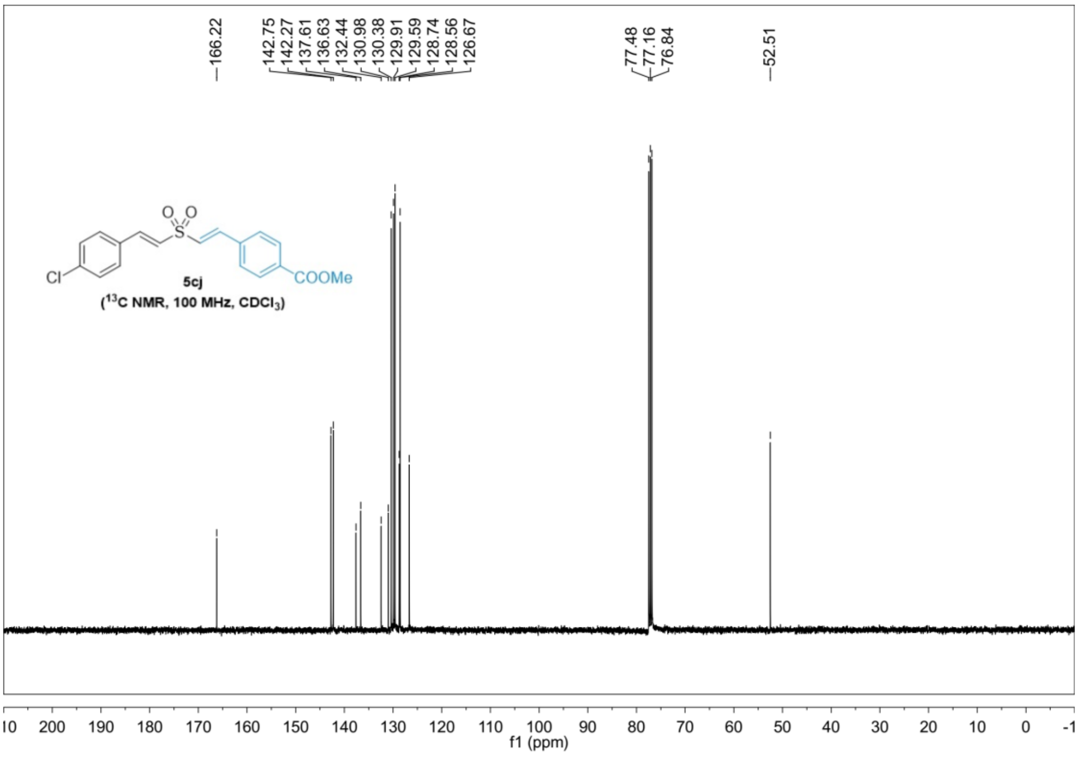
166.22
142.75
142.27
137.61
136.63
132.44
130.98
130.38
129.91
129.59
128.74
128.56
126.67
77.48
77.16
76.84
52.51
Cl
COOMe
5cj
(^{13}C NMR, 100 MHz, CDCl$_3$)
f1 (ppm)

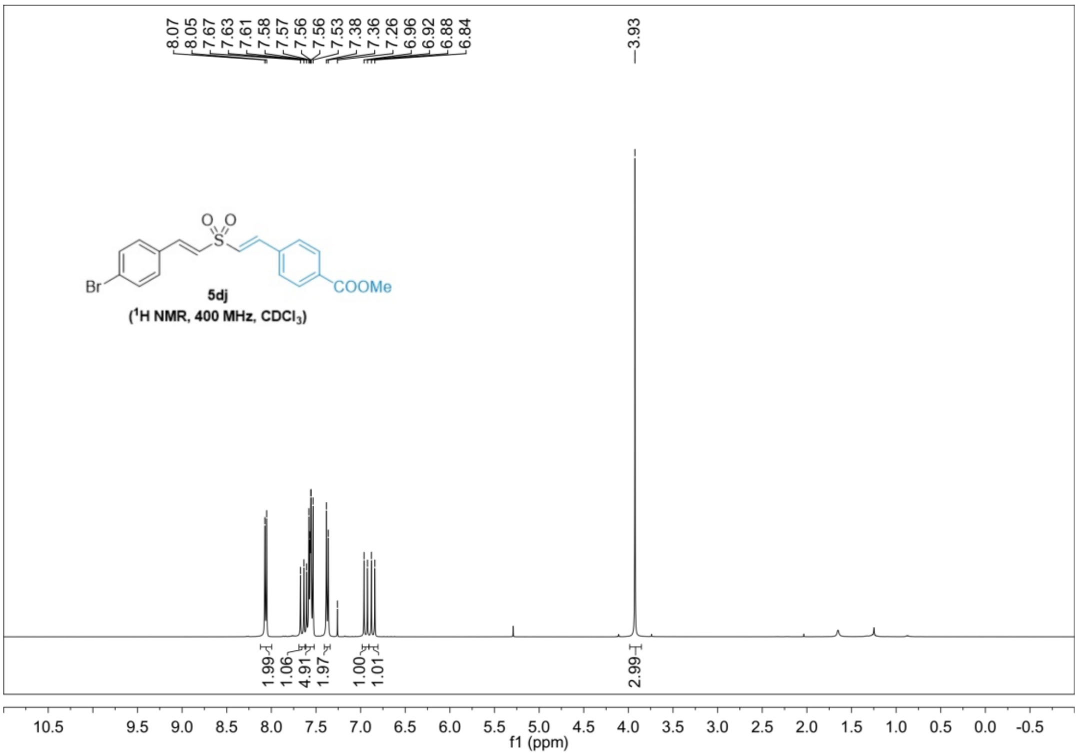
8.07
8.05
7.67
7.63
7.61
7.58
7.57
7.56
7.56
7.53
7.38
7.36
7.26
6.96
6.92
6.88
6.84
3.93
Br
COOMe
5dj
(1H NMR, 400 MHz, CDCl3)
1.99
1.06
4.91
1.97
1.00
1.01
2.99
f1 (ppm)

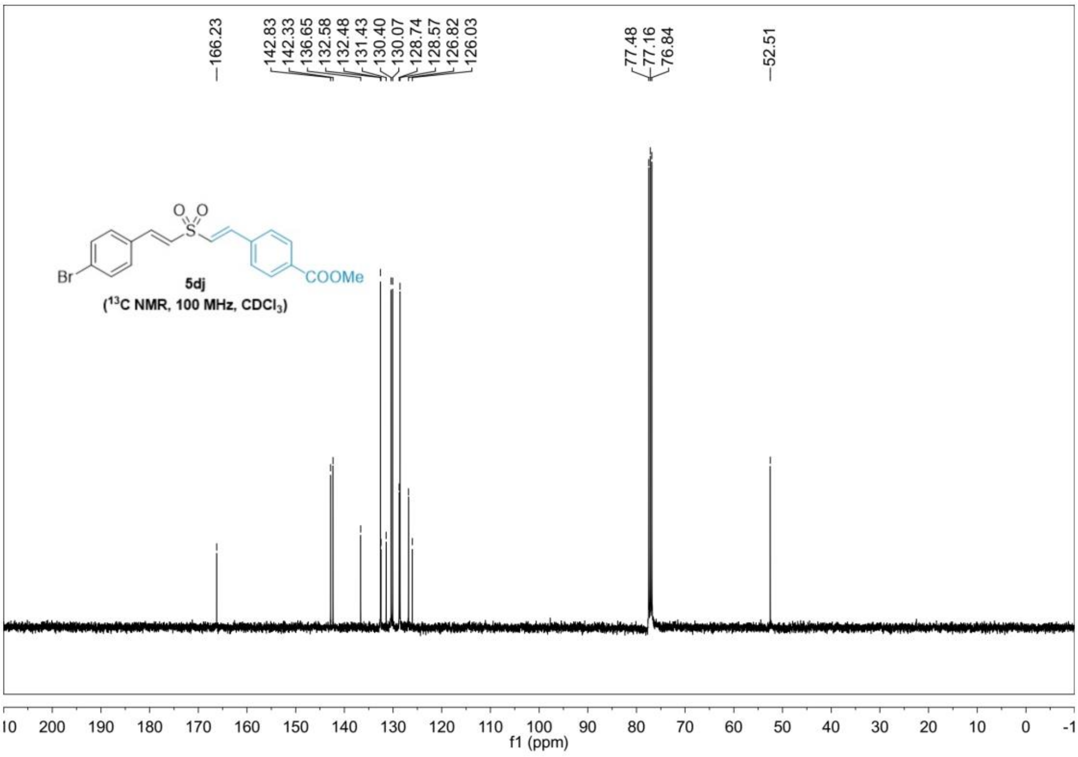
166.23
142.83
142.33
136.65
132.58
132.48
131.43
130.40
130.07
128.74
128.57
126.82
126.03
77.48
77.16
76.84
52.51
Br
COOMe
5dj
(13C NMR, 100 MHz, CDCl3)
f1 (ppm)

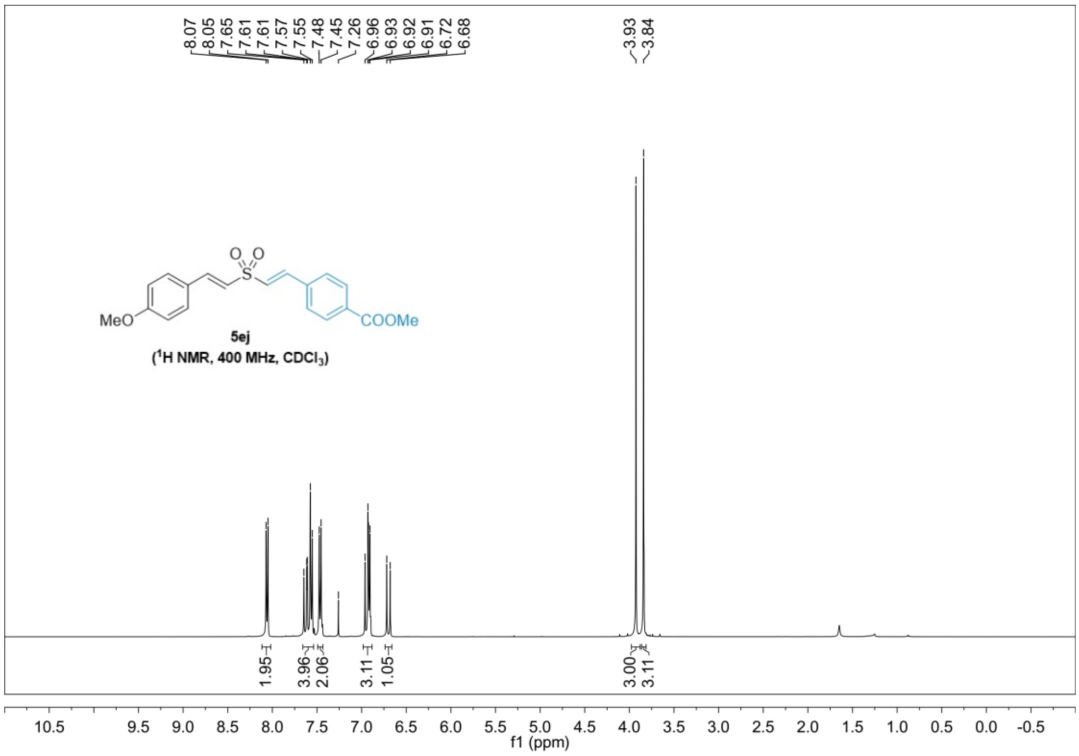
8.07
8.05
7.65
7.61
7.61
7.57
7.55
7.48
7.45
7.26
6.96
6.93
6.92
6.91
6.72
6.68
3.93
3.84
MeO
COOMe
5ej
(1H NMR, 400 MHz, CDCl3)
1.95
3.96
2.06
3.11
1.05
3.00
3.11
f1 (ppm)

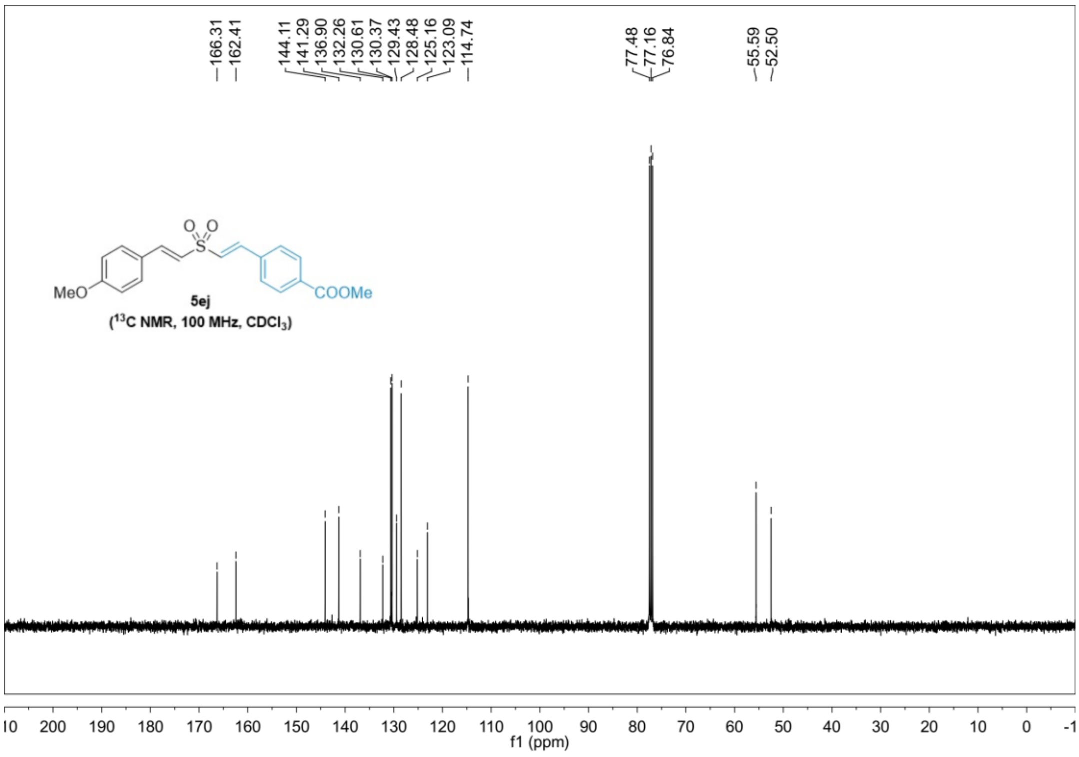
166.31
162.41
144.11
141.29
136.90
132.26
130.61
130.37
129.43
128.48
125.16
123.09
114.74
77.48
77.16
76.84
55.59
52.50
MeO
COOMe
5ej
(13C NMR, 100 MHz, CDCl3)
f1 (ppm)

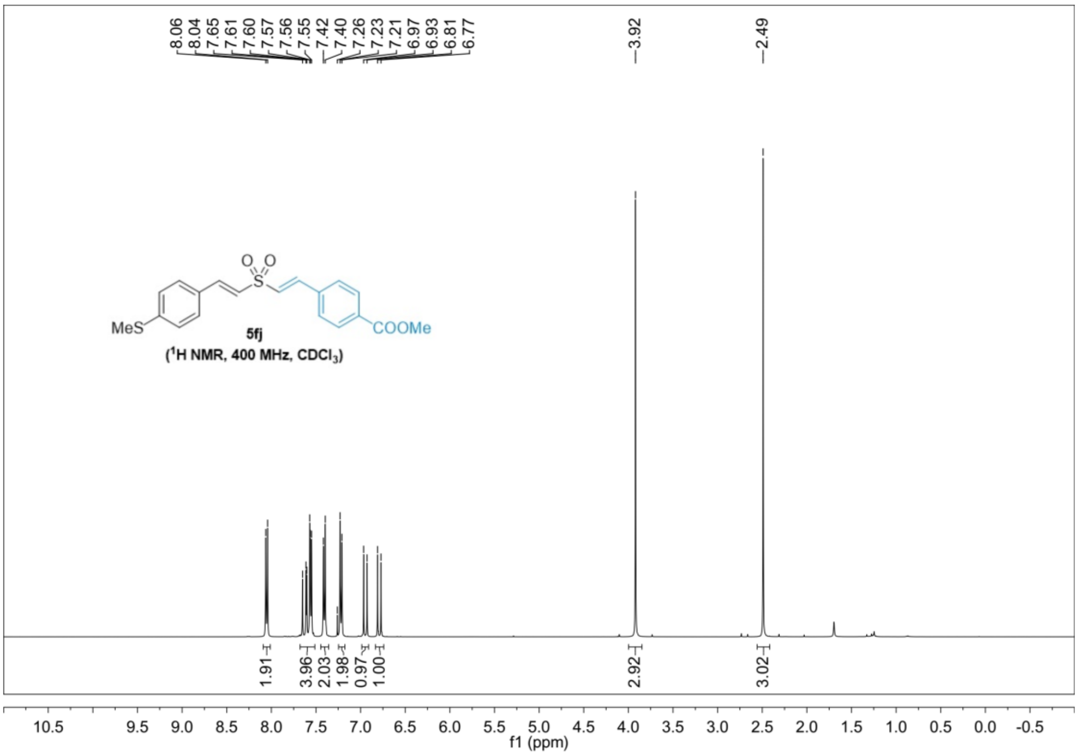

8.06
8.04
7.65
7.61
7.60
7.57
7.56
7.55
7.42
7.40
7.26
7.23
7.21
6.97
6.93
6.81
6.77
3.92
2.49
MeS
COOMe
5fj
(^{1}H NMR, 400 MHz, CDCl$_3$)
1.91
3.96
2.03
1.98
0.97
1.00
2.92
3.02
f1 (ppm)

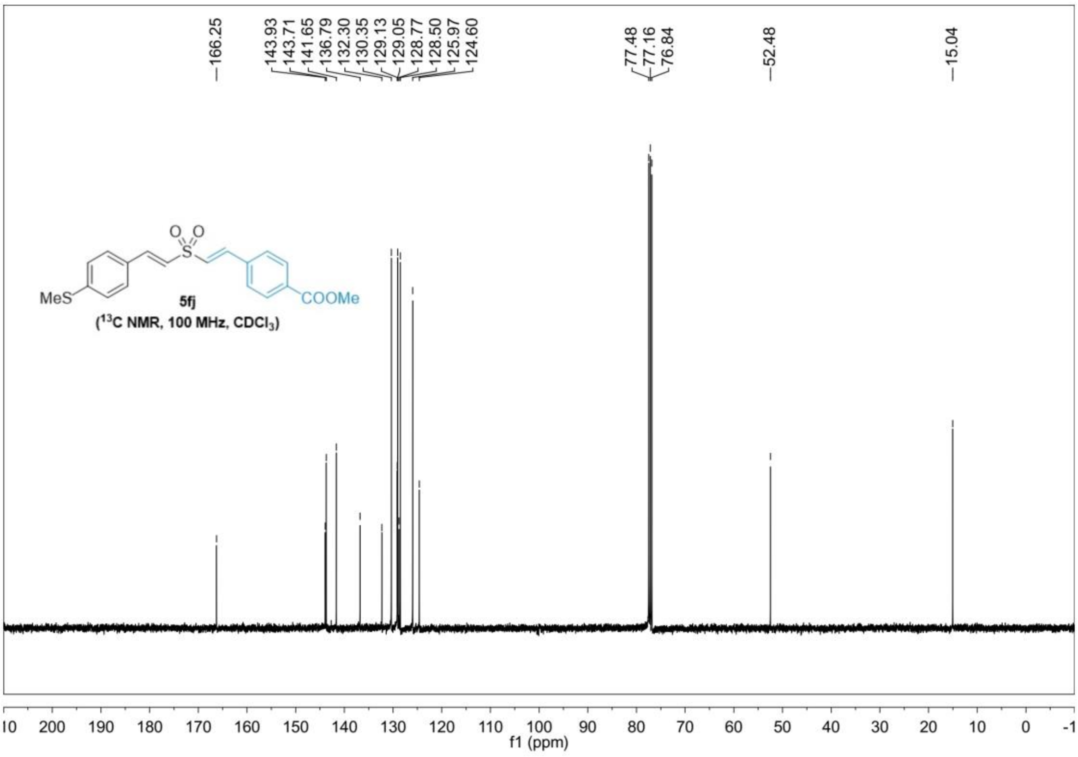

166.25
143.93
143.71
141.65
136.79
132.30
130.35
129.13
129.05
128.77
128.50
125.97
124.60
77.48
77.16
76.84
52.48
15.04
MeS
COOMe
5fj
(^{13}C NMR, 100 MHz, CDCl$_3$)
f1 (ppm)

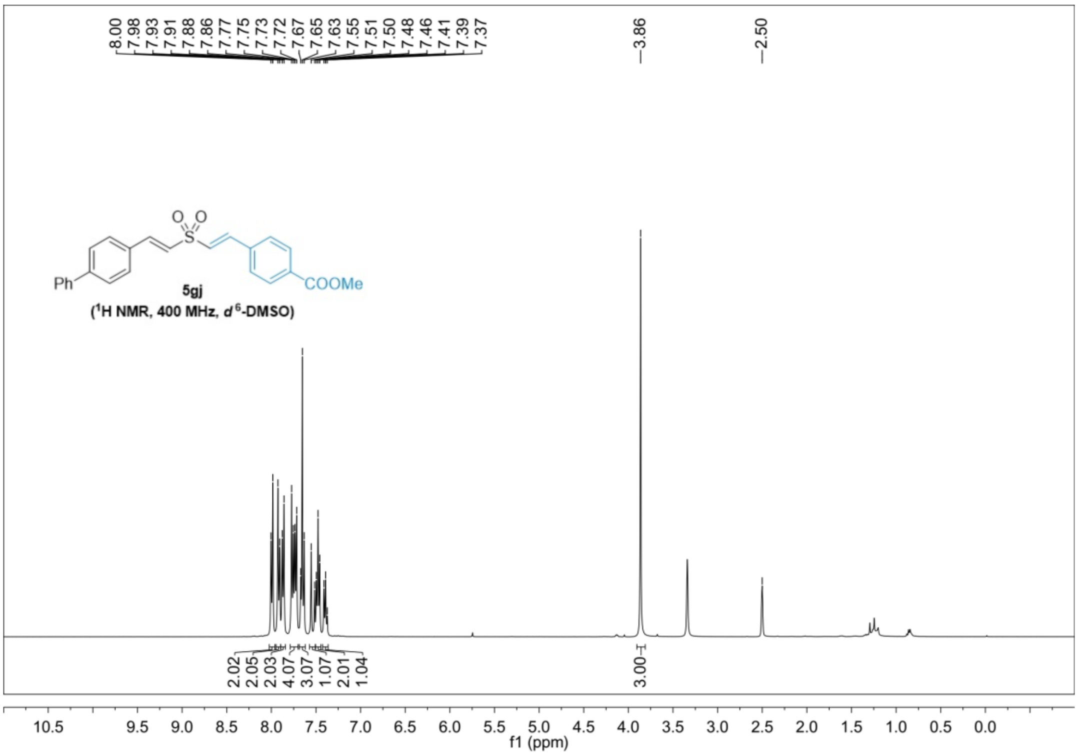
8.00 7.98 7.93 7.91 7.88 7.86 7.77 7.75 7.73 7.72 7.67 7.65 7.63 7.55 7.51 7.50 7.48 7.46 7.41 7.39 7.37
3.86
2.50
Ph
COOMe
5gj
(1H NMR, 400 MHz, d6-DMSO)
2.02 2.05 2.03 4.07 3.07 1.07 2.01 1.04
3.00
10.5 9.5 9.0 8.5 8.0 7.5 7.0 6.5 6.0 5.5 5.0 4.5 4.0 3.5 3.0 2.5 2.0 1.5 1.0 0.5 0.0
f1 (ppm)

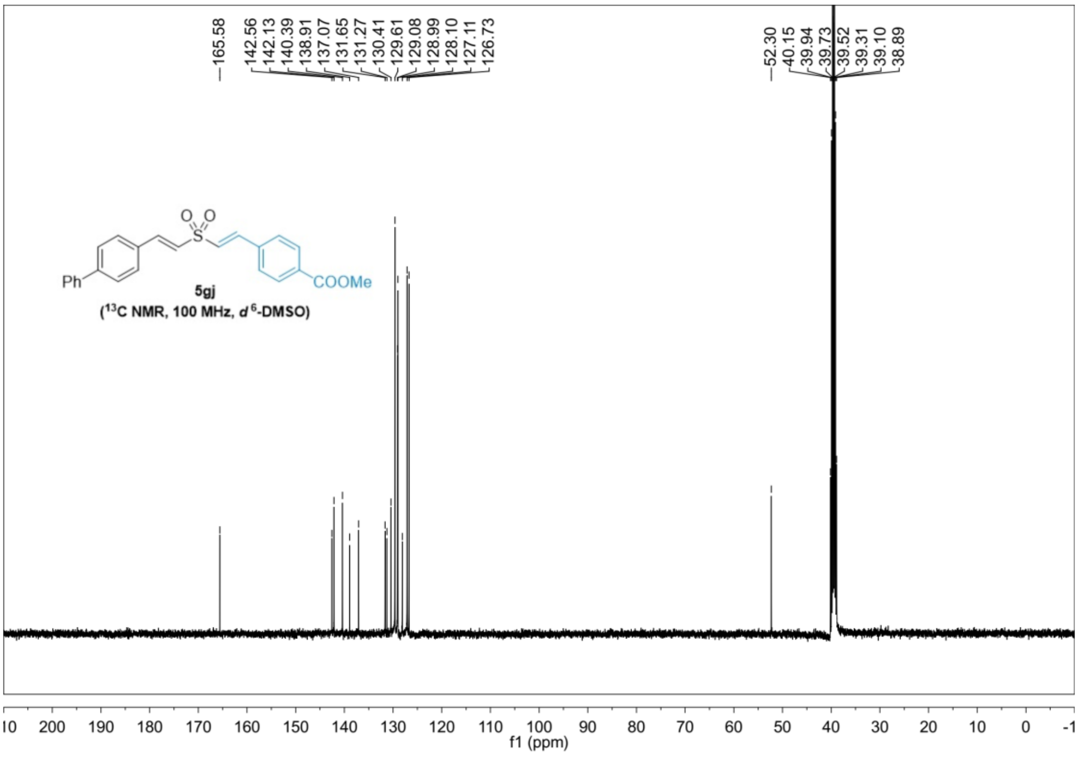
165.58
142.56 142.13 140.39 138.91 137.07 131.65 131.27 130.41 129.61 129.08 128.99 128.10 127.11 126.73
52.30 40.15 39.94 39.73 39.52 39.31 39.10 38.89
Ph
COOMe
5gj
(13C NMR, 100 MHz, d6-DMSO)
200 190 180 170 160 150 140 130 120 110 100 90 80 70 60 50 40 30 20 10 0
f1 (ppm)

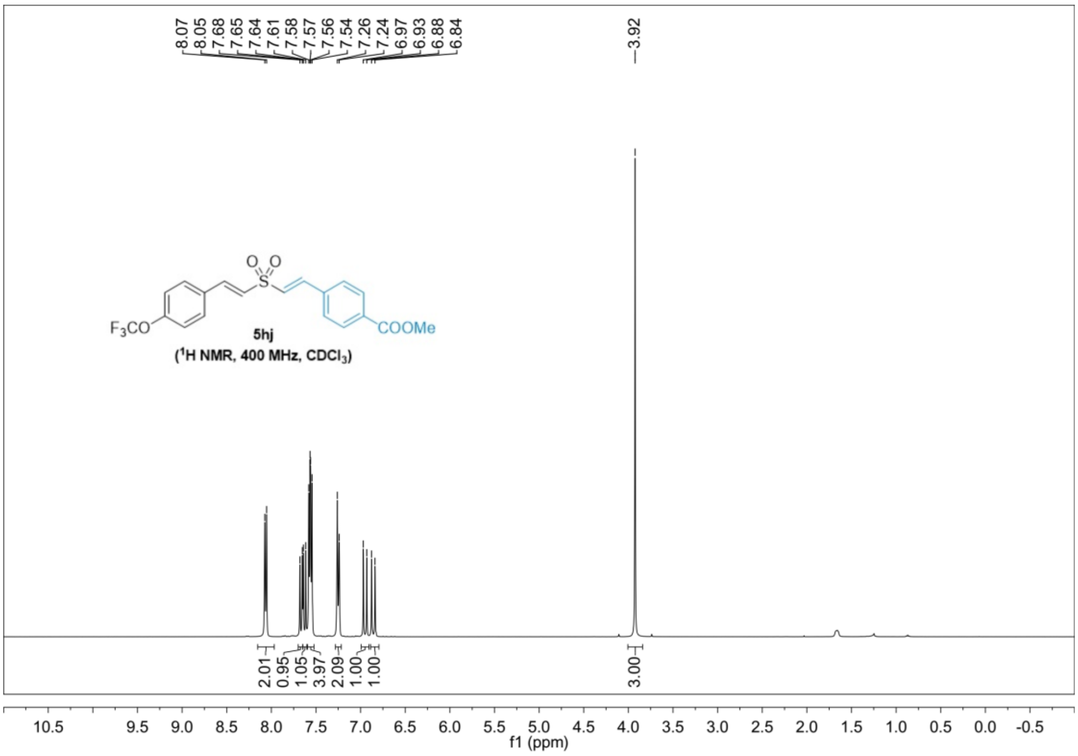
8.07
8.05
7.68
7.65
7.64
7.61
7.58
7.57
7.56
7.54
7.26
7.24
6.97
6.93
6.88
6.84
3.92
F3CO
5hj
COOMe
(1H NMR, 400 MHz, CDCl3)
2.01
0.95
1.05
3.97
2.09
1.00
1.00
3.00
f1 (ppm)

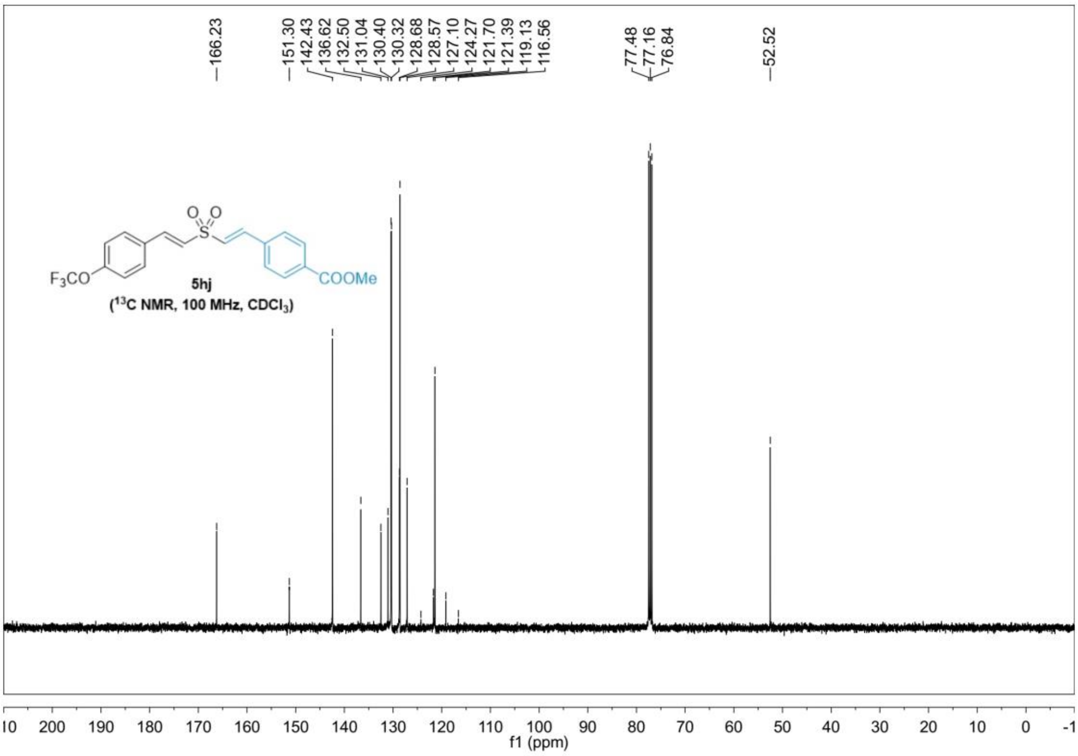
166.23
151.30
142.43
136.62
132.50
131.04
130.40
130.32
128.68
128.57
127.10
124.27
121.70
121.39
119.13
116.56
77.48
77.16
76.84
52.52
F3CO
5hj
COOMe
(13C NMR, 100 MHz, CDCl3)
f1 (ppm)

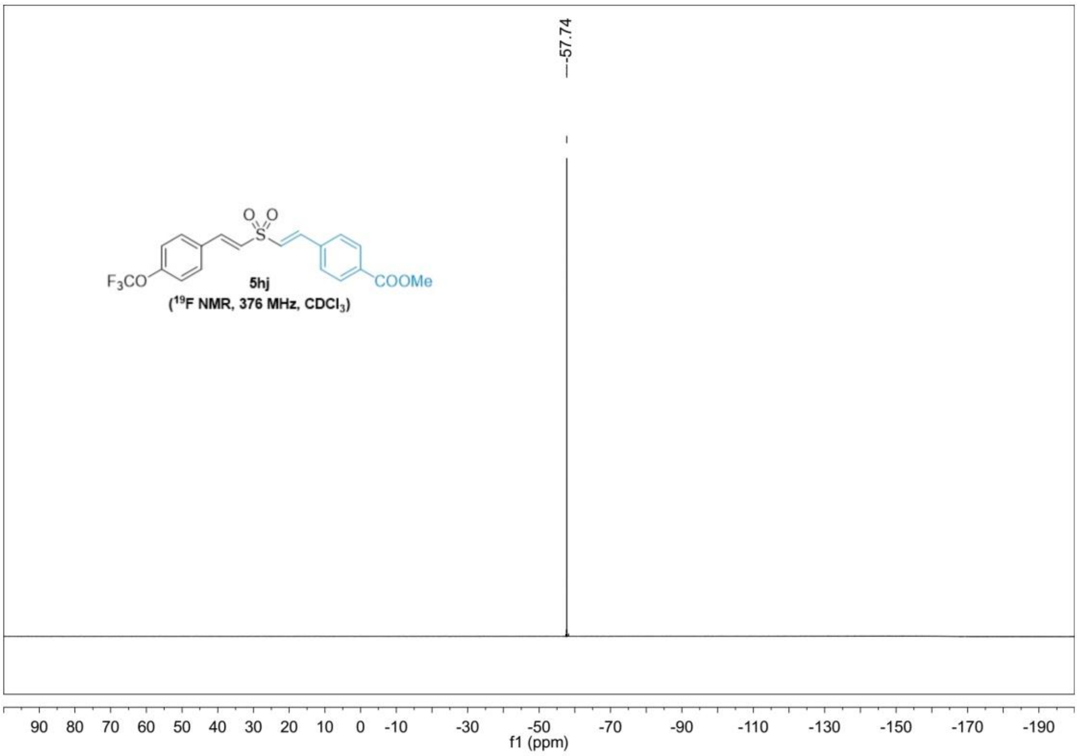
-57.74
F3CO
5hj
(19F NMR, 376 MHz, CDCl3)
COOMe
f1 (ppm)

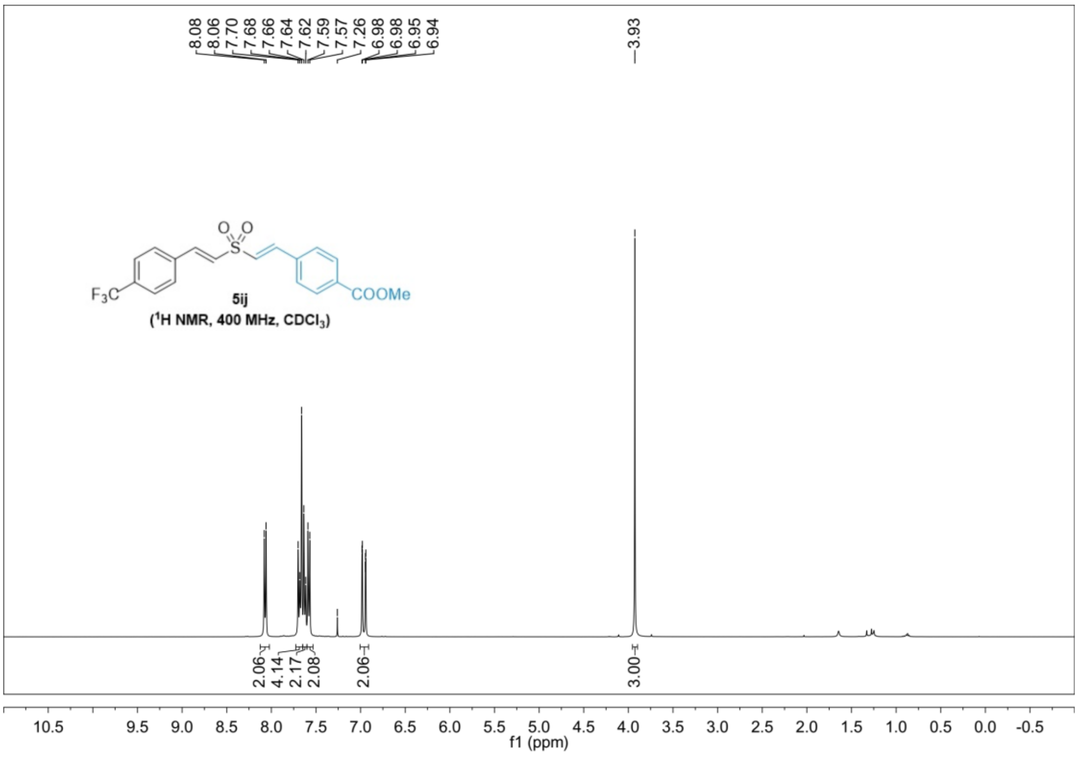
8.08
8.06
7.70
7.68
7.66
7.64
7.62
7.59
7.57
7.26
6.98
6.98
6.95
6.94
3.93
F3C
5ij
(1H NMR, 400 MHz, CDCl3)
COOMe
2.06
4.14
2.17
2.08
2.06
3.00
f1 (ppm)

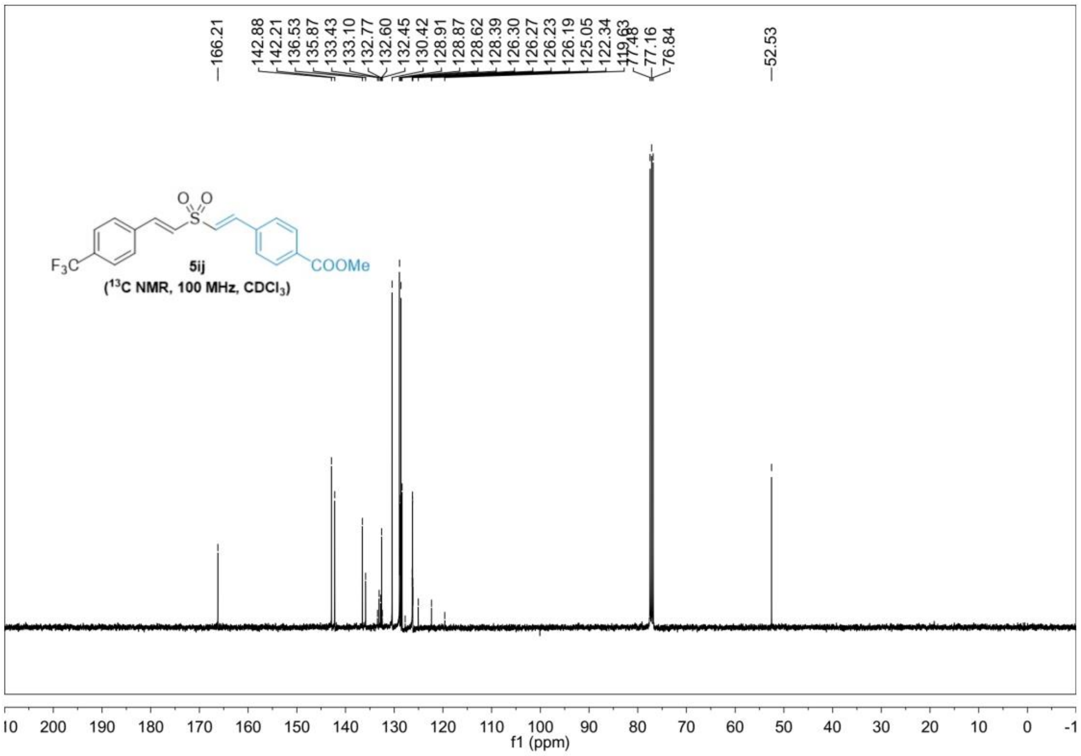

166.21
142.88
142.21
136.53
135.87
133.43
133.10
132.77
132.60
132.45
130.42
128.91
128.87
128.62
128.39
126.30
126.27
126.23
126.19
125.05
122.34
119.63
77.48
77.16
76.84
52.53
F3C
COOMe
5ij
(13C NMR, 100 MHz, CDCl3)
f1 (ppm)

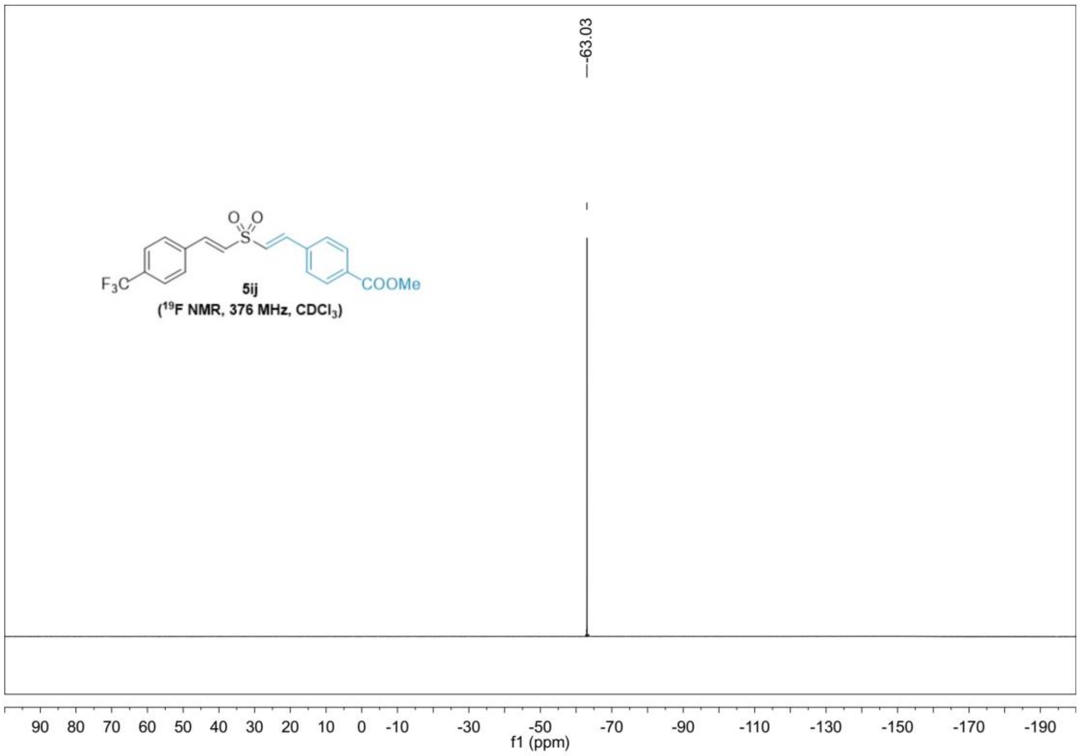

-63.03
F3C
COOMe
5ij
(19F NMR, 376 MHz, CDCl3)
f1 (ppm)

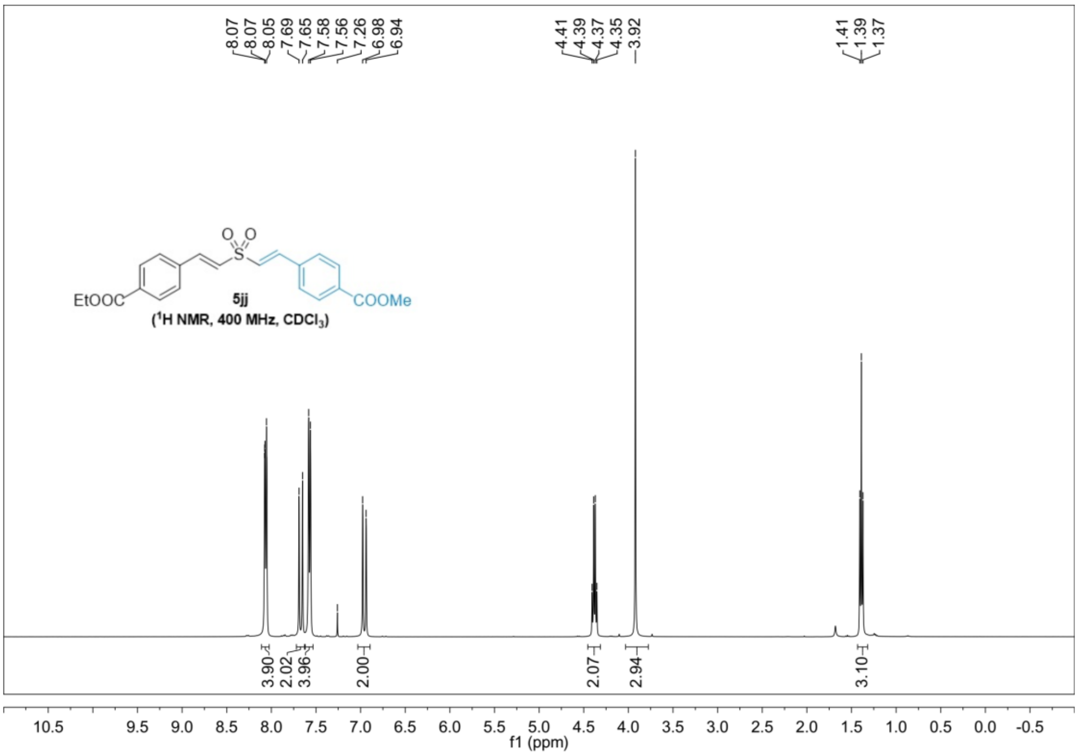
8.07
8.07
8.05
7.69
7.65
7.58
7.56
7.26
6.98
6.94
4.41
4.39
4.37
4.35
3.92
1.41
1.39
1.37
EtOOC
COOMe
5jj
(1H NMR, 400 MHz, CDCl3)
3.90
2.02
3.96
2.00
2.07
2.94
3.10
f1 (ppm)

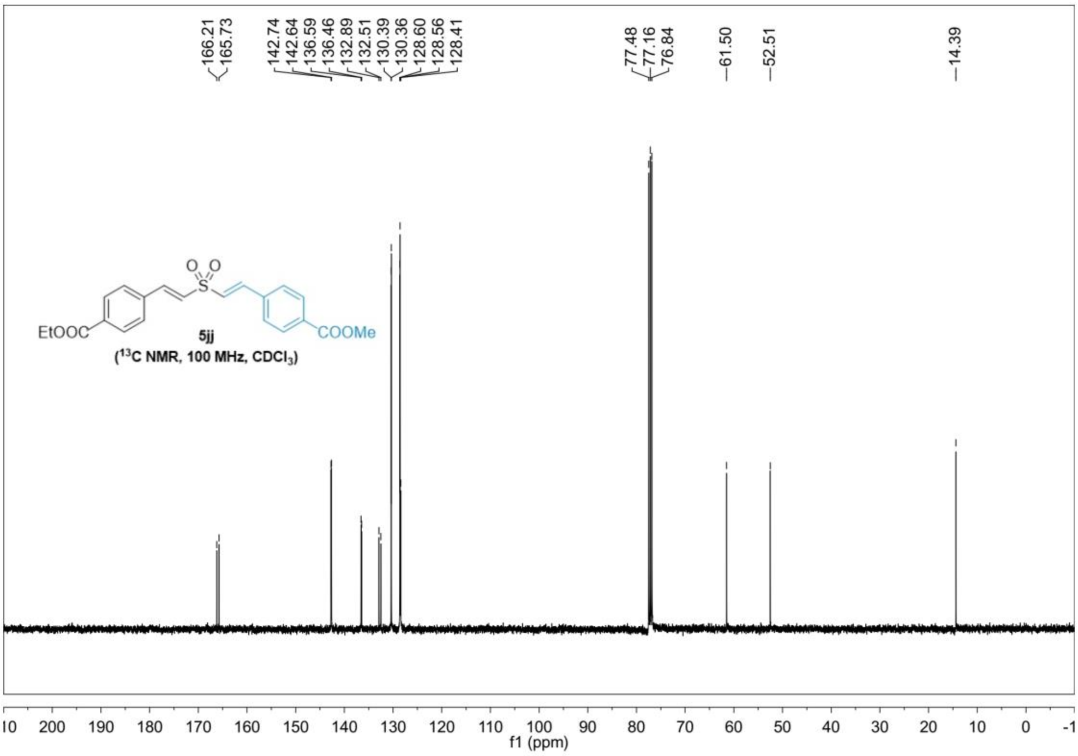
166.21
165.73
142.74
142.64
136.59
136.46
132.89
132.51
130.39
130.36
128.60
128.56
128.41
77.48
77.16
76.84
61.50
52.51
14.39
EtOOC
COOMe
5jj
(13C NMR, 100 MHz, CDCl3)
f1 (ppm)

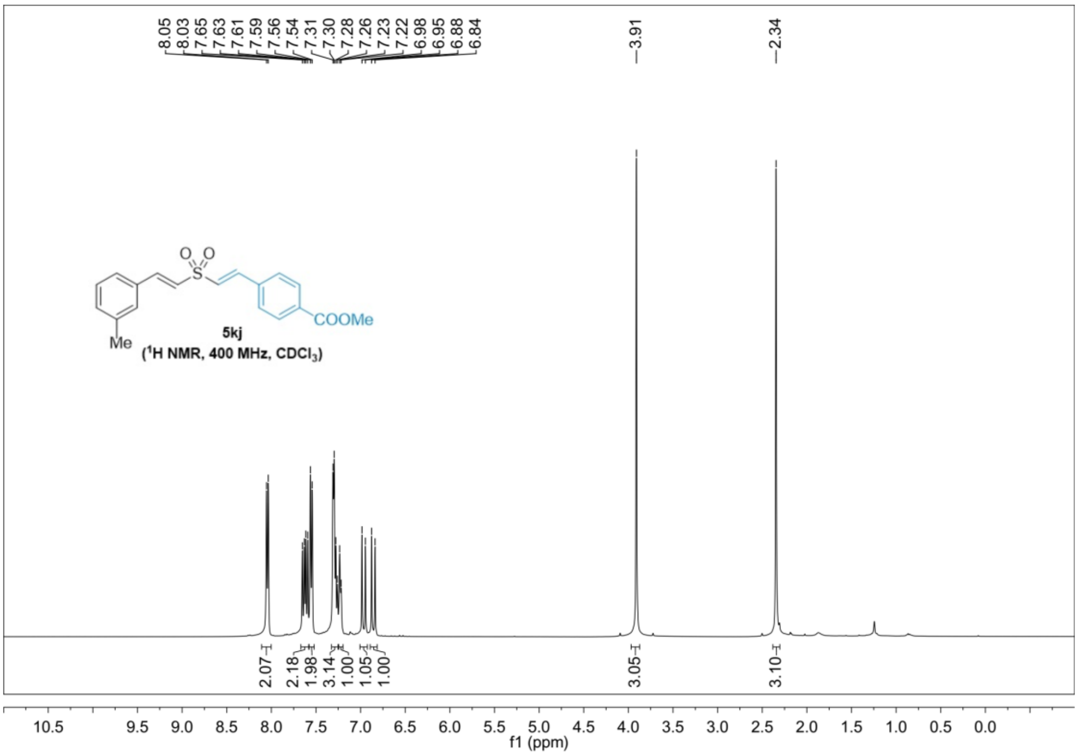
8.05
8.03
7.65
7.63
7.61
7.59
7.56
7.54
7.31
7.30
7.28
7.26
7.23
7.22
6.98
6.95
6.88
6.84
3.91
2.34
COOMe
Me
5kj
(1H NMR, 400 MHz, CDCl3)
2.07
2.18
1.98
3.14
1.00
1.05
1.00
3.05
3.10
f1 (ppm)

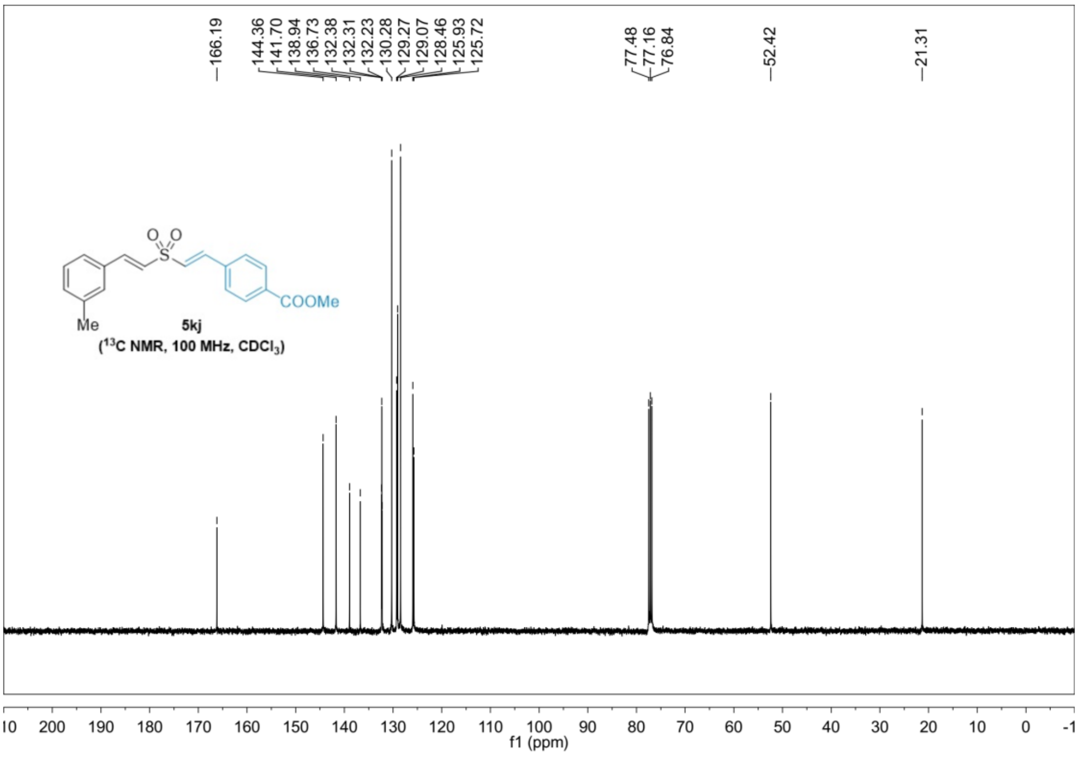
166.19
144.36
141.70
138.94
136.73
132.38
132.31
132.23
130.28
129.27
129.07
128.46
125.93
125.72
77.48
77.16
76.84
52.42
21.31
COOMe
Me
5kj
(13C NMR, 100 MHz, CDCl3)
f1 (ppm)

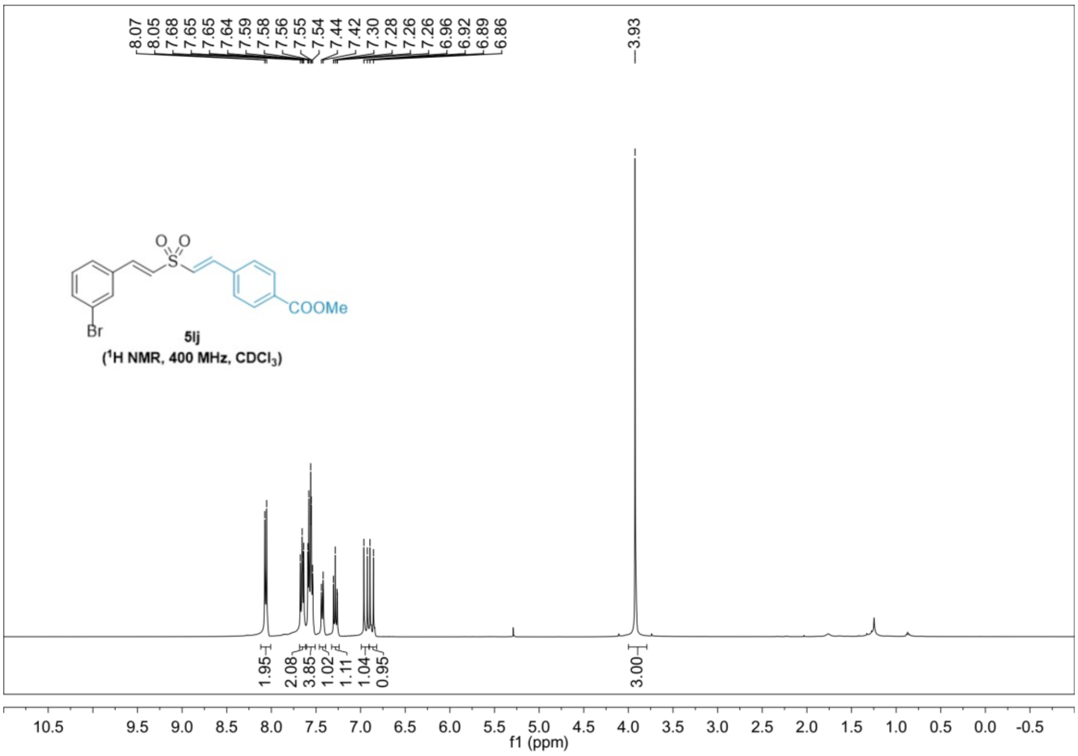
8.07
8.05
7.68
7.65
7.65
7.64
7.59
7.58
7.56
7.55
7.54
7.44
7.42
7.30
7.28
7.26
6.96
6.92
6.89
6.86
3.93
5lj
(1H NMR, 400 MHz, CDCl3)
COOMe
Br
1.95
2.08
3.85
1.02
1.11
1.04
0.95
3.00
f1 (ppm)

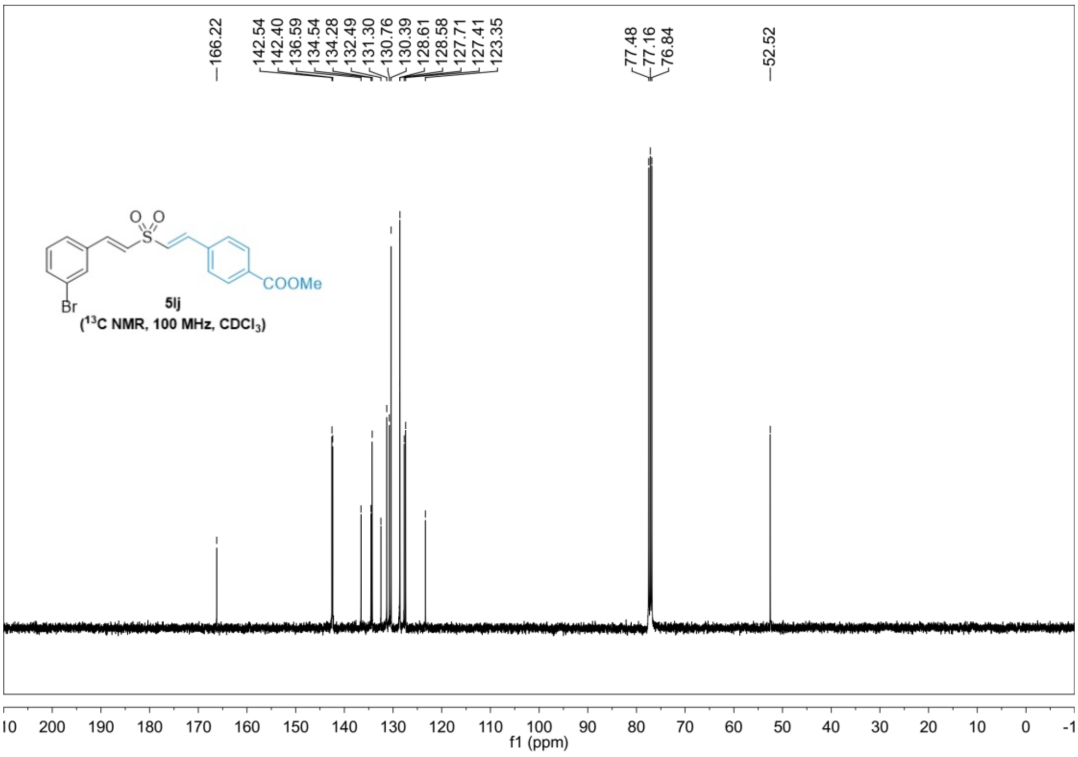
166.22
142.54
142.40
136.59
134.54
134.28
132.49
131.30
130.76
130.39
128.61
128.58
127.71
127.41
123.35
77.48
77.16
76.84
52.52
5lj
(13C NMR, 100 MHz, CDCl3)
COOMe
Br
f1 (ppm)

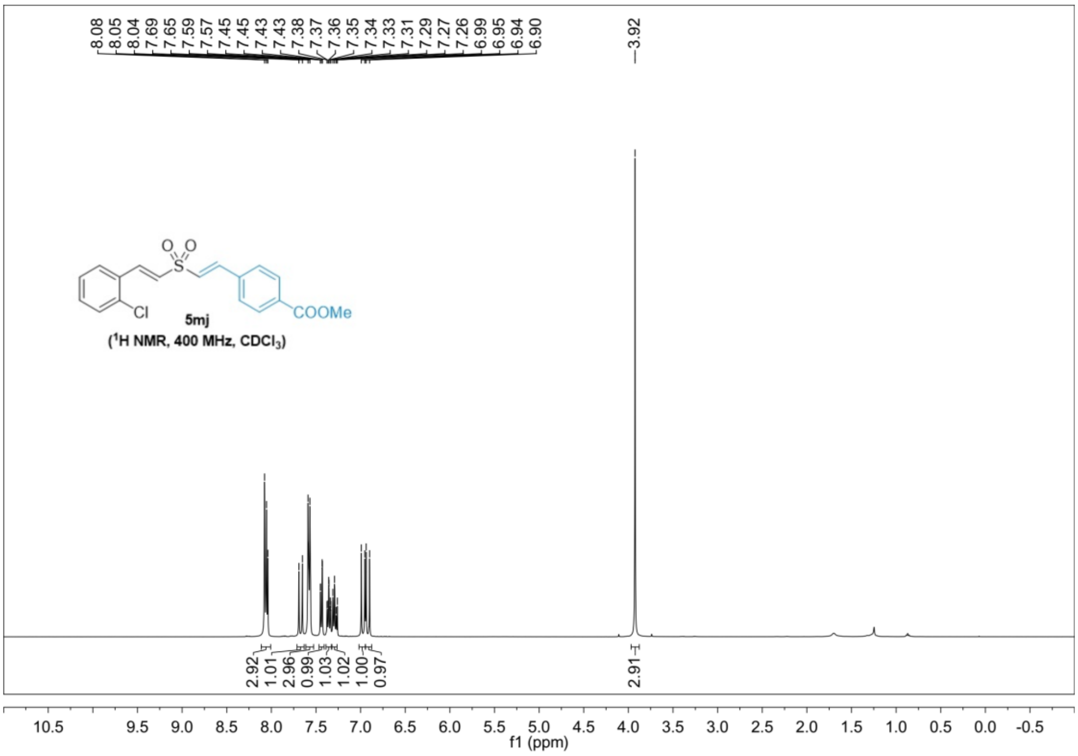
8.08 8.05 8.04 7.69 7.65 7.59 7.57 7.45 7.45 7.43 7.43 7.38 7.37 7.36 7.35 7.34 7.33 7.31 7.29 7.27 7.26 6.99 6.95 6.94 6.90
3.92
Cl
COOMe
5mj
(1H NMR, 400 MHz, CDCl3)
2.92 1.01 2.96 0.99 1.03 1.02 1.00 0.97
2.91
10.5 9.5 9.0 8.5 8.0 7.5 7.0 6.5 6.0 5.5 5.0 4.5 4.0 3.5 3.0 2.5 2.0 1.5 1.0 0.5 0.0 -0.5
f1 (ppm)

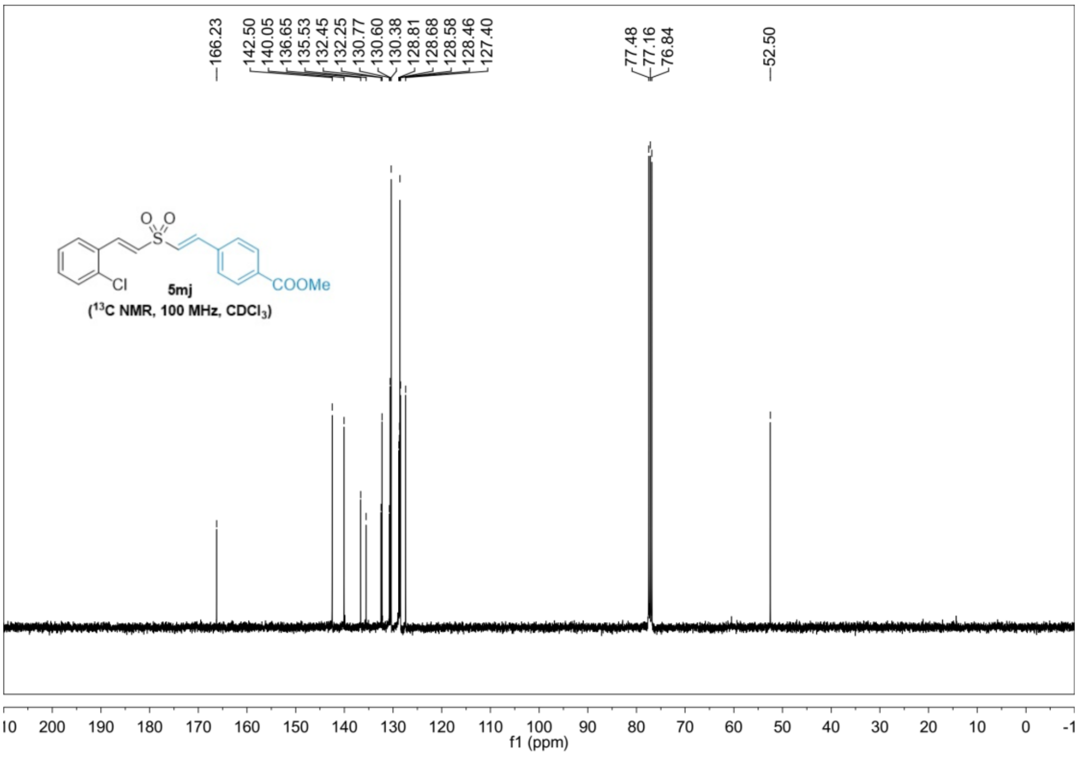
166.23
142.50 140.05 136.65 135.53 132.45 132.25 130.77 130.60 130.38 128.81 128.68 128.58 128.46 127.40
77.48 77.16 76.84
52.50
Cl
COOMe
5mj
(13C NMR, 100 MHz, CDCl3)
200 190 180 170 160 150 140 130 120 110 100 90 80 70 60 50 40 30 20 10 0
f1 (ppm)

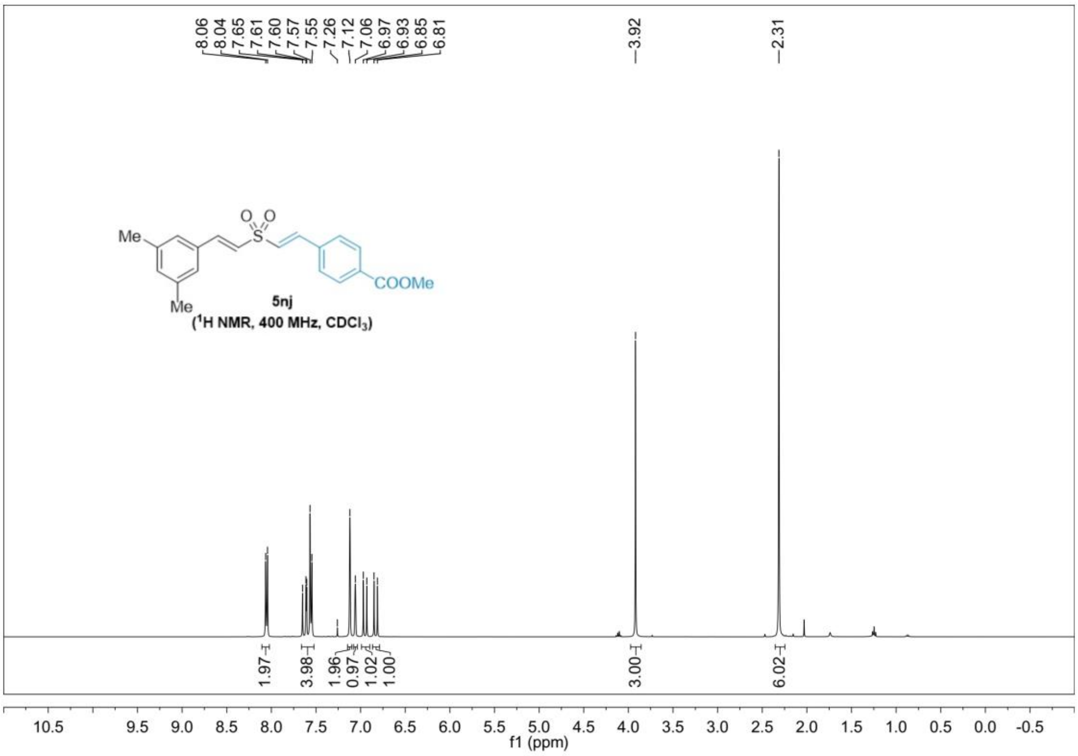
8.06
8.04
7.65
7.61
7.60
7.57
7.55
7.26
7.12
7.06
6.97
6.93
6.85
6.81
3.92
2.31
Me
Me
COOMe
5nj
(1H NMR, 400 MHz, CDCl3)
1.97
3.98
1.96
0.97
1.02
1.00
3.00
6.02
f1 (ppm)

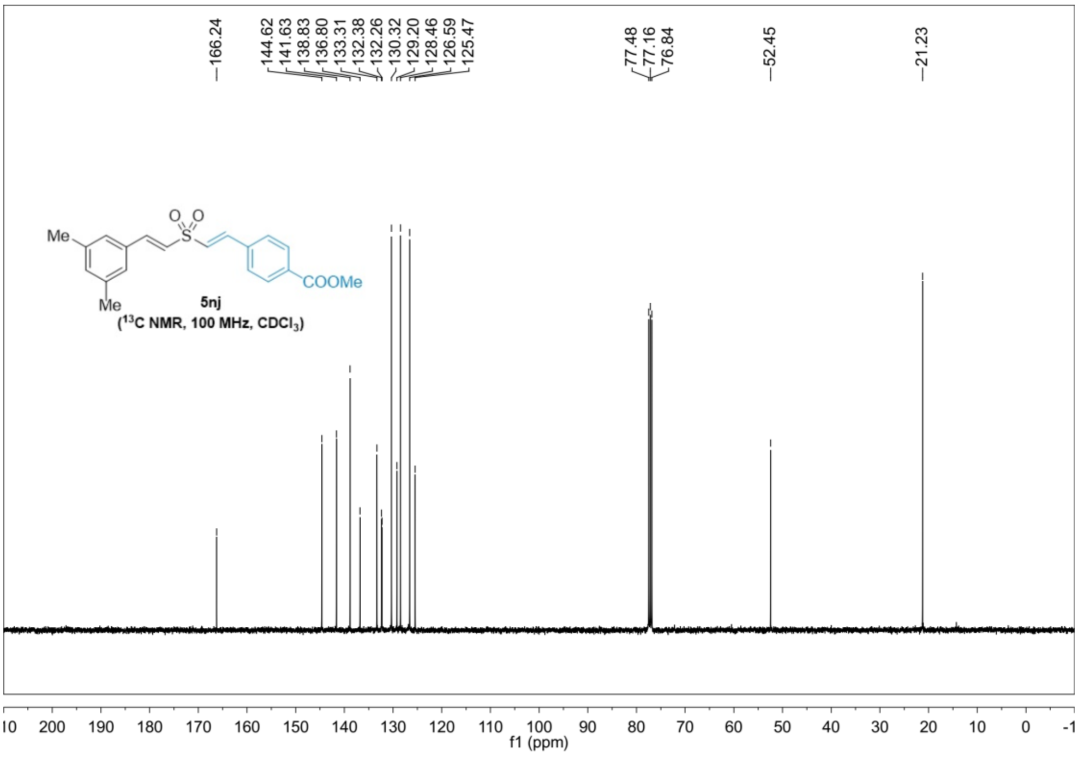
166.24
144.62
141.63
138.83
136.80
133.31
132.38
132.26
130.32
129.20
128.46
126.59
125.47
77.48
77.16
76.84
52.45
21.23
Me
Me
COOMe
5nj
(13C NMR, 100 MHz, CDCl3)
f1 (ppm)

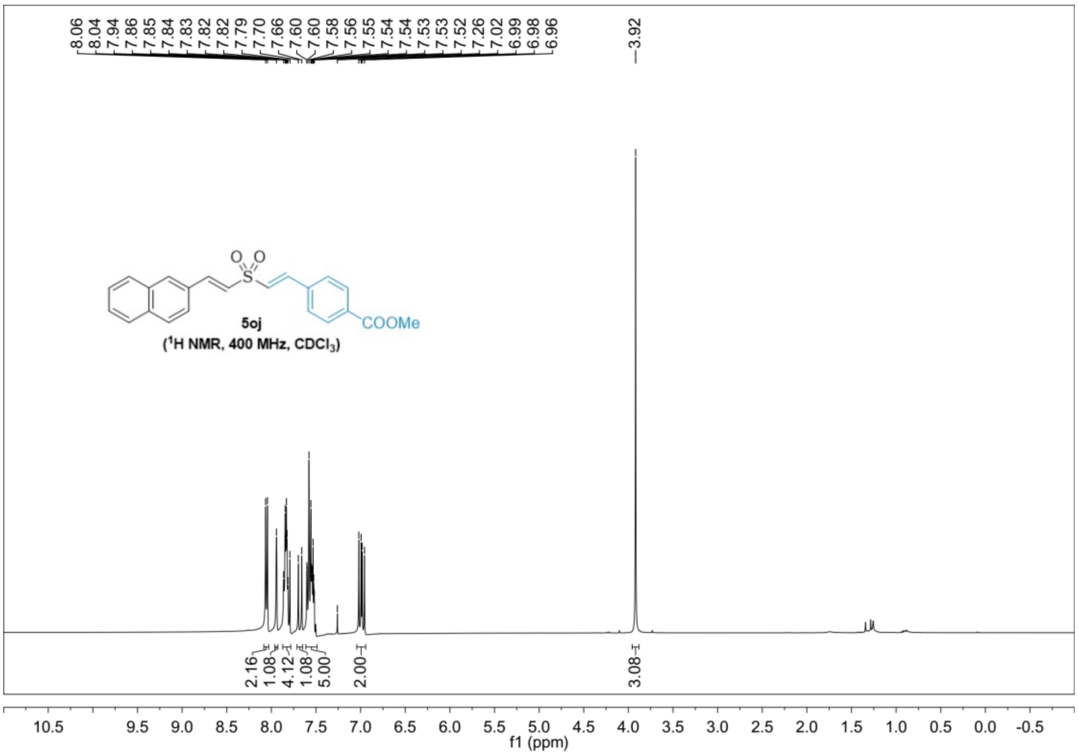
5oj
(1H NMR, 400 MHz, CDCl3)
COOMe
f1 (ppm)

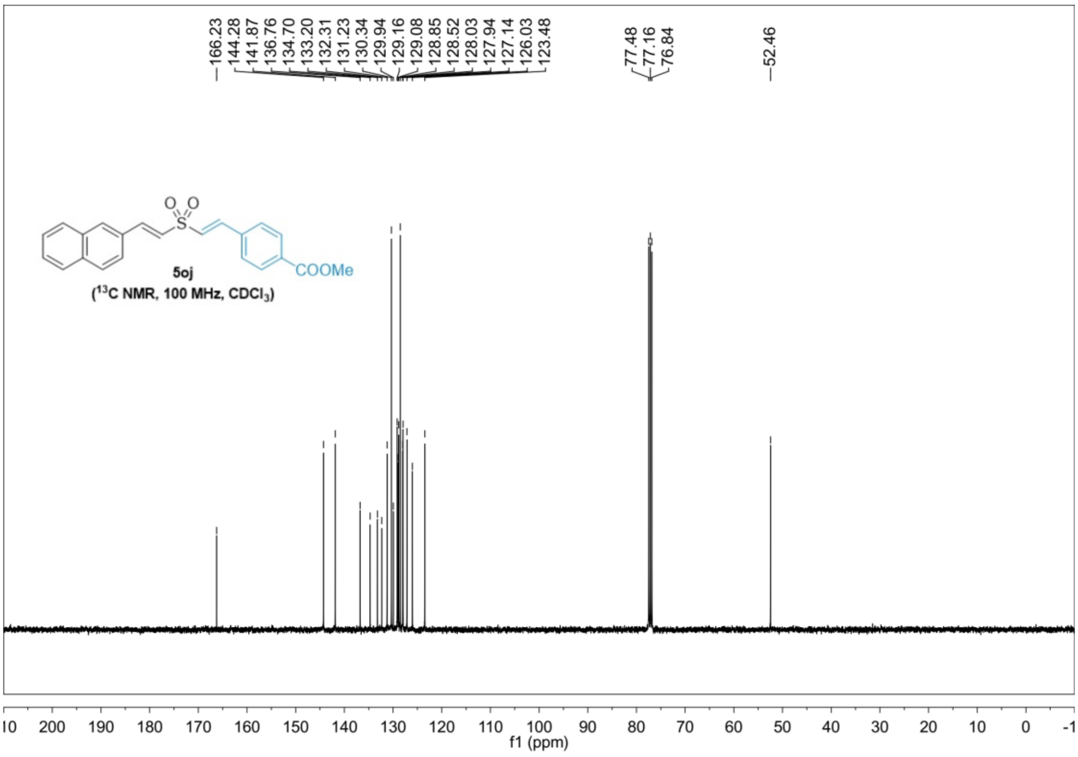
5oj
(13C NMR, 100 MHz, CDCl3)
COOMe
f1 (ppm)

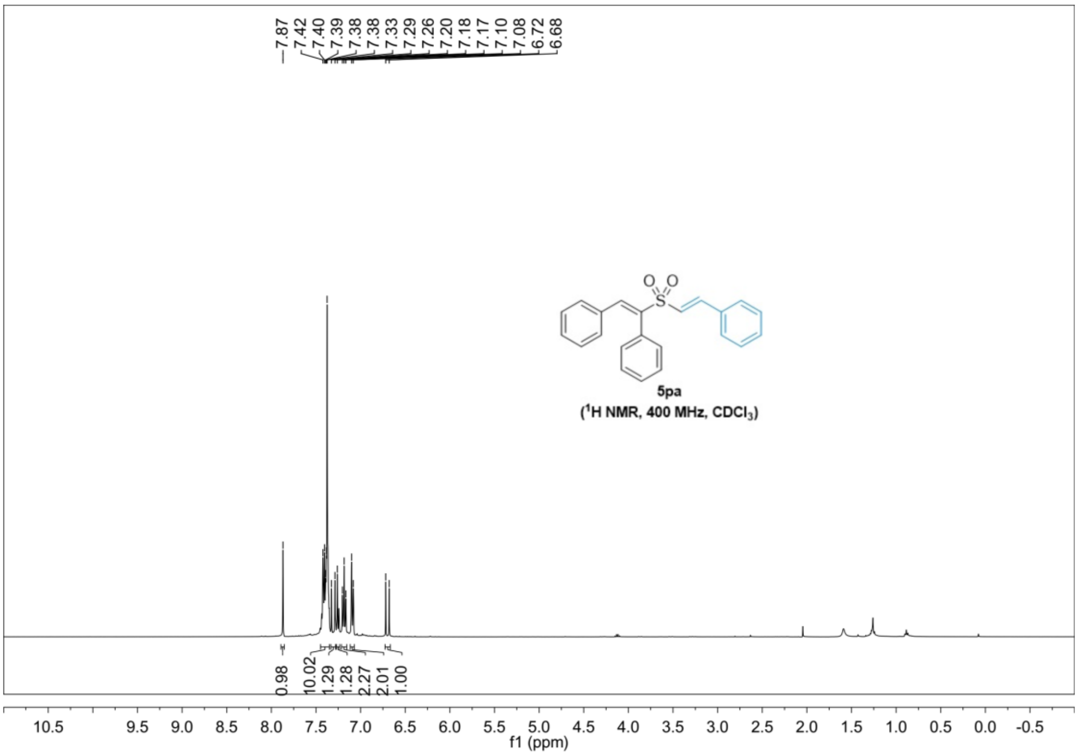
7.87
7.42
7.40
7.39
7.38
7.38
7.33
7.29
7.26
7.20
7.18
7.17
7.10
7.08
6.72
6.68
5pa
(^{1}H NMR, 400 MHz, CDCl$_3$)
0.98
10.02
1.29
1.28
2.27
2.01
1.00
f1 (ppm)

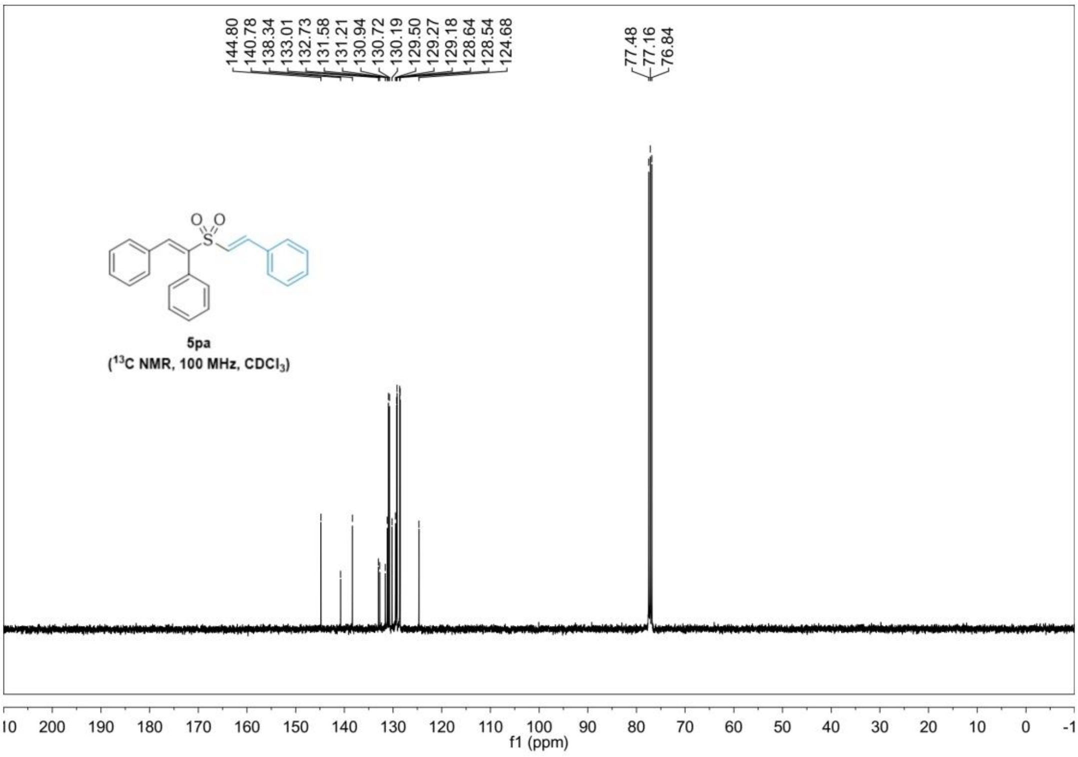
144.80
140.78
138.34
133.01
132.73
131.58
131.21
130.94
130.72
130.19
129.50
129.27
129.18
128.64
128.54
124.68
77.48
77.16
76.84
5pa
(^{13}C NMR, 100 MHz, CDCl$_3$)
f1 (ppm)

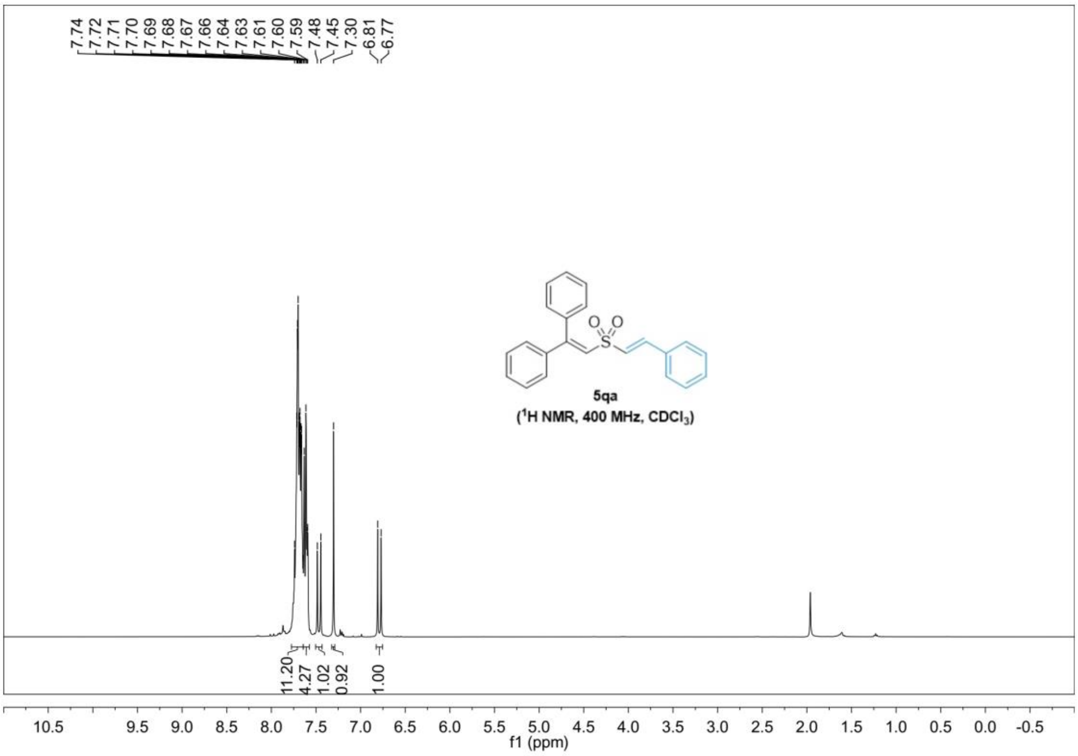
7.74
7.72
7.71
7.70
7.69
7.68
7.67
7.66
7.64
7.63
7.61
7.60
7.59
7.48
7.45
7.30
6.81
6.77
5qa
(^{1}H NMR, 400 MHz, CDCl$_3$)
11.20
4.27
1.02
0.92
1.00
10.5 9.5 9.0 8.5 8.0 7.5 7.0 6.5 6.0 5.5 5.0 4.5 4.0 3.5 3.0 2.5 2.0 1.5 1.0 0.5 0.0 -0.5
f1 (ppm)

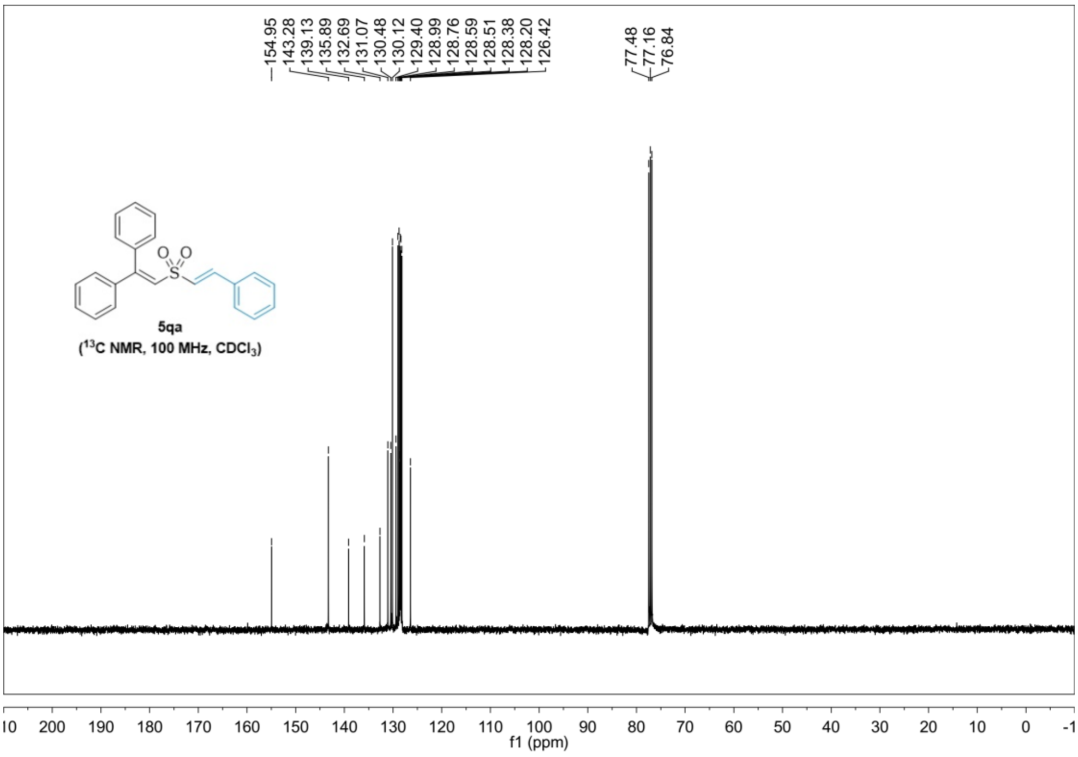
154.95
143.28
139.13
135.89
132.69
131.07
130.48
130.12
129.40
128.99
128.76
128.59
128.51
128.38
128.20
126.42
77.48
77.16
76.84
5qa
(^{13}C NMR, 100 MHz, CDCl$_3$)
200 190 180 170 160 150 140 130 120 110 100 90 80 70 60 50 40 30 20 10 0
f1 (ppm)

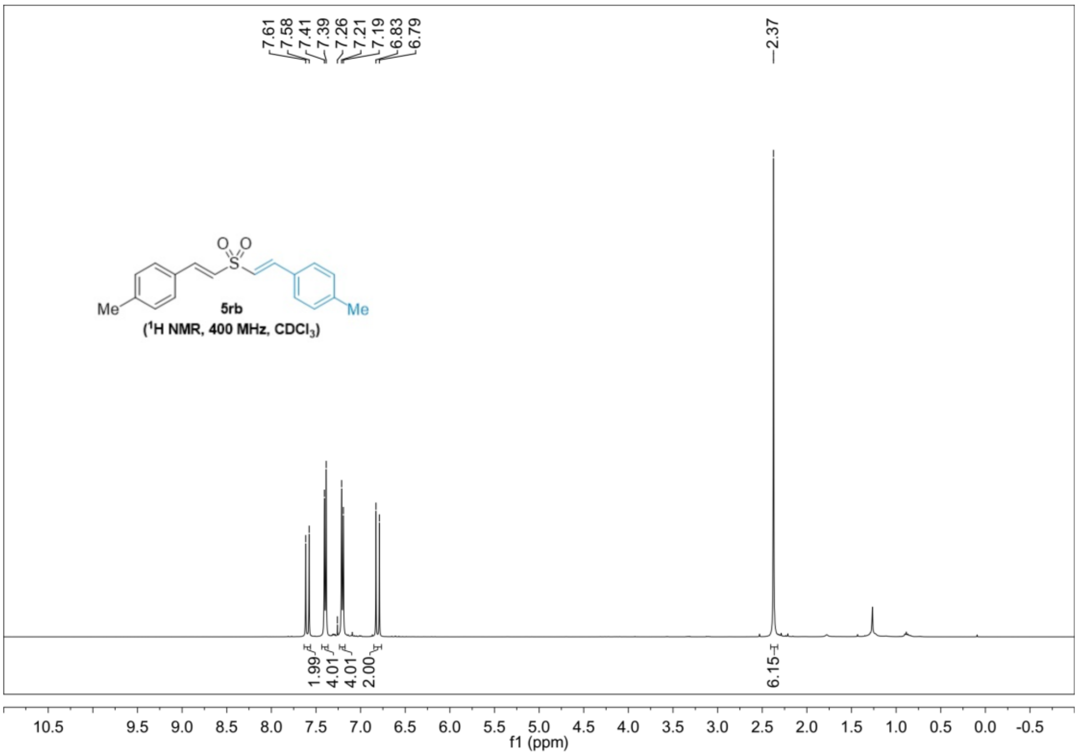
7.61
7.58
7.41
7.39
7.26
7.21
7.19
6.83
6.79
2.37
Me
O
O
S
Me
5rb
(^{1}H NMR, 400 MHz, CDCl$_3$)
1.99
4.01
4.01
2.00
6.15
10.5
9.5
9.0
8.5
8.0
7.5
7.0
6.5
6.0
5.5
5.0
4.5
4.0
3.5
3.0
2.5
2.0
1.5
1.0
0.5
0.0
-0.5
f1 (ppm)

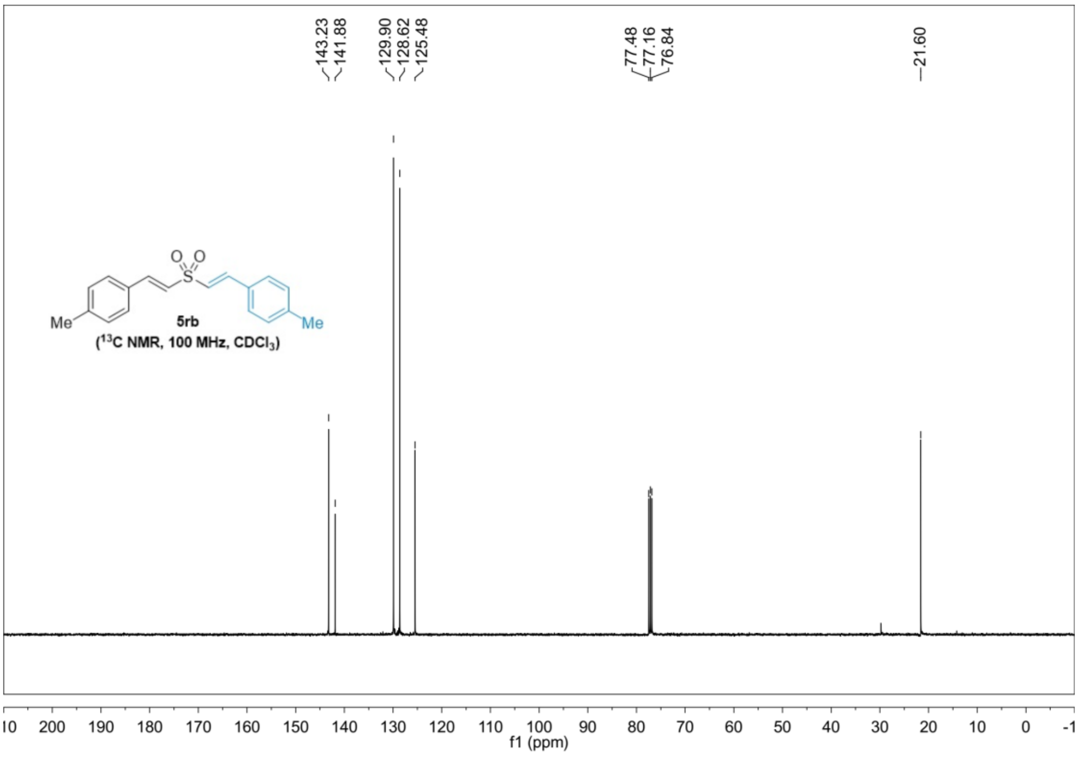
143.23
141.88
129.90
128.62
125.48
77.48
77.16
76.84
21.60
Me
O
O
S
Me
5rb
(^{13}C NMR, 100 MHz, CDCl$_3$)
200
190
180
170
160
150
140
130
120
110
100
90
80
70
60
50
40
30
20
10
0
f1 (ppm)

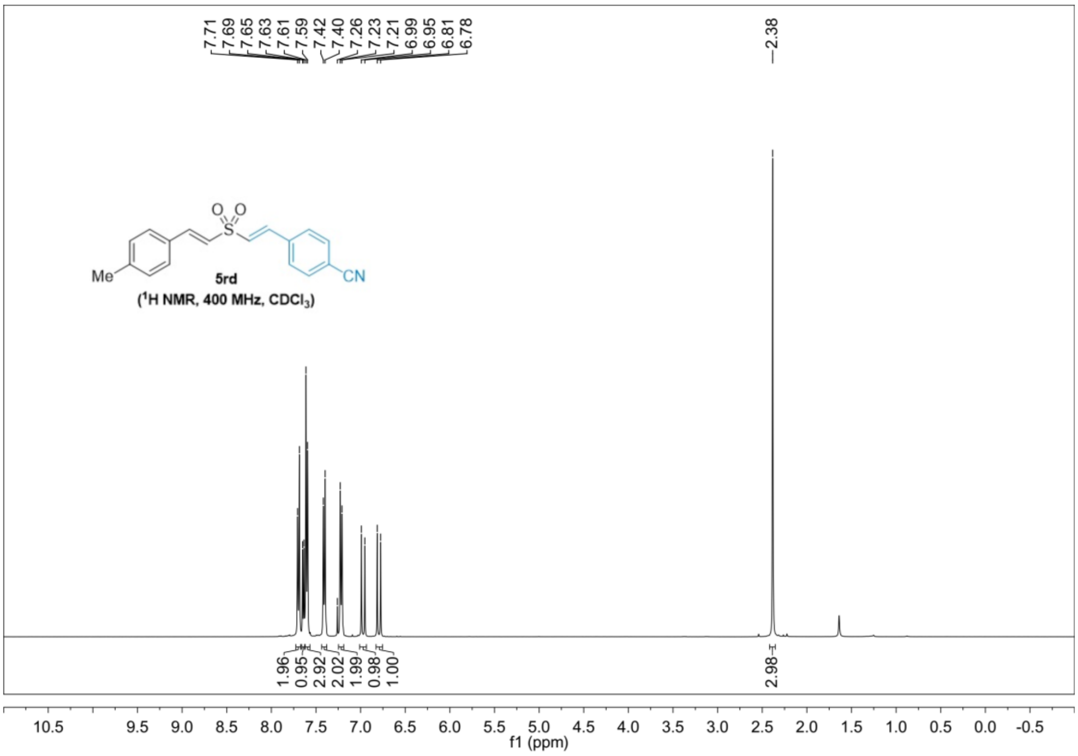
7.71
7.69
7.65
7.63
7.61
7.59
7.42
7.40
7.26
7.23
7.21
6.99
6.95
6.81
6.78
2.38
5rd
(1H NMR, 400 MHz, CDCl3)
1.96
0.95
2.92
2.02
1.99
0.98
1.00
2.98
f1 (ppm)

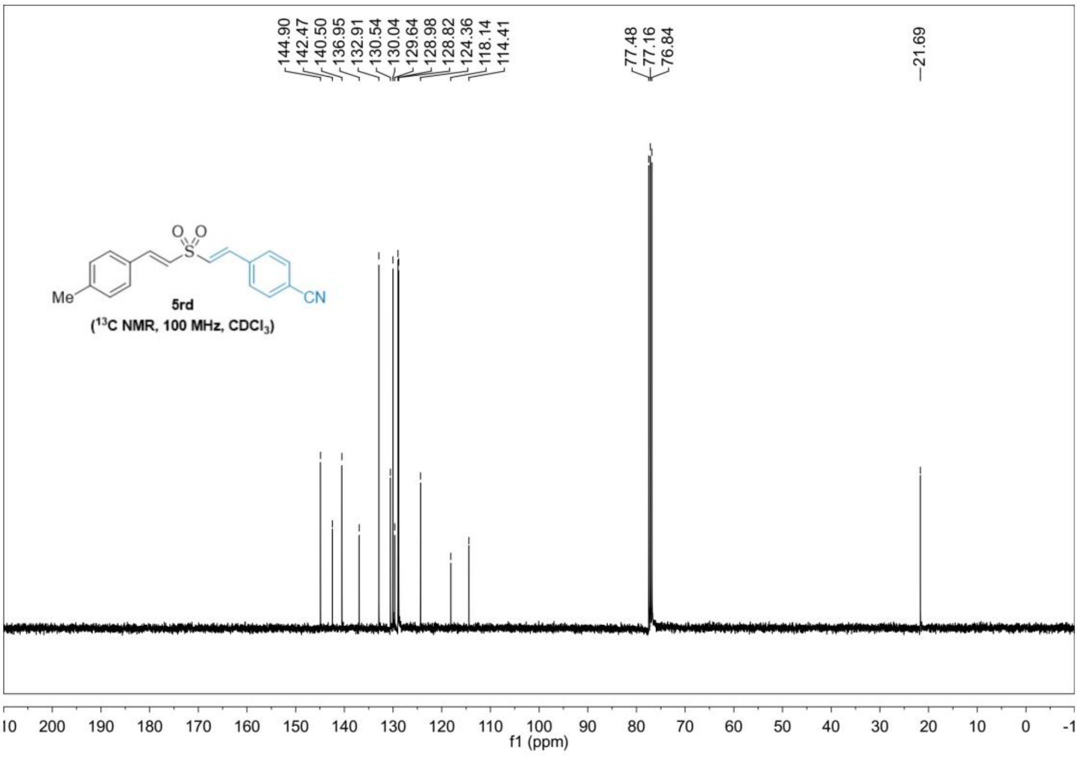
144.90
142.47
140.50
136.95
132.91
130.54
130.04
129.64
128.98
128.82
124.36
118.14
114.41
77.48
77.16
76.84
21.69
5rd
(13C NMR, 100 MHz, CDCl3)
f1 (ppm)

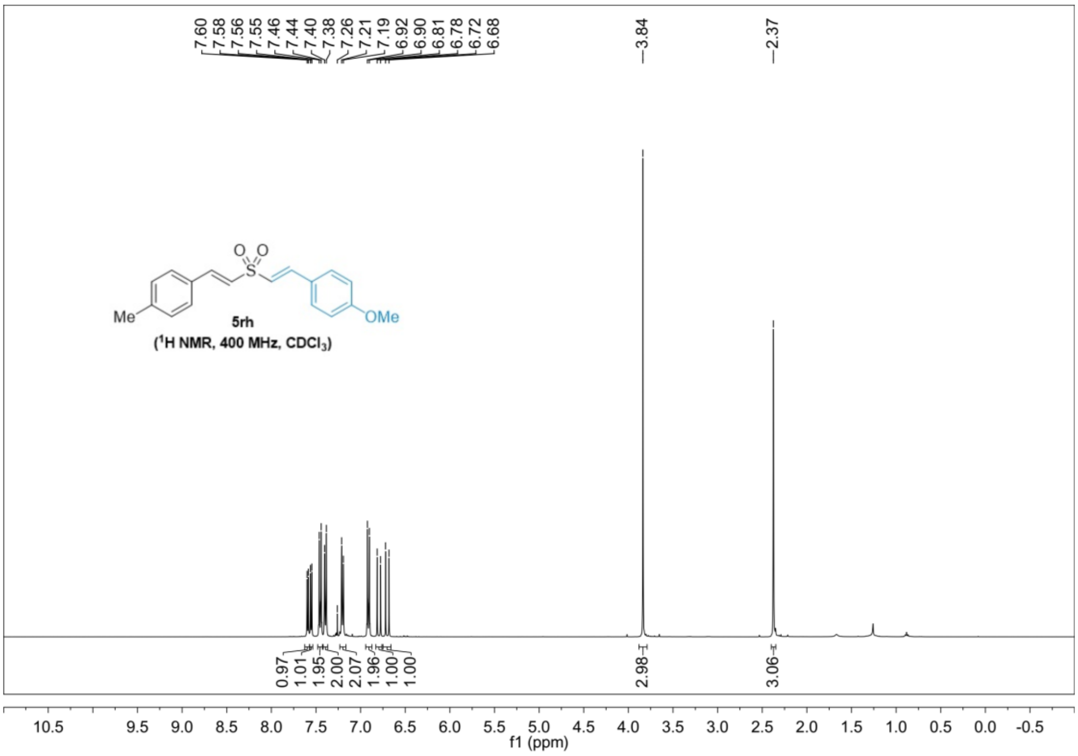
7.60 7.58 7.56 7.55 7.46 7.44 7.40 7.38 7.26 7.21 7.19 6.92 6.90 6.81 6.78 6.72 6.68
3.84
2.37
Me
OMe
5rh
(^{1}H NMR, 400 MHz, CDCl$_3$)
0.97 1.01 1.95 2.00 2.07 1.96 1.00 1.00
2.98
3.06
f1 (ppm)

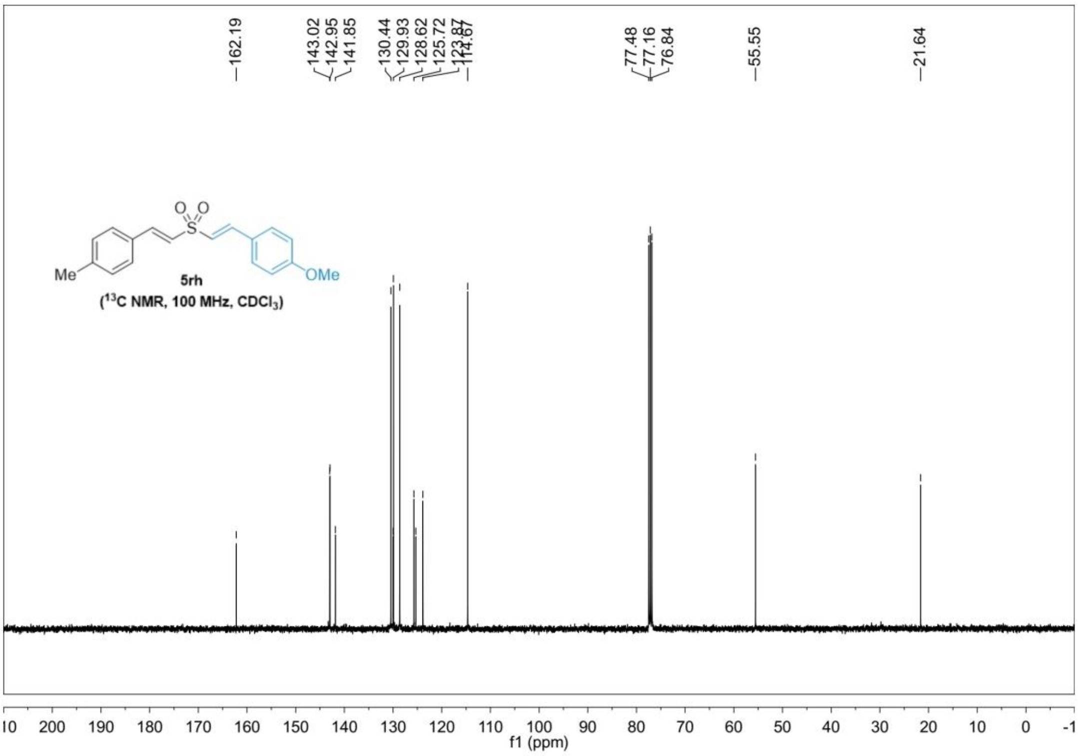
162.19
143.02 142.95 141.85
130.44 129.93 128.62 125.72 123.87 114.67
77.48 77.16 76.84
55.55
21.64
Me
OMe
5rh
(^{13}C NMR, 100 MHz, CDCl$_3$)
f1 (ppm)

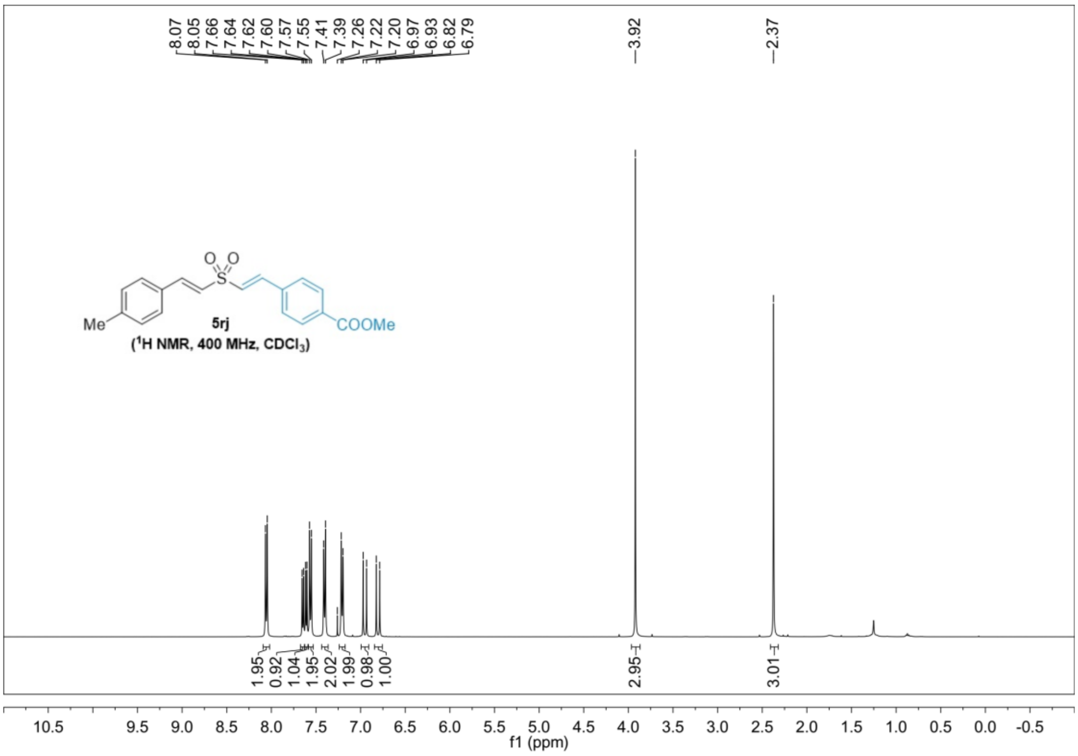

8.07 8.05 7.66 7.64 7.62 7.60 7.57 7.55 7.41 7.39 7.26 7.22 7.20 6.97 6.93 6.82 6.79
3.92
2.37
Me
COOMe
5rj
(¹H NMR, 400 MHz, CDCl3)
1.95 0.92 1.04 1.95 2.02 1.99 0.98 1.00
2.95
3.01
f1 (ppm)

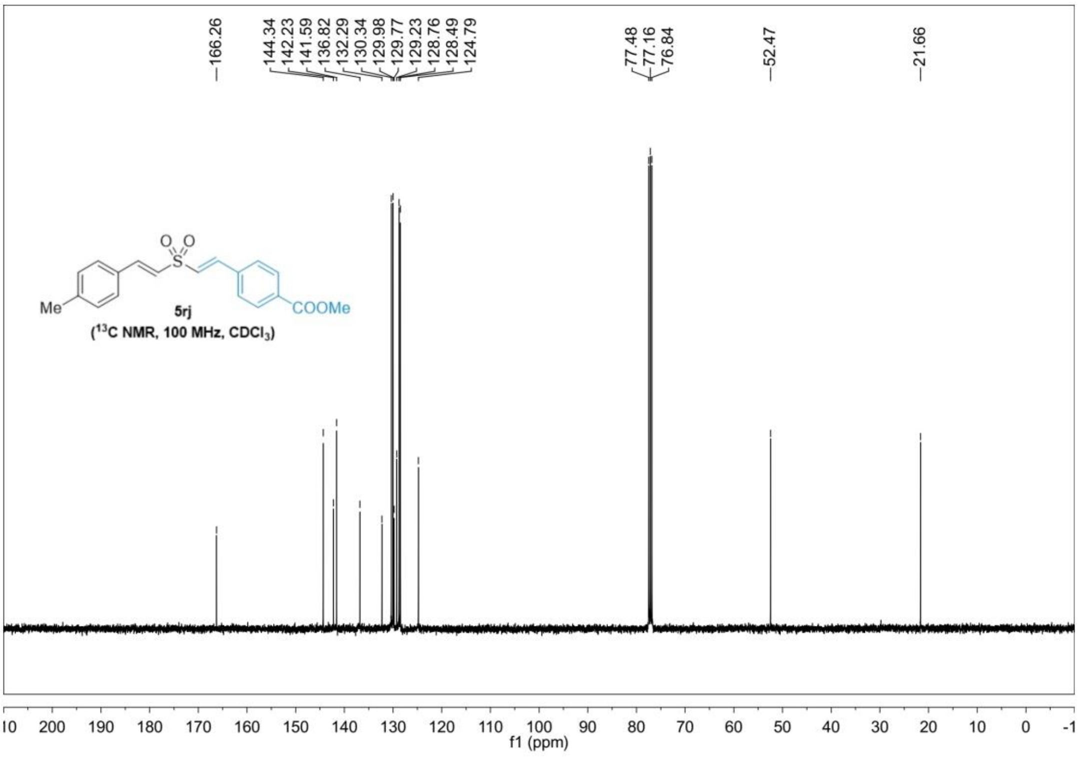

166.26
144.34 142.23 141.59 136.82 132.29 130.34 129.98 129.77 129.23 128.76 128.49 124.79
77.48 77.16 76.84
52.47
21.66
Me
COOMe
5rj
(¹³C NMR, 100 MHz, CDCl3)
f1 (ppm)

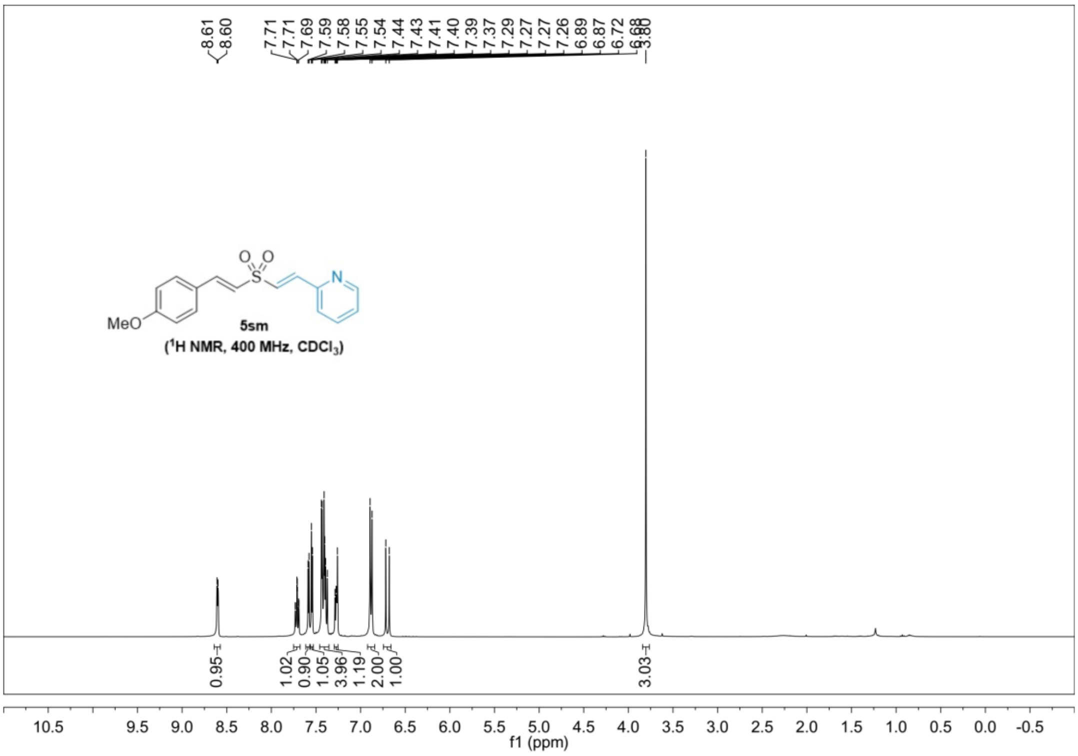
8.61
8.60
7.71
7.71
7.69
7.59
7.58
7.55
7.54
7.44
7.43
7.41
7.40
7.39
7.37
7.29
7.27
7.27
7.26
6.89
6.87
6.72
6.68
3.80
O O
S
N
MeO
5sm
(^{1}H NMR, 400 MHz, CDCl$_3$)
0.95
1.02
0.90
1.05
3.96
1.19
2.00
1.00
3.03
10.5 9.5 9.0 8.5 8.0 7.5 7.0 6.5 6.0 5.5 5.0 4.5 4.0 3.5 3.0 2.5 2.0 1.5 1.0 0.5 0.0 -0.5
f1 (ppm)

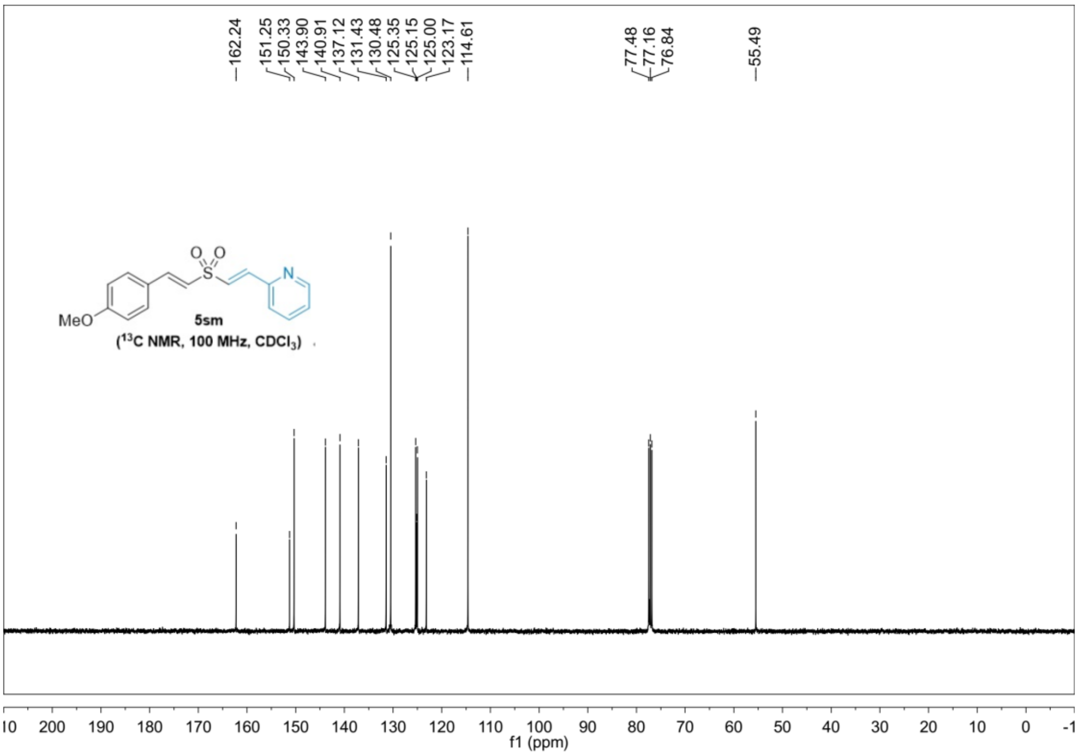
162.24
151.25
150.33
143.90
140.91
137.12
131.43
130.48
125.35
125.15
125.00
123.17
114.61
77.48
77.16
76.84
55.49
O O
S
N
MeO
5sm
(^{13}C NMR, 100 MHz, CDCl$_3$)
10 200 190 180 170 160 150 140 130 120 110 100 90 80 70 60 50 40 30 20 10 0 -1
f1 (ppm)

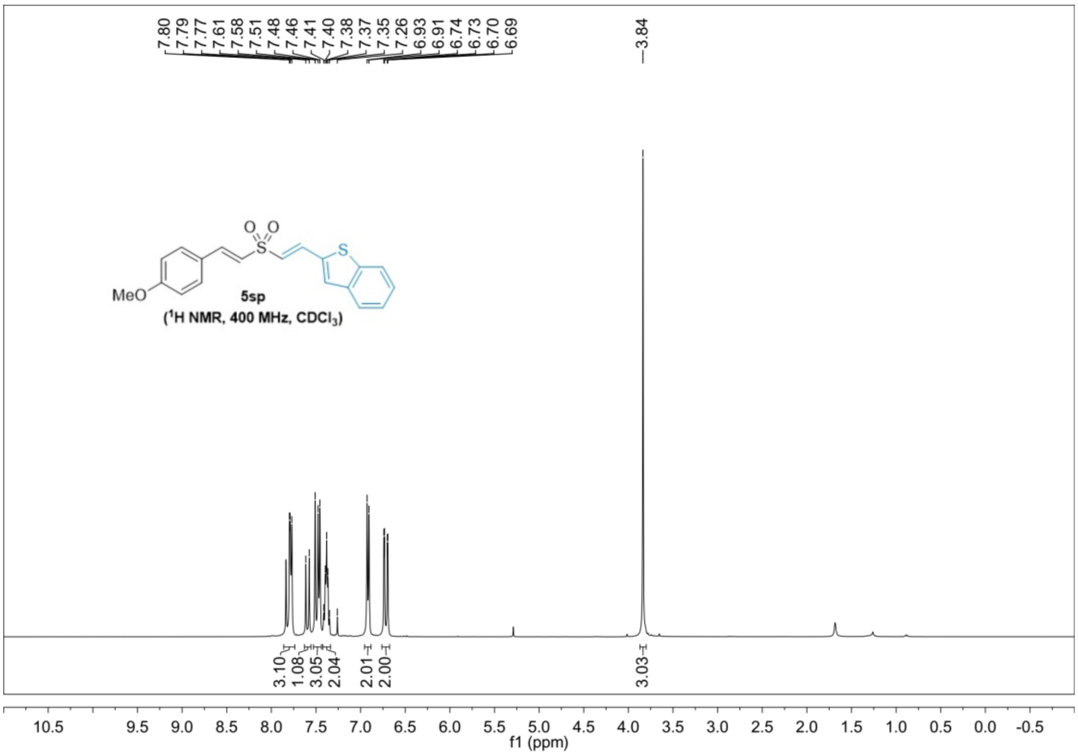
7.80
7.79
7.77
7.61
7.58
7.51
7.48
7.46
7.41
7.40
7.38
7.37
7.35
7.26
6.93
6.91
6.74
6.73
6.70
6.69
3.84
MeO
O
O
S
S
5sp
(1H NMR, 400 MHz, CDCl3)
3.10
1.08
3.05
2.04
2.01
2.00
3.03
f1 (ppm)

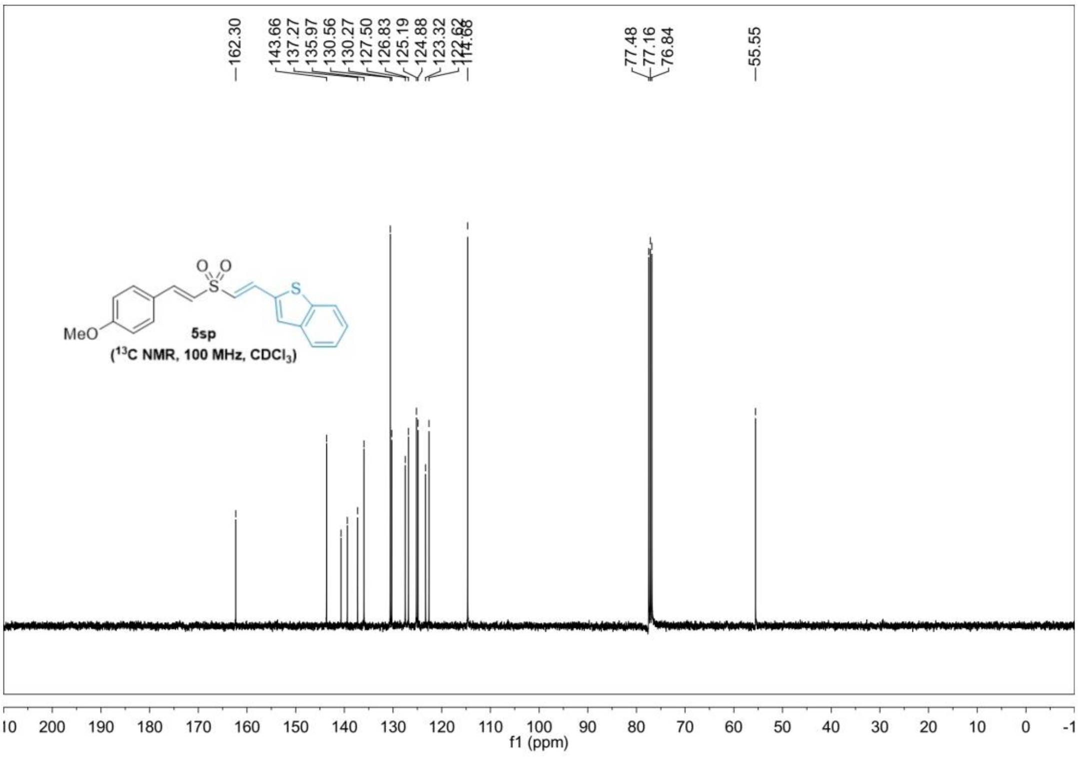
162.30
143.66
137.27
135.97
130.56
130.27
127.50
126.83
125.19
124.88
123.32
77.48
77.16
76.84
55.55
MeO
O
O
S
S
5sp
(13C NMR, 100 MHz, CDCl3)
f1 (ppm)

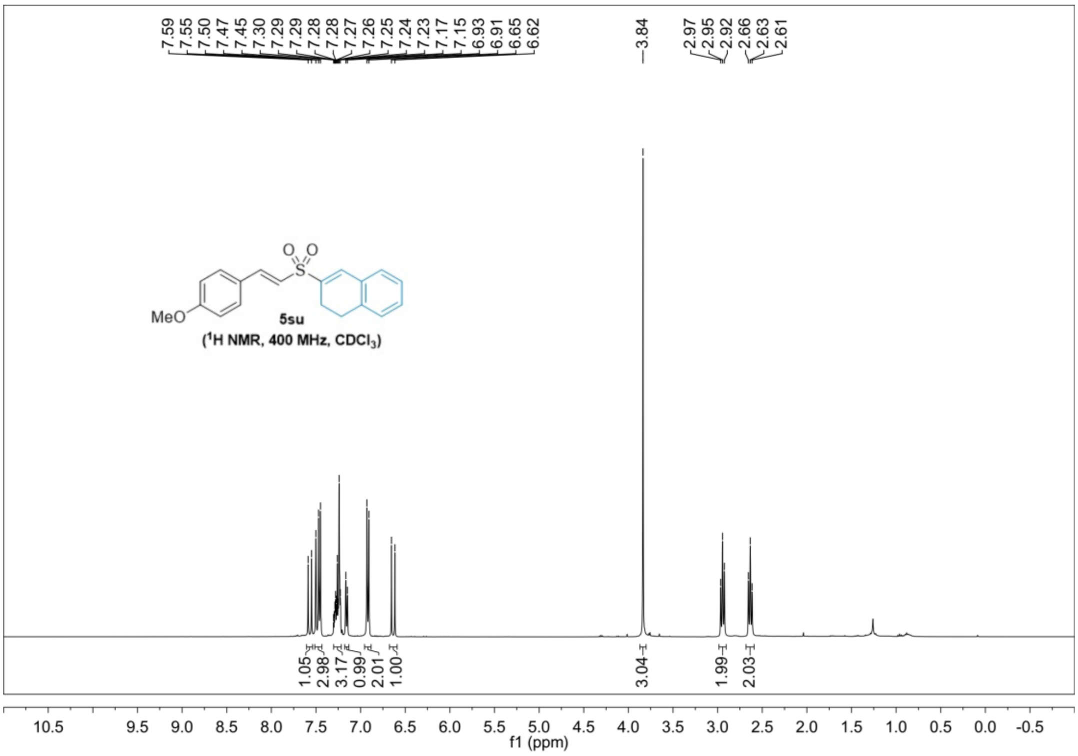
7.59
7.55
7.50
7.47
7.45
7.30
7.29
7.29
7.28
7.28
7.27
7.26
7.25
7.24
7.23
7.17
7.15
6.93
6.91
6.65
6.62
3.84
2.97
2.95
2.92
2.66
2.63
2.61
MeO
5su
(1H NMR, 400 MHz, CDCl3)
1.05
2.98
3.17
0.99
2.01
1.00
3.04
1.99
2.03
f1 (ppm)

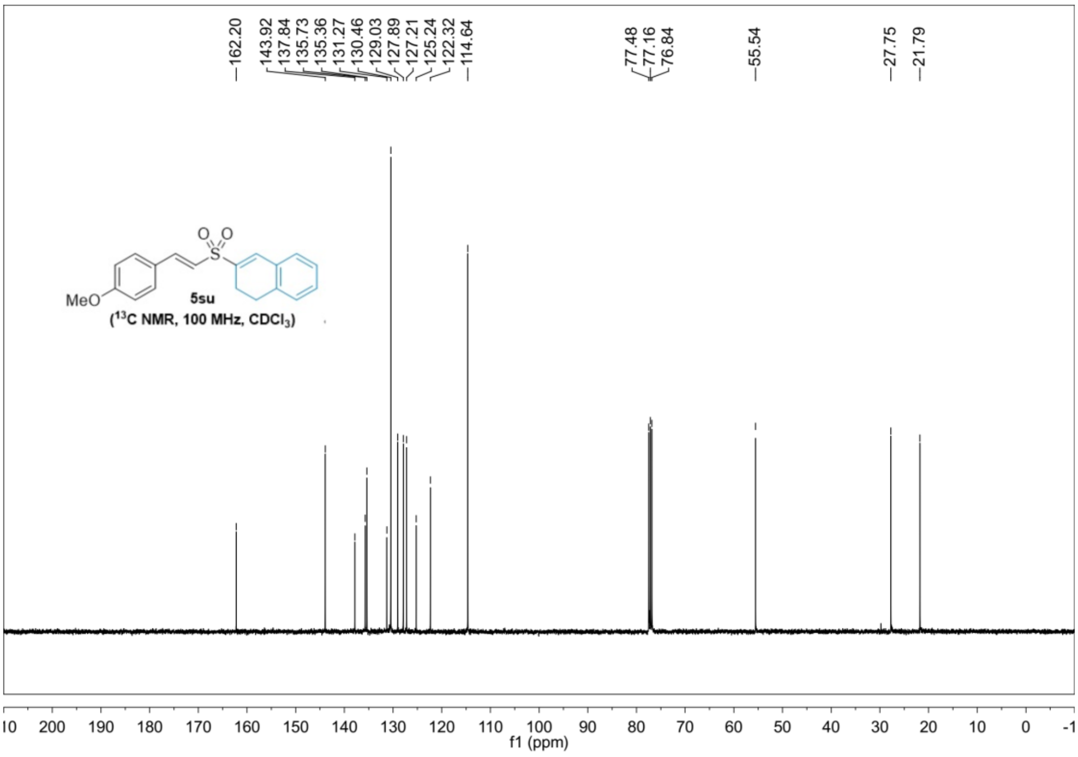
162.20
143.92
137.84
135.73
135.36
131.27
130.46
129.03
127.89
127.21
125.24
122.32
114.64
77.48
77.16
76.84
55.54
27.75
21.79
MeO
5su
(13C NMR, 100 MHz, CDCl3)
f1 (ppm)

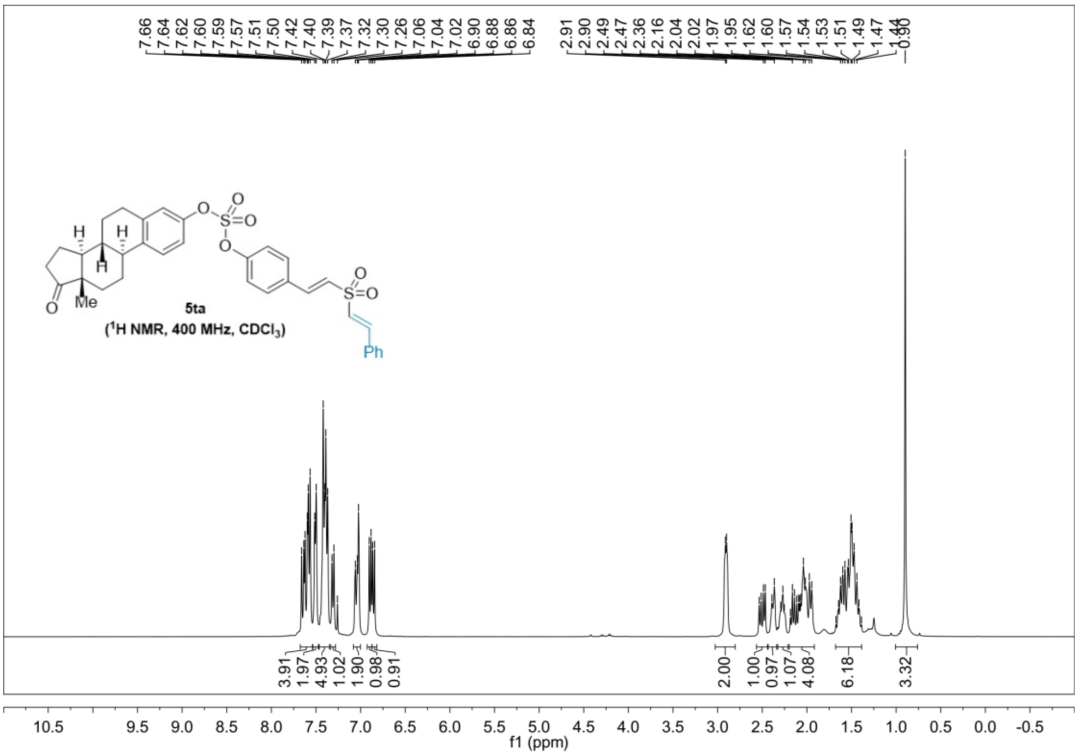
5ta
(¹H NMR, 400 MHz, CDCl₃)
Ph
f1 (ppm)

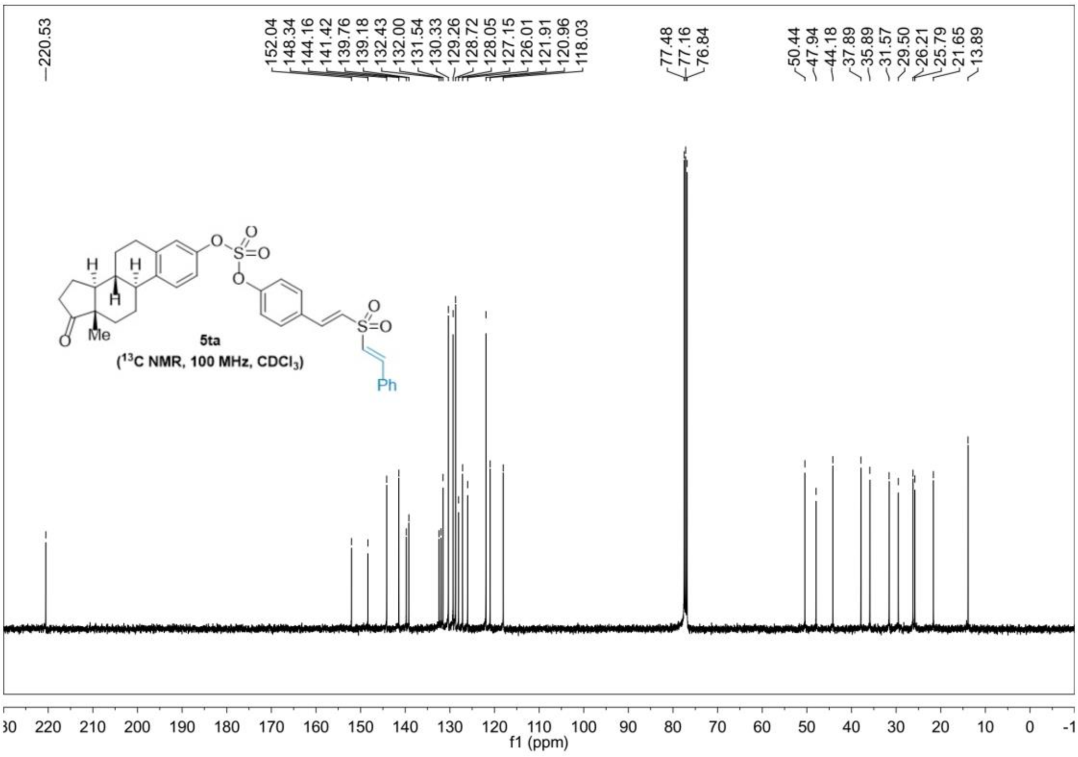
5ta
(¹³C NMR, 100 MHz, CDCl₃)
Ph
f1 (ppm)

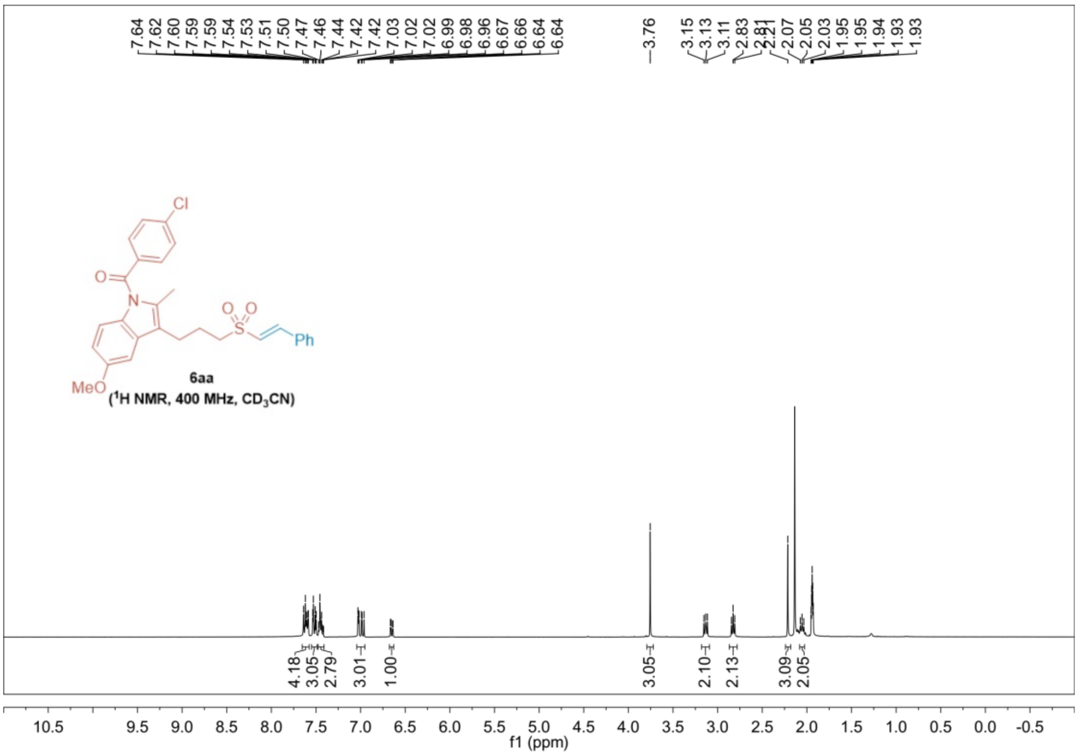
6aa
(1H NMR, 400 MHz, CD3CN)
f1 (ppm)

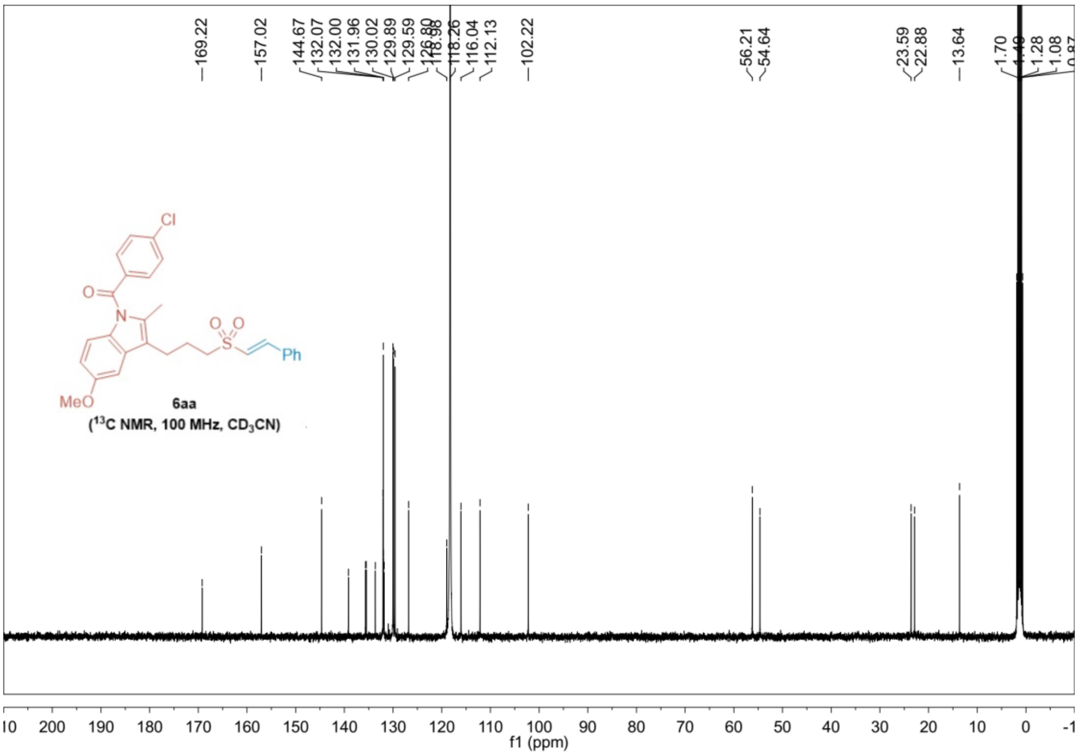
6aa
(13C NMR, 100 MHz, CD3CN)
f1 (ppm)

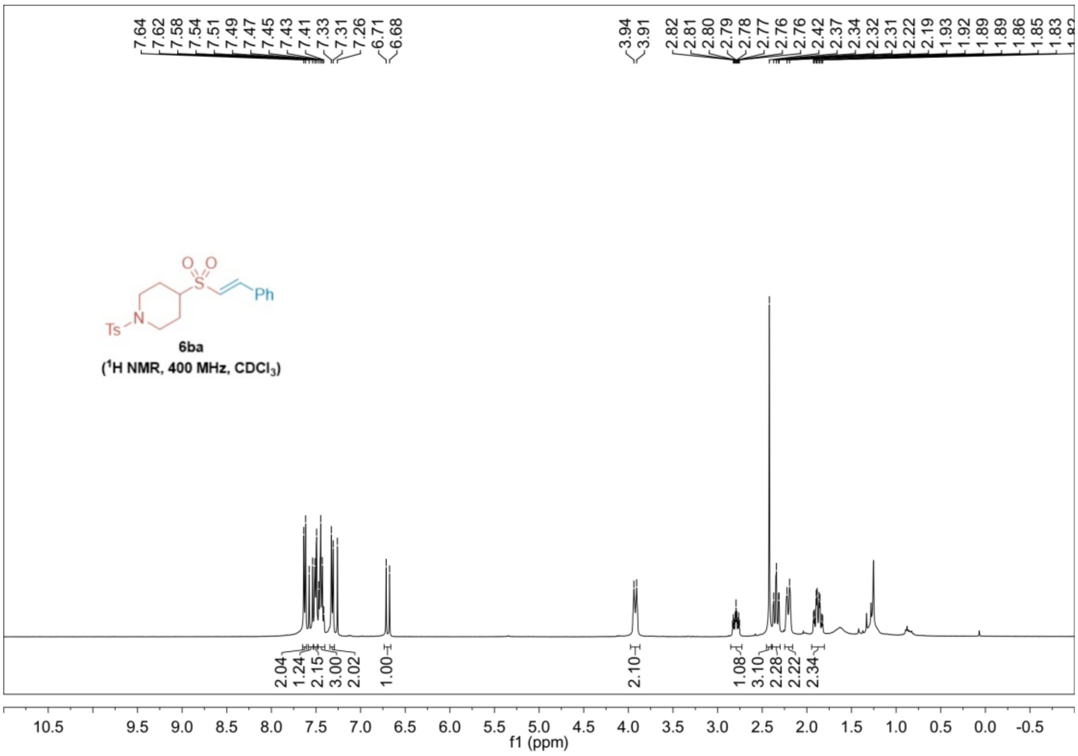
7.64
7.62
7.58
7.54
7.51
7.49
7.47
7.45
7.43
7.41
7.33
7.31
7.26
6.71
6.68
3.94
3.91
2.82
2.81
2.80
2.79
2.78
2.77
2.76
2.76
2.42
2.37
2.34
2.32
2.31
2.22
2.19
1.93
1.92
1.89
1.89
1.86
1.85
1.83
Ts
N
S
O
O
Ph
6ba
(1H NMR, 400 MHz, CDCl3)
2.04
1.24
2.15
3.00
2.02
1.00
2.10
1.08
3.10
2.28
2.22
2.34
10.5
9.5
9.0
8.5
8.0
7.5
7.0
6.5
6.0
5.5
5.0
4.5
4.0
3.5
3.0
2.5
2.0
1.5
1.0
0.5
0.0
-0.5
f1 (ppm)

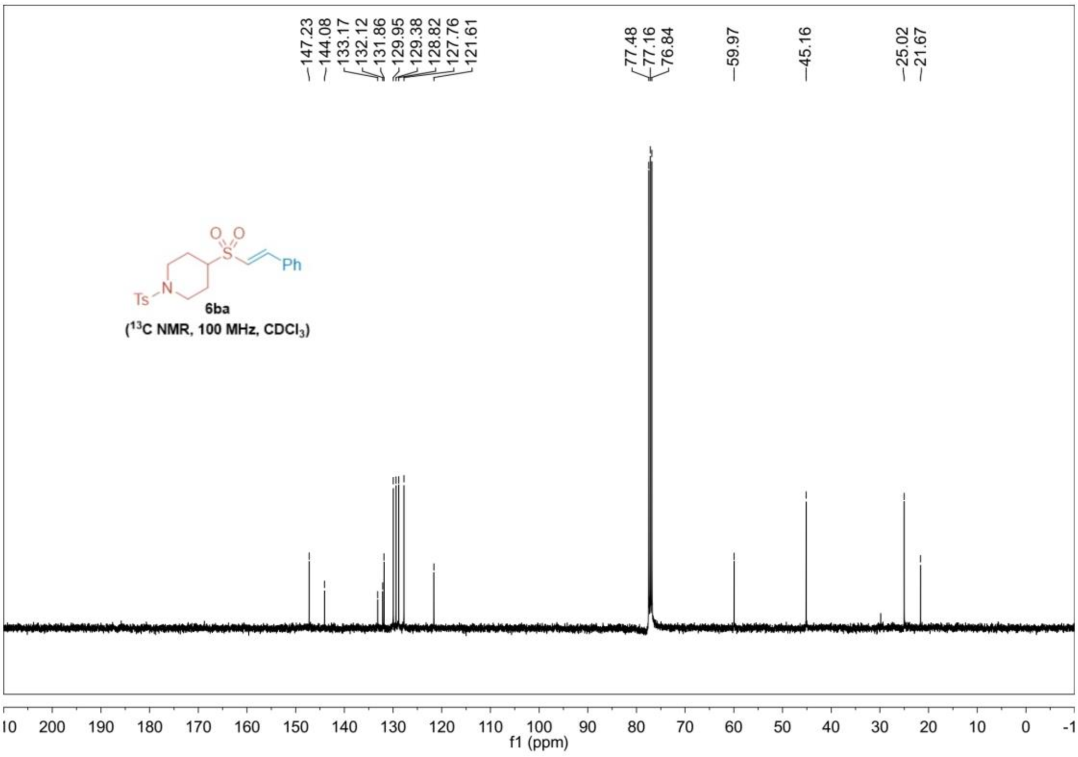
147.23
144.08
133.17
132.12
131.86
129.95
129.38
128.82
127.76
121.61
77.48
77.16
76.84
59.97
45.16
25.02
21.67
Ts
N
S
O
O
Ph
6ba
(13C NMR, 100 MHz, CDCl3)
200
190
180
170
160
150
140
130
120
110
100
90
80
70
60
50
40
30
20
10
0
f1 (ppm)

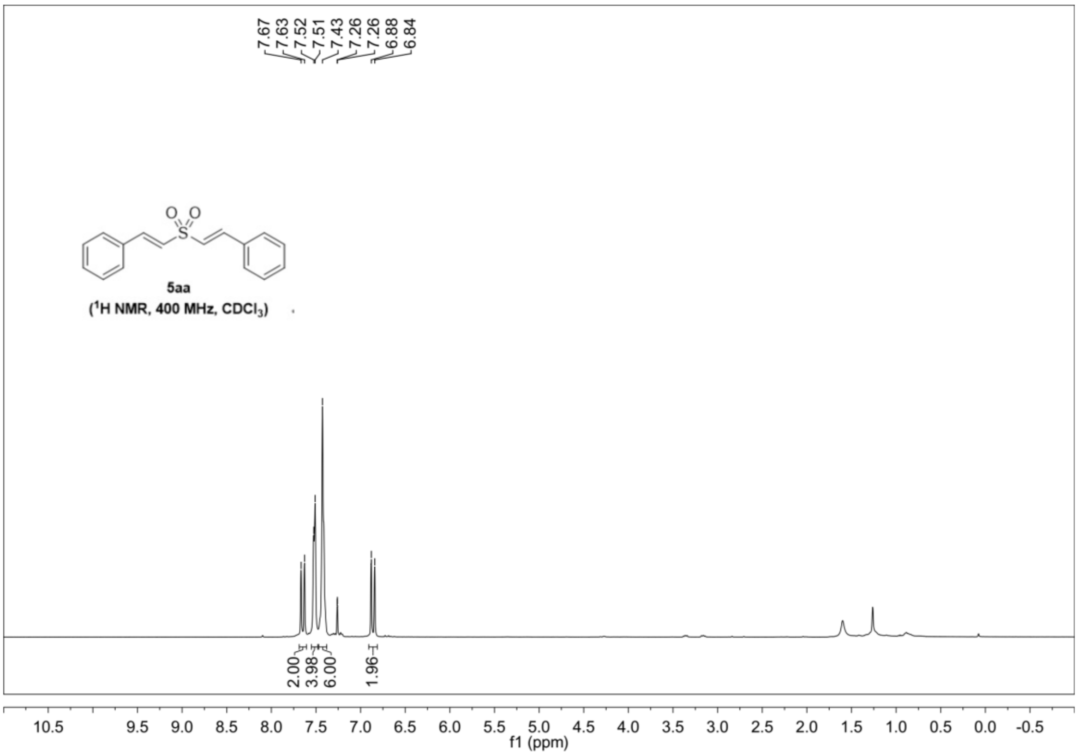
7.67
7.63
7.52
7.51
7.43
7.26
7.26
6.88
6.84
5aa
(^{1}H NMR, 400 MHz, CDCl$_3$)
2.00
3.98
6.00
1.96
10.5 9.5 9.0 8.5 8.0 7.5 7.0 6.5 6.0 5.5 5.0 4.5 4.0 3.5 3.0 2.5 2.0 1.5 1.0 0.5 0.0 -0.5
f1 (ppm)

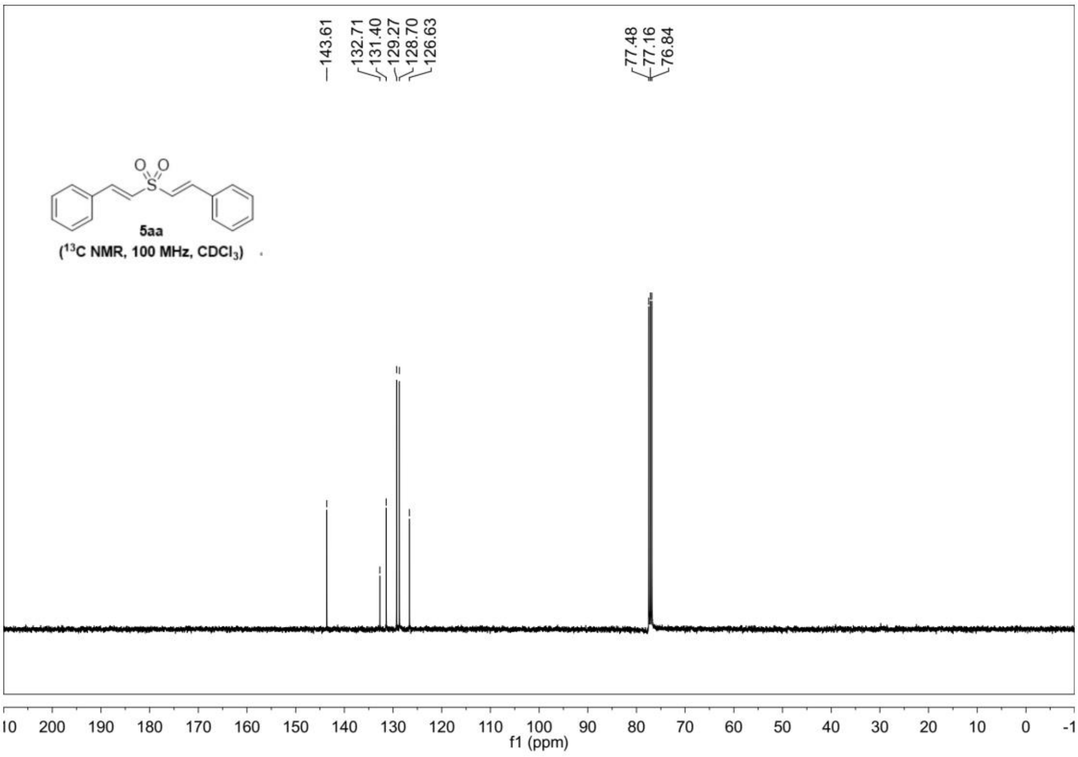
143.61
132.71
131.40
129.27
128.70
126.63
77.48
77.16
76.84
5aa
(^{13}C NMR, 100 MHz, CDCl$_3$)
200 190 180 170 160 150 140 130 120 110 100 90 80 70 60 50 40 30 20 10 0
f1 (ppm)

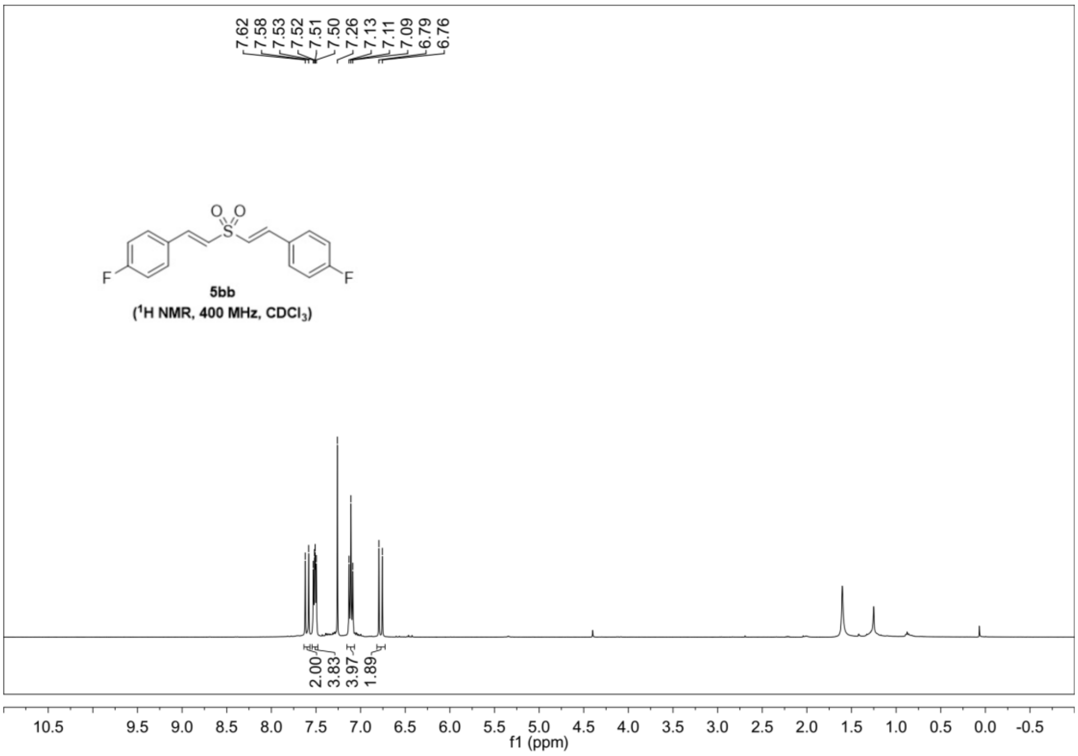
7.62
7.58
7.53
7.52
7.51
7.50
7.26
7.13
7.11
7.09
6.79
6.76
F
O O
S
F
5bb
(1H NMR, 400 MHz, CDCl3)
2.00
3.83
3.97
1.89
10.5 9.5 9.0 8.5 8.0 7.5 7.0 6.5 6.0 5.5 5.0 4.5 4.0 3.5 3.0 2.5 2.0 1.5 1.0 0.5 0.0 -0.5
f1 (ppm)

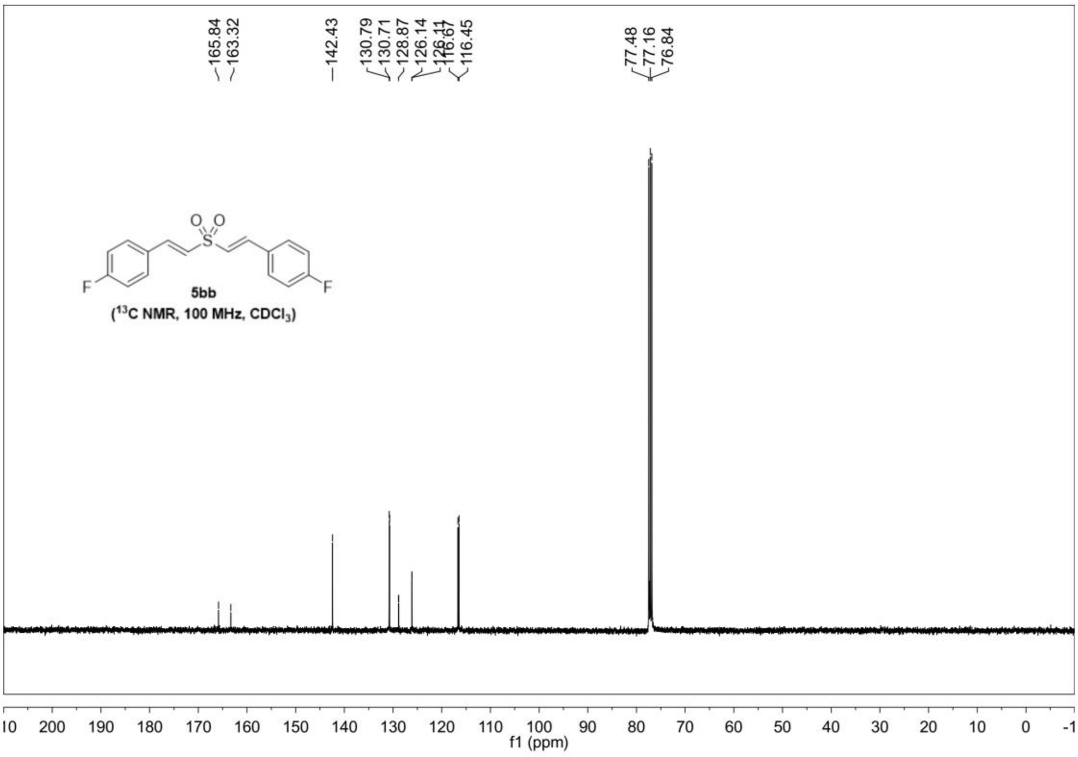
165.84
163.32
142.43
130.79
130.71
128.87
126.14
126.11
116.67
116.45
77.48
77.16
76.84
F
O O
S
F
5bb
(13C NMR, 100 MHz, CDCl3)
200 190 180 170 160 150 140 130 120 110 100 90 80 70 60 50 40 30 20 10 0
f1 (ppm)

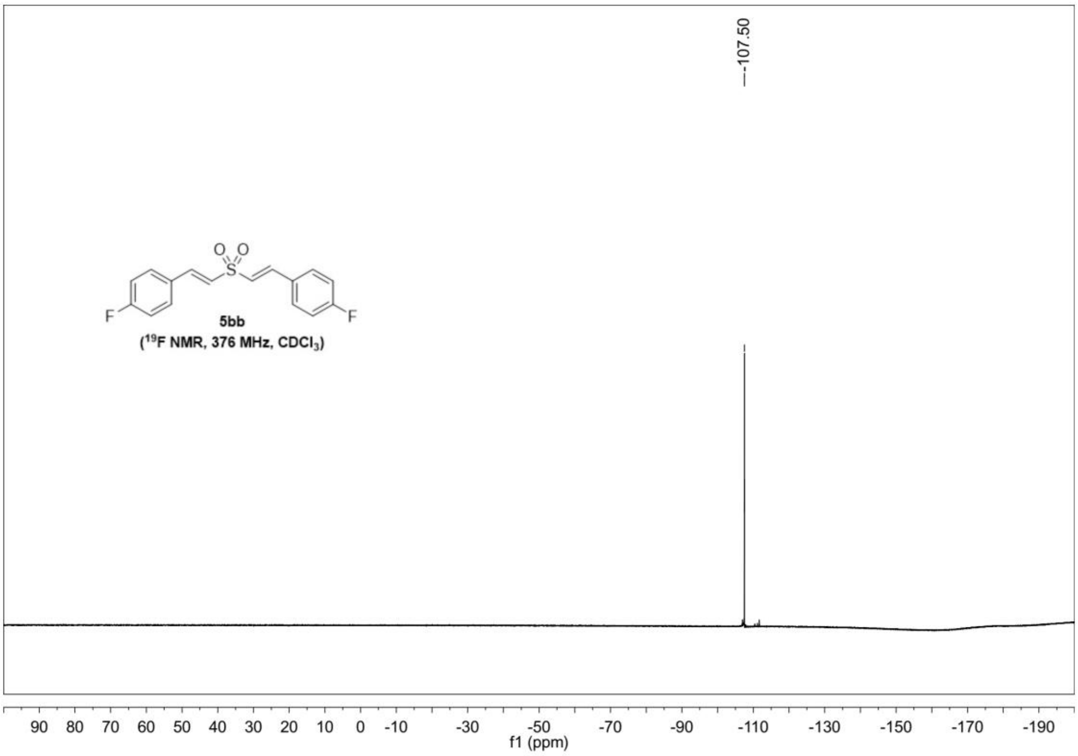
-107.50
5bb
(19F NMR, 376 MHz, CDCl3)
90 80 70 60 50 40 30 20 10 0 -10 -30 -50 -70 -90 -110 -130 -150 -170 -190
f1 (ppm)

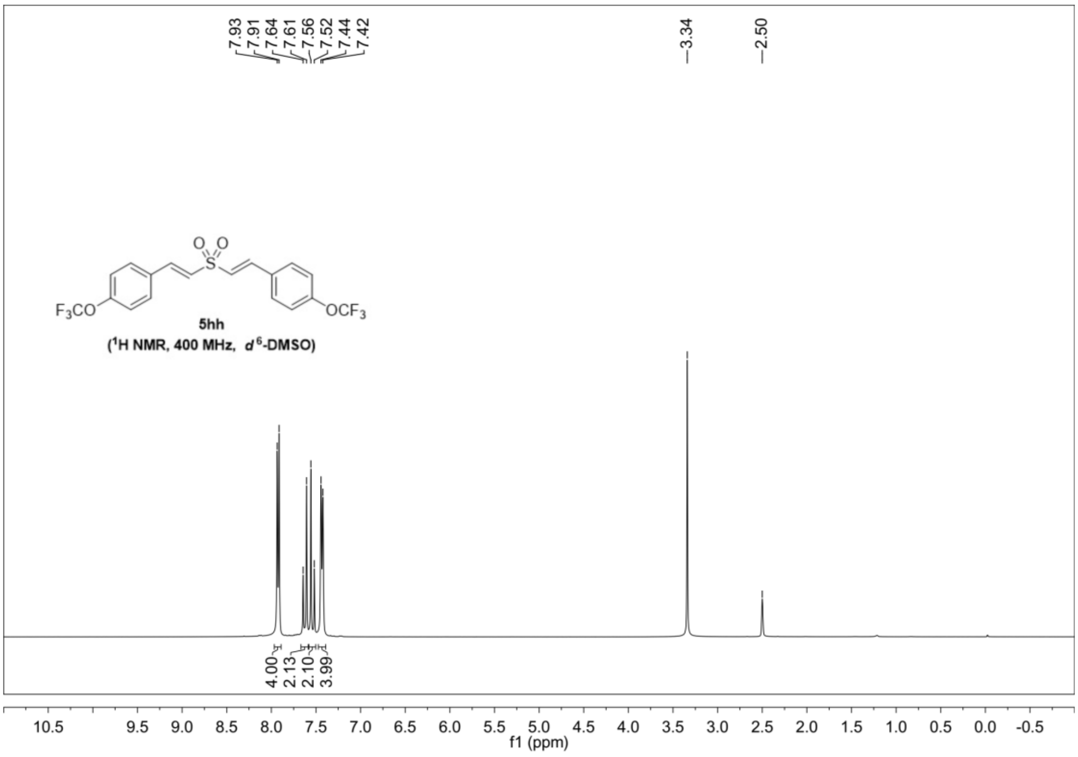
7.93
7.91
7.64
7.61
7.56
7.52
7.44
7.42
3.34
2.50
F3CO
OCF3
5hh
(1H NMR, 400 MHz, d6-DMSO)
4.00
2.13
2.10
3.99
10.5 9.5 9.0 8.5 8.0 7.5 7.0 6.5 6.0 5.5 5.0 4.5 4.0 3.5 3.0 2.5 2.0 1.5 1.0 0.5 0.0 -0.5
f1 (ppm)

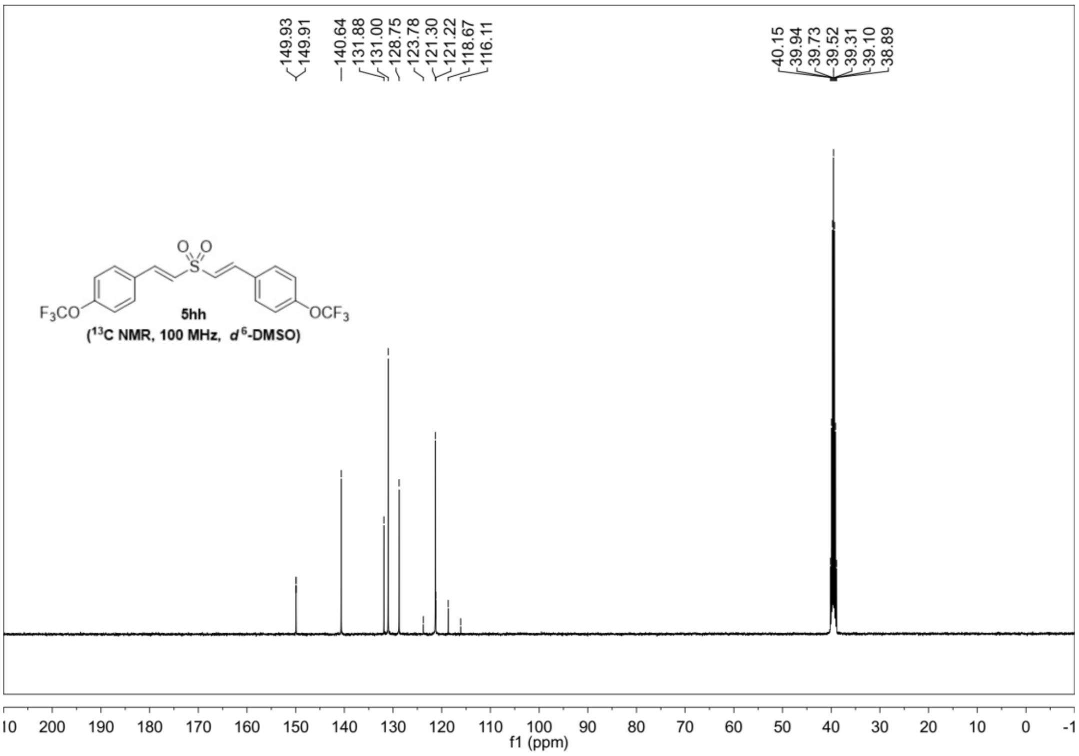
149.93
149.91
140.64
131.88
131.00
128.75
123.78
121.30
121.22
118.67
116.11
40.15
39.94
39.73
39.52
39.31
39.10
38.89
F3CO
OCF3
5hh
(13C NMR, 100 MHz, d6-DMSO)
f1 (ppm)

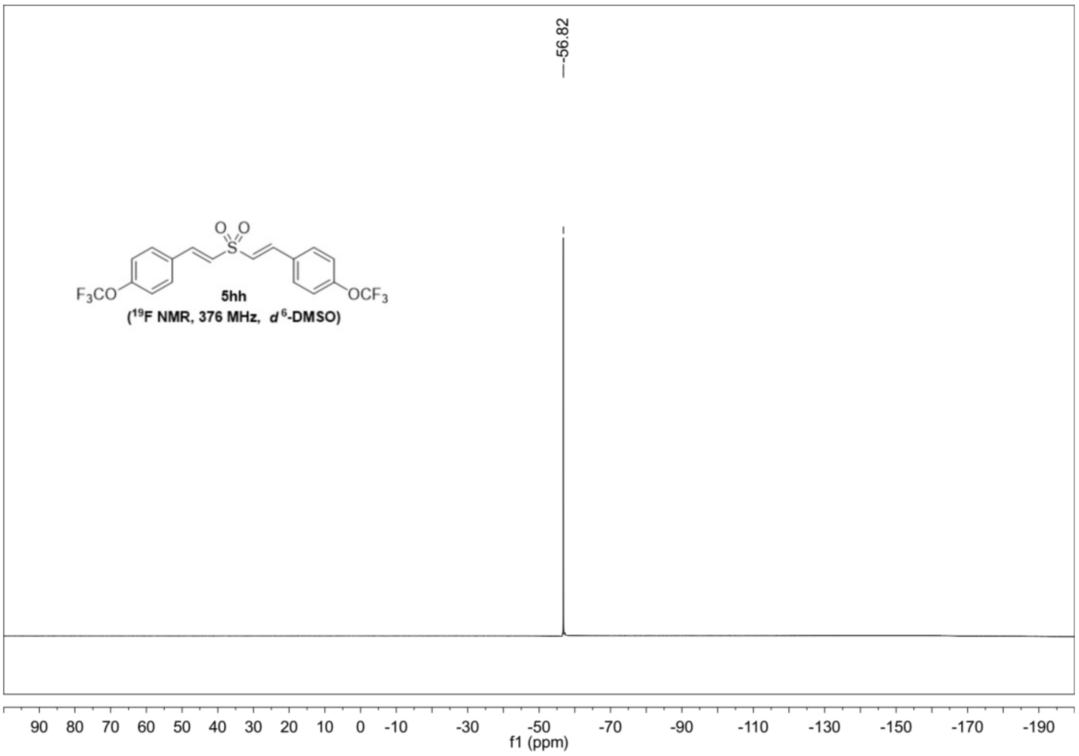
-56.82
F3CO
OCF3
5hh
(19F NMR, 376 MHz, d6-DMSO)
f1 (ppm)

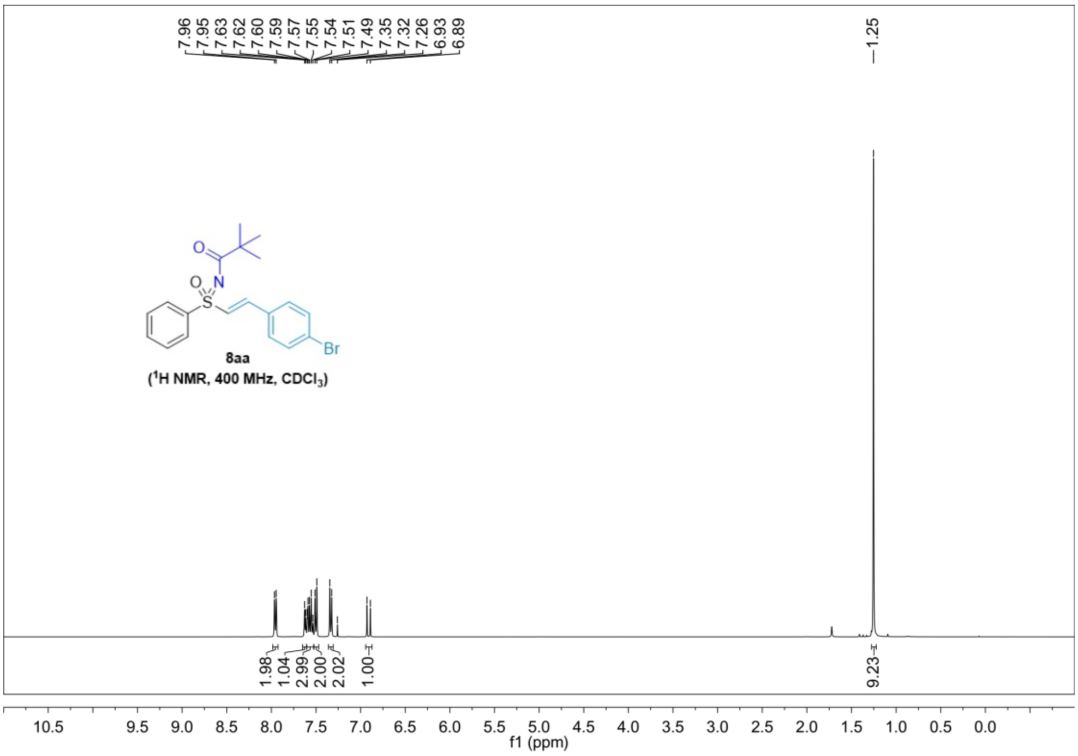
7.96
7.95
7.63
7.62
7.60
7.59
7.57
7.55
7.54
7.51
7.49
7.35
7.32
7.26
6.93
6.89
1.25
O
N
O
S
Br
8aa
(1H NMR, 400 MHz, CDCl3)
1.98
1.04
2.99
2.00
2.02
1.00
9.23
10.5
9.5
9.0
8.5
8.0
7.5
7.0
6.5
6.0
5.5
5.0
4.5
4.0
3.5
3.0
2.5
2.0
1.5
1.0
0.5
0.0
f1 (ppm)

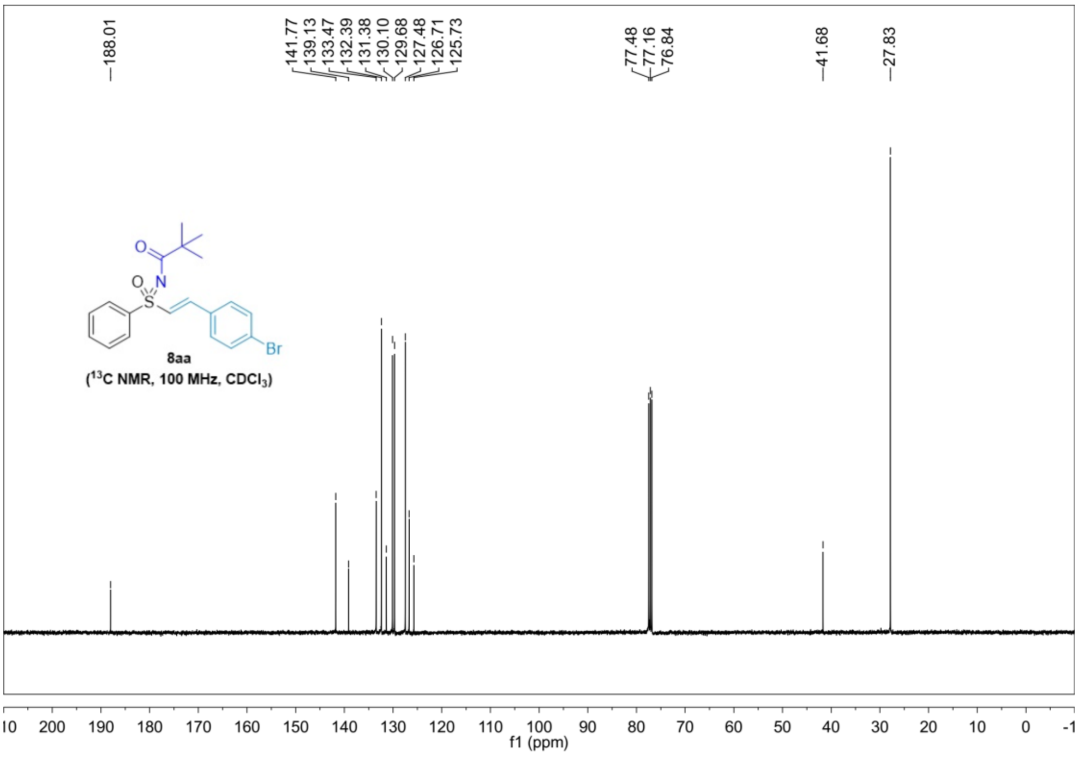
188.01
141.77
139.13
133.47
132.39
131.38
130.10
129.68
127.48
126.71
125.73
77.48
77.16
76.84
41.68
27.83
O
N
O
S
Br
8aa
(13C NMR, 100 MHz, CDCl3)
200
190
180
170
160
150
140
130
120
110
100
90
80
70
60
50
40
30
20
10
0
f1 (ppm)

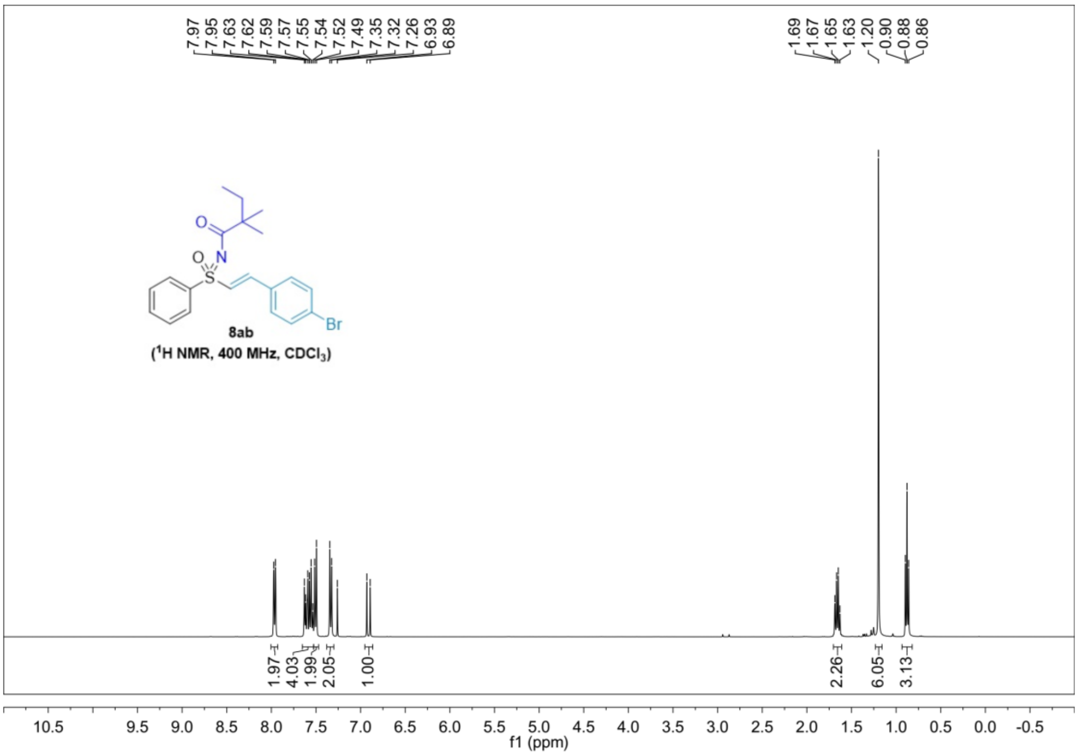
7.97
7.95
7.63
7.62
7.59
7.57
7.55
7.54
7.52
7.49
7.35
7.32
7.26
6.93
6.89
1.69
1.67
1.65
1.63
1.20
0.90
0.88
0.86
O
N
S
Br
8ab
(1H NMR, 400 MHz, CDCl3)
1.97
4.03
1.99
2.05
1.00
2.26
6.05
3.13
10.5 9.5 9.0 8.5 8.0 7.5 7.0 6.5 6.0 5.5 5.0 4.5 4.0 3.5 3.0 2.5 2.0 1.5 1.0 0.5 0.0 -0.5
f1 (ppm)

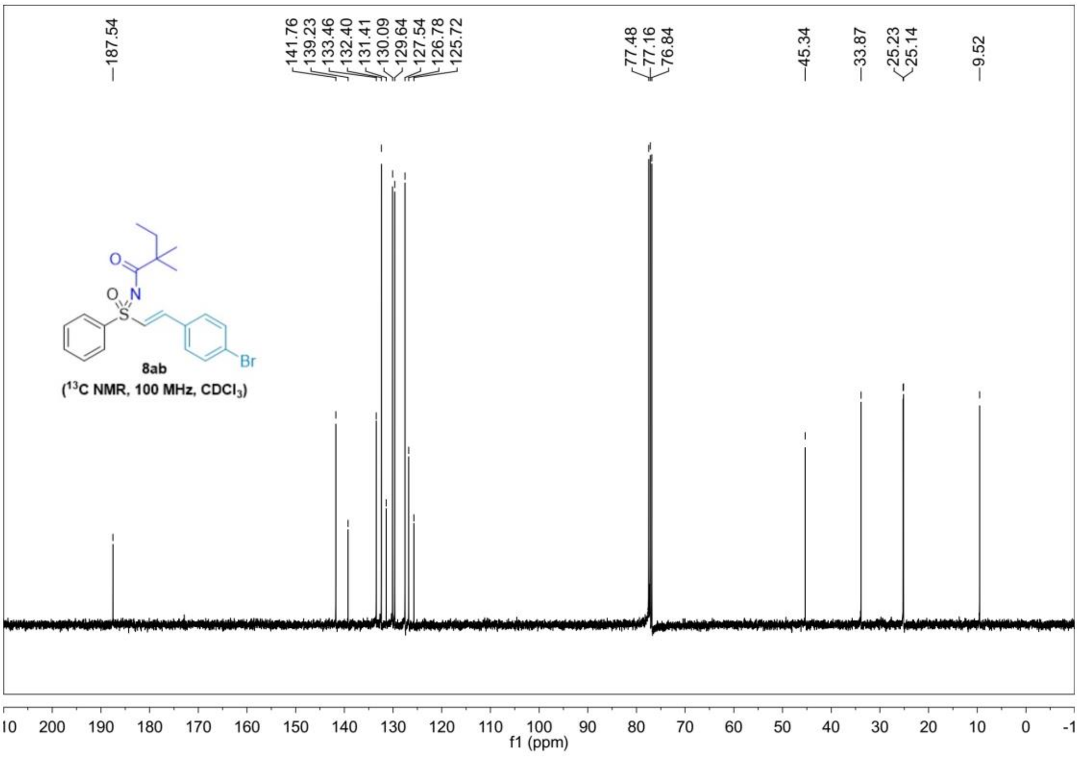
187.54
141.76
139.23
133.46
132.40
131.41
130.09
129.64
127.54
126.78
125.72
77.48
77.16
76.84
45.34
33.87
25.23
25.14
9.52
O
N
S
Br
8ab
(13C NMR, 100 MHz, CDCl3)
10 200 190 180 170 160 150 140 130 120 110 100 90 80 70 60 50 40 30 20 10 0 -1
f1 (ppm)

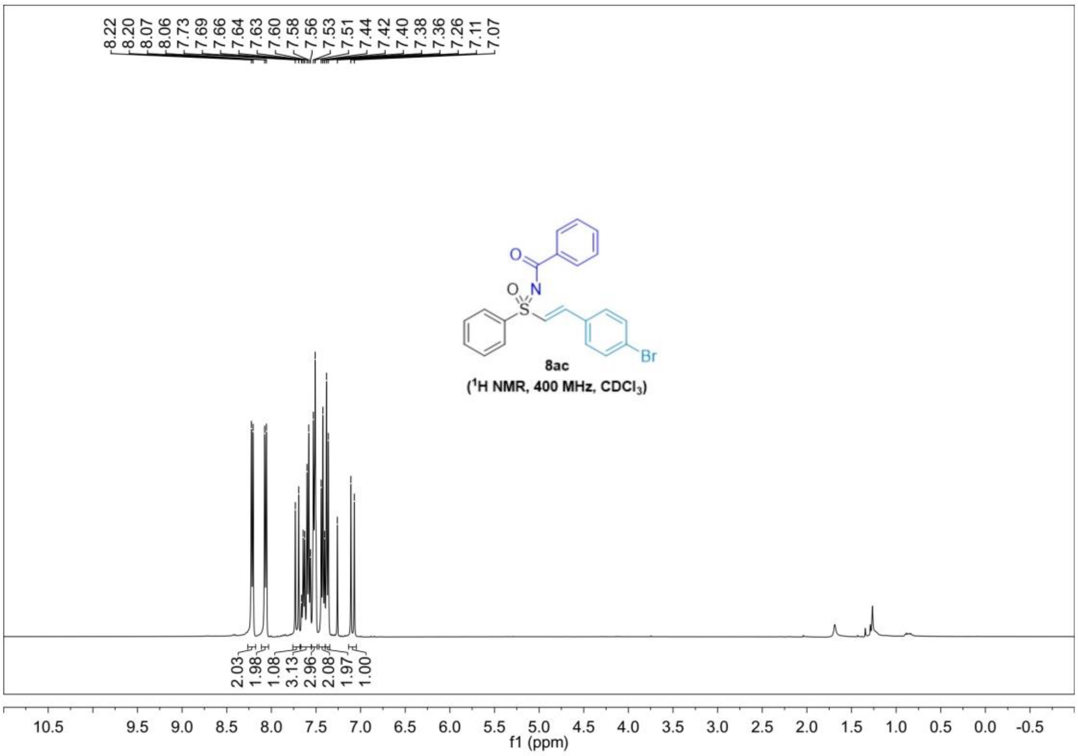
8.22
8.20
8.07
8.06
7.73
7.69
7.66
7.64
7.63
7.60
7.58
7.56
7.53
7.51
7.44
7.42
7.40
7.38
7.36
7.26
7.11
7.07
O
N
O
S
Br
8ac
(^{1}H NMR, 400 MHz, CDCl$_3$)
2.03
1.98
1.08
3.13
2.96
2.08
1.97
1.00
10.5 9.5 9.0 8.5 8.0 7.5 7.0 6.5 6.0 5.5 5.0 4.5 4.0 3.5 3.0 2.5 2.0 1.5 1.0 0.5 0.0 -0.5
f1 (ppm)

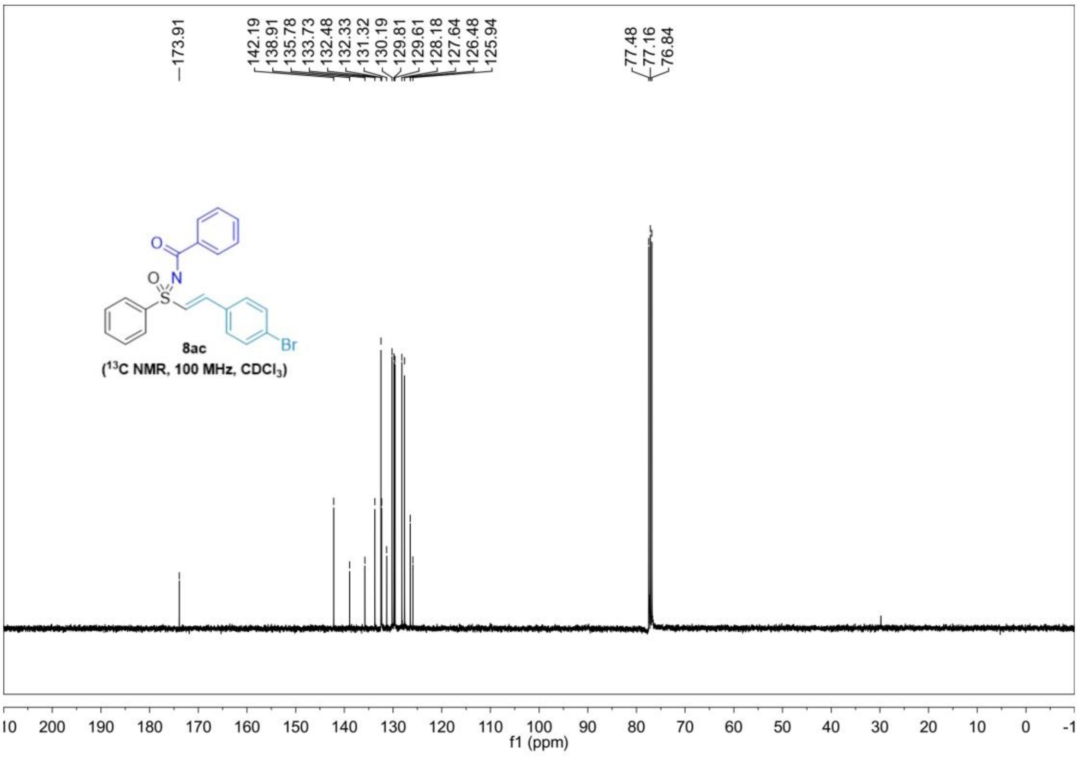
173.91
142.19
138.91
135.78
133.73
132.48
132.33
131.32
130.19
129.81
129.61
128.18
127.64
126.48
125.94
77.48
77.16
76.84
O
N
O
S
Br
8ac
(^{13}C NMR, 100 MHz, CDCl$_3$)
200 190 180 170 160 150 140 130 120 110 100 90 80 70 60 50 40 30 20 10 0
f1 (ppm)

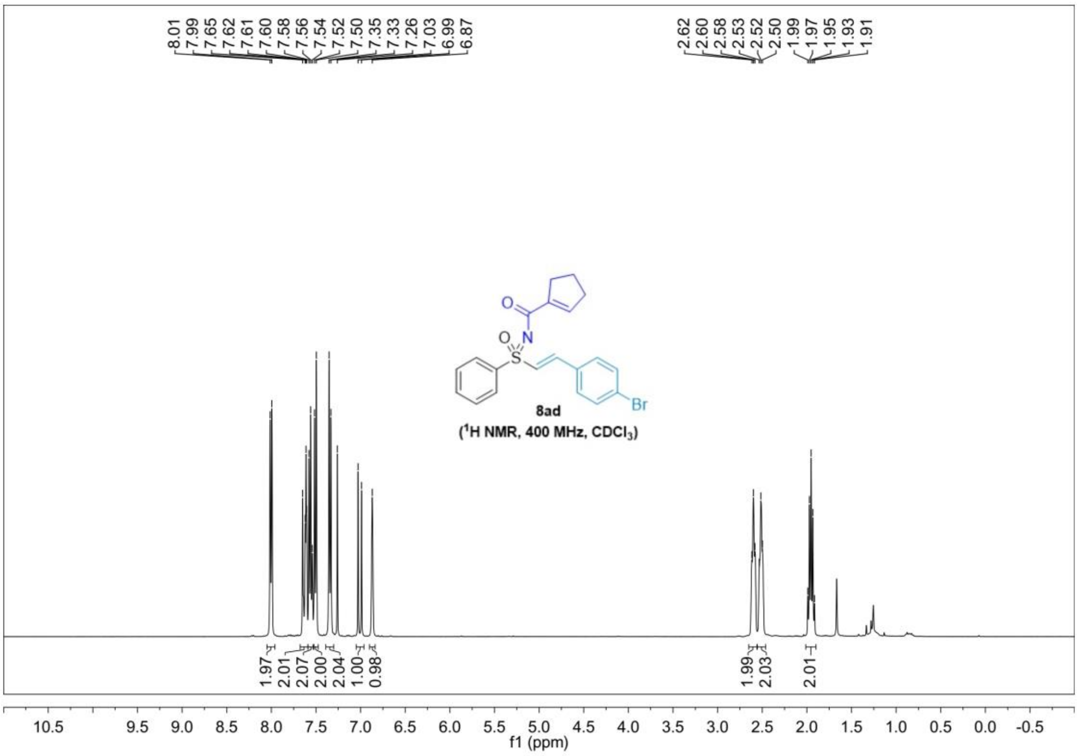
8.01
7.99
7.65
7.62
7.61
7.60
7.58
7.56
7.54
7.52
7.50
7.35
7.33
7.26
7.03
6.99
6.87
2.62
2.60
2.58
2.53
2.52
2.50
1.99
1.97
1.95
1.93
1.91
8ad
(^{1}H NMR, 400 MHz, CDCl$_3$)
1.97
2.01
2.07
2.00
2.04
1.00
0.98
1.99
2.03
2.01
f1 (ppm)

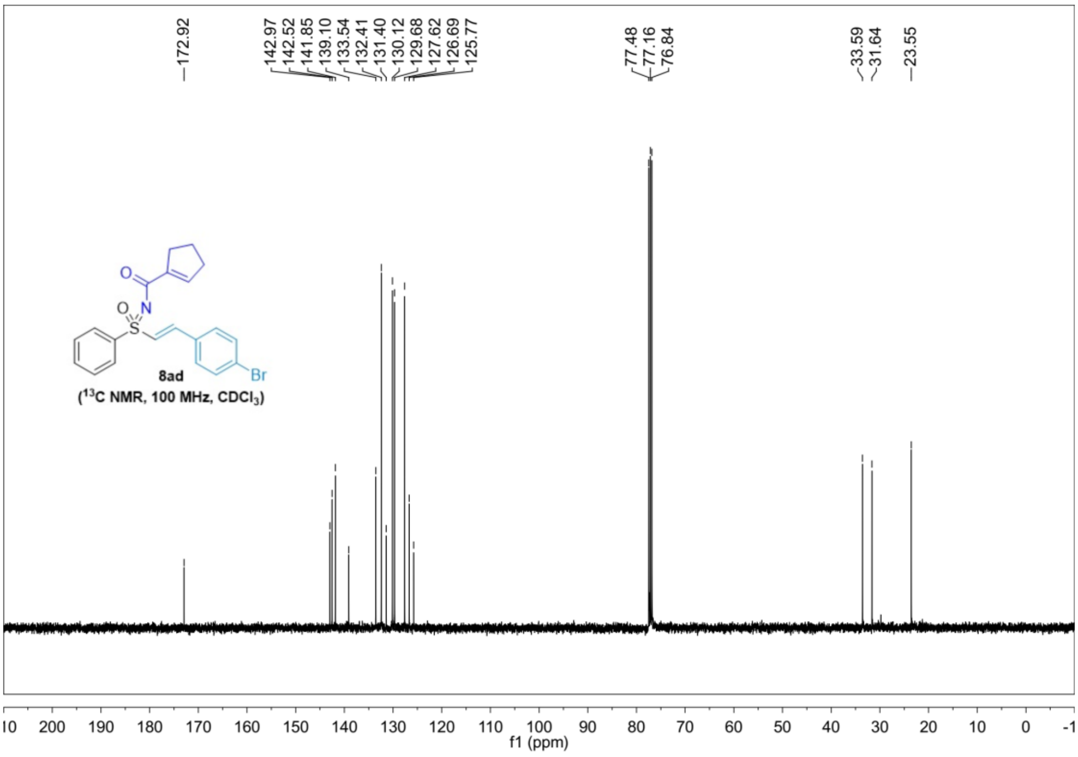
172.92
142.97
142.52
141.85
139.10
133.54
132.41
131.40
130.12
129.68
127.62
126.69
125.77
77.48
77.16
76.84
33.59
31.64
23.55
8ad
(^{13}C NMR, 100 MHz, CDCl$_3$)
f1 (ppm)

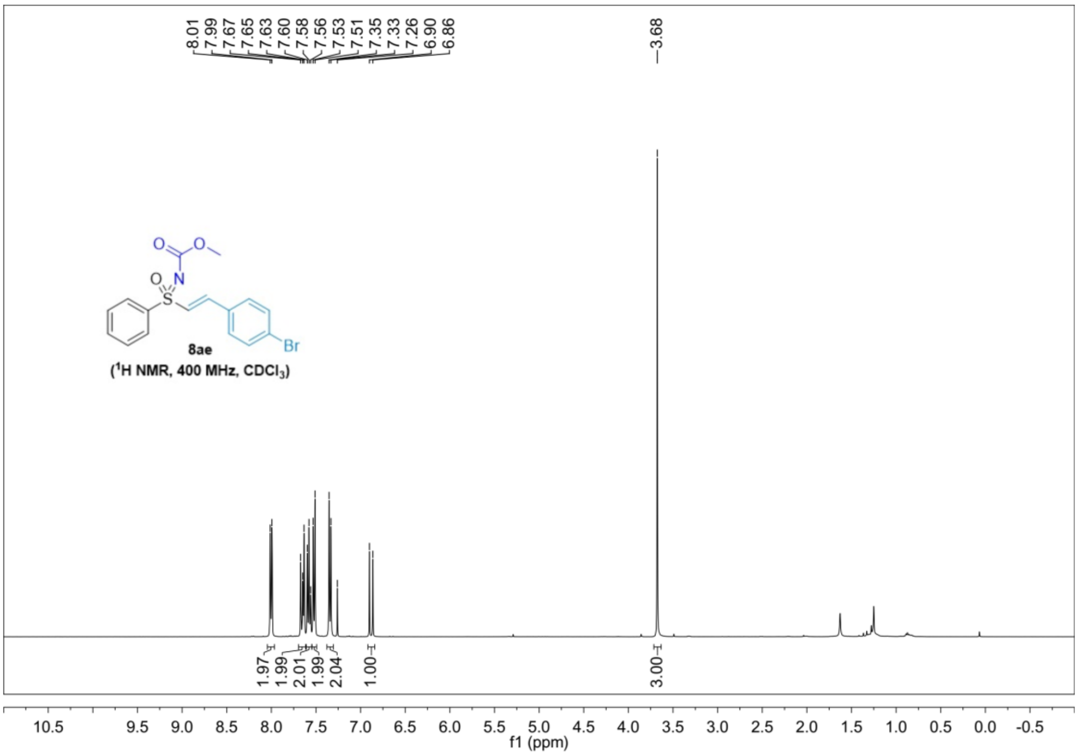
8.01
7.99
7.67
7.65
7.63
7.60
7.58
7.56
7.53
7.51
7.35
7.33
7.26
6.90
6.86
3.68
8ae
(¹H NMR, 400 MHz, CDCl3)
Br
1.97
1.99
2.01
1.99
2.04
1.00
3.00
f1 (ppm)

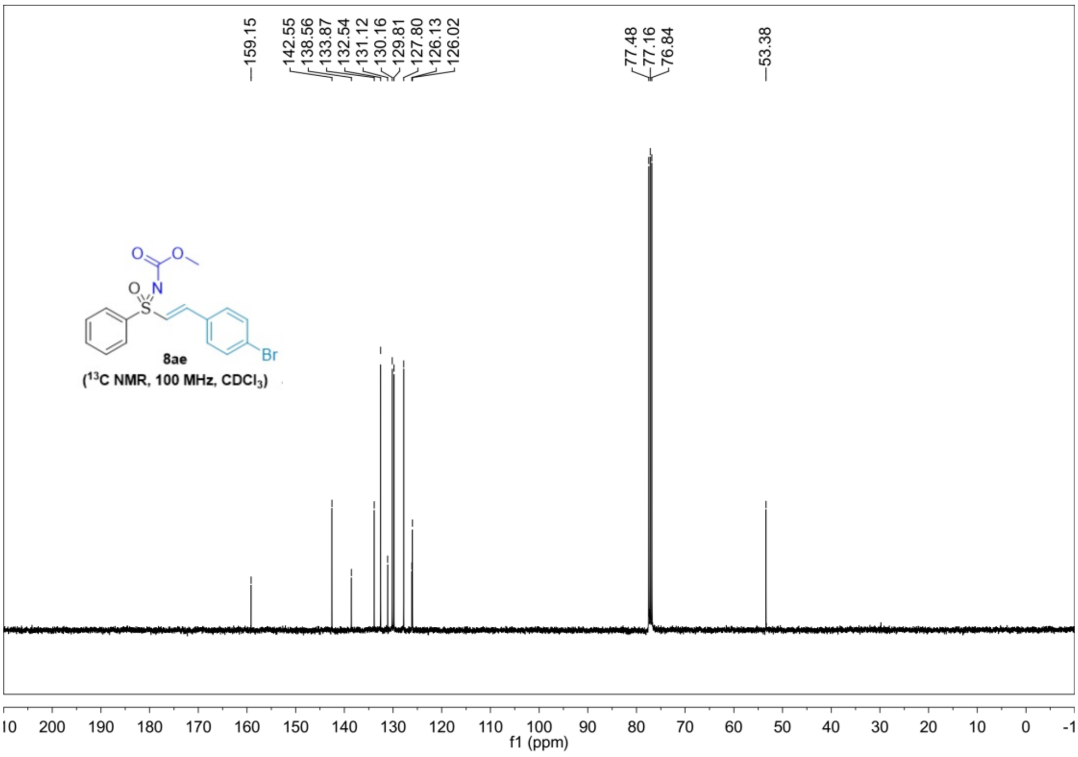
159.15
142.55
138.56
133.87
132.54
131.12
130.16
129.81
127.80
126.13
126.02
77.48
77.16
76.84
53.38
8ae
(¹³C NMR, 100 MHz, CDCl3)
Br
f1 (ppm)

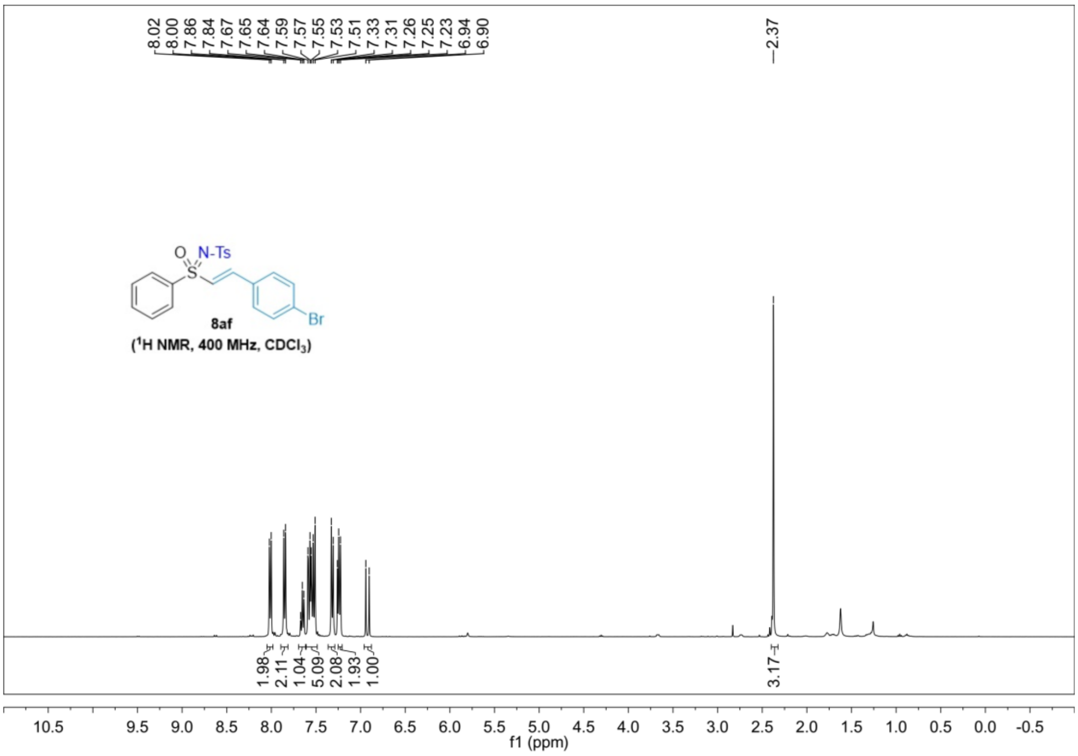

8.02
8.00
7.86
7.84
7.67
7.65
7.64
7.59
7.57
7.55
7.53
7.51
7.33
7.31
7.26
7.25
7.23
6.94
6.90
2.37
O
N-Ts
S
Br
8af
(1H NMR, 400 MHz, CDCl3)
1.98
2.11
1.04
5.09
2.08
1.93
1.00
3.17
f1 (ppm)

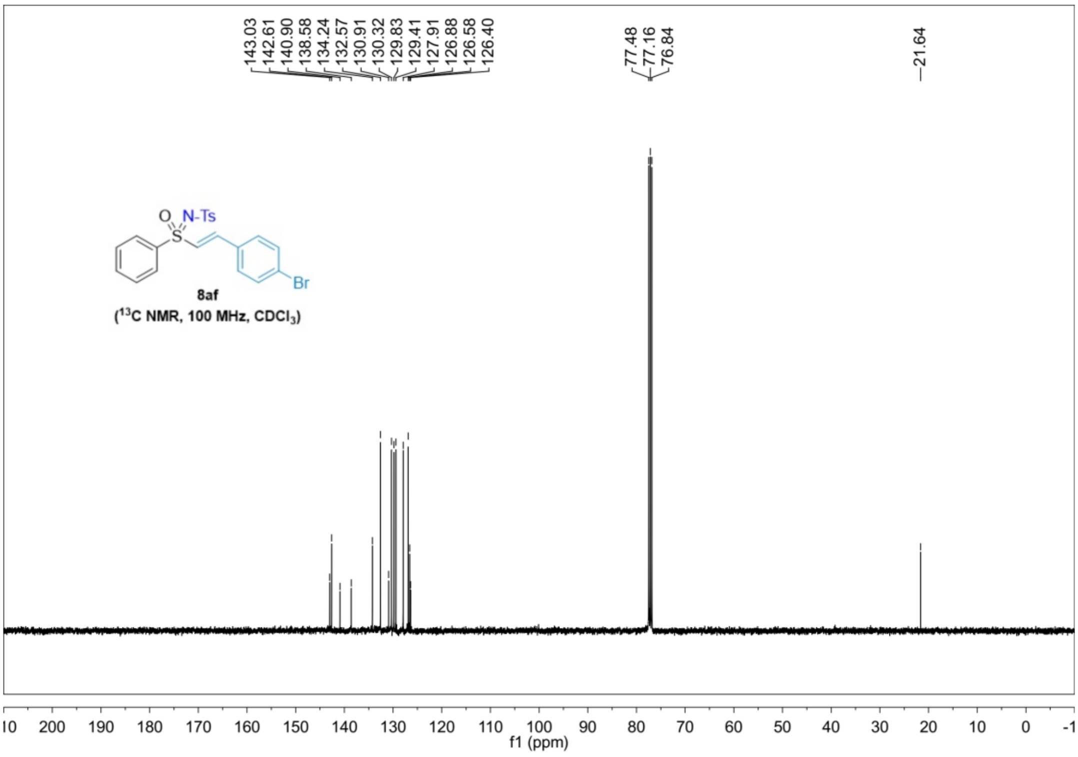

143.03
142.61
140.90
138.58
134.24
132.57
130.91
130.32
129.83
129.41
127.91
126.88
126.58
126.40
77.48
77.16
76.84
21.64
O
N-Ts
S
Br
8af
(13C NMR, 100 MHz, CDCl3)
f1 (ppm)

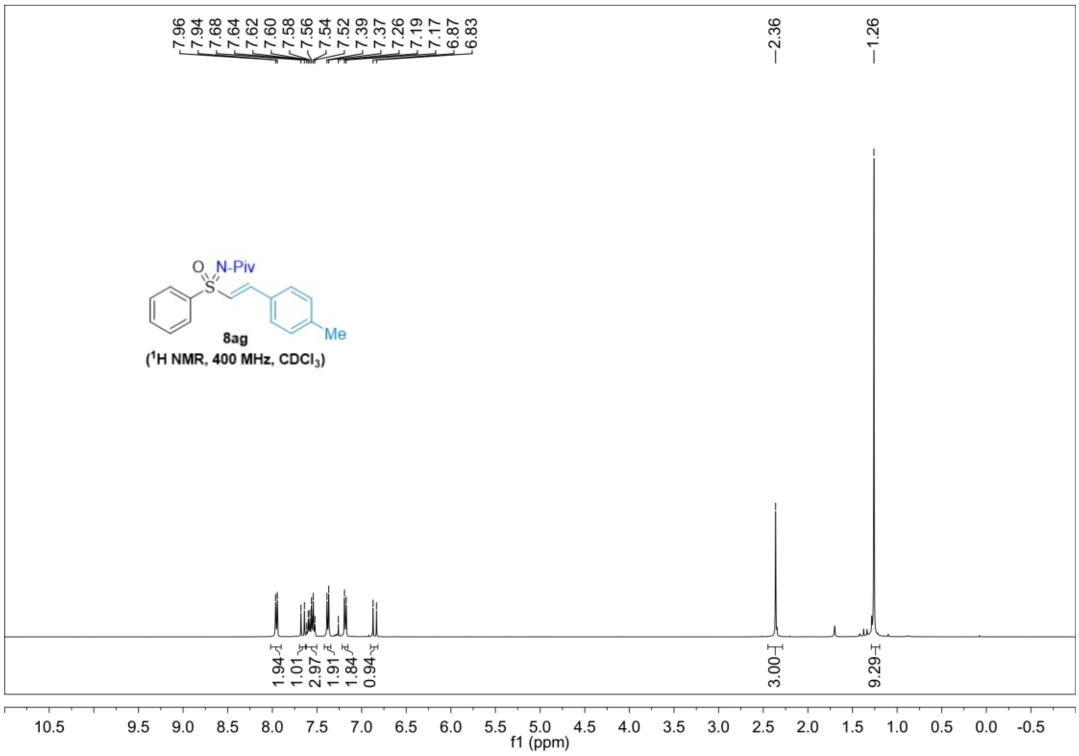

7.96 7.94 7.68 7.64 7.62 7.60 7.58 7.56 7.54 7.52 7.39 7.37 7.26 7.19 7.17 6.87 6.83
2.36
1.26
N-Piv
Me
8ag
(1H NMR, 400 MHz, CDCl3)
1.94 1.01 2.97 1.91 1.84 0.94
3.00
9.29
f1 (ppm)

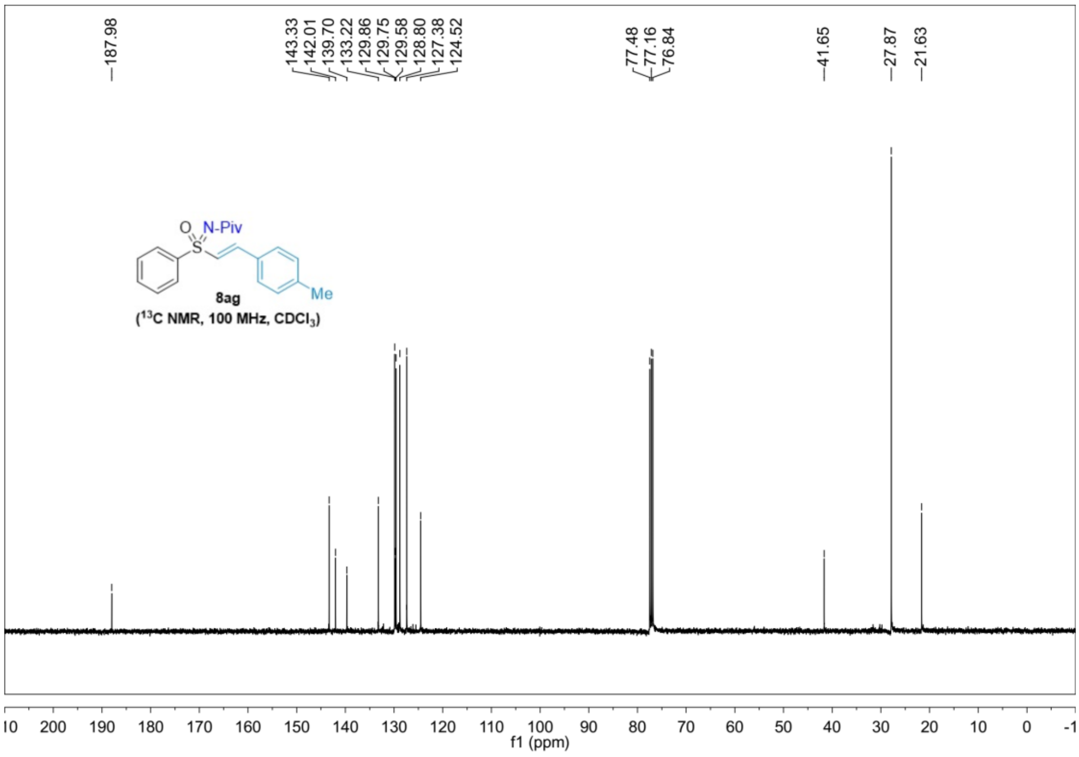

187.98
143.33 142.01 139.70 133.22 129.86 129.75 129.58 128.80 127.38 124.52
77.48 77.16 76.84
41.65
27.87 21.63
N-Piv
Me
8ag
(13C NMR, 100 MHz, CDCl3)
f1 (ppm)

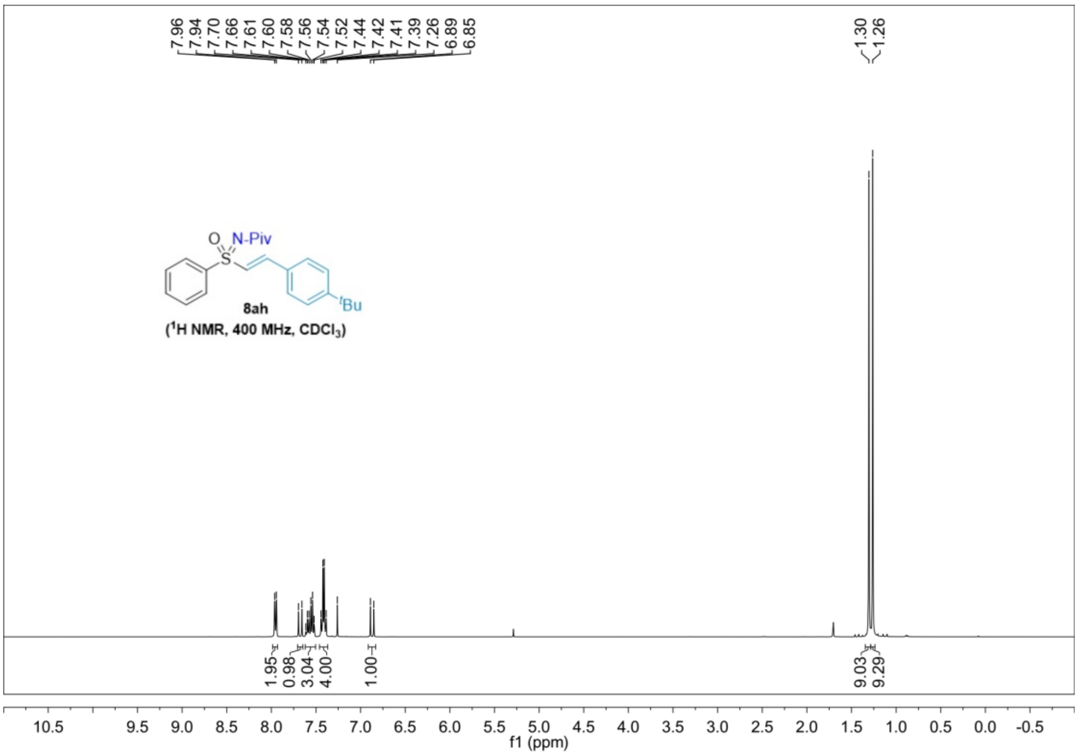
7.96
7.94
7.70
7.66
7.61
7.60
7.58
7.56
7.54
7.52
7.44
7.42
7.41
7.39
7.26
6.89
6.85
1.30
1.26
N-Piv
8ah
(¹H NMR, 400 MHz, CDCl₃)
1.95
0.98
3.04
4.00
1.00
9.03
9.29
f1 (ppm)

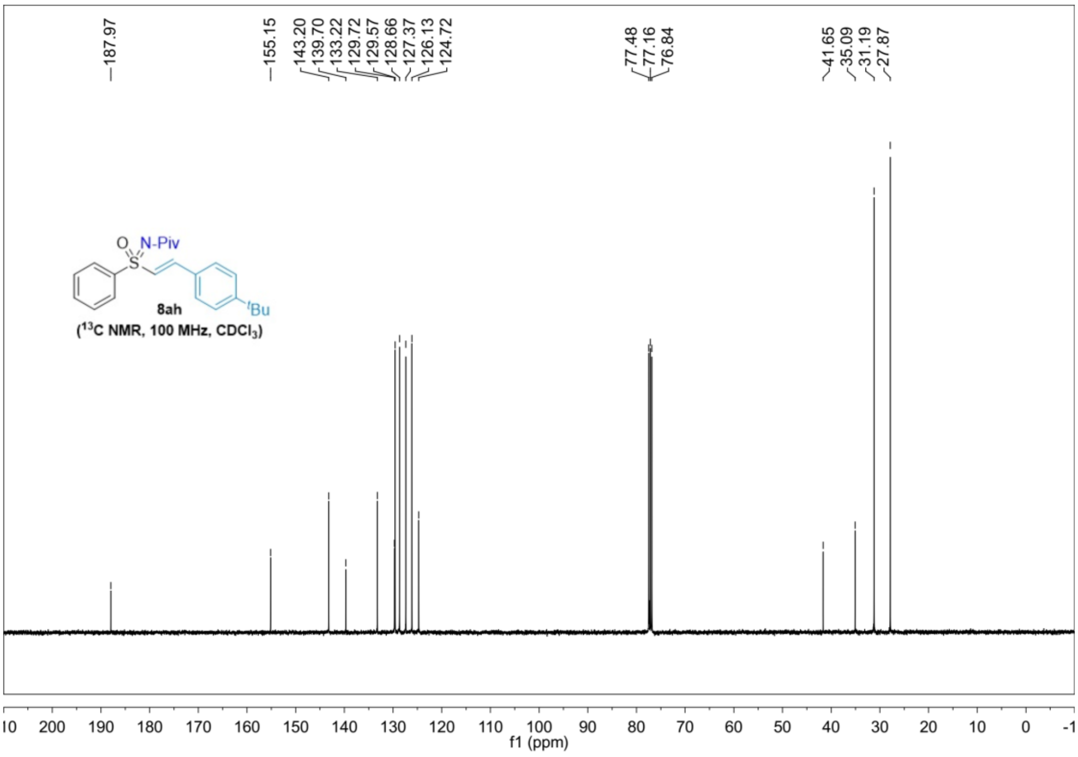
187.97
155.15
143.20
139.70
133.22
129.72
129.57
128.66
127.37
126.13
124.72
77.48
77.16
76.84
41.65
35.09
31.19
27.87
N-Piv
8ah
(¹³C NMR, 100 MHz, CDCl₃)
f1 (ppm)

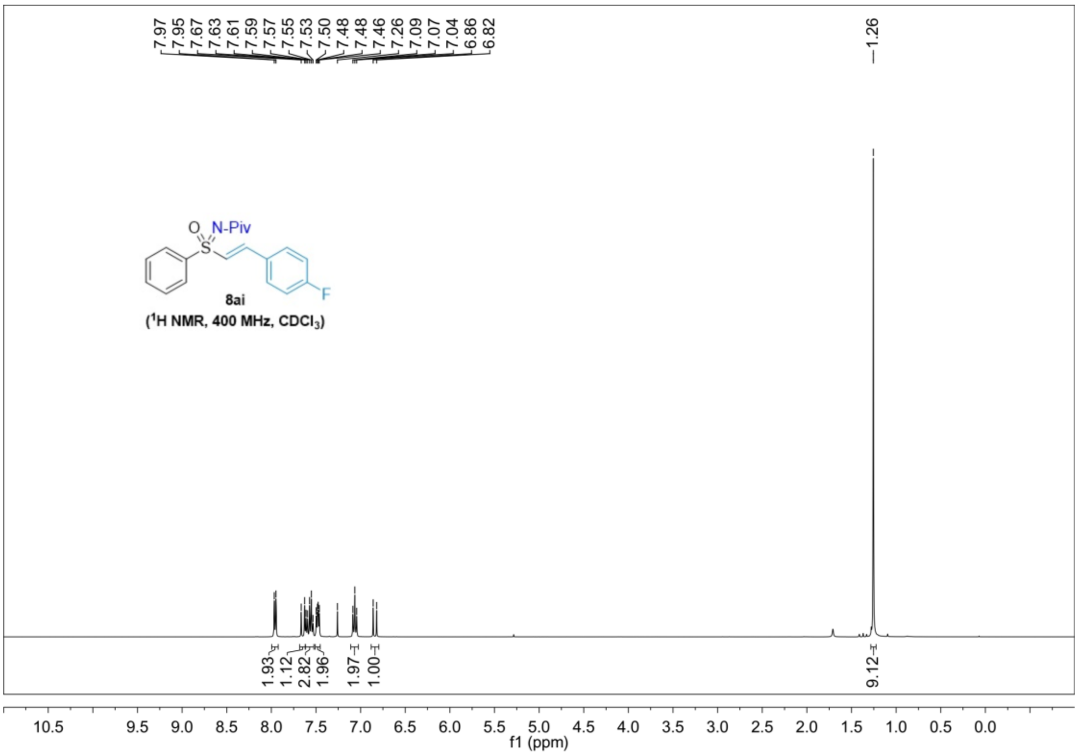
7.97
7.95
7.67
7.63
7.61
7.59
7.57
7.55
7.53
7.50
7.48
7.48
7.46
7.26
7.09
7.07
7.04
6.86
6.82
1.26
O N-Piv
S
F
8ai
(1H NMR, 400 MHz, CDCl3)
1.93
1.12
2.82
1.96
1.97
1.00
9.12
f1 (ppm)

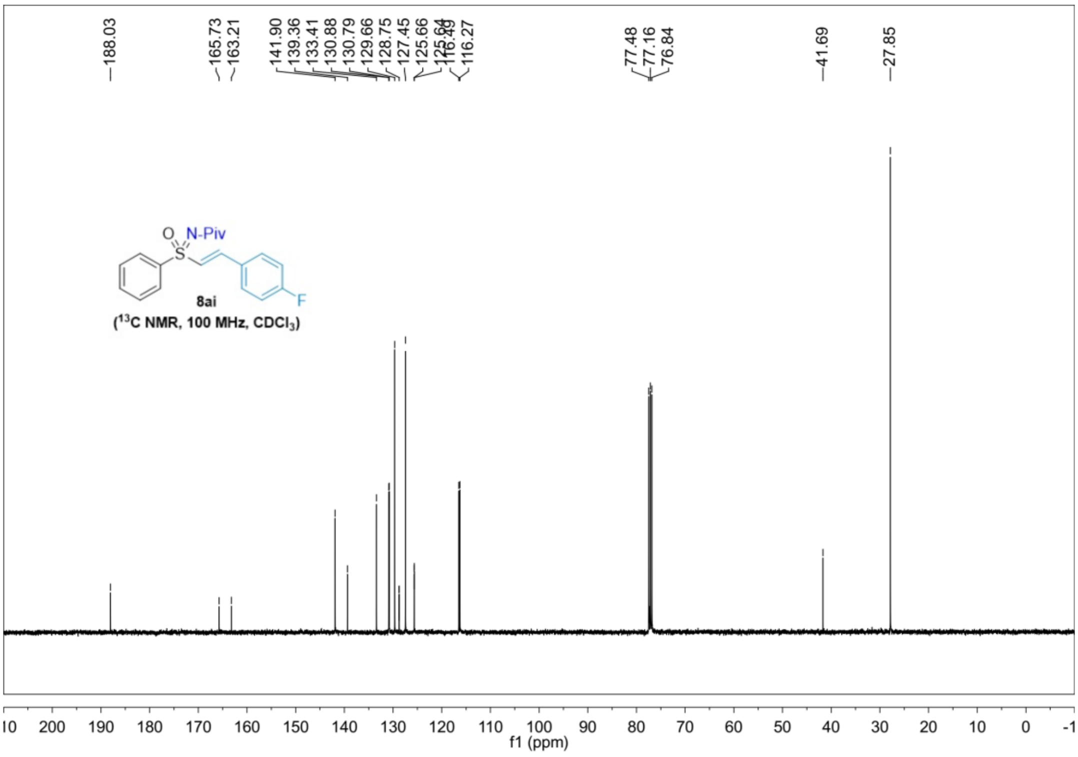
188.03
165.73
163.21
141.90
139.36
133.41
130.88
130.79
129.66
128.75
127.45
125.66
125.64
116.49
116.27
77.48
77.16
76.84
41.69
27.85
O N-Piv
S
F
8ai
(13C NMR, 100 MHz, CDCl3)
f1 (ppm)

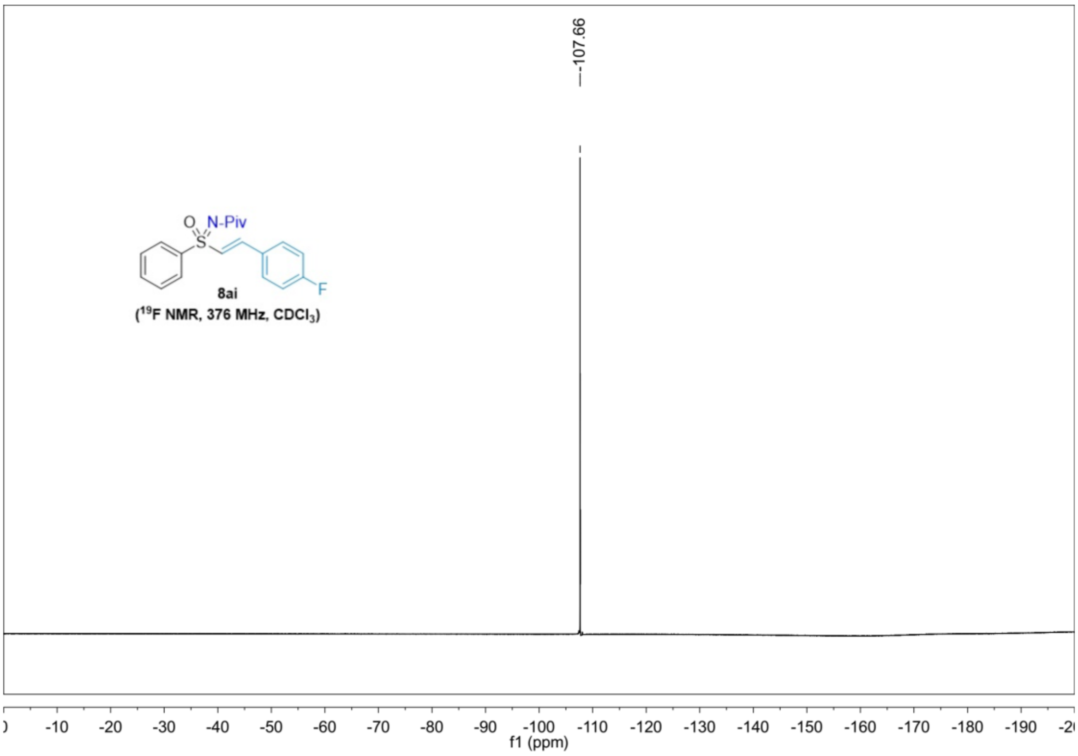
-107.66
N-Piv
8ai
(19F NMR, 376 MHz, CDCl3)
f1 (ppm)

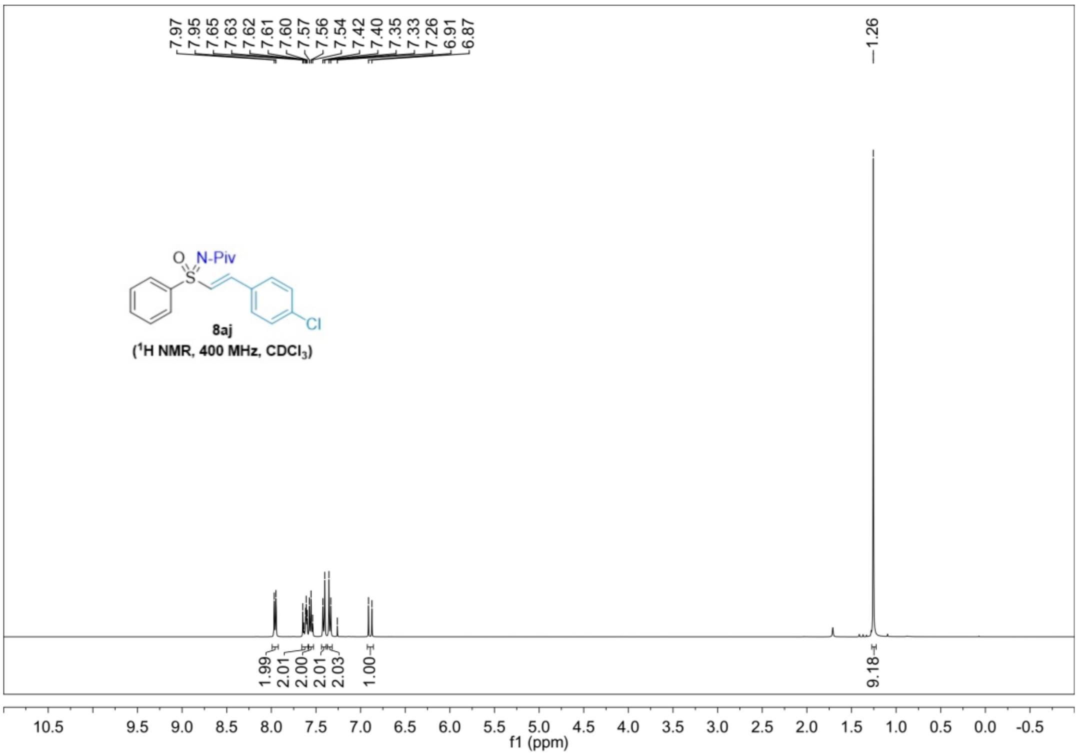
7.97 7.95 7.65 7.63 7.62 7.61 7.60 7.57 7.56 7.54 7.42 7.40 7.35 7.33 7.26 6.91 6.87
1.26
N-Piv
Cl
8aj
(1H NMR, 400 MHz, CDCl3)
1.99 2.01 2.00 2.01 2.03 1.00
9.18
f1 (ppm)

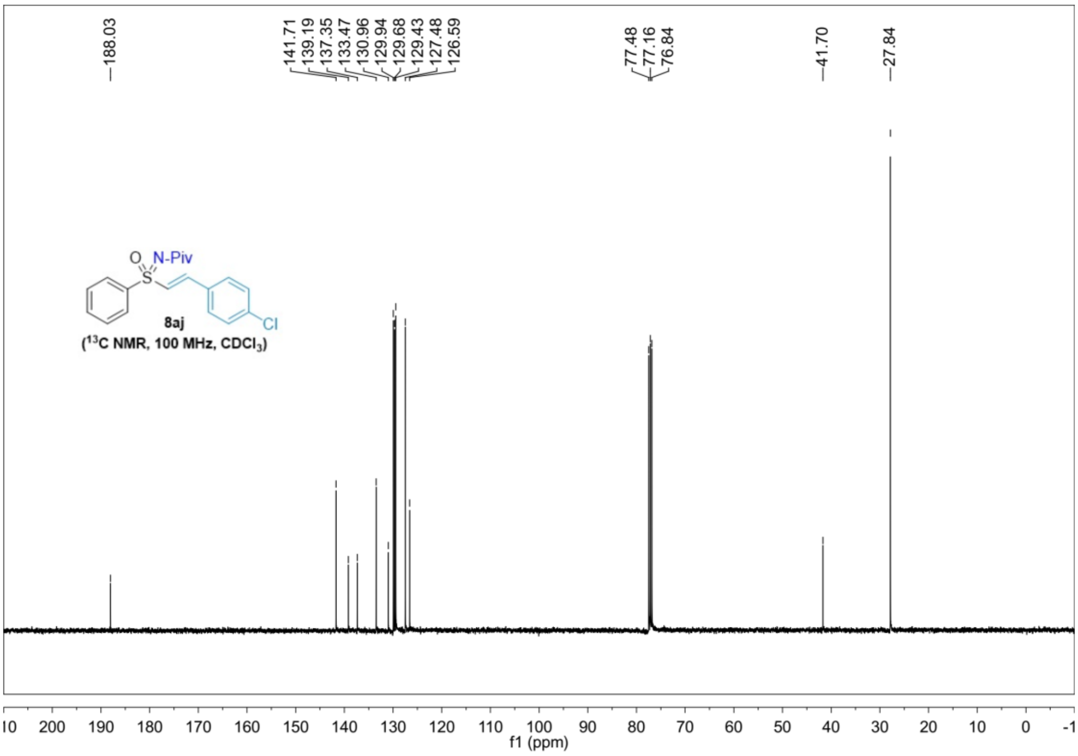
188.03
141.71
139.19
137.35
133.47
130.96
129.94
129.68
129.43
127.48
126.59
77.48
77.16
76.84
41.70
27.84
N-Piv
O
S
Cl
8aj
(13C NMR, 100 MHz, CDCl3)
f1 (ppm)

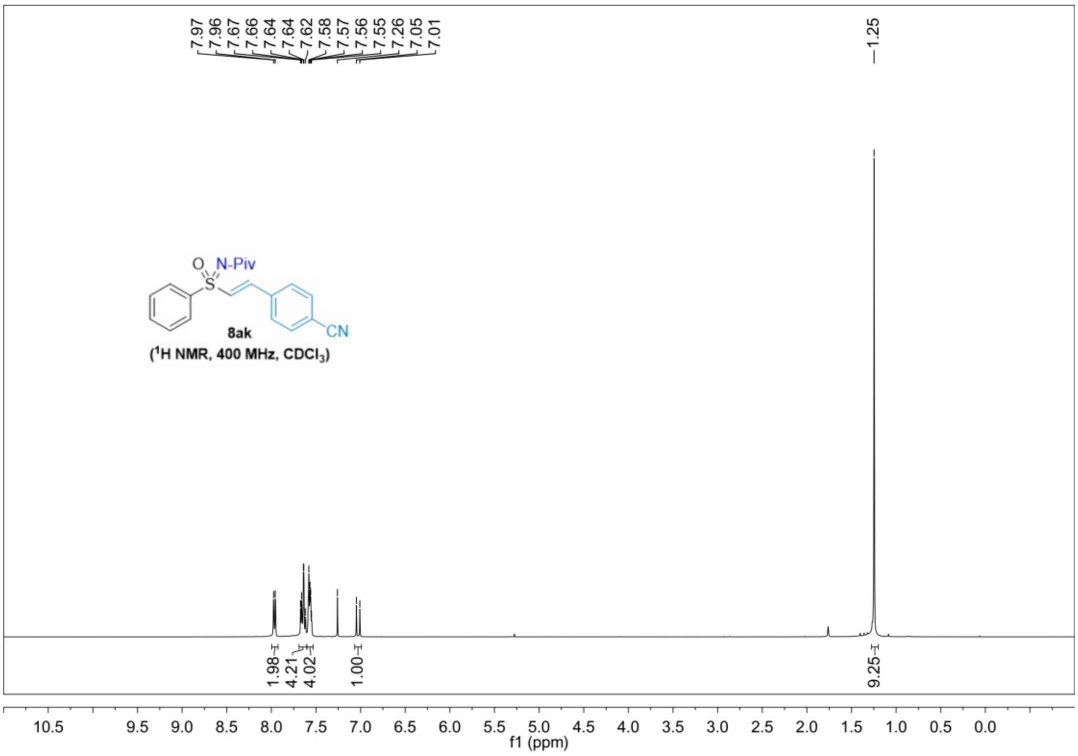
7.97
7.96
7.67
7.66
7.64
7.64
7.62
7.58
7.57
7.56
7.55
7.26
7.05
7.01
1.25
N-Piv
O
S
CN
8ak
(1H NMR, 400 MHz, CDCl3)
1.98
4.21
4.02
1.00
9.25
f1 (ppm)

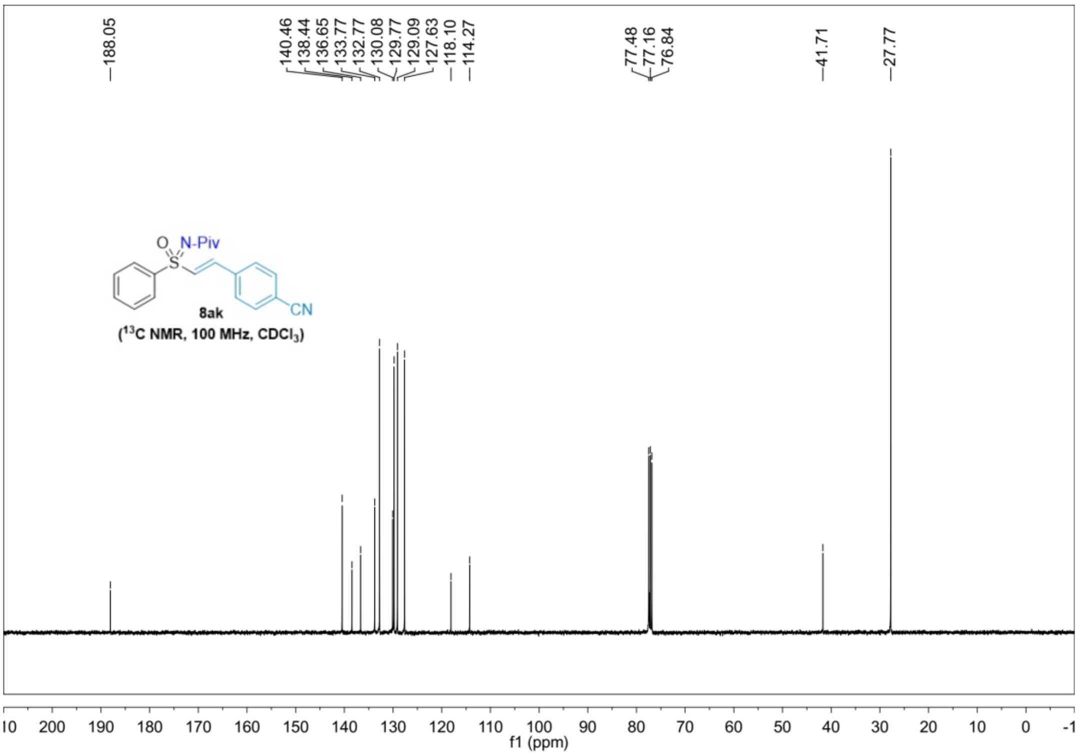
188.05
140.46
138.44
136.65
133.77
132.77
130.08
129.77
129.09
127.63
118.10
114.27
77.48
77.16
76.84
41.71
27.77
N-Piv
O
S
CN
8ak
(13C NMR, 100 MHz, CDCl3)
f1 (ppm)

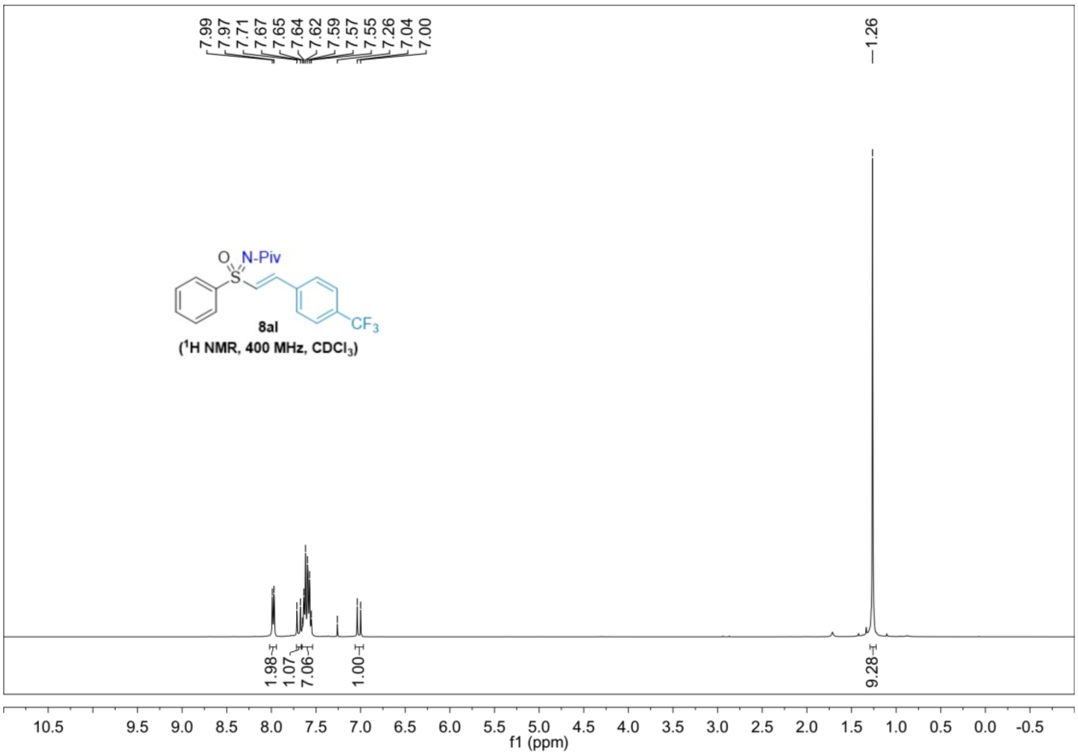
7.99
7.97
7.71
7.67
7.65
7.64
7.62
7.59
7.57
7.55
7.26
7.04
7.00
1.26
N-Piv
O
S
CF3
8al
(1H NMR, 400 MHz, CDCl3)
1.98
1.07
7.06
1.00
9.28
f1 (ppm)

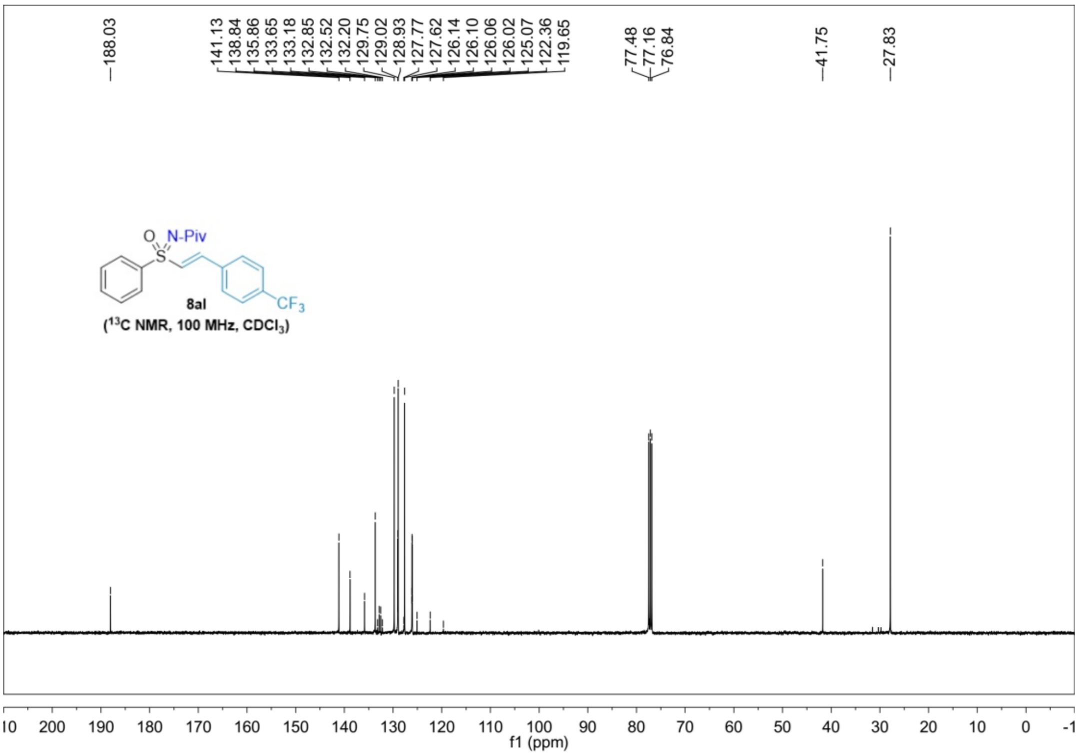
188.03
141.13
138.84
135.86
133.65
133.18
132.85
132.52
132.20
129.75
129.02
128.93
127.77
127.62
126.14
126.10
126.06
126.02
125.07
122.36
119.65
77.48
77.16
76.84
41.75
27.83
N-Piv
8al
(^{13}C NMR, 100 MHz, CDCl$_3$)
f1 (ppm)

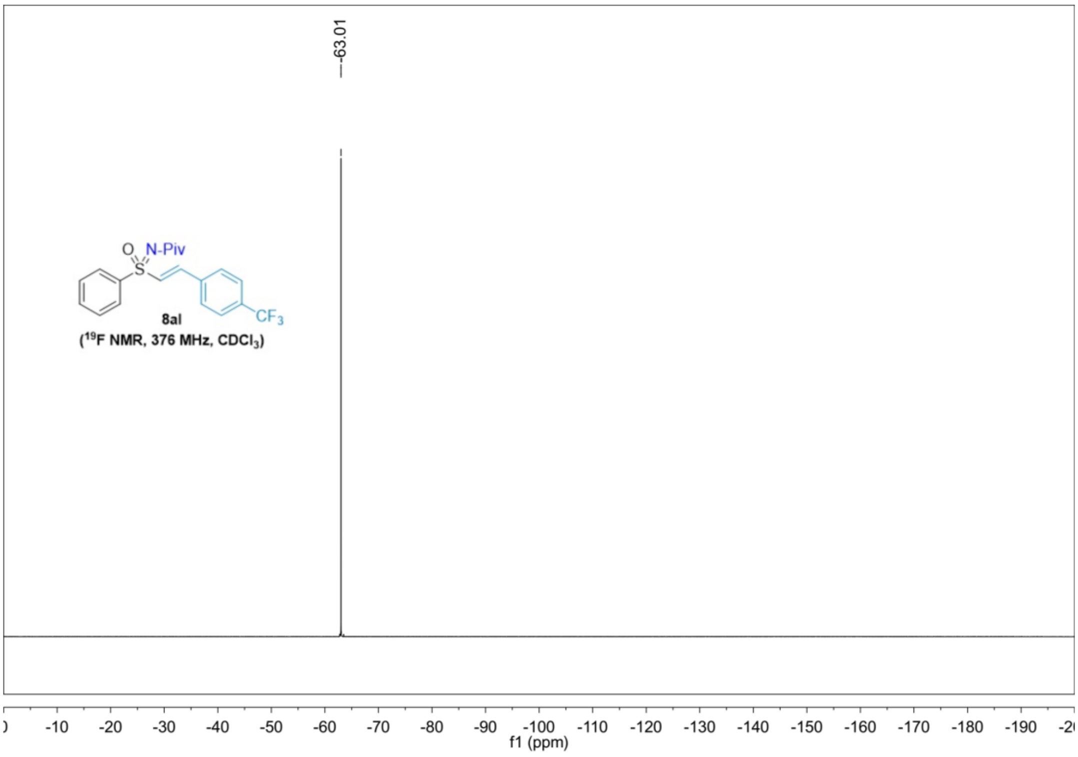
-63.01
N-Piv
8al
(^{19}F NMR, 376 MHz, CDCl$_3$)
f1 (ppm)

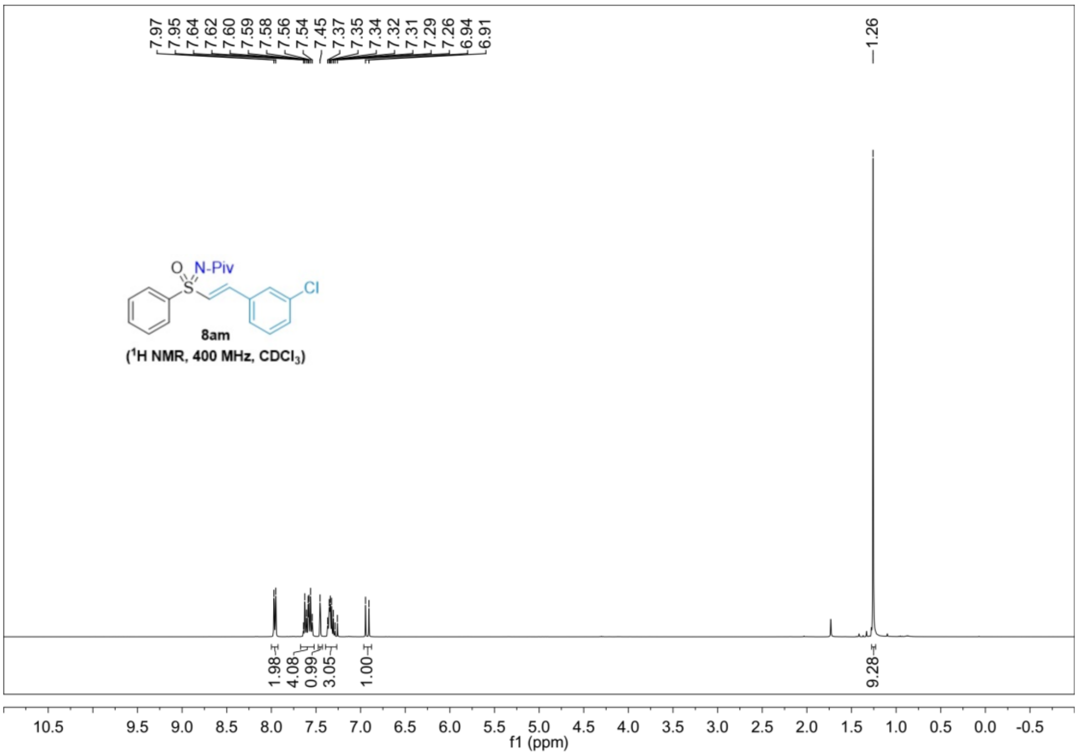
7.97
7.95
7.64
7.62
7.60
7.59
7.58
7.56
7.54
7.45
7.37
7.35
7.34
7.32
7.31
7.29
7.26
6.94
6.91
1.26
N-Piv
O
S
Cl
8am
(1H NMR, 400 MHz, CDCl3)
1.98
4.08
0.99
3.05
1.00
9.28
f1 (ppm)

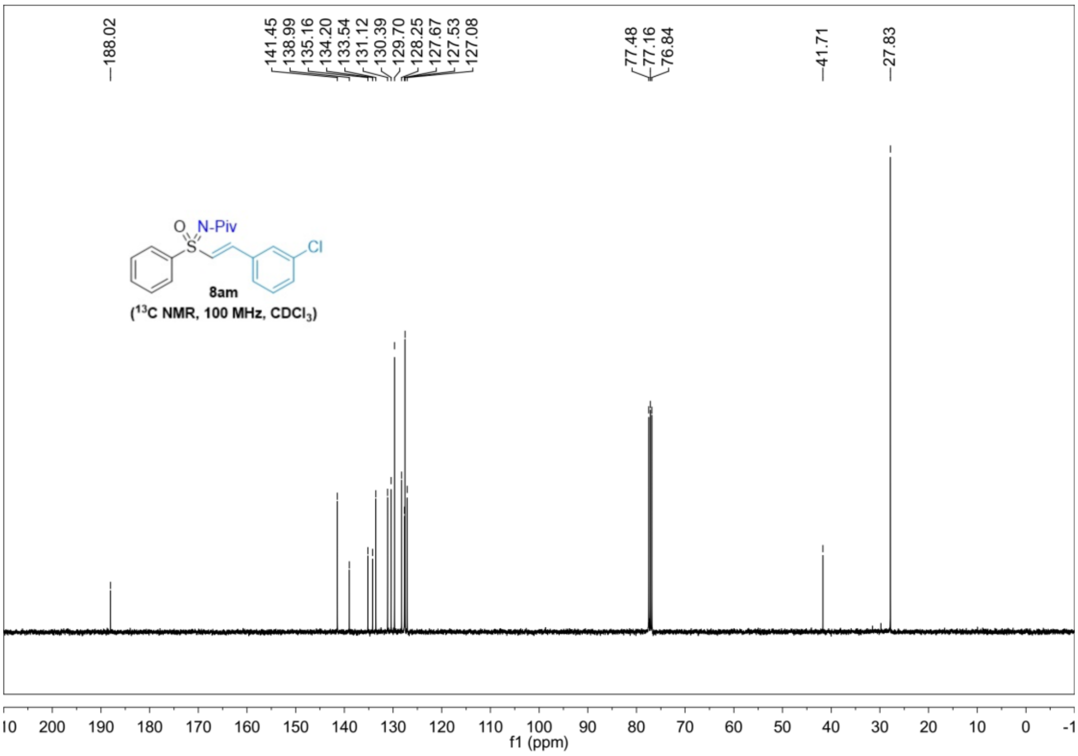
188.02
141.45
138.99
135.16
134.20
133.54
131.12
130.39
129.70
128.25
127.67
127.53
127.08
77.48
77.16
76.84
41.71
27.83
N-Piv
O
S
Cl
8am
(13C NMR, 100 MHz, CDCl3)
f1 (ppm)

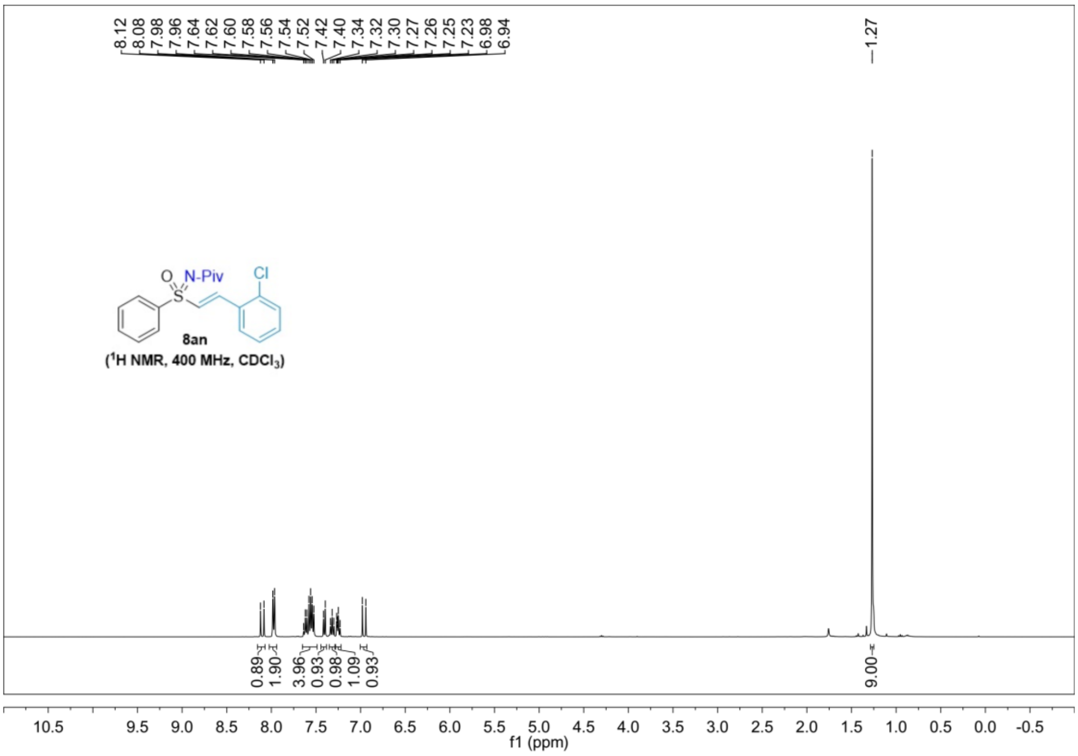
8.12
8.08
7.98
7.96
7.64
7.62
7.60
7.58
7.56
7.54
7.52
7.42
7.40
7.34
7.32
7.30
7.27
7.26
7.25
7.23
6.98
6.94
1.27
O
N-Piv
S
Cl
8an
(1H NMR, 400 MHz, CDCl3)
0.89
1.90
3.96
0.93
0.98
1.09
0.93
9.00
10.5 9.5 9.0 8.5 8.0 7.5 7.0 6.5 6.0 5.5 5.0 4.5 4.0 3.5 3.0 2.5 2.0 1.5 1.0 0.5 0.0 -0.5
f1 (ppm)

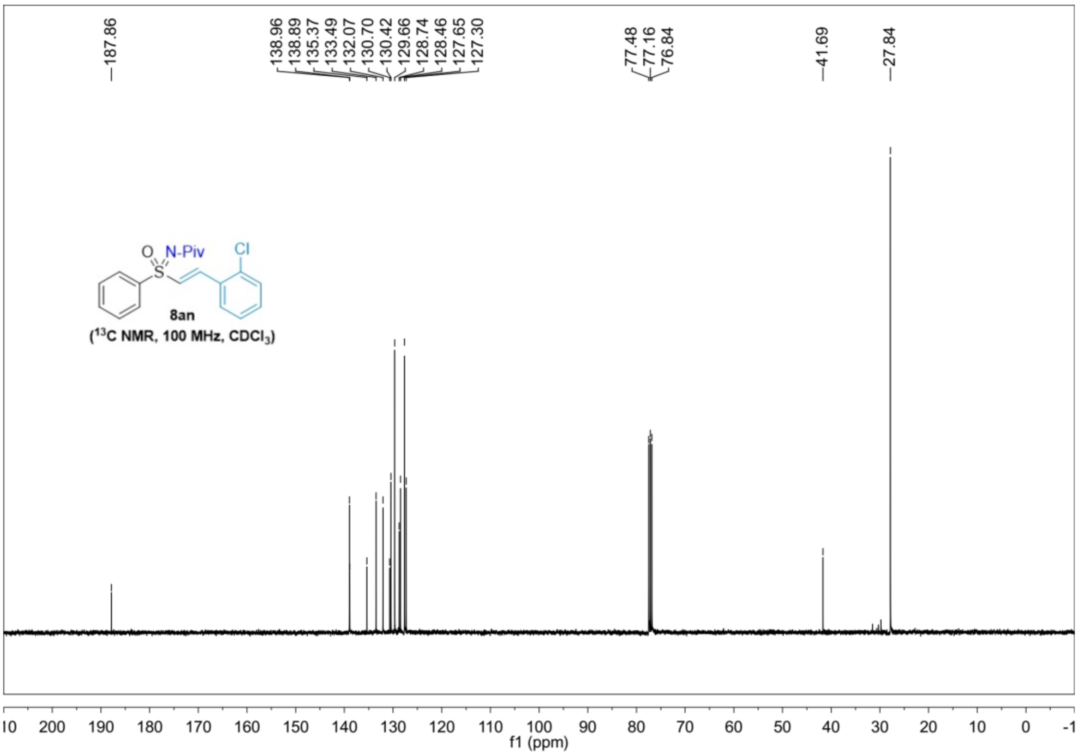
187.86
138.96
138.89
135.37
133.49
132.07
130.70
130.42
129.66
128.74
128.46
127.65
127.30
77.48
77.16
76.84
41.69
27.84
O
N-Piv
S
Cl
8an
(13C NMR, 100 MHz, CDCl3)
200 190 180 170 160 150 140 130 120 110 100 90 80 70 60 50 40 30 20 10 0
f1 (ppm)

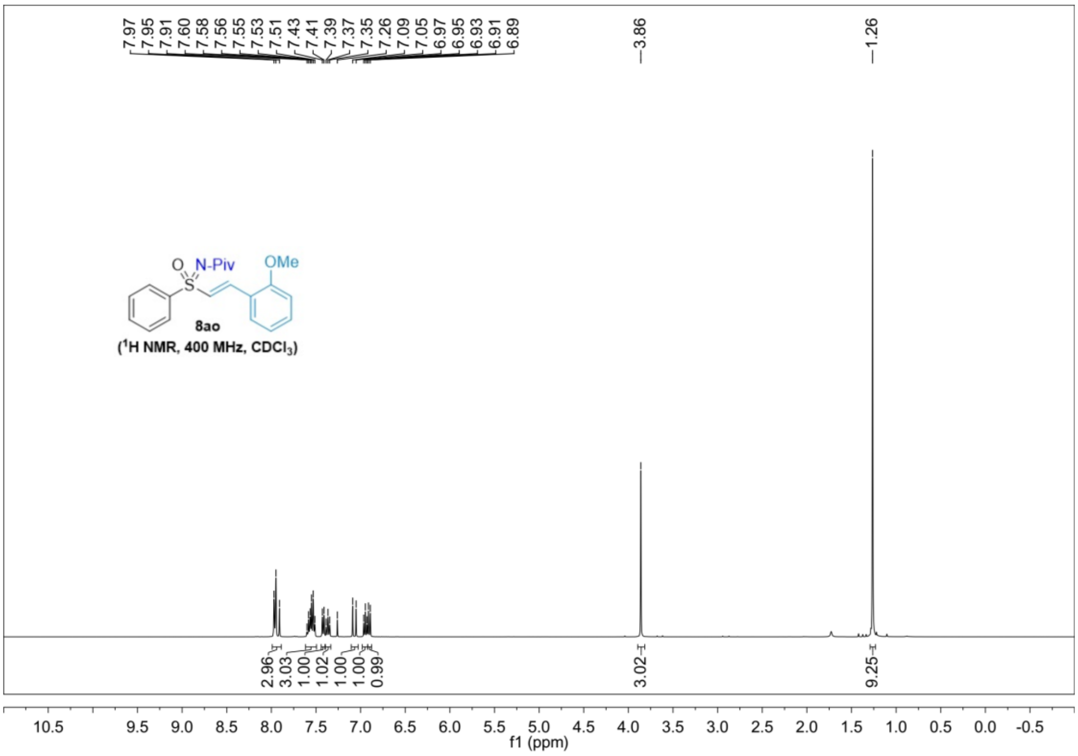
8ao
(1H NMR, 400 MHz, CDCl3)
f1 (ppm)

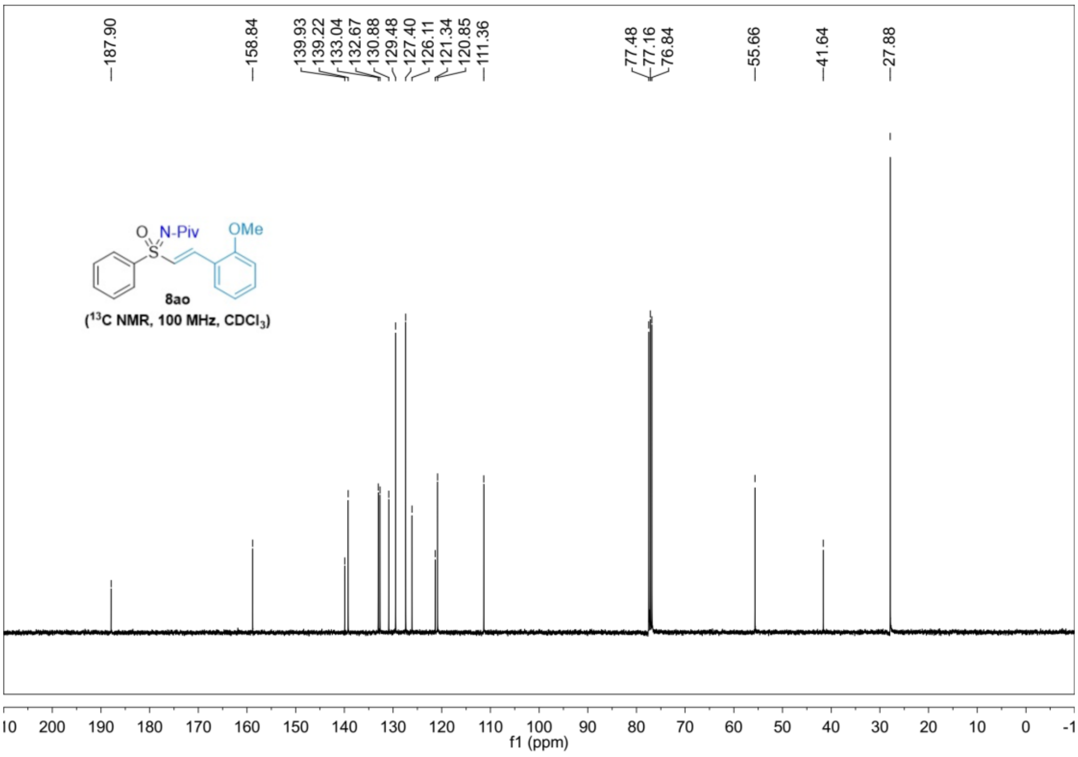
8ao
(13C NMR, 100 MHz, CDCl3)
f1 (ppm)

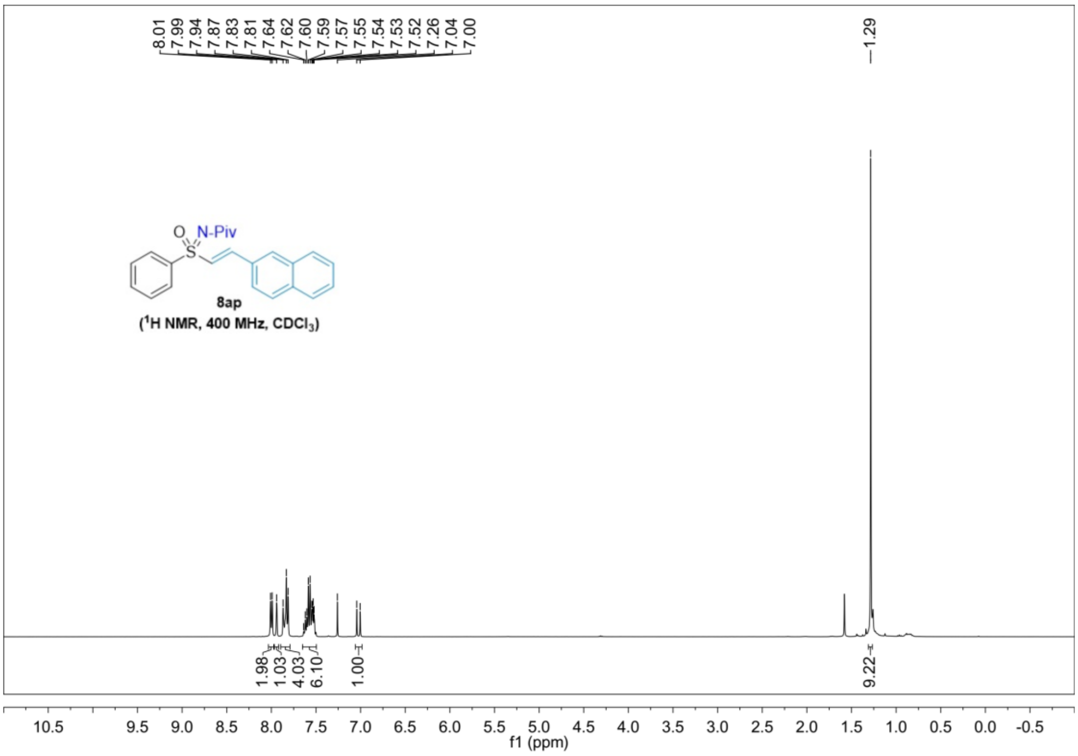
8.01
7.99
7.94
7.87
7.83
7.81
7.64
7.62
7.60
7.59
7.57
7.55
7.54
7.53
7.52
7.26
7.04
7.00
1.29
O
N-Piv
S
8ap
(1H NMR, 400 MHz, CDCl3)
1.98
1.03
4.03
6.10
1.00
9.22
10.5
9.5
9.0
8.5
8.0
7.5
7.0
6.5
6.0
5.5
5.0
4.5
4.0
3.5
3.0
2.5
2.0
1.5
1.0
0.5
0.0
-0.5
f1 (ppm)

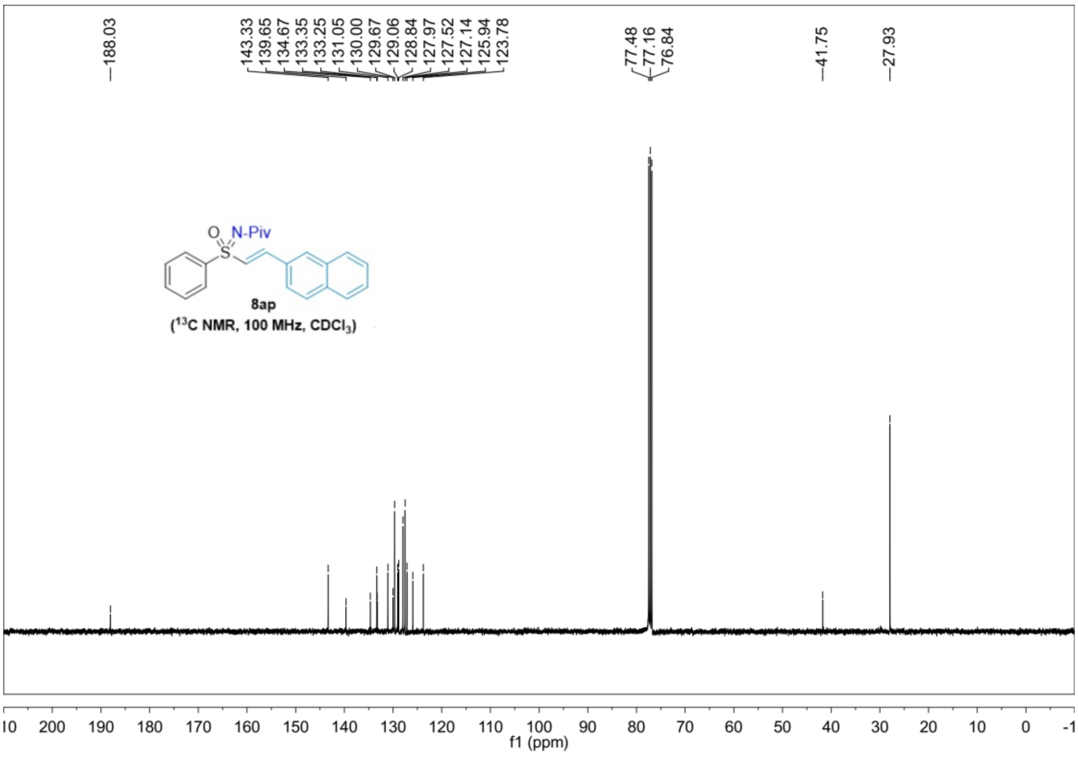
188.03
143.33
139.65
134.67
133.35
133.25
131.05
130.00
129.67
129.06
128.84
127.97
127.52
127.14
125.94
123.78
77.48
77.16
76.84
41.75
27.93
O
N-Piv
S
8ap
(13C NMR, 100 MHz, CDCl3)
200
190
180
170
160
150
140
130
120
110
100
90
80
70
60
50
40
30
20
10
0
f1 (ppm)

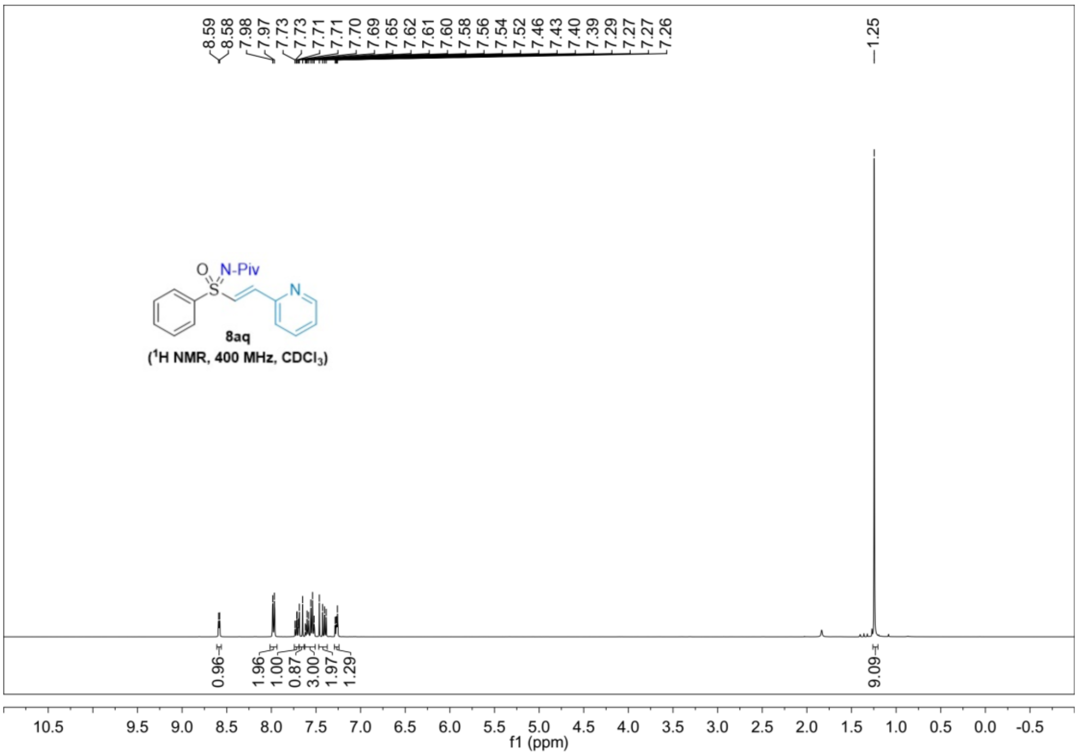
8.59
8.58
7.98
7.97
7.73
7.73
7.71
7.70
7.69
7.65
7.62
7.61
7.60
7.58
7.56
7.54
7.52
7.46
7.43
7.40
7.39
7.29
7.27
7.27
7.26
1.25
N-Piv
8aq
(1H NMR, 400 MHz, CDCl3)
0.96
1.96
1.00
0.87
3.00
1.97
1.29
9.09
f1 (ppm)

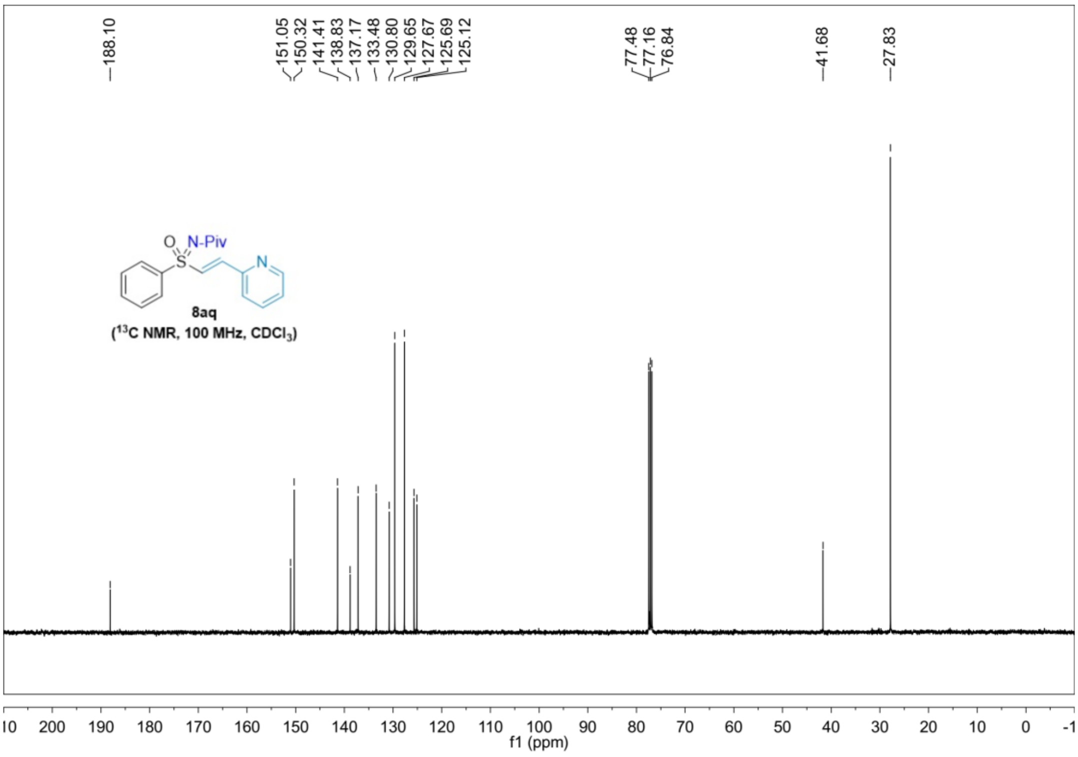
188.10
151.05
150.32
141.41
138.83
137.17
133.48
130.80
129.65
127.67
125.69
125.12
77.48
77.16
76.84
41.68
27.83
N-Piv
8aq
(13C NMR, 100 MHz, CDCl3)
f1 (ppm)

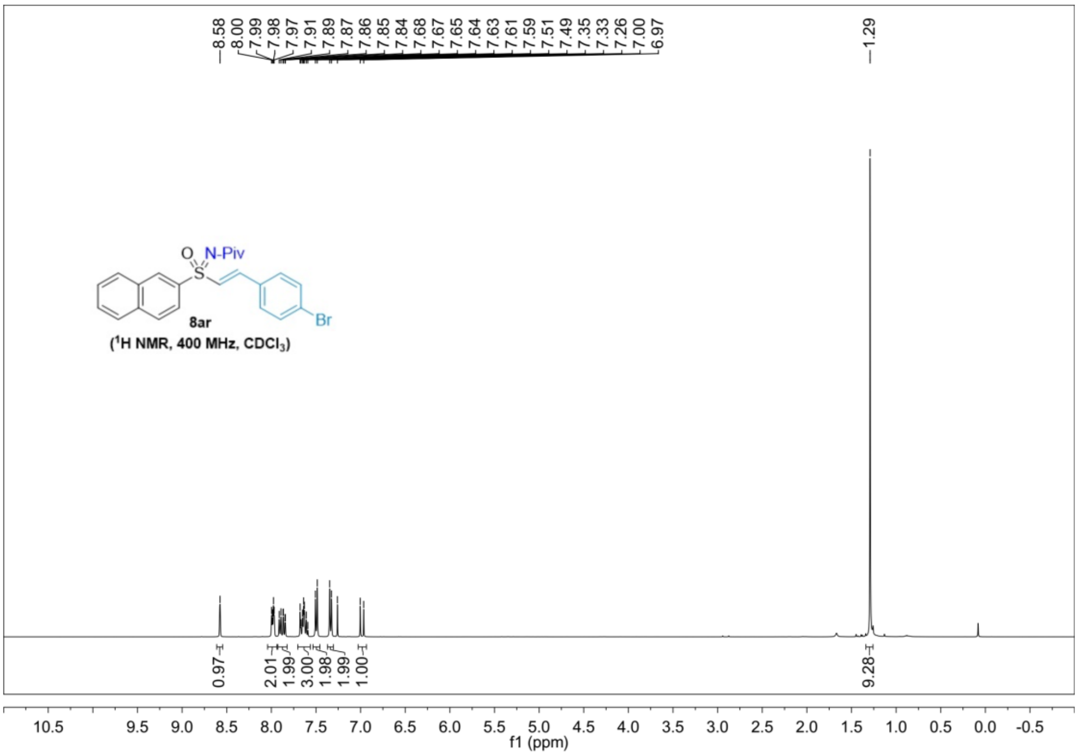
8.58
8.00
7.99
7.98
7.97
7.91
7.89
7.87
7.86
7.85
7.84
7.68
7.67
7.65
7.64
7.63
7.61
7.59
7.51
7.49
7.35
7.33
7.26
7.00
6.97
1.29
N-Piv
O
S
Br
8ar
(¹H NMR, 400 MHz, CDCl3)
0.97
2.01
1.99
3.00
1.98
1.99
1.00
9.28
10.5
9.5
9.0
8.5
8.0
7.5
7.0
6.5
6.0
5.5
5.0
4.5
4.0
3.5
3.0
2.5
2.0
1.5
1.0
0.5
0.0
-0.5
f1 (ppm)

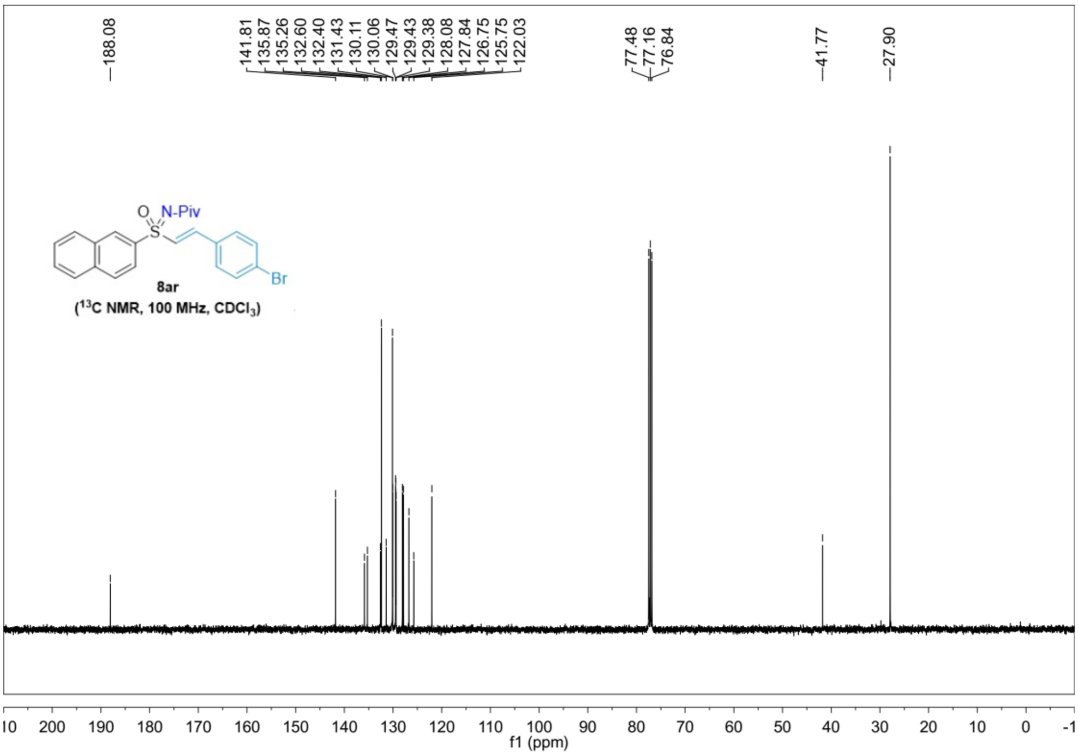
188.08
141.81
135.87
135.26
132.60
132.40
131.43
130.11
130.06
129.47
129.43
129.38
128.08
127.84
126.75
125.75
122.03
77.48
77.16
76.84
41.77
27.90
N-Piv
O
S
Br
8ar
(¹³C NMR, 100 MHz, CDCl3)
200
190
180
170
160
150
140
130
120
110
100
90
80
70
60
50
40
30
20
10
0
f1 (ppm)

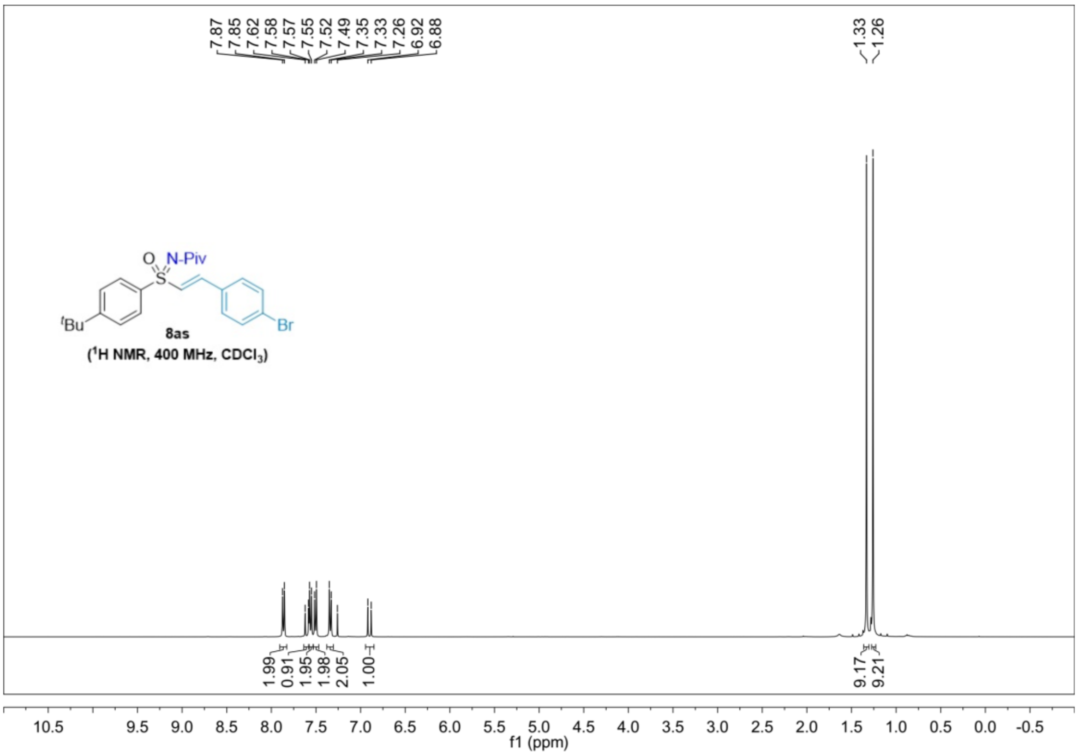
7.87
7.85
7.62
7.58
7.57
7.55
7.52
7.49
7.35
7.33
7.26
6.92
6.88
1.33
1.26
N-Piv
O
S
tBu
Br
8as
(1H NMR, 400 MHz, CDCl3)
1.99
0.91
1.95
1.98
2.05
1.00
9.17
9.21
f1 (ppm)

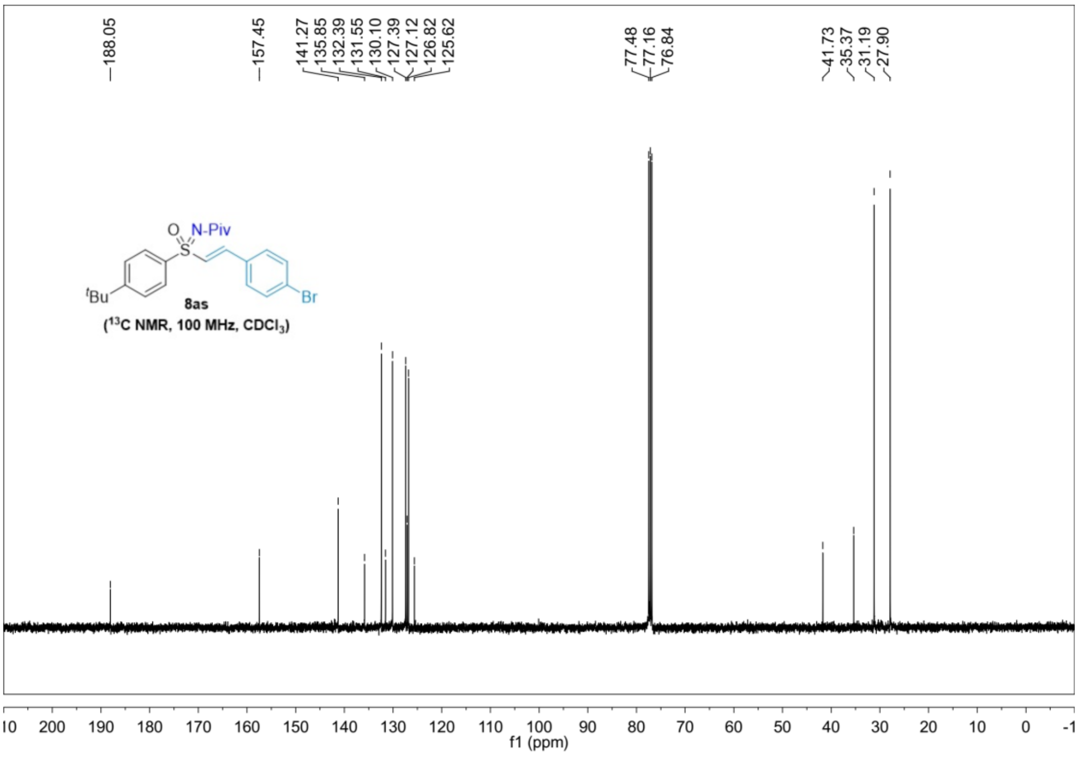
188.05
157.45
141.27
135.85
132.39
131.55
130.10
127.39
127.12
126.82
125.62
77.48
77.16
76.84
41.73
35.37
31.19
27.90
N-Piv
O
S
tBu
Br
8as
(13C NMR, 100 MHz, CDCl3)
f1 (ppm)

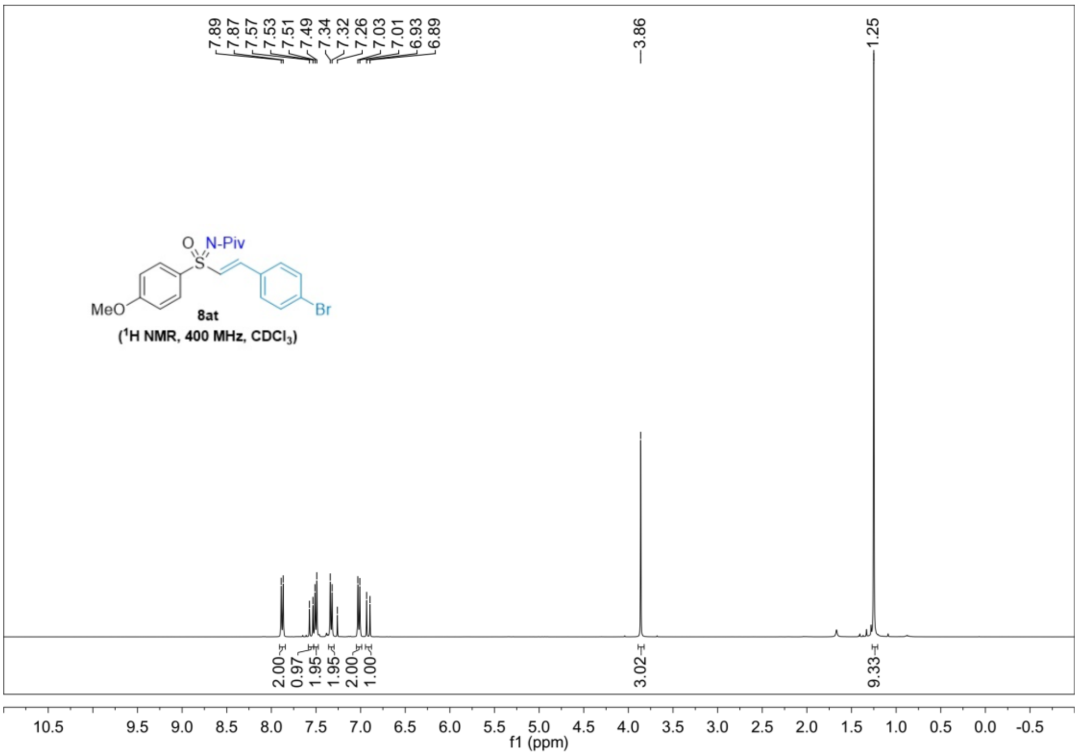
7.89
7.87
7.57
7.53
7.51
7.49
7.34
7.32
7.26
7.03
7.01
6.93
6.89
3.86
1.25
O
N-Piv
S
MeO
Br
8at
(1H NMR, 400 MHz, CDCl3)
2.00
0.97
1.95
1.95
2.00
1.00
3.02
9.33
10.5
9.5
9.0
8.5
8.0
7.5
7.0
6.5
6.0
5.5
5.0
4.5
4.0
3.5
3.0
2.5
2.0
1.5
1.0
0.5
0.0
-0.5
f1 (ppm)

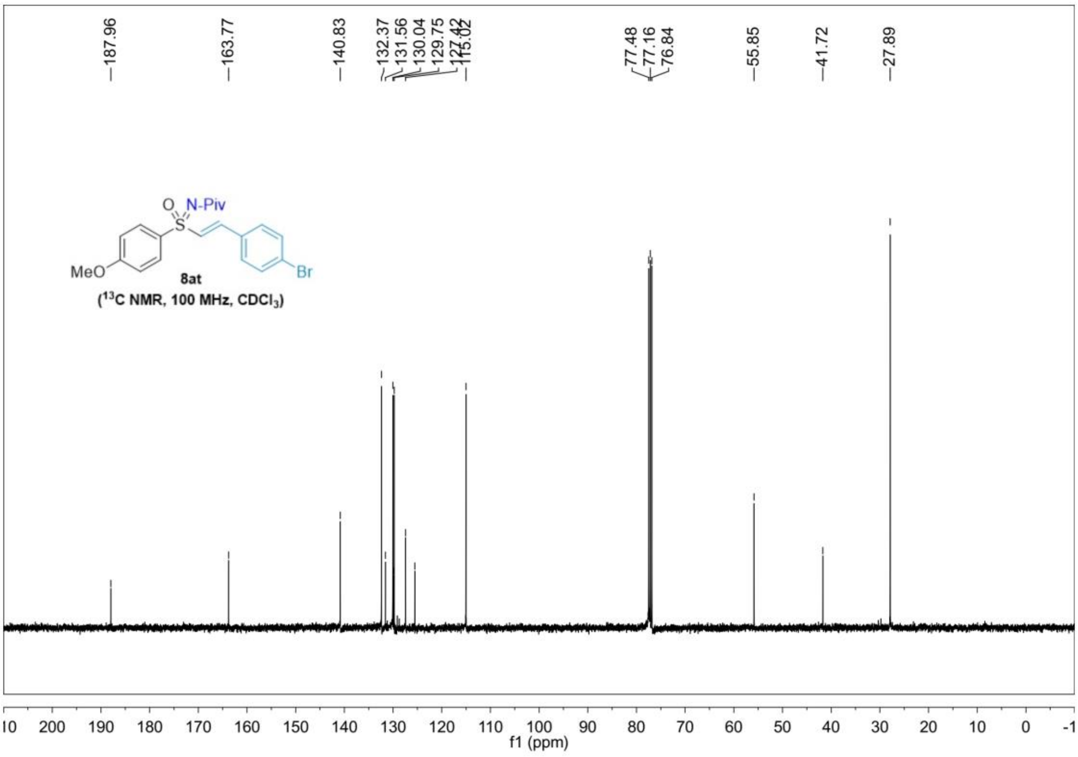
187.96
163.77
140.83
132.37
131.56
130.04
129.75
127.42
115.02
77.48
77.16
76.84
55.85
41.72
27.89
O
N-Piv
S
MeO
Br
8at
(13C NMR, 100 MHz, CDCl3)
200
190
180
170
160
150
140
130
120
110
100
90
80
70
60
50
40
30
20
10
0
f1 (ppm)

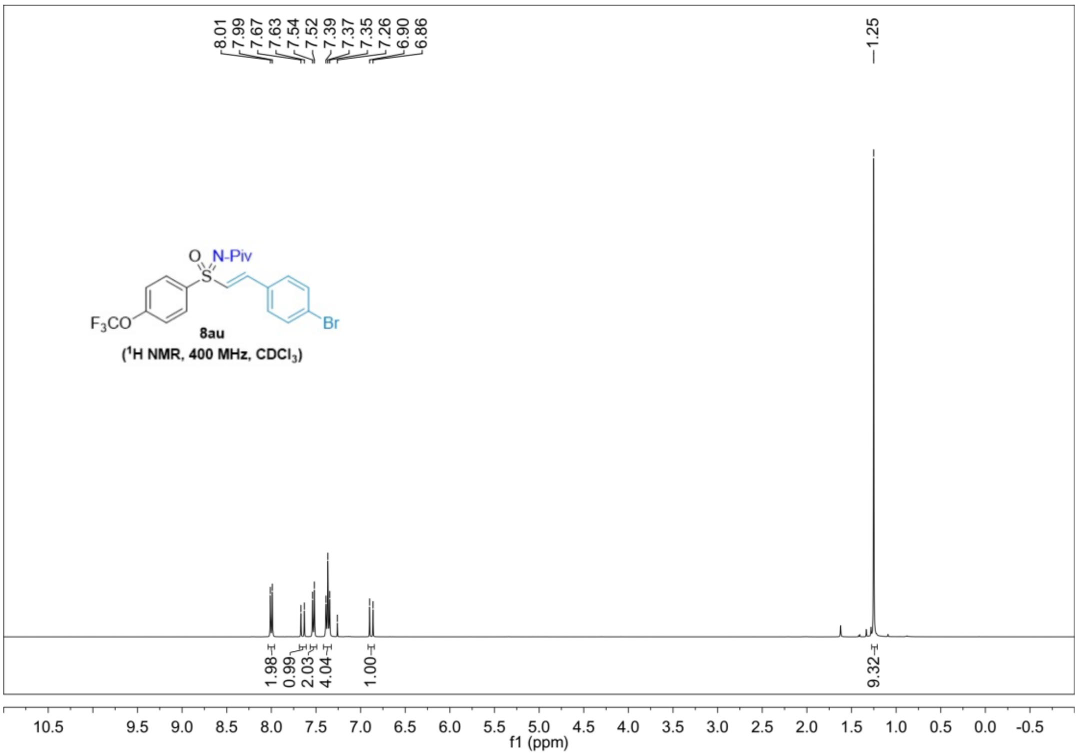
8.01
7.99
7.67
7.63
7.54
7.52
7.39
7.37
7.35
7.26
6.90
6.86
1.25
N-Piv
O
S
F_3CO
Br
8au
(^{1}H NMR, 400 MHz, CDCl$_3$)
1.98
0.99
2.03
4.04
1.00
9.32
10.5 9.5 9.0 8.5 8.0 7.5 7.0 6.5 6.0 5.5 5.0 4.5 4.0 3.5 3.0 2.5 2.0 1.5 1.0 0.5 0.0 -0.5
f1 (ppm)

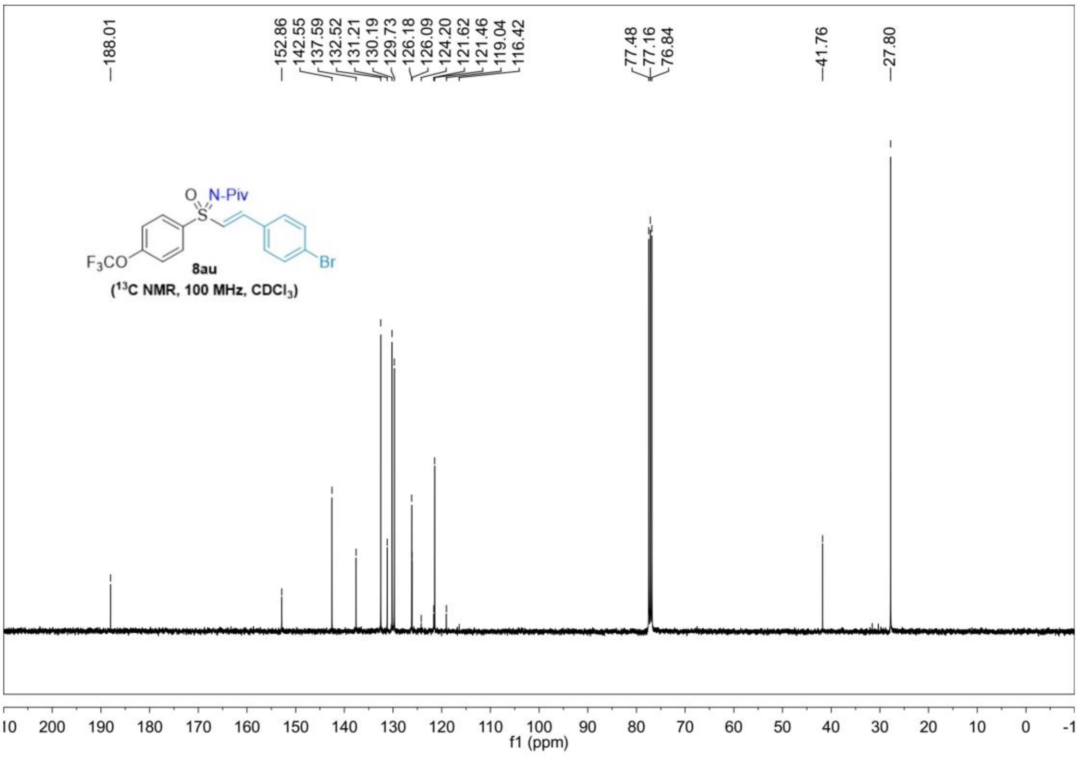
188.01
152.86
142.55
137.59
132.52
131.21
130.19
129.73
126.18
126.09
124.20
121.62
121.46
119.04
116.42
77.48
77.16
76.84
41.76
27.80
N-Piv
O
S
F_3CO
Br
8au
(^{13}C NMR, 100 MHz, CDCl$_3$)
200 190 180 170 160 150 140 130 120 110 100 90 80 70 60 50 40 30 20 10 0
f1 (ppm)

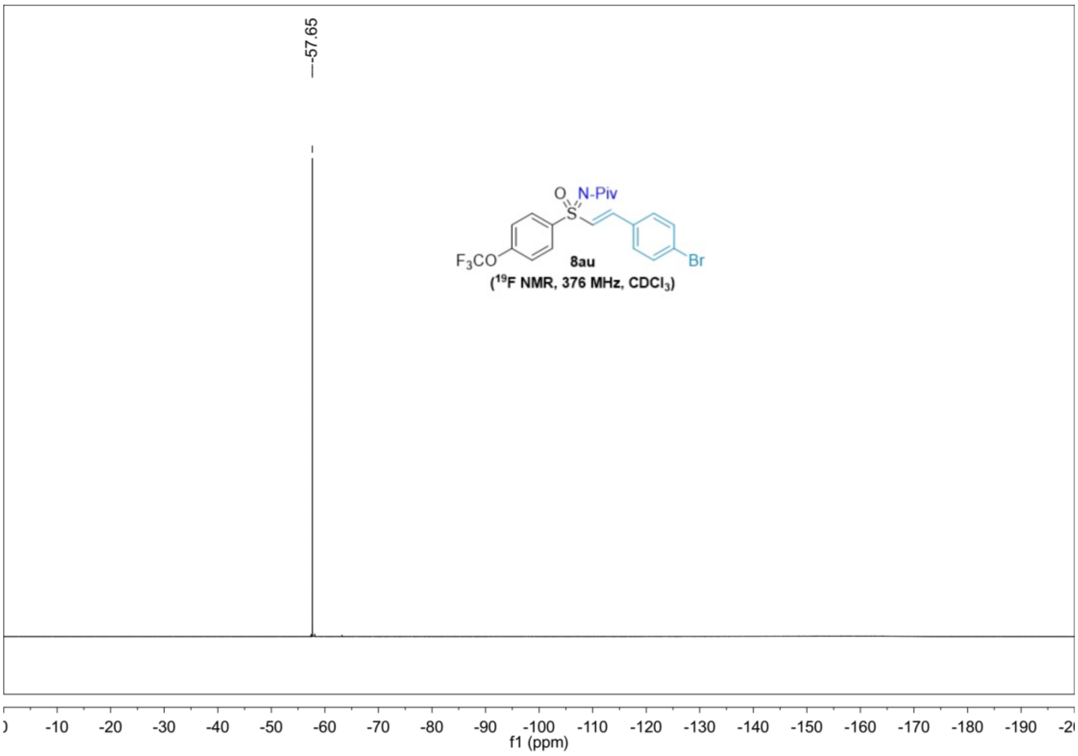
-57.65
N-Piv
O
S
F_3CO
Br
8au
(^{19}F NMR, 376 MHz, $CDCl_3$)
f1 (ppm)

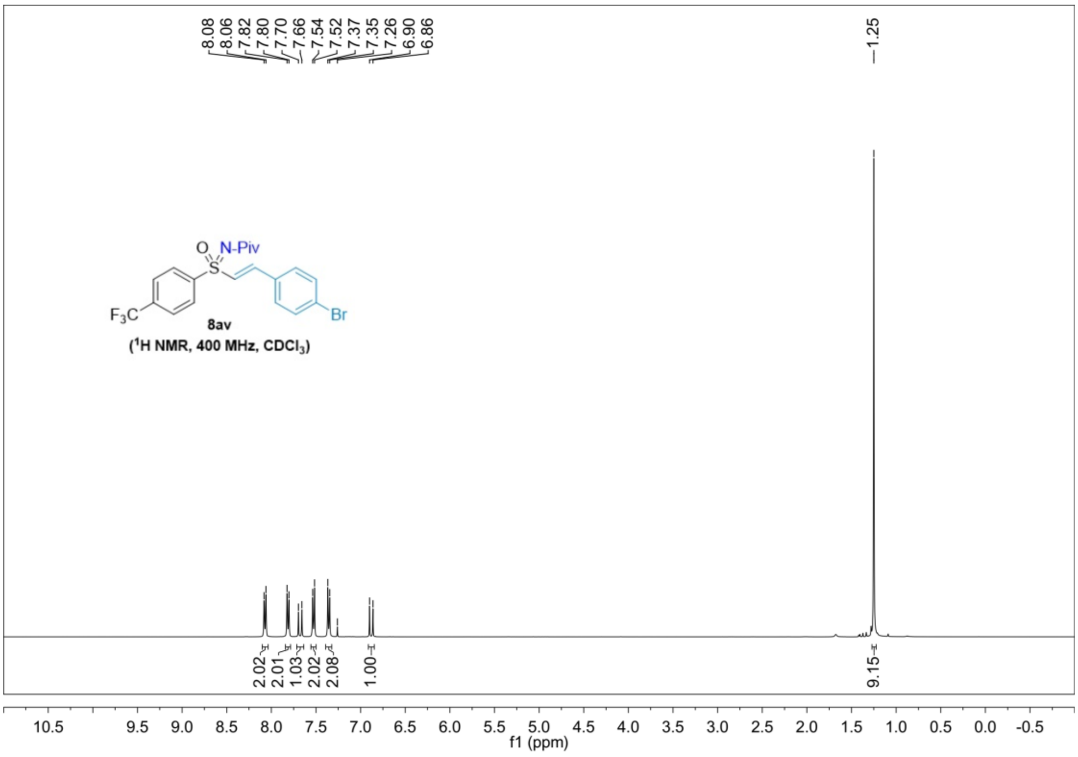
8.08
8.06
7.82
7.80
7.70
7.66
7.54
7.52
7.37
7.35
7.26
6.90
6.86
1.25
N-Piv
O
S
F_3C
Br
8av
(^{1}H NMR, 400 MHz, $CDCl_3$)
2.02
2.01
1.03
2.02
2.08
1.00
9.15
f1 (ppm)

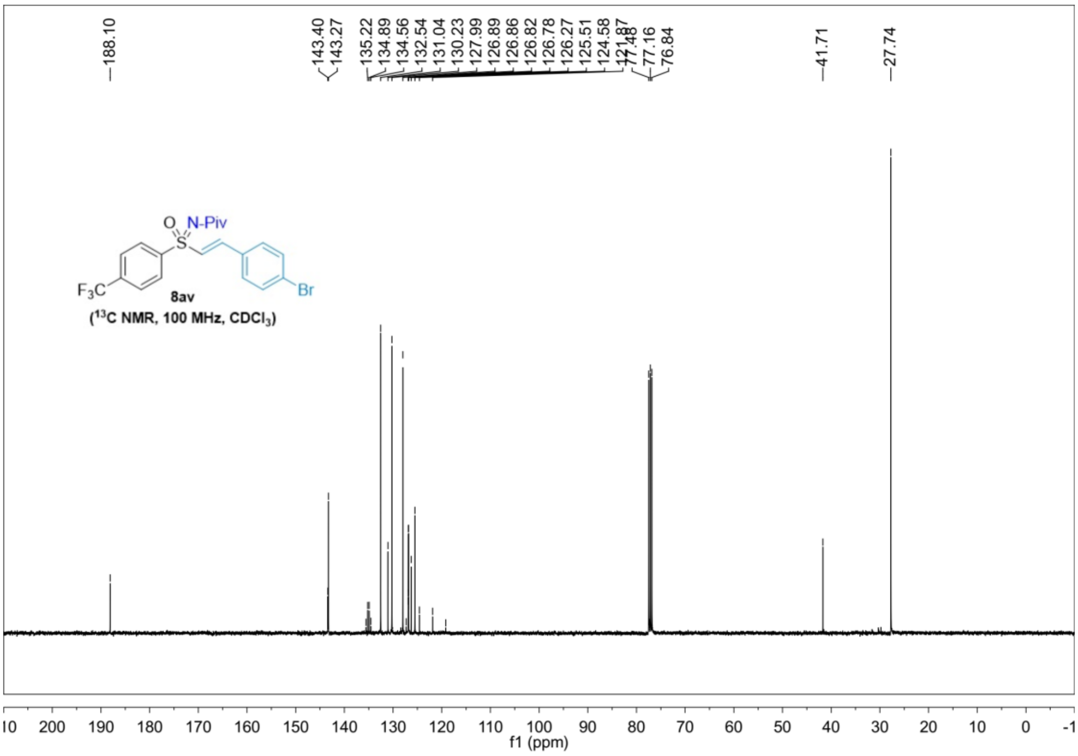
188.10
143.40
143.27
135.22
134.89
134.56
132.54
131.04
130.23
127.99
126.89
126.86
126.82
126.78
126.27
125.51
124.58
121.87
77.48
77.16
76.84
41.71
27.74
N-Piv
F3C
Br
8av
(13C NMR, 100 MHz, CDCl3)
f1 (ppm)

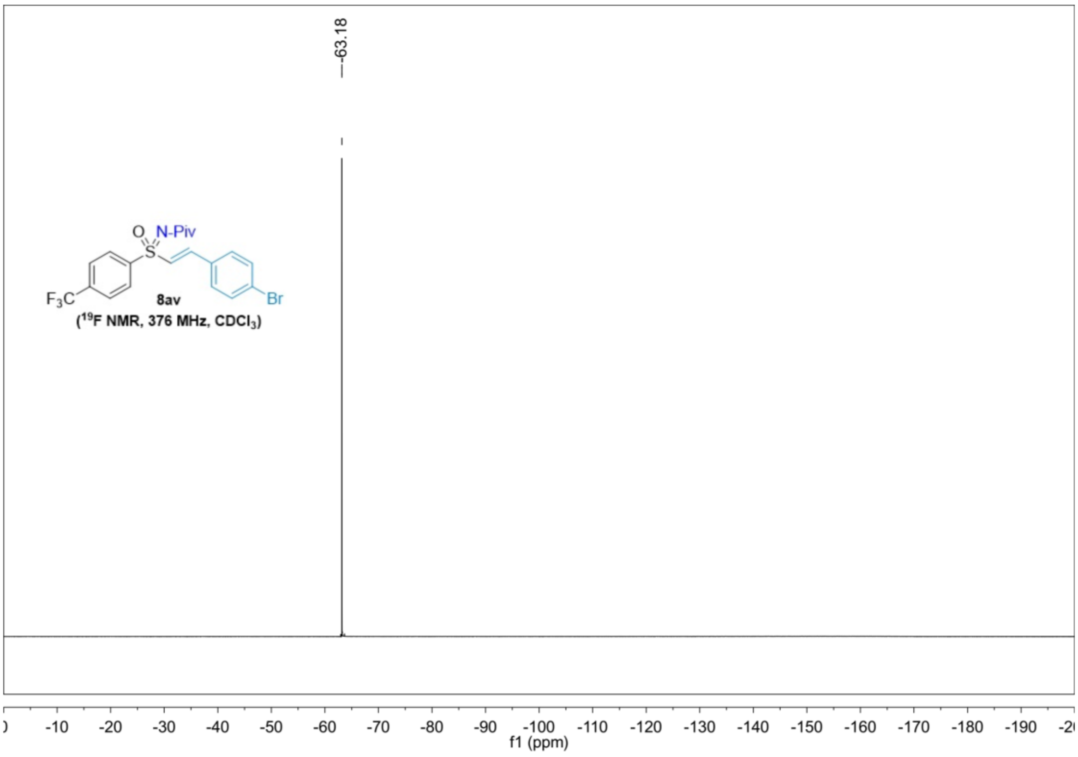
-63.18
N-Piv
F3C
Br
8av
(19F NMR, 376 MHz, CDCl3)
f1 (ppm)

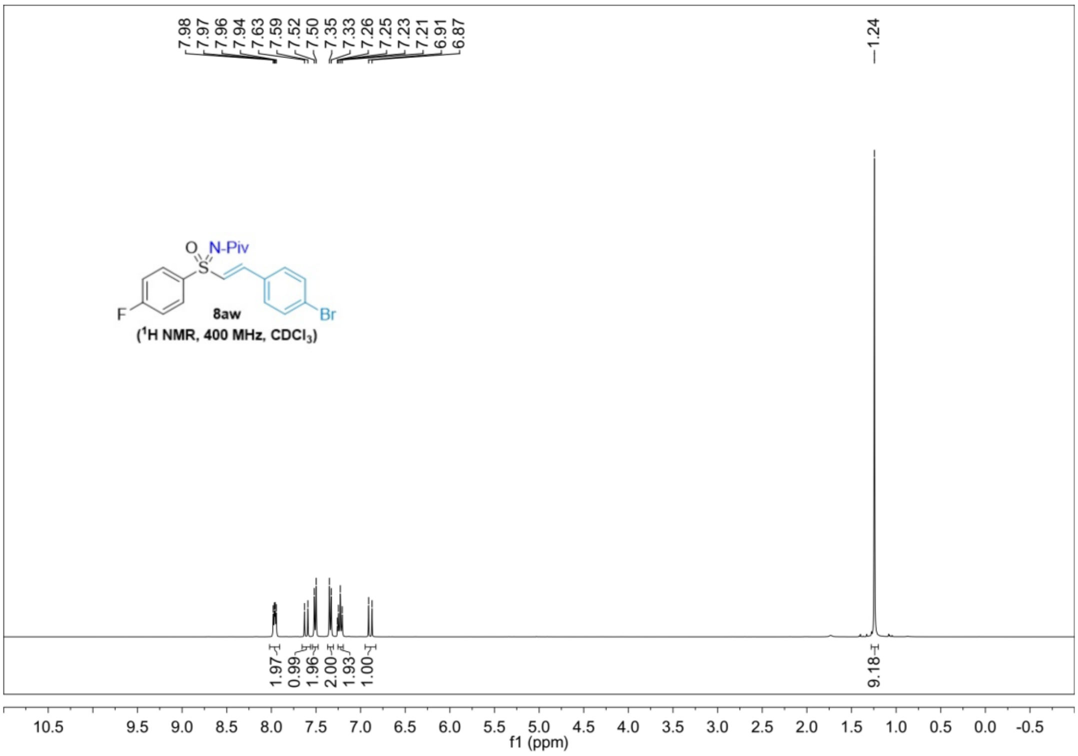
7.98
7.97
7.96
7.94
7.63
7.59
7.52
7.50
7.35
7.33
7.26
7.25
7.23
7.21
6.91
6.87
1.24
N-Piv
O
S
F
Br
8aw
(1H NMR, 400 MHz, CDCl3)
1.97
0.99
1.96
2.00
1.93
1.00
9.18
f1 (ppm)

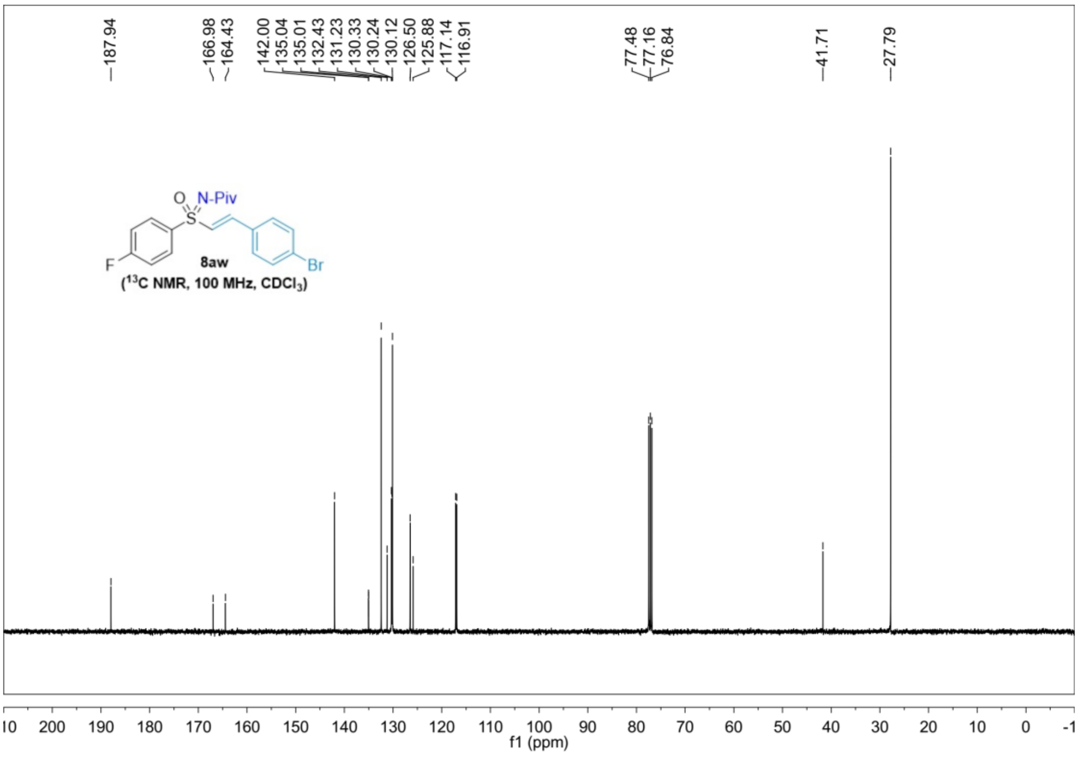
187.94
166.98
164.43
142.00
135.04
135.01
132.43
131.23
130.33
130.24
130.12
126.50
125.88
117.14
116.91
77.48
77.16
76.84
41.71
27.79
N-Piv
O
S
F
Br
8aw
(13C NMR, 100 MHz, CDCl3)
f1 (ppm)

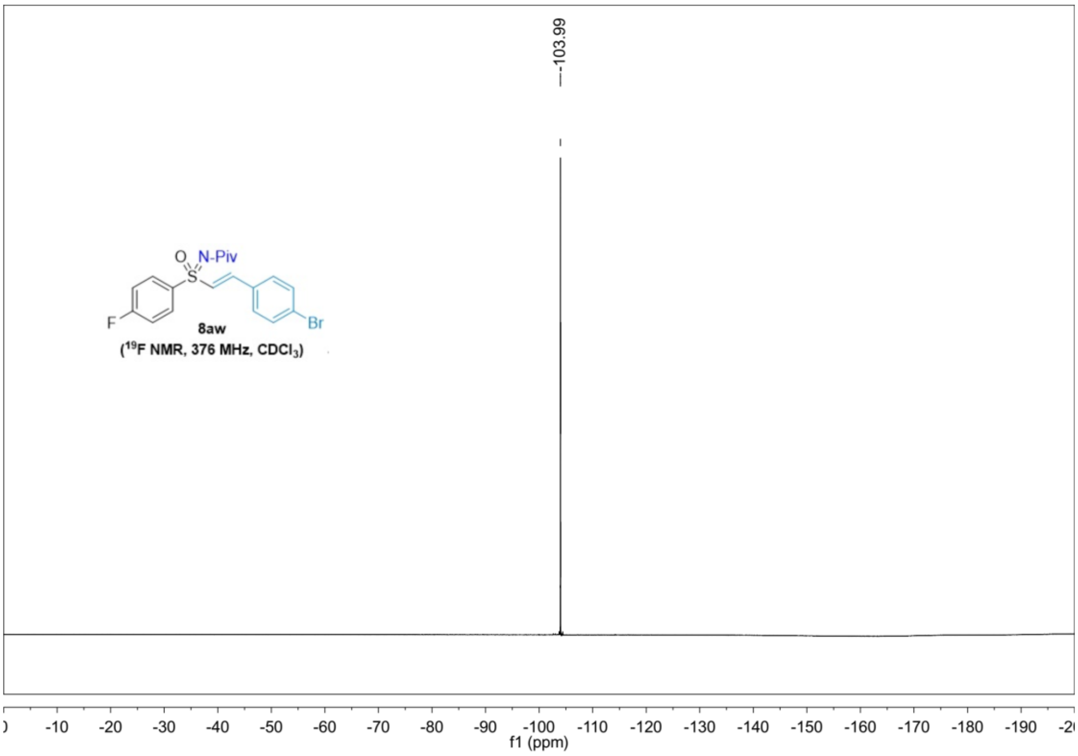

-103.99
O N-Piv
S
F
Br
8aw
(^{19}F NMR, 376 MHz, CDCl$_3$)
0 -10 -20 -30 -40 -50 -60 -70 -80 -90 -100 -110 -120 -130 -140 -150 -160 -170 -180 -190
f1 (ppm)

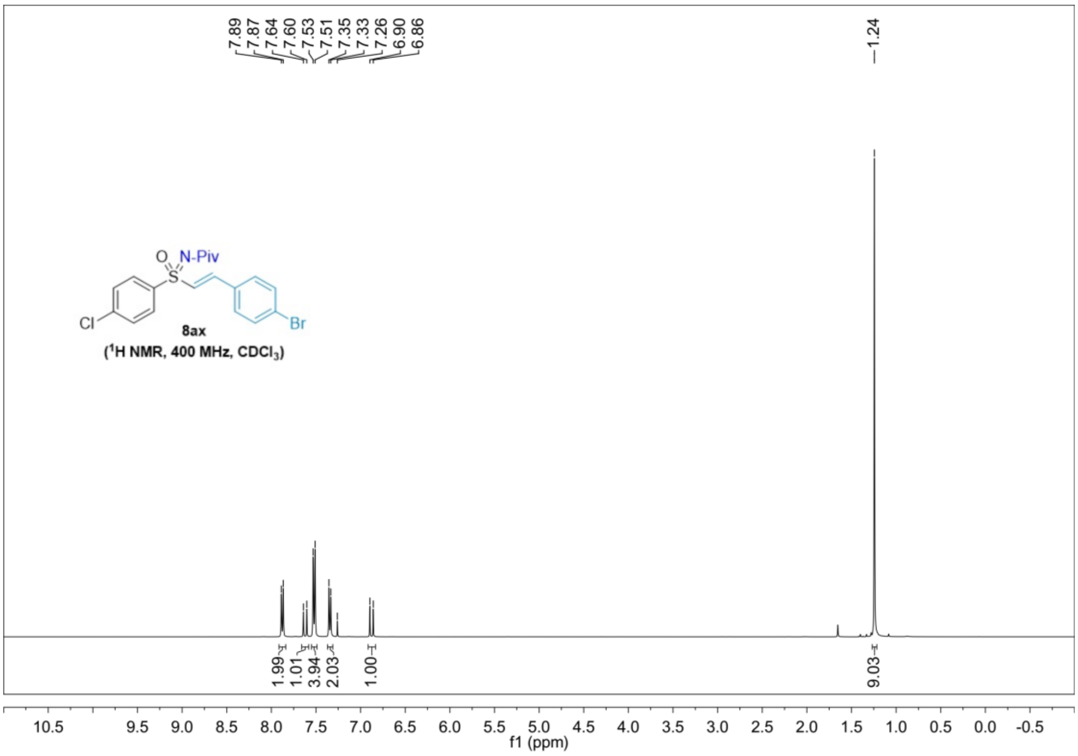

7.89 7.87 7.64 7.60 7.53 7.51 7.35 7.33 7.26 6.90 6.86
1.24
O N-Piv
S
Cl
Br
8ax
(^{1}H NMR, 400 MHz, CDCl$_3$)
1.99 1.01 3.94 2.03 1.00
9.03
10.5 9.5 9.0 8.5 8.0 7.5 7.0 6.5 6.0 5.5 5.0 4.5 4.0 3.5 3.0 2.5 2.0 1.5 1.0 0.5 0.0 -0.5
f1 (ppm)

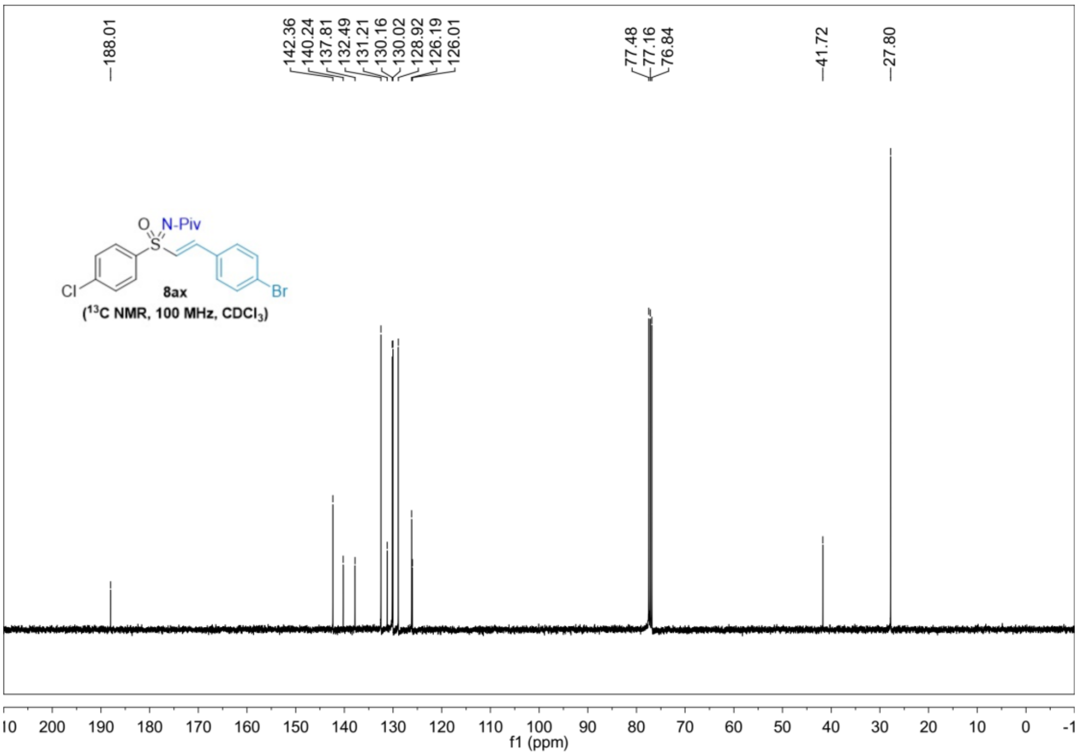
188.01
142.36
140.24
137.81
132.49
131.21
130.16
130.02
128.92
126.19
126.01
77.48
77.16
76.84
41.72
27.80
O
N-Piv
S
Cl
Br
8ax
(^{13}C NMR, 100 MHz, CDCl$_3$)
f1 (ppm)

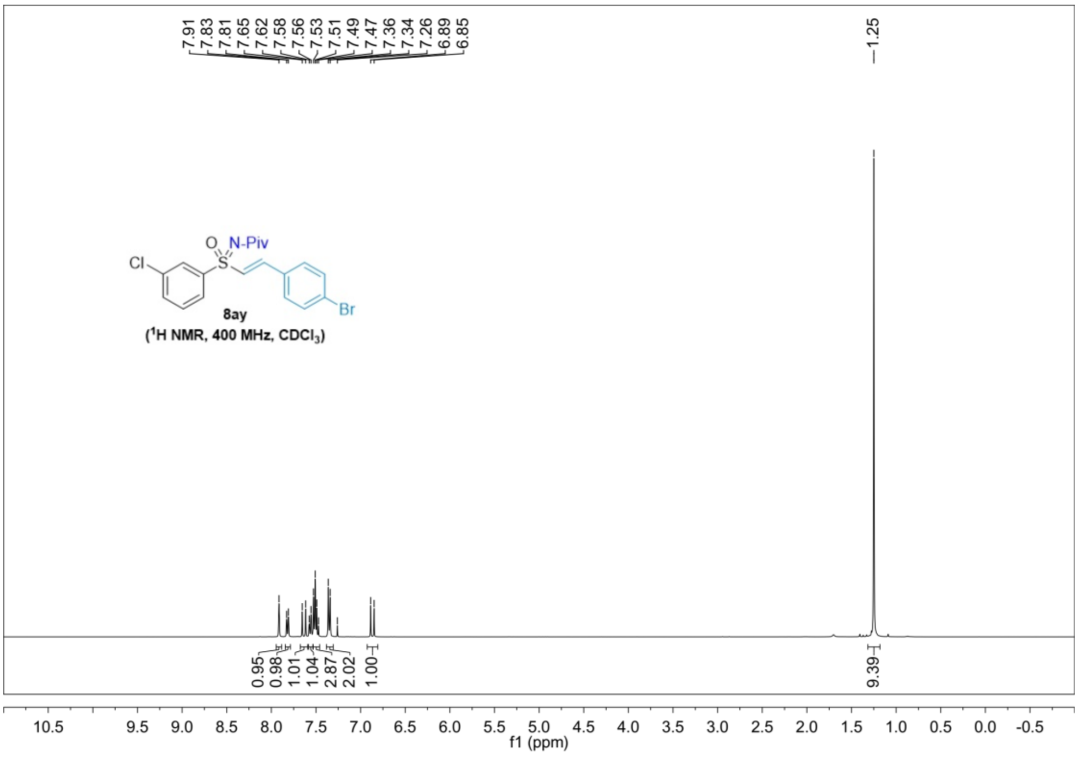
7.91
7.83
7.81
7.65
7.62
7.58
7.56
7.53
7.51
7.49
7.47
7.36
7.34
7.26
6.89
6.85
1.25
O
N-Piv
Cl
S
Br
8ay
(^{1}H NMR, 400 MHz, CDCl$_3$)
0.95
0.98
1.01
1.04
2.87
2.02
1.00
9.39
f1 (ppm)

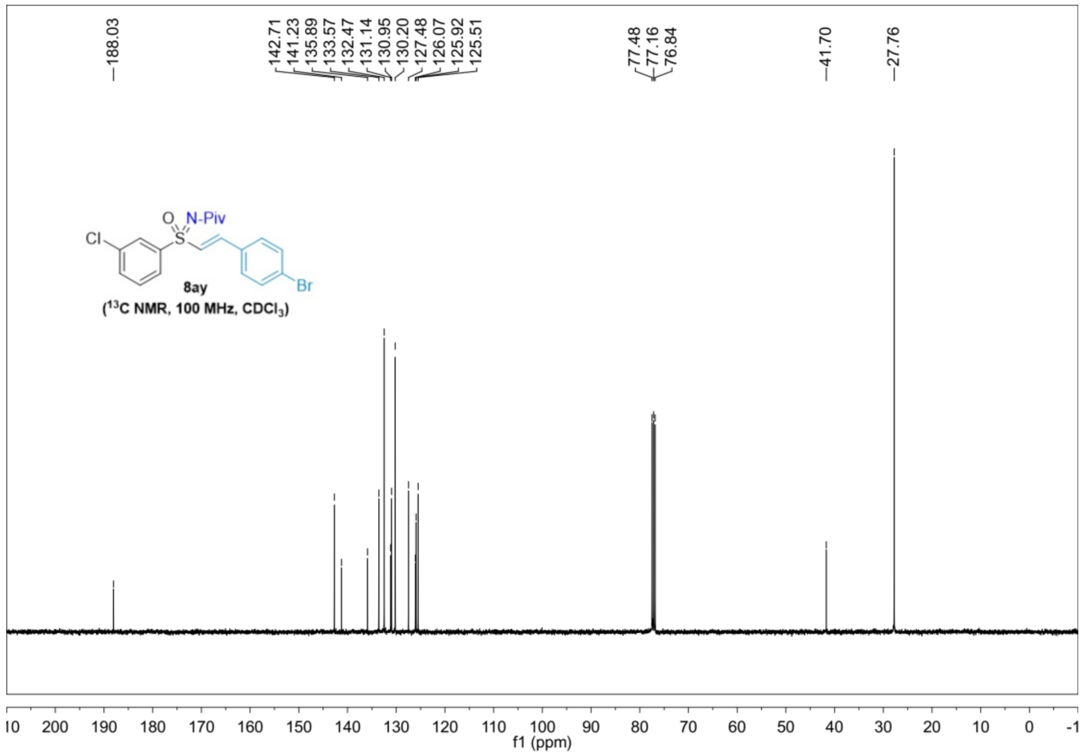
188.03
142.71
141.23
135.89
133.57
132.47
131.14
130.95
130.20
127.48
126.07
125.92
125.51
77.48
77.16
76.84
41.70
27.76
O
N-Piv
S
Cl
Br
8ay
(^{13}C NMR, 100 MHz, CDCl$_3$)
f1 (ppm)

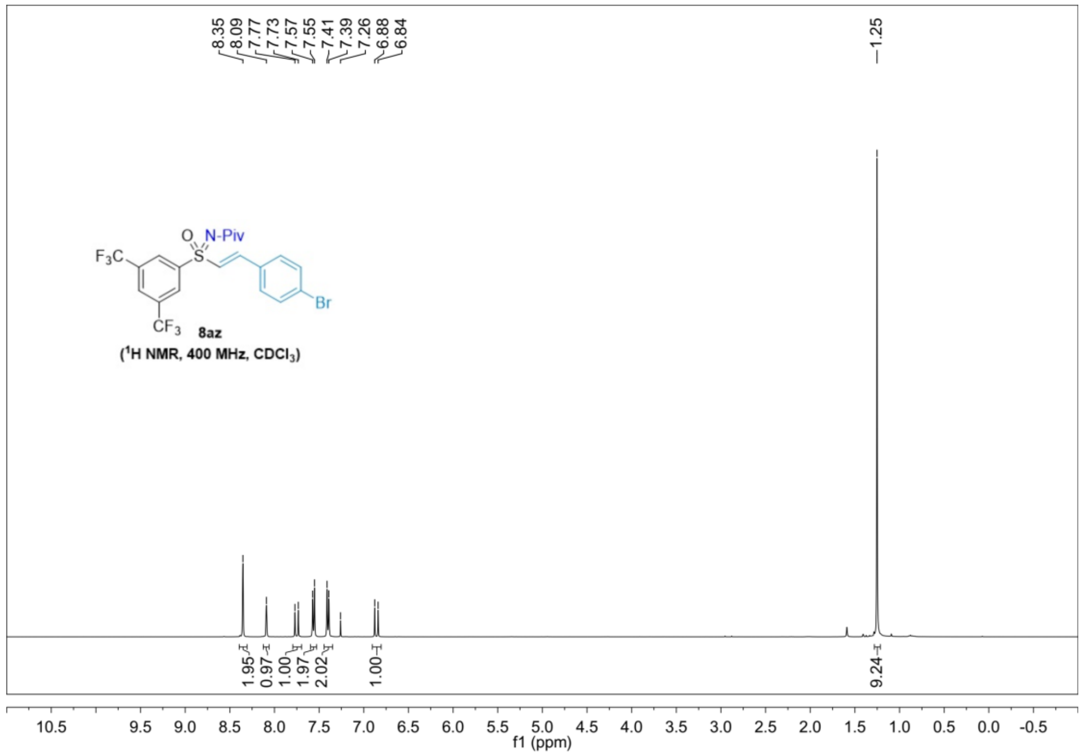
8.35
8.09
7.77
7.73
7.57
7.55
7.41
7.39
7.26
6.88
6.84
1.25
O
N-Piv
S
F$_3$C
CF$_3$
Br
8az
(^{1}H NMR, 400 MHz, CDCl$_3$)
1.95
0.97
1.00
1.97
2.02
1.00
9.24
f1 (ppm)

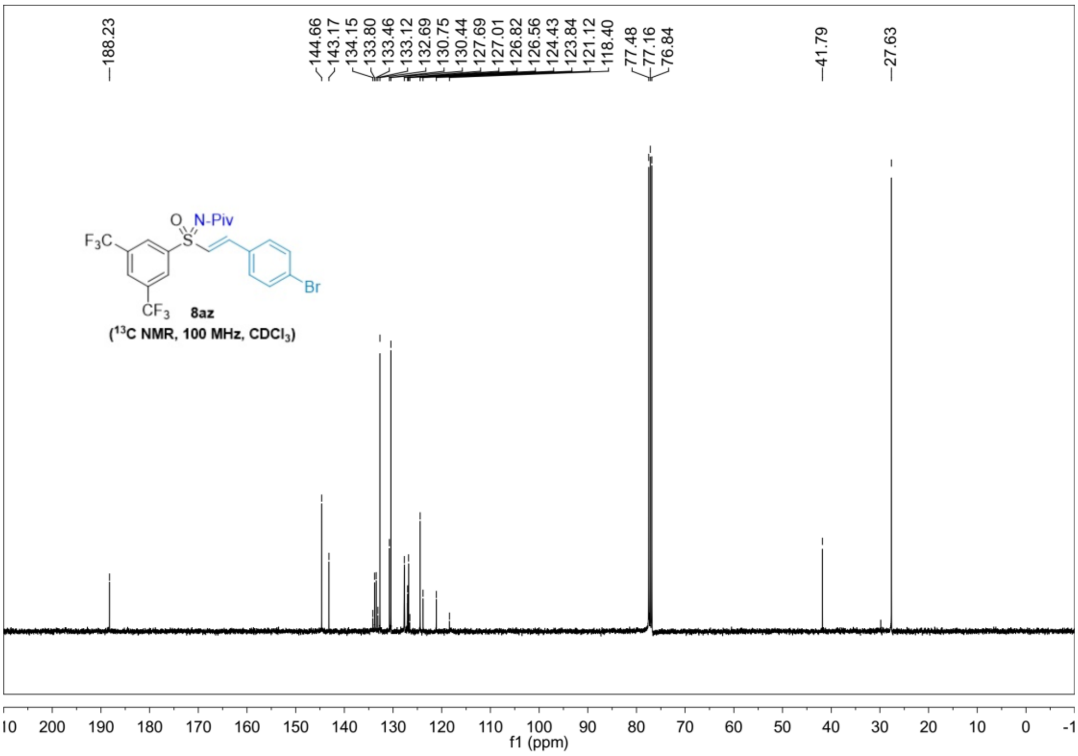
188.23
144.66
143.17
134.15
133.80
133.46
133.12
132.69
130.75
130.44
127.69
127.01
126.82
126.56
124.43
123.84
121.12
118.40
77.48
77.16
76.84
41.79
27.63
N-Piv
O
S
F3C
CF3
Br
8az
(13C NMR, 100 MHz, CDCl3)
f1 (ppm)

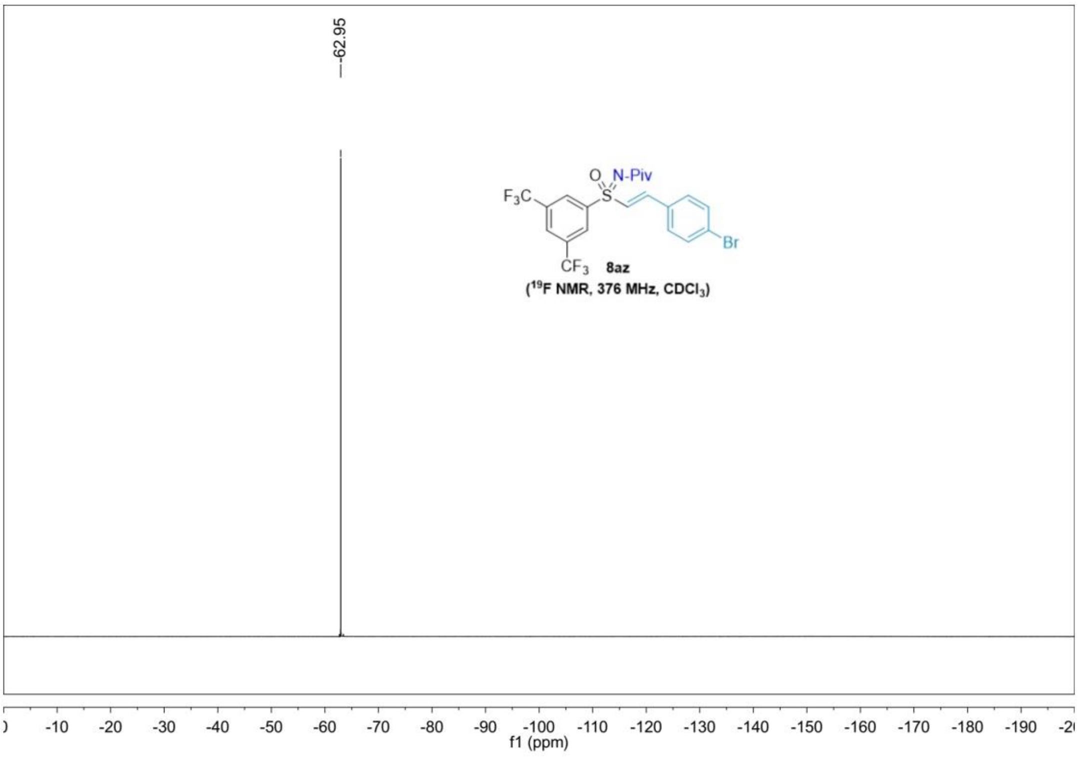
-62.95
N-Piv
O
S
F3C
CF3
Br
8az
(19F NMR, 376 MHz, CDCl3)
f1 (ppm)

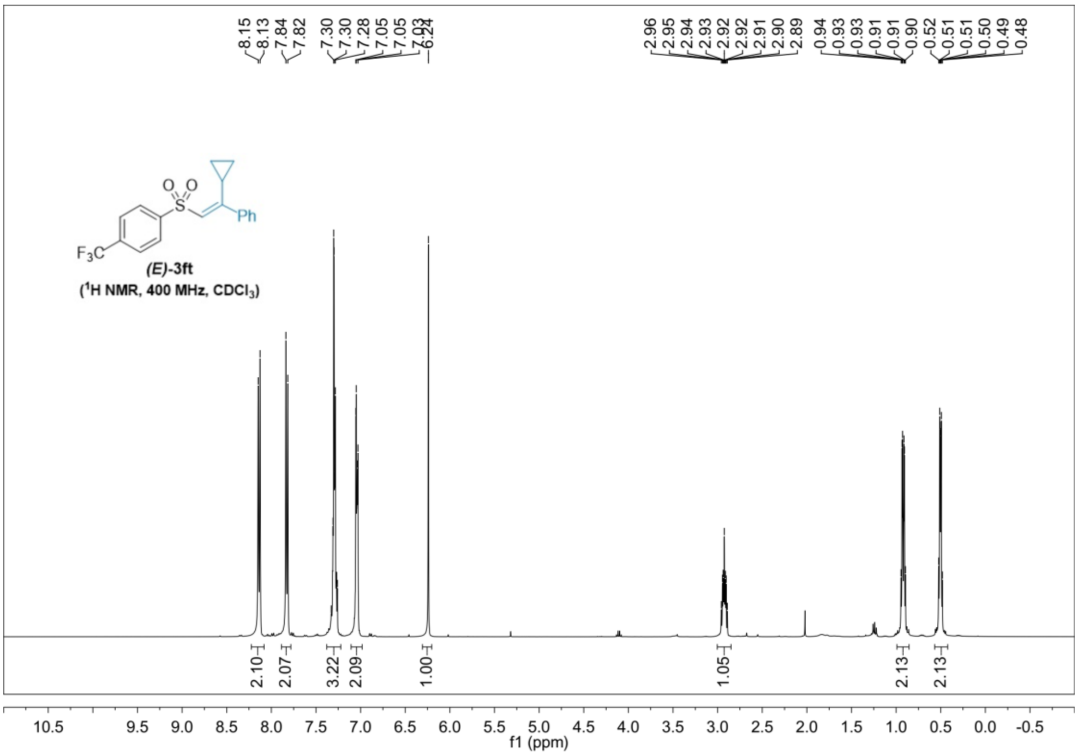
(E)-3ft
(¹H NMR, 400 MHz, CDCl₃)
f1 (ppm)

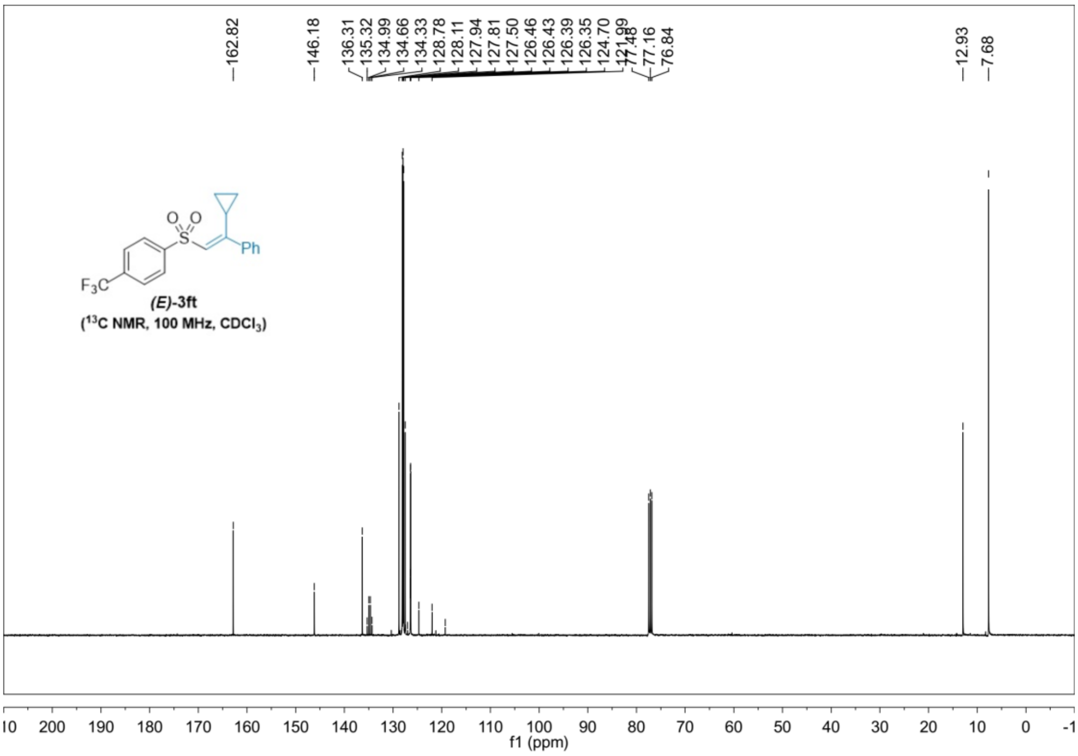
(E)-3ft
(¹³C NMR, 100 MHz, CDCl₃)
f1 (ppm)

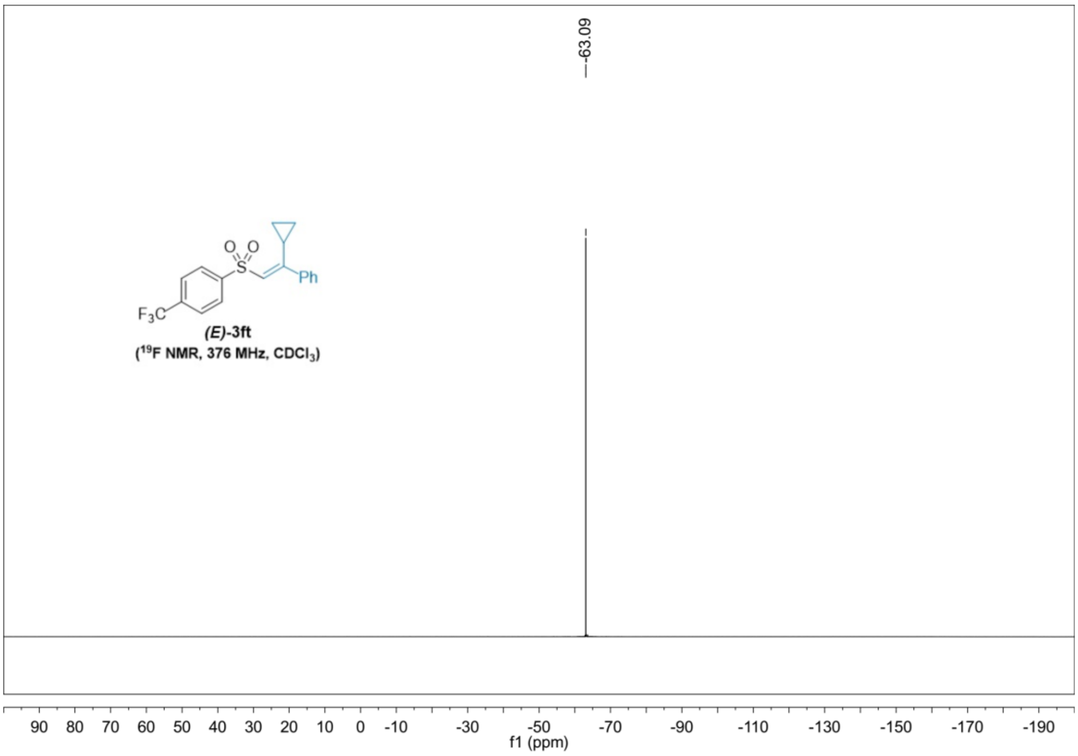

-63.09
(E)-3ft
(^{19}F NMR, 376 MHz, CDCl$_3$)
f1 (ppm)

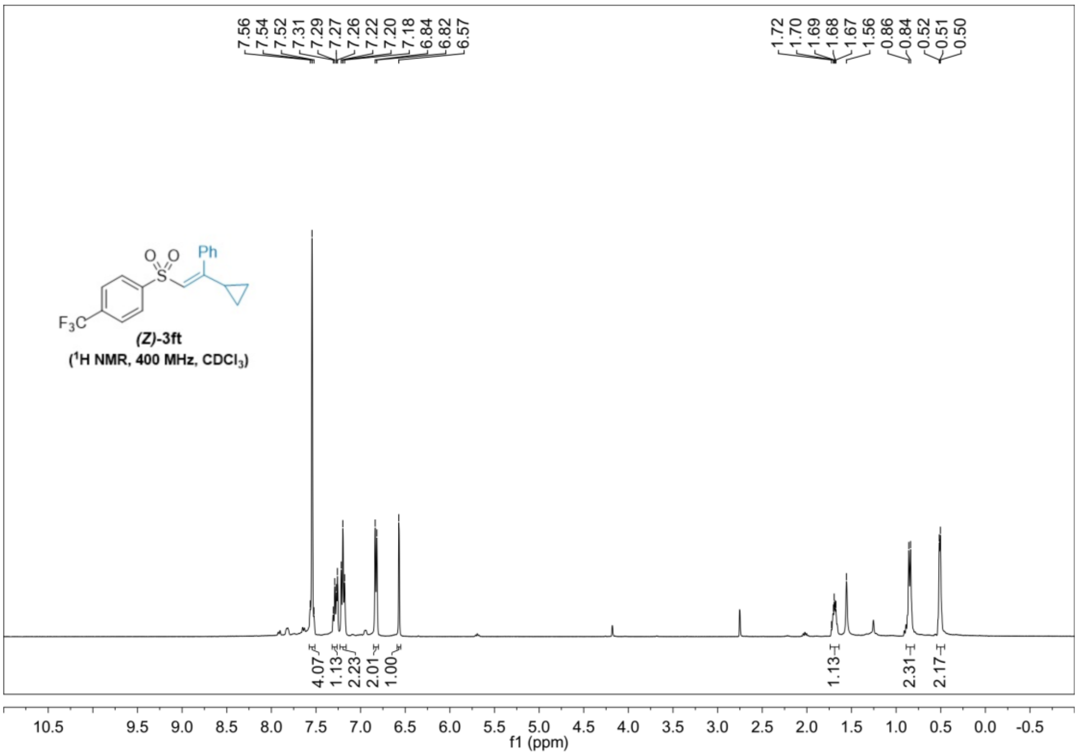

7.56 7.54 7.52 7.31 7.29 7.27 7.26 7.22 7.20 7.18 6.84 6.82 6.57
1.72 1.70 1.69 1.68 1.67 1.56 0.86 0.84 0.52 0.51 0.50
(Z)-3ft
(^{1}H NMR, 400 MHz, CDCl$_3$)
4.07 1.13 2.23 2.01 1.00 1.13 2.31 2.17
f1 (ppm)

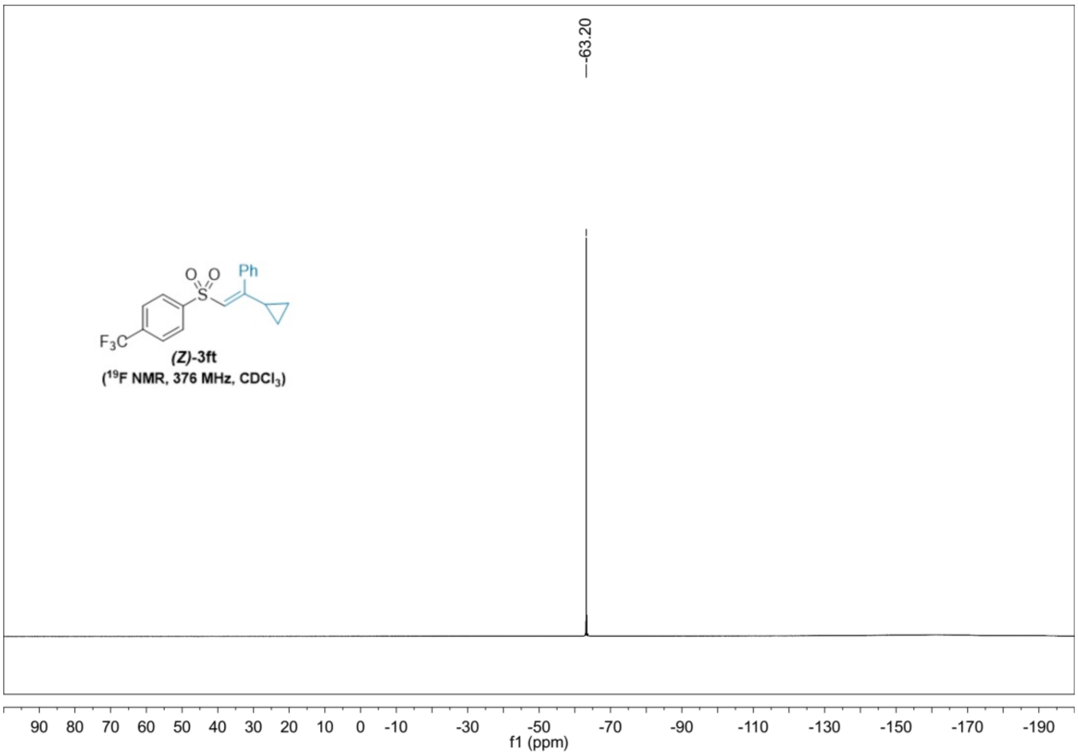

-63.20
F3C
Ph
(Z)-3ft
(19F NMR, 376 MHz, CDCl3)
90 80 70 60 50 40 30 20 10 0 -10 -30 -50 -70 -90 -110 -130 -150 -170 -190
f1 (ppm)

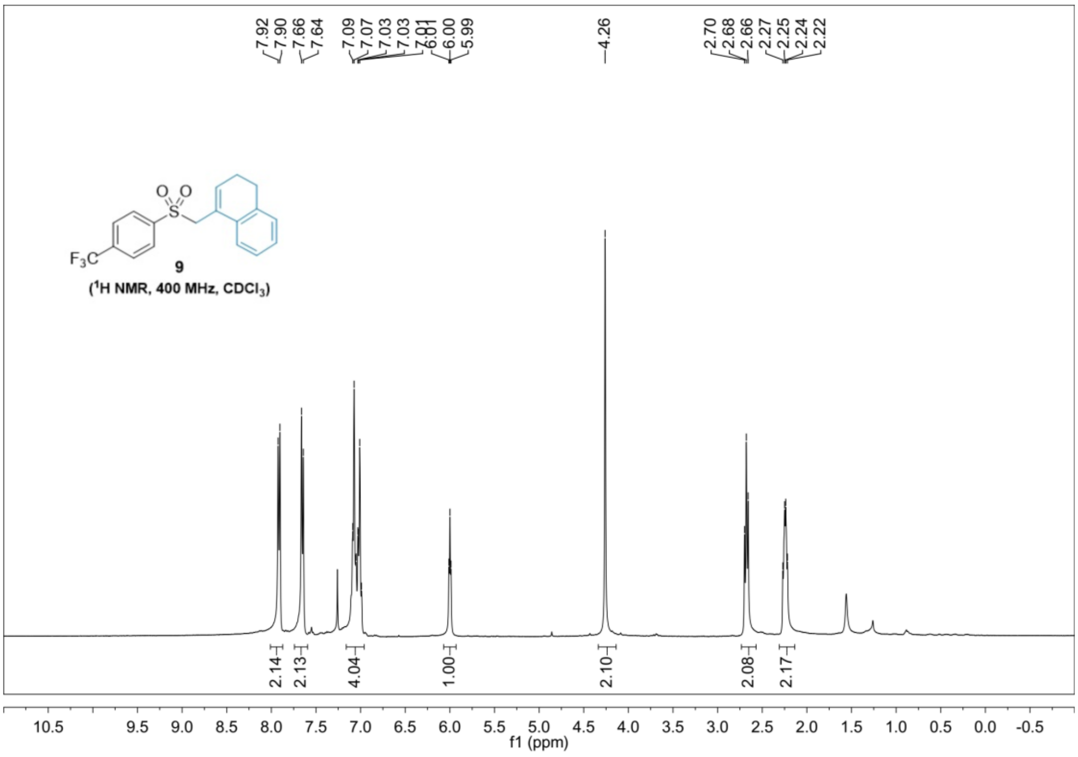

7.92 7.90 7.66 7.64 7.09 7.07 7.03 7.03 7.01 6.00 5.99
4.26
2.70 2.68 2.66 2.27 2.25 2.24 2.22
F3C
9
(1H NMR, 400 MHz, CDCl3)
2.14 2.13 4.04 1.00 2.10 2.08 2.17
10.5 9.5 9.0 8.5 8.0 7.5 7.0 6.5 6.0 5.5 5.0 4.5 4.0 3.5 3.0 2.5 2.0 1.5 1.0 0.5 0.0 -0.5
f1 (ppm)

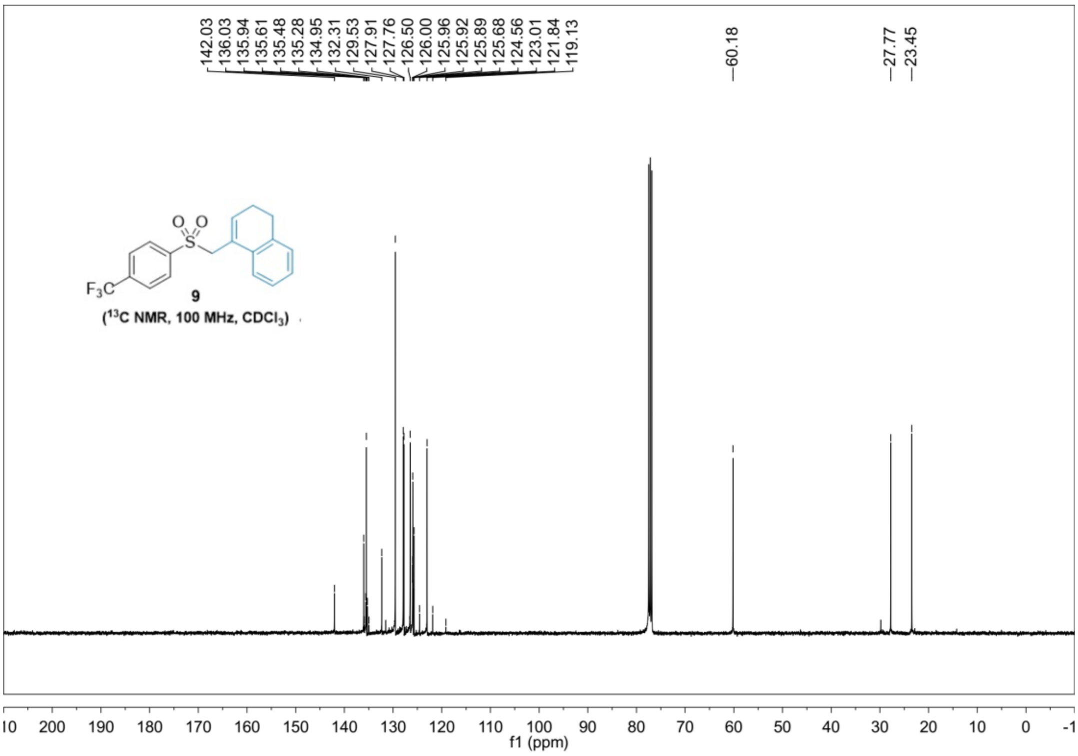
142.03
136.03
135.94
135.61
135.48
135.28
134.95
132.31
129.53
127.91
127.76
126.50
126.00
125.96
125.92
125.89
125.68
124.56
123.01
121.84
119.13
60.18
27.77
23.45
F3C
9
(13C NMR, 100 MHz, CDCl3)
f1 (ppm)

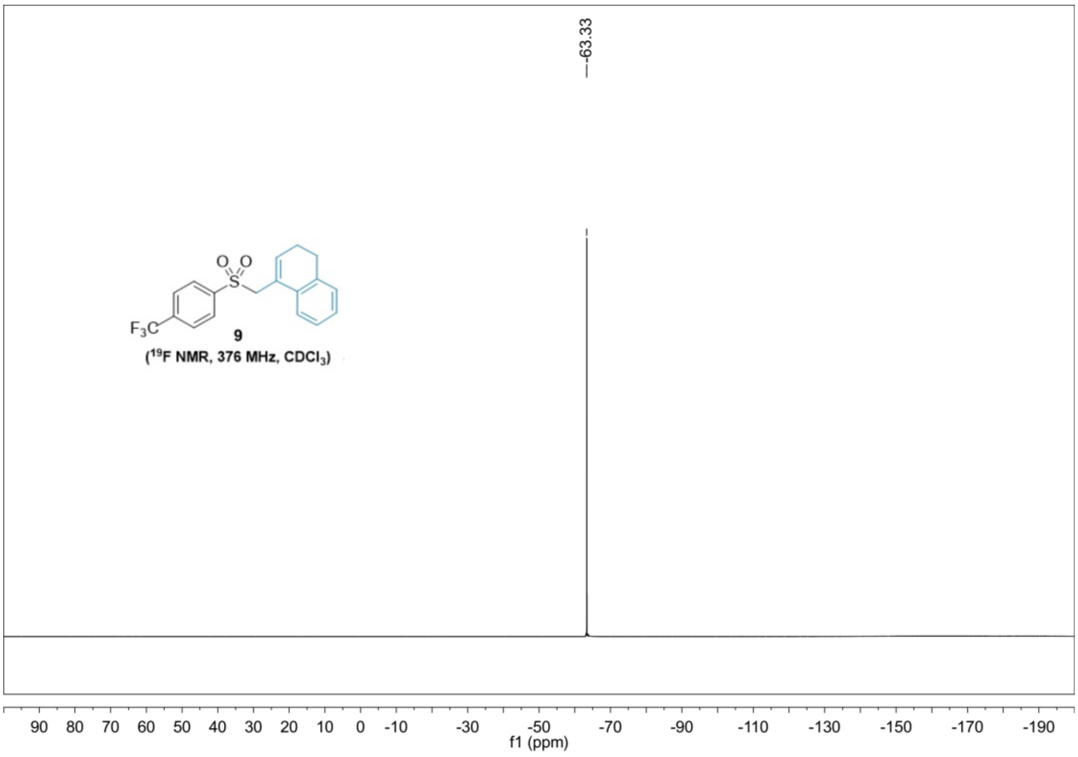
-63.33
F3C
9
(19F NMR, 376 MHz, CDCl3)
f1 (ppm)

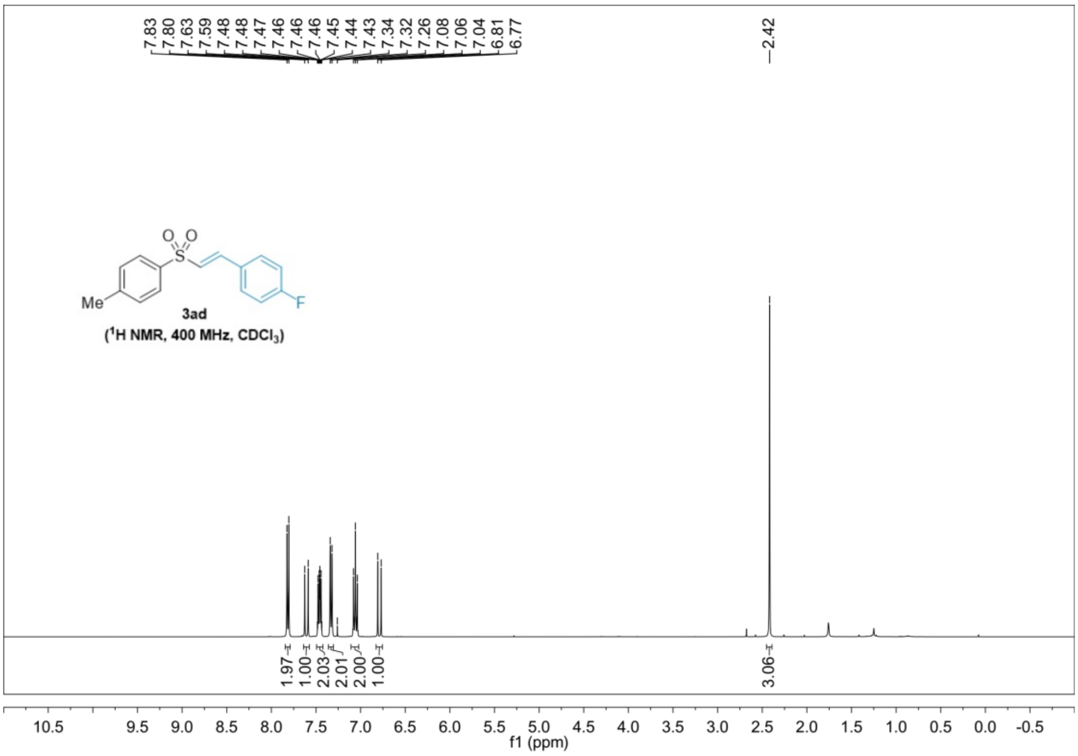
7.83
7.80
7.63
7.59
7.48
7.48
7.47
7.46
7.46
7.46
7.45
7.44
7.43
7.34
7.32
7.26
7.08
7.06
7.04
6.81
6.77
2.42
Me
F
3ad
(¹H NMR, 400 MHz, CDCl₃)
1.97
1.00
2.03
2.01
2.00
1.00
3.06
f1 (ppm)

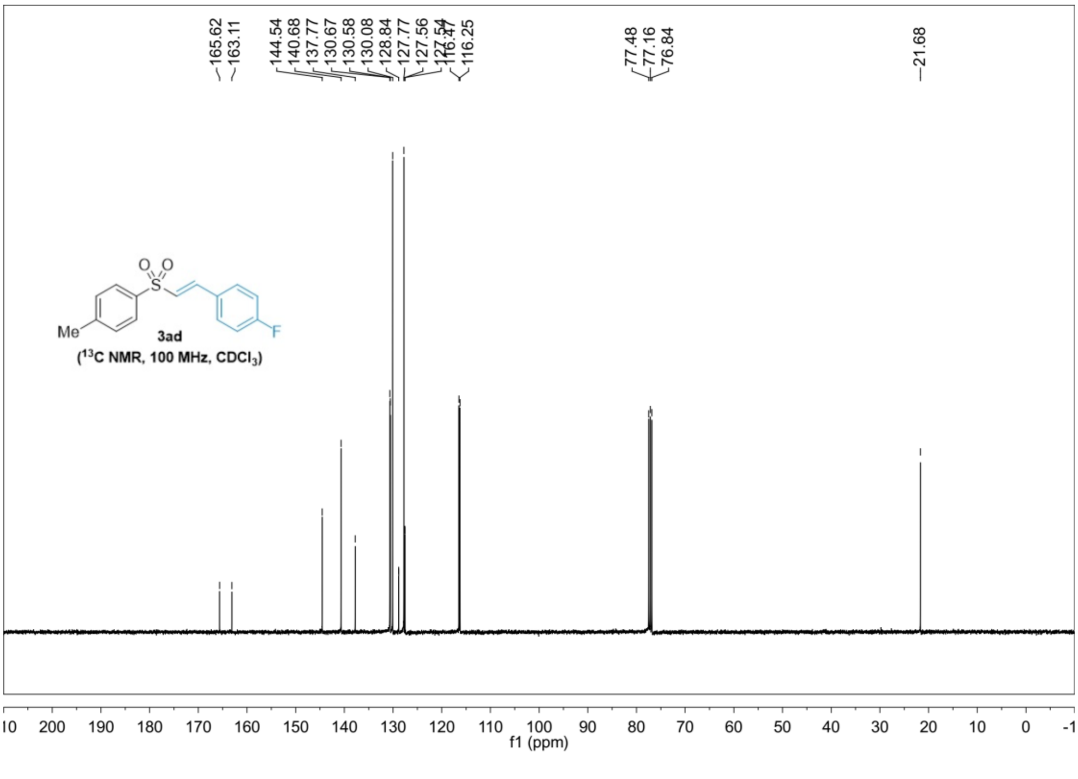
165.62
163.11
144.54
140.68
137.77
130.67
130.58
130.08
128.84
127.77
127.56
127.54
116.47
116.25
77.48
77.16
76.84
21.68
Me
F
3ad
(¹³C NMR, 100 MHz, CDCl₃)
f1 (ppm)

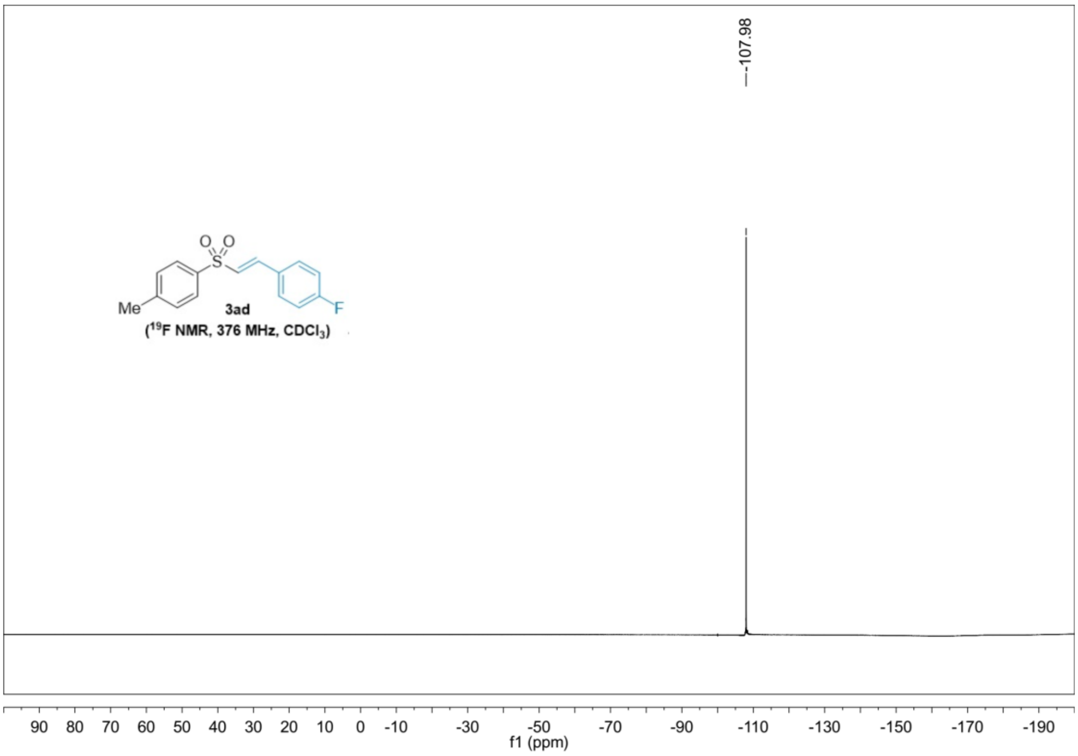
-107.98
Me
3ad
(19F NMR, 376 MHz, CDCl3)
F
90 80 70 60 50 40 30 20 10 0 -10 -30 -50 -70 -90 -110 -130 -150 -170 -190
f1 (ppm)

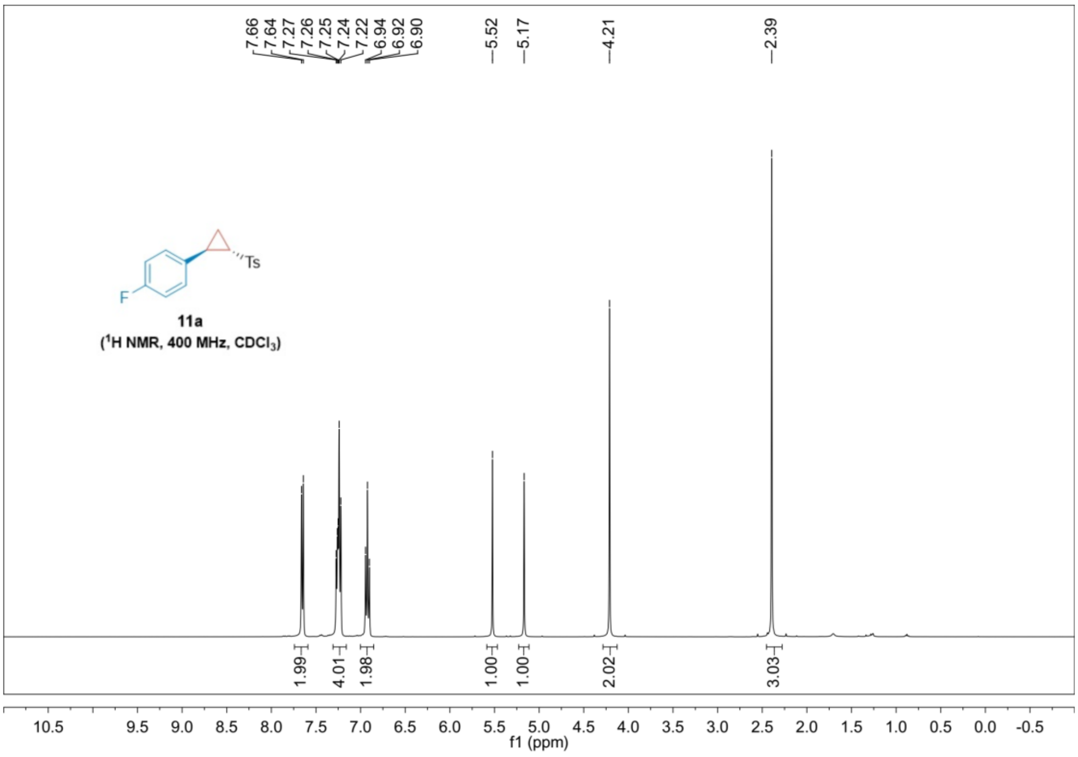
7.66 7.64 7.27 7.26 7.25 7.24 7.22 6.94 6.92 6.90
5.52
5.17
4.21
2.39
Ts
F
11a
(1H NMR, 400 MHz, CDCl3)
1.99 4.01 1.98 1.00 1.00 2.02 3.03
10.5 9.5 9.0 8.5 8.0 7.5 7.0 6.5 6.0 5.5 5.0 4.5 4.0 3.5 3.0 2.5 2.0 1.5 1.0 0.5 0.0 -0.5
f1 (ppm)

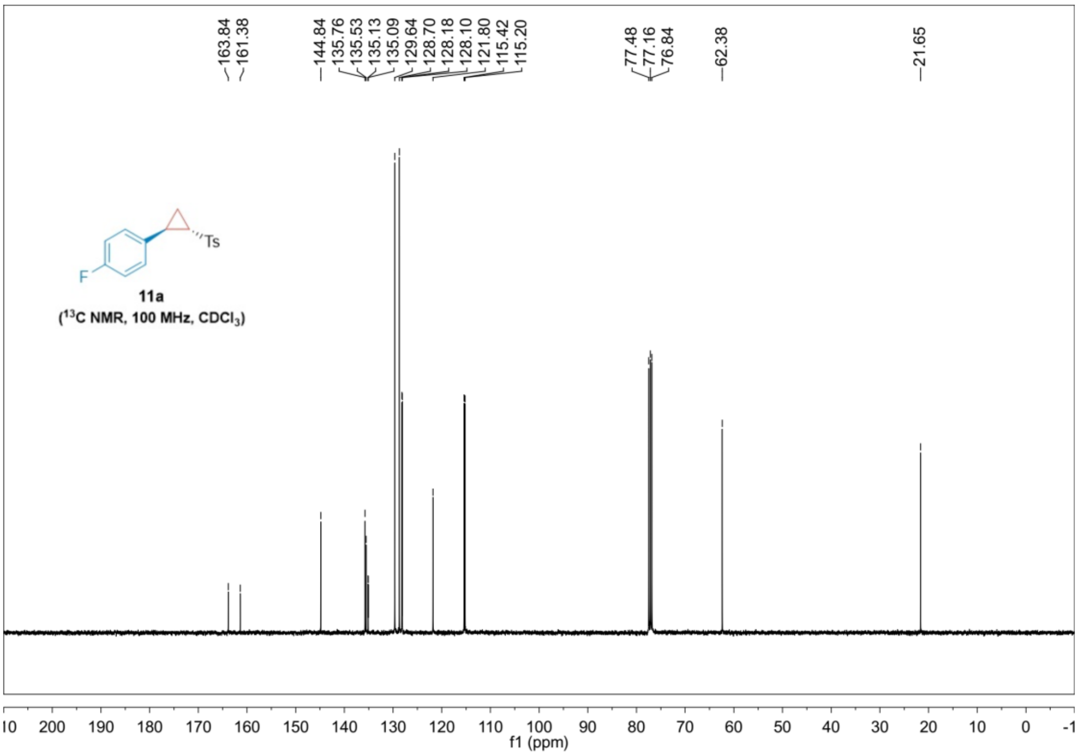
163.84
161.38
144.84
135.76
135.53
135.13
135.09
129.64
128.70
128.18
128.10
121.80
115.42
115.20
77.48
77.16
76.84
62.38
21.65
Ts
F
11a
(13C NMR, 100 MHz, CDCl3)
f1 (ppm)

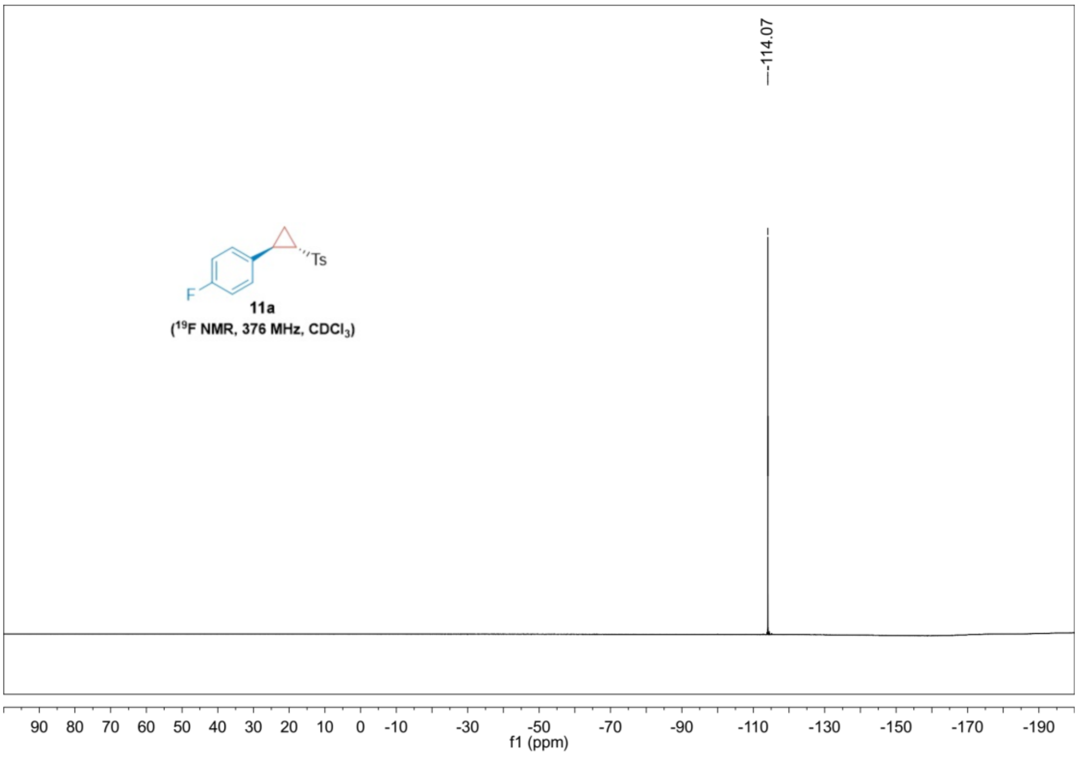
-114.07
Ts
F
11a
(19F NMR, 376 MHz, CDCl3)
f1 (ppm)

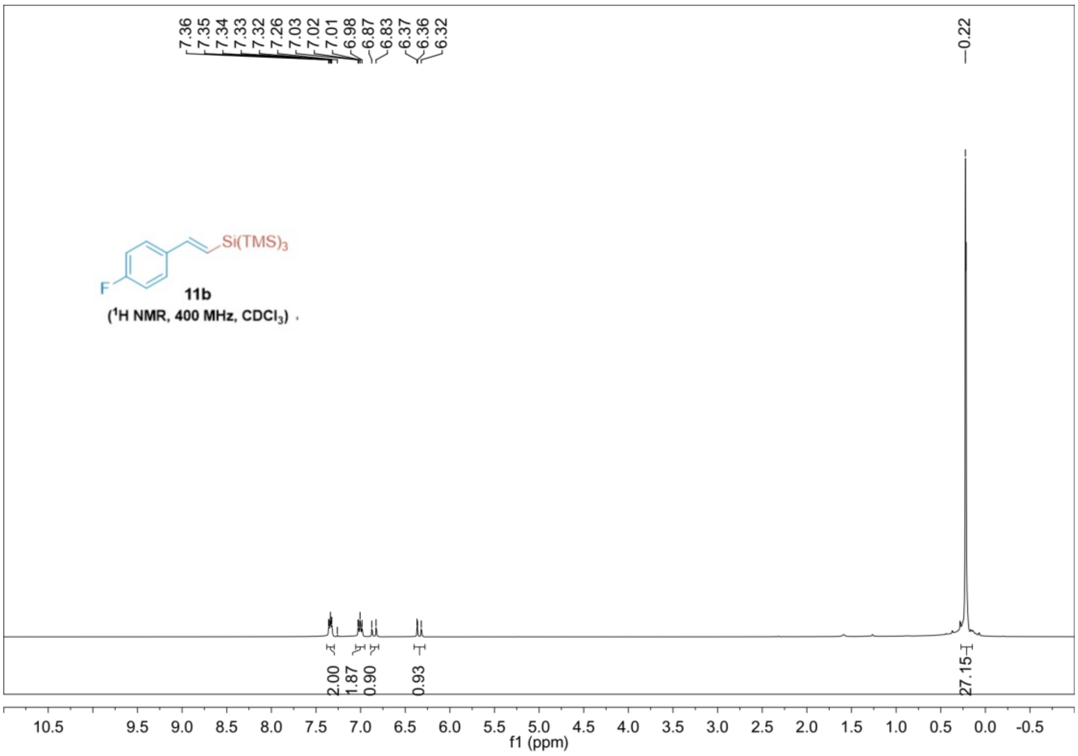
7.36
7.35
7.34
7.33
7.32
7.26
7.03
7.02
7.01
6.98
6.87
6.83
6.37
6.36
6.32
0.22
Si(TMS)3
F
11b
(1H NMR, 400 MHz, CDCl3)
2.00
1.87
0.90
0.93
27.15
10.5
9.5
9.0
8.5
8.0
7.5
7.0
6.5
6.0
5.5
5.0
4.5
4.0
3.5
3.0
2.5
2.0
1.5
1.0
0.5
0.0
-0.5
f1 (ppm)

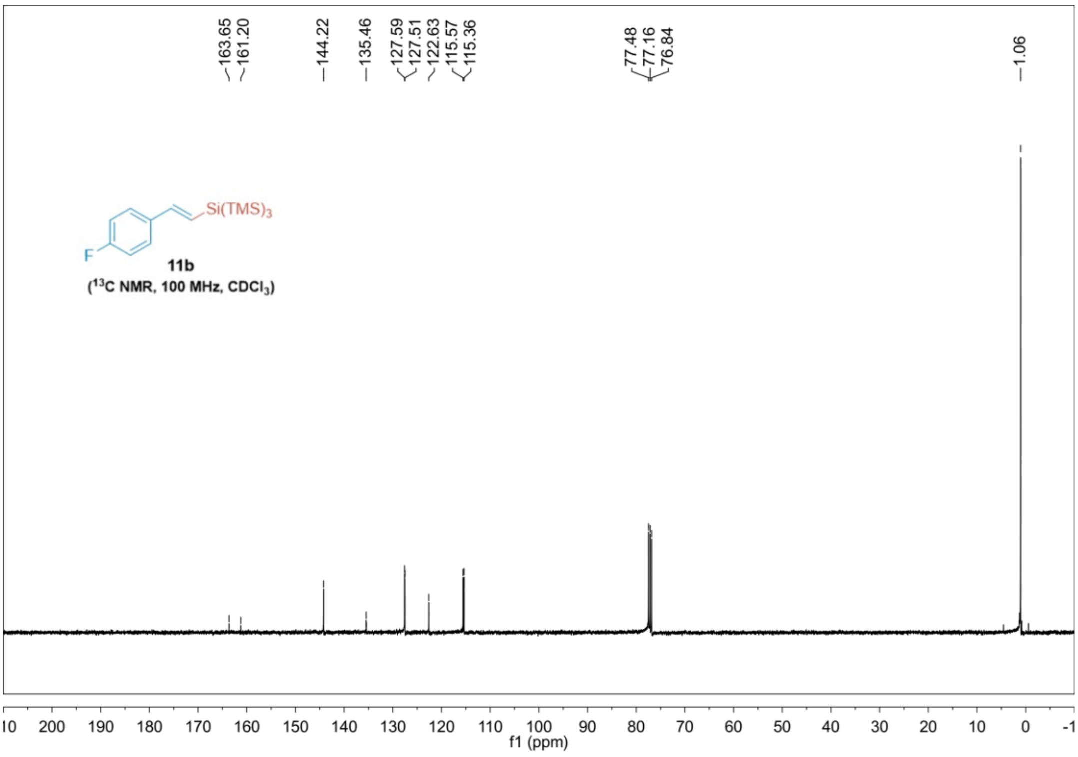
163.65
161.20
144.22
135.46
127.59
127.51
122.63
115.57
115.36
77.48
77.16
76.84
1.06
Si(TMS)3
F
11b
(13C NMR, 100 MHz, CDCl3)
200
190
180
170
160
150
140
130
120
110
100
90
80
70
60
50
40
30
20
10
0
f1 (ppm)

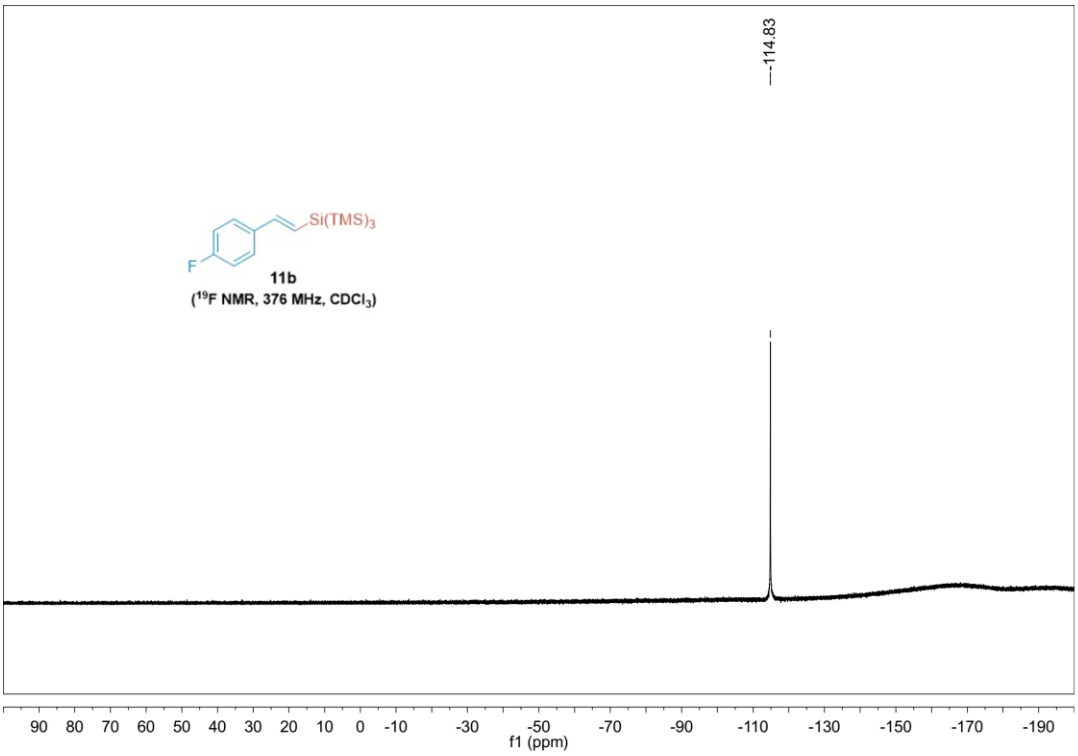

-114.83
Si(TMS)3
F
11b
(19F NMR, 376 MHz, CDCl3)
90 80 70 60 50 40 30 20 10 0 -10 -30 -50 -70 -90 -110 -130 -150 -170 -190
f1 (ppm)

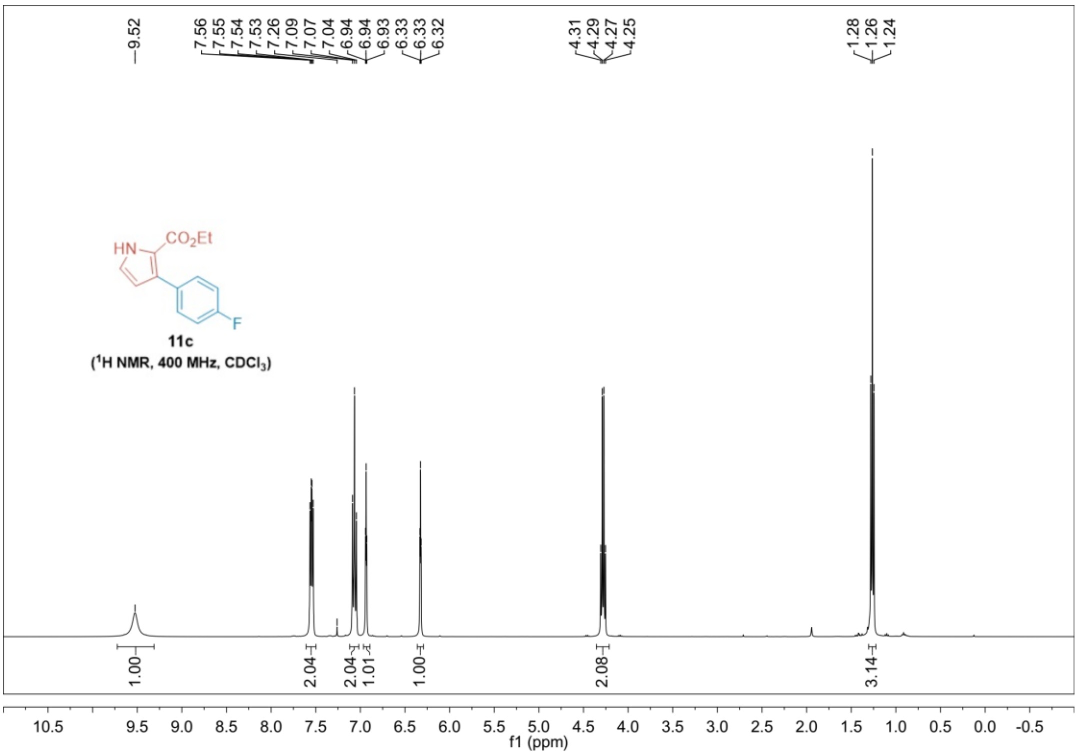

9.52
7.56 7.55 7.54 7.53 7.26 7.09 7.07 7.04 6.94 6.93 6.33 6.33 6.32
4.31 4.29 4.27 4.25
1.28 1.26 1.24
HN
CO2Et
F
11c
(1H NMR, 400 MHz, CDCl3)
1.00 2.04 2.04 1.01 1.00 2.08 3.14
10.5 9.5 9.0 8.5 8.0 7.5 7.0 6.5 6.0 5.5 5.0 4.5 4.0 3.5 3.0 2.5 2.0 1.5 1.0 0.5 0.0 -0.5
f1 (ppm)

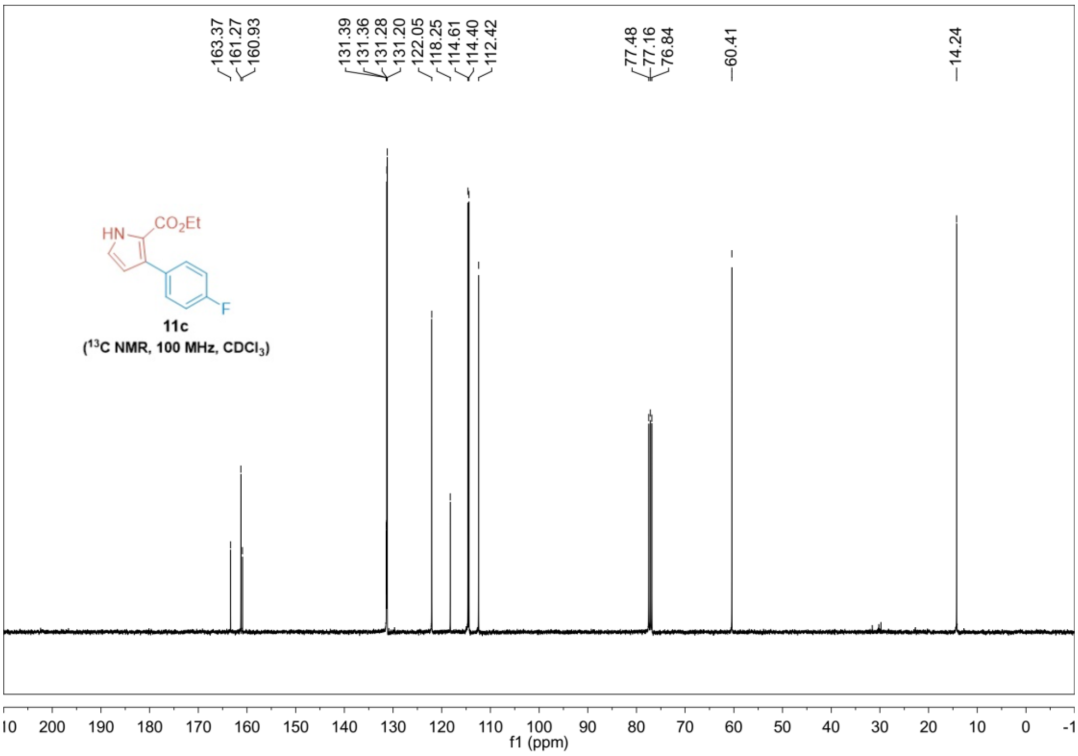

163.37
161.27
160.93
131.39
131.36
131.28
131.20
122.05
118.25
114.61
114.40
112.42
77.48
77.16
76.84
60.41
14.24
HN
CO2Et
F
11c
(13C NMR, 100 MHz, CDCl3)
f1 (ppm)

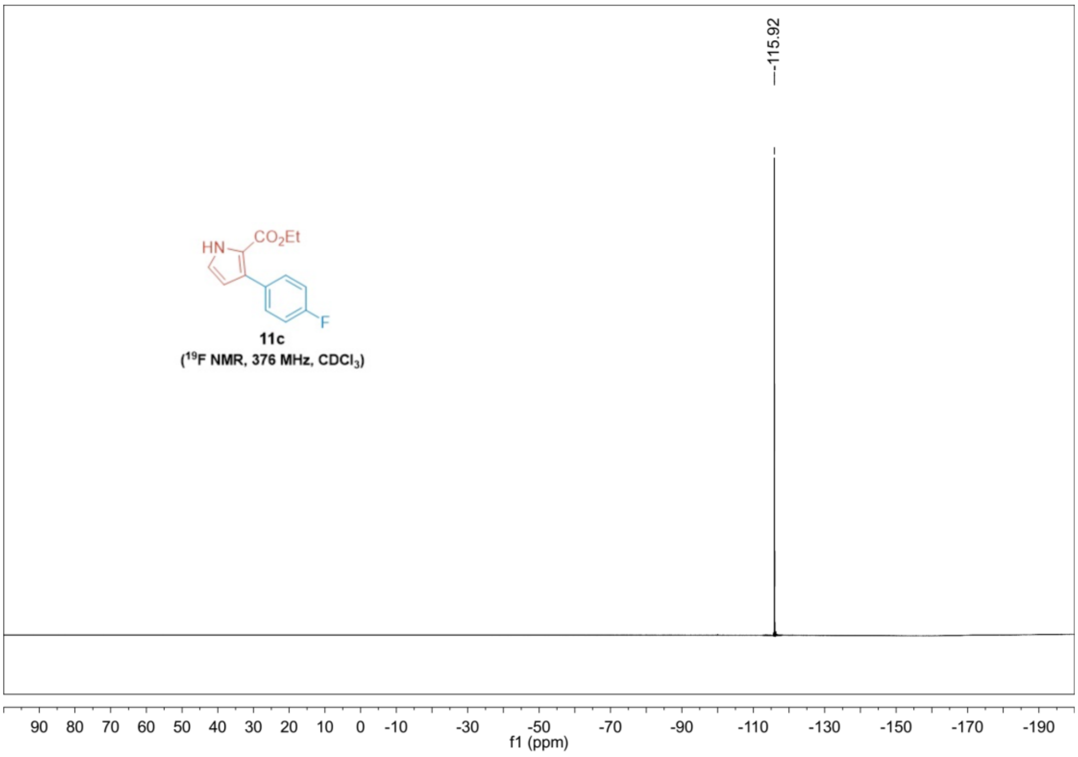

-115.92
HN
CO2Et
F
11c
(19F NMR, 376 MHz, CDCl3)
f1 (ppm)

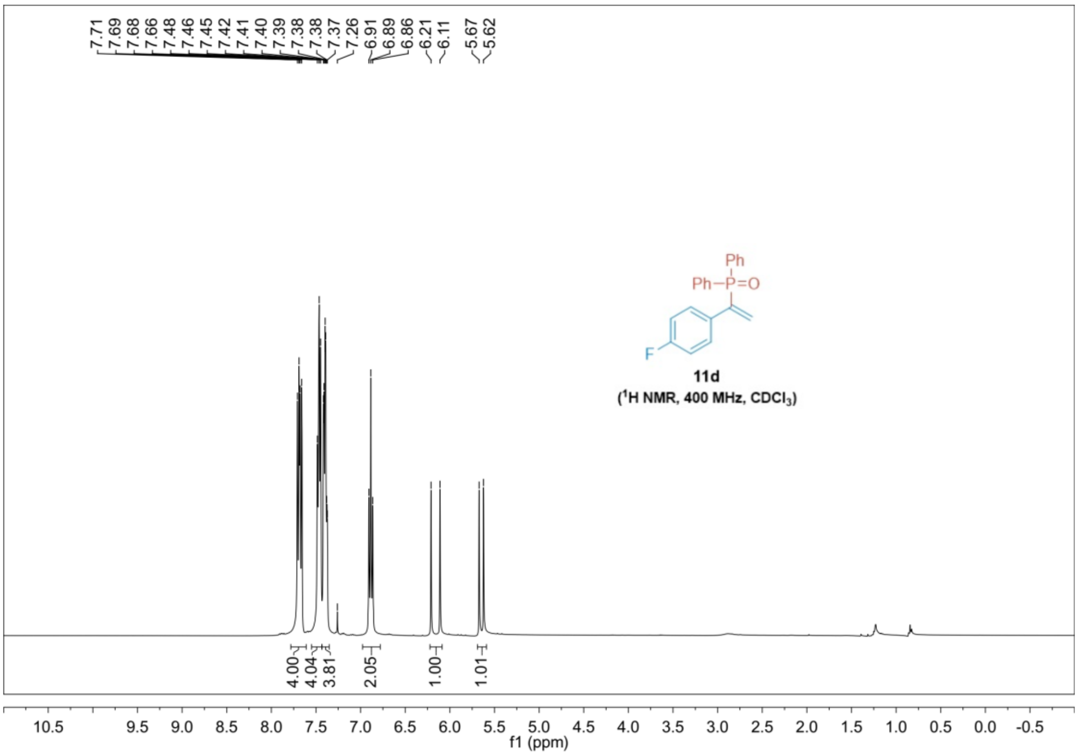

7.71
7.69
7.68
7.66
7.48
7.46
7.45
7.42
7.41
7.40
7.39
7.38
7.38
7.37
7.26
6.91
6.89
6.86
6.21
6.11
5.67
5.62
Ph
Ph–P=O
F
11d
(1H NMR, 400 MHz, CDCl3)
4.00
4.04
3.81
2.05
1.00
1.01
f1 (ppm)

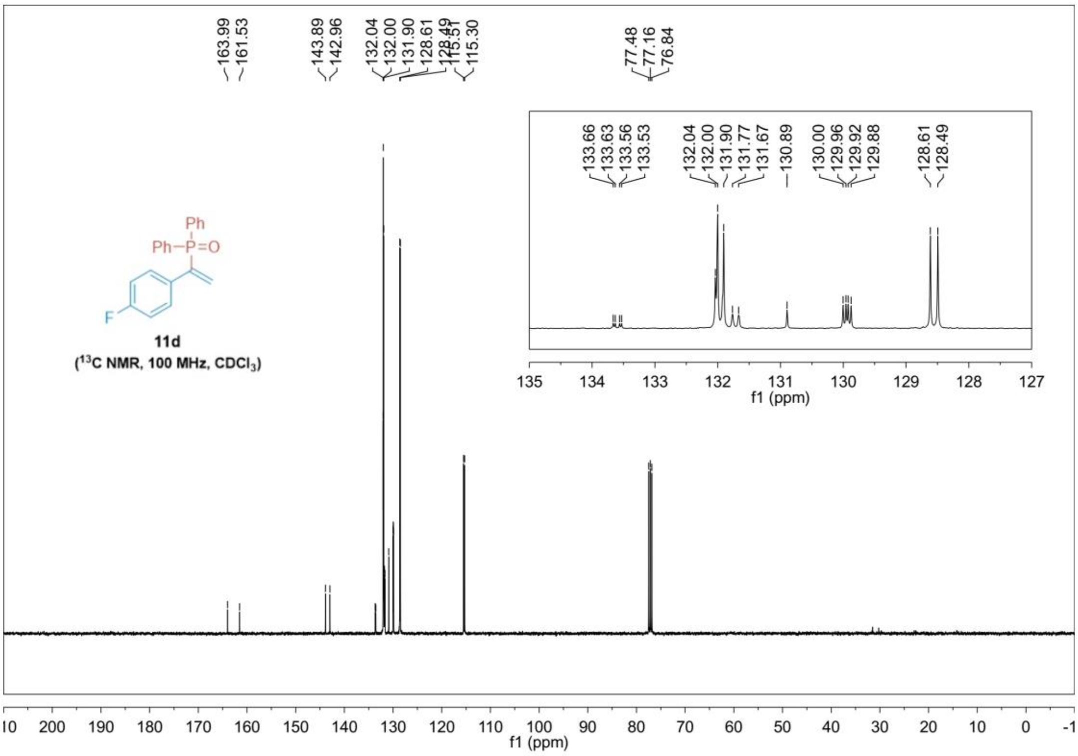

163.99
161.53
143.89
142.96
132.04
132.00
131.90
128.61
128.49
115.51
115.30
77.48
77.16
76.84
133.66
133.63
133.56
133.53
131.77
131.67
130.89
130.00
129.96
129.92
129.88
Ph
Ph–P=O
F
11d
(13C NMR, 100 MHz, CDCl3)
f1 (ppm)

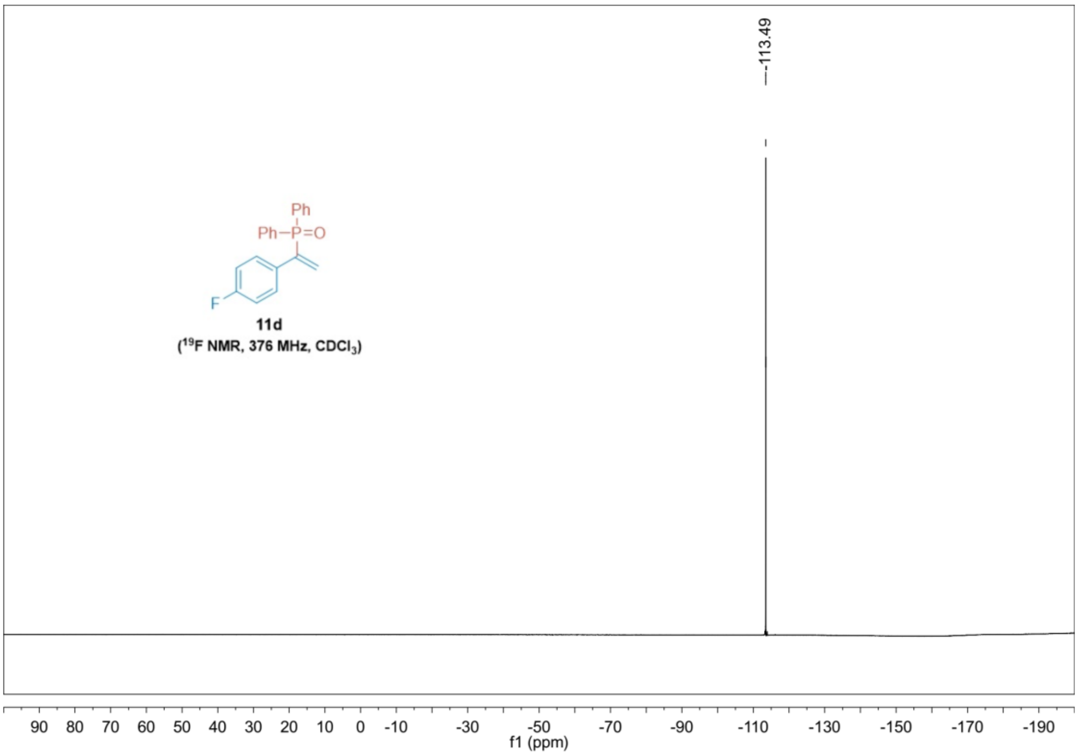

11d
(^{19}F NMR, 376 MHz, CDCl$_3$)

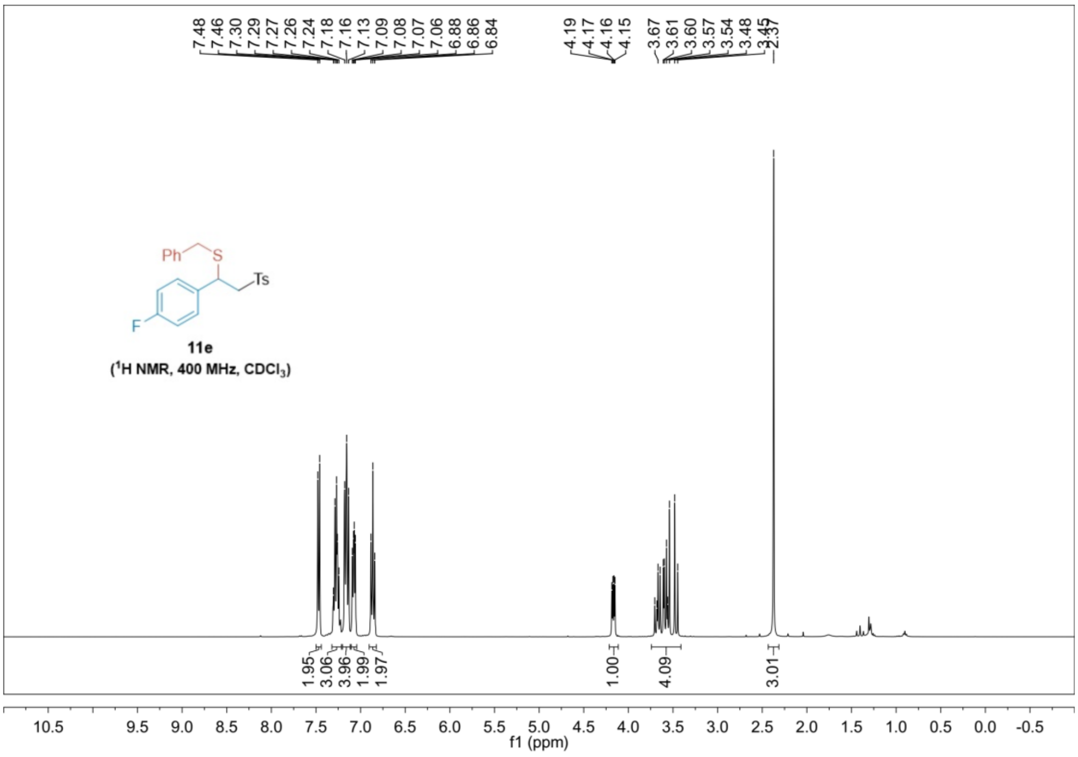

11e
(^{1}H NMR, 400 MHz, CDCl$_3$)

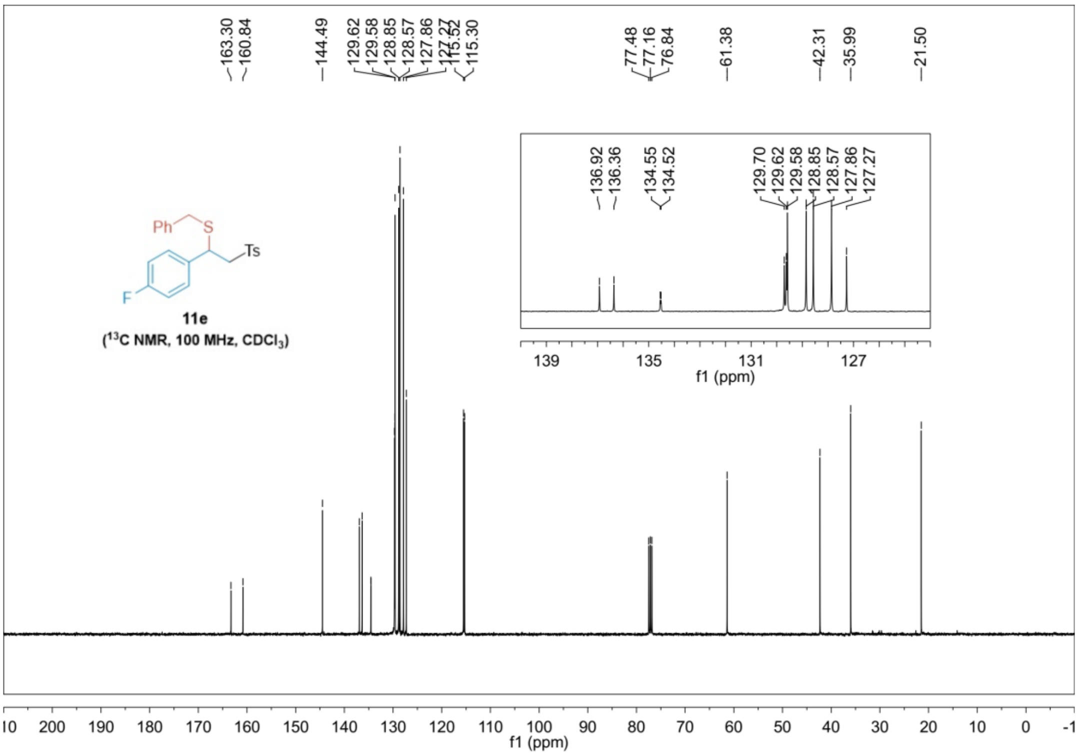

163.30
160.84
144.49
129.62
129.58
128.85
128.57
127.86
127.27
115.52
115.30
77.48
77.16
76.84
61.38
42.31
35.99
21.50
136.92
136.36
134.55
134.52
129.70
129.62
129.58
128.85
128.57
127.86
127.27
139
135
131
127
f1 (ppm)
Ph
S
Ts
F
11e
(^{13}C NMR, 100 MHz, CDCl$_3$)
200
190
180
170
160
150
140
130
120
110
100
90
80
70
60
50
40
30
20
10
0
f1 (ppm)

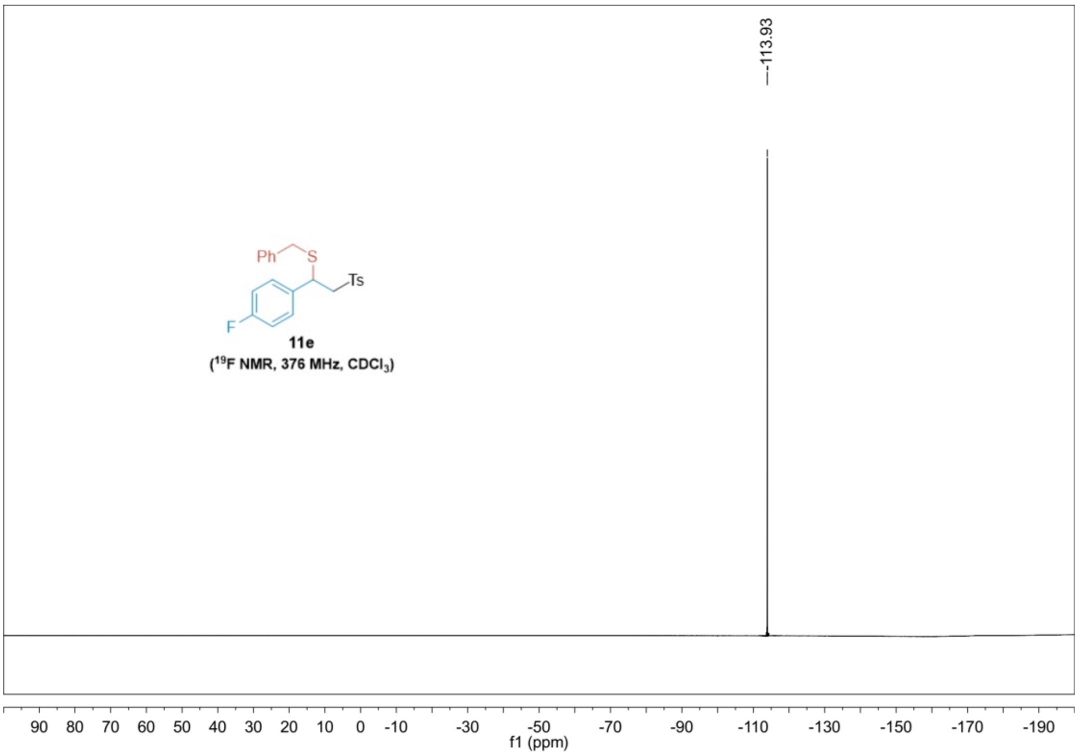

-113.93
Ph
S
Ts
F
11e
(^{19}F NMR, 376 MHz, CDCl$_3$)
90
80
70
60
50
40
30
20
10
0
-10
-30
-50
-70
-90
-110
-130
-150
-170
-190
f1 (ppm)

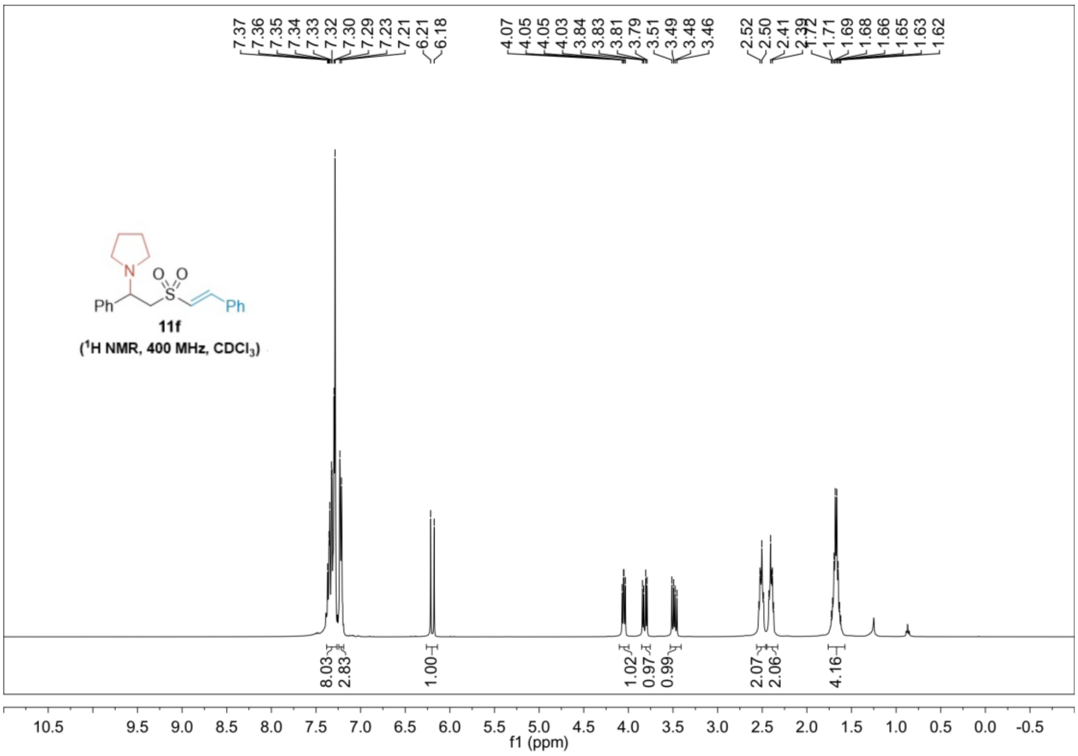
7.37 7.36 7.35 7.34 7.33 7.32 7.30 7.29 7.23 7.21 6.21 6.18
4.07 4.05 4.05 4.03 3.84 3.83 3.81 3.79 3.51 3.49 3.48 3.46
2.52 2.50 2.41 2.39 1.72 1.71 1.69 1.68 1.66 1.65 1.63 1.62
Ph
Ph
11f
(¹H NMR, 400 MHz, CDCl₃)
8.03 2.83 1.00 1.02 0.97 0.99 2.07 2.06 4.16
10.5 9.5 9.0 8.5 8.0 7.5 7.0 6.5 6.0 5.5 5.0 4.5 4.0 3.5 3.0 2.5 2.0 1.5 1.0 0.5 0.0 -0.5
f1 (ppm)

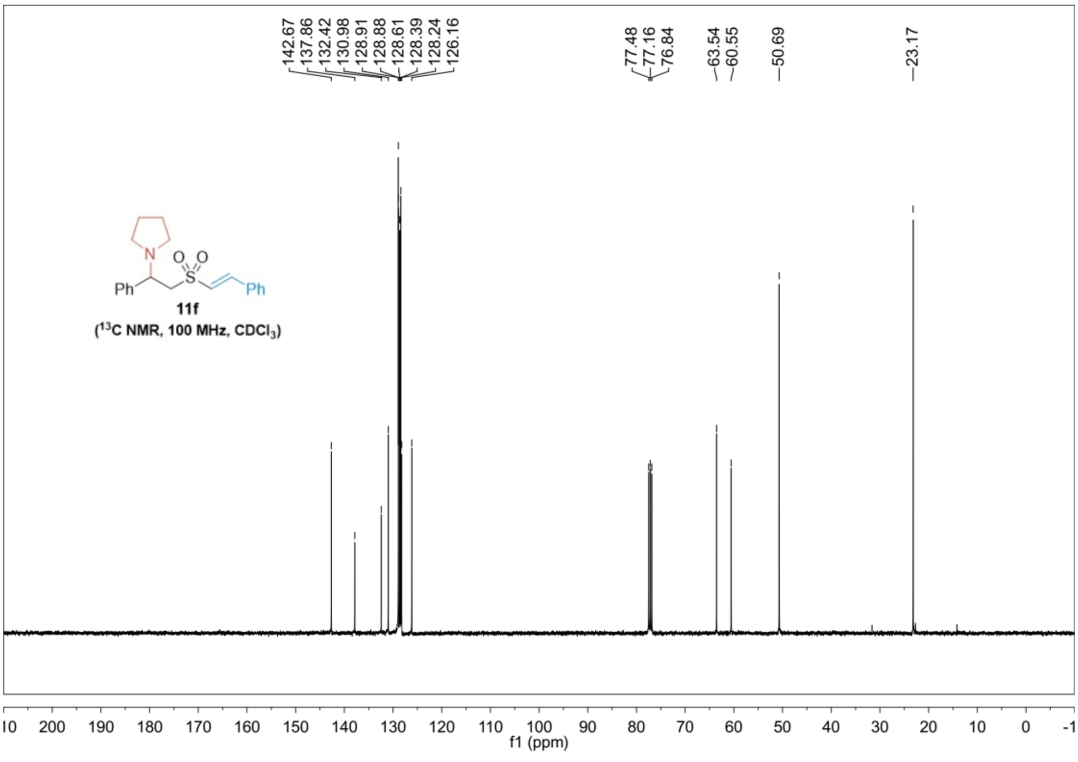
142.67 137.86 132.42 130.98 128.91 128.88 128.61 128.39 128.24 126.16
77.48 77.16 76.84
63.54 60.55 50.69 23.17
Ph
Ph
11f
(¹³C NMR, 100 MHz, CDCl₃)
200 190 180 170 160 150 140 130 120 110 100 90 80 70 60 50 40 30 20 10 0
f1 (ppm)

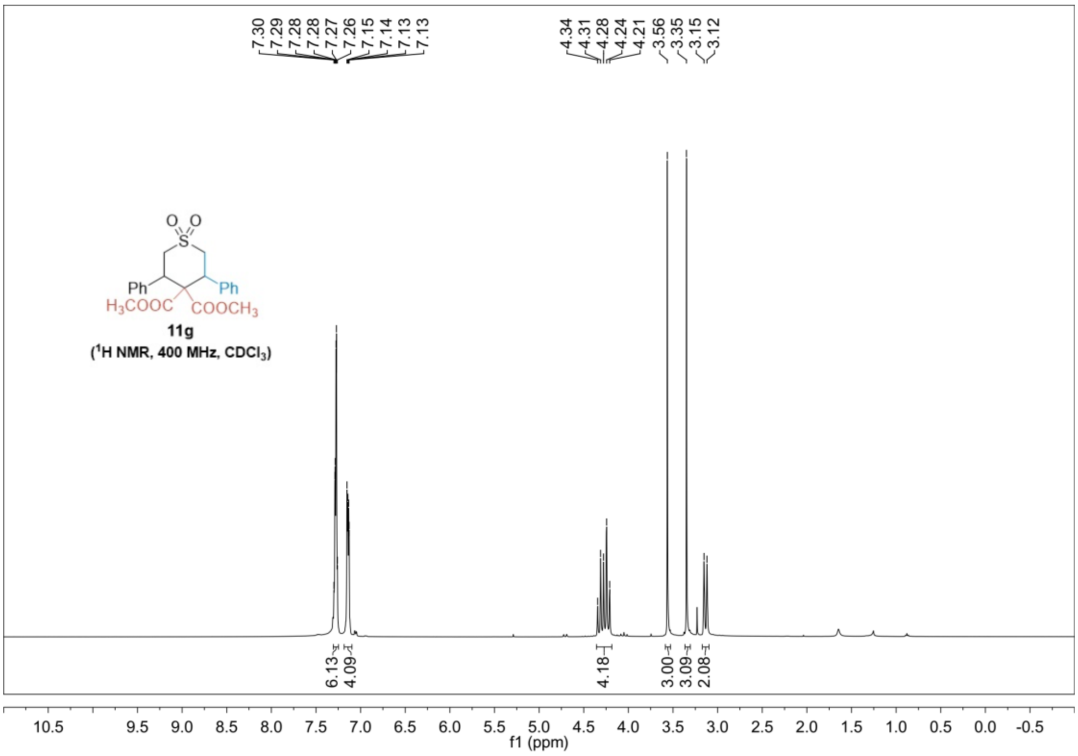
7.30
7.29
7.28
7.28
7.27
7.26
7.15
7.14
7.13
7.13
4.34
4.31
4.28
4.24
4.21
3.56
3.35
3.15
3.12
Ph
Ph
H3COOC
COOCH3
11g
(1H NMR, 400 MHz, CDCl3)
6.13
4.09
4.18
3.00
3.09
2.08
f1 (ppm)

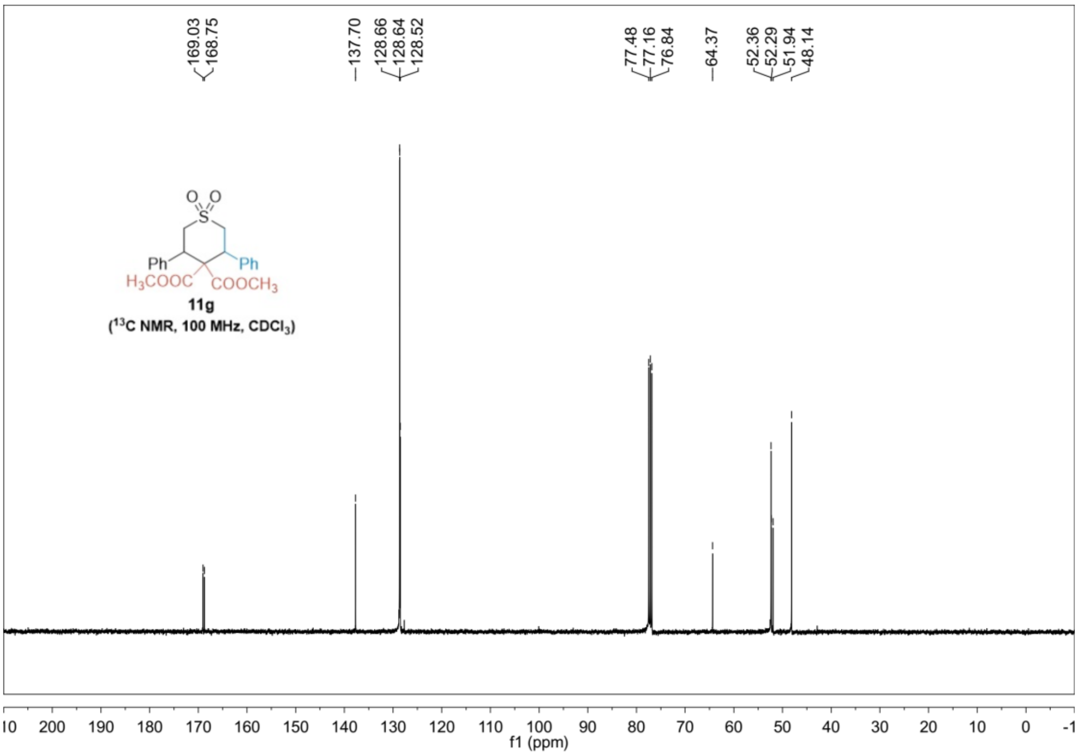
169.03
168.75
137.70
128.66
128.64
128.52
77.48
77.16
76.84
64.37
52.36
52.29
51.94
48.14
Ph
Ph
H3COOC
COOCH3
11g
(13C NMR, 100 MHz, CDCl3)
f1 (ppm)

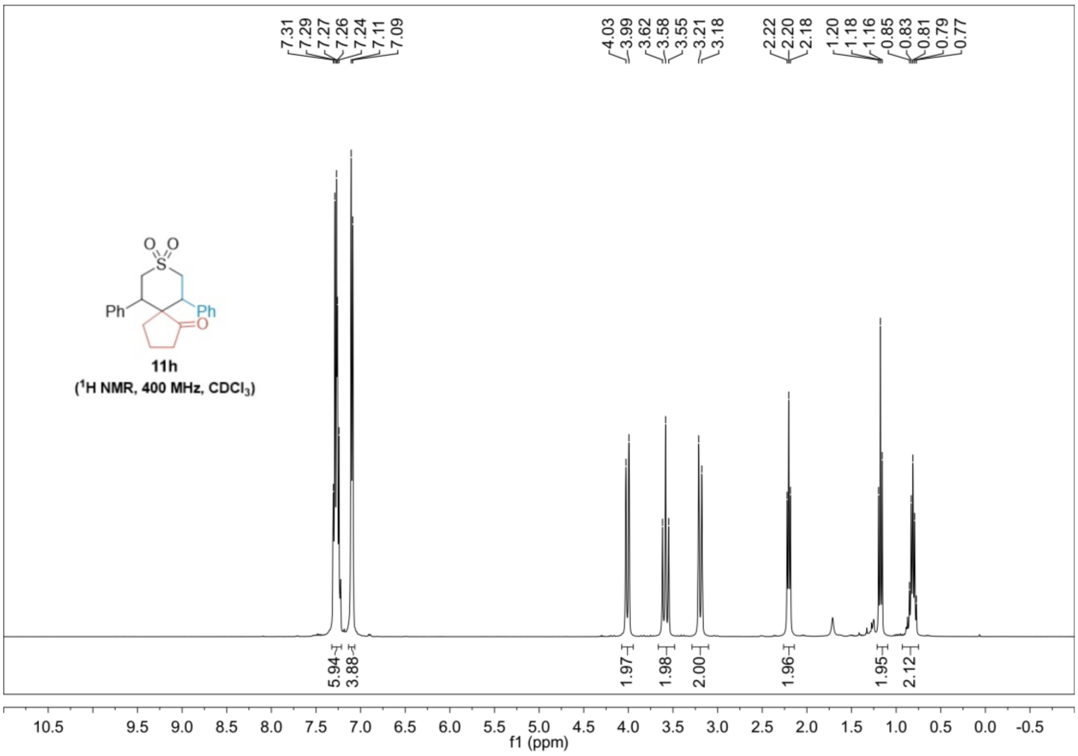
7.31
7.29
7.27
7.26
7.24
7.11
7.09
4.03
3.99
3.62
3.58
3.55
3.21
3.18
2.22
2.20
2.18
1.20
1.18
1.16
0.85
0.83
0.81
0.79
0.77
Ph
Ph
O
11h
(1H NMR, 400 MHz, CDCl3)
5.94
3.88
1.97
1.98
2.00
1.96
1.95
2.12
f1 (ppm)

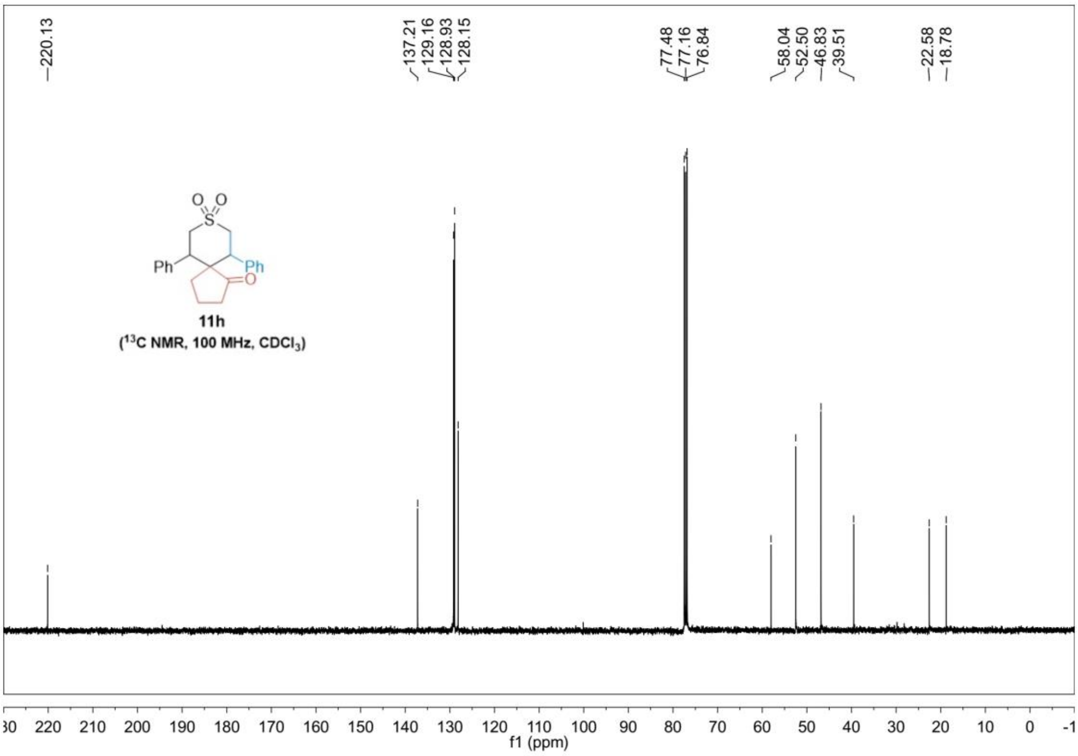
220.13
137.21
129.16
128.93
128.15
77.48
77.16
76.84
58.04
52.50
46.83
39.51
22.58
18.78
Ph
Ph
O
11h
(13C NMR, 100 MHz, CDCl3)
f1 (ppm)

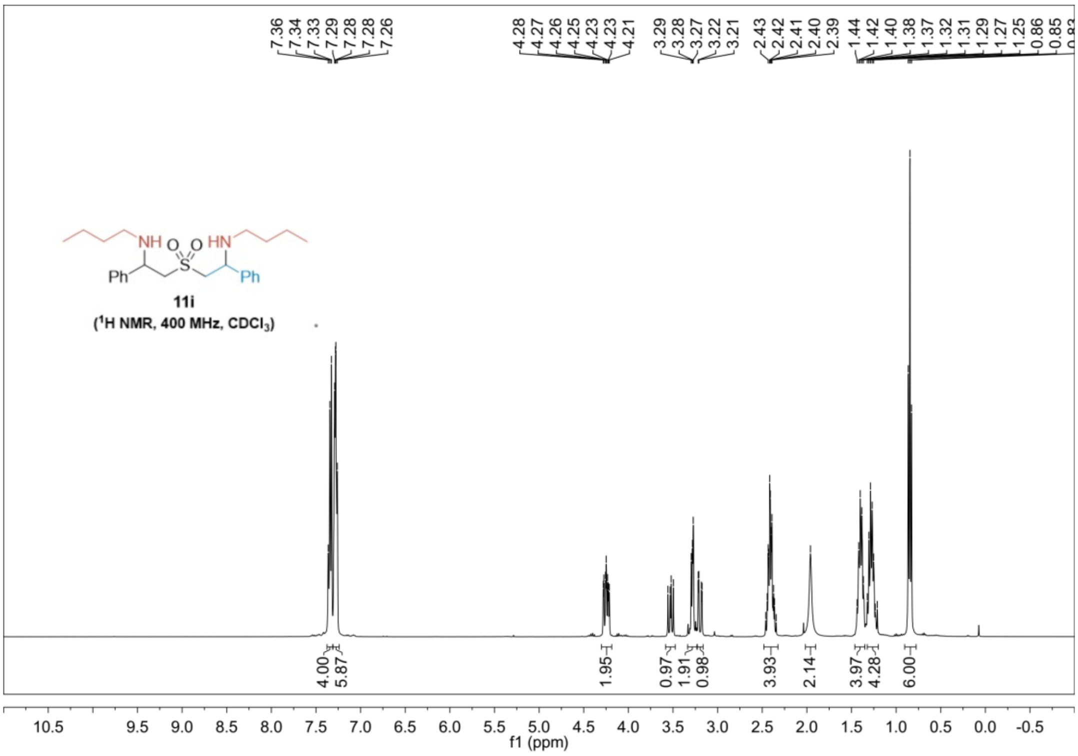
11i
(1H NMR, 400 MHz, CDCl3)
f1 (ppm)

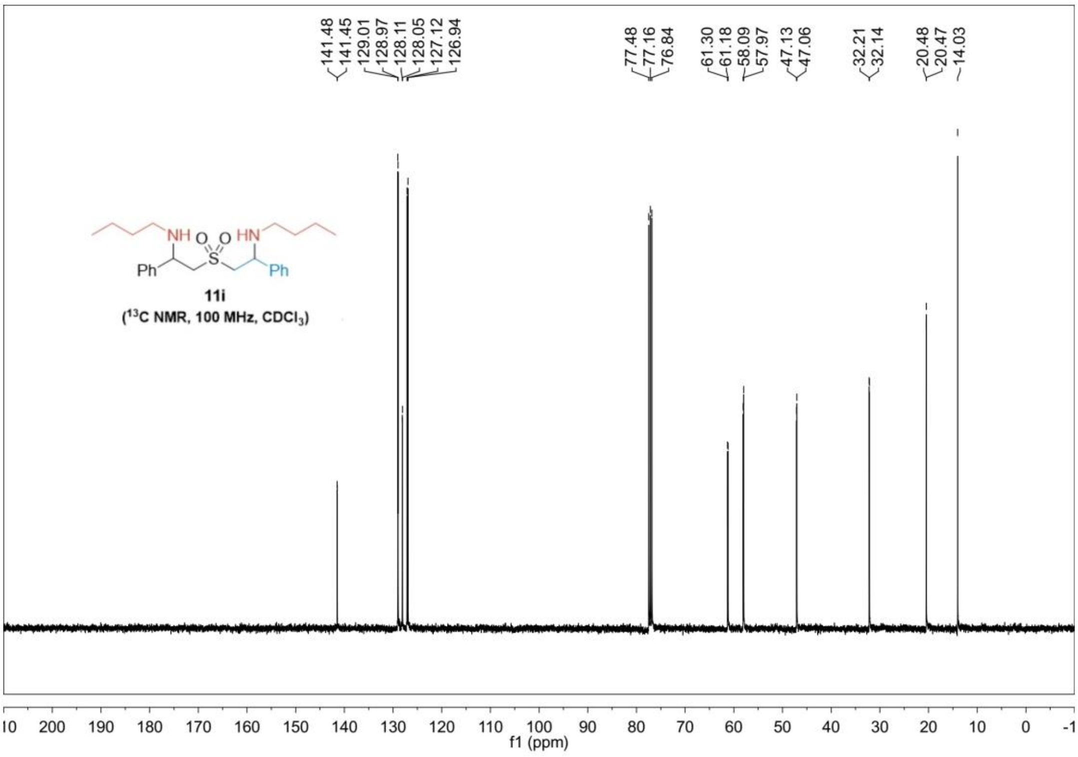
11i
(13C NMR, 100 MHz, CDCl3)
f1 (ppm)

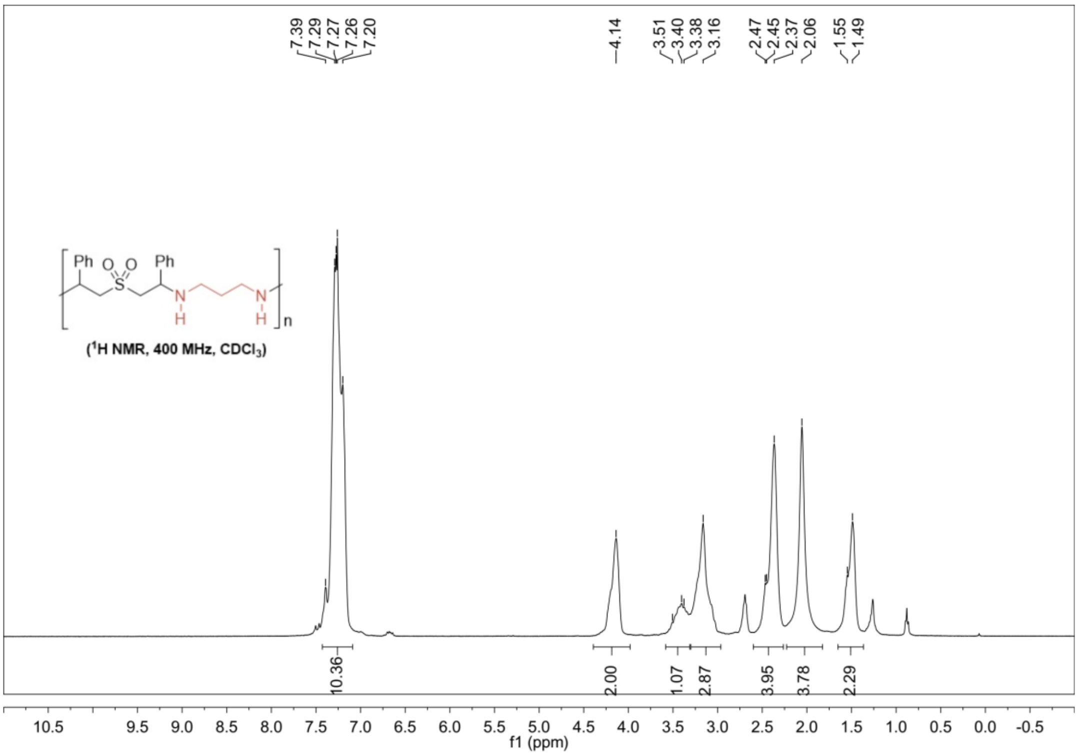
7.39
7.29
7.27
7.26
7.20
4.14
3.51
3.40
3.38
3.16
2.47
2.45
2.37
2.06
1.55
1.49
Ph
O O
S
Ph
N
H
N
H
n
(1H NMR, 400 MHz, CDCl3)
10.36
2.00
1.07
2.87
3.95
3.78
2.29
10.5 9.5 9.0 8.5 8.0 7.5 7.0 6.5 6.0 5.5 5.0 4.5 4.0 3.5 3.0 2.5 2.0 1.5 1.0 0.5 0.0 -0.5
f1 (ppm)

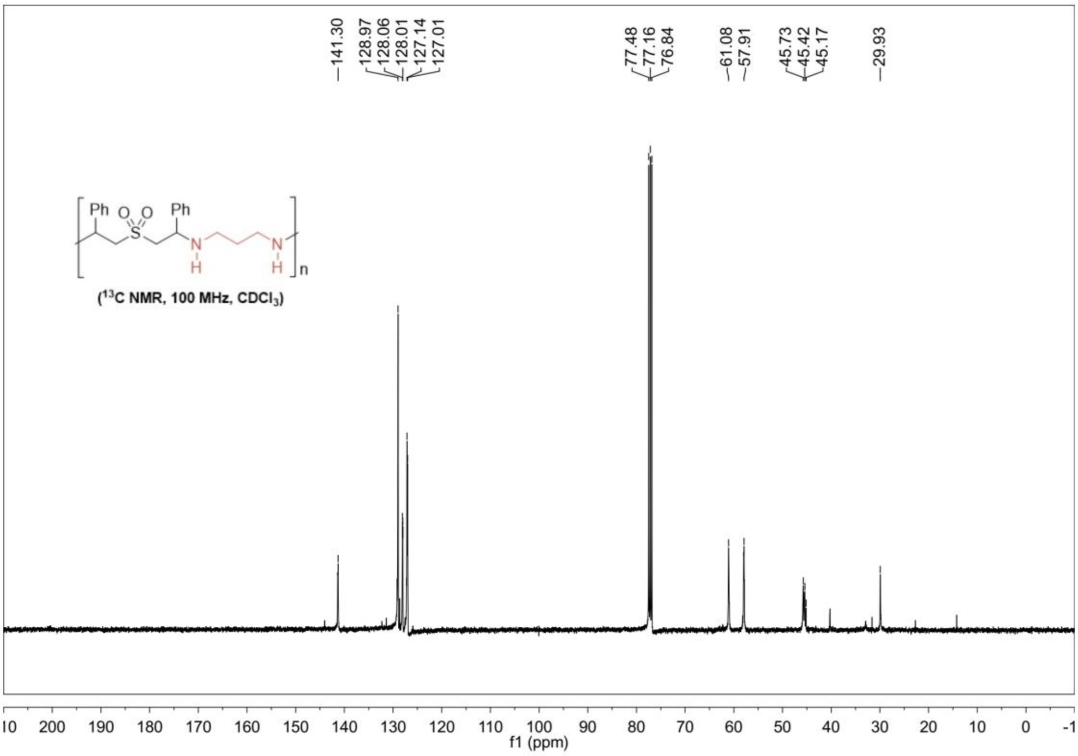
141.30
128.97
128.06
128.01
127.14
127.01
77.48
77.16
76.84
61.08
57.91
45.73
45.42
45.17
29.93
Ph
O O
S
Ph
N
H
N
H
n
(13C NMR, 100 MHz, CDCl3)
200 190 180 170 160 150 140 130 120 110 100 90 80 70 60 50 40 30 20 10 0
f1 (ppm)

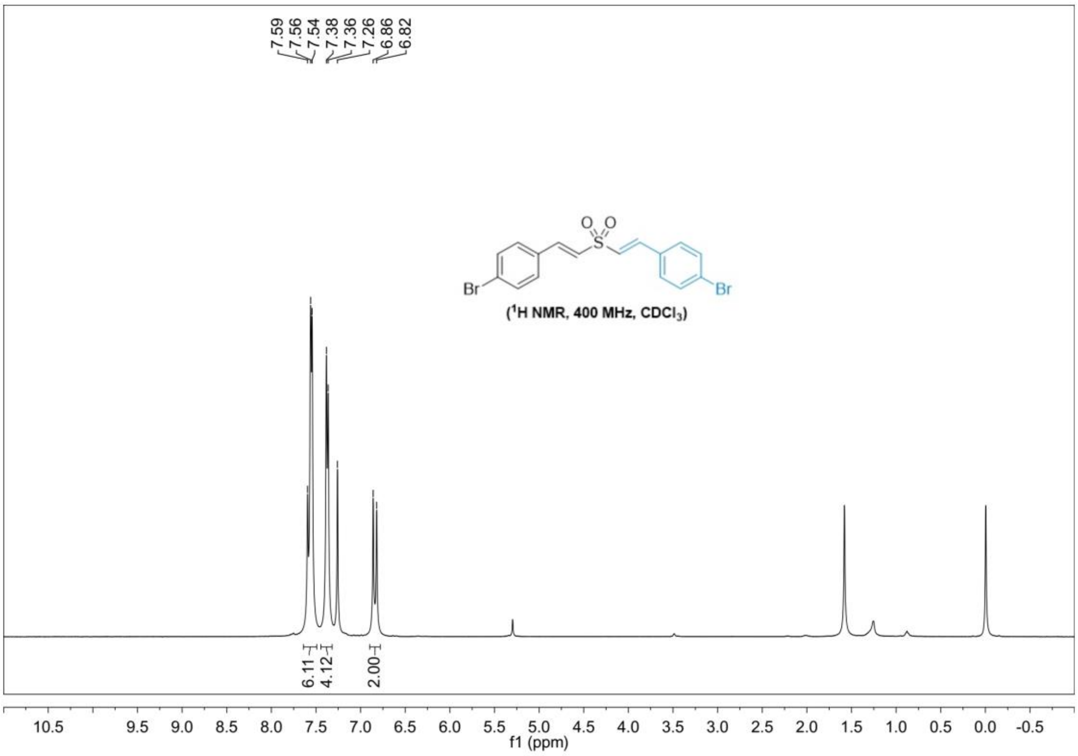

7.59
7.56
7.54
7.38
7.36
7.26
6.86
6.82
Br
O O
S
Br
(1H NMR, 400 MHz, CDCl3)
6.11
4.12
2.00
10.5 9.5 9.0 8.5 8.0 7.5 7.0 6.5 6.0 5.5 5.0 4.5 4.0 3.5 3.0 2.5 2.0 1.5 1.0 0.5 0.0 -0.5
f1 (ppm)

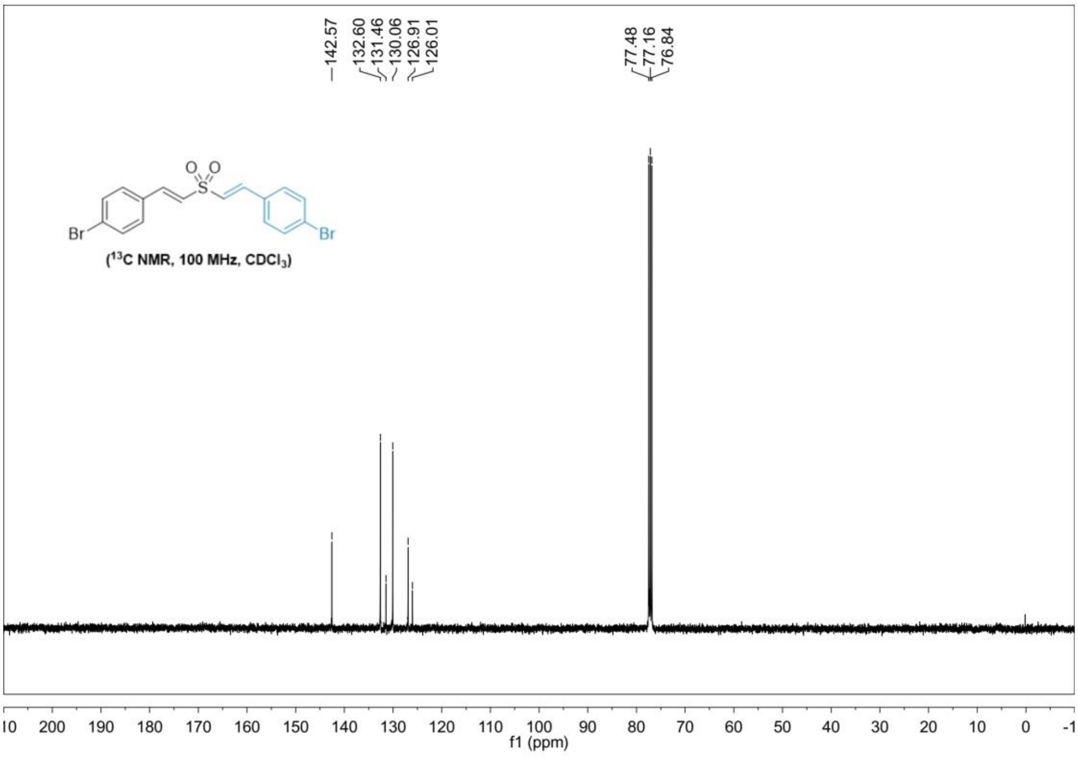

142.57
132.60
131.46
130.06
126.91
126.01
77.48
77.16
76.84
Br
O O
S
Br
(13C NMR, 100 MHz, CDCl3)
200 190 180 170 160 150 140 130 120 110 100 90 80 70 60 50 40 30 20 10 0
f1 (ppm)

Section 8. Supplementary references

1. Dong, J., Krasnova, L., Finn, M. G., Sharpless, K. B. Sulfur (VI) Fluoride Exchange (SuFEx): Another Good Reaction for Click Chemistry. *Angew. Chem. Int. Ed.* **2014**, *53*, 9430-9448.

2. Zhong, T., Pang, M. K., Chen, Z.-D. Copper-free Sandmeyer-type Reaction for the Synthesis of Sulfonyl Fluorides. *Org. Lett.* **2020**, *22*, 3072-3078.

3. Davies, A. T., Curto, J. M., Bagley, S. W. One-pot Palladium-Catalyzed Synthesis of Sulfonyl Fluorides from Aryl Bromides. *Chem. Sci*. **2017**, *8*, 1233-1237.

4. Wang, L., Cornella, J. A Unified Strategy for Arylsulfur (VI) Fluorides from Aryl Halides: Access to Ar-SOF_3 Compounds. *Angew. Chem. Int. Ed.* **2020**, *59*, 23510-23515.

5. Magre, M., Cornella, J. Redox-Neutral Organometallic Elementary Steps at Bismuth: Catalytic Synthesis of Aryl Sulfonyl Fluorides. *J. Am. Chem. Soc.* **2021**, *143*, 21497-21502.

6. Nielsen, M. K., Ugaz, C. R., Li, W., Doyle, A. G. PyFluor: A Low-Cost, Stable, and Selective Deoxyfluorination Reagent. *J. Am. Chem. Soc.* **2015**, *137*, 9571-9574.

7. Matesic, L., Wyatt, N. A., Fraser, B. H., Roberts, M. P., Pham, T. Q., Greguric, I. Ascertaining the Suitability of Aryl Sulfonyl Fluorides for [^{18}F] Radiochemistry Applications: A Systematic Investigation Using Microfluidics. *J. Org. Chem.* **2013**, *78*, 11262-11270.

8. Chatelain, P., Muller, C., Sau, A., Brykczynska, D., Bahadori, M., Rowley, C. N., Moran, J. Desulfonative Suzuki-Miyaura Coupling of Sulfonyl Fluorides. *Angew. Chem. Int. Ed.* **2021**, *60*, 25307-25312.

9. Pagire, S. K., Paria, S., Reiser, O. Synthesis of *β*-Hydroxysulfones from Sulfonyl Chlorides and Alkenes Utilizing Visible Light Photocatalytic Sequences. *Org. Lett.* **2016**, *18*, 2106-2109.

10. Song, C.-X., Chen, P., Tang, Y. Carboxylation of Styrenes with CBr_4 and DMSO via Cooperative Photoredox and Cobalt Catalysis. *RSC Adv.* **2017**, *7*, 11233-11243.

11. Movahhed, S., Westphal, J., Dindaroglu, M., Falk, A., Schmalz, H. G. Low-Pressure Cobalt-Catalyzed Enantioselective Hydrovinylation of Vinylarenes. *Chem. Eur. J.* **2016**, *22*, 7381-7384.

12. Zhang, M., Chen, M., Ding, X. The Photoredox-Catalyzed Hydrosulfamoylation of Styrenes and its Application in the Novel Synthesis of Naratriptan. *Chem. Commun.* **2021**, *57*, 9140-9143.

13. Molloy, J. J., Seath, C. P., West, M. J., McLaughlin, C., Fazakerley, N. J., Kennedy, A. R., Nelson, D. J., Watson, A. J. B. Interrogating Pd(II) Anion Metathesis Using a Bifunctional Chemical Probe: A Transmetalation Switch. *J. Am. Chem. Soc.* **2018**, *140*, 126-130.

14. Lei, C., Yip, Y. J., Zhou, J. S. Nickel-Catalyzed Direct Synthesis of Aryl Olefins from Ketones and Organoboron Reagents under Neutral Conditions. *J. Am. Chem. Soc.* **2017**, *139*, 6086-6089.

15. Zhao, W., Gockel, S., Hull, K. Anti-Markovnikov Hydro(amino)alkylation of Vinylarenes via Photoredox Catalysis. *Nat. Commun.* **2021**, *12*, 5956.

16. Julia, F., Yan, J., Paulus, F., Ritter, T. Vinyl Thianthrenium Tetrafluoroborate: A Practical and Versatile Vinylating Reagent Made from Ethylene. *J. Am. Chem. Soc.* **2021**, *143*, 12992-12998.

17. Zha, G.-F., Zheng, Q., Leng, J., Wu, P., Qin, H.-L., Sharpless, K. B. Palladium-Catalyzed Fluorosulfonylvinylation of Organic Iodides. *Angew. Chem. Int. Ed.* **2017**, *56*, 4849-4852.

18. Zha, G.-F., Bare, G. A. L., Leng, J., Shang, Z.-P., Luo, Z., Qin, H.-L. Gram-Scale Synthesis of β-(Hetero)Arylethenesulfonyl Fluorides via a $Pd(OAc)_2$ Catalyzed Oxidative Heck Process with DDQ or $AgNO_3$ as an Oxidant. *Adv. Synth. Catal.* **2017**, *359*, 3237-3242.

19. Wang, P., Zhang, H.-H., Nie, X.-L., Xu, T.-X., Liao, S.-H. Photoredox Catalytic Radical Fluorosulfonylation of Olefins Enabled by a Bench-Stable Redox-Active Fluorosulfonyl Radical Precursor. *Nat. Commun.* **2022**, *13*, 3370.
20. Xu, R.-T., Xu, T.-T., Yang, M.-C., Cao, T.-P., Liao, S.-H. A Rapid Access to Aliphatic Sulfonyl Fluorides. *Nat. Commun.* **2019**, *10*, 3752.
21. Chen, Z.-T., Zhou, X., Yi, J.-T., Diao, H.-J., Chen, Q.-L., Lu, G., Weng, J. Catalytic Decarboxylative Fluorosulfonylation Enabled by Energy-Transfer-Mediated Photocatalysis. *Org. Lett.* **2022**, *24*, 2474-2478.
22. Nie, X., Xu, T., Song, J., Devaraj, A., Zhang, B., Chen, Y., Liao, S. Radical Fluorosulfonylation: Accessing Alkenyl Sulfonyl Fluorides from Alkenes. *Angew. Chem. Int. Ed.* **2021**, *60*, 3956-3960.
23. Vogel, J., Hammami, R., Ko, A., Datta, H., Eiben, Y., Labenne, K., McCarver, E., Yilmaz, E., Melvin, P. Synthesis of Highly Reactive Sulfone Iminium Fluorides and Their Use in Deoxyfluorination and Sulfur Fluoride Exchange Chemistry. *Org. Lett.* **2022**, *24*, 5962−5966.
24. Mukherjee, H., Debreczeni, J., Breed, J., Tentarelli, S., Aquila, B., Dowling, J. E., Whitty, A., Grimste, N. P. A. A Study of the Reactivity of $S^{(VI)}$–F Containing Warheads with Nucleophilic Amino-acid Side Chains under Physiological Conditions. *Org. Biomol. Chem.* **2017**,*15*, 9685-9695.
25. Kowalczyk, R., Edmunds, A. J. F., Hall, R., Bolm, C. Synthesis of CF_3-Substituted Sulfoximines from Sulfonimidoyl Fluorides. *Org. Lett.* **2011**, *13*, 768-771.
26. Guo, J.-K., Kuang, G.-W., Rong, J., Li, L.-C., Ni, C.-F., Hu, J.-B. Rapid Deoxyfluorination of Alcohols with *N*-Tosyl-4-chlorobenzenesulfonimidoyl Fluoride (SulfoxFluor) at Room Temperature. *Chem. Eur. J.* **2019**, *25*, 7259-7264.
27. Zhang, G.-F., Xu, S.-J., Xie, X.-Q., Ding, C.-R., Shan, S. Direct Synthesis of *N*-Sulfinyl- and *N*-Sulfonylimines via Copper/L-proline-catalyzed Aerobic Oxidative Cascade Reaction of Alcohols with Sulfinamides or Sulfonamides. *RSC adv.* **2017**, *7*, 9431-9435.
28. Aota, Y., Kano, T., Maruoka, K. Asymmetric Synthesis of Chiral Sulfoximines through the *S*-Alkylation of Sulfinamides. *Angew. Chem. Int. Ed.* **2019**, *58*, 17661-17665.
29. Xu, Y., Tang, X., Hu, W., Wu, W., Jiang, H. Transition-metal-free Synthesis of Vinyl Sulfones via Tandem Cross-Decarboxylative/Coupling Reactions of Sodium Sulfinates and Cinnamic Acids. *Green Chem.* **2014**, *16*, 3720-3723.
30. Li, F., Zhang, G., Liu, Y., Zhu, B., Leng, Y., Wu, J. Cu-Catalyzed Dehydrogenative Olefinsulfonation of Alkyl Arenes. *Org. Lett.* **2020**, *22*, 8791-8795.
31. Liu, L., Xue, P., Chen, Q., Wang, C. Copper-Catalyzed Heck-Type Couplings of Sulfonyl Chlorides with Olefins: Efficient and Rapid Access to Vinyl Sulfones. *Tetrahedron Lett.* **2021**, *80*, 153319.
32. Ratushnyy, M., Kamenova, M., Gevorgyan, V. A mild Light-Induced Cleavage of the S-O Bond of Aryl Sulfonate Esters Enables Efficient Sulfonylation of Vinylarenes. *Chem. Sci.* **2018**, *9*, 7193-7197.
33. Bos, P. H., Minnaard, A. J. Feringa, B. L. Catalytic Asymmetric Conjugate Addition of Grignard Reagents to α, β-Unsaturated Sulfones *Org. Lett.* **2008**, *10*, 4219-4222.
34. Tang, S., Wu, Y., Liao, W., Bai, R., Liu, C., Lei, A. Revealing the Metal-Like Behavior of Iodine: An Iodide-Catalysed Radical Oxidative Alkenylation. *Chem. Commun.* **2014**, *50*, 4496-4499.
35. Zhang, G., Fu, J. G., Zhao, Q., Zhang, G. S., Li, M. Y., Feng, C. G., Lin, G. Q. Silver-Promoted Synthesis of Vinyl Sulfones from Vinyl Bromides and Sulfonyl Hydrazides in Water. *Chem. Commun.* **2020**, *56*, 4688-4691.

36. Zhang, G., Zhang, L., Yi, H., Luo, Y., Qi, X., Tung, C.-H., Wu, L.-Z., Lei, A. Visible-Light Induced Oxidant-Free Oxidative Cross-Coupling for Constructing Allylic Sulfones from Olefins and Sulfinic acids. *Chem. Commun.* **2016**, *52*, 10407-10410.
37. Hiroko, Y., Dagmar, K., Stephan, G., Otto, H-H. 1,4-Pentadien-3-ones, XXIV. A Convenient Synthesis of Substituted Distyryl Sulfones. *Archiv. der. Pharmazie*, **1985**, *318*, 208-282.
38. Chang, M.-Y., Wu, Y.-S., Hsiao, Y.-T. Gram-Scale Synthesis of β-Sulfonyl Styrenes. *Synthesis* **2018**, *50*, A–H.
39. Wang, Q. L., Zhang, W. Z., Zhou, Q., Zhou, C.-S., Xiong, B.-Q., Tang, K.-W., Liu, Y. Visible-Light-Mediated Difunctionalization of Vinylcyclopropanes for the Synthesis of 1-Sulfonylmethyl-3,4-Dihydronaphthalenes. *Org. Biomol. Chem.* **2019**, *17*, 7918-7926.
40. Leibler, I. N., Tekle-Smith, M. A., Doyle, A. G. A General Strategy for C(sp^3)-H Functionalization with Nucleophiles Using Methyl Radical as a Hydrogen Atom Abstractor. *Nat. Commun.* **2021**, *12*, 6950.
41. Alič, B., Tavčar, G. Reaction of N-heterocyclic Carbene (NHC) with Different HF Sources And Ratios-A Free Fluoride Reagent Based on Imidazolium Fluoride. *J. Fluorine Chem.* **2016**, *192*, 141-146.
42. Roth, H. G., Romero, N. A., Nicewicz, D. A. Experimental and Calculated Electrochemical Potentials of Common Organic Molecules for Applications to Single-Electron Redox Chemistry. *Synlett.* **2016**, *27*, 714-723.
43. Padmaja, A., Reddy, K. R., Reddy, N. S., Reddy, D. B. Phase Transfer Catalyzed Cyclopropanation of Trans-Styryl Aryl Sulfones with Dimethyl Sulfoxonium Methylide. *Phosphorus, Sulfur, and Silicon and the Related Elements* **2006**, *152*, 91-98.
44. Ge, Q.-Q., Qian, J.-S., Xuan, J. Electron Donor-Acceptor Complex Enabled Decarboxylative Sulfonylation of Cinnamic Acids under Visible-Light Irradiation. *J. Org. Chem.* **2019**, *84*, 8691-8701.
45. Padmavathi, V., Subbaiah, D., Reddy, B. Double Michael Addition Reactions to Tetrakis Sulfonyl Activated Olefins. *J. Heterocyclic Chem.* **2005**, *42*, 255-258.
46. Topchiy, M. A., Asachenko, A. F., Nechaev, M. S. Solvent-Free Buchwald-Hartwig Reaction of Aryl and Heteroaryl Halides with Secondary Amines. *Eur. J. Org. Chem.* **2014**, *16*, 3319-3322.
47. Monçalves, M., Rampon, D. d. S., Schneider, P. H., Rodembusch, F. S., Silveira, C. d. C. Divinyl Sulfides/Sulfones-Based D–π–A–π–D Dyes as Efficient non-Aromatic Bridges for π-Conjugated Compounds. *Dyes Pigm.* **2014**, *102*, 71-78.
